# Presenting a Framework to Professionalize Health Supply Chain Management

**DOI:** 10.9745/GHSP-D-23-00119

**Published:** 2025-05-09

**Authors:** Andrew N. Brown, Barry Chovitz, Richard dos Santos, Michael Egharevba, Bridget McHenry, Erin Meier, Dominique Zwinkels

**Affiliations:** a Management Sciences for Health, Canberra, Australia.; b Global Health Supply Chain Program-Procurement Supply Management Project, Chemonics International, Washington, DC, USA.; c People that Deliver, Copenhagen, Denmark.; d Global Health Supply Chain Program-Procurement Supply Management Project, IntraHealth International, Washington, DC, USA.; e Global Health Training, Advisory, and Support Contract, a Public Health Institute contract for U.S. Agency for International Development, Washington, DC, USA.; f IntraHealth International, Chapel Hill, NC, USA.; g People that Deliver/UNICEF, Copenhagen, Denmark.

## Abstract

**Introduction:**

Many countries have an insufficient supply of adequately skilled supply chain workers to manage health commodities, and no global standards in education or experience exist for the supply chain management (SCM) workforce managing health products. We present a professionalization framework for the health SCM workforce that provides a systematic process that countries can use to standardize and elevate the health SCM profession.

**Methods:**

In 2019, semistructured interviews were conducted with individuals from 10 leading organizations supporting in-country public health supply chains to explore approaches for an SCM professionalization framework. Interview data were analyzed using direct thematic analysis. Findings were then validated through a validation workshop with 11 individuals from 8 leading SCM organizations.

**Results:**

Four associated components of this framework were developed: (1) The Library of Competencies and Designations contains management competencies grouped in 7 domains and supply chain technical competencies assigned to 5 professional designations; (2) The Collection of Roles and Job Descriptions contains 96 sample job descriptions; (3) The Mapping of Education displays the education offerings relevant to each competency across the 5 professional designations; and (4) The Implementation Approach for Health Supply Chains leads project teams to systematically apply these 3 tools in the supply chain context of a country.

**Conclusion:**

The SCM Professionalisation Framework provides a valuable tool to increase the supply of and demand for health SCM workers, increasing the recognition and use of SCM professionals within national health systems. Its utilization is a critical step in addressing the current workforce gap, particularly in low- and middle-income countries, and ensuring that the health SCM workforce possesses the right competencies, skills, and qualifications to fulfill its roles. The comprehensive framework can be used by governments, employers, and education institutions to define and align SCM professional standards, competencies, and curricula with job requirements.

## Abstract (precis)

The Supply Chain Management (SCM) Professionalisation Framework—a valuable tool to initiate awareness and advocacy in recognizing SCM professionals within national health systems—can be used to define and align SCM professional standards, competencies, and curricula, thus strengthening the labor market for health SCM professionals.

## Teaser

**Key Findings:**

- In many countries, an adequate supply of workers who have the competencies required for effective supply chain management (SCM) is not available.
- The lack of professional standards for health SCM workers can impact workforce planning and limit workforce development initiatives and programs.
- By clearly defining the pathways for developing competencies across different professional designations, this 4-part SCM professionalization framework offers a blueprint for aligning education, training, and workforce development efforts to meet health SCM needs effectively.

**Key Implications:**

- Applying the SCM Professionalisation Framework in a country context is a critical step toward ensuring the health SCM workforce possesses the right competencies, skills, and qualifications to fulfill its roles. Applying this framework is expected to improve the availability and use of health SCM professionals.
- By offering a standardized approach to health SCM professionalization, this framework provides stakeholders seeking to generate important changes in the health system labor market with a starting point to focus their efforts and a holistic approach to pursue.

## INTRODUCTION

Health supply chain management (HSCM) is a key enabler for achieving universal health coverage, as a reliable supply of quality, affordable medicines is required for increased service coverage.^1^^,^^2^ The health supply chain personnel managing and delivering essential medicines and other health commodities require substantial technical and managerial capacities to operate complex supply chain systems effectively.^3^^–^^5^ In many countries, an adequate supply of workers with the competencies required for effective supply chain management (SCM) is not available. Insufficient numbers of competent staff can lead to poor performance of the supply chain system.^6^^–^^10^

Many countries mandate that staff managing health commodities have a medical certification.^11^^–^^14^ Very few countries regulate or have established standards in education or experience for HSCM professionals,^3^^,^^15^ and there are no formal requirements that define supply chain competency. In low- and middle-income countries (LMICs), the staff assigned to HSCM positions are almost always clinical staff, who have received only short-term training in HSCM, and as they rotate and migrate, staffing gaps open.^4^^,^^6^^,^^16^^,^^17^ The lack of professional standards for HSCM workers can impact workforce planning and limit workforce development initiatives and programs. Without professionalization, the skills and competencies required for HSCM are neither recognized nor defined,^3^ and without the presence of a local supply chain professional body, the domain of SCM is often overlooked, especially within the context of health professionals. Without a recognized professional group for HSCM and no presence of professional associations or councils for HSCM workers, the oversight, education, and production of qualified workers are limited. For example, formal educational qualifications, credentialing processes, and professional development opportunities are reduced.^3^^,^^6^

Job descriptions for HSCM positions do not accurately capture the SCM components,^18^ impacting demand for skilled HSCM workers. Job descriptions also lack standardized process competence metrics, which can limit necessary skills-building opportunities, such as harmonized learning offerings and credentialing. Without a formal career progression path, coupled with unstructured formal learning options, HSCM workers are short-lived in many supply chain positions. As a result, SCM workforce positions that exist within the public health supply chain of LMICs often do not require the relevant SCM qualifications.

Prior approaches to develop the HSCM workforce have focused on building capacity at the individual level (e.g., through skills training and improved supervision) and the organizational level (e.g., through improved organizational strategies, methods, or bodies for HSCM) but have not adequately addressed various elements at the national level, such as the policies on national education requirements or professional designations for HSCM professions. A systematic approach is needed to advocate the required change in HSCM professionals and to ensure they have grounding according to the defined competencies in SCM. Previous work has called for a framework to support the establishment of supply chain roles as a profession within human resources for health.^18^^–^^20^ Previous efforts supporting professionalization of the HSCM workforce raised awareness of the need to professionalize established global coordination coalitions and took first steps at the country level in a limited set of countries to strengthen institutions.^20^ The CapacityPlus Project created a conceptual, lifecycle approach for professionalization of HSCM workers with 3 pillars: (1) education, (2) employment, and (3) support.^20^ That approach called for “formally recognized avenues for education, training, and certification; suitable job descriptions and ladders for career progression; structures such as associations for interacting with peers and communicating the needs of workers with a common voice; and opportunities for continuing professional development.”^20^ Although the conceptual framework supported advocacy for professionalization, it did not provide tools or a specific implementation approach. People that Deliver (PtD) has developed tools, resources, and approaches to improve supply chain management competencies in the existing HSCM workforce.^5^^,^^21^ The PtD Competency Compendium^5^ captured all competencies needed within a national health supply chain. However, PtD understood that without an implementation approach and a labor market perspective, this competency model alone would not be a sufficient mechanism to achieve professionalization. In addition, PtD introduced a Human Resources for SCM Theory of Change to understand the factors related to skills, staffing, motivation, and working conditions required to optimize workforce performance.^21^ However, a broader approach that considered the labor market for SCM workers was lacking. A locally accepted professionalization framework with an identified career path, competencies, and education standards is needed to increase the availability and use of supply chain professionals in a country context, thus catalyzing the labor market.

A systematic approach is needed to advocate the required change in HSCM professionals and ensure they have grounding according to the defined competencies in SCM.

Studies of the labor market for the HSCM workforce have indicated critical imbalances.^14^^,^^22^ There is a shortage of mid-level logistics management professionals in many emerging markets due to increasing levels of logistical activity, uncompetitive salaries and rewards,^1^ and technical advances outpacing the speed at which workers can develop the needed skills.^22^ In Rwanda, a qualitative analysis of the HSCM labor market identified that supply and skills of the health SCM workforce must be better aligned to match labor market demand.^14^ The results of this detailed, country-based assessment of the HSCM labor market identified the need for an approach that would catalyze the HSCM labor market and increase the supply of and demand for HSCM workers.^14^

A professionalization approach aims to expand the number and skills of HSCM workers and to institutionalize health supply chain professionals as a specific occupational group that is not necessarily linked to the pharmaceutical cadre.^19^^,^^20^ A framework is needed that provides a path for governments to create professional standards for HSCM workers so that HSCM is recognized as a profession with defined standards. This comprehensive framework can support governments, employers of HSCM workers, and training institutions producing these workers to remedy the imbalances in the HSCM labor market, particularly in LMICs. In terms of demand for these professionals, the framework can support employers to clearly define the HSCM competencies for distinct roles and each professional level. Conversely, when looking at the supply of these professionals, the framework can be used to ensure learning and teaching courses exist to produce workers with these competencies. Within this framework, a project implementation approach is needed to organize collaboration among the various actors to address the activities needed to catalyze the whole SCM labor market.

This article presents a professionalization framework for the SCM workforce responsible for health products supply. The SCM Professionalisation Framework contains 4 components that provide a foundation and a systematic process for countries to create standards for the HSCM profession in various country contexts (Supplement 1). The components include the Library of Competencies and Professional Designations (Supplement 2), the Mapping of Education for Health Supply Chains (Supplement 3), the Collection of Roles and Descriptions for Health Supply Chains(Supplement 4), and the Implementation Approach for Health Supply Chains (Supplement 5).

## METHODS

A qualitative approach was used to gather information and perspectives on the need for professionalizing HSCM workers, potential approaches to professionalization, what a professionalization framework should contain, and what country implementation could look like. A qualitative approach that engaged key stakeholders was thought to be the best approach to understand these issues in detail. PtD used a stepwise, iterative approach, described later, to develop this framework. Each step provided the data needed to conduct the next step, and we provided constant feedback and redirection as experts based on our experience developing and applying this framework.

### Understanding Approaches to Professionalizing the Health Supply Chain Management Workforce

PtD used semistructured interviews followed by a validation workshop to understand the need for and potential approaches to professionalizing supply chain workers in the public health sector. In 2019, 10 interviews were conducted with individuals from leading organizations supporting in-country public health supply chains, including donor organizations, international supply chain and logistics associations, and private health supply organizations (the list of organizations is included in Supplement 6). Interviewees were selected using purposive sampling to represent diverse aspects of the public and private health supply chain sectors from international and national perspectives, with a focus on LMICs. Selection was conducted via a call for participation by PtD, IntraHealth, the U.S. Agency for International Development (USAID) Global Health Supply Chain Program-Procurement and Supply Management project, USAID, SAPICS (the professional body for SCM in South Africa), and existing private sector networks that represented organizations involved in producing the supply of SCM workers or those with demand for SCM workers. Table 1 describes the characteristics of the interviewees. Of the 10 interviewees, 7 were in top-level management positions and 3 were middle-level personnel. Interviewees were employed at 2 private sector companies, 6 international nongovernmental organizations supporting public sector supply chains, and 1 national-level nongovernmental organization. The interview process captured perspectives on professionalizing the SCM workforce responsible for health products and gathered insights on how to implement an SCM professionalization framework. We conducted interviews remotely, in English, using an 11-question interview tool that we developed (Supplement 6). We pilot-tested the interview tool before use. Interviews lasted approximately 1 hour. No invitees refused to participate or dropped out. In interviews, we took notes and created digital voice recordings. Interview data were analyzed using direct thematic analysis. Findings, such as attitudes toward each question and themes, were then validated through a validation workshop.

**TABLE 1.** Participant Characteristics of the Qualitative Study

**Characteristic**	**No. (%)**
Interviews (N=10)	
Sex	
Male	3 (30.0)
Female	7 (70.0)
Professional level	
Upper management	7 (70.0)
Middle-level personnel	3 (30.0)
Organization type	
Private sector	2 (20.0)
Public sector	1 (10.0)
International NGO	6 (60.0)
National NGO	1 (10.0)
Validation workshop (N=11)	
Sex	
Male	5 (45.5)
Female	6 (54.5)
Professional level	
Upper management	8 (72.7)
Middle-level personnel	3 (27.3)
Organization type	
Private sector	3 (27.3)
Public sector	2 (18.2)
International NGO	5 (45.5)
National NGO	1 (9.0)

Abbreviation: NGO, nongovernmental organization.

The validation workshop was conducted to compare other SCM professional association approaches and the existing PtD compendium. The workshop also aimed to work toward a consensus on what a professionalization framework may look like and what country implementation would look like. The themes highlighted from the interviews were further discussed in the workshop, and the interviewee recommendations regarding next steps were discussed and explored.

The workshop included 11 participants from 8 leading SCM organizations (Supplement 6). Organizations were purposively selected to provide diverse perspectives across the health supply chain sector, with a focus on LMICs. Table 1 describes the characteristics of workshop participants. Three workshop participants also participated in the interviews; the other 8 workshop participants did not participate in the interviews.

### Review of Existing Literature on Supply Chain Management Competencies

Existing public health supply chain competency frameworks and private-sector SCM competency frameworks were reviewed to compare content and structure. Job descriptions and organizational hierarchies were also collected from public- and private-sector health supply organizations in multiple countries, including DSV Global Transport and Logistics, UNICEF, United Pharmaceutical Distributors, and IntraHealth.

**FIGURE 1:**
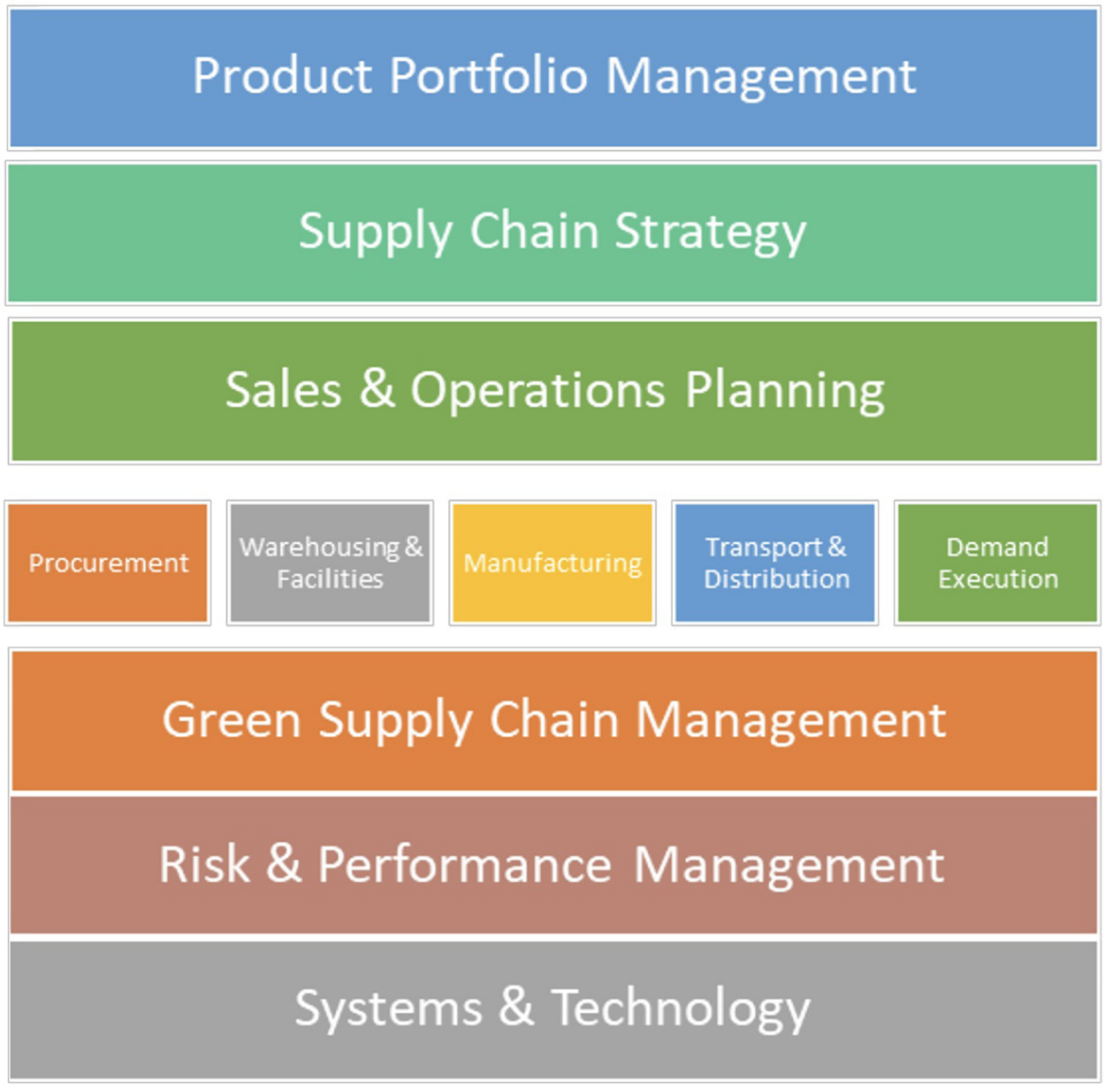
SAPICS Supply Chain Competency Framework^16^ Showing Eleven Supply Chain Competency Groups

The primary frameworks available were compared, including the PtD Competency Compendium for HSCM (PCC)^5^ and the Supply Chain Competency Framework^16^ from SAPICS. These were selected through a targeted review based on the expertise of the authors and experts interviewed. Table 2 captures the domains and career progression specifications of the reviewed frameworks. The PCC^5^ was developed in 2014 to capture all competencies needed within a national health supply chain. SAPICS, which works across Africa, established the Supply Chain Competency Framework as a key building block for developing SCM professional competency. The framework is not specific to health. It contains SCM competencies across 11 competency areas and includes 5 levels of professional designation in its career progression.^16^ Figure 1 outlines the SAPICS Supply Chain Competency Framework used in this work, which was the basis for the SAPICS supply chain competency development and designation assessment.

**TABLE 2.** Competency Frameworks in the Health Supply Chain Space Reviewed

**Framework Name**	**Domains**	**Career Progression**
SAPICS Supply Chain Competency Framework^16^	Product portfolio management Supply chain strategy Sales and operations planning Procurement Warehousing and facilities Manufacturing Transport and distribution Demand execution Green supply chain management Risk and performance management Systems & technology	Supply Chain Management Associate (NQF) 4 Endorsed Supply Chain Management Practitioner NQF 5 Supply Chain Management Advanced Practitioner (NQF 6/7) Supply Chain Management Executive NQF 8 Supply Chain Management Leader Conferred
People that Deliver Competency Compendium^5^	Selection and quantification procurement Storage and distribution Use Resource management Professional and personal	Not specified

Abbreviation: NQF, National Qualifications Framework.

### Development of the Library of Competencies and Designations

The foundational tool of the SCM Professionalisation Framework is the Library of Competencies and Designations. Central to its development was an understanding that a detailed description of HSCM competency requirements across job maturity levels was needed to improve the supply of and demand for SCM workers. Once SCM competencies were defined, PtD could further articulate job descriptions and career paths, as well as matching education offerings.

The PCC was chosen as the base for developing the SCM Professionalisation Framework’s Library of Competencies and Designations, given that it is specific to HSCM. PtD reviewed the PCC against the SAPICS Supply Chain Competency Framework to ensure the content related to SCM was valid and inform the optimal structure for the SCM professionalization framework’s competency component.

The PCC lists domains, competencies, and behavioral competencies. It does not contain professional designations or show how behavioral competencies change across a progression of career levels. To illustrate how competence progresses across career levels and provide suggestions for professional designation hierarchies for future country professional bodies, professional designations were added to the framework. PtD first needed to define how many professional levels would be required to capture the general progression and needs of the health SCM professional landscape. To define professional levels, PtD reviewed the structure of the SAPICS Supply Chain Competency Framework along with job descriptions and hierarchical structures from private and public SCM organizations. PtD evaluated the received private- and public-sector job descriptions with a view to categorizing these roles and associated supply chain activities to the point where there was no overlap, nor could roles be further categorized. PtD also evaluated other professional bodies across sectors to understand the standard viewpoints on the number of designations and found 5 levels to be the most ideal for their purposes. Next, Bloom’s Taxonomy of Educational Objectives^23^ was used as a guide to organize increasing levels of expertise. Informed by our review of existing professional body frameworks, PtD identified 5 professional designations: associate, practitioner, specialist, professional, and leader.

PtD aligned the 5 professional designations with the levels described in Bloom’s Taxonomy as expertise increases: knowledge (associate), comprehension (associate), application (practitioner), analysis (specialist), evaluation (professional), and synthesis (leader). Using Bloom’s Taxonomy as a guide, PtD adapted each behavioral competency in the PCC and defined the behavioral competency required for each of the 5 professional levels. Moving from associate to leader, the behavioral competencies increase in responsibility.

### Data Analysis

Interview data were analyzed using direct thematic analyses. Findings, such as attitudes toward each question, were validated through a validation workshop. To analyze validation workshop data, each participant’s responses were clustered and discussed with workshop attendees, and the resulting findings were documented. To review existing tools, we used thematic analyses to compare the SAPICS Supply Chain Competency Framework and the PCC. We chose this approach due to the structure of the competency data in each tool. The PCC contained multilevel singular statements that must be understood by considering the path from one statement to the next. The SAPICS Supply Chain Competency Framework contained broad statements of competency. We analyzed the core themes of each sentence in both tools.

### Development of the Collection of Roles and Job Descriptions for Health Supply Chains

The Library of Competencies details all the competencies a health supply chain system requires. To help governments and SCM employers translate the behavioral competencies into roles and job descriptions within their organization, PtD developed a reference guide of roles and job descriptions using its previous work on developing job descriptions as a foundation (unpublished data). First, PtD’s prior catalog of job descriptions and a sample of organizational hierarchies were reviewed to identify and create a standard hierarchical structure for organizing the example roles. PtD defined 4 hierarchical levels for this framework: strategic, managerial, operational, and tactical (Box). Next, PtD developed sample job descriptions for a range of roles.

BOX Definitions of Hierarchy of Organizational Levels

**Strategic:** Applies strategic, systems thinking; directs and advises; manages change; and influences internal and external stakeholders.

**Managerial:** Develops, improves, and fulfills organizational and functional objectives and manages efficiency, quality, and risk.

**Operational:** Provides and executes guidance on procedures and processes that are connected.

**Tactical:** Executes the process and assists operational levels to perform their overarching duties.

To add the competency and education content to the job descriptions, PtD referenced the SCM competencies in the Library of Competencies and Designations and the Mapping of Education (described later in this article). For each sample job description, PtD defined the role’s professional designation and assigned the relevant competencies from the Library of Competencies and Designations. Then, PtD listed the appropriate behavioral competencies, given the professional level of the position. Next, PtD added key performance indicators by aligning with various SCM frameworks for the competencies and activities included in the job description. Finally, PtD listed possible education and training opportunities using the developed Mapping of Education for Health Supply Chains. PtD listed suggested training opportunities, available qualifications, and available certifications to meet the competencies required in each job description. The Collection of Roles and Job Descriptions for Health Supply Chains tool includes the following headings: technical competencies, personal/management competencies, basal technology competencies, key performance indicators, training, qualifications available, and certifications available.

### Development of the Mapping of Education for Health Supply Chains

To inform HSCM workers of how to acquire the educational qualifications needed for career progression, PtD developed a list of available certifications and qualifications for each competency and designation level. To identify qualifications, PtD selected a sample national education framework, the South African Qualifications Authority (SAQA), to identify a list of available courses and qualifications while ensuring consistency and completeness. All registered learning programs in South Africa are registered with SAQA, and this authority maintains databases of over 14,000 qualifications for health- and non-health-related subjects. First, PtD removed any qualifications that did not relate to the 7 domains of the Library of Competencies. Next, PtD developed a set of natural language processing algorithms to compare behavioral competencies with SAQA course descriptions and information. If course descriptions significantly overlapped with competencies, these courses were identified as matches.

After identifying all relevant qualifications from SAQA, certificate courses from the following sources were added to provide a nonexhaustive starting point for review: Coursera, Massachusetts Institute of Technology, edukazi.com, Empower, Chartered Institute of Procurement and Supply, Chartered Institute of Logistics & Transport, I+ Solutions, Association of Supply Chain Management/APICS, SAPICS, and Next Level Purchasing Association. These sources were selected by reviewing lists of known collaborators of local professional bodies and PtD. Many of these organizations also represent the largest credentialing bodies within the SCM community.

To organize the Mapping of Education, PtD selected the same structure as the Library of Competencies and Designations and maintained the competency and 5-level professional designations. While the Library of Competencies displays behavioral competencies for each professional level, the Mapping of Education lists the relevant qualifications and certifications.

### Development of the Country Implementation Approach

**FIGURE 2:**
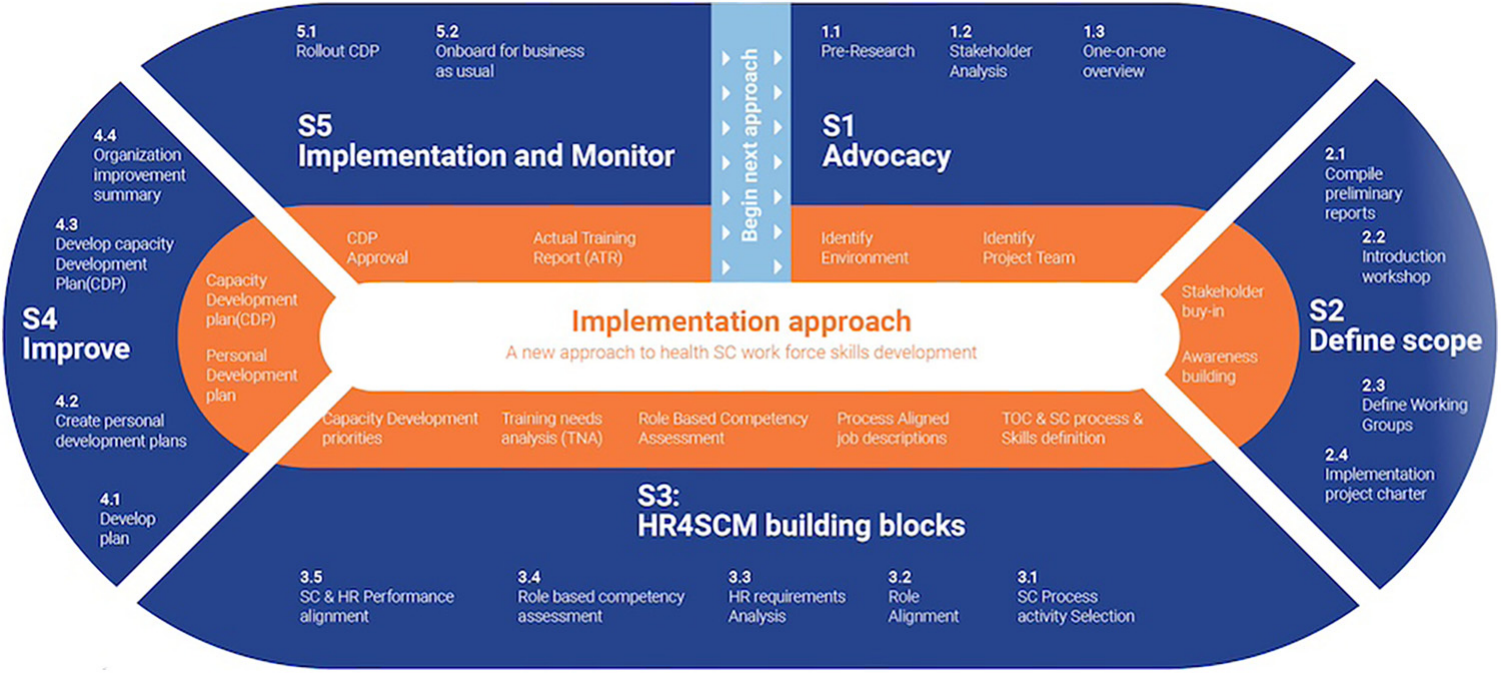
Overview of the Country Implementation Approach for Health Supply Chains

With 3 critical tools developed for professionalizing the HSCM workforce—the Library of Competencies and Designations, the Collection of Roles and Job Descriptions, and the Mapping of Education—PtD created an implementation approach methodology and guide for country teams to follow (Figure 2). PtD defined 5 steps for implementation: (1) advocacy to identify and secure agreement from stakeholders to professionalize the HSCM workforce; (2) define the scope, which defines an implementation plan for the project; (3) Human Resources for SCM building blocks, which aligns a country’s roles and processes to the framework; (4) improve, which defines a professionalization plan, organizational capacity development plan, and personal development plans; and (5) implement and monitor to roll out approved plans. PtD then defined the activities and outputs for each step.

## RESULTS

This section presents the findings from the interviews and data validation workshop and describes the 4 components of the SCM Professionalisation Framework: (1) the Library of Competencies and Professional Designations, (2) the Mapping of Education for Health Supply Chains, (3) the Collection of Roles and Descriptions for Health Supply Chains, and (4) the Implementation Approach for Health Supply Chains. The main result of this initiative is an SCM professionalization framework informed by findings from the data validation workshop and interviews and with concepts included to satisfy the need identified during these steps. The approach employed to merge the PtD PCC and SAPICS Supply Chain Competency Framework models was also discussed and validated through the workshop. The resulting framework is expected to bolster the supply of and demand for skilled personnel within the health care supply chain industry.

The SCM Professionalisation Framework is expected to bolster the supply of and demand for skilled personnel within the health care supply chain industry.

### Findings From Interviews and Validation Workshop

The interview and workshop participants, a sample of SCM professionals responsible for the supply of health products, indicated a strong interest in having an SCM professionalization framework and provided suggestions for how to develop the framework. Participants confirmed that reviewing and comparing the PCC and SAPICS Supply Chain Competency Framework would result in a framework applicable to actors within both the public and private health sectors of LMICs. The participants also believed that a professionalization framework could help improve the supply of and demand for skilled HSCM workers. In terms of design, participants advised that the framework should cover public and private health sectors, include a process to adapt the tool to various country contexts, and include links to job roles.

### Supply Chain Management Professionalisation Framework

#### The Library of Competencies and Designations

The Library of Competencies and Designations maintained the 6 domains of the PCC (selection and quantification; procurement; storage and distribution; use; resource management; and professional & personal) and added a seventh domain: technology (Supplement 2). The number of competencies grew from 37 to 41 across the existing 6 domains and 15 competencies were added under the new technology domain for a total of 56 competencies across 7 domains (Table 3). In addition, the descriptions of 9 behavioral competencies were expanded and captured in more detail. The library defines 672 behavioral competencies, which state the workplace expectations for each competency. For each competency, multiple behavioral competencies are listed. These behavioral competencies are adapted and provided for 5 professional levels, resulting in 3,360 behavioral competencies categorized across 5 levels. (Table 4 describes the 5 professional designations and shows an example of how each behavioral competency has been adapted and defined for each professional designation level).

**TABLE 3.** Health Supply Chain Management Competency Domains and Competencies Included in the Library of Competencies and Designations

**Domain**	**Competency**
Selection & Quantification	1.1 Select the appropriate product
	1.2 Define the specifications of the product, including product quality
	1.3 List any special considerations for the product (e.g., temperature requirements, size, implications for infrastructure)
	1.4 Forecast and quantify product needs
	1.5 Supply planning
Procurement	2.1 Manage procurement costs and budget
	2.2 Build and maintain supplier relationships
	2.3 Manage tendering processes and supplier agreements
	2.4 Undertake contract management and risk
	2.4.1 Place commodity orders
	2.4.2 Manage contracts
	2.4.3 Address risk and ensure quality management
	2.5 Ensure quality of products
	2.6 Manage import and export of products
	2.7 Manage donations of products
	2.8 Prepare for product supply during disasters and emergencies
	2.9 Undertake or manage manufacturing or compounding of products
Storage	3.1 Undertake storage, warehousing, and inventory management
	3.1.1 Manage storage of commodities during emergency
	3.2 Supply commodities to facilities
	3.3 Supply commodities to sections within a facility
	3.4 Manage transport for commodities
	3.4.1 Manage transport for commodities during disaster
	3.5 Manage disposal of products (e.g., expired, damaged, redundant products)
	3.5.1 Define and direct process for managing redundant and returned stock
	3.5.2 Manage process for disposal of returned stock
	3.6 Manage manufacturing or compounding of products
	3.7 Manage re-packing of products
	3.8 Customer relationship management
	3.9 Facility design
Use and Dispense	4.1 Understand use of medical products (e.g., safety procedures, dispensing protocols, standard treatment/testing guidelines)
	4.2 Provide information and advice to the product user/patient
	4.3 Report product use/consumption
	4.4 Dispense or provide commodities to patients/users (i.e., ensuring the product arrives at “the last mile” appropriately)
Resource Management	5.1 Design and implement supply chain system and strategies
	5.2 Oversee operation of a logistics management information system
	5.3 Implement risk management and monitoring and evaluation activities for the supply chain
	5.3.1 Ensure monitoring and evaluation activities are completed
	5.3.2 Implement risk management activities
	5.4 Manage outsourcing of supply chain management functions
	5.5 Manage and plan projects
	5.5.1 Develop and direct project plans
	5.5.2 Execute strategic decision-making
	5.5.3 Manage partnerships
	5.5.4 Direct/participate in teamwork
	5.6 Manage financial activities
	5.7 Oversee/support human resources (e.g., recruitment, training, team management/supervision)
	5.7.1 Manage staff recruitment process
	5.7.2 Train staff
	5.7.3 Supervise staff
	5.7.4 Assessing human resources systems
	5.8 Prepare for product supply during disasters and emergencies
Professional and Personal	6.1 Demonstrate generic skills (e.g., literacy, numeracy, technology)
	6.1.1 Exhibit high understanding of literacy and numeracy
	6.2 Demonstrate strong communication skills
	6.2.1 Practice cultural awareness
	6.3 Utilize problem-solving skills
	6.3.1 Negotiate
	6.3.2 Practice effective time management
	6.3.3 Take risk into account and implement security measures
	6.4 Exhibit professional and ethical values
	6.4.1 Demonstrate integrity
	6.4.2 Engage in continuous professional development
	6.5 Prove leadership abilities
	6.5.1 Demonstrate resilience and ability to manage stress
	6.6 Abide by rules/laws/legislation
Technology	7.1 Data science
	7.2 Blockchain
	7.3 Unmanned aerial vehicles
	7.4 Temperature and monitoring
	7.5 Planning systems
	7.6 Enterprise resource planning system includes function of logistics management information system
	7.7 Automation
	7.8 Artificial intelligence
	7.9 Additive manufacturing
	7.10 Internet of things
	7.11 Cloud computing
	7.12 Basic office skills
	7.13 Have a command of technology
	7.14 eProcurement
	7.15 Global positioning system

**TABLE 4.** Definitions of Health Supply Chain Management Professional Designations and Example of Behavioral Competency Across Designations

**Professional Designation**	**Description**	**Competency 3.1 Undertake Storage, Warehousing, and Inventory Management**
		Behavioral competency: Ensure accurate verification of rolling stocks.
Associate	Equivalent to entry level in the competency framework. This is an execution level designation.	Awareness of the importance of accurate verification of rolling stocks.
Practitioner	Practitioner level is the first management level in the competency framework. This is an execution level designation with some supervisory and management competencies.	Understand the importance of accurate verification of rolling stocks.
Specialist	Specialist level is the mid-management level designation. Typically associated with management level accountability depending on domain.	Ensure accurate verification of rolling stocks.
Professional	Professional level is the first strategic level designation and is typically characterized by analysis and input into strategic decision-making.	Measure the accuracy of verification of rolling stocks.
Leader	Leader level is the primary strategic level designation and is characterized by long-term decision-making competencies.	Develop processes that accurately verify rolling stocks.

Source: Supplement 2.

The library covers the services and practices of the health supply chain across public and private sectors and defines which competencies are required and at which professional level. The domains are process-dependent, and roles are not prescribed. If the activity is undertaken, the country must decide which roles should undertake that activity; the worker undertaking the activity must possess the appropriate competency.

To support countries in operationalizing the Library of Competencies and Professional Designations and establish a professionalized HSCM workforce, 3 additional complementary components were developed.

#### The Collection of Roles and Job Descriptions for Health Supply Chains

The Collection of Roles and Job Descriptions for HSCs includes 93 sample job descriptions that reflect the full scope of SCM functions and roles and sample organograms for a range of supply chain roles, spanning all competencies (Supplement 3). This tool provides an example of how countries can use and build job descriptions based on the framework’s Library of Competencies and Mapping of Education.

#### The Mapping of Education of Health Supply Chains

The Mapping of Education of Health Supply Chains is a directory of education qualifications and certifications directly related to the competencies across the 5 professional designations of the competency framework (Supplement 4). The resulting mapping contains over 250 courses from various providers, who provide guidance on the type of offerings found at a global level to serve as the basis for local alignment and inclusion. The qualifications align with the 5-level professional designations for each behavioral competency. The courses, as mapped, then need to be considered in a country context against a country’s education framework. It is imperative to understand the education framework within a country context and what is acceptable within a career framework in that context. Table 5 shows the structure of the Mapping of Education and provides a sample of its contents.

**TABLE 5.** Education Mapping for One Health Supply Chain Management Competency

**Competency**	**Relevant Qualifications With no Currently Assigned Designation**	**Qualification to Fulfill Associate Designation Requirement**	**Qualification to Fulfill Practitioner Designation Requirement**	**Qualification to Fulfill Specialist Designation Requirement**	**Qualification to Fulfill Professional Designation Requirement**
2.2 Build and maintain supplier relationships	CIPS Level 2 Certificate in Procurement and Supply Operations Interpret and apply International commercial terms skills program	CIPS Level 3 Advanced Certificate in Procurement and Supply Operations	CIPS Level 4 Diploma in Procurement and Supply Logistics and Supply Chain Management Massachusetts Institute of Technology APICS Principles of operations management series certificate SPSM (Essential Procurement Skills) SPSM2 (Global Procurement Management) Diploma in International Trade Management in Exports/Imports Higher Certificate in Export Management	CIPS Level 5 Advanced Diploma in Procurement and Supply APICS Certified in production and inventory management certificate SPSM3 (Enterprise-Wide Procurement Influence)	CIPS Level 6 Professional Diploma in Procurement and Supply

Abbreviations: CIPS, Charted Institute for Procurement and Supply; SPSM, Senior Professional in Supply Management

#### The Implementation Approach for Health Supply Chains

To aid local country teams and implementers, the Implementation Approach for Health Supply Chains was developed to serve as a unified methodology that systematically guides countries to achieve their stated professionalization objectives in a detailed phased approach (Supplement 5) across 5 steps. Figure 2 provides an overview of the implementation approach with its 5 steps, 18 deliverables, and key objectives. This phased approach ensures the necessary foundational advocacy and the pre-work required is undertaken so that the 3 tools can be used effectively (Library of Competencies, Mapping of Education, and Collection of Roles and Job Descriptions). The approach also ensures implementers developing an SCM professionalization framework can successfully engage structural organizations, such as professional bodies, professional councils, bodies that approve education pathways, public service commission, as well as the functional supply and demand actors, to accomplish transformational change.

**FIGURE 3:**
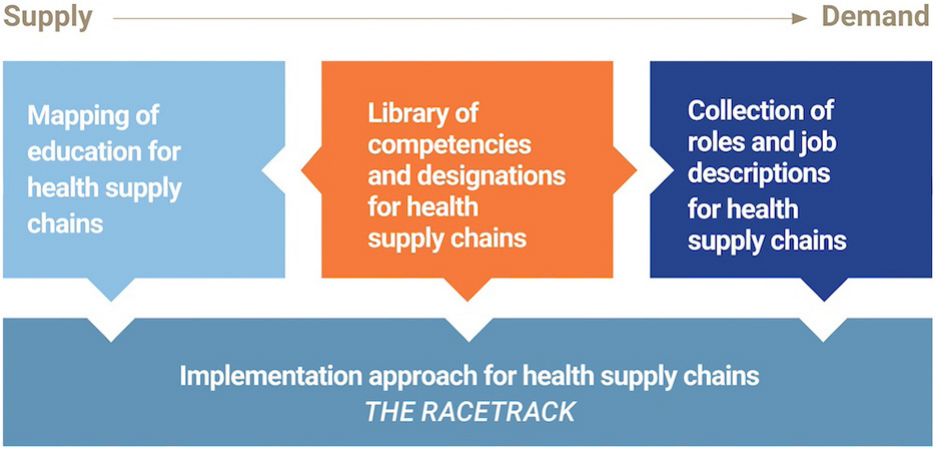
Elements of the SCM Professionalisation Framework Source: Supplement 1.

Figure 3 illustrates how the 4 components of the SCM Professionalisation Framework fit together. The Library of Competencies is the central document that provides standards for both the Mapping of Education, which promotes the supply of skilled HSCM workers, and the Collection of Roles, which promotes demand for workers. Underlying these 3 components, the Implementation Approach for Health Supply Chains guides users on applying these tools within a specific country context.

## DISCUSSION

In this article, we have proposed the contents and components for an SCM professionalization framework that can be applied in any country context, particularly focusing on LMICs, to improve the availability and use of SCM professionals for health supply chain operations. PtD’s comprehensive SCM Professionalisation Framework aligns career path, education, and professional growth in HSCM. We have extracted and developed a library of competencies and designations. Using these defined competencies as a basis, we developed a collection of roles and job descriptions for health supply chains, a mapping of education to build defined competencies, and guidance on an implementation approach to help organizations adopt this framework.

The SCM workforce managing essential health products requires substantial technical and managerial capacities.^3^^–^^5^ However, there is an inadequate supply of HSCM workers with the required competencies in many countries, and few studies have explored the gaps in supply of and demand for this workforce.^14^ Labor market analyses have explored the supply and demand of clinical health workers, such as doctors, nurses, midwives, and pharmacists^24^^,^^25^; however, these analyses do not mention or consider the supply chain workforce managing health products nor the supply chain activities that clinical staff carry out. Staff managing health supply chains, particularly in LMICs, are nearly always clinical staff, who receive only short-term training in SCM, and as staff rotate, staffing gaps emerge.^4^^,^^6^^,^^16^^,^^17^ A systematic approach is needed to advocate establishing health SCM professionals as an occupational group or ensuring that health care professionals serving in these positions have defined competencies in SCM.^26^

Professionalization pursuits for many bodies are in various stages of progress. While midwives and community health workers have sought or undergone professionalization processes for increased recognition,^27^^,^^28^ others, like those in the humanitarian sector, are in the process of professionalizing some of the sector’s many related professions.^29^^–^^31^ Though competency frameworks have been developed for certain humanitarian professions,^32^^–^^34^ a study of humanitarian professions found that “very few humanitarian profession areas had developed agreed competency frameworks or certification mechanisms for individuals. None has a formally recognized professional association, although coordination bodies, membership networks, and active communities of practice are numerous.”^31^ For health SCM professionals, there currently isn’t an official body at the global or local level that is recognized as the standard-setting body or that takes responsibility for professionals in terms of ethical practice of SCM and career development.

Achieving universal health coverage depends on effective HSCM and a competent workforce to provide the reliable supply of affordable, quality medicines required for increased service coverage.^1^^,^^2^ By increasing awareness of career paths in HSCM and developing a pipeline of qualified workers, efforts to professionalize the HSCM workforce can increase the supply of skilled workers. This SCM professionalization framework is a critical and significant step to defining global professional standards of the HSCM profession, articulating HSCM competencies and career pathways, and defining clear learning and teaching courses to produce a skilled HSCM workforce. Establishing health supply chain professionals as an occupational group can benefit employers by continuously producing a pool of appropriately skilled workers. It can benefit workers by creating a career path and sense of prestige, which creates an enabling environment for motivation and the retention of a skilled workforce whose efforts contribute immensely to improved SCM outcomes, such as increased availability of health products. Applied together, the elements of this framework can encourage growth in the number and skills of HSCM workers and their use. We expect that by creating a systematic implementation approach with standardized tools that enable the process of professionalization to begin, country teams and implementers will yield results more quickly.

Achieving universal health coverage depends on effective HSCM and a competent workforce to provide the reliable supply of affordable, quality medicines required for increased service coverage.

PtD has explored the main ways the SCM Professionalisation Framework can be used to improve the supply and demand of supply chain professionals. Governments can use the framework to define HSCM professional standards and competencies. Employers can better define competency requirements for needed positions, creating a clearer demand for the HSCM workforce. The framework can be used to identify training needs and shape curricula at institutions that educate HSCM professionals. HSCM professionals can map career paths and benefit from professional recognition.

PtD has developed guidance on an implementation approach (Supplement 5). However, more work is needed to explore the challenges faced in successfully adopting the SCM Professionalisation Framework and applying the Implementation Approach. The ministries of health in Rwanda, Nigeria, and Mozambique are working toward implementing this framework. Each country has its own needs and is at a different stage of implementation. Their experience shows promise that the framework presented in this article is useful and can be applied in various contexts and that subsequent country applications will benefit from this standardized guidance.

### Country Applications of the Supply Chain Management Professionalisation Framework

In Rwanda, a local HSCM professionalization framework is being developed to strengthen the HSCM workforce, guided by the PtD SCM Professionalisation Framework. The project team conducted advocacy meetings, defined the framework’s scope, and is currently aligning supply chain activities with the competency framework. They are applying process mapping tools in the public sector, focusing on the central medical stores and health facilities. Plans include expanding the framework’s application to regulatory institutions and the education sector to standardize training curricula and align it with market needs.

Mozambique is implementing an HSCM professionalization framework nationwide, starting with a redesign of the central medical stores. The Ministry of Health adopted the SCM Professionalisation Framework process and is completing Step 3 of the Implementation Approach, where a supply chain mapping tool identifies job roles and competencies. A gap analysis was performed to compare job descriptions with available education. The ongoing development of competency-based assessment tools aims to inform a capacity development plan that will bridge gaps in education, processes, and performance.

In Nigeria, the Ministry of Health is working on creating an SCM professionalization framework to develop a career path structure for state-level health supply chain managers and other practitioners. The professionalization process aims to standardize the HSCM workforce and support supply chain integration efforts. The project is nearing the end of Step 2 of the Implementation Approach, with a team conducting a stakeholder analysis and collecting data through interviews and surveys to inform a project charter and a customized implementation plan for Nigeria based on the PtD Implementation Approach.

### Limitations

While we believe the interviews and validation workshop participants that informed the development of the SCM Professionalisation Framework provided a sufficient representation of the needs of the HSCM community, a wider engagement of participants with the framework—happening in countries like those previously cited—will show opportunities to strengthen this framework. In addition, there is a risk that implementers could use a tool from this framework in isolation without following the Implementation Approach, which would limit the success of the results.

## CONCLUSION

Effective HSCM is a critical enabler for achieving universal health coverage, which requires a reliable supply of quality, affordable medicines for increased service coverage. The SCM Professionalisation Framework is a critical step to institutionalize HSCM professionals and strengthen human resources for HSCM. Applying this framework in a country context is expected to improve the availability of SCM professionals managing health products. The framework can support governments in defining SCM professional standards and competencies, help employers describe SCM competency requirements, enable the HSCM workforce to map career pathways, and allow education institutions producing HSCM professionals to shape curricula. The SCM Professionalisation Framework is being adopted by ministries of health in multiple countries and is perceived to be valuable; however, formal evaluation is still required. Further research will be important to build the evidence base on country use of this framework and its effect on the HSCM profession.

## Supplementary Material

GHSP-D-23-00119-Meier-Supplement2.xlsx

GHSP-D-23-00119-Meier-Supplement4.xlsx

GHSP-D-23-00119-Meier-Supplement5.pdf

GHSP-D-23-00119-Meier-Supplement3.pdf

GHSP-D-23-00119-Meier-Supplement1.pdf

GHSP-D-23-00119-Meier-Supplement6.pdf

## Notes

**Peer Reviewed**

**First Published Online:** August 20, 2024.
