# Supplementary material for: Presenting a Framework to Professionalize Health Supply Chain Management
Source: Glob Health Sci Pract. 2025 May 9;13(Suppl 1):e2300119. doi: 10.9745/GHSP-D-23-00119 (PMC12063754; doi:10.9745/GHSP-D-23-00119)
Supplement: GHSP-D-23-00119-Meier-Supplement5.pdf [file GHSP-D-23-00119-Meier-Supplement5.pdf]

SCM Professionalisation Framework

IMPLEMENTATION APPROACH FOR HEALTH SUPPLY CHAINS

NOVEMBER 2020

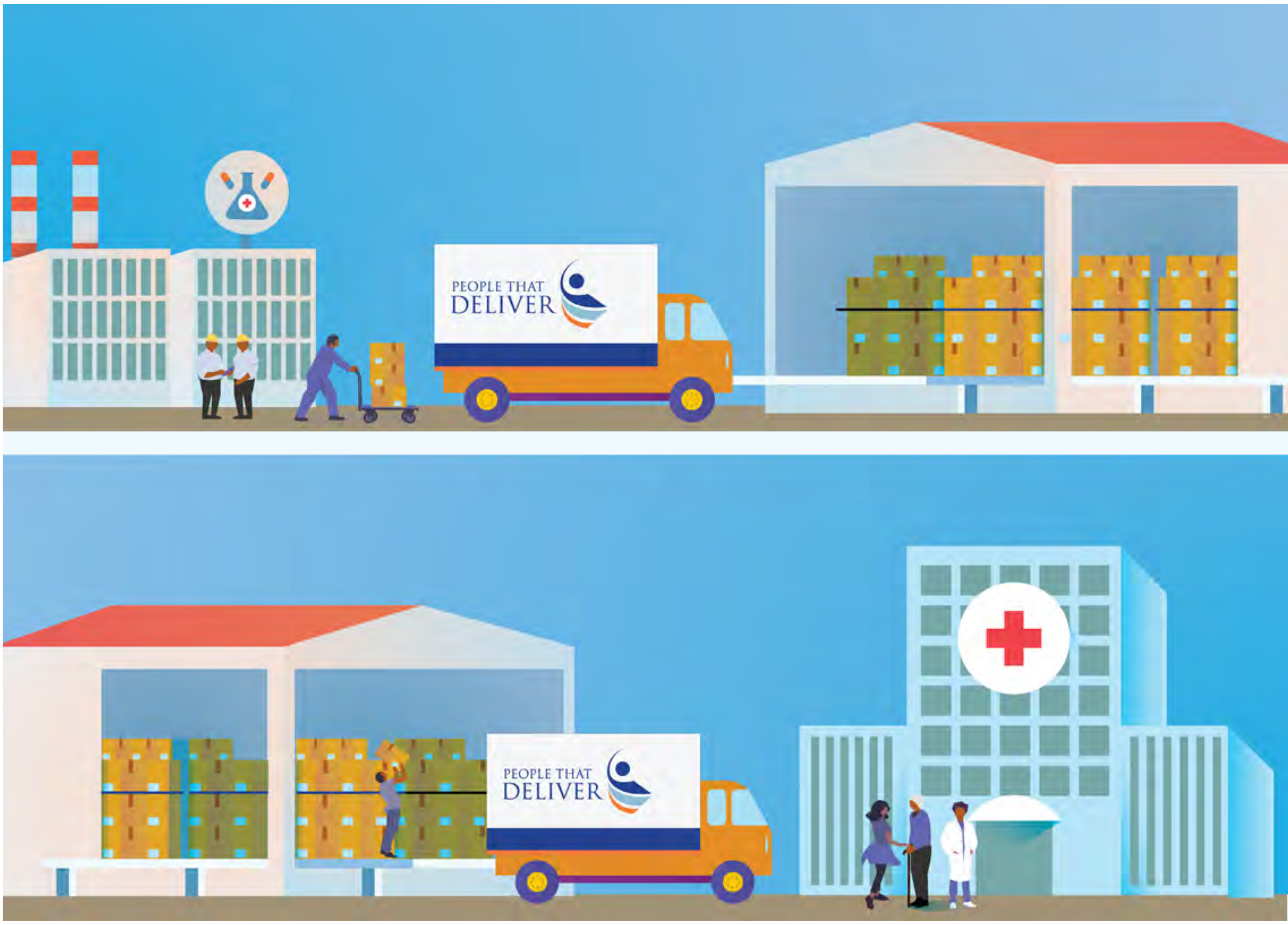

PEOPLE THAT DELIVER

SCM Professionalisation Framework

IMPLEMENTATION APPROACH
FOR HEALTH SUPPLY CHAINS

NOVEMBER 2020

PEOPLE THAT DELIVER

Authors

Andrew dos Santos

MPhil, BSc (Hons)., SCOR-P, CPIMDirector, CLX

Pretoria, South Africa.
andrew@clx.co.za

Richard dos Santos

BBA, CPIM, CSCP, Director, SAPICS

Copenhagen, Denmark,
richard@sapics.org.za

Bridget McHenry

MPH, CSCP, Senior Organisational Development Advisor

Commodities Security & Logistics Division, Bureau for Global Health | Office of Population & Reproductive Health USAID

Arlington, Virginia, USA.
bmchenry@usaid.gov

Michael Egharevba

BPharm MBA, MPH, PGDSCM, PGDDA, SPSM, SPSM2

Workforce Development Specialist

USAID Global Health Supply Chain-Procurement Supply Management (GHSC-PSM) Project

Arlington, Virginia, USA.
megharevba@ghsc-psm.org

Dr Andrew Brown

PhD, BPharm, GCHE

Senior Director Health Workforce Development (PtD Coalition Member)

IntraHealth, Chapple Hill, North Carolina, USA.
abrown@intrahealth.org

Dominique Zwinkels

Executive Manager, People that Deliver (PtD)

Copenhagen, Denmark
dzwinkels@unicef.org

Barry Chovitz

USAID Global Health Supply Chain-Procurement Supply Management (GHSC-PSM) Project

bchovitz@ghsc-psm.org

James Johnson

USAID Global Health Supply Chain-Procurement Supply Management (GHSC-PSM) Project

jajohnson@ghsc-psm.org

Acknowledgements

This work was funded by the USAID Global Health bureau's Family Planning and Reproductive Health (FP/RH) programme. This work was a joint activity of the USAID Global Health Supply Chain Programme-Procurement and Supply Management (GHSC-PSM) project, People that Deliver (PtD), SAPICS, and USAID.

The authors would like to thank the members of these organisations for their contribution and cooperation as together we seek to increase the availability of medicines to beneficiaries. Many colleagues offered their time for interviews and to engage in focus group discussions; we thank you for your contribution.

The SCM Professionalisation Framework is dedicated to Andrew dos Santos. His hard work and knowledge were central to the development of this resource.

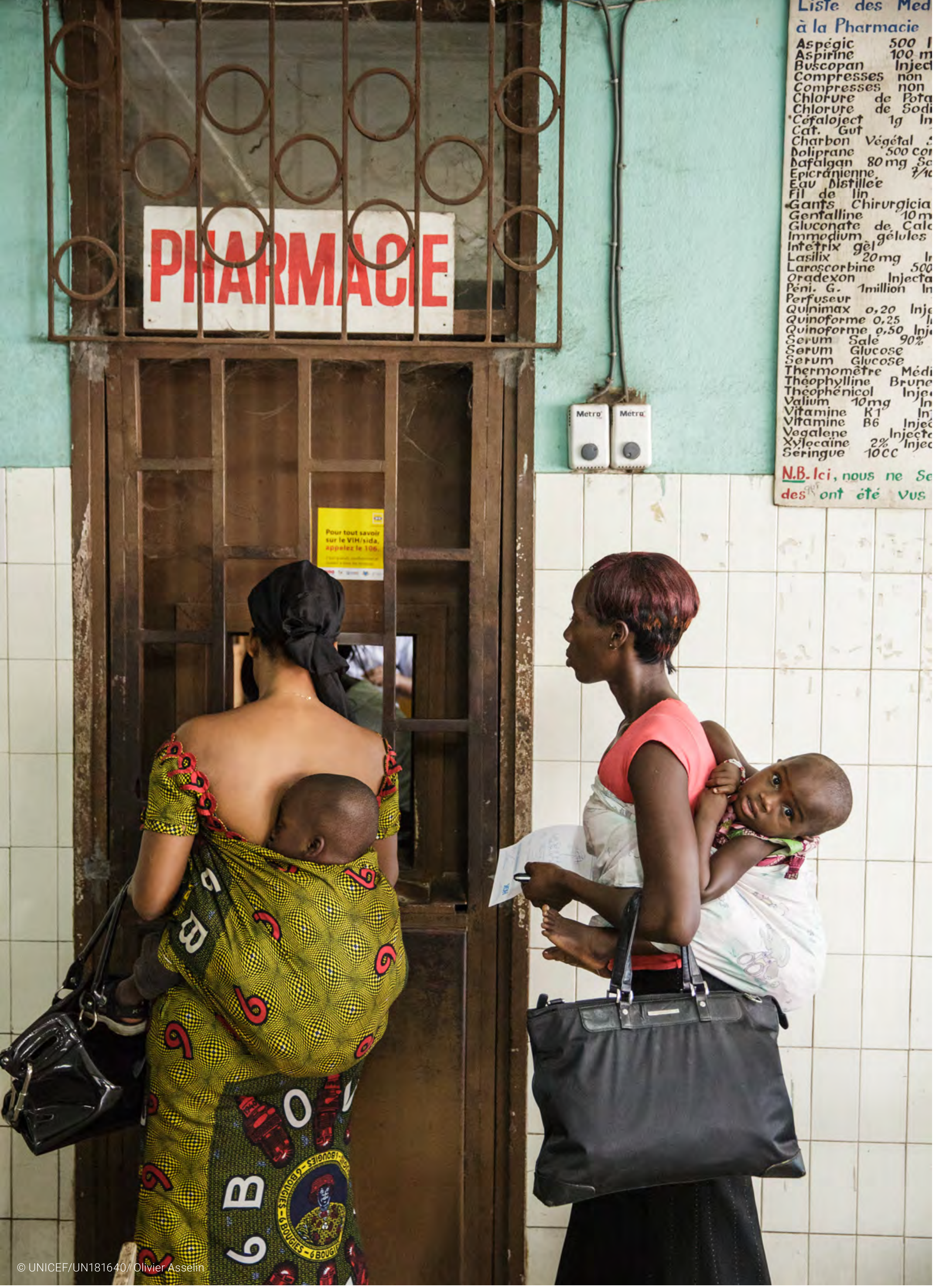

© UNICEF/UN181640/Olivier Asselin

Contents

Authors 4

Acknowledgements 5

Overview 8

Library of Competencies & Designations for Health Supply Chains 11

- Competency terminology and definitions 11
- Methodology 14

Collection of Roles and Job Descriptions for Health Supply Chains (Demand) 17

- Methodology 18

Mapping of Education for Health Supply Chains 22

- Methodology 23

Implementation Approach Objectives 26

Appendix A 36

List of Abbreviations

ABBREVIATION	MEANING
GHSC-PSM	USAID Global Health Supply Chain Programme-Procurement and Supply Management
HSCM	Health supply chain management
JD	Job descriptions
NLP	Natural Language Processing
PtD	People that Deliver
SAPICS	Industry Association for Supply Chain Management in South Africa
SAQA	South African Qualifications Authority
SC	Supply chain
SCM	Supply chain management

Overview

To better understand issues in the availability and use of supply chain management (SCM) human resources in a country context, the USAID Global Health Supply Chain Programme-Procurement and Supply Management (GHSC-PSM) project, in conjunction with USAID and People that Deliver (PtD), considers that a “whole of SCM labour market” approach provides a deeper and more holistic understanding of the SCM employment environment.

The whole of the SCM labour market refers to the supply and demand for SCM labour in which employees are the supply and employers the demand in a specific country context. The country context includes urban, regional and more remote environments and encompasses all the sectors where SCM technical personnel are employed in that country.

Key stakeholders include government (ministries of labour, education, planning and health), professional associations, academic institutions, private sector (resources industries, fast-moving goods, health, third-party logistics providers (3PL) and fourth-party logistics providers (4PL)), and the humanitarian and development sectors.

In 2019 GHSC-PSM, in collaboration with PtD, SAPICS and USAID, published a SCM Professionalisation Framework white paper outlining how a SCM professionalisation framework could be used by:

- Governments to define the professional standards of the profession
- Employers to articulate SCM competency requirements and career pathways in their organisations
- Learning institutions to define clear learning and teaching courses
- SCM employees to map out a professional career in SCM

In 2020, the same consortium worked together to complete the necessary elements of the ‘SCM professionalisation framework’. This framework has a ***‘Library of Competencies & Designations for Health Supply Chains’*** at its core meeting public and private sector needs. The competency framework then acts as the ‘standard’ to ensure an aligned ***‘Mapping of Education for Health Supply Chains’*** (supply) and ***‘Collection of Roles and Job Descriptions for Health Supply Chains’*** (demand), for a particular country context. Further, a ***‘Implementation Approach for Health Supply Chains’*** provides clear guidance on how to begin this journey of change. Figure 1 shows the interrelationship of these elements.

Although each of the SCM Professionalisation Framework components can be used by themselves it is believed that most benefit is obtained from using the ***‘Implementation Approach for Health Supply Chains’*** to create lasting systems change.

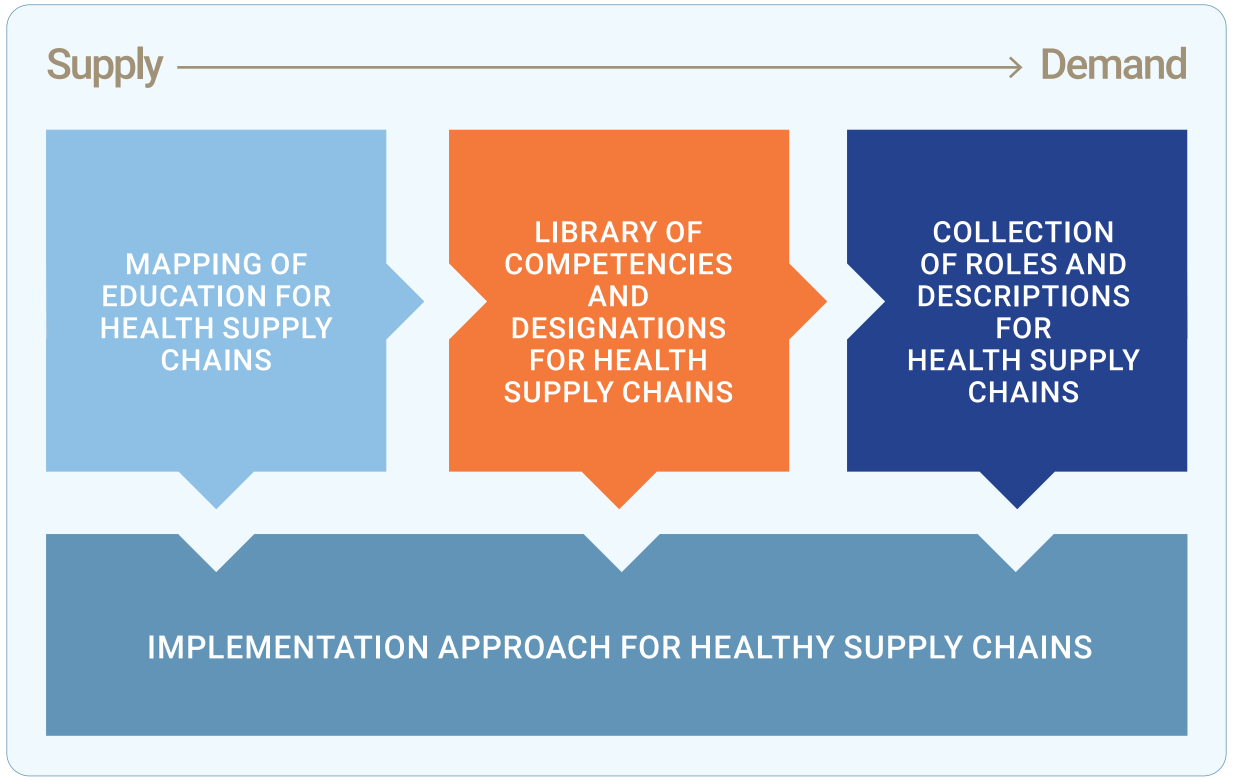

Figure 1:
Elements of the PtD Professionalisation Competency Framework

Library of Competencies & Designations for Health Supply Chains

Building on the PtD Health Supply Chain Management (HSCM) Competency Compendium (2014), this serviced-based (non-cadre specific) framework presents seven competency domains that cover the practice of HSCM across the public and private sector (Figure 1). This comprehensive framework provides ‘behaviour statements’ outlining workplace expectations in all listed competency domains and competency groups. This revised version provides a five-level maturity or designation classification for each competency area, acting as a ‘standard’ for comparing education requirements and related job descriptions.

Collection of Roles and Job Descriptions for Health Supply Chains (Demand)

Building on previous work conducted by PtD (2018), this tool provides a systematic way to build job descriptions and consider SCM roles with reference to the Library of Competencies & Designations for Health Supply Chains. An example set of SCM job descriptions and organisational charts is provided.

Mapping of Education for Health Supply Chains (Supply)

This tool provides an explanation of how an education and training framework should be considered within a country context, to align with SCM job roles. The tool also uses a machine learning approach to review existing SCM education and training opportunities to determine alignment with domains and levels within the Library of Competencies & Designations for Health Supply Chains.

Implementation Approach for Health Supply Chains

This tool provides an overview of the step by step activities that can be undertaken over a three-week period to engage local stakeholders advocating the need to implement a SCM Professionalisation approach. This methodology also validates a plan of action to apply the Library of Competencies & Designations for Health Supply Chains, Collection of Roles and Job Descriptions for Health Supply Chains and Mapping of Education for Health Supply Chains to systematically improve SCM professionalisation in the country context.

Library of Competencies & Designations for Health Supply Chains

Competency terminology and definitions

Internationally, the use of competency-related terminology can vary, so the following definitions are offered here to encourage common understanding:

Competency compendium: A comprehensive catalogue of competency areas with associated behavioural competencies referencing common supply chain processes and job level activities.

Domains: The high-level groups, or clusters of competency areas, within the compendium. Traditionally, domains do not exceed six. The six domains used in this PtD competency compendium are selection and quantification; procurement; storage and distribution; use; resource management; and professional and personal (Figure 3).

Competency area: The overarching capacity/skills of a person to perform in a specific area. For example, within the domain of procurement, PtD has suggested the following competency areas:

2.1 Manage procurement costs and budget
2.2 Build and maintain supplier relationships
2.3 Manage tendering processes and supplier agreements
2.4 Execute management of contract, including risk and quality management
2.5 Assure quality of products
2.6 Manage import and export of products
2.7 Manage donations of products

Behavioural competencies:

Expressions of what work activities are performed and observed when professionals apply motives, traits and skills to a relevant task.

For example, for the domain of procurement and the competency area of manage tendering processes and supplier agreements, such behavioural competencies may include “develop bidding documents”, “use WHO prequalification system to confirm quality suppliers” , “manage a tender process using country systems” , or “formalise contracts with successful companies.” This compendium contains behavioural competencies and references to required knowledge, depending on the source document used.

Competency Framework:

A collection of competency areas with associated behavioural competencies that define the expected requirements of a particular cadre/profession (Figure 2).

For example, a competency framework may be developed for a warehouse manager, while a separate competency framework would be required for a pharmacist. The composition of country-based, cadre-specific competency frameworks will also depend on the structure of the supply chain and at which levels various competencies are allocated.

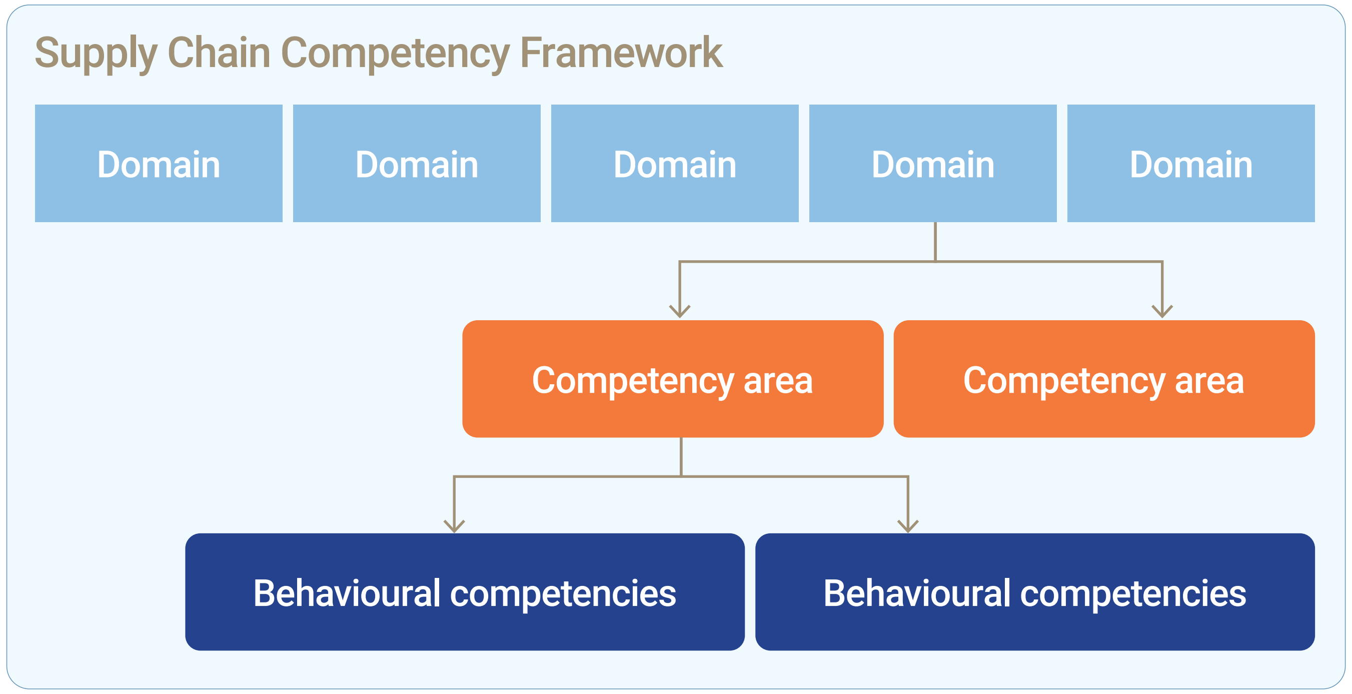

Figure 2:
The structure of competency frameworks

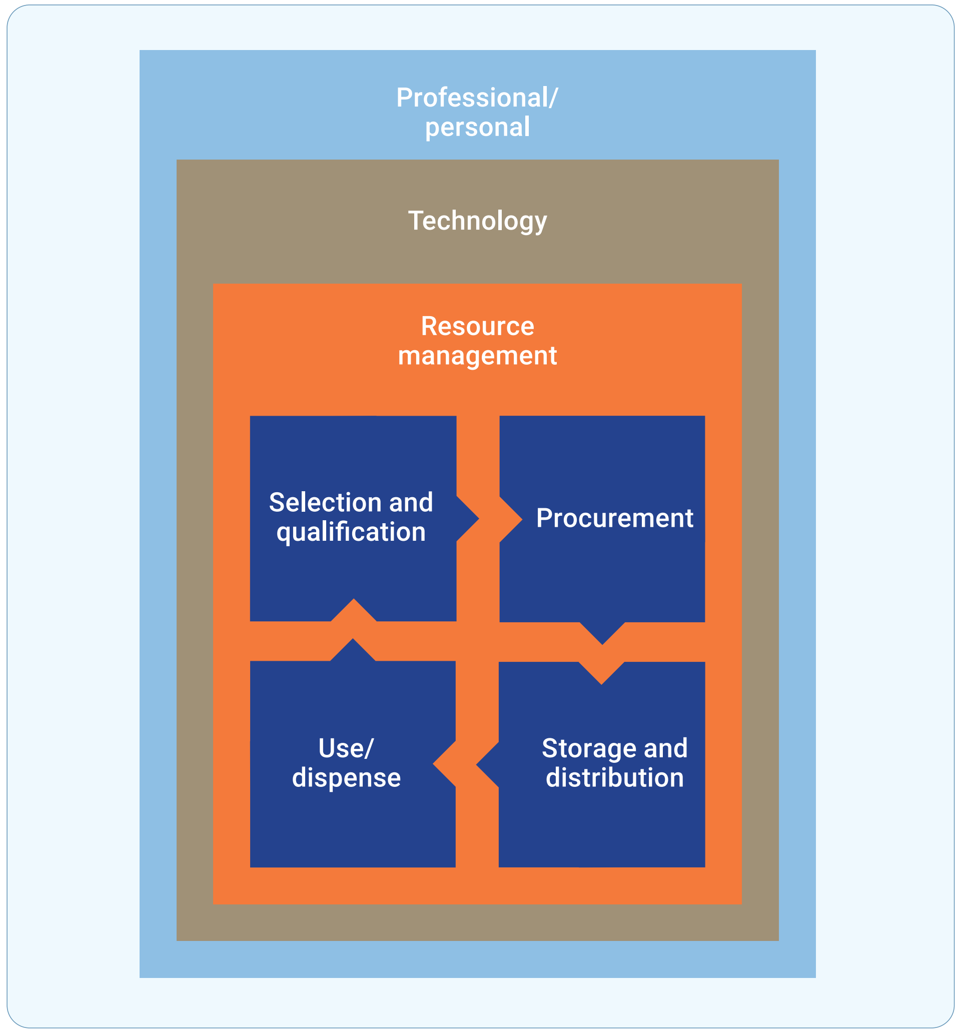

Figure 3:
The relationship of the six domains within the PtD Competency Compendium for Health Supply Chain Management

Designation levels:

Accommodating the differing levels of work, focus and scope as well as denoting training and education required at each level. Each designation level has been aligned to ensure articulation between levels or elimination of competency overlap. The competency framework is divided into designation levels namely:

Associate

Associate level is the entry level of the competency framework. This is an execution level designation.

Practitioner

Practitioner level is the first management level in the competency framework. This is an execution level designation with some supervisory and management competencies.

Specialist

Specialist level is the mid-management level designation, typically associated with management level accountability depending on domain.

Professional
Professional level is the first strategic level designation and is typically characterised by analysis and input into strategic decision making.

Leader
Leader level is the primary strategic level designation and is characterised by long-term decision-making competencies.

Each designation level has a set of associated terms which denote the level and nature of a particular competence the individual must possess in order in order to fulfil the workplace duties associated with a specific activity.

E.g. In the Storage Domain the behavioural competency:

Ensure accurate verification of rolling stocks, the Associate designation level is required to have an "Awareness of the importance of accurate verification of rolling stocks" while the Practitioner needs to "Understand the importance of accurate verification of rolling stocks".

In this case the difference is indicated by the terms Awareness and Understand. There are several terms used across the competency which have been referenced from multiple educational and vocational resource.

For a detailed description of the competency framework, refer to the Overview of the SCM Professionalisation Framework and the Library of Competencies and Designations for Health Supply Chains.

Methodology

The very nature of a professional body is that it encapsulates a path to professionalisation. For this reason, the PtD Competency Compendium for Health Supply Chain Management is an ideal base for the Library of Competencies & Designations for Health Supply Chains. In order to encapsulate a full pathway however, the competencies needed to be expanded in responsibility and complexity. Additionally, PtD had already extensively reviewed the document and researched additions to the framework. These additions were added before the expansion of the competencies and then developed in the same manner as the rest of the framework.

In addition to the expansion according to the previous review, the authors conducted an extensive review, comparing the framework to current best practice supply chain process frameworks in order to verify its validity in the broader sense of supply chain management. Where necessary wording was expanded. However this analysis was used more in developing the framework into the higher and lower levels as required. In order to build out these levels the authors had to first define how many levels would be needed. For this there were a few inputs: the first was the structure of other supply chain professional body frameworks and the second was job descriptions and hierarchies from private and public organisations either generously donated or from previous projects, which both allowed the framework to be compatible with the majority of hierarchies as well as other professional bodies in the sample set (Appendix A). From these inputs it was determined that five levels would be required to capture the complexity exhibited by these documents bearing in mind that the levels are in themselves only indicative and can be edited by countries during their own implementation processed.

To expand the PtD Competency Compendium for Health Supply Chain Management into the above-mentioned levels a trusted framework was required. Bloom's taxonomy of cognitive and affective domains was used to increase complexity of the competency. The previous analysis using supply chain frameworks was used to increase responsibility using the appropriate terms associated with the domain and aligned to the designation. The final domains chosen are named accordingly and are aligned to Bloom's taxonomy.

- Associate
- Practitioner
- Specialist
- Professional
- Leader

Table 1: Alignment of Blooms Taxonomy to Professional Designations

Knowledge	Comprehension	Application	Analysis	Evaluation	Synthesis
Associate	Associate	Practitioner	Specialist	Professional	Leader

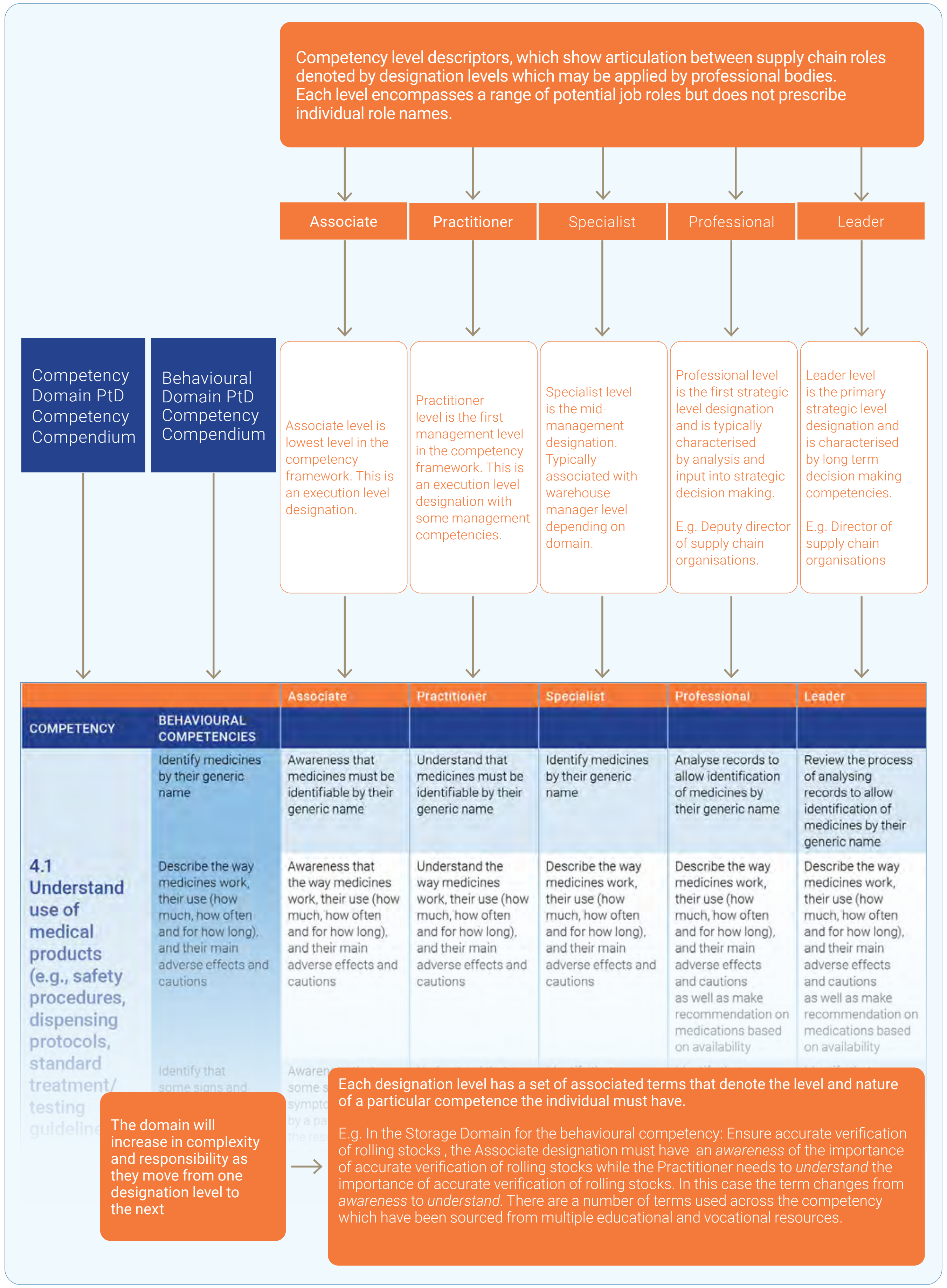

Figure 4:
Description of how Library of Competencies & Designations for Health Supply Chains is constructed

Collection of Roles and Job Descriptions for Health Supply Chains (Demand)

The SCM Professionalisation Framework aims to professionalise supply chain management personnel, which should have the effect of streamlining multiple aspects of supply chain management in a country or organisation. However, the country or organisation must have a need for the personnel, in other words a demand must be created. The Collection of Roles and Job Descriptions for Health Supply Chains was created for this purpose and it outlines ways in which the Library of Competencies & Designations for Health Supply Chains can be organised operationally to create this demand.

There are several ways that this job description (JD) compendium can assist you, whether you are involved in operations management or workforce development.

This collection was designed to be a reference guide to assist human resources and supply chain practitioners in the field in designing their organisational hierarchies and planning for human resource initiatives across the healthcare supply chain.

Although the job description templates are provided as a point of reference for workforce development activities, they are best used with the Implementation Approach for Health Supply Chains. The Implementation Approach contains details pertaining to whole of labour market considerations, where these templates can be used as a component in creating a tailored workforce development plan.

Methodology

The Collection of Roles and Job Descriptions for Health Supply Chains (Demand) was created by PtD (2018) and received input from multiple country examples to create the base job descriptions. These base job descriptions were then compared in detail to a set of hierarchies that encompass the majority of roles as below to create the hierarchy found in this document. Based on aggregated descriptions of the job descriptions studied, the base job descriptions were assigned a primary domain and primary competencies. It must be stressed however that the example in this document is only one of many ways to combine the job descriptions.

Once the base job descriptions were created the metrics and training were defined. In order to define these, the domains in which the job descriptions reside and the primary competencies were used to align the job descriptions to various supply chain management frameworks. These frameworks define standard practices and metrics for the activities to which the job description pertains. These alignments were then used to assign standard metrics to the job descriptions.

Finally, using the competencies assigned to each job description, the Mapping of Education for Health Supply Chains was used to provide education possibilities to teach the competencies required for an individual to take up each job description.

To fully use the collection, one must look at the attributes of a job description (JD) which are structured using the following table headers:

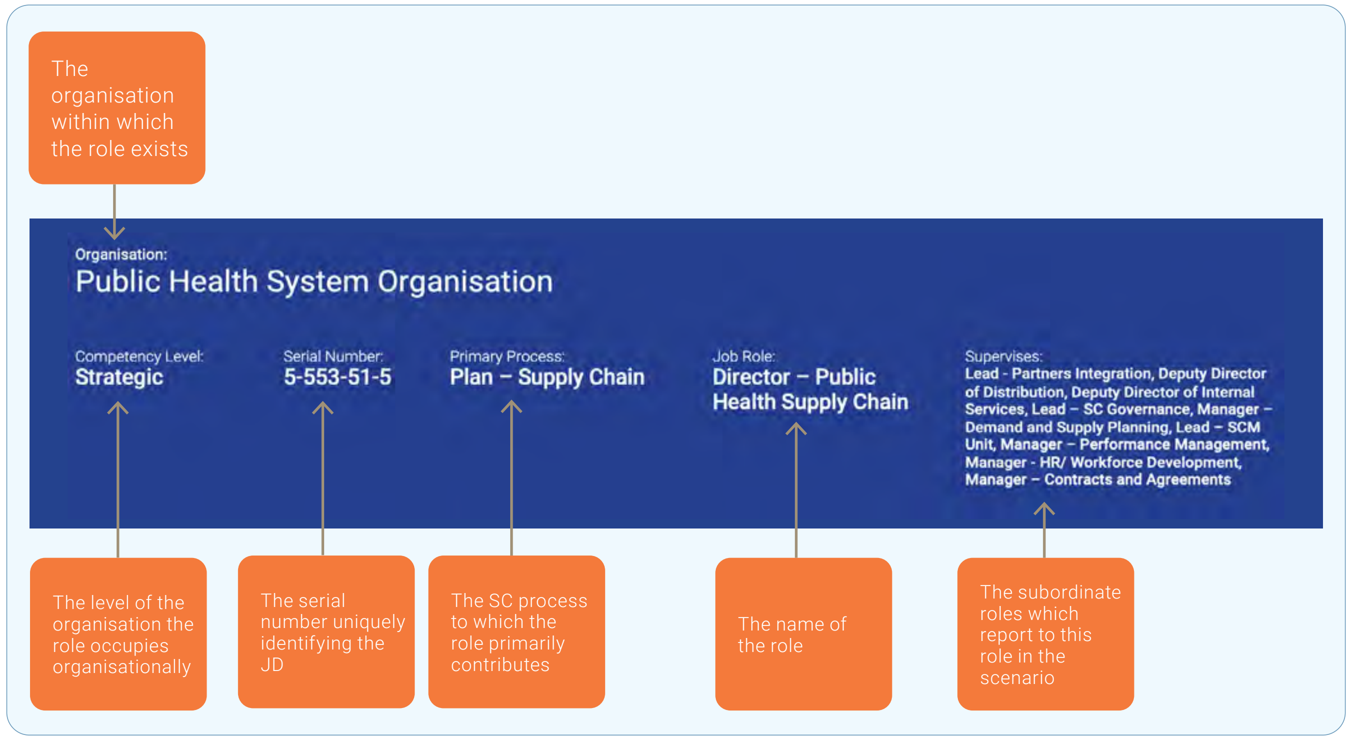

Figure 5:
Description of how the Collection of Roles and Job Descriptions for Health Supply Chains is constructed

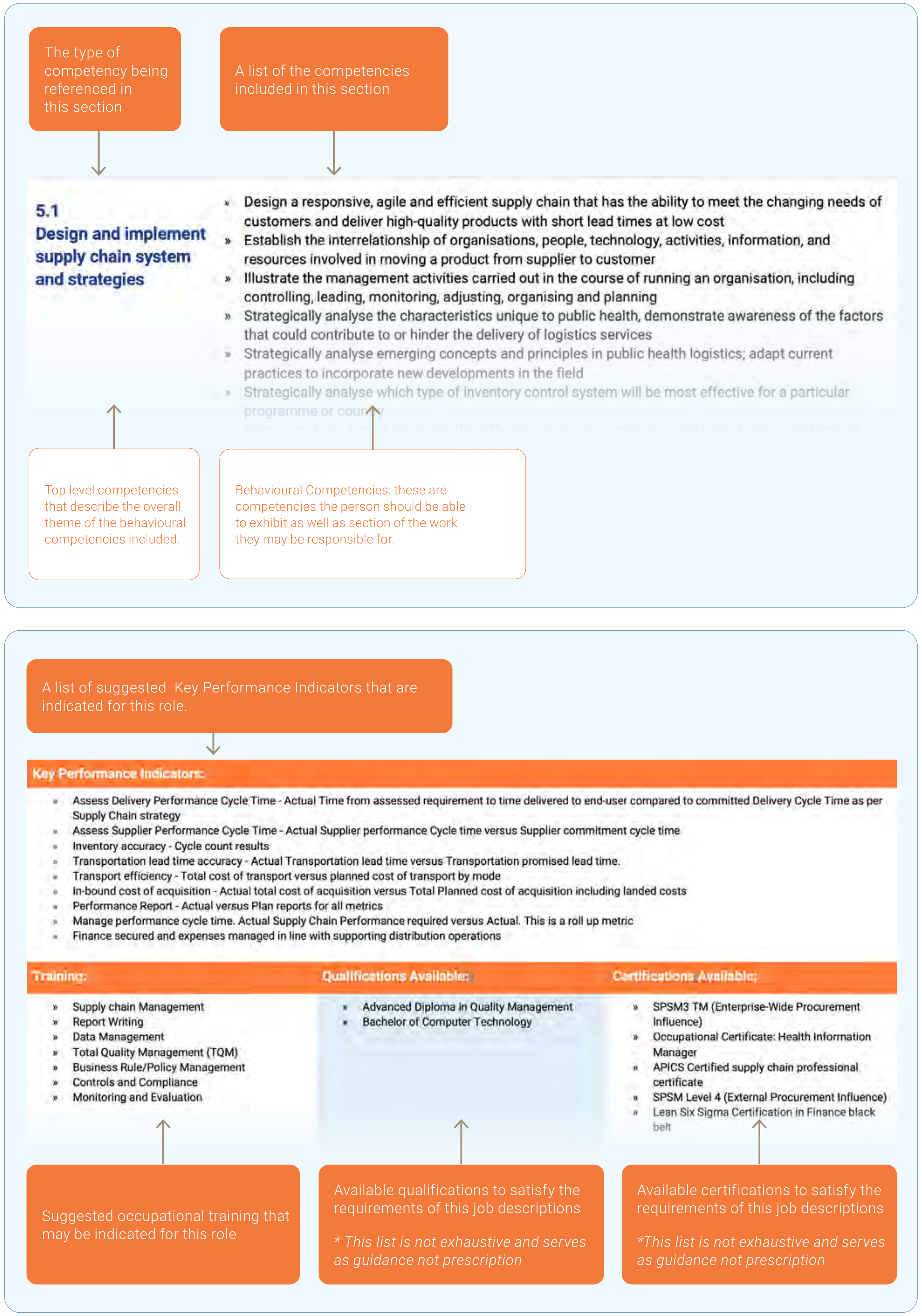

Figure 5:
Description of how the Collection of Roles and Job Descriptions for Health Supply Chains is constructed (cont)

While these are standardised job roles, it is fully expected that countries will alter and merge the JDs according to their needs.

Level Definitions:

Organisations levels are defined in the collection according to the following hierarchy:

Strategic:
Applies strategic, systems thinking; directs and advises, manages change, and influences internal and external stakeholders.

Managerial:
Develops, improves and fulfils organisational and functional objectives; manages efficiency, quality and risk.

Operational:
Provides and executes guidance on procedures and processes that are connected.

Tactical
Executes the process and assists operational levels to perform their overarching duties.

Organisations

Public health system organisation:
This refers to the organisational structures found in public sector health systems established to deliver health services to country populations. The primary purpose of the organisations that collectively make up the public health system is to ensure access to quality care through programmes that target all populations. As access to quality medicines, supplies and equipment is a core component of care, the public health system also ensures that systems are in place to design, procure, deliver and manage supply chains. This collection refers to public health system roles that are involved in ensuring functioning supply chain (SC) systems are in place while not necessarily executing the SC functions (these SC functions and roles are captured under the SC organisation type). Meanwhile, this organisation type includes the service delivery levels of public health systems (such as hospital, clinics and community health posts) that play a role as "customers" of supply chain systems.

The public health system is also typically tiered, where the decision making authority for health and SC programming is the purview of a high level structure (a central body, or a state or regional structure under devolved health systems), and functions and roles are 'cascaded' to lower, intermediate levels (such as regions in the case of centralised public systems or provinces).

Supply chain (SC) organisation:
This refers to the entities involved in carrying out core SC functions to service the needs of the public health system with quality, timely and adequate medicines, supplies and equipment. The SC Organisation may be public sector owned, or a parastatal or a privately owned entity. The collection of roles for this organisation type is intended to reflect the full scope of functions and roles involved to ensure high performing, reliable supply chains. Typically, the SC organisation will include structures in different locations (such as branches or hubs) to more cost-effectively provide SC services closer to populations.

Scenario hierarchy

The Collection of Roles and Job Descriptions for Health Supply Chains displays only one possible organisation of job roles in a country and the authors decided to use a version of a semi-autonomous supply chain organisation. Therefore, it is assumed that the primary supply chain domain knowledge is held by this organisation. Assuming this, the highest level of structure in the public health system would then perform a regulatory role, maintaining the SC organisation's compliance and synchronisation with national priorities.

What this means is that any supply chain role from the intermediate to the community levels will report to the supply chain organisation creating a flow of reporting and information that will align with the public health supply chain operating model. The hierarchy used for this scenario is displayed in Figure 6 below. The organisations in this document are also colour-coded according to the below diagram.

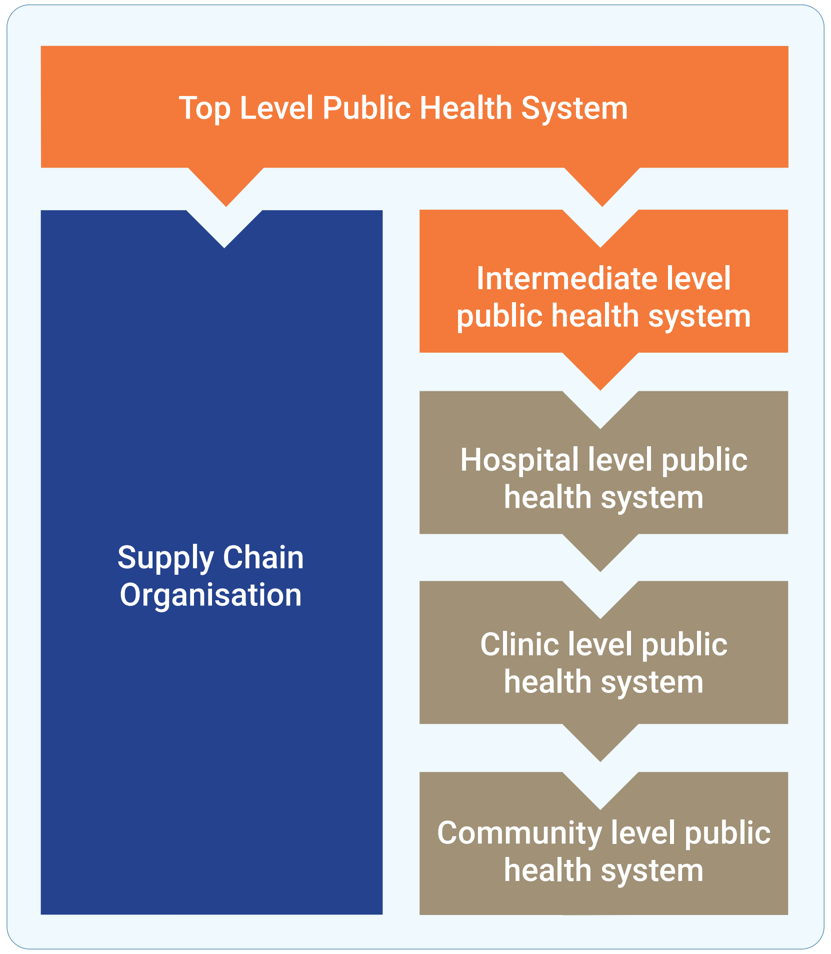

Figure 6:
Depiction of hierarchy used for generation of standardized job descriptions with corresponding colour codes.

Mapping of Education for Health Supply Chains

To improve the supply of supply chain professionals there needs to be significant support from academia as well as vocational training providers, certification and professional bodies in-country. To this end PtD has developed a list of available qualifications and certifications that can assist in acquiring the skills needed to be deemed a professional in the healthcare supply chain sector.

This list is by no means exhaustive nor is it prescriptive, it is only a reflection of courses readily available to the authors at the time, which directly correlate with the skills and competencies specified in the Competency Framework and job descriptions. It includes more than 250 courses from various providers around the world.

In the Education Framework document, the user will find a similar structure to the Competency Framework, the difference is that there are no competency descriptors but rather in their place, a list of courses that have exhibited content for those competency descriptors.

The Education Framework is designed to give the user an indication of the types of education available to fill the skills gaps found in the country, but it is recognised that each country may have its own regulations and prerequisites

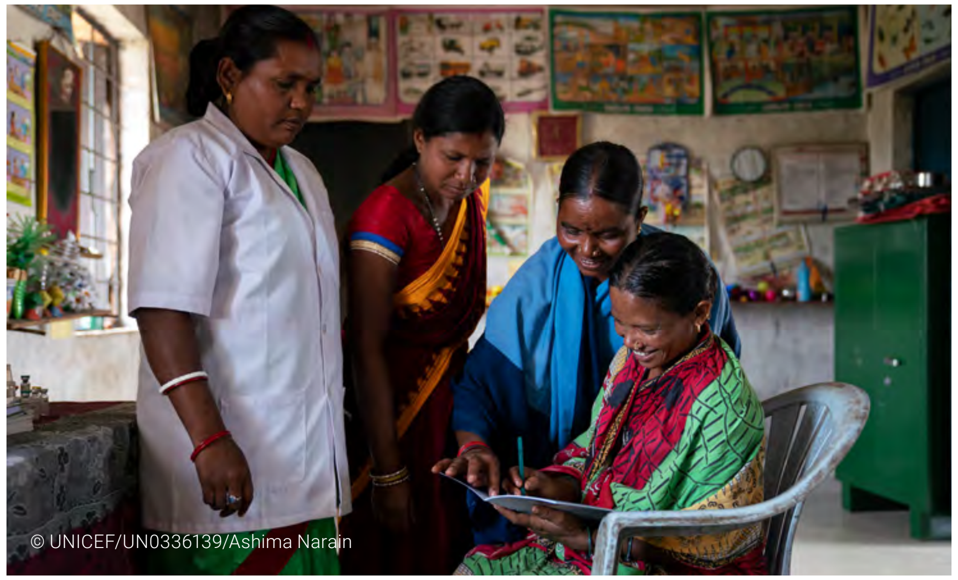

© UNICEF/UN0336139/Ashima Narain

Methodology

In order to develop the Mapping of Education for Health Supply Chains a model needed to be chosen. The authors had a firm grasp and easy access to one of the leading education frameworks in Africa, namely the South African Qualifications Authority (SAQA). SAQA contains more than 14,000 qualifications for evaluation spanning various subjects, including but not limited to public healthcare (see Appendix C: SAQA structure).

Thus, because this dataset was non-specific, the first step was to filter out irrelevant courses. The competency framework was used as a first-pass filter: anything that did not directly relate to the competency framework was removed, leaving 436 courses that related to the seven domains of the competency framework.

The competency framework comprises more than 3,000 competencies across the levels and behavioural competencies. To speed up the process a set of natural language processing (NLP) algorithms was developed and deployed against the dataset.

The NLP algorithms were used to compare behavioural competencies with the course description, outcomes and overall information contained in the SAQA course records. Where there was significant overlap, the course was said to match the competency. Only the top 5% of matches were kept as candidates for the Mapping of Education for Health Supply Chains. Once the first pass with SAQA was completed, certificate courses were added to this list and assigned in a similar method. This had two effects: one was to expand the dataset and the other was to partially validate the model as these certificate courses are more focussed than the degrees from SAQA and easier to validate. The results of this validation were that less than 2% of allocations made for the certificate programme were judged to be erroneous.

At this point a candidate list was created including SAQA and various certificate courses. These courses were compared to the competencies that matched them and removed where inaccuracies were observed to be less than 5%, validating the model and finalising the Mapping of Education for Health Supply Chains. The full list of course sources can be found below.

Courses are listed from:

- Coursera
- MIT
- edukazi.com
- South African Qualifications Authority
  (All registered qualifications in South Africa)
- Empower
- CIPS

› CILT
› I+ Solutions
› ASCM/APICS
› SAPICS
› Next Level Purchasing Association

If you are a training provider, your courses may be included in this list by contacting PtD and supplying the requisite information to be added to future revisions of this document.

An example of how to navigate this framework is displayed below:

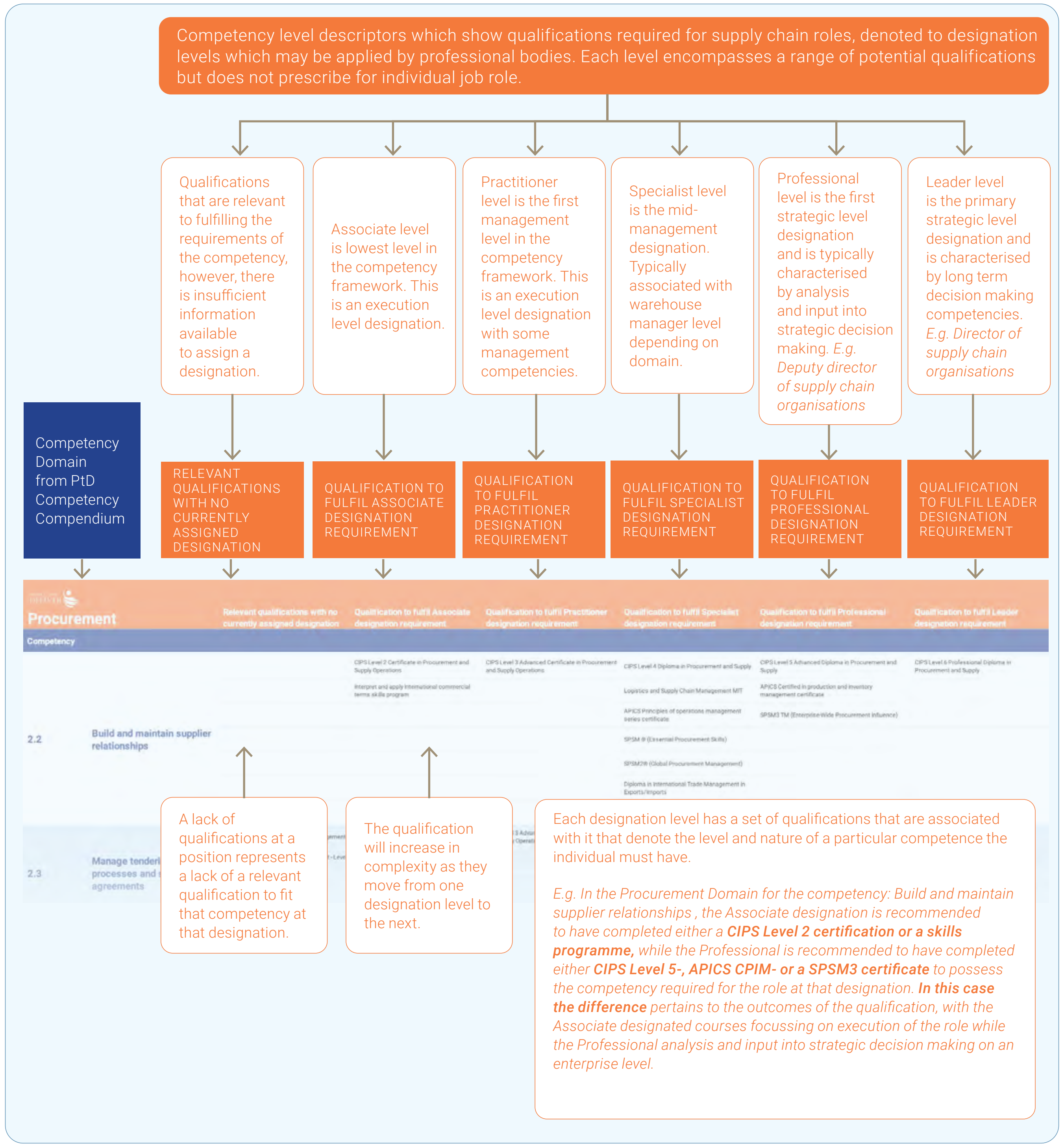

Figure 7:
Description of how the Mapping of Education for Health Supply Chains is constructed

A note on GHSC-PSM

GHSC-PSM recognises that without a strong, skilled workforce at the national and local levels, system-based and technological improvements won't have their intended effect. Achieving a well-performing, motivated workforce requires strengthening the organisational systems, processes, and environment in which supply chain workers perform their duties.

The GHSC-PSM vision is to foster self-sufficient organisations with institutionalised systems. This in turn will help ensure high performance from a professionalised and consumer-centred workforce for effective and efficient delivery of health commodities through to the last mile.

The project goes beyond basic capacity building to look at long-term solutions to organisational and people development, considering the development of human resources systems as an investment. We do this by providing technical support to continuously improve the systems, processes and factors affecting an organisation's ability to plan for, manage, and support professionalised national cadres of supply chain professionals.

A note on People that Deliver

With more than 250 organisational members globally, PtD advocates interventions that improve the demand and supply of a qualified health supply chain professional in organisations, which in turn strengthens the individual practitioners within those organisations. Since 2011, PtD has contributed significantly to the human resources body of knowledge available for health supply chain practitioners. As custodians of the stepped-approach toolkit, which features the Competency Compendium for Health Supply Chain Management, PtD was an obvious partner in considering an SCM professional framework.

A note on SAPICS

SAPICS has taken the step to professionalise SCM in South Africa by assuming the role of the professional body for supply chain management. Having served the profession for 50 years, it is well positioned to provide the services of the professional body that will see it uplift supply chain management as a profession as well as the practices and people within it. It also assists in fostering relationships with government to assist in addressing strategic imperatives for economic transformation in South Africa and the continent of Africa more broadly. SAPICS awards professional designations based on technical supply chain competencies.

Implementation approach objectives

The goal of this approach is to provide a guided set of activities for improvement teams to follow and in so doing, leverage the created workforce development tools (Library of Competencies & Designations for Health Supply Chains, Mapping of Education for Health Supply Chains, Collection of Roles and Job Descriptions for Health Supply Chains) to create and implement an improvement programme capable of achieving sustainable supply chain improvement through workplace and people enablement (SCM professionalisation). By applying this approach, it becomes possible to catalyse the whole SCM labour market by addressing both the supply and demand factors through a focus on competency requirements and adjusting the structure and elements of the government systems to catalyse and improve the health supply chain management labour market.

The approach enables the country implementation team to achieve these objectives by:

1. Providing a basis for continuous supply chain improvement by examining supply chain processes, workforce components and validating their alignment.
    a. With knowledge of current supply chain activities, the team is able to compare supply chain workforce development activities, at all levels, to those of a common baseline set and identify gaps in the current workforce skills and capabilities in order to promote remediation in critical performance areas.
2. Embedding continuous learning and skills enhancement practices which are focused on improving supply chain performance through workforce development.
    a. With a standardised approach to increasing supply chain performance through workforce development, this approach seeks also to foster alignment between private and public sector actors, academic and vocational training institutes and professional bodies and associations.
    b. Catalyse the supply chain labour market and make structural change in a country context

The approach links process elements through a competency model, job descriptions, key performance indicators, education pathways and a workforce-enabling implementation methodology in a unique standardised format using the SCM Professionalisation Framework tools.

The unique nature and power of the approach is largely derived from using these elements together with a predefined relationship between each enabling tool.

The Implementation Approach: Introduction

The racetrack (Figure 8) is a high-level diagram describing the five phases necessary to complete a supply chain workforce improvement programme. The steps are as follows:

1. Advocacy
2. Define the Scope
3. HR4SCM Building Blocks
4. Improve
5. Implement and Monitor

The racetrack diagram denotes a linear progression path for project teams to follow, with each step and gate being completed before moving on to the next step. Workforce development and supply chain improvement, however, is never fully completed therefore it is best conceived as a continuous cycle of workforce improvement projects.

Whilst the scope and objectives of an individual project's initiatives may differ, the approach provides a configurable, standardised repository of workforce tools, templates and best practices to guide improvement teams.

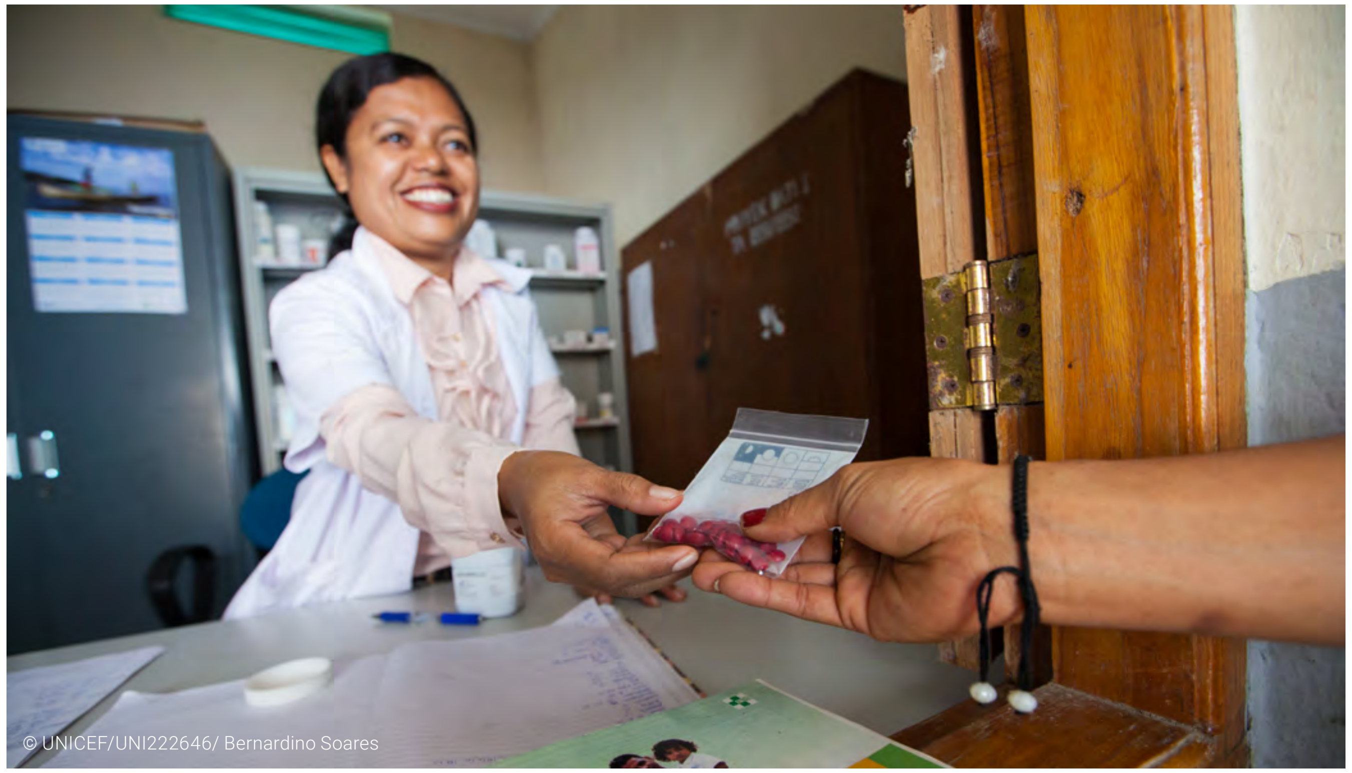
© UNICEF/UNI222646/ Bernardino Soares

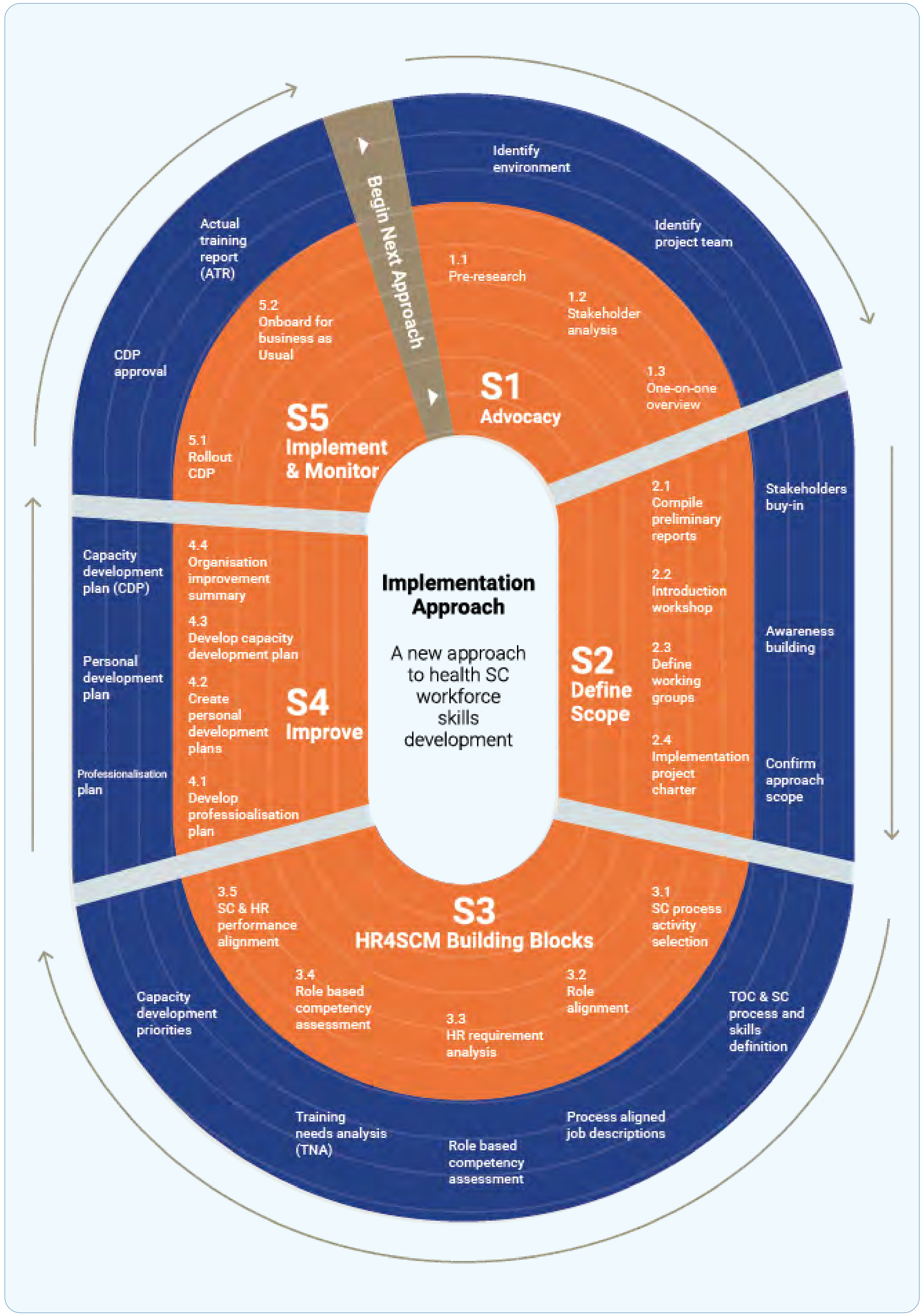

Figure 8:
Full Implementation Racetrack

Step 1 (S1) - Advocacy:

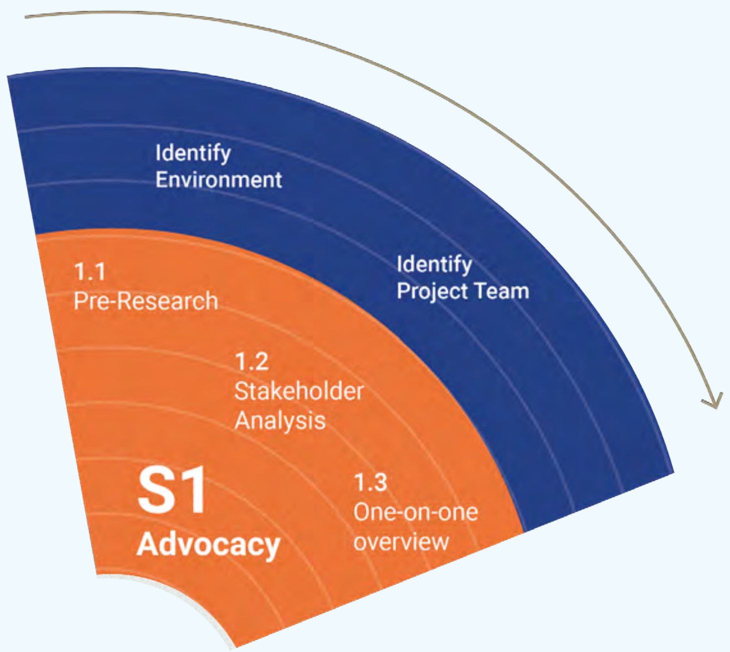

Key objectives

— Identify environment

— Identify project team

Deliverables

— Pre-research output

— Stakeholder analysis

— On-on-one overviews

The first step of the implementation approach is to secure buy-in from stakeholders. There are several activities that will ensure the success of the implementation at this stage. This step assumes that the country or organisation has expressed interest in professionalising its public healthcare practitioners.

The primary output of this step is to define and obtain:

- Motivations and outcomes
  - What are the individual team motivations for change?
- Initial stakeholder map outlining top 3 individuals key to success
  - Generate a formal document defining the responsible roles in the improvement programme and which individuals occupy each role.
- A project champion and sponsor
- Permission to move forward into scoping

Tools

- Advocacy collateral (pamphlets, presentations for communication of ideas, email templates)
  - To assist in initial communication of the need for professionalisation.
  - To communicate the process of professionalising the healthcare supply chain workforce.
- Stakeholder responsibilities mapping template

Resourcing

This S1 Advocacy activity requires the availability of two main individuals with a third being identified by the end of this step:

- Coach
  - The coach understands the need for professionalisation, knowledge of the implementation approach and the professionalisation approach.
  - This individual will also act as the project lead and will offer most support to the in-country representative. Much of the S1 process should occur before the coach arrives in country.
  - This individual would typically be external to the country or organisation where professionalisation is needed.

- In-country representative (ICR)
  - This individual is located within the country or organisation where implementation is being explored. The individual has a good knowledge of the power structures, stakeholders and processes of the government in-country. The ICR will facilitate the engagement of the coach with the decision-making stakeholders in-country.

  This individual would typically come from an organisation within the country. For example, the chairperson of the pharmaceutical council, the head pharmacist of the ministry of health or central medical stores, or an academic in pharmacy or supply chain management from an in-country academic institution.

- Sponsor
  - This individual is a member of the country leadership or organisational executive team. The sponsor works with the implementation team, typically assisting with matters such as funding, clarifying the scope, monitoring progress and influencing stakeholders in order to benefit the improvement programme. This individual performs the following activities:
    - Reviews and approves recommended changes proposed by the implementation
    - Champions and initiates the professionalisation improvement programme
    - Obtains and maintains commitment to the implementation programme from the senior management team
    - Champions the implementation programme within all levels of the country and supply chain

Activity sequencing:

1. Typically, the ICR will approach the coach with a need for professionalisation in their country. During this initial engagement the coach will assist the ICR in designing an initial desktop study pertaining to the required elements of the healthcare sector for the country or organisation.
2. Once the ICR and coach have established contact and concluded the desktop study, the ICR is able to begin advocating, in their environment, for the need for professionalisation using the results of this study as a guide for who to target first. It is essential that the ICR be supported by the coach. The ICR may also want to use the advocacy collateral to communicate the need more effectively to the individuals identified.
3. ICR polls the leadership stakeholders for tacit acceptance of engagement and begins to document the initial motivations for engagement from the various elements within the country.
4. Output for this is the identification of decision-making stakeholders and permission to begin country or organisation engagement with the coach directly.
5. The ICR and coach should have one-on-one discussions with the decision making stakeholders identified to obtain their individual buy-in. This could be done via phone or video call.
6. One of these stakeholders must become the executive sponsor for the project, the role of this individual is to remove barriers from the top level for the project team and get permission to move on to scoping.

Outputs of Step 1:

— Identification and onboarding of sponsor

— Authorisation and plan for the next programme phase

These steps encompass just one scenario and are indications of the types of activity required to get to the outputs of Step 1.

Step 2 (S2) - Define Scope:

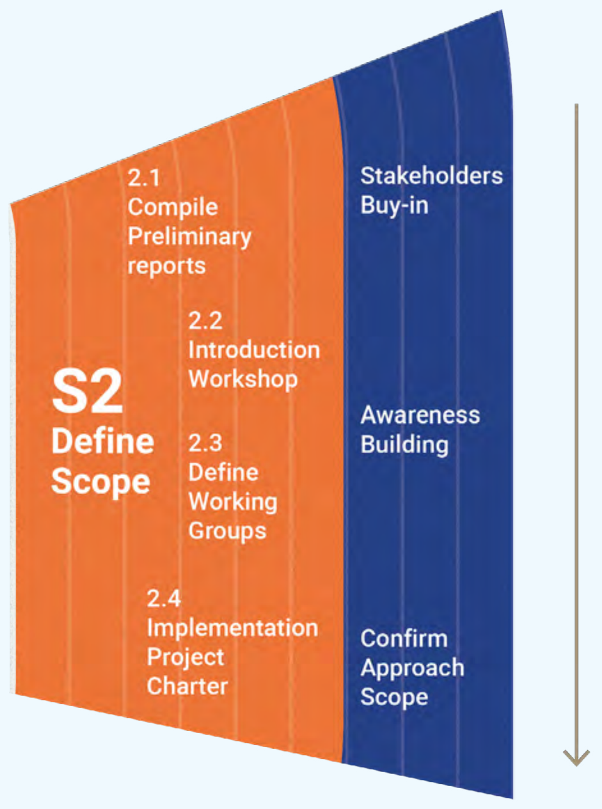

Key objectives

— Stakeholder buy-in

— Awareness building

— Confirm approach scope

— Authorisation to implement

Deliverables

— Compile preliminary reports

— Introduction workshop

— Define work groups

— Project charter

This step provides the approach scoping activities which define the work activities that need to be included in the project. The objective of this step is to develop a project charter that outlines the team for implementation, key stakeholders and the role of relevant professionals in government and professional structures. Additionally, this step provides a basis for stakeholder buy-in, awareness building and the definition of working groups required during the further implementation steps.

The final activity will result in the presentation of the implementation plan to the project sponsor and an implementation case (Project Charter) for dissemination to the leadership structures of the public health organisation in the country.

Tools:

— Introductory workshop materials (slides, guidebook, handouts)

› To communicate the details of the process of professionalising the healthcare supply chain workforce using the SCM Professionalisation Framework

› To detail the types of stakeholder engagement required for Steps 3-5 on the stepped approach

› To get final buy in from stakeholders for the professionalisation of the healthcare supply chain workforce

— Sample project plan

› Outlines typical engagement requirements including Steps 3 – 5 activity sequencing, duration and level of effort for core project team and working groups

— Project Charter template

› The outline of the implementation case detailing the points that should be covered as a minimum to communicate the Project Charter.

› The Project Charter will describe the project in its entirety including goal, objectives, how the project activities will be carried out and who the stakeholders are.

— Implementation presentation template

› Encompasses summaries of the introductory workshop materials to bring high level executives into the project quickly.

› To communicate the elements included in the project plan for consumption by the highest-level decision makers in the country or organisation.

› Designed to assist in obtaining final approval to proceed with Steps 3-5 of the stepped approach.

Activity sequencing:

1. Before the coach visits the country the last of the research and preliminary reports need to be drawn up; this could be done by further desk studies and interviews with individuals from within the elements identified in the previous step. The collection of this data may require the assistance of the sponsor in order to get adequate participation. The coach, ICR and sponsor will need to acquire at least the following information:

   › Supply chain processes

   › Educational landscape

   › Human resources standards for progression e.g. public service standards

   › Professional organisations, bodies and councils, their scope, authority and understanding of general processes

2. From this information the leadership stakeholders should be updated from the previous step to make sure that all the supply chain and workforce disciplines are represented in the workshop following this step. This stakeholder analysis will also form the core of the working group definition later in the process.

3. The ICR will now attempt to arrange a meeting with these stakeholders during which the coach will host an introductory workshop to raise awareness of the need for professionalisation and buy-in. These stakeholders and/or members from their team will be included in conversations about the working groups, the results of which will be included in the final plan. At this point the coach will be in full engagement in-country.

4. Once buy-in is attained, additional one-on-one discussions can be held to explain to stakeholders what to expect during the further implementation steps, attain further design input and finalise team structure. These discussions will also be important to obtain final buy-in and decide the team members to be proposed for the working groups. During this step, the detailed project plan can also be completed with the working group commitments.

Potential working groups:

— Policy

— Education and academia

— Supply processes (operating model)

— Technology

— Human resources/job description definition

— Professional associations

— Change management

Once a draft project team staffing plan has been developed an additional consensus building session should be held to inform all involved how the working groups may work holistically. This could be a one-day workshop.

At this point it should be possible to conclude any final engagements that arise as well as begin to complete the final Project Charter. Depending on how long this takes the coach may still be in-country and can work closely with the ICR to develop the Project Charter and final presentation.

After the Project Charter and final presentation is completed it is advised that a session be conducted for the sponsor to align thinking and create a strategy to obtain buy-in from the top healthcare structures.

The final step should be to conduct a session or series of sessions with the healthcare leadership structures to authorise Step 3 of the stepped approach.

Outputs of Step 2

- Completion of Project Charter
- Authorisation and Project Charter for the next programme phase

These steps encompass just one scenario and are indications of the types of activities required to accomplish the outputs of Step 2.

The Project Charter will be used to guide the later activities contained within the Racetrack and Implementation approach. It contains the scope of the capacity building requirements and details the enabling actors and environment under which this change can be supported. For example, there could be country wide scope with the aim of aligning and catalysing supply and demand factors for health supply chain management professionalisation or an organisational focused (private or public) narrowing scope considering what can be influenced regarding supply and demand factors and alignment with SCM processes within the organisation.

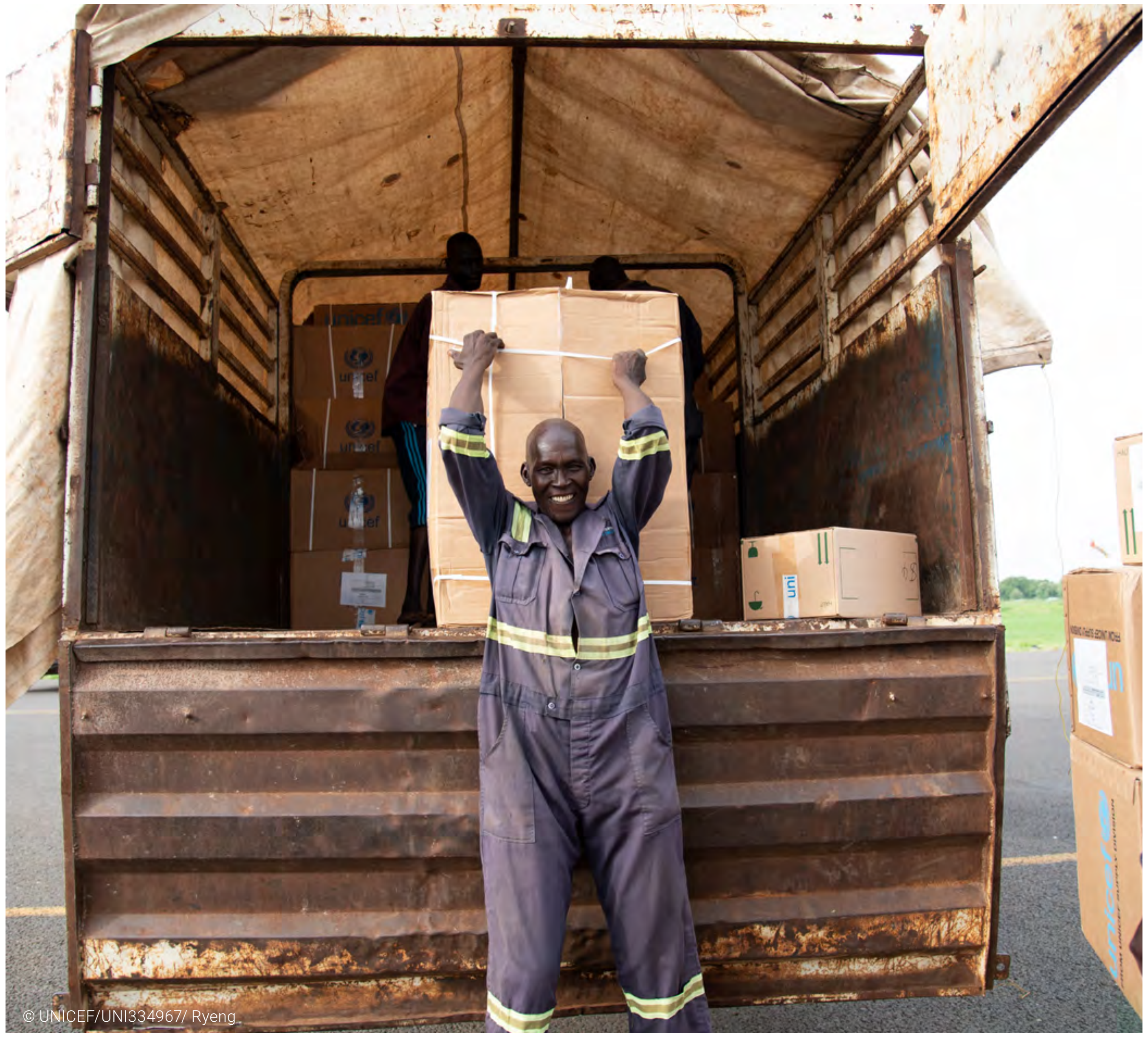

© UNICEF/UNI334967/ Ryeng

Appendix A

Overview of Steps 3 to 5

3.1 SC process activity selection

Objectives

- Align current in country supply chain activities with the competency framework
- Agree on a champion organisation for professional body (must have the mandate to represent the profession)

3.2 Role alignment

Objectives

- Translation of SCM process to role and alignment of roles with designations and careers paths

3.3 HR requirements analysis

Objectives

- Define gaps discovered through the alignment of roles and competencies with processes

3.4 Role based competency assessment

Objectives

- Roll out competency-based assessments and define competency gaps

3.5 SC and HR performance alignment

Objectives

- Define baseline metrics for individuals and supply chain performance based on selected KPIs

4.1 Develop professionalisation plan

Objectives

— To develop the project plan for professionalisation including designation, education and mentorship

4.2 Create personal development plans

Objectives

— Creation of personal development plans from the capacity development plan

4.3 Develop organisational capacity development plan

Objectives

— To quantify and plan organisation-wide education and designation activities and balance available resources

4.4 Organisation improvement summary

Objectives

— Linking Professionalisation with intended organisational performance for the executive. Embedding professionalisation into the DNA of the organisation.

— To obtain confirmed buy-in from the executive with the required resources to develop the plan.

5.1 Roll-out approved organisational capacity development plan

Objectives

— Roll-out approved organisational capacity development plan

5.2 Onboard for business as usual

Objectives

— Integrate and create formal structures to manage the ongoing professionalisation.

PEOPLE THAT DELIVER

People that Deliver

@Pplthatdeliver

www.peoplethatdeliver.org
