# Supplementary material for: Presenting a Framework to Professionalize Health Supply Chain Management
Source: Glob Health Sci Pract. 2025 May 9;13(Suppl 1):e2300119. doi: 10.9745/GHSP-D-23-00119 (PMC12063754; doi:10.9745/GHSP-D-23-00119)
Supplement: GHSP-D-23-00119-Meier-Supplement3.pdf [file GHSP-D-23-00119-Meier-Supplement3.pdf]

SCM Professionalisation Framework

COLLECTION OF ROLES AND JOB DESCRIPTIONS FOR HEALTH SUPPLY CHAINS

NOVEMBER 2020

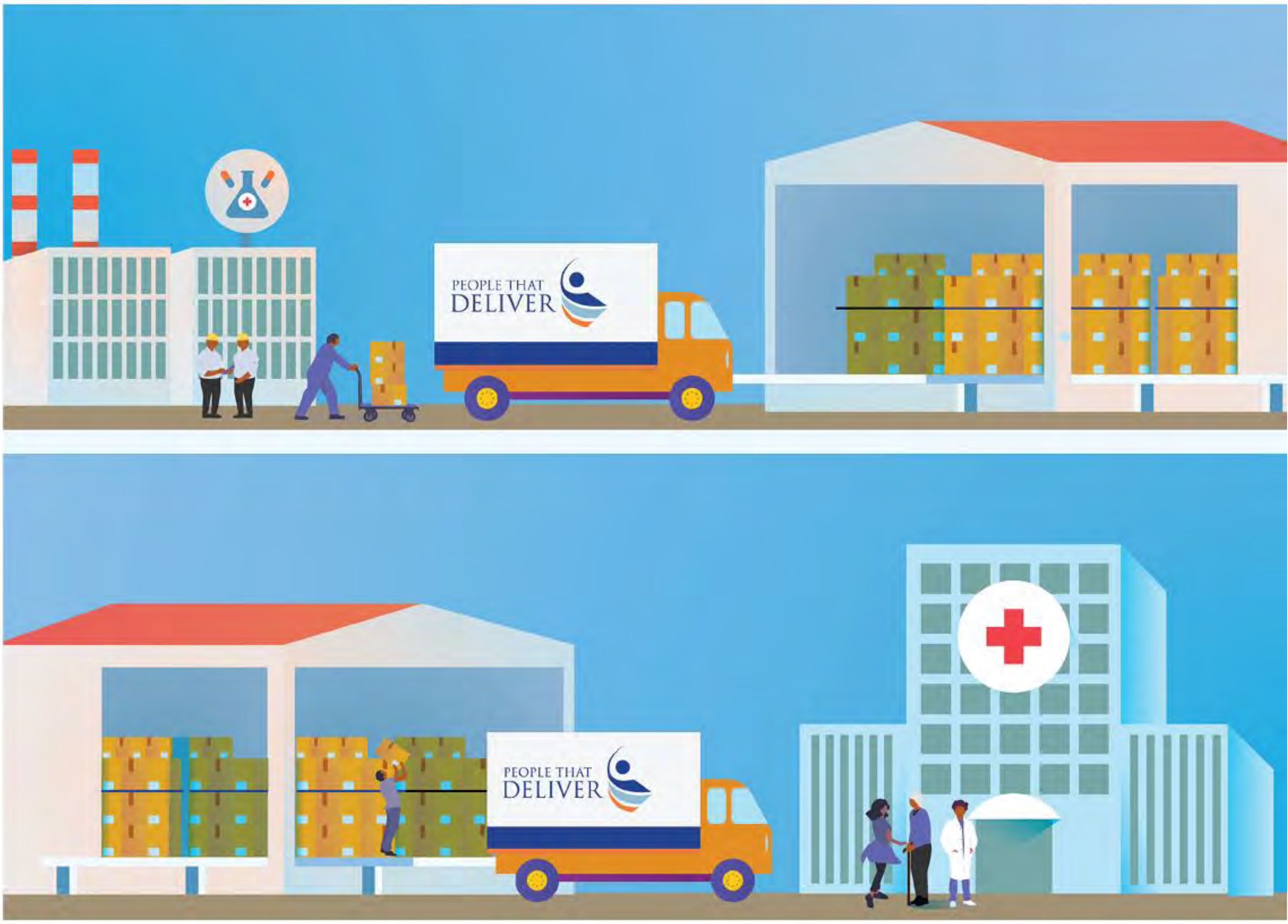

PEOPLE THAT DELIVER

Authors

Andrew dos Santos,
MPhil, BSc (Hons)., SCOR-P, CPIM
Director, CLX
Pretoria, South Africa
andrew@clx.co.za

Richard dos Santos,
BBA, CPIM, CSCP
Director, SAPICS
Copenhagen, Denmark,
richard@sapics.org.za

Bridget McHenry,
MPH, CSCP, Senior Organisational Development Advisor
Commodities Security & Logistics Division, Bureau for Global Health | Office of Population & Reproductive Health USAID
Arlington, Virginia, USA.
bmchenry@usaid.gov

Michael Egharevba
BPharm, MBA, MPH, PGDSCM, PGDDA, SPSM, SPSM2
Workforce Development Specialist
USAID Global Health Supply Chain-Procurement Supply Management (GHSC-PSM) Project Arlington, Virginia, USA.
megharevba@ghsc-psm.org

Dr Andrew Brown,
PhD, BPharm, GCHE
Senior Director Health Workforce Development (PtD Coalition Member)
IntraHealth, Chapel Hill, North Carolina, USA.
abrown@intrahealth.org

Dominique Zwinkels
Executive Manager, People that Deliver (PtD)
Copenhagen, Denmark
dzwinkels@unicef.org

Barry Chovitz
USAID Global Health Supply Chain-Procurement Supply Management (GHSC-PSM) Project
bchovitz@ghsc-psm.org

James Johnson
USAID Global Health Supply Chain-Procurement Supply Management (GHSC-PSM) Project
jajohnson@ghsc-psm.org

Hany Abdallah
U3 SystemsWork International, LLC
Washington, DC, USA
hany@u3systemswork.com

Acknowledgements

This work was funded by the Global Fund to Fight AIDS, Tuberculosis and Malaria and USAID Global Health bureau's Family Planning and Reproductive Health (FP/RH) programme. This work was a joint activity of the USAID Global Health Supply Chain Programme-Procurement and Supply Management (GHSC-PSM) project, People that Deliver (PtD), SAPICS, and USAID.

The authors would like to thank the members of these organisations for their contribution and cooperation as together we seek to increase the availability of medicines to beneficiaries.

A special thanks to the following individuals who volunteered their time for interviews, to engage in focus group discussions, and/or provided feedback for this work; we thank you for your contribution.

Adebayo Adekola, Capacity Development Specialist, Supply Chain Strengthening Centre, UNICEF Supply Division

Narcisse Agbessi, Specialist in inventory management and logistics at the National Malaria Control Program of Benin

Herman Mawuéna Bonou, Business Analyst and Supply Chain & Logistics Specialist, Benin

Tom Brown, Senior Program Officer, Supply Chain Systems, Bill & Melinda Gates Foundation

Clinton De Souza, Managing Partner, Celsian Consulting LLC

Robert Duncan, Director, B & C Business Services Limited

Dr Bonface Fundafunda, Regional Lead, East and Southern Africa, Africa Resource Centre

Simon Tendai Garikayi, Principal Consultant, Regenerative Customized Solutions

Ingrid Gázquez, Learning and Development Specialist, Supply Chain, the Global Fund to Fight AIDS, Tuberculosis and Malaria

Bernardin Habumukiza, Registered Pharmacist, National Pharmacy Council of Rwanda

Denise Harrison, Senior Market Development Advisor, USAID

Steven Harsono, Head of Public Health, Asia Pacific, IQVIA

Cindy Hayward (Responsible Pharmacist) - DHL Supply Chain

Dave Hudson CPIM DDPP DDLP, S A Coaching: Supply Chain and Operations Specialist and Executive Coach, South Africa

Wanda Jaskiewicz, Project Director, HRH2030/Chemonics

Khadija Jamaloodien, Director of Affordable Medicines, National Department of Health, South Africa

Dr Gamal Khalfalla Mohamed Ali, Senior Technical Advisor, Pamela Steele Associates

Arati Krishnamoorthy, Senior Data Scientist, USAID

Mike Krautmann, Research Manager - Healthcare Initiative, William Davidson Institute

Paul Lalvani, Dean and Director, Empower School of Health

Arthur Lees-Rolfe, Lead Consultant, Pharmaceutical Systems Africa Malawi

Mr GCJ Lubbe (Cobus) Private and Volunteer Consultant

Glenda Maitin, SCOR-P Program Director, Public Health Supply Chain Initiative, Association for Supply Chain Management (ASCM)

Juvenal Majoro, Senior Health System Strengthening Advisor, USAID Global Health Supply Chain-Procurement and Supply Management Project

Dr Chris Masila, Principal Health Partner/Lead Consultant, PractHealth Consulting Ltd

Dr Lloyd Matowe, PhD, Msc, Bpharm, Director, Pharmaceutical Systems Africa, PtD Coalition Chair

Ricardo Missihoun, Certified Lean Supply Chain Leader

Eomba Motomoke, Technical Assistant, Expert in Supply Chain at PNAM in DRC

Maggie Murphy, Technical Advisor, John Snow Inc.

Annette Naude, General Manager, Pharmaceutical & Healthcare Air & Sea DSV South Africa (Pty) Ltd

Philip Oguche, CSCP, Supply Chain Information Systems Advisor, Chemonics International/GHSC-PSM Nigeria

Bolaji Olawoyin, Country Director, Association for Supply Chain Management (ASCM), Nigeria

Grace Toyin Omole, Pharmaceutical Systems Africa Consultant

Walter Proper, Executive Director, International Association of Public Health Logisticians

Kevin Richardson, Chief Executive, Chartered Institute of Logistics and Transport (CILT)

Stanley Robertson, Supplier Manager | Managed Solutions | South Africa, Imperial Logistics

Boris Ruffino, PtD coalition member- Law and HR4SC Specialist - Lean Six Sigma Certified

Sanjay Saha, Health System Consultant

William Henry Schneider (Bill) CPIM, CSSC, HND Ind Eng, HND Elec Eng. ND Eng Chairman, (EREB) Education and Research Executive Board Canada, Program Director, VCARE Canada Value Chain Applications Research & Education Canada

David Sprent, Independent Advisor

Lerato Thebede, Supply Chain & Logistics Subject Matter Expert and CPIM Instructor

Eric Wakaria, Country Director Association for Supply Chain Management, Kenya

The SCM Professionalisation Framework is dedicated to Andrew dos Santos. His hard work and knowledge were central to the development of this resource.

Contents

Authors 2

Acknowledgements 4

Overview 11

Collection of Roles and Job Descriptions for Health Supply Chains (Demand) 13

Mapping of Education for Health Supply Chains(Supply) 13

Implementation Approach for Health Supply Chains 13

Library of Competencies & Designations for Health Supply Chains 14

Competency terminology and definitions 14

Behavioural competencies 15

Competency framework 15

Designation levels 15

Methodology 18

Collection of Roles and Job Descriptions for Health Supply Chains (Demand) 21

Methodology 22

Level Definitions 24

Organisations 24

Scenario Hierarchy 25

High level overview of job descriptions 26

Mapping of Education for Health Supply Chains 30

Methodology 31

A Note on GHSC-PSM 33

A Note on People that Deliver 33

A Note on SAPICS 33

Public Health System Organisation 34

Strategic 34

Director – Public Health Supply Chain 34

Lead – SC Governance 42

Lead – SCM Unit 50

Managerial 57

Lead - Partners Integration 57

Manager – Contracts and Agreements 64
Lead – Compliance and Risk Management 70
Operational 75
Manager - Occupational Safety 75
SC-focused Organisation 80
Strategic 80
Lead – Network Planning 80
Lead – Data Intelligence and Analytics 90
Lead – Process Improvement and SC Adaptability 95
Lead – Planning and Performance 103
Deputy Director of Distribution 113
Deputy Director of Internal Services 121
Managerial 127
Manager – Procurement 127
Manager – Finance and Administration 137
Manager – Data and Use 146
Manager – QA/ QMS 148
Manager – HR/ Workforce Development 155
Lead – Compliance / Internal Audit 163
Manager – Warehouse and Distribution 172
Manager – Customer and Sales 180
Head - Cold Chain Management 187
Manager - Contract and 3PLs 192
Lead - Partners Integration 196
Manager - Logistics Planning 203
Manager – Demand and Supply Planning 210
Manager – Performance Management 220
Manager – SC Data and Analytics 227
Manager - HR/ Workforce Development 236
Manager – Contracts and Agreements 243
Manager – Programmes (Donations) and Global Partnerships 249
Operational 255
Officer– Procurement 255
Officer – Product Selection Use 261
Officer – Supply Planning/ Supplier Management 266

Officer – Inventory and Demand Planning ... 273
Officer – Network Analyst ... 279
Officer – Strategic Procurement ... 284
Officer – Tender Management ... 291
Officer – Contracts ... 299
Officer – Data and Use ... 305
Officer – Data Management ... 311
Officer – QA/ QC ... 313
Officer – Import and Clearance ... 318
Officer – Receiving ... 323
Head – Warehouse and Inventory Management ... 330
Officer – Order Processing/ Management ... 337
Head – Transport and Delivery Management ... 342
Lead – Waste and Returns Management ... 349
Manager - Occupational Safety ... 355
Lead - Demand and Supply Planning ... 360
Lead - Returns Management ... 367
Officer - Logistics Planning ... 373
Specialist – Contracts ... 380
Officer – Performance Monitoring ... 387
Officer – SC Data Quality and Use ... 393
Officer - Human Resources Specialist ... 404
Officer – Programmes (Donations) Procurement ... 411
Officer – SC Data and Analytics ... 417
Tactical ... 427
Assistant – Storage ... 427
Assistant – Delivery ... 433
Assistant – Inventory ... 439
Assistant – Pick, Pack ... 445
Assistant – Receiving ... 449
Assistant - Human Resources Assistant ... 457
Public Health Organisation – Intermediate Level ... 464
Strategic ... 464
Lead – SC Governance ... 464
Managerial ... 471

Head – Demand and Supply Planning 471
Lead - Procurement 479
Lead – Receiving and Inventory Management 487
Lead – Returns and Disposal 496
Manager – Performance Management 501
Lead – Compliance and Risk Management 508
Lead - HR/ Workforce Development 514
Lead – Health SC System Strengthening 520
Operational 532
Officer – Demand and Supply Planning 532
Officer– Procurement 537
Officer – Receiving and Inventory Management 543
Manager - Occupational Safety 551
Officer – Performance Monitoring 556
Tactical 562
Assistant – Storage 562
Public Health Organisation – Hospital Service Delivery 568
Managerial 568
Head – Demand and Supply Planning 568
Lead - Procurement 576
Lead – Receiving and Inventory Management 584
Lead – Returns and Disposal 594
Operational 599
Officer – Demand and Supply Planning 599
Officer– Procurement 604
Officer – Receiving and Inventory Management 610
Manager - Occupational Safety 618
Tactical 623
Assistant – Storage 623
Public Health Organisation – Clinic Service Delivery Level 629
Managerial 629
Lead – Procurement and Supply 629
Operational 637
Officer – Receiving and Inventory Management 637
Officer – Procurement and Supply 646

Public Health Organisation – Community Service Delivery Level 651
Tactical 651
Clerk – Supply 651
Clerk – Receiving and Storage 658

List of Abbreviations

ABBREVIATION	MEANING
**GHSC-PSM**	USAID Global Health Supply Chain Programme-Procurement and Supply Management
**HSCM**	Health Supply Chain Management
**JD**	Job Descriptions
**NLP**	Natural Language Processing
**PtD**	People that Deliver
**SAPICS**	Industry Association for Supply Chain Management in South Africa
**SAQA**	South African Qualifications Authority
**SC**	Supply Chain
**SCM**	Supply Chain Management

Overview

To better understand issues regarding the availability and use of supply chain management (SCM) human resources in low- and middle-income countries, the USAID Global Health Supply Chain Program - Procurement and Supply Management (GHSC-PSM) project, in conjunction with USAID and People that Deliver (PtD), considers that a 'whole of SCM labour market' approach provides a deeper and more holistic understanding of the SCM employment environment.

The 'whole of SCM labour market' refers to the supply and demand for SCM labour in which employees are the supply and employers are the demand in a specific country context. The country context includes urban, regional, and remote environments, and encompasses all the sectors where SCM technical personnel are employed in a country.

Key stakeholders include governments (ministries of labour, education, planning and health), professional associations, academic institutions, the private sector (resources industries, fast-moving goods, health, third-party logistics providers (3PLs) and fourth-party logistics providers (4PLs)), and the humanitarian and development sectors

In 2019 GHSC-PSM, in collaboration with PtD, SAPICS and USAID published a SCM Professionalisation Framework white paper outlining how a SCM professionalisation framework could be used by:

› Governments to define the professional standards of the profession

› Employers to articulate SCM competency requirements and career pathways in their organisations

› Learning institutions to define clear learning and teaching courses, and

› SCM employees to map out a professional career in SCM

In 2020, the same consortium worked together to complete the necessary elements of the 'SCM professionalisation framework'. This framework has a '***Library of Competencies and Designations for Health Supply Chains***' at its core, meeting public and private sector needs. The competency framework then acts as the *standard* to ensure an aligned 'Mapping of Education for Health Supply Chains' (supply) and 'Collection of Roles and Job Descriptions for Health Supply Chains' (demand), for a particular country context. Further, an 'Implementation Approach for Health Supply Chains' provides clear guidance on how to begin this journey of change. Figure 1 shows the interrelationship between these elements.

Although each of the SCM Professionalisation Framework components can be used by itself, most benefit is obtained by using the ***'Implementation Approach for Health Supply Chains'*** to create lasting systems change.

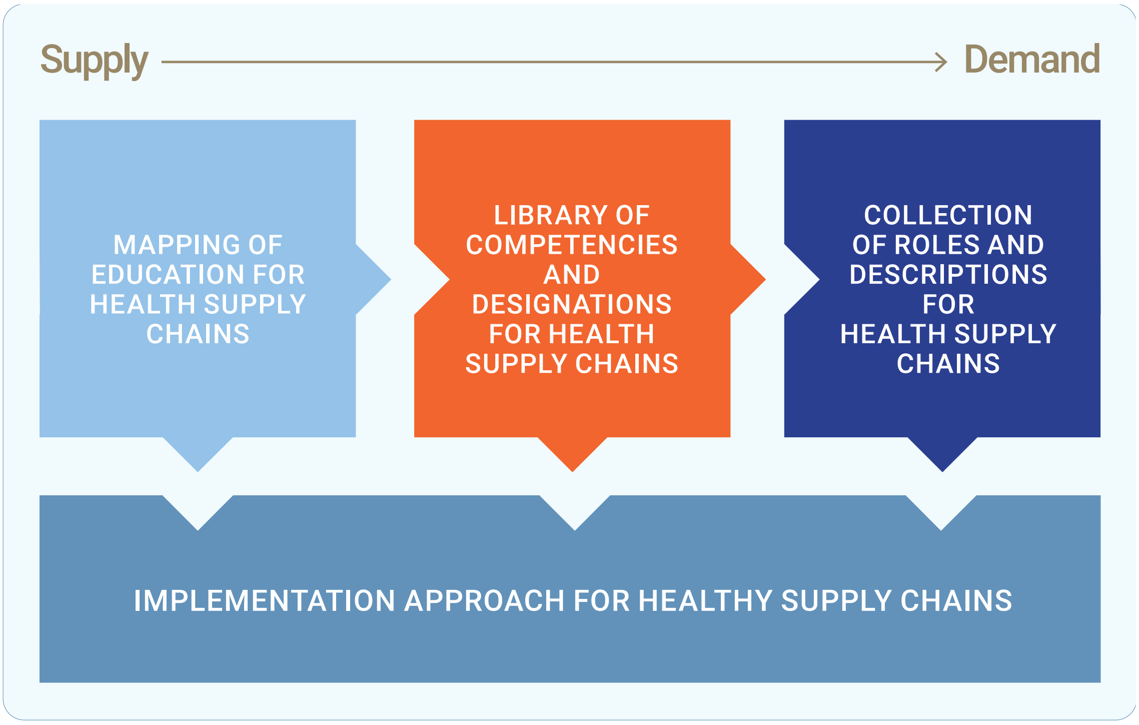

Figure 1:
Elements of the PtD Professionalisation Competency Framework

Library of Competencies and Designations for HealthSupply Chains

Building on the PtD Health Supply Chain Management (HSCM) Competency Compendium (2014), this serviced-based (non-cadre specific) framework presents seven competency domains that cover the practice of HSCM across the public and private sectors. This comprehensive framework provides 'behaviour statements' outlining workplace expectations in all listed competency domains and competency groups. This revised version provides a five-level maturity or designation classification for each competency area, acting as a standard for comparing education requirements and related job descriptions.

Collection of Roles and Job Descriptions for HealthSupply Chains (Demand)

Building on work completed during phase one by PtD in 2018, this tool provides a systematic way to build job descriptions, considering all the SCM roles that exist in health supply chain management. An example set of SCM job descriptions is provided.

Mapping of Education for Health Supply Chains(Supply)

This tool aligns SCM job roles with education and training frameworks. The tool was developed using a machine learning approach which analysed existing SCM education and training opportunities to determine alignment with domains and levels within the Library of Competencies and Designations for Health Supply Chains.

Implementation Approach for Health Supply Chains

This tool provides an overview of the step by step activities that can be undertaken to engage local stakeholders advocating the need to implement a SCM Professionalisation approach. This methodology also validates a plan of action to apply the three components together to systematically improve SCM professionalisation in the country context.

Library of Competencies & Designations for Health Supply Chains

Competency terminology and definitions

Internationally, the use of competency-related terminology can vary, so the following definitions are offered here for common understanding:

Competency compendium: A comprehensive catalogue of competency areas with associated behavioural competencies referencing common supply chain processes and job level activities.

Domains: The high-level groups, or clusters of competency areas, within the compendium. Traditionally, domains do not exceed six. The six domains used in the PtD competency compendium are selection and quantification, procurement, storage and distribution, use, resource management, and professional and personal (Figure 3). The new Library of Competencies and Designations for Health Supply Chains comprises seven domains, including Technology, the newest domain.

Competency area: The overarching capacity and skills a person needs to perform in a specific area. For example, within the domain of procurement, PtD suggests the following competency areas:

2.1 Manage procurement costs and budget
2.2 Build and maintain supplier relationships
2.3 Manage tendering processes and supplier agreements
2.4 Execute management of contract, including risk and quality management
2.5 Assure quality of products
2.6 Manage import and export of products
2.7 Manage donations of products

Behavioural competencies:

The combination of traits, abilities and motivations necessary for effective performance regardless of your position within an organisation.

For example, for the domain of procurement and the competency area of *Manage tendering processes and supplier agreements,* behavioural competencies include 'demonstrate knowledge of local and international tendering procedures', 'ensure ethical behaviour, abiding by conflict of interest policies', 'follow contract approval process, including contract negotiations', or 'award and manage contracts, resolving any issues as they arise'. The PtD competency compendium contains behavioural competencies and states the level and type of knowledge required, depending on the source document used.

Competency framework:

A collection of competency areas with the associated behavioural competencies which define the expected requirements of a particular cadre/profession (Figure 2).

For example, a competency framework may be developed for a warehouse manager, while a separate competency framework would be required for a pharmacist. The composition of country-based, cadre-specific competency frameworks will also depend on the structure of the supply chain and at which levels various competencies are allocated.

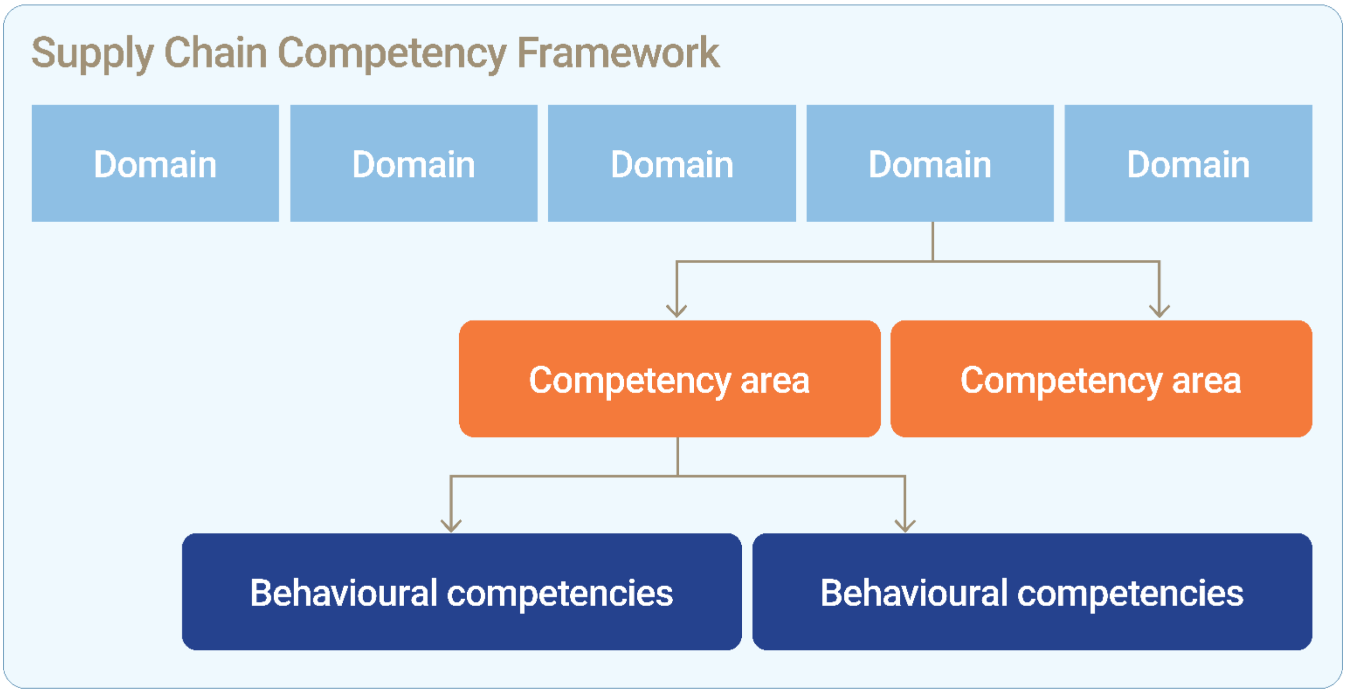

Figure 2:
The structure of competency frameworks

Designation levels:

These define the differing levels of work, focus, and scope, and specify the training and education required at each level. The designation levels build upon one another, starting from the most junior level to the most senior level. Detailed descriptions of each competency area are provided for each designation level to clearly define the requirements needed. The competency framework is divided into the following designation levels:

Associate

Associate level is the entry level of the competency framework. This is an execution level designation.

Practitioner

Practitioner level is the first management level in the competency framework. This is an execution level designation with some supervisory and management competencies.

Specialist

Specialist level is the mid-management level designation, typically associated with management-level accountability depending on the domain.

Professional

Professional level is the first strategic level designation and is typically characterised by analysis and input into strategic decision making.

Leader

Leader level is the primary strategic level designation and is characterised by long-term decision-making competencies.

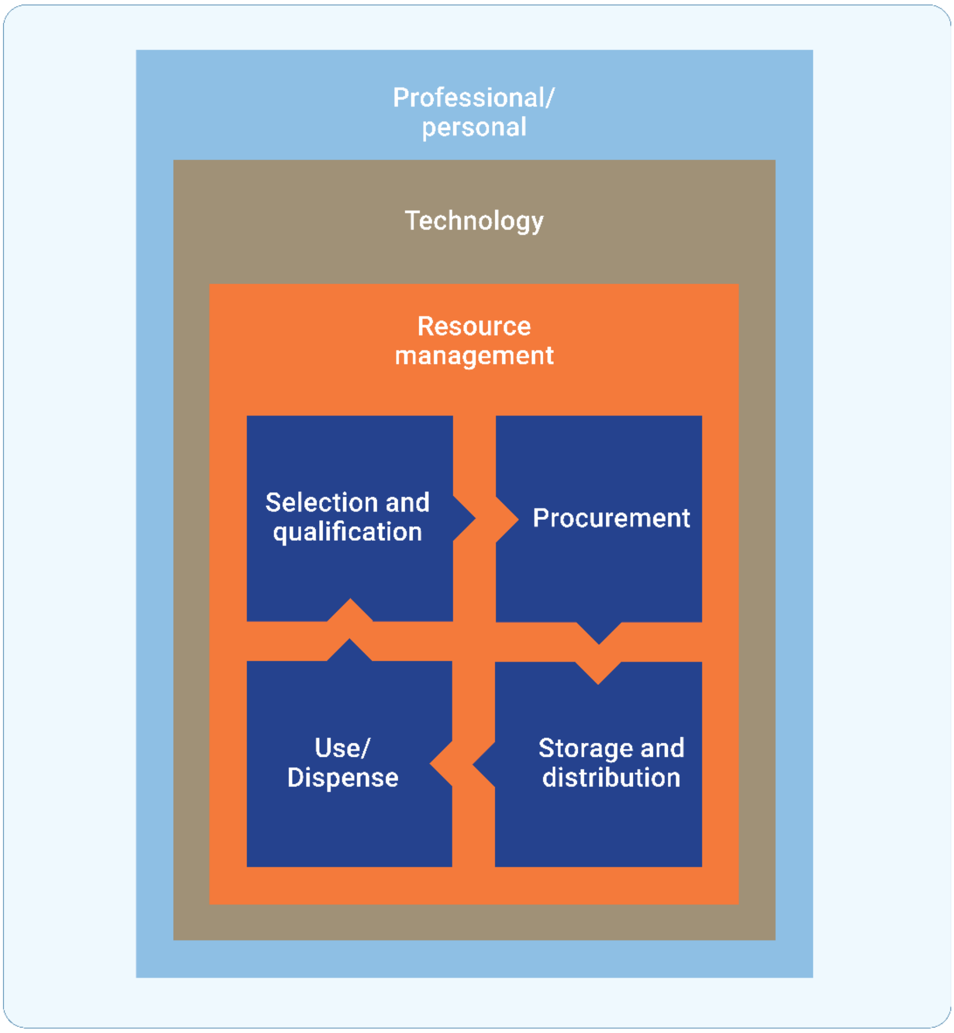

Figure 3:
The relationship of the six domains within the PtD Competency Compendium for Health Supply Chain Management

Each designation level has a set of associated terms which denote the level and nature of a particular competency the individual must possess to fulfil the workplace duties associated with a specific activity. For example, in the Storage Domain the behavioural competency states:

To ensure the accurate verification of rolling stocks, the Associate designation level is required to have an "Awareness of the importance of accurate verification of rolling stocks" while the Practitioner needs to "Understand the importance of accurate verification of rolling stocks".

In this case the difference is indicated by the terms "Awareness" and "Understand". There are several terms used across the competency which have been referenced from multiple educational and vocational resources.

For a detailed description of the competency framework, refer to the Overview of the SCM Professionalisation Framework and the Library of Competencies and Designations for Health Supply Chains.

Methodology

The very nature of a professional body is that it encapsulates a path to professionalisation. For this reason, the PtD Competency Compendium for Health Supply Chain Management is an ideal foundation for the Library of Competencies and Designations for Health Supply Chains. To capture a full pathway, however, the competencies needed to be expanded in responsibility and complexity. Additionally, PtD had already extensively reviewed the document and considered additions to the framework. These additions were added before the expansion of the competencies and then elaborated upon in the same manner as the rest of the framework.

In addition to the expansion, the authors conducted an extensive review, comparing the framework to current best practice supply chain process frameworks to verify its validity in the context of supply chain management. Where necessary, wording was expanded. To build out these designation levels the authors had to first define how many levels would be needed. For this there were few inputs: the first was the structure of other supply chain professional body frameworks and the second was job descriptions and hierarchies from private and public organisations either generously donated or from previous projects. This allowed the framework to be compatible with most hierarchies as well as other professional bodies in the sample set. From these inputs it was determined that five levels would be required to capture the complexity exhibited by these documents, bearing in mind that the levels are in themselves only indicative and can be edited by countries during their own implementation processes.

To expand the PtD Competency Compendium for Health Supply Chain Management into the above-mentioned levels a trusted framework was required. Bloom’s taxonomy of cognitive and affective domains was used to increase the complexity of the competency. The previous analysis using supply chain frameworks was used to increase responsibility using the appropriate terms associated with the domain and aligned to the designation. The final domains chosen are named accordingly and are aligned to Bloom’s taxonomy.

- Associate
- Practitioner
- Specialist
- Professional
- Leader

Table 1: Alignment of Blooms Taxonomy to Professional Designations

Knowledge	Comprehension	Application	Analysis	Evaluation	Synthesis
Associate	Associate	Practitioner	Specialist	Professional	Leader

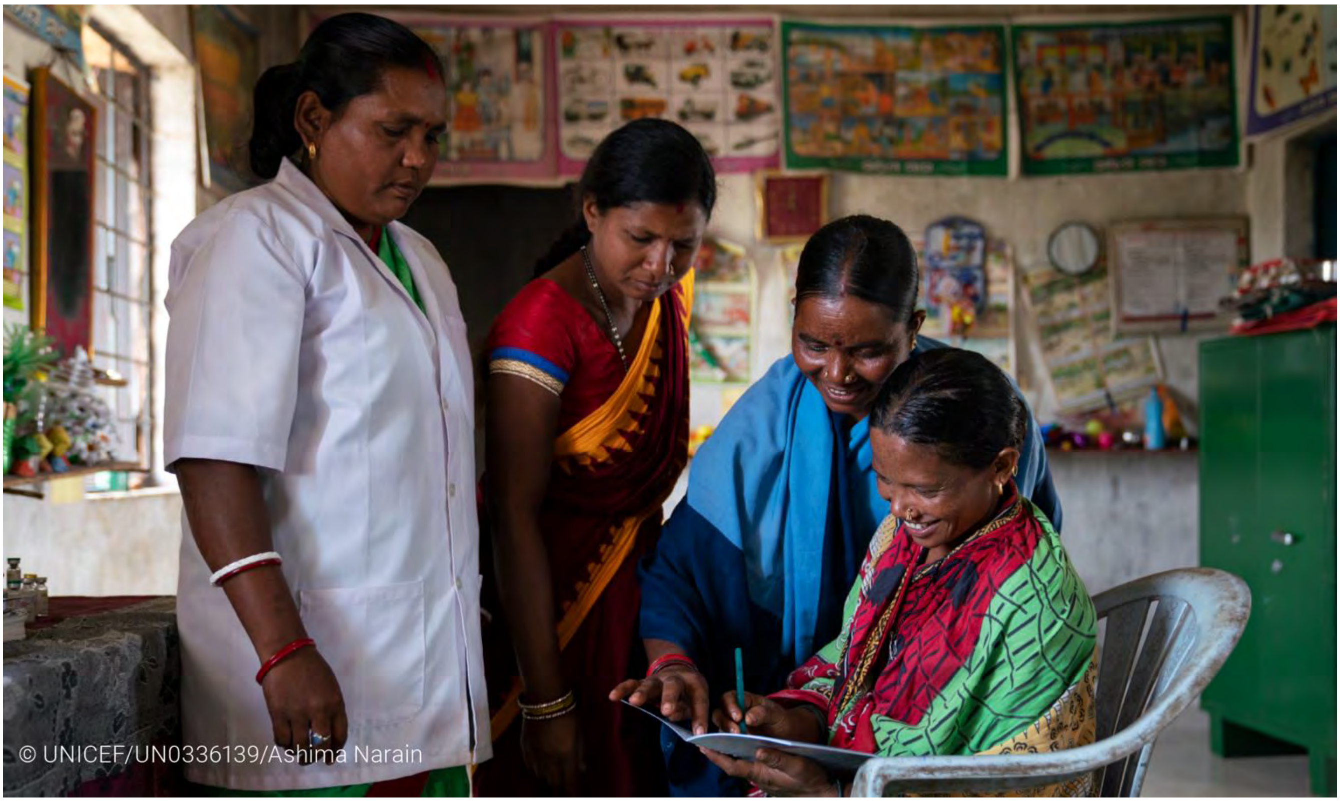
© UNICEF/UN0336139/Ashima Narain

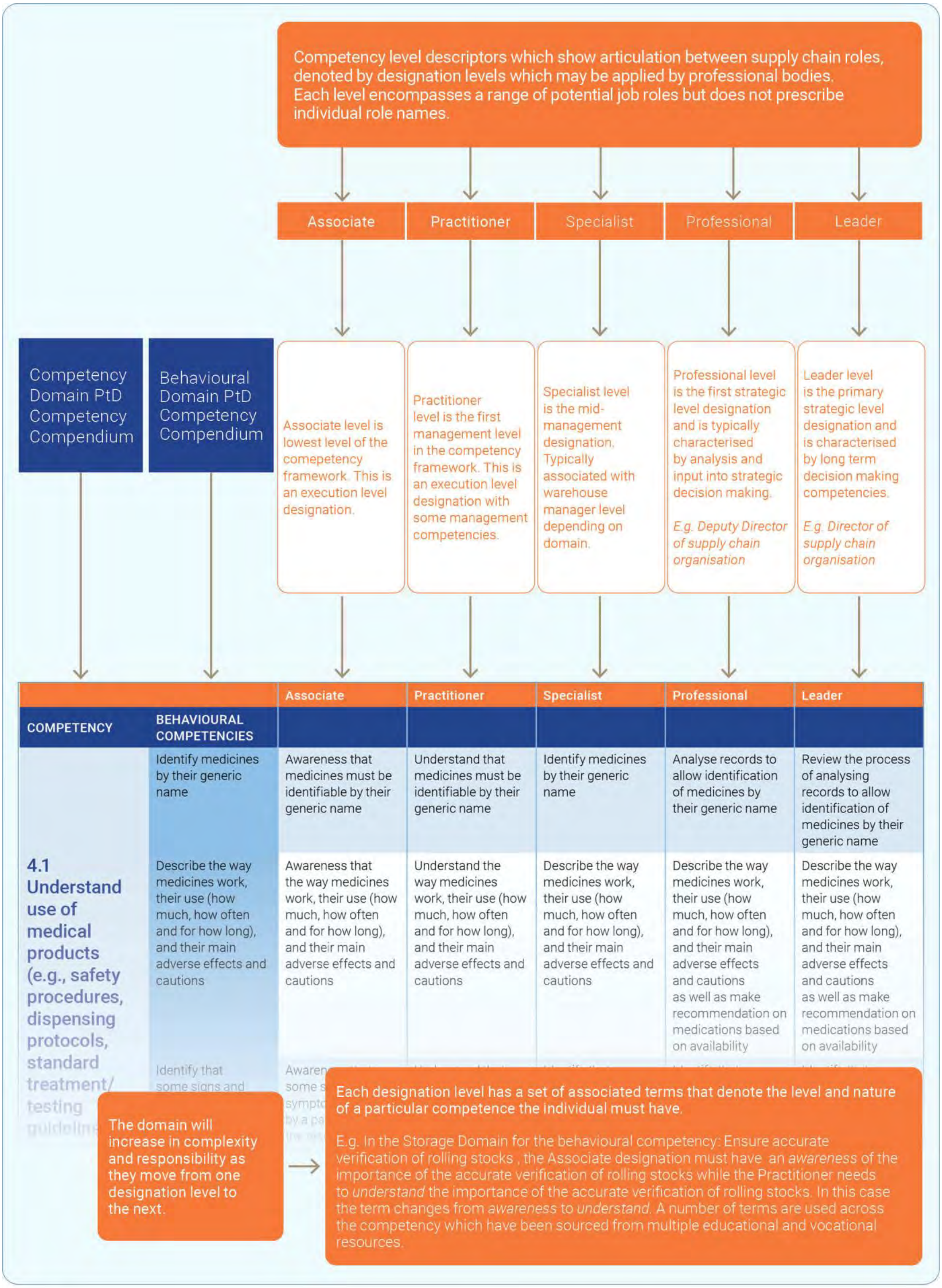

Figure 4:
Description of how the Library of Competencies & Designations for Health Supply Chains is constructed

Collection of Roles and Job Descriptions for Health Supply Chains (Demand)

The SCM Professionalisation Framework aims to professionalise supply chain management personnel and should have the effect of streamlining multiple aspects of supply chain management in a country or organisation. However, the country or organisation must have a need for personnel, in other words, demand must be created. The Collection of Roles and Job Descriptions for Health Supply Chains was created for this purpose: it outlines ways in which the Library of Competencies and Designations for Health Supply Chains can be organised operationally to create this demand.

There are several ways that this job description (JD) collection can assist you, whether you are involved in operations management or workforce development.

This collection was designed to be a reference guide to assist human resources and supply chain practitioners in the field in designing their organisational hierarchies and planning for human resource initiatives across the health supply chain.

Although the job description templates are provided as a point of reference for workforce development activities, they are best used with the Implementation Approach for Health Supply Chains. The Implementation Approach contains details pertaining to whole of labour market considerations, where these templates can be used as a component in creating a tailored workforce development plan.

Methodology

The Collection of Roles and Job Descriptions for Health Supply Chains (Demand) was created by PtD in 2018 and received input from multiple country examples to create the job descriptions: this was the first phase. In phase two these base job descriptions were then compared with a set of hierarchies that encompass the majority of roles to create the hierarchy found in this document. Based on aggregated descriptions of the job descriptions studied, the base job descriptions were assigned a primary domain and primary competencies.

It must be emphasised, however, that the example in this document is only one of many ways to combine the job descriptions.

Once the base job descriptions were created, the metrics and training needed to be defined. To define the training and metrics, the domains in which the job descriptions reside, and the primary competencies were used to align the job descriptions to various supply chain management frameworks. These frameworks define standard practices and metrics for the activities to which the job descriptions pertain. These alignments were then used to assign standard metrics to the job descriptions.

Finally, using the competencies assigned to each job description, the Mapping of Education for Health Supply Chains was used to provide education possibilities to teach the competencies required for an individual to perform each role.

While these are standardised job roles, the authors expect organisations will alter and merge the JDs according to their needs.

To make full use of the collection it is necessary to review the attributes of a JD, which are structured using the following table headers.

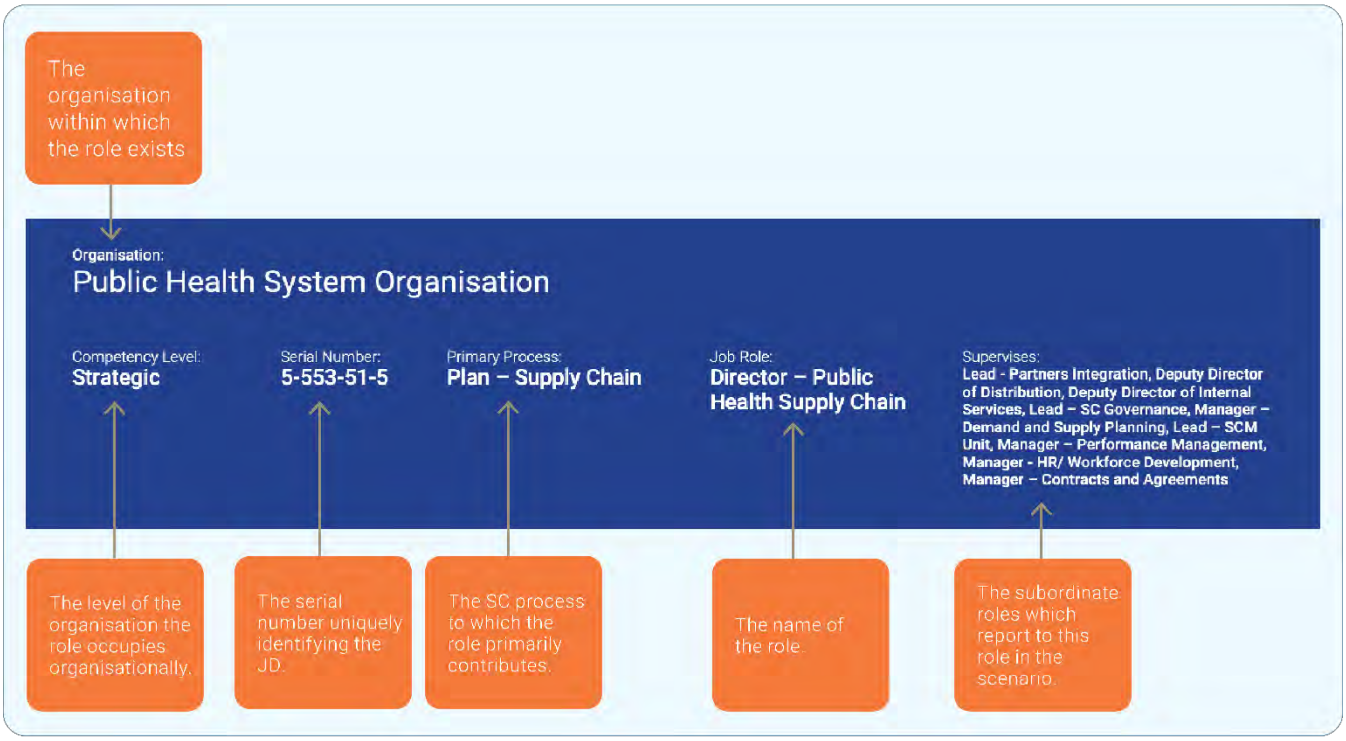

Figure 5:
Description of how the Collection of Roles and Job Descriptions for Health Supply Chains is constructed

The type of competency being referenced in this section.

A list of the competencies included in this section

5.1 Design and implement supply chain system and strategies

- Design a responsive, agile and efficient supply chain that has the ability to meet the changing needs of customers and deliver high-quality products with short lead times at low cost
- Establish the interrelationship of organisations, people, technology, activities, information, and resources involved in moving a product from supplier to customer
- Illustrate the management activities carried out in the course of running an organisation, including controlling, leading, monitoring, adjusting, organising and planning
- Strategically analyse the characteristics unique to public health, demonstrate awareness of the factors that could contribute to or hinder the delivery of logistics services
- Strategically analyse emerging concepts and principles in public health logistics; adapt current practices to incorporate new developments in the field
- Strategically analyse which type of inventory control system will be most effective for a particular programme or country

Top level competencies that describe the overall theme of the behavioral competencies included.

Behavioral Competencies, these are competencies the person should be able to exhibit as well as section of the work they may be responsible for.

A list of suggested Key Performance Indicators that are indicated for this role.

Key Performance Indicators:

- Assess Delivery Performance Cycle Time - Actual Time from assessed requirement to time delivered to end-user compared to committed Delivery Cycle Time as per Supply Chain strategy
- Assess Supplier Performance Cycle Time - Actual Supplier performance Cycle time versus Supplier commitment cycle time
- Inventory accuracy - Cycle count results
- Transportation lead time accuracy - Actual Transportation lead time versus Transportation promised lead time.
- Transport efficiency - Total cost of transport versus planned cost of transport by mode
- In-bound cost of acquisition - Actual total cost of acquisition versus Total Planned cost of acquisition including landed costs
- Performance Report - Actual versus Plan reports for all metrics
- Manage performance cycle time. Actual Supply Chain Performance required versus Actual. This is a roll up metric
- Finance secured and expenses managed in line with supporting distribution operations

Training:	Qualifications Available:	Certifications Available:
- Supply chain Management<br>- Report Writing<br>- Data Management<br>- Total Quality Management (TQM)<br>- Business Rule/Policy Management<br>- Controls and Compliance<br>- Monitoring and Evaluation	- Advanced Diploma in Quality Management<br>- Bachelor of Computer Technology	- SPSM3 TM (Enterprise-Wide Procurement Influence)<br>- Occupational Certificate: Health Information Manager<br>- APICS Certified supply chain professional certificate<br>- SPSM Level 4 (External Procurement Influence)<br>- Lean Six Sigma Certification in Finance black belt
Suggested occupational training that may be indicated for this role.	Available qualifications to satisfy the requirements of this job descriptions<br><br>** this list is not exhaustive and serves as guidance not prescription*	Available certifications to satisfy the requirements of this job descriptions<br><br>**this list is not exhaustive and serves as guidance not prescription*

Figure 5:
Description of how the Collection of Roles and Job Descriptions for Health Supply Chains is constructed (cont)

Level definitions

Organisational levels are defined in the collection according to the following hierarchy:

Strategic:

Applies strategic, systems thinking; directs and advises; manages change, and influences internal and external stakeholders.

Managerial:

Develops, improves and fulfils organisational and functional objectives; manages efficiency, quality and risk

Operational:

Provides and executes guidance on procedures and processes that are connected

Tactical

Executes the process and assists operational levels to perform their overarching duties.

Organisations

Public health system organisation:

This refers to the organisational structures found in public health systems established to deliver health services to country populations. The primary purpose of the organisations that collectively make up the public health system is to ensure access to quality care through programmes that target all populations. As access to quality medicines, supplies and equipment is a core component of care, the public health system also ensures that systems are in place to design, procure, deliver, and manage supply chains. This collection refers to public health system roles that are involved in ensuring functioning supply chain (SC) systems are in place while not necessarily executing the SC functions (these SC functions and roles are captured under the SC organisation type below). Meanwhile, this organisational type includes the service delivery levels of public health systems (such as hospital, clinics, and community health posts) that play a role as “customers” of supply chain systems.

The public health system is also typically tiered, where the decision making authority for health and SC programming is the purview of a high-level structure (a central body, or a state or regional structure under devolved health systems) and functions and roles are *cascaded* to lower, intermediate levels (such as regions in the case of centralised public systems, or provinces or districts.)

Supply chain (SC) organisation:

This refers to the entities involved in carrying out core SC functions to service the needs of the public health system with quality, timely and adequate medicines, supplies and equipment. The SC organisation may be publicly owned, parastatal or privately owned. The collection of roles for this organisational type is intended to reflect the full scope of functions and roles to ensure high performing, reliable supply chains. Typically, the SC organisation will include structures in different locations

(such as branches or hubs) to provide SC services closer to the population more cost-effectively.

Scenario hierarchy

The *Collection of Roles and Job Descriptions for Health Supply Chains* displays only one possible organisation of job roles in a country and the authors decided to use a version of a semi-autonomous supply chain organisation. Therefore, it is assumed that the primary supply chain domain knowledge is held by this organisation. Assuming this, the highest level of structure in the public health system would then perform a regulatory role, maintaining the SC organisation's compliance and synchronisation with national priorities.

What this means is that any supply chain role from the intermediate to the community levels will report to the supply chain organisation creating a flow of reporting and information that will align with the public health supply chain operating model. The hierarchy used for this scenario is displayed in Figure 6 below. The organisations in this document are also colour-coded according to the below diagram.

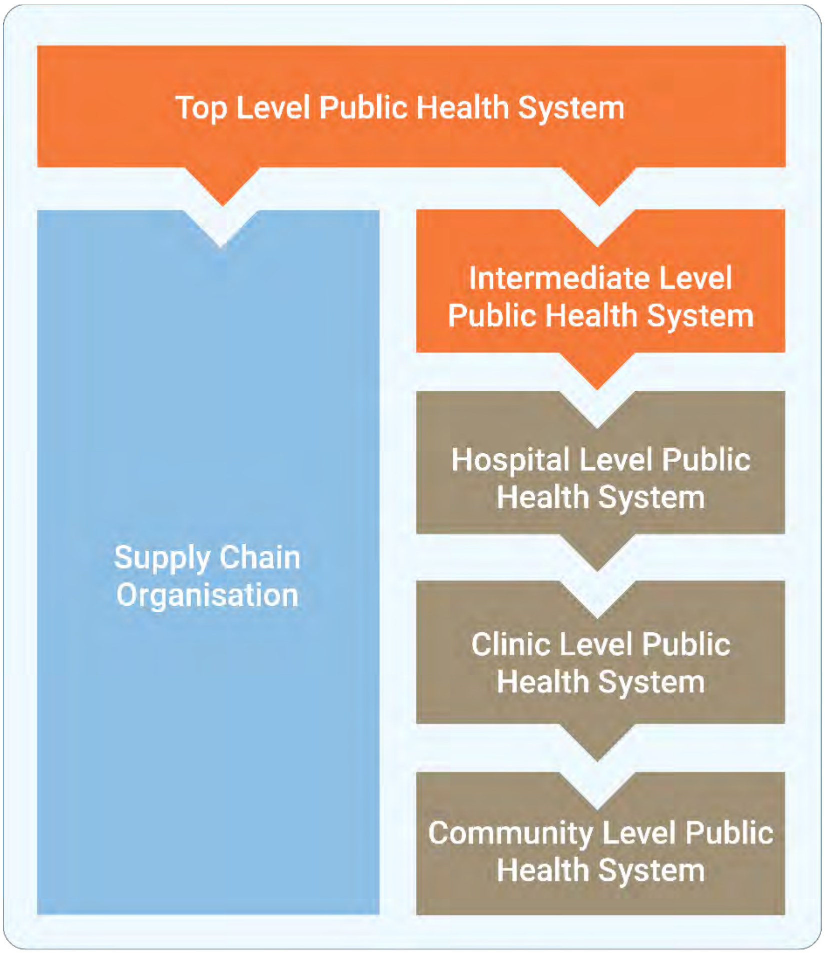

Figure 6:
Depiction of hierarchy used for generation of standardised job descriptions with corresponding colour codes

High level overview of job descriptions

	SC-focused Organisation	Public Health System Organisation	Public Health Organisation – Intermediate Level	Public Health Organisation – Hospital Service Delivery	Public Health Organisation – Clinic Service Delivery Level	Public Health Organisation – Community Service Delivery Level
**Deliver**						
Managerial	■		■	■		
Lead – Receiving and Inventory Management			■	■		
Manager – Customer and Sales	■					
Manager – Warehouse and Distribution	■					
Operational	■		■	■	■	
Head – Customer Order Processing/ Management	■					
Head – Transport and Delivery Management	■					
Head – Warehouse and Inventory Management	■					
Officer – Receiving and Inventory Management			■	■	■	
Tactical	■		■	■		
Assistant – Delivery	■					
Assistant – Inventory	■					
Assistant – Pick, Pack	■					
Assistant – Storage	■		■	■	■	
**Enable – Human Resources**						
Managerial	■		■			
Lead - HR/ Workforce Development			■			
Manager - HR/ Workforce Development	■					
Manager – HR/ Workforce Development	■					
Operational	■					
Officer - Human Resources Specialist	■					
Tactical	■					
Assistant - Human Resources Assistant	■					
**Enable – Contracts**						
Operational	■					
Officer – Contracts	■					
**Enable – Contracts & Agreements**						
Managerial	■	■				
Manager – Contracts and Agreements	■	■				
Operational	■					
Specialist – Contracts	■					
**Enable – Data & Information**						
Strategic	■					
Lead – Data Intelligence and Analytics	■					

Managerial						
Manager – Data and Use						
Manager – SC Data and Analytics						
Operational						
Officer – Data and Use						
Officer – Data Management						
Officer – SC Data Quality and Use						
**Enable – Performance**						
Strategic						
Lead – Planning and Performance						
Lead – Process Improvement and SC Adaptability						
Managerial						
Head - Cold Chain Management						
Lead – Health SC System Strengthening						
Manager - Contract and 3PLs						
**Enable – Procurement**						
Managerial						
Lead - Procurement						
Manager – Finance and Administration						
Operational						
Officer – Strategic Procurement						
Officer – Tender Management						
Officer– Procurement						
**Enable – Regulatory (& Individual) Compliance**						
Managerial						
Lead – Compliance / Internal Audit						
Lead – Compliance and Risk Management						
Lead - Partners Integration						
Operational						
Manager - Occupational Safety						
**Enable – Risk**						
Managerial						
Manager – QA/ QMS						
Operational						
Officer – QA/ QC						
**Enable – SC Networking**						
Strategic						
Lead – Network Planning						
Operational						
Officer – Network Analyst						
**Enable – SC Performance**						
Strategic						
Lead – SCM Unit						

Managerial						
Manager – Performance Management						
Operational						
Officer – Performance Monitoring						
**Plan – Deliver**						
Managerial						
Manager - Logistics Planning						
Operational						
Officer - Logistics Planning						
**Plan – Procurement**						
Managerial						
Head – Demand and Supply Planning						
Manager – Demand and Supply Planning						
Manager – Procurement						
Operational						
Officer – Demand and Supply Planning						
Officer – Inventory and Demand Planning						
Officer – Product Selection Use						
Officer – Supply Planning/ Supplier Management						
**Plan – Supply Chain**						
Strategic						
Deputy Director of Distribution						
Deputy Director of Internal Services						
Director – Public Health Supply Chain						
Lead – SC Governance						
Operational						
Lead - Demand and Supply Planning						
**Procure**						
Managerial						
Lead – Procurement and Supply						
Manager – Programmes (Donations) and Global Partnerships						
Operational						
Officer – Import and Clearance						
Officer – Procurement and Supply						
Officer – Programmes (Donations) Procurement						
Officer – Receiving						
Tactical						
Assistant – Receiving						
Clerk – Receiving and Storage						
Clerk – Supply						
**Return**						
Managerial						
Lead – Returns and Disposal						

Operational						
Lead - Returns Management						
Lead – Waste and Returns Management						

Mapping of Education for Health Supply Chains

To improve the supply of SCM professionals there needs to be significant support for it in academia as well as from vocational training providers, certification, and professional bodies in-country. Consequently, PtD has developed a list of available qualifications and certifications that can assist in acquiring the skills needed to be deemed a professional in the health supply chain sector.

This list is by no means exhaustive nor is it prescriptive, it is only a reflection of the courses readily available to the authors at the time of writing, which directly correlate with the skills and competencies specified in the competency framework and job descriptions. It includes more than 250 courses from various providers around the world.

In the Education Framework document, the user will find a similar structure to the competency framework; the difference is that there are no competency descriptors but rather in their place, a list of courses that have exhibited content for those competency descriptors.

The Education Framework is designed to give the user a broad idea of the types of education available to fill the skills gaps found in the country, but the authors recognise that each country may have its own regulations and prerequisites.

Methodology

In order to develop the Mapping of Education for Health Supply Chains a model needed to be chosen. The authors had a firm grasp of and easy access to one of the leading education frameworks in Africa, namely the South African Qualifications Authority (SAQA). SAQA contains more than 14,000 qualifications for evaluation spanning various subjects, including but not limited to public healthcare.

Thus, because this dataset was non-specific, the first step was to filter out irrelevant courses. The competency framework was used as a first-pass filter: anything that did not directly relate to the competency framework was removed, leaving 436 courses that related to the seven domains of the competency framework.

The competency framework comprises more than 3,000 competencies across the levels and behavioural competencies. To speed up the process a set of natural language processing (NLP) algorithms was developed and deployed against the dataset.

The NLP algorithms were used to compare behavioural competencies with the course description, outcomes and overall information contained in the SAQA course records. Where there was significant overlap, the course was said to match the competency. Only the top 5% of matches were kept as candidates for the Mapping of Education for Health Supply Chains. Once the first pass with SAQA was completed, certificate courses were added to this list and assigned in a similar method. This had two effects: one was to expand the dataset and the other was to partially validate the model as these certificate courses are more focussed than the degrees from SAQA and easier to validate. The results of this validation were that less than 2% of allocations made for the certificate programme were judged to be erroneous.

At this point a candidate list was created including SAQA and various certificate courses. These courses were compared to the competencies that matched them and removed where inaccuracies were observed to be less than 5%, validating the model and finalising the Mapping of Education for Health Supply Chains. The full list of course sources can be found below.

Courses are listed from:

› Coursera

› MIT

› edukazi.com

› South African Qualifications Authority
(All registered qualifications in South Africa)

› Empower

› CIPS

› CILT

› I+ Solutions

› ASCM/APICS

› SAPICS

› Next Level Purchasing Association

If you are a training provider your courses may be included on this list by contacting PtD and supplying the requisite information and added to future revisions of this document.

An example of how to navigate this framework is displayed below.

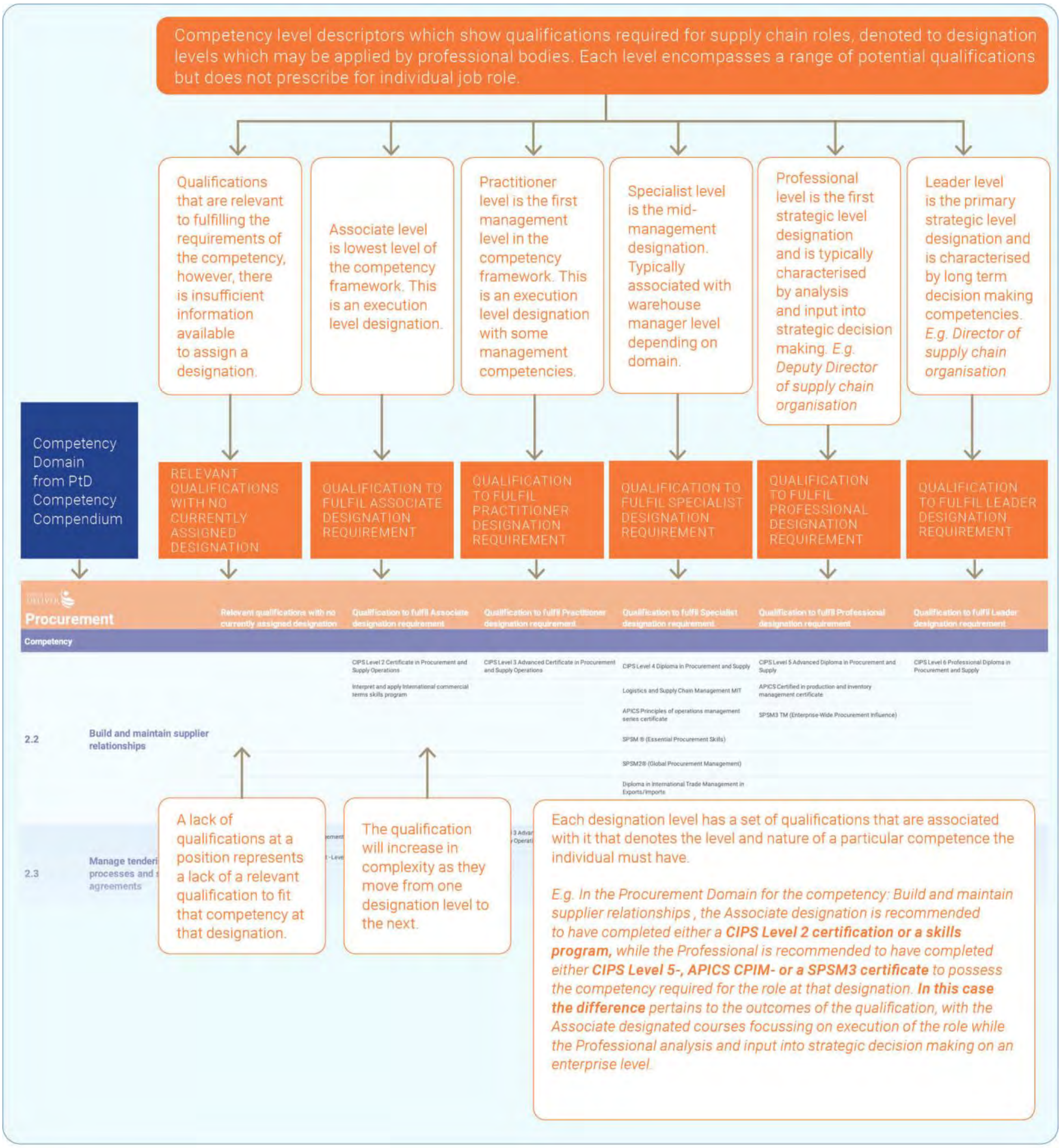

Figure 7:
Description of how the Mapping of Education for Health Supply Chains is constructed

A note on GHSC-PSM

GHSC-PSM recognises that without a strong, skilled workforce at the national and local levels, system-based and technological improvements won't have their intended effect. Developing a well-performing, motivated workforce requires strengthening the organisational systems, processes and environment in which supply chain workers perform their duties.

The GHSC-PSM vision is to foster self-sufficient organisations with institutionalised systems. This in turn will help ensure high performance from a professionalised and consumer-centred workforce for effective and efficient delivery of health commodities through to the last mile.

The project goes beyond basic capacity building to look at long-term solutions to organisational and people development, considering the development of human resources systems as an investment. We do this by providing technical support to continuously improve the systems, processes and factors affecting an organisation's ability to plan for, manage and support professionalised national cadres of supply chain professionals.

A note on People that Deliver

PtD advocates interventions that improve the demand and supply of qualified health supply chain professionals in organisations, which in turn strengthens the individual practitioners within those organisations. Since 2011, PtD has contributed significantly to the human resources body of knowledge available for health supply chain practitioners. As custodians of the Competency Compendium for Health Supply Chain Management, PtD was an obvious partner in developing a SCM professionalisation framework.

A note on SAPICS

SAPICS has taken the step to professionalise SCM in South Africa by assuming the role of the professional body for supply chain management. Having served the profession for 50 years, it is well positioned to provide the services that will see it elevate supply chain management as a profession as well as the practices and people within it. It also assists in fostering relationships with governments to assist in addressing strategic imperatives for economic transformation in South Africa and the continent of Africa more broadly. SAPICS awards professional designations based on technical supply chain competencies.

Public Health System Organisation

Strategic
Director – Public Health Supply Chain

Organisation:

Public Health System Organisation

Competency Level:	Serial Number:	Primary Process:	Job Role:	Supervises:
Strategic	5-553-51-5	Plan – Supply Chain	Director – Public Health Supply Chain	Lead - Partners Integration, Deputy Director of Distribution, Deputy Director of Internal Services, Lead – SC Governance, Manager – Demand and Supply Planning, Lead – SCM Unit, Manager – Performance Management, Manager - HR/ Workforce Development, Manager – Contracts and Agreements

Contributions	Articulates the Supply Chain Strategy to the Public Health System Organisation. Facilitates the execution of the Strategy, is accountable for the performance of the Supply Chain.
Personal/Management Competency(ies):	**5.1 Design and implement supply chain system and strategies**<br>**5.5 Manage and plan projects**<br>**5.5.2 Execute strategic decision making**<br>**5.5.3 Manage partnerships**<br>**6.5 Prove leadership abilities**<br>**6.5.1 Demonstrate resilience and ability to manage stress**<br>**6.6 Abide by rules / laws / legislation**

5.1 Design and implement supply chain system and strategies

- » Design a responsive, agile and efficient supply chain that has the ability to meet the changing needs of customers and deliver high-quality products with short lead times at low cost
- » Establish the interrelationship of organisations, people, technology, activities, information, and resources involved in moving a product from supplier to customer
- » Illustrate the management activities carried out in the course of running an organisation, including controlling, leading, monitoring, adjusting, organising and planning

- Strategically analyse the characteristics unique to public health, demonstrate awareness of the factors that could contribute to or hinder the delivery of logistics services
- Strategically analyse emerging concepts and principles in public health logistics; adapt current practices to incorporate new developments in the field
- Strategically analyse which type of inventory control system will be most effective for a particular programme or country
- Demonstrate in-depth knowledge the difference between continuous and period review systems of inventory control
- Develop and implement a formal logistics strategy and plan

5.5 Manage and plan projects

5.5.2 Execute strategic decision making

- Demonstrate making decisions regarding one's own workload and area of responsibility
- Illustrate the process of prioritizing competing tasks and perform them quickly and efficiently according to their importance; finding new ways of organizing or planning work to accomplish it more efficiently
- Implement the proper time and space for consultation around decisions being made.
- Implement the process of considering the input of resources including staff needed
- Develop the process of checking assumptions against facts
- Strategically analyse the key issues in a complex situation and come to the heart of the problem quickly
- Strategically analyse relevant information before making decisions
- Make strategic decisions in the interest of the organisation's goals
- Demonstrate how to analyse, evaluate and execute tough decisions when necessary
- Strategically analyse the impact of decisions on others' work or team goals
- Develop the process of communicating decisions and ensure they are incorporated into policies and processes
- Undertake inclusive and consultative strategic planning to establish strategic objectives
- Strategically analyse and implement emerging trends and practices
- Outline the macro and long-term consequences of decisions

5.5.3 Manage partnerships	» Implement the concepts of partnership working<br>» Develop the process of supporting implementation of partnership programmes<br>» Experiment with and trial new ideas with partners<br>» Build partnerships to deliver programme and increase impact<br>» Promote innovation and creativity in partnership work<br>» Develop the process of communicating key information with partner members<br>» Develop the process of involving and valuing partners in all aspects of programming<br>» Strategically analyse problems with supply chain relationships<br>» Illustrate the process of communicating effectively with nurses, doctors and other members of the healthcare team<br>» Develop the process of responding to consumer complaints or comments about services and/or advice received<br>» Illustrate collaboration and consultation across the sector and encourage the team to work with others to gain market insight<br>» Develop the process of sharing input from other key actors in the humanitarian sector and develop relationships with a cross-section of actors<br>» Direct the process of meeting the reporting requirements of vertical programmes<br>» Illustrate the structure of the health system at a national level and explain this to others<br>» Illustrate of the roles of other members of the healthcare team (including with consumers) in a way that engenders understanding and confidence in the team and its members<br>» Direct the structure of the organisation, environment and/or service in which they work<br>» Illustrate how vertical programmes work within the health system<br>» Establish the communication network to achieve work outcomes<br>» Demonstrate in-depth knowledge of the structure of the health system at the provincial/regional level and explain this to others<br>» Demonstrate in-depth knowledge of the roles and functions of government agencies in regulating and supporting supply chain organisations<br>» Demonstrate in-depth knowledge of change management (e.g. PtD Theory of Change)
6.5 Prove leadership abilities	» Strategically analyse input to meetings and programme development<br>» Analyse future trends and issues, and assist the organisation in meeting the challenges<br>» Develop a process to contribute to a collaborative working environment<br>» Demonstrate a drive for change and improvement develops opportunities for the organisation and sector<br>» Demonstrate channelling energy and ideas toward resolving issues

	» Demonstrate the practise of communicating with influence<br>» Demonstrate showing courage to take an unpopular stance when needed<br>» Demonstrate steering and implementing change organisationally<br>» Establish the culture of anticipating and resolving conflict<br>» Establish a culture of showing initiative in working methods<br>» Exemplify personal drive and integrity<br>» Demonstrate serving as a role model for others to follow<br>» Demonstrate leadership and practice management skills, initiative and efficiency<br>» Demonstrate inspiring others through professional excellence, innovation and communication<br>» Illustrate situations in which a change in leadership style would be warranted (e.g., directive versus consultative style in the event of a crisis)
6.5.1 Demonstrate resilience and ability to manage stress	» Demonstrate identifying symptoms of stress and take steps to reduce stress<br>» Demonstrate seeing the bigger picture and help others to<br>» Demonstrate recovering quickly from setbacks<br>» Demonstrate drawing on previous experience and support mechanisms to reduce the impact of stress on self and others<br>» Create a working environment that aims to minimise pressure and stress<br>» Demonstrate coping well under pressure, particularly in difficult environments<br>» Demonstrate acting as a role model for others and display courage under difficult circumstances<br>» Develop a methodology for recognising the limitations of staff and taking action to limit their exposure to harm when needed<br>» Implement the use of personal support mechanisms<br>» Demonstrate helping others identify personal support mechanisms<br>» Direct organisational policy to support self-care in agencies
6.6 Abide by rules / laws / legislation	» Ensure compliance with the legislation that covers the practice of public health supply chains and healthcare, and describe its purpose<br>» Implement requirements of professional codes, guidelines and standards adopted as part of the legislative framework<br>» Develop methodology to keep up to date with changes in legal instruments, as informed by national-level managers<br>» Ensure compliance with international regulations in decision making for the distribution system, including customs regulations on import and export requirements (e.g., trade tariffs and duties on imported goods) and security regulations (e.g., 2007 SAFE Ports Act, NAFTA or European Union trade agreements)<br>» Demonstrate in-depth knowledge of regulatory affairs and the key aspects of pharmaceutical registration and legislation

	» Demonstrate in-depth knowledge of the principles of business economics and intellectual property rights, including patent interpretation<br>» Demonstrate in-depth knowledge of applicable procurement law and the practical effects of a contract's terms and conditions<br>» Strategically analyse current industry and government regulations governing sustainability<br>» Demonstrate in-depth knowledge of key hazardous materials handling laws<br>» Implement policies and procedures relating to workplace safety that are consistent with agreed or recognised standards<br>» Implement professional standards and conventions, as well as workplace policies and procedures, for preparing pharmaceutical products<br>» Develop all standard operating procedures<br>» Implement and direct the process of complying with data privacy and data protection laws
Basal Technology Competency(ies):	7.1 Data Science<br>7.4 Temperature and monitoring<br>7.5 Planning systems<br>7.6 ERP includes function of LMIS<br>7.12 Basic Office Skills<br>7.13 Have a command of technology
7.1 Data Science	» Strategically analyse end to end MIS technology components that are inherent in advanced MIS systems and explore how these may be relevant in different supply chain contexts<br>» Implement emerging technological approaches to data visualization and analysis<br>» Strategically analyse how data science can be applied within supply chain<br>» Demonstrate the ability to interact, provide guidance for data (dashboard) consolidation in various levels and use the tech tools<br>» Support movement of data into electronic form at lowest/remote SC level<br>» Demonstrate the ability to work with relational database
7.4 Temperature and monitoring	» Strategically analyse the current technology approaches for Temperature monitoring and sensors<br>» Strategically analyse what is required to implement this technology in a country context<br>» Strategically analyse infrastructure requirement for heat-sensitive items at peripheral 'lower level' of SC depending on volume and nature of items to handle;<br>» Implement regular preventive maintenance and calibration of cold-chain equipment with biomedical engineers or logisticians<br>» Implement the policy for temperature monitoring tools during transportation and storage

» Strategically analyse the steps involved in health logistic management cycle: selection-order-delivery-reception-storage-distribution-inventory

» Demonstrate in-depth knowledge of hardware qualification and system validation of temperature monitoring

» Implement and direct mass data management of temperature monitoring

7.5 Planning systems

» Implement blockchain and understand the building blocks for planning systems

» Analyse planning systems as a value-add capability for relevant global health use cases

7.6 ERP includes function of LMIS

» Implement a Master Data Management System

» Demonstrate an in-depth understanding of the critical components of an ERP system

» Demonstrate an in-depth understanding of the meaning of common document types found within an ERP/LMIS

» Demonstrate an in-depth understanding of the logic of data visibility within an ERP

» Demonstrate in-depth knowledge on the importance of data integrity, record management and information security

7.12 Basic Office Skills

» Demonstrate a good understanding of common presentation authoring packages

» Demonstrate a good understanding of common spreadsheet authoring packages

» Demonstrate a good understanding of common document authoring packages

» Demonstrate a good understanding of common email authoring packages

» Demonstrate a good understanding of common instant messaging packages

» Demonstrate a good understanding of common video conferencing authoring packages

7.13 Have a command of technology

» Implement the use of technology suitable to the job held

» Review new developments and technologies in the sector

» Analyse new technologies and recognise potential benefits for the sector

» Analyse the resources and support that are provided across the organisation to enable colleagues to make the best use of available technology

» Develop the organisations' strategy for technology use

» Strategically analyse how to minimise environmental damage through technology use

» Implement the use of technology to maximise effectiveness and efficiency

	» Implement the use of field-based technology (e.g., radio, general packet radio service (GPRS), satellite phone)
Advanced Technology Competency(ies):	7.2 Blockchain<br>7.3 Unmanned Aerial Vehicles (UAVs)<br>7.7 Automation<br>7.8 Artificial Intelligence<br>7.9 Additive Manufacturing<br>7.10 Internet of things<br>7.11 Cloud Computing<br>7.14 eProcurement
7.2 Blockchain	» Strategically analyse blockchain as a value-add capability for relevant global health use cases
7.3 Unmanned Aerial Vehicles (UAVs)	» Demonstrate in-depth knowledge of the current UAV landscape, their application and the enabling environment required to support country implementation<br>» Strategically analyse UAVs as a value-add capability for relevant global health use cases<br>» Demonstrate an in-depth understanding of the laws/ regulations around the use of UAV's
7.7 Automation	» Strategically analyse automation as a value-add capability for relevant global health use cases
7.8 Artificial Intelligence	» Strategically analyse artificial intelligence as a value-add capability for relevant global health use cases
7.9 Additive Manufacturing	» Strategically analyse additive manufacturing as a value-add capability for relevant global health use cases
7.10 Internet of things (IoT)	» Strategically analyse the opportunity of IoT used in conjunction with AI<br>» Strategically analyse the internet of things as a value-add capability for relevant global health use cases
7.11 Cloud Computing	» Strategically analyse cloud computing as a value-add capability for relevant global health use cases

7.14 eProcurement	» Develop a methodology on how to transact with an eProcurement system where necessary<br>» Strategically analyse eProcurement as a value-add capability for relevant global health use cases<br>» Strategically analyse the benefits and limitations of eProcurement to best utilise the system

Key Performance Indicators:

- Manage performance cycle time
- Performance Report - Actual versus Plan reports for all metrics
- Transport efficiency - Total cost/unit of transport versus planned cost/unit of transport by mode
- In-bound cost of acquisition - Actual total cost /unit of acquisition versus Total Planned cost/unit of acquisition including landed costs
- Cost per Unit delivered (includes all costs, including product costs)
- Months stock on hand
- Fill rate percentage (% of goods supplied versus ordered, at the Unit level)

Training:	Qualifications Available:	Certifications Available:
» SC Optimization<br>» Scenario Planning<br>» Integrated Business/ SC Planning<br>» Supply chain Risk<br>» Executive training	» Master of Philosophy in Health Innovation<br>» Doctor of Philosophy in Health Sciences<br>» Master of Pharmaceutical Sciences	» APICS Certified supply chain professional certificate<br>» CIPS Level 6 Professional Diploma in Procurement and Supply

Lead – SC Governance

Organisation:

Public Health System Organisation

Competency Level:	Serial Number:	Primary Process:	Job Role:	Supervises:
Strategic	5-532-64-4	Plan – Supply Chain	Lead – SC Governance	Lead – Compliance / Internal Audit, Lead – Compliance and Risk Management

Contributions	» Ensures that the Governance Enabling process is current and appropriate to the execution of the Public Health System Organisation. Supports the Public Health Care Supply Chain Strategy by participating in the Strengthening of the Governance process
Personal/Management Competency(ies):	» 5.3 Implement risk management and monitoring and evaluation activities for the supply chain<br>» 5.3.2 Implement risk management activities<br>» 6.1 Demonstrate generic skills (e.g., literacy, numeracy, technology)<br>» 6.1.1 Exhibit high understanding of literacy and numeracy<br>» 6.2 Demonstrate strong communication skills<br>» 6.2.1 Practice cultural awareness<br>» 6.4 Exhibit professional and ethical values<br>» 6.4.1 Demonstrate integrity<br>» 6.4.2 Engage in continuous professional development
5.3 Implement risk management and monitoring and evaluation activities for the supply chain	
5.3.2 Implement risk management activities	» Determine risks affecting supply, transformation, delivery and customer demand<br>» Determine what constitutes an unsafe working conditions and how to take prompt, corrective action; stay alert to and take preventive action against hazards and threats; recommend measures to protect employees from hazardous working conditions; follow protocol for reporting safety violations; and investigate accidents to determine how to prevent them in the future<br>» Demonstrate training programmes/drills in which personnel participate (e.g., fire and armed hold-up drills, safe manual handling practices, use of mandatory safety clothing)<br>» Analyse systems of review (e.g., audit) to check compliance with policies and procedures intended to maintain workplace safety (e.g., manual handling techniques, correct use of protective clothing)<br>» Determine the process of ensuring equipment and tools are operating to prescribed standards

	» Determine key security systems for the workplace (e.g., for cash, narcotics and other controlled substances, investigational drugs, consumer records, entry and exit points) and levels of access and/or authority applicable to each<br>» Demonstrate the prevention strategies adopted (e.g., protocols, security barriers, fixed or personal duress alarms)<br>» Evaluate documented critical incidents<br>» Engage in contingency planning and managing operational continuity<br>» Ensure that there is an understanding of what a safe working environment is and engender a shared commitment from all personnel to creating one<br>» Demonstrate knowledge of relevant safety and health laws and regulations; comply with safety codes, standards and guidelines; adhere to worksite safety programmes; understand emergency response plans when they exist<br>» Demonstrate knowledge of how to handle hazardous commodities and first aid procedures
6.1 Demonstrate generic skills (e.g., literacy, numeracy, technology)	
6.1.1 Exhibit high understanding of literacy and numeracy	» Encourage speaking clearly and confidently, organizing information in a logical manner, and considering voice tone and pace<br>» Demonstrate responding to verbal messages and other cues (active listening)<br>» Demonstrate expressing ideas and opinions clearly in written and verbal form<br>» Demonstrate communicating information accurately, concisely and confidently in writing and verbally<br>» Demonstrate a level of mathematics suitable to the job held<br>» Demonstrate knowledge of visual presentation techniques, including charting, histograms and flow sheets<br>» Demonstrate reading and comprehending at a level necessary to properly complete duties of the position<br>» Demonstrate the process of interpreting written information in documents, such as reports, SOPs, LMIS forms, graphs, calendars, schedules, notices and directions<br>» Demonstrate paying attention to detail and identifying the main ideas, detecting inconsistencies and identifying missing information in documents<br>» Demonstrate computer skills — such as using Word, Excel, PowerPoint and the internet — suitable to the job level<br>» Demonstrate the process of analysing and validating KPIs<br>» Participate in the evaluation of activities that act as standards against which the KPIs are measured<br>» Demonstrate the process of performing critical self-review on work before submission

6.2 Demonstrate strong communication skills

- Demonstrate being truthful and trustworthy, and supplying accurate information at all times
- Demonstrate working as part of a workplace team
- Determine vocabulary and communication style and form for both written and verbal communication that is appropriate for the situation, audience and material being communicated (e.g., avoid unnecessary jargon, clearly explain medical and SCM terminology)
- Demonstrate open communication within constraints of confidentiality
- Analyse communications about logistics
- Demonstrate participation in meetings, expressing one's opinions, being aware of others' needs, and being appropriately assertive when required
- Demonstrate perspective into the point of view of others, understanding their needs and goals
- Demonstrate the practise of building trust, rapport and credibility with others
- Determine the communication needs and concerns of others and respond to them
- Demonstrate avoiding conflict between work and personal interests
- Demonstrate the practice of meaningful two-way communication
- Demonstrate influence on others by persuasively presenting thoughts and ideas
- Demonstrate assertiveness skills to deal with unreasonable requests and/or refusals that would compromise practice or consumer care
- Demonstrate a positive, supportive and appreciative attitude
- Demonstrate the ability to balance the organisation's needs and the customer's needs
- Demonstrate self-control by maintaining composure and keeping emotions in check, even in difficult situations; deal calmly and effectively with stressful situations
- Demonstrate the expressing of opinions and providing information in written and/or verbal form in a manner that does not elicit concern, anger or other adverse response
- Demonstrate the use of a systematic process for following up that demonstrates written reports have been received and understood
- Evaluate the means by which responses to input to the work environment are monitored
- Seek practical ways to overcome barriers to communication
- Ensure language representation (e.g., interpreters) at meetings when appropriate
- Demonstrate tackling difficult situations and resolving disputes between staff
- Demonstrate giving both positive and negative feedback sensitively
- Demonstrate maintaining and improving communication
- Demonstrate the process of giving and requesting feedback frequently
- Demonstrate the ability to communicate effectively with shop stewards and union representatives

6.2.1 Practice cultural awareness

- Demonstrate cultural awareness and sensitivity, treating all people with fairness, respect and dignity
- Demonstrate anti-discriminatory practices in the organisation, including HR and disciplinary procedures
- Demonstrate cultural sensitivity, equality and fairness at all levels of the organisation
- Demonstrate challenging discriminatory behaviour directly and sensitively
- Demonstrate acting in a non-discriminatory way toward individuals and groups
- Demonstrate the process of integrating cultural awareness in learning and development approaches
- Demonstrate avoiding stereotypical responses by examining one's own behaviour and bias
- Demonstrate managing cultural diversity in teams and make the most of differences
- Demonstrate the use of strategies and/or resources for communicating effectively with people from different cultural backgrounds
- Supply information relating to values, beliefs and cultural backgrounds of consumers that may influence the way professional services are provided

6.4 Exhibit professional and ethical values

6.4.1 Demonstrate integrity

- Demonstrate accepting responsibility for work tasks and performance
- Demonstrate working within a framework of clearly understood humanitarian values and ethics
- Demonstrate standing by decisions and holding others accountable when necessary
- Demonstrate not abusing one's own power or position
- Demonstrate the process of managing team members to ensure they do not abuse their power or position
- Determine when individuals or the organisation is straying from organisation goals, and challenge them to uphold ethics
- Demonstrate resisting undue political pressure in decision making
- Demonstrate supporting staff in maintaining ethical stances
- Demonstrate making time in team for ethical inquiry and reflection
- Demonstrate showing consistency between expressed principles and behaviour
- Ensure that principles, values and ethics are embedded in policy
- Demonstrate acting without consideration of personal gain
- Demonstrate promoting transparency in decision making structures and processes
- Ensure programmes are acting with integrity, and recognise the impact of not doing so
- Demonstrate recognising one's own limitations and act upon them
- Demonstrate all standard operating procedures

» Demonstrate working in a safe and legal way
» Demonstrate respect, dignity and consideration for consumers
» Determine the impact of a no-blame culture on reporting and preventing recurrence of incidents
» Evaluate requests of colleagues that might be regarded as unreasonable
» Demonstrate communicating (verbally and by example) expectations of the desired standards and approaches to be adopted
» Determine roles and responsibilities in terms of the position statement/duty statement of the position held
» Demonstrate punctuality
» Demonstrate applying professional care and expertise to deliver high quality of services
» Demonstrate care and attention to detail in undertaking work activities
» Determine appropriate attire and presentation for the role and situation
» Demonstrate recognising and taking responsibility for emotions
» Demonstrate an environment in which others can talk and act without fear of repercussion
» Demonstrate keeping commitments and not letting people down
» Demonstrate being trusting and cooperative when working alongside others
» Demonstrate taking on various responsibilities within the department as the need arises

6.4.2 Engage in continuous professional development

» Demonstrate seeking out opportunities to learn, and integrating new knowledge and skills into work
» Determine gaps in knowledge and skills for completing specific tasks, and develop a plan to acquire them
» Demonstrate listening to and inviting feedback from others on one's own performance
» Demonstrate using lifelong learning (continuous striving to gain knowledge and maintain competence) in the context of career development and the professional's role in delivering healthcare services
» Analyse organisational systems for capturing learning and ensuring lessons learned
» Employ reflective learning within the team, in which lessons are captured and integrated into future projects
» Promote continuous learning as an integral part of organisational performance
» Demonstrate being open to new ideas and different perspectives
» Demonstrate keeping up to date in the place of work with input from supervisors
» Demonstrate documenting continual professional development activities
» Demonstrate the use of multiple techniques to acquire new knowledge and skills; process and retain information; and identify when it is necessary to acquire new knowledge and skills.
» Convene professional organisations and/or committees

- Determine and/or demonstrate quality improvement and/or quality assurance activities participated in
- Demonstrate reflecting on performance and seeking what needs improvement
- Demonstrate the process of engaging in personal career development, identifying occupational interests, strengths, options and opportunities
- Demonstrate contributing to others' professional development
- Demonstrate sharing experiences and learning internally and externally
- Demonstrate seeking out challenging projects outside of core experience and achieve solid results

Basal Technology Competency(ies):	» 7.5 Planning systems<br>» 7.6 ERP includes function of LMIS<br>» 7.12 Basic Office Skills<br>» 7.13 Have a command of technology
7.5 Planning systems	» Analyse blockchain and understand the building blocks for planning systems<br>» Evaluate planning systems as a value-add capability for relevant global health use cases
7.6 ERP includes function of LMIS	» Evaluate a Master Data Management System<br>» Demonstrate an understanding of the critical components of an ERP system<br>» Demonstrate an understanding of the meaning of common document types found within an ERP/LMIS<br>» Demonstrate an understanding of the logic of data visibility within an ERP<br>» Demonstrate the importance of data integrity, record management and information security
7.12 Basic Office Skills	» Demonstrate a good understanding of common presentation authoring packages<br>» Demonstrate a good understanding of common spreadsheet authoring packages<br>» Demonstrate a good understanding of common document authoring packages<br>» Demonstrate a good understanding of common email authoring packages<br>» Demonstrate a good understanding of common instant messaging packages<br>» Demonstrate a good understanding of common video conferencing authoring packages
7.13 Have a command of technology	» Demonstrate the use of technology suitable to the job held<br>» Monitor new developments and technologies in the sector

	» Experiment with new technologies and recognising potential benefits for the sector<br>» Ensure that resources and support are provided across the organisation to enable colleagues to make the best use of available technology<br>» Determine what the organisations' strategy is for technology use<br>» Determine how to minimise environmental damage through technology use<br>» Analyse the use of technology to maximise effectiveness and efficiency<br>» Analyse the use of field-based technology (e.g., radio, general packet radio service (GPRS), satellite phone)
Advanced Technology Competency(ies):	7.2 Blockchain<br>7.7 Automation<br>7.11 Cloud Computing
7.2 Blockchain	» Evaluate blockchain as a value-add capability for relevant global health use cases
7.7 Automation	» Evaluate automation as a value-add capability for relevant global health use cases
7.11 Cloud Computing	» Evaluate cloud computing as a value-add capability for relevant global health use cases

Key Performance Indicators:

» Assess Delivery Performance Cycle Time - Actual Time from assessed requirement to time delivered to end-user compared to committed Delivery Cycle Time as per Supply Chain strategy

» Assess Supplier Performance Cycle Time - Actual Supplier performance Cycle time versus Supplier commitment cycle time

» Inventory accuracy - Cycle count results

» Transportation lead time accuracy - Actual Transportation lead time versus Transportation promised lead time.

» Transport efficiency - Total cost of transport versus planned cost of transport by mode

» In-bound cost of acquisition - Actual total cost of acquisition versus Total Planned cost of acquisition including landed costs

» Manage performance cycle time

» Performance Report - Actual versus Plan reports for all metrics

» Order fill rate (quantity and item lines fulfilled according to the DP requisitions)

» Stock according to plan/ to show overstock or understock

» Availability of health commodities/Stock out

» Expiration of health products (total value of expired products/Value of inventory + value of expiries)

» Inventory accuracy for warehouse management system vs physical count

Training:	Qualifications Available:	Certifications Available:

» Risk and exception management<br>» Process Auditing	» Bachelor of Business Administration<br>» Bachelor of Supply Chain Management	» SPSM3 TM (Enterprise-Wide Procurement Influence)<br>» Occupational Certificate: Health Information Manager<br>» APICS Certified supply chain professional certificate

Lead – SCM Unit

Organisation:

Public Health System Organisation

Competency Level:	Serial Number:	Primary Process:	Job Role:	Supervises:
Strategic	3-31-34-4	Enable – SC Performance	Lead – SCM Unit	Lead – Receiving and Inventory Management, Manager – Performance Management, Manager – SC Data and Analytics, Manager – Programmes (Donations) and Global Partnerships, Lead – Health SC System Strengthening

Contributions	Creates and Maintains all the Enablers to perform evaluation of SC Organisation and structure, SOP, LMIS etc by continuously monitoring impacts on SC objectives, performance and KPIs required as prescribed in Supply Chain System Organisation Operating Model.
Technical Competency(ies):	3.1 Undertake storage, warehousing and inventory management<br>3.1.1 Manage storage of commodities during emergency<br>3.2 Supply commodities to facilities

3.1 Undertake storage, warehousing and inventory management

- Analyse methods to distribute products among suppliers, distribution centres, warehouses and customers through a logistics network
- Analyse a total systems approach to designing and managing the entire flow of information, materials, and services
- Determine the choice of equipment or materials based on suitability for intended use, accuracy, safety of use and cost
- Analyse the warehouse configuration to have formal storage locations that identify the row, rack section, level and shelf location, typically with an alphanumeric location bar code or label
- Evaluate how medicines are appropriately stored, considering temperature, access and cleanliness
- Determine the appropriate use of, and ability to maintain, the cold chain
- Analyse the movement and storage of materials within a warehouse
- Analyse the process of securing the medical store and limiting access to staff
- Analyse organisation processes, instructions, rules and parameters for warehouse and inventory management and use of equipment (e.g., annual stock turns, expiry date tracking, stock procurement, rotation and retrieval, equipment maintenance)
- Analyse policies and procedures for stock handling, distribution and withdrawal consistent with maintaining safety (e.g., policies for distribution of concentrated electrolyte solutions and cytotoxics, identification of cytotoxic drug products within the work environment, separation of like-named or like-packaged products)
- Analyse requirements for information on the supplies being stored

	» Analyse processes to consolidate several items into larger units for fewer handlings<br>» Analyse methods of stock rotation (e.g., first in first out, FIFO, or first to expire first out, FEFO)<br>» Analyse the process of entering the quantity received and updating the warehouse management system software with the stock on hand when each order is delivered<br>» Determine required levels of stock and/or equipment in specific situations<br>» Measure the accuracy of verification of rolling stocks<br>» Implement the methodology for monitoring the location and condition of the supplies being stored<br>» Analyse information on the storage locations and facilities<br>» Analyse any problems in using the storage locations and facilities
3.1.1 Manage storage of commodities during emergency	» Analyse the different types of warehouses available for emergency or disaster supply<br>» Analyse the choice of storage site for emergency or disaster supply<br>» Evaluate the staff required for the storage of emergency or disaster supply<br>» Determine the equipment and material required for the warehousing of emergency or disaster supply<br>» Conduct a rapid assessment of logistical needs in emergencies
3.2 Supply commodities to facilities	» Determine the sources of distribution demand (customers)<br>» Demonstrate the use of order policies for planned order generation<br>» Calculate re-supply quantities using a variety of inventory methods, including visual review, two bin, periodic review, order point and just in time<br>» Evaluate the process of preparing and using order schedules<br>» Evaluate safety stock calculations<br>» Analyse the use of economic order quantity (EOQ) principles in ordering<br>» Evaluate the effectivity of using pull and push inventory control systems<br>» Determine a regular, rather than urgent, order culture<br>» Determine order-filling priorities in relation to delivery opportunities and urgency<br>» Analyse the parameters under which one must screen orders (e.g. modify order quantities on the basis of available stock, impact on service delivery, distance of facility from hospital)<br>» Evaluate assembling, checking, picking and packing of orders<br>» Demonstrate making and using dispatch lists and invoices<br>» Balance supply with demand, considering both lead time and demand variability created by supply patterns not matching demand patterns<br>» Analyse information on the storage locations and facilities<br>» Measure customer satisfaction and develop loyal customers by using performance metrics taken from the customer perspective, with criteria such as on-time delivery, perception of quality, complaints and length of wait times
Personal/Management Competency(ies):	5.3 Implement risk management and monitoring and evaluation activities for the supply chain<br>5.3.1 Ensure monitoring and evaluation activities are completed<br>5.5 Manage and plan projects

	5.5.2 Execute strategic decision making<br>6.5 Prove leadership abilities<br>6.5.1 Demonstrate resilience and ability to manage stress<br>6.6 Abide by rules / laws / legislation
5.3 Implement risk management and monitoring and evaluation activities for the supply chain	
5.3.1 Ensure monitoring and evaluation activities are completed	» Perform periodic evaluations of pertinent information — such as problem symptoms from knowledgeable sources, carrying these through to the problems, potential causes and root causes of the problem<br>» Determine the process of quantifying the operational performance of similar companies and establish internal targets based on best-in-class results<br>» Analyse key performance measurements and continuous process improvement initiatives to improve process quality on a continual basis<br>» Analyse the results of the analyses provided<br>» Determine the process of controlling and checking errors, taking corrective action so deviation from standards are minimised and the organisation's goals are achieved<br>» Analyse the use of appropriate technological developments to improve the system<br>» Demonstrate that continuous process improvement is an accepted way of organisational life<br>» Demonstrate ways of eliminating unnecessary steps in system design<br>» Analyse processes that strive to eliminate waste<br>» Review processes to encourage sustainability (e.g., reducing carbon footprint, establishing a paperless office, using renewable energy)<br>» Demonstrate the systematic approach used to close process or system performance gaps through streamlining and cycle time reduction, and identify and eliminate causes of quality below specifications, process variation and non-value-adding activities<br>» Determine the relationship between technology and process functionality<br>» Determine how policy, guidelines and regulatory reforms impact SC performance
5.5 Manage and plan projects	
5.5.2 Execute strategic decision making	» Determine how one can make decisions regarding one's own workload and area of responsibility<br>» Determine the process of prioritizing competing tasks and perform them quickly and efficiently according to their importance; finding new ways of organizing or planning work to accomplish it more efficiently<br>» Evaluate the proper time and space for consultation around decisions being made.<br>» Demonstrate the process of considering the input of resources including staff needed

	» Analyse assumptions against facts<br>» Determine the key issues in a complex situation and come to the heart of the problem quickly<br>» Evaluate relevant information before making decisions<br>» Provide input to strategic decisions in the interest of the organisation's goals<br>» Evaluate when to analyse, evaluate and execute tough decisions when necessary<br>» Analyse the impact of decisions on others' work or team goals<br>» Communicate decisions and ensure they are incorporated into policies and processes<br>» Demonstrate inclusive and consultative strategic planning to establish strategic objectives<br>» Evaluate emerging trends and practices<br>» Analyse the macro and long-term consequences of decisions
6.5 Prove leadership abilities	» Analyse input to meetings and programme development<br>» Look for future trends and issues, and assist the organisation in meeting the challenges<br>» Contribute to a collaborative working environment<br>» Demonstrate a drive for change and improvement develops opportunities for the organisation and sector<br>» Demonstrate channelling energy and ideas toward resolving issues<br>» Demonstrate the practise of communicating with influence<br>» Demonstrate showing courage to take an unpopular stance when needed<br>» Demonstrate steering and implementing change organisationally<br>» Demonstrate anticipating and resolving conflict<br>» Demonstrate showing initiative in working methods<br>» Exemplify personal drive and integrity<br>» Demonstrate serving as a role model for others to follow<br>» Demonstrate leadership and practice management skills, initiative and efficiency<br>» Demonstrate inspiring others through professional excellence, innovation and communication<br>» Determine situations in which a change in leadership style would be warranted (e.g., directive versus consultative style in the event of a crisis)
6.5.1 Demonstrate resilience and ability to manage stress	» Demonstrate identifying symptoms of stress and take steps to reduce stress<br>» Demonstrate seeing the bigger picture and help others to<br>» Demonstrate recovering quickly from setbacks<br>» Demonstrate drawing on previous experience and support mechanisms to reduce the impact of stress on self and others<br>» Determine a working environment that aims to minimise pressure and stress<br>» Demonstrate coping well under pressure, particularly in difficult environments

- Demonstrate acting as a role model for others and display courage under difficult circumstances
- Demonstrate recognising the limitations of staff and taking action to limit their exposure to harm when needed
- Demonstrate making use of personal support mechanisms
- Demonstrate helping others identify personal support mechanisms
- Determine organisational policy to support self-care in agencies

6.6 Abide by rules / laws / legislation

- Demonstrate compliance with the legislation that covers the practice of public health supply chains and healthcare, and describe its purpose
- Analyse requirements of professional codes, guidelines and standards adopted as part of the legislative framework
- Demonstrate keeping up to date with changes in legal instruments, as informed by national-level managers
- Demonstrate compliance with international regulations in decision making for the distribution system, including customs regulations on import and export requirements (e.g., trade tariffs and duties on imported goods) and security regulations (e.g., 2007 SAFE Ports Act, NAFTA or European Union trade agreements)
- Demonstrate applying and understanding regulatory affairs and the key aspects of pharmaceutical registration and legislation
- Demonstrate knowledge of the principles of business economics and intellectual property rights, including the basics of patent interpretation
- Demonstrate knowledge of applicable procurement law and the practical effects of a contract’s terms and conditions
- Evaluate current industry and government regulations governing sustainability
- Demonstrate a working knowledge of key hazardous materials handling laws
- Determine policies and procedures relating to workplace safety that are consistent with agreed or recognised standards
- Analyse professional standards and conventions, as well as workplace policies and procedures, for preparing pharmaceutical products
- Demonstrate all standard operating procedures
- Evaluate the process of complying with data privacy and data protection laws

Basal Technology Competency(ies):

7.4 Temperature and monitoring

7.6 ERP includes function of LMIS

7.12 Basic Office Skills

7.13 Have a command of technology

7.4 Temperature and monitoring

- Analyse the current technology approaches for Temperature monitoring and sensors
- Evaluate what is required to implement this technology in a country context
- Evaluate infrastructure requirement for heat-sensitive items at peripheral ‘lower level’ of SC depending on volume and nature of items to handle;
- Analyse regular preventive maintenance and calibration of cold-chain equipment with biomedical engineers or logisticians

	» Evaluate the policy for temperature monitoring tools during transportation and storage<br>» Demonstrate the steps involved in health logistic management cycle: selection-order-delivery-reception-storage-distribution-inventory<br>» Determine hardware qualification and system validation of temperature monitoring<br>» Evaluate mass data management of temperature monitoring
7.6 ERP includes function of LMIS	» Evaluate a Master Data Management System<br>» Demonstrate an understanding of the critical components of an ERP system<br>» Demonstrate an understanding of the meaning of common document types found within an ERP/LMIS<br>» Demonstrate an understanding of the logic of data visibility within an ERP<br>» Demonstrate the importance of data integrity, record management and information security
7.12 Basic Office Skills	» Demonstrate a good understanding of common presentation authoring packages<br>» Demonstrate a good understanding of common spreadsheet authoring packages<br>» Demonstrate a good understanding of common document authoring packages<br>» Demonstrate a good understanding of common email authoring packages<br>» Demonstrate a good understanding of common instant messaging packages<br>» Demonstrate a good understanding of common video conferencing authoring packages
7.13 Have a command of technology	» Demonstrate the use of technology suitable to the job held<br>» Monitor new developments and technologies in the sector<br>» Experiment with new technologies and recognising potential benefits for the sector<br>» Ensure that resources and support are provided across the organisation to enable colleagues to make the best use of available technology<br>» Determine what the organisations' strategy is for technology use<br>» Determine how to minimise environmental damage through technology use<br>» Analyse the use of technology to maximise effectiveness and efficiency<br>» Analyse the use of field-based technology (e.g., radio, general packet radio service (GPRS), satellite phone)
Advanced Technology Competency(ies):	7.2 Blockchain<br>7.11 Cloud Computing
7.2 Blockchain	» Evaluate blockchain as a value-add capability for relevant global health use cases
7.11 Cloud Computing	» Evaluate cloud computing as a value-add capability for relevant global health use cases

Key Performance Indicators:

- Accuracy of Risk Register
- Cycle time of QA/QC report
- Overall value at risk
- Partner/Donor integration, policy, guidelines and regulatory reforms
- QA/QC report
- Risk / Mitigation Costs
- Time to Recovery (TTR)
- Adherence to formulisation of Supply Chain Performance Requirements for Planning, Procurement (including contracts and Donor and Global Partner agreements. Actual formalised agreements versus total Donor and Global Partner programmes
- Supply Chain Master Data Reliability - Vendor Master Data accuracy audit vendor master data and determine faulty records versus total vendor records
- Supply Chain Master Data Reliability - Item Master Data Accuracy. Audit Item /SKU Mater Data accuracy and determine total faulty or incomplete records versus total records expressed as a percentage.

Training:	Qualifications Available:	Certifications Available:
» SC Optimization<br>» Scenario Planning<br>» Integrated Business/ SC Planning<br>» Supply chain Risk<br>» Executive training<br>» Advanced Supply Chain Management	» Master of Philosophy in Business Management with options in Supply Chain Management<br>» Postgraduate Diploma in Archives and Records Management	» Occupational Certificate: Supply and Distribution Manager<br>» APICS Certified in logistics transportation and distribution certificate<br>» Lean Six Sigma Certification in Warehousing black belt<br>» APICS Certified Supply Chain Professional

Managerial

Lead - Partners Integration

Organisation: Public Health System Organisation				
Competency Level:	Serial Number:	Primary Process:	Job Role:	Supervises:
**Managerial**	**5-55-66-4**	**Enable – Regulatory (Individual) Compliance**	**Lead - Partners Integration**	**-**

Contributions	Responsible for liaising and coordinating with partner organisations, understanding partner organisation drivers and finding win-win situations between them.
Personal/Management Competency(ies):	5.5 Manage and plan projects<br>5.5.1 Develop and direct project plans<br>5.5.2 Execute strategic decision making<br>5.5.3 Manage partnerships<br>5.5.4 Direct/participate in teamwork<br>6.1 Demonstrate generic skills (e.g., literacy, numeracy, technology)<br>6.1.1 Exhibit high understanding of literacy and numeracy<br>6.2 Demonstrate strong communication skills<br>6.2.1 Practice cultural awareness<br>6.6 Abide by rules / laws / legislation

5.5 Manage and plan projects

5.5.1 Develop and direct project plans

- » Evaluate obstacles and critical events for ensuring the six rights of logistics management (right quantity, product, condition, place, time and cost) and develop contingency plans to address them; monitor progress and take necessary corrective action when needed
- » Determine an operational plan that is consistent with the strategic plan, links specific goals and strategies, and identifies the performance indicators to be used to measure achievements
- » Demonstrate the use of information gathering techniques from stakeholders, analysing situations and identifying implications to make correct decisions
- » Review the questions, “Where are we going?” and “How are we going to get there?” and create a specific and purposeful path to achieve this.
- » Demonstrate the process reference model to managing supply chain processes (i.e., integrating business process re-engineering, benchmarking and process measurement)
- » Analyse the current state of a process and derive the desired future state
- » Demonstrate the process of selecting and applying tools or technological solutions to frequently encountered problems
- » Analyse the process of providing staff with the necessary tools to carry out plans

	» Demonstrate the ability to monitor progress and make changes as required<br>» Convene meetings for planning, organisation and monitoring logistics activities<br>» Ensure staff are aware that they are accountable for achieving the desired results<br>» Determine how planning, organising and managing resources will bring about the successful completion of projects<br>» Determine the key features of a successful change management strategy
5.5.2 Execute strategic decision making	» Determine how one can make decisions regarding one's own workload and area of responsibility<br>» Determine the process of prioritizing competing tasks and perform them quickly and efficiently according to their importance; finding new ways of organizing or planning work to accomplish it more efficiently<br>» Evaluate the proper time and space for consultation around decisions being made.<br>» Demonstrate the process of considering the input of resources including staff needed<br>» Analyse assumptions against facts<br>» Determine the key issues in a complex situation and come to the heart of the problem quickly<br>» Evaluate relevant information before making decisions<br>» Provide input to strategic decisions in the interest of the organisation's goals<br>» Evaluate when to analyse, evaluate and execute tough decisions when necessary<br>» Analyse the impact of decisions on others' work or team goals<br>» Communicate decisions and ensure they are incorporated into policies and processes<br>» Demonstrate inclusive and consultative strategic planning to establish strategic objectives<br>» Evaluate emerging trends and practices<br>» Analyse the macro and long-term consequences of decisions
5.5.3 Manage partnerships	» Demonstrate the concepts of partnership working<br>» Determine the process of supporting implementation of partnership programmes<br>» Analyse the process of experimenting with and trialling new ideas with partners<br>» Analyse partnerships to deliver programme and increase impact<br>» Analyse innovation and creativity in partnership work<br>» Communicate key information with partner members<br>» Involve and value partners in all aspects of programming<br>» Analyse problems with supply chain relationships<br>» Demonstrate the process of communicating effectively with nurses, doctors and other members of the healthcare team<br>» Analyse the process of responding to consumer complaints or comments about services and/or advice received<br>» Analyse collaboration and consultation across the sector and encourage the team to work with others to gain market insight

	» Encourage input from other key actors in the humanitarian sector and develop relationships with a cross-section of actors<br>» Analyse the process of meeting the reporting requirements of vertical programmes<br>» Analyse the structure of the health system at a national level and explain this to others<br>» Analyse the roles of other members of the healthcare team (including with consumers) in a way that engenders understanding and confidence in the team and its members<br>» Analyse the structure of the organisation, environment and/or service in which they work<br>» Demonstrate how vertical programmes work within the health system<br>» Evaluate the communication network established to achieve work outcomes<br>» Demonstrate knowledge of the structure of the health system at the provincial/regional level and explain this to others<br>» Analyse the roles and functions of government agencies in regulating and supporting supply chain organisations<br>» Demonstrate knowledge of change management (e.g. PtD Theory of Change)
5.5.4 Direct/participate in teamwork	» Work with team members within their area of authority to establish achievable goals and strategies that are consistent with the objectives established for the organisation as a whole<br>» Evaluate an organisational chart that shows the lines of reporting and responsibility among staff<br>» Determine a culture of actively contributing a perspective and making a positive contribution to team-based problem solving and decision making<br>» Determine a culture of providing feedback, encouragement and support to team members for progressing strategic goals<br>» Monitor team performance in relation to the organisation's mission and goals<br>» Enable the creation of a culture of teams thinking for themselves and resolving problems<br>» Demonstrate the process of encouraging and harnessing diversity within the team to boost team effectiveness<br>» Demonstrate the process of encouraging and supporting the team to work through its stages of development and perform well<br>» Determine roles and responsibilities in relation to employees' expertise and the expectations of collaborating team members<br>» Demonstrate where their position fits in the structure (organisation, SC, healthcare sector) , the responsibilities their position entails and the accountabilities linked to the position
6.1 Demonstrate generic skills (e.g., literacy, numeracy, technology)	
6.1.1 Exhibit high understanding of	» Encourage speaking clearly and confidently, organizing information in a logical manner, and considering voice tone and pace<br>» Demonstrate responding to verbal messages and other cues (active listening)

literacy and numeracy	» Demonstrate expressing ideas and opinions clearly in written and verbal form<br>» Demonstrate communicating information accurately, concisely and confidently in writing and verbally<br>» Demonstrate a level of mathematics suitable to the job held<br>» Demonstrate knowledge of visual presentation techniques, including charting, histograms and flow sheets<br>» Demonstrate reading and comprehending at a level necessary to properly complete duties of the position<br>» Demonstrate the process of interpreting written information in documents, such as reports, SOPs, LMIS forms, graphs, calendars, schedules, notices and directions<br>» Demonstrate paying attention to detail and identifying the main ideas, detecting inconsistencies and identifying missing information in documents<br>» Demonstrate computer skills — such as using Word, Excel, PowerPoint and the internet — suitable to the job level<br>» Demonstrate the process of analysing and validating KPIs<br>» Participate in the evaluation of activities that act as standards against which the KPIs are measured<br>» Demonstrate the process of performing critical self-review on work before submission
6.2 Demonstrate strong communication skills	» Demonstrate being truthful and trustworthy, and supplying accurate information at all times<br>» Demonstrate working as part of a workplace team<br>» Determine vocabulary and communication style and form for both written and verbal communication that is appropriate for the situation, audience and material being communicated (e.g., avoid unnecessary jargon, clearly explain medical and SCM terminology)<br>» Demonstrate open communication within constraints of confidentiality<br>» Analyse communications about logistics<br>» Demonstrate participation in meetings, expressing one’s opinions, being aware of others’ needs, and being appropriately assertive when required<br>» Demonstrate perspective into the point of view of others, understanding their needs and goals<br>» Demonstrate the practise of building trust, rapport and credibility with others<br>» Determine the communication needs and concerns of others and respond to them<br>» Demonstrate avoiding conflict between work and personal interests<br>» Demonstrate the practice of meaningful two-way communication<br>» Demonstrate influence on others by persuasively presenting thoughts and ideas<br>» Demonstrate assertiveness skills to deal with unreasonable requests and/or refusals that would compromise practice or consumer care<br>» Demonstrate a positive, supportive and appreciative attitude<br>» Demonstrate the ability to balance the organisation’s needs and the customer’s needs<br>» Demonstrate self-control by maintaining composure and keeping emotions in check, even in difficult situations; deal calmly and effectively with stressful situations

	» Demonstrate the expressing of opinions and providing information in written and/or verbal form in a manner that does not elicit concern, anger or other adverse response<br>» Demonstrate the use of a systematic process for following up that demonstrates written reports have been received and understood<br>» Evaluate the means by which responses to input to the work environment are monitored<br>» Seek practical ways to overcome barriers to communication<br>» Ensure language representation (e.g., interpreters) at meetings when appropriate<br>» Demonstrate tackling difficult situations and resolving disputes between staff<br>» Demonstrate giving both positive and negative feedback sensitively<br>» Demonstrate maintaining and improving communication<br>» Demonstrate the process of giving and requesting feedback frequently<br>» Demonstrate the ability to communicate effectively with shop stewards and union representatives
6.2.1 Practice cultural awareness	» Demonstrate cultural awareness and sensitivity, treating all people with fairness, respect and dignity<br>» Demonstrate anti-discriminatory practices in the organisation, including HR and disciplinary procedures<br>» Demonstrate cultural sensitivity, equality and fairness at all levels of the organisation<br>» Demonstrate challenging discriminatory behaviour directly and sensitively<br>» Demonstrate acting in a non-discriminatory way toward individuals and groups<br>» Demonstrate the process of integrating cultural awareness in learning and development approaches<br>» Demonstrate avoiding stereotypical responses by examining one's own behaviour and bias<br>» Demonstrate managing cultural diversity in teams and make the most of differences<br>» Demonstrate the use of strategies and/or resources for communicating effectively with people from different cultural backgrounds<br>» Supply information relating to values, beliefs and cultural backgrounds of consumers that may influence the way professional services are provided
6.6 Abide by rules / laws / legislation	» Demonstrate compliance with the legislation that covers the practice of public health supply chains and healthcare, and describe its purpose<br>» Analyse requirements of professional codes, guidelines and standards adopted as part of the legislative framework<br>» Demonstrate keeping up to date with changes in legal instruments, as informed by national-level managers<br>» Demonstrate compliance with international regulations in decision making for the distribution system, including customs regulations on import and export requirements (e.g., trade tariffs and duties on imported goods) and security regulations (e.g., 2007 SAFE Ports Act, NAFTA or European Union trade agreements)<br>» Demonstrate applying and understanding regulatory affairs and the key aspects of pharmaceutical registration and legislation

- » Demonstrate knowledge of the principles of business economics and intellectual property rights, including the basics of patent interpretation
- » Demonstrate knowledge of applicable procurement law and the practical effects of a contract's terms and conditions
- » Evaluate current industry and government regulations governing sustainability
- » Demonstrate a working knowledge of key hazardous materials handling laws
- » Determine policies and procedures relating to workplace safety that are consistent with agreed or recognised standards
- » Analyse professional standards and conventions, as well as workplace policies and procedures, for preparing pharmaceutical products
- » Demonstrate all standard operating procedures
- » Evaluate the process of complying with data privacy and data protection laws

Basal Technology Competency(ies):	7.12 Basic Office Skills<br>7.13 Have a command of technology

7.12 Basic Office Skills

- » Demonstrate a good understanding of common presentation authoring packages
- » Demonstrate a good understanding of common spreadsheet authoring packages
- » Demonstrate a good understanding of common document authoring packages
- » Demonstrate a good understanding of common email authoring packages
- » Demonstrate a good understanding of common instant messaging packages
- » Demonstrate a good understanding of common video conferencing authoring packages

7.13 Have a command of technology

- » Demonstrate the use of technology suitable to the job held
- » Monitor new developments and technologies in the sector
- » Experiment with new technologies and recognising potential benefits for the sector
- » Ensure that resources and support are provided across the organisation to enable colleagues to make the best use of available technology
- » Determine what the organisations' strategy is for technology use
- » Determine how to minimise environmental damage through technology use
- » Analyse the use of technology to maximise effectiveness and efficiency
- » Analyse the use of field-based technology (e.g., radio, general packet radio service (GPRS), satellite phone)

Key Performance Indicators:

- » % of formalized agreements between partners and donors. Actual number of partner and donor agreements versus formalized agreements
- » Regular Feedback to Donors and Partners on performance of programmes and or donations

Training:	Qualifications Available:	Certifications Available:

» Legislation and Standards<br>» International trade regulation<br>» Partnership Management	» Bachelor of Commerce in Philosophy, Politics and Economics<br>» Bachelor of Science Honours: Pharmacology<br>» Bachelor of Accounting Honours	» APICS Certified in production and inventory management certificate<br>» Lean Six Sigma Certification in Healthcare black belt

Manager – Contracts and Agreements

Organisation:

Public Health System Organisation

Competency Level:	Serial Number:	Primary Process:	Job Role:	Supervises:
Managerial	2-242-23-3	Enable – Contracts Agreements	Manager – Contracts and Agreements	Manager - Contract and 3PLs, Specialist – Contracts
Contributions	Maintains and enhances the Contractual capacity of the Public Health System Organisation. Ensures that the Enable processes are adhered to as prescribed in the Public Health System Organisation's Operating Model. Maintains contract and agreement records current and relevant and deploys appropriate governance methodology to each contract or agreement.			
Technical Competency(ies):	2.3 Manage tendering processes and supplier agreements<br>2.4 Undertake contract management and risk<br>2.4.1 Place commodity orders<br>2.4.2 Manage contracts<br>2.4.3 Addresses risk and ensures quality management			

2.3 Manage tendering processes and supplier agreements

- Demonstrate the correct use of request for qualifications (RFQ), invitations to bid (ITB) and request for proposals (RFP) methods
- Demonstrate knowledge of local and international tendering procedures (e.g., open competitive bidding, restricted tender, competitive negotiation, direct procurement)
- Write detailed specifications for tenders
- Describe the high-level guidance required for high-value and politically sensitive procurements
- Describe the process of developing and managing contracts
- Describe the procurement, return and exchange policies
- List appropriate legislation and policy in regard to procurement processes, and what is required to comply
- Ensure ethical behaviour, abiding by conflict of interest policies
- Describe the process of evaluating tender bids based on the specification and evaluation criteria including capability of the supplier, problems relating to outcomes of the contract.
- Follow contract approval process, including contract negotiations
- Discuss some of the key issues relevant to negotiating supply contracts (e.g., volume usage over time, price volume agreements, alternate supplier clauses, duration of contract, period of review, terms for contract termination and renewal)
- Describe the process of awarding and managing contracts, resolving any issues as they arise
- Describe the process of debriefing unsuccessful suppliers

	» Describe the ways in which compliance with purchasing policies and procedures is monitored<br>» Participate in procurement planning including a yearly activities schedule and developing a wide range of briefs, specifications and commercial documentation<br>» Identify strategic sourcing projects, capturing benefits, work requirements, impacts and cost savings<br>» Participate in cross-functional teams handling strategic procurements<br>» Describe effective internal and external procurement networks<br>» List local and international health commodity prices and understand the factors affecting them<br>» Describe the process of engaging clients across the public sector in presentations demonstrating the benefits of a range of strategic projects designed to ensure cost savings and value for money for government procurement
2.4 Undertake contract management and risk	
2.4.1 Place commodity orders	» Obtain authorisations to administer the contracts<br>» Describe the type of supplies required<br>» Obtain reference sources and supplier catalogues to clarify required product and its availability<br>» Comply with policies and procedures to order required stock and equipment<br>» Confirm the orders with suppliers<br>» Identify any problems in placing orders<br>» Comply with procedures for placing orders<br>» Identify any problems with order delivery and recommend options for progressing order delivery<br>» Ensure effective mechanisms for checking invoices exist and take action to adjust payments accordingly<br>» Receive or raise requisitions and arrange purchase orders and payments
2.4.2 Manage contracts	» Conduct reviews of performance against agreed key performance indicators<br>» Provide timely and expert guidance to remediate procurement performance issues<br>» Describe contract responsiveness and negotiate changes to a contract (contract variations)<br>» Manage contract disputes and terminate contracts legally and appropriately if necessary<br>» Describe the importance of supplier control mechanisms, contract administration and supplier management
2.4.3 Addresses risk and ensures quality management	» Describe the process of providing risk assessments for the procurement process (technical, commercial, administrative)<br>» Use key performance indicators for assessing procurement and supplier performance and effectiveness<br>» Identify any problems with the procurement of supplies in the supply chain<br>» Identify the contingency plan for shortages<br>» Describe recommended options for improving the performance of suppliers

	» Apply procurement portfolio analysis and risk assessment (supply positioning)<br>» Describe the fundamentals of risk planning and assessment
Personal/Management Competency(ies):	6.1 Demonstrate generic skills (e.g., literacy, numeracy, technology)<br>6.1.1 Exhibit high understanding of literacy and numeracy<br>6.2 Demonstrate strong communication skills<br>6.2.1 Practice cultural awareness
6.1 Demonstrate generic skills (e.g., literacy, numeracy, technology)	
6.1.1 Exhibit high understanding of literacy and numeracy	» Speak clearly and confidently, organize information in a logical manner, and consider voice tone and pace<br>» Respond to verbal messages and other cues (active listening)<br>» Express ideas and opinions clearly in written and verbal form<br>» Communicate information accurately, concisely and confidently in writing and verbally<br>» Practice a level of mathematics suitable to the job held<br>» Describe visual presentation techniques, including charting, histograms and flow sheets<br>» Read and comprehend at a level necessary to properly complete duties of the position<br>» Interpret written information in documents, such as reports, SOPs, LMIS forms, graphs, calendars, schedules, notices and directions<br>» Pay attention to detail and identify the main ideas, detect inconsistencies and identify missing information in documents<br>» Apply computer skills — such as using Word, Excel, PowerPoint and the internet — suitable to the job level<br>» Describe the process of analysing and validating KPIs<br>» Participate in the evaluation of activities that act as standards against which the KPIs are measured<br>» Apply the process of performing critical self-review on work before submission
6.2 Demonstrate strong communication skills	» Be truthful and trustworthy, and supply accurate information at all times<br>» Work as part of a workplace team<br>» Describe vocabulary and communication style and form for both written and verbal communication that is appropriate for the situation, audience and material being communicated (e.g., avoid unnecessary jargon, clearly explain medical and SCM terminology)<br>» Encourage open communication within constraints of confidentiality<br>» Organize communications about logistics<br>» Encourage participation in meetings, expressing one's opinions, being aware of others' needs, and being appropriately assertive when required

	» Apply perspective into the point of view of others, understanding their needs and goals<br>» Encourage building trust, rapport and credibility with others<br>» Anticipate the communication needs and concerns of others and respond to them<br>» Avoid conflict between work and personal interests<br>» Encourage meaningful two-way communication<br>» Influence others by persuasively presenting thoughts and ideas<br>» Apply assertiveness skills to deal with unreasonable requests and/or refusals that would compromise practice or consumer care<br>» Maintain a positive, supportive and appreciative attitude<br>» Apply the ability to balance the organisation's needs and the customer's needs<br>» Use self-control by maintaining composure and keeping emotions in check, even in difficult situations; deal calmly and effectively with stressful situations<br>» Encourage the expressing of opinions and providing information in written and/or verbal form in a manner that does not elicit concern, anger or other adverse response<br>» Apply the use of a systematic process for following up that demonstrates written reports have been received and understood<br>» Describe the means by which responses to input to the work environment are monitored<br>» Seek practical ways to overcome barriers to communication<br>» Identify language representation (e.g., interpreters) at meetings when appropriate<br>» Tackle difficult situations and resolve disputes between staff<br>» Give both positive and negative feedback sensitively<br>» Maintain and improve communication<br>» Apply the process of giving and requesting feedback frequently<br>» Apply the ability to communicate effectively with shop stewards and union representatives
6.2.1 Practice cultural awareness	» Describe cultural awareness and sensitivity, treating all people with fairness, respect and dignity<br>» Describe anti-discriminatory practices in the organisation, including HR and disciplinary procedures<br>» Establish cultural sensitivity, equality and fairness at all levels of the organisation<br>» Challenge discriminatory behaviour directly and sensitively<br>» Act in a non-discriminatory way toward individuals and groups<br>» Integrate cultural awareness in learning and development approaches<br>» Avoid stereotypical responses by examining one's own behaviour and bias<br>» Manage cultural diversity in teams and make the most of differences<br>» Describe strategies and/or resources for communicating effectively with people from different cultural backgrounds

	» Elicit information relating to values, beliefs and cultural backgrounds of consumers that may influence the way professional services are provided
Basal Technology Competency(ies):	7.6 ERP includes function of LMIS<br>7.12 Basic Office Skills<br>7.13 Have a command of technology
7.6 ERP includes function of LMIS	» Support a Master Data Management System<br>» Describe the critical components of an ERP system<br>» Describe the meaning of common document types found within an ERP/LMIS<br>» Describe the logic of data visibility within an ERP<br>» Describe the importance of data integrity, record management and information security
7.12 Basic Office Skills	» Have a good understanding of common presentation authoring packages<br>» Have a good understanding of common spreadsheet authoring packages<br>» Have a good understanding of common document authoring packages<br>» Have a good understanding of common email authoring packages<br>» Have a good understanding of common instant messaging packages<br>» Have a good understanding of common video conferencing authoring packages
7.13 Have a command of technology	» Apply the use of technology suitable to the job held<br>» Provide input on monitoring new developments and technologies in the sector<br>» Identify new technologies and recognising potential benefits for the sector<br>» Identify resources and support that are provided across the organisation to enable colleagues to make the best use of available technology<br>» Ensure that the organisation has a strategy for technology use<br>» Describe how to minimise environmental damage through technology use<br>» Use technology to maximise effectiveness and efficiency<br>» Use field-based technology (e.g., radio, general packet radio service (GPRS), satellite phone)
Advanced Technology Competency(ies):	7.2 Blockchain<br>7.11 Cloud Computing
7.2 Blockchain	» Describe blockchain as a value-add capability for relevant global health use cases

7.11 Cloud Computing	» Describe cloud computing as a value-add capability for relevant global health use cases

Key Performance Indicators:

- On Time Closure of Tenders
- Procurement cycle time
- Tender creation cycle time
- Accuracy of tender documents
- Quality of Supplier Relationship
- Tenders adjudication and allocation on Time performance
- Tenders allocated in accordance with Business Rules
- On Time Closure of Tenders
- Vendor/Supplier Master Data Accuracy
- Item Master Data Accuracy
- Risk avoidance, elimination and or Mitigation Costs
- inbound Transportation Master Data Accuracy by Item SKU
- Item SKU Lead Time Accuracy

Training:	Qualifications Available:	Certifications Available:
» Performance Management<br>» Contract Management<br>» Outsourcing<br>» Legal Frameworks<br>» Basic Transportation Management<br>» Consignment Agreement Development<br>» Customer/Supplier Communication<br>» Intellectual Property/Proprietary Data<br>» International Trade<br>» Procurement<br>» Subcontracting Types (FFP, CP, CPAF, Performance Based)<br>» Supplier Relationship Management (SRM)		» CIPS Level 4 Diploma in Procurement and Supply<br>» SPSM2® (Global Procurement Management)<br>» SPSM ® (Essential Procurement Skills)

Lead – Compliance and Risk Management

Organisation:

Public Health System Organisation

Competency Level:	Serial Number:	Primary Process:	Job Role:	Supervises:
Managerial	5-531-542-3	Enable – Regulatory (Individual) Compliance	Lead – Compliance and Risk Management	-

Contributions	Participates in the Audit and Risk Committee's environmental scanning for Risk. Alerts the committee and relevant members to the probability of risk occurring, investigates and determines possible risk mitigation strategies and actions. Ensures that the Risk enabler process is followed as per the Public Health System Organisation's Operating Model. Updates the risk register. Tests the process for compliance and monitors the risk and compliance metrics for results that are out of the performance bands.
Personal/Management Competency(ies):	5.3 Implement risk management and monitoring and evaluation activities for the supply chain<br>5.3.1 Ensure monitoring and evaluation activities are completed<br>5.4 Manage outsourcing of SCM functions<br>6.1 Demonstrate generic skills (e.g., literacy, numeracy, technology)<br>6.1.1 Exhibit high understanding of literacy and numeracy<br>6.2 Demonstrate strong communication skills<br>6.2.1 Practice cultural awareness

5.3 Implement risk management and monitoring and evaluation activities for the supply chain

5.3.1 Ensure monitoring and evaluation activities are completed

- » Describe the periodic evaluations to maintain processes by gathering pertinent information — such as problem symptoms from knowledgeable sources, carrying these through to the problems, potential causes and root causes of the problem
- » Quantify the operational performance of similar companies and establish internal targets based on best-in-class results
- » Apply key performance measurements and continuous process improvement initiatives to improve process quality on a continual basis
- » Provide results of the analyses to colleagues
- » Control and check errors, taking corrective action so deviation from standards are minimised and the organisation's goals are achieved
- » Identify the use of appropriate technological developments to improve the system
- » Describe that continuous process improvement is an accepted way of organisational life
- » Execute ways of eliminating unnecessary steps in system design

	» Apply processes that strive to eliminate waste<br>» Apply processes to encourage sustainability (e.g., reducing carbon footprint, establishing a paperless office, using renewable energy)<br>» Describe the systematic approach used to close process or system performance gaps through streamlining and cycle time reduction, and identify and eliminate causes of quality below specifications, process variation and non-value-adding activities<br>» Outline the relationship between technology and process functionality<br>» Describe how policy, guidelines and regulatory reforms impact SC performance
5.4 Manage outsourcing of SCM functions	» Provide input on negotiating and compiling contracts with outside parties for delivery of logistics services
6.1 Demonstrate generic skills (e.g., literacy, numeracy, technology)	
6.1.1 Exhibit high understanding of literacy and numeracy	» Speak clearly and confidently, organize information in a logical manner, and consider voice tone and pace<br>» Respond to verbal messages and other cues (active listening)<br>» Express ideas and opinions clearly in written and verbal form<br>» Communicate information accurately, concisely and confidently in writing and verbally<br>» Practice a level of mathematics suitable to the job held<br>» Describe visual presentation techniques, including charting, histograms and flow sheets<br>» Read and comprehend at a level necessary to properly complete duties of the position<br>» Interpret written information in documents, such as reports, SOPs, LMIS forms, graphs, calendars, schedules, notices and directions<br>» Pay attention to detail and identify the main ideas, detect inconsistencies and identify missing information in documents<br>» Apply computer skills — such as using Word, Excel, PowerPoint and the internet — suitable to the job level<br>» Describe the process of analysing and validating KPIs<br>» Participate in the evaluation of activities that act as standards against which the KPIs are measured<br>» Apply the process of performing critical self-review on work before submission
6.2 Demonstrate strong communication skills	» Be truthful and trustworthy, and supply accurate information at all times<br>» Work as part of a workplace team<br>» Describe vocabulary and communication style and form for both written and verbal communication that is appropriate for the situation, audience and material being communicated (e.g., avoid unnecessary jargon, clearly explain medical and SCM terminology)

	» Encourage open communication within constraints of confidentiality<br>» Organize communications about logistics<br>» Encourage participation in meetings, expressing one's opinions, being aware of others' needs, and being appropriately assertive when required<br>» Apply perspective into the point of view of others, understanding their needs and goals<br>» Encourage building trust, rapport and credibility with others<br>» Anticipate the communication needs and concerns of others and respond to them<br>» Avoid conflict between work and personal interests<br>» Encourage meaningful two-way communication<br>» Influence others by persuasively presenting thoughts and ideas<br>» Apply assertiveness skills to deal with unreasonable requests and/or refusals that would compromise practice or consumer care<br>» Maintain a positive, supportive and appreciative attitude<br>» Apply the ability to balance the organisation's needs and the customer's needs<br>» Use self-control by maintaining composure and keeping emotions in check, even in difficult situations; deal calmly and effectively with stressful situations<br>» Encourage the expressing of opinions and providing information in written and/or verbal form in a manner that does not elicit concern, anger or other adverse response<br>» Apply the use of a systematic process for following up that demonstrates written reports have been received and understood<br>» Describe the means by which responses to input to the work environment are monitored<br>» Seek practical ways to overcome barriers to communication<br>» Identify language representation (e.g., interpreters) at meetings when appropriate<br>» Tackle difficult situations and resolve disputes between staff<br>» Give both positive and negative feedback sensitively<br>» Maintain and improve communication<br>» Apply the process of giving and requesting feedback frequently<br>» Apply the ability to communicate effectively with shop stewards and union representatives
6.2.1 Practice cultural awareness	» Describe cultural awareness and sensitivity, treating all people with fairness, respect and dignity<br>» Describe anti-discriminatory practices in the organisation, including HR and disciplinary procedures<br>» Establish cultural sensitivity, equality and fairness at all levels of the organisation<br>» Challenge discriminatory behaviour directly and sensitively<br>» Act in a non-discriminatory way toward individuals and groups<br>» Integrate cultural awareness in learning and development approaches<br>» Avoid stereotypical responses by examining one's own behaviour and bias

	» Manage cultural diversity in teams and make the most of differences<br>» Describe strategies and/or resources for communicating effectively with people from different cultural backgrounds<br>» Elicit information relating to values, beliefs and cultural backgrounds of consumers that may influence the way professional services are provided
Basal Technology Competency(ies):	7.1 Data Science<br>7.5 Planning systems<br>7.6 ERP includes function of LMIS<br>7.12 Basic Office Skills
7.1 Data Science	» Describe the end to end MIS technology components that are inherent in advanced MIS systems and explore how these may be relevant in different supply chain contexts<br>» Identify emerging technological approaches to data visualization and analysis<br>» Identify how data science can be applied within supply chain<br>» Ability to interact, provide guidance for data (dashboard) consolidation in various levels and use the tech tools<br>» Support movement of data into electronic form at lowest/remote SC level<br>» Ability to work with relational database
7.5 Planning systems	» Describe blockchain and understand the building blocks for planning systems<br>» Describe planning systems as a value-add capability for relevant global health use cases
7.6 ERP includes function of LMIS	» Support a Master Data Management System<br>» Describe the critical components of an ERP system<br>» Describe the meaning of common document types found within an ERP/LMIS<br>» Describe the logic of data visibility within an ERP<br>» Describe the importance of data integrity, record management and information security
7.12 Basic Office Skills	» Have a good understanding of common presentation authoring packages<br>» Have a good understanding of common spreadsheet authoring packages<br>» Have a good understanding of common document authoring packages<br>» Have a good understanding of common email authoring packages<br>» Have a good understanding of common instant messaging packages<br>» Have a good understanding of common video conferencing authoring packages
Advanced Technology Competency(ies):	7.2 Blockchain<br>7.7 Automation<br>7.11 Cloud Computing

7.2 Blockchain	» Describe blockchain as a value-add capability for relevant global health use cases
7.7 Automation	» Describe automation as a value-add capability for relevant global health use cases
7.11 Cloud Computing	» Describe cloud computing as a value-add capability for relevant global health use cases

Key Performance Indicators:

- » Evaluates and updates Risk Register, Risk Register up-to-date following Risk and Audit Committee meetings
- » Number of breaches in compliance to business rules versus total number of transactions
- » Audits or reviews audit reports for Cold Supply Chain, Cycle counts, Supplier Performance, Transportation Performance, Delivery Performance and flags items out of performance bands for further investigation. Number of investigations versus Number of deviations
- » Percentage number of transactions breaching legislation versus number of transactions in a period.
- » Number of security and access breaches and reports thereon. Launches root cause analysis investigations and proposes corrective actions.
- » Evaluates Risk Mitigation, Avoidance and Elimination efforts. Number of times risk occurs and Mitigation, Avoidance and or Elimination failed. Total Failed occurrences versus total Items on Risk Register

Training:	Qualifications Available:	Certifications Available:
» Risk and exception management<br>» Supply Chain Performance Measurements<br>» Risk Assessment<br>» Risk Identification<br>» Risk Mitigation<br>» Risk Response Planning	» (CILT) International Diploma in Logistics Transport	

Operational
Manager - Occupational Safety

Organisation

Public Health System Organisation

Competency Level:	Serial Number:	Primary Process:	Job Role:	Supervises:
Operational	3-39-554-641-3	Enable – Regulatory (Individual) Compliance	Manager - Occupational Safety	-

Contributions	Responsible for compliance to the health and safety guidelines for the organisation. Promoting a safe and healthy working environment in the organisation. Responsible to reduce the number of work-related accidents and diseases in the organisation. Responsible to provide equitable compensation benefits to those who may get injured in work-related accidents or contract occupational diseases. Ensures the surveillance of the working environment
Technical Competency(ies):	3.9 Facility design

3.9 Facility design

- Provide inputs to the design and layout of the size and configuration of the facility/building.
- Describe the flow of product through the facility to making sure it is achievable with the selection of material handling equipment
- Achieve the balance of the health and safety requirements of the facility with its performance requirements

Personal/Management Competency(ies):	5.5 Manage and plan projects<br>5.5.4 Direct/participate in teamwork<br>6.1 Demonstrate generic skills (e.g., literacy, numeracy, technology)<br>6.1.1 Exhibit high understanding of literacy and numeracy<br>6.2 Demonstrate strong communication skills<br>6.2.1 Practice cultural awareness<br>6.4 Exhibit professional and ethical values<br>6.4.1 Demonstrate integrity

5.5 Manage and plan projects

5.5.4 Direct/participate in teamwork

- Describe the process of working with team members within their area of authority to establish achievable goals and strategies that are consistent with the objectives established for the organisation as a whole
- Describe an organisational chart that shows the lines of reporting and responsibility among staff
- Actively contribute a perspective and make a positive contribution to team-based problem solving and decision making

- Provide feedback, encouragement and support to team members for progressing strategic goals
- Define team performance in relation to the organisation's mission and goals
- Encourage teams to think for themselves and resolve problems
- Encourage and harness diversity within the team to boost team effectiveness
- Encourage and support the team to work through its stages of development and perform well
- Describe roles and responsibilities in relation to employees' expertise and the expectations of collaborating team members
- Describe where their position fits in the structure (organisation, SC, healthcare sector) , the responsibilities their position entails and the accountabilities linked to the position

6.1 Demonstrate generic skills (e.g., literacy, numeracy, technology)

6.1.1 Exhibit high understanding of literacy and numeracy

- Speak clearly and confidently, organize information in a logical manner, and consider voice tone and pace
- Respond to verbal messages and other cues (active listening)
- Express ideas and opinions clearly in written and verbal form
- Communicate information accurately, concisely and confidently in writing and verbally
- Practice a level of mathematics suitable to the job held
- Describe visual presentation techniques, including charting, histograms and flow sheets
- Read and comprehend at a level necessary to properly complete duties of the position
- Interpret written information in documents, such as reports, SOPs, LMIS forms, graphs, calendars, schedules, notices and directions
- Pay attention to detail and identify the main ideas, detect inconsistencies and identify missing information in documents
- Apply computer skills — such as using Word, Excel, PowerPoint and the internet — suitable to the job level
- Describe the process of analysing and validating KPIs
- Participate in the evaluation of activities that act as standards against which the KPIs are measured
- Apply the process of performing critical self-review on work before submission

6.2 Demonstrate strong communication skills

- Be truthful and trustworthy, and supply accurate information at all times
- Work as part of a workplace team
- Describe vocabulary and communication style and form for both written and verbal communication that is appropriate for the situation, audience and material being communicated (e.g., avoid unnecessary jargon, clearly explain medical and SCM terminology)
- Encourage open communication within constraints of confidentiality

» Organize communications about logistics
» Encourage participation in meetings, expressing one’s opinions, being aware of others’ needs, and being appropriately assertive when required
» Apply perspective into the point of view of others, understanding their needs and goals
» Encourage building trust, rapport and credibility with others
» Anticipate the communication needs and concerns of others and respond to them
» Avoid conflict between work and personal interests
» Encourage meaningful two-way communication
» Influence others by persuasively presenting thoughts and ideas
» Apply assertiveness skills to deal with unreasonable requests and/or refusals that would compromise practice or consumer care
» Maintain a positive, supportive and appreciative attitude
» Apply the ability to balance the organisation’s needs and the customer’s needs
» Use self-control by maintaining composure and keeping emotions in check, even in difficult situations; deal calmly and effectively with stressful situations
» Encourage the expressing of opinions and providing information in written and/or verbal form in a manner that does not elicit concern, anger or other adverse response
» Apply the use of a systematic process for following up that demonstrates written reports have been received and understood
» Describe the means by which responses to input to the work environment are monitored
» Seek practical ways to overcome barriers to communication
» Identify language representation (e.g., interpreters) at meetings when appropriate
» Tackle difficult situations and resolve disputes between staff
» Give both positive and negative feedback sensitively
» Maintain and improve communication
» Apply the process of giving and requesting feedback frequently
» Apply the ability to communicate effectively with shop stewards and union representatives

6.2.1 Practice cultural awareness

» Describe cultural awareness and sensitivity, treating all people with fairness, respect and dignity
» Describe anti-discriminatory practices in the organisation, including HR and disciplinary procedures
» Establish cultural sensitivity, equality and fairness at all levels of the organisation
» Challenge discriminatory behaviour directly and sensitively
» Act in a non-discriminatory way toward individuals and groups
» Integrate cultural awareness in learning and development approaches
» Avoid stereotypical responses by examining one’s own behaviour and bias

» Manage cultural diversity in teams and make the most of differences

» Describe strategies and/or resources for communicating effectively with people from different cultural backgrounds

» Elicit information relating to values, beliefs and cultural backgrounds of consumers that may influence the way professional services are provided

6.4 Exhibit professional and ethical values

6.4.1 Demonstrate integrity

» Accept responsibility for one's own work tasks and performance

» Work within a framework of clearly understood humanitarian values and ethics

» Stand by decisions and hold others accountable when necessary

» Does not abuse one's own power or position

» Manage team members to ensure they do not abuse their power or position

» Identify when individuals or the organisation is straying from organisation goals, and challenge them to uphold ethics

» Resist undue political pressure in decision making

» Support staff in maintaining ethical stances

» Make time in team for ethical inquiry and reflection

» Show consistency between expressed principles and behaviour

» Provide input to ensure that principles, values and ethics are embedded in policy

» Act without consideration of personal gain

» Promote transparency in decision making structures and processes

» Provide input on ensuring programmes are acting with integrity, and recognising the impact of not doing so

» Recognise one's own limitations and act upon them

» Apply all standard operating procedures

» Work in a safe and legal way

» Demonstrate respect, dignity and consideration for consumers

» Discuss the impact of a no-blame culture on reporting and preventing recurrence of incidents

» Describe requests of colleagues that might be regarded as unreasonable

» Communicate (verbally and by example) expectations of the desired standards and approaches to be adopted

» Describe roles and responsibilities in terms of the position statement/duty statement of the position held

» Demonstrate punctuality

» Explain the obligation to apply professional care and expertise to deliver high quality of services

» Demonstrate care and attention to detail in undertaking work activities

» Describe appropriate attire and presentation for the role and situation

» Recognise and take responsibility for emotions

» Describe an environment in which others can talk and act without fear of repercussion

» Seek to keep commitments and not let people down

» Be trusting and cooperative when working alongside others

» Take on various responsibilities within the department as the need arises

Key Performance Indicators:

» Safety statics - Number of cases due to human error ranked by seriousness this period versus previous periods. A graphical representation would be indicated.

» Safety statics - Number of cases due to faulty equipment, ranked by seriousness of injury this period versus previous periods. A graphical representation would be indicated.

» Audit of safety equipment results this period versus previous periods

» Audit of Occupational safety signage and posters. This period versus previous periods

» Occupational safety educational awareness programmes delivered. Actual this period versus Planned this period

» Root cause analysis reports per reported incident. This period versus Last Periods

i

Training:	Qualifications Available:	Certifications Available:
» SHE compliance training<br>» Reporting standards training		» APICS Principles of operations management series certificate<br>» Logistics and Supply Chain Management MIT<br>» SCPro™ Certification<br>» Occupational Certificate: Maintenance Planner<br>» Occupational Certificate: Compliance Officer

SC-focused Organisation

Strategic
Lead – Network Planning

Organisation

SC-focused Organisation

Competency Level:	Serial Number:	Primary Process:	Job Role:	Supervises:
Strategic	5-51-32-532-4	Enable – SC Networking	Lead – Network Planning	Officer – Network Analyst

Contributions	Design and Maintenance of the Supply Chain Network. Determination of least cost routes and material locations.
Technical Competency(ies):	3.2 Supply commodities to facilities

3.2 Supply commodities to facilities

- » Determine the sources of distribution demand (customers)
- » Demonstrate the use of order policies for planned order generation
- » Calculate re-supply quantities using a variety of inventory methods, including visual review, two bin, periodic review, order point and just in time
- » Evaluate the process of preparing and using order schedules
- » Evaluate safety stock calculations
- » Analyse the use of economic order quantity (EOQ) principles in ordering
- » Evaluate the effectivity of using pull and push inventory control systems
- » Determine a regular, rather than urgent, order culture
- » Determine order-filling priorities in relation to delivery opportunities and urgency
- » Analyse the parameters under which one must screen orders (e.g. modify order quantities on the basis of available stock, impact on service delivery, distance of facility from hospital)
- » Evaluate assembling, checking, picking and packing of orders
- » Demonstrate making and using dispatch lists and invoices
- » Balance supply with demand, considering both lead time and demand variability created by supply patterns not matching demand patterns
- » Analyse information on the storage locations and facilities
- » Measure customer satisfaction and develop loyal customers by using performance metrics taken from the customer perspective, with

criteria such as on-time delivery, perception of quality, complaints and length of wait times

Personal/Management Competency(ies):	5.1 Design and implement supply chain system and strategies<br>5.3 Implement risk management and monitoring and evaluation activities for the supply chain<br>5.3.2 Implement risk management activities<br>5.5 Manage and plan projects<br>5.5.1 Develop and direct project plans<br>5.5.2 Execute strategic decision making<br>5.5.3 Manage partnerships<br>5.5.4 Direct/participate in teamwork<br>6.5 Prove leadership abilities<br>6.5.1 Demonstrate resilience and ability to manage stress<br>6.6 Abide by rules / laws / legislation
5.1 Design and implement supply chain system and strategies	» Demonstrate a responsive, agile and efficient supply chain that has the ability to meet the changing needs of customers and deliver high-quality products with short lead times at low cost<br>» Demonstrate the interrelationship of organisations, people, technology, activities, information, and resources involved in moving a product from supplier to customer<br>» Demonstrate the management activities carried out in the course of running an organisation, including controlling, leading, monitoring, adjusting, organising and planning<br>» Determine the characteristics unique to public health, demonstrate awareness of the factors that could contribute to or hinder the delivery of logistics services<br>» Demonstrate awareness of emerging concepts and principles in public health logistics; adapt current practices to incorporate new developments in the field<br>» Determine which type of inventory control system will be most effective for a particular programme or country<br>» Demonstrate the difference between continuous and period review systems of inventory control<br>» Determine and demonstrate a formal logistics strategy and plan
5.3 Implement risk management and monitoring and evaluation activities for the supply chain	
5.3.2 Implement risk management activities	» Determine risks affecting supply, transformation, delivery and customer demand<br>» Determine what constitutes an unsafe working conditions and how to take prompt, corrective action; stay alert to and take preventive action against hazards and threats; recommend measures to protect

employees from hazardous working conditions; follow protocol for reporting safety violations; and investigate accidents to determine how to prevent them in the future

» Demonstrate training programmes/drills in which personnel participate (e.g., fire and armed hold-up drills, safe manual handling practices, use of mandatory safety clothing)

» Analyse systems of review (e.g., audit) to check compliance with policies and procedures intended to maintain workplace safety (e.g., manual handling techniques, correct use of protective clothing)

» Determine the process of ensuring equipment and tools are operating to prescribed standards

» Determine key security systems for the workplace (e.g., for cash, narcotics and other controlled substances, investigational drugs, consumer records, entry and exit points) and levels of access and/or authority applicable to each

» Demonstrate the prevention strategies adopted (e.g., protocols, security barriers, fixed or personal duress alarms)

» Evaluate documented critical incidents

» Engage in contingency planning and managing operational continuity

» Ensure that there is an understanding of what a safe working environment is and engender a shared commitment from all personnel to creating one

» Demonstrate knowledge of relevant safety and health laws and regulations; comply with safety codes, standards and guidelines; adhere to worksite safety programmes; understand emergency response plans when they exist

» Demonstrate knowledge of how to handle hazardous commodities and first aid procedures

5.5 Manage and plan projects

5.5.1 Develop and direct project plans

» Evaluate obstacles and critical events for ensuring the six rights of logistics management (right quantity, product, condition, place, time and cost) and develop contingency plans to address them; monitor progress and take necessary corrective action when needed

» Determine an operational plan that is consistent with the strategic plan, links specific goals and strategies, and identifies the performance indicators to be used to measure achievements

» Demonstrate the use of information gathering techniques from stakeholders, analysing situations and identifying implications to make correct decisions

» Review the questions, “Where are we going?” and “How are we going to get there?” and create a specific and purposeful path to achieve this.

» Demonstrate the process reference model to managing supply chain processes (i.e., integrating business process re-engineering, benchmarking and process measurement)

» Analyse the current state of a process and derive the desired future state

- Demonstrate the process of selecting and applying tools or technological solutions to frequently encountered problems
- Analyse the process of providing staff with the necessary tools to carry out plans
- Demonstrate the ability to monitor progress and make changes as required
- Convene meetings for planning, organisation and monitoring logistics activities
- Ensure staff are aware that they are accountable for achieving the desired results
- Determine how planning, organising and managing resources will bring about the successful completion of projects
- Determine the key features of a successful change management strategy

5.5.2 Execute strategic decision making

- Determine how one can make decisions regarding one's own workload and area of responsibility
- Determine the process of prioritizing competing tasks and perform them quickly and efficiently according to their importance; finding new ways of organizing or planning work to accomplish it more efficiently
- Evaluate the proper time and space for consultation around decisions being made.
- Demonstrate the process of considering the input of resources including staff needed
- Analyse assumptions against facts
- Determine the key issues in a complex situation and come to the heart of the problem quickly
- Evaluate relevant information before making decisions
- Provide input to strategic decisions in the interest of the organisation's goals
- Evaluate when to analyse, evaluate and execute tough decisions when necessary
- Analyse the impact of decisions on others' work or team goals
- Communicate decisions and ensure they are incorporated into policies and processes
- Demonstrate inclusive and consultative strategic planning to establish strategic objectives
- Evaluate emerging trends and practices
- Analyse the macro and long-term consequences of decisions

5.5.3 Manage partnerships

- Demonstrate the concepts of partnership working
- Determine the process of supporting implementation of partnership programmes
- Analyse the process of experimenting with and trialling new ideas with partners
- Analyse partnerships to deliver programme and increase impact

- Analyse innovation and creativity in partnership work
- Communicate key information with partner members
- Involve and value partners in all aspects of programming
- Analyse problems with supply chain relationships
- Demonstrate the process of communicating effectively with nurses, doctors and other members of the healthcare team
- Analyse the process of responding to consumer complaints or comments about services and/or advice received
- Analyse collaboration and consultation across the sector and encourage the team to work with others to gain market insight
- Encourage input from other key actors in the humanitarian sector and develop relationships with a cross-section of actors
- Analyse the process of meeting the reporting requirements of vertical programmes
- Analyse the structure of the health system at a national level and explain this to others
- Analyse the roles of other members of the healthcare team (including with consumers) in a way that engenders understanding and confidence in the team and its members
- Analyse the structure of the organisation, environment and/or service in which they work
- Demonstrate how vertical programmes work within the health system
- Evaluate the communication network established to achieve work outcomes
- Demonstrate knowledge of the structure of the health system at the provincial/regional level and explain this to others
- Analyse the roles and functions of government agencies in regulating and supporting supply chain organisations
- Demonstrate knowledge of change management (e.g. PtD Theory of Change)

5.5.4 Direct/participate in teamwork

- Work with team members within their area of authority to establish achievable goals and strategies that are consistent with the objectives established for the organisation as a whole
- Evaluate an organisational chart that shows the lines of reporting and responsibility among staff
- Determine a culture of actively contributing a perspective and making a positive contribution to team-based problem solving and decision making
- Determine a culture of providing feedback, encouragement and support to team members for progressing strategic goals
- Monitor team performance in relation to the organisation’s mission and goals
- Enable the creation of a culture of teams thinking for themselves and resolving problems
- Demonstrate the process of encouraging and harnessing diversity within the team to boost team effectiveness

	» Demonstrate the process of encouraging and supporting the team to work through its stages of development and perform well<br>» Determine roles and responsibilities in relation to employees' expertise and the expectations of collaborating team members<br>» Demonstrate where their position fits in the structure (organisation, SC, healthcare sector) , the responsibilities their position entails and the accountabilities linked to the position
6.5 Prove leadership abilities	» Analyse input to meetings and programme development<br>» Look for future trends and issues, and assist the organisation in meeting the challenges<br>» Contribute to a collaborative working environment<br>» Demonstrate a drive for change and improvement develops opportunities for the organisation and sector<br>» Demonstrate channelling energy and ideas toward resolving issues<br>» Demonstrate the practise of communicating with influence<br>» Demonstrate showing courage to take an unpopular stance when needed<br>» Demonstrate steering and implementing change organisationally<br>» Demonstrate anticipating and resolving conflict<br>» Demonstrate showing initiative in working methods<br>» Exemplify personal drive and integrity<br>» Demonstrate serving as a role model for others to follow<br>» Demonstrate leadership and practice management skills, initiative and efficiency<br>» Demonstrate inspiring others through professional excellence, innovation and communication<br>» Determine situations in which a change in leadership style would be warranted (e.g., directive versus consultative style in the event of a crisis)
6.5.1 Demonstrate resilience and ability to manage stress	» Demonstrate identifying symptoms of stress and take steps to reduce stress<br>» Demonstrate seeing the bigger picture and help others to<br>» Demonstrate recovering quickly from setbacks<br>» Demonstrate drawing on previous experience and support mechanisms to reduce the impact of stress on self and others<br>» Determine a working environment that aims to minimise pressure and stress<br>» Demonstrate coping well under pressure, particularly in difficult environments<br>» Demonstrate acting as a role model for others and display courage under difficult circumstances<br>» Demonstrate recognising the limitations of staff and taking action to limit their exposure to harm when needed

	» Demonstrate making use of personal support mechanisms<br>» Demonstrate helping others identify personal support mechanisms<br>» Determine organisational policy to support self-care in agencies
6.6 Abide by rules / laws / legislation	» Demonstrate compliance with the legislation that covers the practice of public health supply chains and healthcare, and describe its purpose<br>» Analyse requirements of professional codes, guidelines and standards adopted as part of the legislative framework<br>» Demonstrate keeping up to date with changes in legal instruments, as informed by national-level managers<br>» Demonstrate compliance with international regulations in decision making for the distribution system, including customs regulations on import and export requirements (e.g., trade tariffs and duties on imported goods) and security regulations (e.g., 2007 SAFE Ports Act, NAFTA or European Union trade agreements)<br>» Demonstrate applying and understanding regulatory affairs and the key aspects of pharmaceutical registration and legislation<br>» Demonstrate knowledge of the principles of business economics and intellectual property rights, including the basics of patent interpretation<br>» Demonstrate knowledge of applicable procurement law and the practical effects of a contract's terms and conditions<br>» Evaluate current industry and government regulations governing sustainability<br>» Demonstrate a working knowledge of key hazardous materials handling laws<br>» Determine policies and procedures relating to workplace safety that are consistent with agreed or recognised standards<br>» Analyse professional standards and conventions, as well as workplace policies and procedures, for preparing pharmaceutical products<br>» Demonstrate all standard operating procedures<br>» Evaluate the process of complying with data privacy and data protection laws
Basal Technology Competency(ies):	7.1 Data Science<br>7.4 Temperature and monitoring<br>7.6 ERP includes function of LMIS<br>7.12 Basic Office Skills<br>7.13 Have a command of technology
7.1 Data Science	» Analyse end to end MIS technology components that are inherent in advanced MIS systems and explore how these may be relevant in different supply chain contexts<br>» Determine emerging technological approaches to data visualization and analysis

	» Analyse how data science can be applied within supply chain<br>» Demonstrate the ability to interact, provide guidance for data (dashboard) consolidation in various levels and use the tech tools<br>» Support movement of data into electronic form at lowest/remote SC level<br>» Demonstrate the ability to work with relational database
7.4 Temperature and monitoring	» Analyse the current technology approaches for temperature monitoring and sensors<br>» Evaluate what is required to implement this technology in a country context<br>» Evaluate infrastructure requirement for heat-sensitive items at peripheral ‘lower level’ of SC depending on volume and nature of items to handle;<br>» Analyse regular preventive maintenance and calibration of cold-chain equipment with biomedical engineers or logisticians<br>» Evaluate the policy for temperature monitoring tools during transportation and storage<br>» Demonstrate the steps involved in health logistic management cycle: selection-order-delivery-reception-storage-distribution-inventory<br>» Determine hardware qualification and system validation of temperature monitoring<br>» Evaluate mass data management of temperature monitoring
7.6 ERP includes function of LMIS	» Evaluate a Master Data Management System<br>» Demonstrate an understanding of the critical components of an ERP system<br>» Demonstrate an understanding of the meaning of common document types found within an ERP/LMIS<br>» Demonstrate an understanding of the logic of data visibility within an ERP<br>» Demonstrate the importance of data integrity, record management and information security
7.12 Basic Office Skills	» Demonstrate a good understanding of common presentation authoring packages<br>» Demonstrate a good understanding of common spreadsheet authoring packages<br>» Demonstrate a good understanding of common document authoring packages<br>» Demonstrate a good understanding of common email authoring packages<br>» Demonstrate a good understanding of common instant messaging packages<br>» Demonstrate a good understanding of common video conferencing authoring packages

7.13 Have a command of technology	» Demonstrate the use of technology suitable to the job held<br>» Monitor new developments and technologies in the sector<br>» Experiment with new technologies and recognising potential benefits for the sector<br>» Ensure that resources and support are provided across the organisation to enable colleagues to make the best use of available technology<br>» Determine what the organisations' strategy is for technology use<br>» Determine how to minimise environmental damage through technology use<br>» Analyse the use of technology to maximise effectiveness and efficiency<br>» Analyse the use of field-based technology (e.g., radio, general packet radio service (GPRS), satellite phone)

Advanced Technology Competency(ies):	7.7 Automation
7.7 Automation	» Evaluate automation as a value-add capability for relevant global health use cases

Key Performance Indicators:

- Availability - Stock out rate - Number of Items with failed deliveries due to unavailability and or Number of Items whose availability is bound to be compromised because Stock on Hand less Planned Demand in the planning horizon is less than Stock on Hand + Pipeline stock in the planning horizon.
- Availability - Stock levels - Stock on hand versus Planned Stock on Hand
- Inventory days of supply versus Planned Inventory as stipulated in the Operations Plan
- Manage Integrated Supply Chain Transportation Cycle Time
- Manage Integrated Supply Chain Transportation Cycle Time - Actual Transportation Cycle time versus Plan Transportation Cycle Time
- Manage supply chain network inbound cycle time - Actual Network Inbound Cycle Time vs Planned Network Inbound Cycle Time
- Supply adequacy - Actual stock holding plus Pipeline Stock versus Demand Plan
- Total SCM Costs vs Planned Total SCM Planned Costs

Training:	Qualifications Available:	Certifications Available:
» Benchmarking<br>» Performance Management<br>» Optimization<br>» Supply Chain Performance Measurements<br>» Outsourcing	» Postgraduate Diploma in International Shipping<br>» (CILT) International Advanced Diploma in Logistics & Transport	» SPSM3 TM (Enterprise-Wide Procurement Influence)<br>» APICS Certified in production and inventory management certificate<br>» Occupational Certificate: Health Information Manager

» Prioritization<br>» Asset Management<br>» Capacity Planning/Management<br>» Enterprise Business Process<br>» Logistics network modelling<br>» Supply Chain Management		» APICS Certified in logistics transportation and distribution certificate

Lead – Data Intelligence and Analytics

Organisation:

SC-focused Organisation

Competency Level:	Serial Number:	Primary Process:	Job Role:	Supervises:
Strategic	7-79-76-551-4	Enable – Data Information	Lead – Data Intelligence and Analytics	Manager – Data and Use

Contributions	Ensuring a data stream from source transactions, building analytic models and mining data available in the Supply Chain transaction records with a view to build predictability models.
Personal/Management Competency(ies):	5.5 Manage and plan projects<br>5.5.1 Develop and direct project plans<br>5.5.2 Execute strategic decision making<br>6.5 Prove leadership abilities<br>6.5.1 Demonstrate resilience and ability to manage stress<br>6.6 Abide by rules / laws / legislation

5.5 Manage and plan projects

5.5.1 Develop and direct project plans

- » Evaluate obstacles and critical events for ensuring the six rights of logistics management (right quantity, product, condition, place, time and cost) and develop contingency plans to address them; monitor progress and take necessary corrective action when needed
- » Determine an operational plan that is consistent with the strategic plan, links specific goals and strategies, and identifies the performance indicators to be used to measure achievements
- » Demonstrate the use of information gathering techniques from stakeholders, analysing situations and identifying implications to make correct decisions
- » Review the questions, "Where are we going?" and "How are we going to get there?" and create a specific and purposeful path to achieve this.
- » Demonstrate the process reference model to managing supply chain processes (i.e., integrating business process re-engineering, benchmarking and process measurement)
- » Analyse the current state of a process and derive the desired future state
- » Demonstrate the process of selecting and applying tools or technological solutions to frequently encountered problems
- » Analyse the process of providing staff with the necessary tools to carry out plans
- » Demonstrate the ability to monitor progress and make changes as required
- » Convene meetings for planning, organisation and monitoring logistics activities
- » Ensure staff are aware that they are accountable for achieving the desired results

	» Determine how planning, organising and managing resources will bring about the successful completion of projects<br>» Determine the key features of a successful change management strategy
5.5.2 Execute strategic decision making	» Determine how one can make decisions regarding one's own workload and area of responsibility<br>» Determine the process of prioritizing competing tasks and perform them quickly and efficiently according to their importance; finding new ways of organizing or planning work to accomplish it more efficiently<br>» Evaluate the proper time and space for consultation around decisions being made.<br>» Demonstrate the process of considering the input of resources including staff needed<br>» Analyse assumptions against facts<br>» Determine the key issues in a complex situation and come to the heart of the problem quickly<br>» Evaluate relevant information before making decisions<br>» Provide input to strategic decisions in the interest of the organisation's goals<br>» Evaluate when to analyse, evaluate and execute tough decisions when necessary<br>» Analyse the impact of decisions on others' work or team goals<br>» Communicate decisions and ensure they are incorporated into policies and processes<br>» Demonstrate inclusive and consultative strategic planning to establish strategic objectives<br>» Evaluate emerging trends and practices<br>» Analyse the macro and long-term consequences of decisions
6.5 Prove leadership abilities	» Analyse input to meetings and programme development<br>» Look for future trends and issues, and assist the organisation in meeting the challenges<br>» Contribute to a collaborative working environment<br>» Demonstrate a drive for change and improvement develops opportunities for the organisation and sector<br>» Demonstrate channelling energy and ideas toward resolving issues<br>» Demonstrate the practise of communicating with influence<br>» Demonstrate showing courage to take an unpopular stance when needed<br>» Demonstrate steering and implementing change organisationally<br>» Demonstrate anticipating and resolving conflict<br>» Demonstrate showing initiative in working methods<br>» Exemplify personal drive and integrity<br>» Demonstrate serving as a role model for others to follow<br>» Demonstrate leadership and practice management skills, initiative and efficiency<br>» Demonstrate inspiring others through professional excellence, innovation and communication

	» Determine situations in which a change in leadership style would be warranted (e.g., directive versus consultative style in the event of a crisis)
6.5.1 Demonstrate resilience and ability to manage stress	» Demonstrate identifying symptoms of stress and take steps to reduce stress<br>» Demonstrate seeing the bigger picture and help others to<br>» Demonstrate recovering quickly from setbacks<br>» Demonstrate drawing on previous experience and support mechanisms to reduce the impact of stress on self and others<br>» Determine a working environment that aims to minimise pressure and stress<br>» Demonstrate coping well under pressure, particularly in difficult environments<br>» Demonstrate acting as a role model for others and display courage under difficult circumstances<br>» Demonstrate recognising the limitations of staff and taking action to limit their exposure to harm when needed<br>» Demonstrate making use of personal support mechanisms<br>» Demonstrate helping others identify personal support mechanisms<br>» Determine organisational policy to support self-care in agencies
6.6 Abide by rules / laws / legislation	» Demonstrate compliance with the legislation that covers the practice of public health supply chains and healthcare, and describe its purpose<br>» Analyse requirements of professional codes, guidelines and standards adopted as part of the legislative framework<br>» Demonstrate keeping up to date with changes in legal instruments, as informed by national-level managers<br>» Demonstrate compliance with international regulations in decision making for the distribution system, including customs regulations on import and export requirements (e.g., trade tariffs and duties on imported goods) and security regulations (e.g., 2007 SAFE Ports Act, NAFTA or European Union trade agreements)<br>» Demonstrate applying and understanding regulatory affairs and the key aspects of pharmaceutical registration and legislation<br>» Demonstrate knowledge of the principles of business economics and intellectual property rights, including the basics of patent interpretation<br>» Demonstrate knowledge of applicable procurement law and the practical effects of a contract’s terms and conditions<br>» Evaluate current industry and government regulations governing sustainability<br>» Demonstrate a working knowledge of key hazardous materials handling laws<br>» Determine policies and procedures relating to workplace safety that are consistent with agreed or recognised standards<br>» Analyse professional standards and conventions, as well as workplace policies and procedures, for preparing pharmaceutical products<br>» Demonstrate all standard operating procedures<br>» Evaluate the process of complying with data privacy and data protection laws
Basal Technology Competency(ies):	7.1 Data Science

	7.6 ERP includes function of LMIS<br>7.13 Have a command of technology
7.1 Data Science	» Analyse end to end MIS technology components that are inherent in advanced MIS systems and explore how these may be relevant in different supply chain contexts<br>» Determine emerging technological approaches to data visualization and analysis<br>» Analyse how data science can be applied within supply chain<br>» Demonstrate the ability to interact, provide guidance for data (dashboard) consolidation in various levels and use the tech tools<br>» Support movement of data into electronic form at lowest/remote SC level<br>» Demonstrate the ability to work with relational database
7.6 ERP includes function of LMIS	» Evaluate a Master Data Management System<br>» Demonstrate an understanding of the critical components of an ERP system<br>» Demonstrate an understanding of the meaning of common document types found within an ERP/LMIS<br>» Demonstrate an understanding of the logic of data visibility within an ERP<br>» Demonstrate the importance of data integrity, record management and information security
7.13 Have a command of technology	» Demonstrate the use of technology suitable to the job held<br>» Monitor new developments and technologies in the sector<br>» Experiment with new technologies and recognising potential benefits for the sector<br>» Ensure that resources and support are provided across the organisation to enable colleagues to make the best use of available technology<br>» Determine what the organisations' strategy is for technology use<br>» Determine how to minimise environmental damage through technology use<br>» Analyse the use of technology to maximise effectiveness and efficiency<br>» Analyse the use of field-based technology (e.g., radio, general packet radio service (GPRS), satellite phone)

Key Performance Indicators:

» Assess Delivery Performance Cycle Time - Actual Time from assessed requirement to time delivered to end-user compared to committed Delivery Cycle Time as per Supply Chain strategy

» Assess Supplier Performance Cycle Time - Actual Supplier performance Cycle time versus Supplier commitment cycle time

» Inventory accuracy - Cycle count results

» Transportation lead time accuracy - Actual Transportation lead time versus Transportation promised lead time.

» Transport efficiency - Total cost of transport versus planned cost of transport by mode

» In-bound cost of acquisition - Actual total cost of acquisition versus Total Planned cost of acquisition including landed costs

» Manage performance cycle time

» Performance Report - Actual versus Plan reports for all metrics

Training:	Qualifications Available:	Certifications Available:
» IT Management<br>» Supply chain management<br>» Report writing<br>» IT project Management<br>» IT System Implementation	» Bachelor of Computer Technology<br>» Bachelor of Engineering Science: Information Technology and Computer Systems<br>» Bachelor of Computing<br>» Bachelor of Supply Chain Management<br>» Bachelor of Mathematics<br>» Bachelor of Data Science<br>» Bachelor of in Pharmaceutical Sciences specialising in epidemiology	» CIPS Level 5 Advanced Diploma in Procurement and Supply<br>» APICS Certified in logistics transportation and distribution certificate<br>» SPSM Level 4 (External Procurement Influence)

Lead – Process Improvement and SC Adaptability

Organisation:

SC-focused Organisation

Competency Level:	Serial Number:	Primary Process:	Job Role:	Supervises:
Strategic	5-531-79-4	Enable – Performance	Lead – Process Improvement and SC Adaptability	-

Contributions	In conjunction with Operations evaluate the performance of the Supply Chain investigate deviations, establish the root causes of the deviations and suggest interventions to eliminate the source of the deviations. Be able to do cost benefit analysis so that improvement suggestions have a positive ROI.
Personal/Management Competency(ies):	5.3 Implement risk management and monitoring and evaluation activities for the supply chain<br>5.3.1 Ensure monitoring and evaluation activities are completed<br>5.5 Manage and plan projects<br>5.5.1 Develop and direct project plans<br>5.5.2 Execute strategic decision making<br>5.6 Manage financial activities<br>6.1 Demonstrate generic skills (e.g., literacy, numeracy, technology)<br>6.1.1 Exhibit high understanding of literacy and numeracy<br>6.2 Demonstrate strong communication skills<br>6.2.1 Practice cultural awareness

5.3 Implement risk management and monitoring and evaluation activities for the supply chain

5.3.1 Ensure monitoring and evaluation activities are completed	» Perform periodic evaluations of pertinent information — such as problem symptoms from knowledgeable sources, carrying these through to the problems, potential causes and root causes of the problem<br>» Determine the process of quantifying the operational performance of similar companies and establish internal targets based on best-in-class results<br>» Analyse key performance measurements and continuous process improvement initiatives to improve process quality on a continual basis<br>» Analyse the results of the analyses provided<br>» Determine the process of controlling and checking errors, taking corrective action so deviation from standards are minimised and the organisation's goals are achieved

» Analyse the use of appropriate technological developments to improve the system

» Demonstrate that continuous process improvement is an accepted way of organisational life

» Demonstrate ways of eliminating unnecessary steps in system design

» Analyse processes that strive to eliminate waste

» Review processes to encourage sustainability (e.g., reducing carbon footprint, establishing a paperless office, using renewable energy)

» Demonstrate the systematic approach used to close process or system performance gaps through streamlining and cycle time reduction, and identify and eliminate causes of quality below specifications, process variation and non-value-adding activities

» Determine the relationship between technology and process functionality

» Determine how policy, guidelines and regulatory reforms impact SC performance

5.5 Manage and plan projects

5.5.1 Develop and direct project plans

» Evaluate obstacles and critical events for ensuring the six rights of logistics management (right quantity, product, condition, place, time and cost) and develop contingency plans to address them; monitor progress and take necessary corrective action when needed

» Determine an operational plan that is consistent with the strategic plan, links specific goals and strategies, and identifies the performance indicators to be used to measure achievements

» Demonstrate the use of information gathering techniques from stakeholders, analysing situations and identifying implications to make correct decisions

» Review the questions, “Where are we going?” and “How are we going to get there?” and create a specific and purposeful path to achieve this.

» Demonstrate the process reference model to managing supply chain processes (i.e., integrating business process re-engineering, benchmarking and process measurement)

» Analyse the current state of a process and derive the desired future state

» Demonstrate the process of selecting and applying tools or technological solutions to frequently encountered problems

» Analyse the process of providing staff with the necessary tools to carry out plans

» Demonstrate the ability to monitor progress and make changes as required

» Convene meetings for planning, organisation and monitoring logistics activities

» Ensure staff are aware that they are accountable for achieving the desired results

» Determine how planning, organising and managing resources will bring about the successful completion of projects

» Determine the key features of a successful change management strategy

5.5.2 Execute strategic decision making	» Determine how one can make decisions regarding one's own workload and area of responsibility<br>» Determine the process of prioritizing competing tasks and perform them quickly and efficiently according to their importance; finding new ways of organizing or planning work to accomplish it more efficiently<br>» Evaluate the proper time and space for consultation around decisions being made.<br>» Demonstrate the process of considering the input of resources including staff needed<br>» Analyse assumptions against facts<br>» Determine the key issues in a complex situation and come to the heart of the problem quickly<br>» Evaluate relevant information before making decisions<br>» Provide input to strategic decisions in the interest of the organisation's goals<br>» Evaluate when to analyse, evaluate and execute tough decisions when necessary<br>» Analyse the impact of decisions on others' work or team goals<br>» Communicate decisions and ensure they are incorporated into policies and processes<br>» Demonstrate inclusive and consultative strategic planning to establish strategic objectives<br>» Evaluate emerging trends and practices<br>» Analyse the macro and long-term consequences of decisions
5.6 Manage financial activities	» Determine rules for managing all resources with care, applying budgetary principles<br>» Take ownership for meeting budgets and donor requirements<br>» Analyse budgets (national, regional, etc.) as necessary for work (e.g., wages budget, touring budget, stationery budget, project budgets for using NGO funds)<br>» Produce timely and clear financial reports for funders and donors<br>» Keep records following government budgeting, accounting and financial practices<br>» Maintain an overview of multiple budgets from multiple sources<br>» Seek and use information on financial funding requirements<br>» Analyse the process of ensuring timely decision making with regard to financial shortfalls<br>» Analyse budget tracking for logistics activities according to established procedures<br>» Analyse financial statements and explain components of a balance sheet and income statement<br>» Create interactive decision support models that allow the development of multiple scenarios and demonstrate the sensitivity of multiple independent variables<br>» Determine the success or failure of a business using financial accounting

	» Evaluate the calculations of the total system cost of delivering a product or service to the customer<br>» Analyse the key considerations for developing a business plan<br>» Evaluate the structure against which business performance will be monitored (e.g., cost centres, chart of accounts)<br>» Analyse key performance indicators used to monitor business performance (e.g., turnover, profitability)<br>» Determine strategies for minimising the risk of fraudulent activity (e.g., reconciliation of purchase orders, receipts and payment approvals; dual signatory arrangements for funds transfers)<br>» Evaluate investigative processes and options for confirming the existence of fraudulent activity (e.g., sample audits, forensic accounting services)<br>» Prepare funding applications for trusts and grants<br>» Demonstrate the process of ensuring financial transparency<br>» Evaluate the general monetary value of medicines and equipment<br>» Secure financing and manage budgets to support distribution operations<br>» Demonstrate the ability to translate financial information into SC language<br>» Demonstrate SC finance knowledge
6.1 Demonstrate generic skills (e.g., literacy, numeracy, technology)	
6.1.1 Exhibit high understanding of literacy and numeracy	» Encourage speaking clearly and confidently, organizing information in a logical manner, and considering voice tone and pace<br>» Demonstrate responding to verbal messages and other cues (active listening)<br>» Demonstrate expressing ideas and opinions clearly in written and verbal form<br>» Demonstrate communicating information accurately, concisely and confidently in writing and verbally<br>» Demonstrate a level of mathematics suitable to the job held<br>» Demonstrate knowledge of visual presentation techniques, including charting, histograms and flow sheets<br>» Demonstrate reading and comprehending at a level necessary to properly complete duties of the position<br>» Demonstrate the process of interpreting written information in documents, such as reports, SOPs, LMIS forms, graphs, calendars, schedules, notices and directions<br>» Demonstrate paying attention to detail and identifying the main ideas, detecting inconsistencies and identifying missing information in documents<br>» Demonstrate computer skills — such as using Word, Excel, PowerPoint and the internet — suitable to the job level<br>» Demonstrate the process of analysing and validating KPIs<br>» Participate in the evaluation of activities that act as standards against which the KPIs are measured

	» Demonstrate the process of performing critical self-review on work before submission
6.2 Demonstrate strong communication skills	» Demonstrate being truthful and trustworthy, and supplying accurate information at all times<br>» Demonstrate working as part of a workplace team<br>» Determine vocabulary and communication style and form for both written and verbal communication that is appropriate for the situation, audience and material being communicated (e.g., avoid unnecessary jargon, clearly explain medical and SCM terminology)<br>» Demonstrate open communication within constraints of confidentiality<br>» Analyse communications about logistics<br>» Demonstrate participation in meetings, expressing one’s opinions, being aware of others’ needs, and being appropriately assertive when required<br>» Demonstrate perspective into the point of view of others, understanding their needs and goals<br>» Demonstrate the practise of building trust, rapport and credibility with others<br>» Determine the communication needs and concerns of others and respond to them<br>» Demonstrate avoiding conflict between work and personal interests<br>» Demonstrate the practice of meaningful two-way communication<br>» Demonstrate influence on others by persuasively presenting thoughts and ideas<br>» Demonstrate assertiveness skills to deal with unreasonable requests and/or refusals that would compromise practice or consumer care<br>» Demonstrate a positive, supportive and appreciative attitude<br>» Demonstrate the ability to balance the organisation’s needs and the customer’s needs<br>» Demonstrate self-control by maintaining composure and keeping emotions in check, even in difficult situations; deal calmly and effectively with stressful situations<br>» Demonstrate the expressing of opinions and providing information in written and/or verbal form in a manner that does not elicit concern, anger or other adverse response<br>» Demonstrate the use of a systematic process for following up that demonstrates written reports have been received and understood<br>» Evaluate the means by which responses to input to the work environment are monitored<br>» Seek practical ways to overcome barriers to communication<br>» Ensure language representation (e.g., interpreters) at meetings when appropriate<br>» Demonstrate tackling difficult situations and resolving disputes between staff<br>» Demonstrate giving both positive and negative feedback sensitively<br>» Demonstrate maintaining and improving communication<br>» Demonstrate the process of giving and requesting feedback frequently

	» Demonstrate the ability to communicate effectively with shop stewards and union representatives
6.2.1 Practice cultural awareness	» Demonstrate cultural awareness and sensitivity, treating all people with fairness, respect and dignity<br>» Demonstrate anti-discriminatory practices in the organisation, including HR and disciplinary procedures<br>» Demonstrate cultural sensitivity, equality and fairness at all levels of the organisation<br>» Demonstrate challenging discriminatory behaviour directly and sensitively<br>» Demonstrate acting in a non-discriminatory way toward individuals and groups<br>» Demonstrate the process of integrating cultural awareness in learning and development approaches<br>» Demonstrate avoiding stereotypical responses by examining one's own behaviour and bias<br>» Demonstrate managing cultural diversity in teams and make the most of differences<br>» Demonstrate the use of strategies and/or resources for communicating effectively with people from different cultural backgrounds<br>» Supply information relating to values, beliefs and cultural backgrounds of consumers that may influence the way professional services are provided
Basal Technology Competency(ies):	7.1 Data Science<br>7.5 Planning systems<br>7.6 ERP includes function of LMIS<br>7.12 Basic Office Skills<br>7.13 Have a command of technology
7.1 Data Science	» Analyse end to end MIS technology components that are inherent in advanced MIS systems and explore how these may be relevant in different supply chain contexts<br>» Determine emerging technological approaches to data visualization and analysis<br>» Analyse how data science can be applied within supply chain<br>» Demonstrate the ability to interact, provide guidance for data (dashboard) consolidation in various levels and use the tech tools<br>» Support movement of data into electronic form at lowest/remote SC level<br>» Demonstrate the ability to work with relational database
7.5 Planning systems	» Analyse blockchain and understand the building blocks for planning systems<br>» Evaluate planning systems as a value-add capability for relevant global health use cases

7.6 ERP includes function of LMIS	» Evaluate a Master Data Management System<br>» Demonstrate an understanding of the critical components of an ERP system<br>» Demonstrate an understanding of the meaning of common document types found within an ERP/LMIS<br>» Demonstrate an understanding of the logic of data visibility within an ERP<br>» Demonstrate the importance of data integrity, record management and information security
7.12 Basic Office Skills	» Demonstrate a good understanding of common presentation authoring packages<br>» Demonstrate a good understanding of common spreadsheet authoring packages<br>» Demonstrate a good understanding of common document authoring packages<br>» Demonstrate a good understanding of common email authoring packages<br>» Demonstrate a good understanding of common instant messaging packages<br>» Demonstrate a good understanding of common video conferencing authoring packages
7.13 Have a command of technology	» Demonstrate the use of technology suitable to the job held<br>» Monitor new developments and technologies in the sector<br>» Experiment with new technologies and recognising potential benefits for the sector<br>» Ensure that resources and support are provided across the organisation to enable colleagues to make the best use of available technology<br>» Determine what the organisations' strategy is for technology use<br>» Determine how to minimise environmental damage through technology use<br>» Analyse the use of technology to maximise effectiveness and efficiency<br>» Analyse the use of field-based technology (e.g., radio, general packet radio service (GPRS), satellite phone)
Advanced Technology Competency(ies):	7.2 Blockchain<br>7.7 Automation<br>7.11 Cloud Computing
7.2 Blockchain	» Evaluate blockchain as a value-add capability for relevant global health use cases
7.7 Automation	» Evaluate automation as a value-add capability for relevant global health use cases
7.11 Cloud Computing	» Evaluate cloud computing as a value-add capability for relevant global health use cases

Key Performance Indicators:

- Assess Delivery Performance Cycle Time - Actual Time from assessed requirement to time delivered to end-user compared to committed Delivery Cycle Time as per Supply Chain strategy
- Assess Supplier Performance Cycle Time - Actual Supplier performance Cycle time versus Supplier commitment cycle time
- Inventory accuracy - Cycle count results
- Transportation lead time accuracy - Actual Transportation lead time versus Transportation promised lead time.
- Transport efficiency - Total cost of transport versus planned cost of transport by mode
- In-bound cost of acquisition - Actual total cost of acquisition versus Total Planned cost of acquisition including landed costs
- Performance Report - Actual versus Plan reports for all metrics
- Manage performance cycle time. Actual Supply Chain Performance required versus Actual. This is a roll up metric
- Finance secured and expenses managed in line with supporting distribution operations

Training:	Qualifications Available:	Certifications Available:
» Supply chain Management<br>» Report Writing<br>» Data Management<br>» Total Quality Management (TQM)<br>» Business Rule/Policy Management<br>» Controls and Compliance<br>» Monitoring and Evaluation	» Advanced Diploma in Quality Management<br>» Bachelor of Computer Technology	» SPSM3 TM (Enterprise-Wide Procurement Influence)<br>» Occupational Certificate: Health Information Manager<br>» APICS Certified supply chain professional certificate<br>» SPSM Level 4 (External Procurement Influence)<br>» Lean Six Sigma Certification in Finance black belt

Lead – Planning and Performance

Organisation:

SC-focused Organisation

Competency Level:	Serial Number:	Primary Process:	Job Role:	Supervises:
Strategic	5-51-55-65-5	Enable – Performance	Lead – Planning and Performance	-

Contributions	Responsible for analysis of supply chain performance data and the creation of the performance improvement plan and continuous improvement within the organisation. Assists in the creation, upkeep and analysis of the supply chain organisations operating model.
Personal/Management Competency(ies):	5.1 Design and implement supply chain system and strategies<br>5.5 Manage and plan projects<br>5.5.1 Develop and direct project plans<br>5.5.2 Execute strategic decision making<br>5.5.3 Manage partnerships<br>5.5.4 Direct/participate in teamwork<br>5.6 Manage financial activities<br>6.5 Prove leadership abilities<br>6.5.1 Demonstrate resilience and ability to manage stress<br>6.6 Abide by rules / laws / legislation

5.1 Design and implement supply chain system and strategies

» Design a responsive, agile and efficient supply chain that has the ability to meet the changing needs of customers and deliver high-quality products with short lead times at low cost

» Establish the interrelationship of organisations, people, technology, activities, information, and resources involved in moving a product from supplier to customer

» Illustrate the management activities carried out in the course of running an organisation, including controlling, leading, monitoring, adjusting, organising and planning

» Strategically analyse the characteristics unique to public health, demonstrate awareness of the factors that could contribute to or hinder the delivery of logistics services

» Strategically analyse emerging concepts and principles in public health logistics; adapt current practices to incorporate new developments in the field

» Strategically analyse which type of inventory control system will be most effective for a particular programme or country

» Demonstrate in-depth knowledge the difference between continuous and period review systems of inventory control

» Develop and implement a formal logistics strategy and plan

5.5 Manage and plan projects

5.5.1 Develop and direct project plans

» Strategically analyse obstacles and critical events for ensuring the six rights of logistics management (right quantity, product, condition, place, time and cost) and develop contingency plans to address them; monitor progress and take necessary corrective action when needed

» Create an operational plan that is consistent with the strategic plan, links specific goals and strategies, and identifies the performance indicators to be used to measure achievements

» Implement the use of information gathering techniques from stakeholders, analysing situations and identifying implications to make correct decisions

» Analyse the questions, "Where are we going?" and "How are we going to get there?" and create a specific and purposeful path to achieve this.

» Implement the process reference model to managing supply chain processes (i.e., integrating business process re-engineering, benchmarking and process measurement)

» Strategically analyse the current state of a process and derive the desired future state

» Direct and implement the process of selecting and applying tools or technological solutions to frequently encountered problems

» Direct the process of providing staff with the necessary tools to carry out plans

» Implement the process of monitoring progress and making changes as required

» Direct meetings for planning, organisation and monitoring logistics activities

» Implement the process of ensuring staff are aware that they are accountable for achieving the desired results

» Illustrate planning, organising and managing resources to bring about the successful completion of projects

» Demonstrate in-depth knowledge of the key features of a successful change management strategy

5.5.2 Execute strategic decision making

» Demonstrate making decisions regarding one's own workload and area of responsibility

» Illustrate the process of prioritizing competing tasks and perform them quickly and efficiently according to their importance; finding new ways of organizing or planning work to accomplish it more efficiently

» Implement the proper time and space for consultation around decisions being made.

» Implement the process of considering the input of resources including staff needed

» Develop the process of checking assumptions against facts

» Strategically analyse the key issues in a complex situation and come to the heart of the problem quickly

» Strategically analyse relevant information before making decisions

» Make strategic decisions in the interest of the organisation's goals

	» Demonstrate how to analyse, evaluate and execute tough decisions when necessary<br>» Strategically analyse the impact of decisions on others' work or team goals<br>» Develop the process of communicating decisions and ensure they are incorporated into policies and processes<br>» Undertake inclusive and consultative strategic planning to establish strategic objectives<br>» Strategically analyse and implement emerging trends and practices<br>» Outline the macro and long-term consequences of decisions
5.5.3 Manage partnerships	» Implement the concepts of partnership working<br>» Develop the process of supporting implementation of partnership programmes<br>» Experiment with and trial new ideas with partners<br>» Build partnerships to deliver programme and increase impact<br>» Promote innovation and creativity in partnership work<br>» Develop the process of communicating key information with partner members<br>» Develop the process of involving and valuing partners in all aspects of programming<br>» Strategically analyse problems with supply chain relationships<br>» Illustrate the process of communicating effectively with nurses, doctors and other members of the healthcare team<br>» Develop the process of responding to consumer complaints or comments about services and/or advice received<br>» Illustrate collaboration and consultation across the sector and encourage the team to work with others to gain market insight<br>» Develop the process of sharing input from other key actors in the humanitarian sector and develop relationships with a cross-section of actors<br>» Direct the process of meeting the reporting requirements of vertical programmes<br>» Illustrate the structure of the health system at a national level and explain this to others<br>» Illustrate of the roles of other members of the healthcare team (including with consumers) in a way that engenders understanding and confidence in the team and its members<br>» Direct the structure of the organisation, environment and/or service in which they work<br>» Illustrate how vertical programmes work within the health system<br>» Establish the communication network to achieve work outcomes<br>» Demonstrate in-depth knowledge of the structure of the health system at the provincial/regional level and explain this to others<br>» Demonstrate in-depth knowledge of the roles and functions of government agencies in regulating and supporting supply chain organisations<br>» Demonstrate in-depth knowledge of change management (e.g. PtD Theory of Change)

5.5.4 Direct/participate in teamwork	»	Develop a methodology to work with team members within their area of authority to establish achievable goals and strategies that are consistent with the objectives established for the organisation as a whole
	»	Develop an organisational chart that shows the lines of reporting and responsibility among staff
	»	Implement a culture of actively contributing a perspective and making a positive contribution to team-based problem solving and decision making
	»	Create a culture of providing feedback, encouragement and support to team members for progressing strategic goals
	»	Strategically analyse team performance in relation to the organisation's mission and goals
	»	Create a culture of teams thinking for themselves and resolving problems
	»	Develop the process of encouraging and harnessing diversity within the team to boost team effectiveness
	»	Develop the process of encouraging and supporting the team to work through its stages of development and perform well
	»	Develop roles and responsibilities in relation to employees' expertise and the expectations of collaborating team members
	»	Illustrate where their position fits in the structure(organisation, SC, healthcare sector) , the responsibilities their position entails and the accountabilities linked to the position
5.6 Manage financial activities	»	Create rules for managing all resources with care, applying budgetary principles
	»	Take overall responsibility for meeting budgets and donor requirements
	»	Create and manage budgets (national, regional, etc.) as necessary for work (e.g., wages budget, touring budget, stationery budget, project budgets for using NGO funds)
	»	Implement the process of producing timely and clear financial reports for funders and donors
	»	Implement the process of keeping records following government budgeting, accounting and financial practices
	»	Establish the process of maintaining an overview of multiple budgets from multiple sources
	»	Develop the methodology of seeking and using information on financial funding requirements
	»	Develop the methodology to ensure timely decision making with regard to financial shortfalls
	»	Implement budget tracking for logistics activities according to established procedures
	»	Strategically analyse financial statements and explain components of a balance sheet and income statement
	»	Develop interactive decision support models that allow the development of multiple scenarios and demonstrate the sensitivity of multiple independent variables
	»	Illustrate the success or failure of a business using financial accounting

» Strategically analyse the calculations of the total system cost of delivering a product or service to the customer
» Establish the key considerations for developing a business plan
» Strategically analyse the structure against which business performance will be monitored (e.g., cost centres, chart of accounts)
» Implement key performance indicators used to monitor business performance (e.g., turnover, profitability)
» Implement strategies for minimising the risk of fraudulent activity (e.g., reconciliation of purchase orders, receipts and payment approvals; dual signatory arrangements for funds transfers)
» Implement investigative processes and options for confirming the existence of fraudulent activity (e.g., sample audits, forensic accounting services)
» Develop a process to prepare funding applications for trusts and grants
» Ensure financial transparency
» Strategically analyse the general monetary value of medicines and equipment
» Develop a process to secure financing and manage budgets to support distribution operations
» Illustrate the ability to translate financial information into SC language
» Demonstrate in-depth SC finance knowledge

6.5 Prove leadership abilities

» Strategically analyse input to meetings and programme development
» Analyse future trends and issues, and assist the organisation in meeting the challenges
» Develop a process to contribute to a collaborative working environment
» Demonstrate a drive for change and improvement develops opportunities for the organisation and sector
» Demonstrate channelling energy and ideas toward resolving issues
» Demonstrate the practise of communicating with influence
» Demonstrate showing courage to take an unpopular stance when needed
» Demonstrate steering and implementing change organisationally
» Establish the culture of anticipating and resolving conflict
» Establish a culture of showing initiative in working methods
» Exemplify personal drive and integrity
» Demonstrate serving as a role model for others to follow
» Demonstrate leadership and practice management skills, initiative and efficiency
» Demonstrate inspiring others through professional excellence, innovation and communication
» Illustrate situations in which a change in leadership style would be warranted (e.g., directive versus consultative style in the event of a crisis)

6.5.1 Demonstrate resilience and

» Demonstrate identifying symptoms of stress and take steps to reduce stress
» Demonstrate seeing the bigger picture and help others to

ability to manage stress		» Demonstrate recovering quickly from setbacks<br>» Demonstrate drawing on previous experience and support mechanisms to reduce the impact of stress on self and others<br>» Create a working environment that aims to minimise pressure and stress<br>» Demonstrate coping well under pressure, particularly in difficult environments<br>» Demonstrate acting as a role model for others and display courage under difficult circumstances<br>» Develop a methodology for recognising the limitations of staff and taking action to limit their exposure to harm when needed<br>» Implement the use of personal support mechanisms<br>» Demonstrate helping others identify personal support mechanisms<br>» Direct organisational policy to support self-care in agencies
6.6 Abide by rules / laws / legislation		» Ensure compliance with the legislation that covers the practice of public health supply chains and healthcare, and describe its purpose<br>» Implement requirements of professional codes, guidelines and standards adopted as part of the legislative framework<br>» Develop methodology to keep up to date with changes in legal instruments, as informed by national-level managers<br>» Ensure compliance with international regulations in decision making for the distribution system, including customs regulations on import and export requirements (e.g., trade tariffs and duties on imported goods) and security regulations (e.g., 2007 SAFE Ports Act, NAFTA or European Union trade agreements)<br>» Demonstrate in-depth knowledge of regulatory affairs and the key aspects of pharmaceutical registration and legislation<br>» Demonstrate in-depth knowledge of the principles of business economics and intellectual property rights, including patent interpretation<br>» Demonstrate in-depth knowledge of applicable procurement law and the practical effects of a contract's terms and conditions<br>» Strategically analyse current industry and government regulations governing sustainability<br>» Demonstrate in-depth knowledge of key hazardous materials handling laws<br>» Implement policies and procedures relating to workplace safety that are consistent with agreed or recognised standards<br>» Implement professional standards and conventions, as well as workplace policies and procedures, for preparing pharmaceutical products<br>» Develop all standard operating procedures<br>» Implement and direct the process of complying with data privacy and data protection laws
Basal Technology Competency(ies):	7.1 Data Science<br>7.4 Temperature and monitoring<br>7.5 Planning systems<br>7.6 ERP includes function of LMIS	

	7.12 Basic Office Skills<br>7.13 Have a command of technology
7.1 Data Science	» Strategically analyse end to end MIS technology components that are inherent in advanced MIS systems and explore how these may be relevant in different supply chain contexts<br>» Implement emerging technological approaches to data visualization and analysis<br>» Strategically analyse how data science can be applied within supply chain<br>» Demonstrate the ability to interact, provide guidance for data (dashboard) consolidation in various levels and use the tech tools<br>» Support movement of data into electronic form at lowest/remote SC level<br>» Demonstrate the ability to work with relational database
7.4 Temperature and monitoring	» Strategically analyse the current technology approaches for Temperature monitoring and sensors<br>» Strategically analyse what is required to implement this technology in a country context<br>» Strategically analyse infrastructure requirement for heat-sensitive items at peripheral 'lower level' of SC depending on volume and nature of items to handle;<br>» Implement regular preventive maintenance and calibration of cold-chain equipment with biomedical engineers or logisticians<br>» Implement the policy for temperature monitoring tools during transportation and storage<br>» Strategically analyse the steps involved in health logistic management cycle: selection-order-delivery-reception-storage-distribution-inventory<br>» Demonstrate in-depth knowledge of hardware qualification and system validation of temperature monitoring<br>» Implement and direct mass data management of temperature monitoring
7.5 Planning systems	» Implement blockchain and understand the building blocks for planning systems<br>» Analyse planning systems as a value-add capability for relevant global health use cases
7.6 ERP includes function of LMIS	» Implement a Master Data Management System<br>» Demonstrate an in-depth understanding of the critical components of an ERP system<br>» Demonstrate an in-depth understanding of the meaning of common document types found within an ERP/LMIS<br>» Demonstrate an in-depth understanding of the logic of data visibility within an ERP<br>» Demonstrate in-depth knowledge on the importance of data integrity, record management and information security
7.12 Basic Office Skills	» Demonstrate a good understanding of common presentation authoring packages

	» Demonstrate a good understanding of common spreadsheet authoring packages<br>» Demonstrate a good understanding of common document authoring packages<br>» Demonstrate a good understanding of common email authoring packages<br>» Demonstrate a good understanding of common instant messaging packages<br>» Demonstrate a good understanding of common video conferencing authoring packages
7.13 Have a command of technology	» Implement the use of technology suitable to the job held<br>» Review new developments and technologies in the sector<br>» Analyse new technologies and recognise potential benefits for the sector<br>» Analyse the resources and support that are provided across the organisation to enable colleagues to make the best use of available technology<br>» Develop the organisations' strategy for technology use<br>» Strategically analyse how to minimise environmental damage through technology use<br>» Implement the use of technology to maximise effectiveness and efficiency<br>» Implement the use of field-based technology (e.g., radio, general packet radio service (GPRS), satellite phone)
Advanced Technology Competency(ies):	7.2 Blockchain<br>7.3 Unmanned Aerial Vehicles (UAVs)<br>7.7 Automation<br>7.8 Artificial Intelligence<br>7.9 Additive Manufacturing<br>7.10 Internet of things<br>7.11 Cloud Computing<br>7.14 eProcurement
7.2 Blockchain	» Strategically analyse blockchain as a value-add capability for relevant global health use cases
7.3 Unmanned Aerial Vehicles (UAVs)	» Demonstrate in-depth knowledge of the current UAV landscape, their application and the enabling environment required to support country implementation<br>» Strategically analyse UAVs as a value-add capability for relevant global health use cases<br>» Demonstrate an in-depth understanding of the laws/ regulations around the use of UAV's
7.7 Automation	» Strategically analyse automation as a value-add capability for relevant global health use cases

7.8 Artificial Intelligence	» Strategically analyse artificial intelligence as a value-add capability for relevant global health use cases
7.9 Additive Manufacturing	» Strategically analyse additive manufacturing as a value-add capability for relevant global health use cases
7.10 Internet of things (IoT)	» Strategically analyse the opportunity of IoT used in conjunction with AI<br>» Strategically analyse the internet of things as a value-add capability for relevant global health use cases
7.11 Cloud Computing	» Strategically analyse cloud computing as a value-add capability for relevant global health use cases
7.14 eProcurement	» Develop a methodology on how to transact with an eProcurement system where necessary<br>» Strategically analyse eProcurement as a value-add capability for relevant global health use cases<br>» Strategically analyse the benefits and limitations of eProcurement to best utilise the system

Key Performance Indicators:

- % of emergency orders issued in the last 12 months
- % of product selection based on National Essential Medicines List
- % of procured products that meet Stringent Regulatory Authority (SRA) or WHO standards
- Availability - Stock out rate - Number of missed line item quantity deliveries due to stock out versus total line item quantity deliveries
- Availability - Stock levels - Current on hand stock versus Planned on hand stock
- Forecasting and Supply Planning done within the specified cycle time to support the Supply Chain performance requirements.
- Data accuracy - Vendor Master data accuracy, item / sku Master Data accuracy
- Establish Product Demand and Resources Cycle Time and recorded.in the "ERP" system against the products and resources.
- Establish Supply Plan Cycle Times and recorded in the "ERP" system.
- Fill Rate vs Planned fill rate
- Forecast accuracy, measured and reported on.
- Identify, Prioritize, and Aggregate Supply Chain Requirements Cycle Time - Actual Time taken to determine net supply chain requirements by product and SKU vs Planned Time

- Inventory Days of Supply - Actual inventory - Planned Demand over the planning horizon divided by the average daily usage
- Material and Resource Plan valid. Number of valid actions performed versus number of actions performed. (Exception messages trigger due to invalid schedules)
- Order accuracy - Net requirement versus order quantities in the planning horizon
- Procurement planning Cycle Time, by product and resource. Actual Overall planning cycle versus required Overall planning cycle time to achieve Supply Chain performance requirements.
- Ratio between median price of pro- ducts procured and the international median reference value
- Reports submitted on time (for all and separate reports) in accordance with business rules (reporting calendar)
- Supply adequacy - Net requirements are reflected in the Procurement pipeline to secure sufficient future supply.
- Supply Chain Planning Cost recorded and measured against required Supply China Planning Cycle times to accomplish Supply Chain Strategic objectives.
- Supply Plan report; supplier performance report
- Vendor on time delivery (VOTD)

Training:	Qualifications Available:	Certifications Available:
» Project Management<br>» Supply Chain operating models<br>» Data Analysis<br>» Executive Communication		» APICS Certified supply chain professional certificate<br>» CIPS Level 6 Professional Diploma in Procurement and Supply

Deputy Director of Distribution

Organisation:

SC-focused Organisation

Competency Level:	Serial Number:	Primary Process:	Job Role:	Supervises:
**Strategic**	**3-31-34-5**	**Plan – Supply Chain**	**Deputy Director of Distribution**	**Manager – Procurement, Manager – Warehouse and Distribution**

Contributions	Articulate the Distribution Strategy for the Public Health System Organisation. Facilitates the execution of the Distribution Strategy, maintains the alignment between Distribution Strategy and Supply Chain Strategy and ensures that the execution supports the Supply Chain Strategy.
Technical Competency(ies):	3.1 Undertake storage, warehousing and inventory management<br>3.1.1 Manage storage of commodities during emergency<br>3.4 Manage transport for commodities<br>3.4.1 Manage transport for commodities during disaster

3.1 Undertake storage, warehousing and inventory management

- Develop methods to efficiently distribute products among suppliers, distribution centres, warehouses and customers through a logistics network
- Develop a total systems approach to designing and managing the entire flow of information, materials, and services
- Strategically analyse the choice of equipment or materials based on suitability for intended use, accuracy, safety of use and cost
- Define rules to configure warehouses to have formal storage locations that identify the row, rack section, level and shelf location, typically with an alphanumeric location bar code or label
- Develop rules for how medicines are appropriately stored, considering temperature, access and cleanliness
- Develop rules for the appropriate use of, and ability to maintain, the cold chain
- Develop rules for the movement and storage of materials within a warehouse
- Develop methods to secure the medical store and limit access to staff
- Develop organisation processes, instructions, rules and parameters for warehouse and inventory management and use of equipment (e.g., annual stock turns, expiry date tracking, stock procurement, rotation and retrieval, equipment maintenance)
- Develop policies and procedures for stock handling, distribution and withdrawal consistent with maintaining safety (e.g., policies for distribution of concentrated electrolyte solutions and cytotoxics, identification of cytotoxic drug products within the work environment, separation of like-named or like-packaged products)
- Develop a standard for information on the supplies being stored
- Develop processes to consolidate several items into larger units for fewer handlings

	» Review and Implement methods of stock rotation (e.g., first in first out, FIFO, or first to expire first out, FEFO)<br>» Develop a process on how to enter the quantity received and update the warehouse management system software with the stock on hand when each order is delivered<br>» Define policies for the assessment of the required levels of stock and/or equipment in specific situations<br>» Develop processes that accurately verify rolling stocks<br>» Develop a methodology for monitoring the location and condition of the supplies being stored<br>» Develop solutions with the information on the storage locations and facilities<br>» Develop strategies for the resolution of problems in using the storage locations and facilities
3.1.1 Manage storage of commodities during emergency	» Direct processes for the different types of warehouses available for emergency or disaster supply<br>» Directs the choice of storage site for emergency or disaster supply<br>» Direct the staff required for the storage of emergency or disaster supply<br>» Strategically analyse the equipment and material required for the warehousing of emergency or disaster supply<br>» Develop methods on how to conduct rapid assessments of logistical needs in emergencies
3.4 Manage transport for commodities	» Direct distribution activities, including a fleet of vehicles and distribution schedules, to deliver health commodities to facilities.<br>» Develop policies and procedures related to handling and transporting special products such as vaccines, HIV/AIDS drugs, other high value products and narcotics<br>» Strategically analyse shipping methods, considering trade-offs between costs and benefits<br>» Understand strategic impact of compliance regarding all local and national laws/requirements for vehicle safety, driving regulations and licensing<br>» Review and Implement proper packing and labelling methods for transportation of hazardous materials<br>» Strategically analyse how to maximize freight loads while minimizing freight costs<br>» Illustrate efficient use of transportation resources while meeting customers' needs<br>» Direct movement demands with vehicle resources<br>» Develop quantitative techniques for solving logistics problems, such as designing routes and scheduling vehicles<br>» Strategically analyse the delivery of products to hospitals and district health offices with the accompanying dispatch note and report and requisition forms<br>» Formulate transport options for order delivery<br>» Develop a system for implementing a vehicle maintenance plan<br>» Synthesize information on distribution requirements<br>» Strategically analyse any problems in distribution requirements

- Develop the process of equipping distribution sites
- Strategically analyse what information needs to be provided to stakeholders for better coordination of distribution
- Review and Implement tracking devices (tools and indicators)
- Implement the process of training of drivers, van assistants, fleet administrators and expeditors
- Direct the process of supporting investigations around missed OTIF and other failures

3.4.1 Manage transport for commodities during disaster	» Determine which of the different types of transport and their characteristics should be used for emergency or disaster supply<br>» Develop a plan for the achievement of a rapid response logistics plan for deployment in the event of a medical emergency.
Personal/Management Competency(ies):	5.5 Manage and plan projects<br>5.5.2 Execute strategic decision making<br>6.5 Prove leadership abilities<br>6.5.1 Demonstrate resilience and ability to manage stress<br>6.6 Abide by rules / laws / legislation

5.5 Manage and plan projects

5.5.2 Execute strategic decision making

- Demonstrate making decisions regarding one's own workload and area of responsibility
- Illustrate the process of prioritizing competing tasks and perform them quickly and efficiently according to their importance; finding new ways of organizing or planning work to accomplish it more efficiently
- Implement the proper time and space for consultation around decisions being made.
- Implement the process of considering the input of resources including staff needed
- Develop the process of checking assumptions against facts
- Strategically analyse the key issues in a complex situation and come to the heart of the problem quickly
- Strategically analyse relevant information before making decisions
- Make strategic decisions in the interest of the organisation's goals
- Demonstrate how to analyse, evaluate and execute tough decisions when necessary
- Strategically analyse the impact of decisions on others' work or team goals
- Develop the process of communicating decisions and ensure they are incorporated into policies and processes
- Undertake inclusive and consultative strategic planning to establish strategic objectives
- Strategically analyse and implement emerging trends and practices
- Outline the macro and long-term consequences of decisions

6.5 Prove leadership abilities	» Strategically analyse input to meetings and programme development<br>» Analyse future trends and issues, and assist the organisation in meeting the challenges<br>» Develop a process to contribute to a collaborative working environment<br>» Demonstrate a drive for change and improvement develops opportunities for the organisation and sector<br>» Demonstrate channelling energy and ideas toward resolving issues<br>» Demonstrate the practise of communicating with influence<br>» Demonstrate showing courage to take an unpopular stance when needed<br>» Demonstrate steering and implementing change organisationally<br>» Establish the culture of anticipating and resolving conflict<br>» Establish a culture of showing initiative in working methods<br>» Exemplify personal drive and integrity<br>» Demonstrate serving as a role model for others to follow<br>» Demonstrate leadership and practice management skills, initiative and efficiency<br>» Demonstrate inspiring others through professional excellence, innovation and communication<br>» Illustrate situations in which a change in leadership style would be warranted (e.g., directive versus consultative style in the event of a crisis)
6.5.1 Demonstrate resilience and ability to manage stress	» Demonstrate identifying symptoms of stress and take steps to reduce stress<br>» Demonstrate seeing the bigger picture and help others to<br>» Demonstrate recovering quickly from setbacks<br>» Demonstrate drawing on previous experience and support mechanisms to reduce the impact of stress on self and others<br>» Create a working environment that aims to minimise pressure and stress<br>» Demonstrate coping well under pressure, particularly in difficult environments<br>» Demonstrate acting as a role model for others and display courage under difficult circumstances<br>» Develop a methodology for recognising the limitations of staff and taking action to limit their exposure to harm when needed<br>» Implement the use of personal support mechanisms<br>» Demonstrate helping others identify personal support mechanisms<br>» Direct organisational policy to support self-care in agencies
6.6 Abide by rules / laws / legislation	» Ensure compliance with the legislation that covers the practice of public health supply chains and healthcare, and describe its purpose<br>» Implement requirements of professional codes, guidelines and standards adopted as part of the legislative framework<br>» Develop methodology to keep up to date with changes in legal instruments, as informed by national-level managers

- Ensure compliance with international regulations in decision making for the distribution system, including customs regulations on import and export requirements (e.g., trade tariffs and duties on imported goods) and security regulations (e.g., 2007 SAFE Ports Act, NAFTA or European Union trade agreements)
- Demonstrate in-depth knowledge of regulatory affairs and the key aspects of pharmaceutical registration and legislation
- Demonstrate in-depth knowledge of the principles of business economics and intellectual property rights, including patent interpretation
- Demonstrate in-depth knowledge of applicable procurement law and the practical effects of a contract's terms and conditions
- Strategically analyse current industry and government regulations governing sustainability
- Demonstrate in-depth knowledge of key hazardous materials handling laws
- Implement policies and procedures relating to workplace safety that are consistent with agreed or recognised standards
- Implement professional standards and conventions, as well as workplace policies and procedures, for preparing pharmaceutical products
- Develop all standard operating procedures
- Implement and direct the process of complying with data privacy and data protection laws

Basal Technology Competency(ies):	7.1 Data Science<br>7.4 Temperature and monitoring<br>7.5 Planning systems<br>7.6 ERP includes function of LMIS<br>7.12 Basic Office Skills<br>7.13 Have a command of technology

7.1 Data Science

- Strategically analyse end to end MIS technology components that are inherent in advanced MIS systems and explore how these may be relevant in different supply chain contexts
- Implement emerging technological approaches to data visualization and analysis
- Strategically analyse how data science can be applied within supply chain
- Demonstrate the ability to interact, provide guidance for data (dashboard) consolidation in various levels and use the tech tools
- Support movement of data into electronic form at lowest/remote SC level
- Demonstrate the ability to work with relational database

7.4 Temperature and monitoring

- Strategically analyse the current technology approaches for Temperature monitoring and sensors
- Strategically analyse what is required to implement this technology in a country context

	» Strategically analyse infrastructure requirement for heat-sensitive items at peripheral 'lower level' of SC depending on volume and nature of items to handle;<br>» Implement regular preventive maintenance and calibration of cold-chain equipment with biomedical engineers or logisticians<br>» Implement the policy for temperature monitoring tools during transportation and storage<br>» Strategically analyse the steps involved in health logistic management cycle: selection-order-delivery-reception-storage-distribution-inventory<br>» Demonstrate in-depth knowledge of hardware qualification and system validation of temperature monitoring<br>» Implement and direct mass data management of temperature monitoring
7.5 Planning systems	» Implement blockchain and understand the building blocks for planning systems<br>» Analyse planning systems as a value-add capability for relevant global health use cases
7.6 ERP includes function of LMIS	» Implement a Master Data Management System<br>» Demonstrate an in-depth understanding of the critical components of an ERP system<br>» Demonstrate an in-depth understanding of the meaning of common document types found within an ERP/LMIS<br>» Demonstrate an in-depth understanding of the logic of data visibility within an ERP<br>» Demonstrate in-depth knowledge on the importance of data integrity, record management and information security
7.12 Basic Office Skills	» Demonstrate a good understanding of common presentation authoring packages<br>» Demonstrate a good understanding of common spreadsheet authoring packages<br>» Demonstrate a good understanding of common document authoring packages<br>» Demonstrate a good understanding of common email authoring packages<br>» Demonstrate a good understanding of common instant messaging packages<br>» Demonstrate a good understanding of common video conferencing authoring packages
7.13 Have a command of technology	» Implement the use of technology suitable to the job held<br>» Review new developments and technologies in the sector<br>» Analyse new technologies and recognise potential benefits for the sector<br>» Analyse the resources and support that are provided across the organisation to enable colleagues to make the best use of available technology<br>» Develop the organisations' strategy for technology use<br>» Strategically analyse how to minimise environmental damage through technology use

	» Implement the use of technology to maximise effectiveness and efficiency<br>» Implement the use of field-based technology (e.g., radio, general packet radio service (GPRS), satellite phone)
Advanced Technology Competency(ies):	7.2 Blockchain<br>7.3 Unmanned Aerial Vehicles (UAVs)<br>7.7 Automation<br>7.8 Artificial Intelligence<br>7.9 Additive Manufacturing<br>7.10 Internet of things<br>7.11 Cloud Computing<br>7.14 eProcurement
7.2 Blockchain	» Strategically analyse blockchain as a value-add capability for relevant global health use cases
7.3 Unmanned Aerial Vehicles (UAVs)	» Demonstrate in-depth knowledge of the current UAV landscape, their application and the enabling environment required to support country implementation<br>» Strategically analyse UAVs as a value-add capability for relevant global health use cases<br>» Demonstrate an in-depth understanding of the laws/ regulations around the use of UAV's
7.7 Automation	» Strategically analyse automation as a value-add capability for relevant global health use cases
7.8 Artificial Intelligence	» Strategically analyse artificial intelligence as a value-add capability for relevant global health use cases
7.9 Additive Manufacturing	» Strategically analyse additive manufacturing as a value-add capability for relevant global health use cases
7.10 Internet of things (IoT)	» Strategically analyse the opportunity of IoT used in conjunction with AI<br>» Strategically analyse the internet of things as a value-add capability for relevant global health use cases
7.11 Cloud Computing	» Strategically analyse cloud computing as a value-add capability for relevant global health use cases

7.14 eProcurement	» Develop a methodology on how to transact with an eProcurement system where necessary<br>» Strategically analyse eProcurement as a value-add capability for relevant global health use cases<br>» Strategically analyse the benefits and limitations of eProcurement to best utilise the system

Key Performance Indicators:

- Accuracy of Risk Register
- Cycle time of QA/QC report
- Overall value at risk
- Partner/Donor integration, policy, guidelines and regulatory reforms
- QA/QC report
- Risk / Mitigation Costs
- Time to Recovery (TTR)
- Warehouse Efficiency - Time to Pick and Put away versus Planned Time to Pick and Put away. Presupposes the warehouse design promotes wave picking and flow
- Warehouse utilization - Volume used vs Volume Available
- Assess Delivery Performance Cycle Time - Actual Time from assessed requirement to time delivered to end-user compared to committed Delivery Cycle Time as per Supply Chain strategy
- Assess Supplier Performance Cycle Time - Actual Supplier performance Cycle time versus Supplier commitment cycle time

Training:	Qualifications Available:	Certifications Available:
SC Optimization<br>Scenario Planning<br>Integrated Business/ SC Planning<br>Supply chain Risk<br>Executive training<br>Advanced Supply Chain Management	Master of Philosophy in Business Management with options in Supply Chain Management<br>Master of Science in Development Planning<br>Master of Transport Studies	APICS Certified supply chain professional certificate<br>CIPS Level 6 Professional Diploma in Procurement and Supply

Deputy Director of Internal Services

Organisation				
**SC-focused Organisation**				
Competency Level:	Serial Number:	Primary Process:	Job Role:	Supervises:
**Strategic**	**5-531-34-5**	**Plan – Supply Chain**	**Deputy Director of Internal Services**	**Lead – Network Planning, Manager – Finance and Administration, Lead – Data Intelligence and Analytics, Manager – QA/ QMS, Manager – HR/ Workforce Development, Lead – Process Improvement and SC Adaptability, Lead – Planning and Performance, Manager - Occupational Safety, Manager – SC Data and Analytics, Manager - HR/ Workforce Development**
Contributions	Takes the cue from the articulated Supply Chain Strategy and formulates the Administrative framework to ensure adherence to the defined business rules, ensures that processes ensure sound internal controls. Works with Lead - SCM Unit to maintain all enablers.			
Technical Competency(ies):	3.4 Manage transport for commodities<br>3.4.1 Manage transport for commodities during disaster			

3.4 Manage transport for commodities

- Direct distribution activities, including a fleet of vehicles and distribution schedules, to deliver health commodities to facilities.
- Develop policies and procedures related to handling and transporting special products such as vaccines, HIV/AIDS drugs, other high value products and narcotics
- Strategically analyse shipping methods, considering trade-offs between costs and benefits
- Understand strategic impact of compliance regarding all local and national laws/requirements for vehicle safety, driving regulations and licensing
- Review and Implement proper packing and labelling methods for transportation of hazardous materials
- Strategically analyse how to maximize freight loads while minimizing freight costs
- Illustrate efficient use of transportation resources while meeting customers' needs
- Direct movement demands with vehicle resources
- Develop quantitative techniques for solving logistics problems, such as designing routes and scheduling vehicles
- Strategically analyse the delivery of products to hospitals and district health offices with the accompanying dispatch note and report and requisition forms
- Formulate transport options for order delivery
- Develop a system for implementing a vehicle maintenance plan

	» Synthesize information on distribution requirements<br>» Strategically analyse any problems in distribution requirements<br>» Develop the process of equipping distribution sites<br>» Strategically analyse what information needs to be provided to stakeholders for better coordination of distribution<br>» Review and Implement tracking devices (tools and indicators)<br>» Implement the process of training of drivers, van assistants, fleet administrators and expeditors<br>» Direct the process of supporting investigations around missed OTIF and other failures
3.4.1 Manage transport for commodities during disaster	» Determine which of the different types of transport and their characteristics should be used for emergency or disaster supply<br>» Develop a plan for the achievement of a rapid response logistics plan for deployment in the event of a medical emergency.
Personal/Management Competency(ies):	5.3 Implement risk management and monitoring and evaluation activities for the supply chain<br>5.3.1 Ensure monitoring and evaluation activities are completed<br>5.5 Manage and plan projects<br>5.5.2 Execute strategic decision making<br>6.5 Prove leadership abilities<br>6.5.1 Demonstrate resilience and ability to manage stress<br>6.6 Abide by rules / laws / legislation
5.3 Implement risk management and monitoring and evaluation activities for the supply chain	
5.3.1 Ensure monitoring and evaluation activities are completed	» Strategically analyse pertinent information — such as problem symptoms from knowledgeable sources, carrying these through to the problems, potential causes and root causes of the problem to maintain processes<br>» Implement the process of quantifying the operational performance of similar companies and establish internal targets based on best-in-class results<br>» Establish key performance measurements and continuous process improvement initiatives to improve process quality on a continual basis<br>» Strategically analyse the results of the analyses provided<br>» Implement the process of controlling and checking errors, taking corrective action so deviation from standards are minimised and the organisation's goals are achieved<br>» Consider the use of appropriate technological developments to improve the system<br>» Recognise that continuous process improvement is an accepted way of organisational life

	» Implement ways of eliminating unnecessary steps in system design<br>» Develop processes that strive to eliminate waste<br>» Implement processes to encourage sustainability (e.g., reducing carbon footprint, establishing a paperless office, using renewable energy)<br>» Direct the systematic approach used to close process or system performance gaps through streamlining and cycle time reduction, and identify and eliminate causes of quality below specifications, process variation and non-value-adding activities<br>» Strategically analyse the relationship between technology and process functionality<br>» Illustrate how policy, guidelines and regulatory reforms impact SC performance
**5.5 Manage and plan projects**	
**5.5.2 Execute strategic decision making**	» Demonstrate making decisions regarding one's own workload and area of responsibility<br>» Illustrate the process of prioritizing competing tasks and perform them quickly and efficiently according to their importance; finding new ways of organizing or planning work to accomplish it more efficiently<br>» Implement the proper time and space for consultation around decisions being made.<br>» Implement the process of considering the input of resources including staff needed<br>» Develop the process of checking assumptions against facts<br>» Strategically analyse the key issues in a complex situation and come to the heart of the problem quickly<br>» Strategically analyse relevant information before making decisions<br>» Make strategic decisions in the interest of the organisation's goals<br>» Demonstrate how to analyse, evaluate and execute tough decisions when necessary<br>» Strategically analyse the impact of decisions on others' work or team goals<br>» Develop the process of communicating decisions and ensure they are incorporated into policies and processes<br>» Undertake inclusive and consultative strategic planning to establish strategic objectives<br>» Strategically analyse and implement emerging trends and practices<br>» Outline the macro and long-term consequences of decisions
**6.5 Prove leadership abilities**	» Strategically analyse input to meetings and programme development<br>» Analyse future trends and issues, and assist the organisation in meeting the challenges<br>» Develop a process to contribute to a collaborative working environment<br>» Demonstrate a drive for change and improvement develops opportunities for the organisation and sector<br>» Demonstrate channelling energy and ideas toward resolving issues

	» Demonstrate the practise of communicating with influence<br>» Demonstrate showing courage to take an unpopular stance when needed<br>» Demonstrate steering and implementing change organisationally<br>» Establish the culture of anticipating and resolving conflict<br>» Establish a culture of showing initiative in working methods<br>» Exemplify personal drive and integrity<br>» Demonstrate serving as a role model for others to follow<br>» Demonstrate leadership and practice management skills, initiative and efficiency<br>» Demonstrate inspiring others through professional excellence, innovation and communication<br>» Illustrate situations in which a change in leadership style would be warranted (e.g., directive versus consultative style in the event of a crisis)
6.5.1 Demonstrate resilience and ability to manage stress	» Demonstrate identifying symptoms of stress and take steps to reduce stress<br>» Demonstrate seeing the bigger picture and help others to<br>» Demonstrate recovering quickly from setbacks<br>» Demonstrate drawing on previous experience and support mechanisms to reduce the impact of stress on self and others<br>» Create a working environment that aims to minimise pressure and stress<br>» Demonstrate coping well under pressure, particularly in difficult environments<br>» Demonstrate acting as a role model for others and display courage under difficult circumstances<br>» Develop a methodology for recognising the limitations of staff and taking action to limit their exposure to harm when needed<br>» Implement the use of personal support mechanisms<br>» Demonstrate helping others identify personal support mechanisms<br>» Direct organisational policy to support self-care in agencies
6.6 Abide by rules / laws / legislation	» Ensure compliance with the legislation that covers the practice of public health supply chains and healthcare, and describe its purpose<br>» Implement requirements of professional codes, guidelines and standards adopted as part of the legislative framework<br>» Develop methodology to keep up to date with changes in legal instruments, as informed by national-level managers<br>» Ensure compliance with international regulations in decision making for the distribution system, including customs regulations on import and export requirements (e.g., trade tariffs and duties on imported goods) and security regulations (e.g., 2007 SAFE Ports Act, NAFTA or European Union trade agreements)<br>» Demonstrate in-depth knowledge of regulatory affairs and the key aspects of pharmaceutical registration and legislation<br>» Demonstrate in-depth knowledge of the principles of business economics and intellectual property rights, including patent interpretation

- Demonstrate in-depth knowledge of applicable procurement law and the practical effects of a contract's terms and conditions
- Strategically analyse current industry and government regulations governing sustainability
- Demonstrate in-depth knowledge of key hazardous materials handling laws
- Implement policies and procedures relating to workplace safety that are consistent with agreed or recognised standards
- Implement professional standards and conventions, as well as workplace policies and procedures, for preparing pharmaceutical products
- Develop all standard operating procedures
- Implement and direct the process of complying with data privacy and data protection laws

Basal Technology Competency(ies):	7.6 ERP includes function of LMIS<br>7.12 Basic Office Skills<br>7.13 Have a command of technology
7.6 ERP includes function of LMIS	» Implement a Master Data Management System<br>» Demonstrate an in-depth understanding of the critical components of an ERP system<br>» Demonstrate an in-depth understanding of the meaning of common document types found within an ERP/LMIS<br>» Demonstrate an in-depth understanding of the logic of data visibility within an ERP<br>» Demonstrate in-depth knowledge on the importance of data integrity, record management and information security
7.12 Basic Office Skills	» Demonstrate a good understanding of common presentation authoring packages<br>» Demonstrate a good understanding of common spreadsheet authoring packages<br>» Demonstrate a good understanding of common document authoring packages<br>» Demonstrate a good understanding of common email authoring packages<br>» Demonstrate a good understanding of common instant messaging packages<br>» Demonstrate a good understanding of common video conferencing authoring packages
7.13 Have a command of technology	» Implement the use of technology suitable to the job held<br>» Review new developments and technologies in the sector<br>» Analyse new technologies and recognise potential benefits for the sector<br>» Analyse the resources and support that are provided across the organisation to enable colleagues to make the best use of available technology<br>» Develop the organisations' strategy for technology use<br>» Strategically analyse how to minimise environmental damage through technology use<br>» Implement the use of technology to maximise effectiveness and efficiency

	» Implement the use of field-based technology (e.g., radio, general packet radio service (GPRS), satellite phone)
Advanced Technology Competency(ies):	7.2 Blockchain<br>7.7 Automation<br>7.9 Additive Manufacturing<br>7.10 Internet of things<br>7.14 eProcurement
7.2 Blockchain	» Strategically analyse blockchain as a value-add capability for relevant global health use cases
7.7 Automation	» Strategically analyse automation as a value-add capability for relevant global health use cases
7.9 Additive Manufacturing	» Strategically analyse additive manufacturing as a value-add capability for relevant global health use cases
7.10 Internet of things (IoT)	» Strategically analyse the opportunity of IoT used in conjunction with AI<br>» Strategically analyse the internet of things as a value-add capability for relevant global health use cases
7.14 eProcurement	» Develop a methodology on how to transact with an eProcurement system where necessary<br>» Strategically analyse eProcurement as a value-add capability for relevant global health use cases<br>» Strategically analyse the benefits and limitations of eProcurement to best utilise the system

Key Performance Indicators:

- Assess Delivery Performance Cycle Time - Actual Time from assessed requirement to time delivered to end-user compared to committed Delivery Cycle Time as per Supply Chain strategy
- Assess Supplier Performance Cycle Time - Actual Supplier performance Cycle time versus Supplier commitment cycle time
- Inventory accuracy - Cycle count results
- Transportation lead time accuracy - Actual Transportation lead time versus Transportation promised lead time.
- Transport efficiency - Total cost of transport versus planned cost of transport by mode

- In-bound cost of acquisition - Actual total cost of acquisition versus Total Planned cost of acquisition including landed costs
- Manage performance cycle time
- Performance Report - Actual versus Plan reports for all metrics
- Internal Controls Enhance Supply Chain Reliability. Audit Internal Controls measure Number of Internal Controls that negate Supply Chain efficiency versus Total internal controls. Report on the non-conformance
- Business Rules support the Supply Chain Strategy. Business Rules Aligned to Strategy

Training:	Qualifications Available:	Certifications Available:
» Executive training<br>» IT Management<br>» Supply chain risk<br>» Financial management	» Master of Philosophy in Health Innovation	

Managerial
Manager – Procurement

Organisation:

SC-focused Organisation

Competency Level:	Serial Number:	Primary Process:	Job Role:	Supervises:
Managerial	2-21-11-4	Plan – Procurement	Manager – Procurement	Lead - Procurement, Lead – Procurement and Supply, Officer – Product Selection Use, Officer – Supply Planning/ Supplier Management, Officer – Strategic Procurement, Officer – Tender Management, Officer – Contracts, Officer – Import and Clearance, Lead - Returns Management

Contributions	Manages the Procurement department, is accountable for the metrics related to the Procure process.
Technical Competency(ies):	1.1 Select the appropriate product<br>1.2 Define the specifications of the product, including product quality<br>1.5 Supply planning<br>2.1 Manage procurement costs and budget<br>2.2 Build and maintain supplier relationships

1.1 Select the appropriate product	» Analyse the broad concepts of the national medicines policy, essential medicines lists, essential equipment lists, standard treatment guidelines and “dangerous drug” (DDA) policy<br>» Analyse the government system required to add and subtract items from the essential medicines list and the essential equipment list<br>» Ensure adherence the government system required to alter standard treatment guidelines, dangerous drug policy and national medicines policy<br>» Determine the type of supplies and services that are required<br>» Convene regularly scheduled coordination meetings with stakeholders involved in financing, procuring or distributing commodities<br>» Evaluate the steps needed to bring a medicinal product to the market, including the safety, quality, efficacy and pharmacoeconomic assessments of the product
1.2 Define the specifications of the product, including product quality	» Advise on specifications for procurement<br>» Analyse the characteristics of a good specification<br>» Determine the types of specifications<br>» Determine the advantages of functional and performance specifications<br>» Evaluate the contents of a specification<br>» Analyse the procurement staff responsibilities in the specification process<br>» Analyse the quality standard of product<br>» Not Applicable
1.5 Supply planning	» Demonstrate a purchasing and supply plan in line with national priorities<br>» Evaluate the balance supply chain resources and trigger activities to correct any imbalances<br>» Analyse the formalisation of sales and operations plans through sales and operations meeting.<br>» Evaluate the translation of sales and operations plan into executable plans to ensure dispatch of goods in line with overall priorities.<br>» Monitor and manage the national commodity pipeline
2.1 Manage procurement costs and budget	» Ensure the organisation follows public procurement regulations<br>» Ensure adherence to the approved procurement budget<br>» Evaluate financial and management accounting practices within procurement<br>» Analyse commercial factors that contribute toward cost reduction, price savings and value improvement opportunities<br>» Analyse the principles of foreign exchange rates and how they impact prices

2.2 Build and maintain supplier relationships

- Analyse the market
- Analyse adherence to the processes for prequalification and tender contracting
- Determine the factors that define a good supplier
- Effectively locate and source key suppliers, while analysing the total cost associated with procuring an item or service
- Analyse supplier relationship management plans (e.g., sourcing, frequency of meetings, negotiate and monitor benchmarks for performance)
- Analyse of the process of educating suppliers to create value for customers by streamlining processes in the value chain
- Analyse the difference between strategic and non-strategic suppliers and the associated supplier management principles
- Analyse commercial factors that contribute toward cost reduction, price savings and value improvement opportunities
- Ensure the strategic sourcing practices drive down cost across consecutive tenders
- Analyse the process of working with suppliers to agree cost saving opportunities in the whole value chain, with an appropriate cost sharing mechanism applied to ensure a "win-win" outcome
- Evaluate the process of managing supplier risk
- Demonstrate ethical sourcing

Personal/Management Competency(ies):

5.5 Manage and plan projects
5.5.2 Execute strategic decision making
5.5.3 Manage partnerships
6.1 Demonstrate generic skills (e.g., literacy, numeracy, technology)
6.1.1 Exhibit high understanding of literacy and numeracy
6.2 Demonstrate strong communication skills
6.2.1 Practice cultural awareness

5.5 Manage and plan projects

5.5.2 Execute strategic decision making

- Determine how one can make decisions regarding one's own workload and area of responsibility
- Determine the process of prioritizing competing tasks and perform them quickly and efficiently according to their importance; finding new ways of organizing or planning work to accomplish it more efficiently
- Evaluate the proper time and space for consultation around decisions being made.
- Demonstrate the process of considering the input of resources including staff needed
- Analyse assumptions against facts
- Determine the key issues in a complex situation and come to the heart of the problem quickly

- Evaluate relevant information before making decisions
- Provide input to strategic decisions in the interest of the organisation's goals
- Evaluate when to analyse, evaluate and execute tough decisions when necessary
- Analyse the impact of decisions on others' work or team goals
- Communicate decisions and ensure they are incorporated into policies and processes
- Demonstrate inclusive and consultative strategic planning to establish strategic objectives
- Evaluate emerging trends and practices
- Analyse the macro and long-term consequences of decisions

5.5.3 Manage partnerships

- Demonstrate the concepts of partnership working
- Determine the process of supporting implementation of partnership programmes
- Analyse the process of experimenting with and trialling new ideas with partners
- Analyse partnerships to deliver programme and increase impact
- Analyse innovation and creativity in partnership work
- Communicate key information with partner members
- Involve and value partners in all aspects of programming
- Analyse problems with supply chain relationships
- Demonstrate the process of communicating effectively with nurses, doctors and other members of the healthcare team
- Analyse the process of responding to consumer complaints or comments about services and/or advice received
- Analyse collaboration and consultation across the sector and encourage the team to work with others to gain market insight
- Encourage input from other key actors in the humanitarian sector and develop relationships with a cross-section of actors
- Analyse the process of meeting the reporting requirements of vertical programmes
- Analyse the structure of the health system at a national level and explain this to others
- Analyse the roles of other members of the healthcare team (including with consumers) in a way that engenders understanding and confidence in the team and its members
- Analyse the structure of the organisation, environment and/or service in which they work
- Demonstrate how vertical programmes work within the health system
- Evaluate the communication network established to achieve work outcomes

- Demonstrate knowledge of the structure of the health system at the provincial/regional level and explain this to others
- Analyse the roles and functions of government agencies in regulating and supporting supply chain organisations
- Demonstrate knowledge of change management (e.g. PtD Theory of Change)

6.1 Demonstrate generic skills (e.g., literacy, numeracy, technology)

6.1.1 Exhibit high understanding of literacy and numeracy

- Encourage speaking clearly and confidently, organizing information in a logical manner, and considering voice tone and pace
- Demonstrate responding to verbal messages and other cues (active listening)
- Demonstrate expressing ideas and opinions clearly in written and verbal form
- Demonstrate communicating information accurately, concisely and confidently in writing and verbally
- Demonstrate a level of mathematics suitable to the job held
- Demonstrate knowledge of visual presentation techniques, including charting, histograms and flow sheets
- Demonstrate reading and comprehending at a level necessary to properly complete duties of the position
- Demonstrate the process of interpreting written information in documents, such as reports, SOPs, LMIS forms, graphs, calendars, schedules, notices and directions
- Demonstrate paying attention to detail and identifying the main ideas, detecting inconsistencies and identifying missing information in documents
- Demonstrate computer skills — such as using Word, Excel, PowerPoint and the internet — suitable to the job level
- Demonstrate the process of analysing and validating KPIs
- Participate in the evaluation of activities that act as standards against which the KPIs are measured
- Demonstrate the process of performing critical self-review on work before submission

6.2 Demonstrate strong communication skills

- Demonstrate being truthful and trustworthy, and supplying accurate information at all times
- Demonstrate working as part of a workplace team
- Determine vocabulary and communication style and form for both written and verbal communication that is appropriate for the situation, audience and material being communicated (e.g., avoid unnecessary jargon, clearly explain medical and SCM terminology)
- Demonstrate open communication within constraints of confidentiality

- Analyse communications about logistics
- Demonstrate participation in meetings, expressing one's opinions, being aware of others' needs, and being appropriately assertive when required
- Demonstrate perspective into the point of view of others, understanding their needs and goals
- Demonstrate the practise of building trust, rapport and credibility with others
- Determine the communication needs and concerns of others and respond to them
- Demonstrate avoiding conflict between work and personal interests
- Demonstrate the practice of meaningful two-way communication
- Demonstrate influence on others by persuasively presenting thoughts and ideas
- Demonstrate assertiveness skills to deal with unreasonable requests and/or refusals that would compromise practice or consumer care
- Demonstrate a positive, supportive and appreciative attitude
- Demonstrate the ability to balance the organisation's needs and the customer's needs
- Demonstrate self-control by maintaining composure and keeping emotions in check, even in difficult situations; deal calmly and effectively with stressful situations
- Demonstrate the expressing of opinions and providing information in written and/or verbal form in a manner that does not elicit concern, anger or other adverse response
- Demonstrate the use of a systematic process for following up that demonstrates written reports have been received and understood
- Evaluate the means by which responses to input to the work environment are monitored
- Seek practical ways to overcome barriers to communication
- Ensure language representation (e.g., interpreters) at meetings when appropriate
- Demonstrate tackling difficult situations and resolving disputes between staff
- Demonstrate giving both positive and negative feedback sensitively
- Demonstrate maintaining and improving communication
- Demonstrate the process of giving and requesting feedback frequently
- Demonstrate the ability to communicate effectively with shop stewards and union representatives

6.2.1 Practice cultural awareness

- Demonstrate cultural awareness and sensitivity, treating all people with fairness, respect and dignity
- Demonstrate anti-discriminatory practices in the organisation, including HR and disciplinary procedures
- Demonstrate cultural sensitivity, equality and fairness at all levels of the organisation
- Demonstrate challenging discriminatory behaviour directly and sensitively
- Demonstrate acting in a non-discriminatory way toward individuals and groups
- Demonstrate the process of integrating cultural awareness in learning and development approaches
- Demonstrate avoiding stereotypical responses by examining one's own behaviour and bias
- Demonstrate managing cultural diversity in teams and make the most of differences
- Demonstrate the use of strategies and/or resources for communicating effectively with people from different cultural backgrounds
- Supply information relating to values, beliefs and cultural backgrounds of consumers that may influence the way professional services are provided

Basal Technology Competency(ies):	7.4 Temperature and monitoring<br>7.6 ERP includes function of LMIS<br>7.12 Basic Office Skills<br>7.13 Have a command of technology

7.4 Temperature and monitoring

- Analyse the current technology approaches for Temperature monitoring and sensors
- Evaluate what is required to implement this technology in a country context
- Evaluate infrastructure requirement for heat-sensitive items at peripheral 'lower level' of SC depending on volume and nature of items to handle;
- Analyse regular preventive maintenance and calibration of cold-chain equipment with biomedical engineers or logisticians
- Evaluate the policy for temperature monitoring tools during transportation and storage
- Demonstrate the steps involved in health logistic management cycle: selection-order-delivery-reception-storage-distribution-inventory
- Determine hardware qualification and system validation of temperature monitoring
- Evaluate mass data management of temperature monitoring

7.6 ERP includes function of LMIS	» Evaluate a Master Data Management System<br>» Demonstrate an understanding of the critical components of an ERP system<br>» Demonstrate an understanding of the meaning of common document types found within an ERP/LMIS<br>» Demonstrate an understanding of the logic of data visibility within an ERP<br>» Demonstrate the importance of data integrity, record management and information security
7.12 Basic Office Skills	» Demonstrate a good understanding of common presentation authoring packages<br>» Demonstrate a good understanding of common spreadsheet authoring packages<br>» Demonstrate a good understanding of common document authoring packages<br>» Demonstrate a good understanding of common email authoring packages<br>» Demonstrate a good understanding of common instant messaging packages<br>» Demonstrate a good understanding of common video conferencing authoring packages
7.13 Have a command of technology	» Demonstrate the use of technology suitable to the job held<br>» Monitor new developments and technologies in the sector<br>» Experiment with new technologies and recognising potential benefits for the sector<br>» Ensure that resources and support are provided across the organisation to enable colleagues to make the best use of available technology<br>» Determine what the organisations' strategy is for technology use<br>» Determine how to minimise environmental damage through technology use<br>» Analyse the use of technology to maximise effectiveness and efficiency<br>» Analyse the use of field-based technology (e.g., radio, general packet radio service (GPRS), satellite phone)
Advanced Technology Competency(ies):	7.2 Blockchain<br>7.3 Unmanned Aerial Vehicles (UAVs)<br>7.7 Automation<br>7.9 Additive Manufacturing<br>7.11 Cloud Computing<br>7.14 eProcurement

7.2 Blockchain	» Evaluate blockchain as a value-add capability for relevant global health use cases
7.3 Unmanned Aerial Vehicles (UAVs)	» Analyse the current UAV landscape, their application and the enabling environment required to support country implementation<br>» Evaluate UAVs as a value-add capability for relevant global health use cases<br>» Demonstrate an understanding of the laws/ regulations around the use of UAV's
7.7 Automation	» Evaluate automation as a value-add capability for relevant global health use cases
7.9 Additive Manufacturing	» Evaluate additive manufacturing as a value-add capability for relevant global health use cases
7.11 Cloud Computing	» Evaluate cloud computing as a value-add capability for relevant global health use cases
7.14 eProcurement	» Determine how to transact with an eProcurement system where necessary<br>» Evaluate eProcurement as a value-add capability for relevant global health use cases<br>» Evaluate the benefits and limitations of eProcurement to best utilise the system

Key Performance Indicators:

- % of emergency orders issued in the last 12 months
- % of product selection based on National Essential Medicines List
- % of procured products that meet Stringent Regulatory Authority (SRA) or WHO standards
- Availability - Stock out rate - number of missed line item quantity deliveries due to stock out versus total line item quantity deliveries
- Availability - Stock levels - Current on hand stock versus Planned on hand stock
- Data accuracy - Vendor Master data accuracy, item / sku Master Data accuracy
- Demand analysis - Actual Demand versus Planned Demand
- Fill Rate vs Planned fill rate
- Forecast Accuracy - Using various measures of Forecast Error e.g. MAD

» Identify, Prioritize, and Aggregate Supply Chain Requirements Cycle Time - Actual Time taken to determine net supply chain requirements by product and SKU vs Planned Time
» Inventory Days of Supply versus Planned Inventory days of Supply
» Order accuracy - Net requirement versus order quantities in the planning horizon
» Ratio between median price of pro- ducts procured and the international median reference value
» Reports submitted on time (for all and separate reports)
» Supply adequacy - Net requirements are reflected in the Procurement pipeline to secure sufficient future supply.
» Supply Plan report; supplier performance report
» Vendor on time delivery (VOTD)
» Assess Supplier Performance Cycle Time - Actual Supplier performance Cycle time versus Supplier commitment cycle time

Training:	Qualifications Available:	Certifications Available:
» Contract Management<br>» Supplier Relationship Management (SRM)<br>» Analytical Skills<br>» Competitive Billing<br>» Negotiation<br>» Procurement/ RFQ Management	» Bachelor of Business Science<br>» Bachelor of Marketing and Business Science	» Occupational Certificate: Supply and Distribution Manager<br>» SPSM3 TM (Enterprise-Wide Procurement Influence)<br>» APICS Certified in production and inventory management certificate<br>» CIPS Level 5 Advanced Diploma in Procurement and Supply

Manager – Finance and Administration

Organisation:

SC-focused Organisation

Competency Level:	Serial Number:	Primary Process:	Job Role:	Supervises:
Managerial	5-56-64-4	Enable – Procurement	Manager – Finance and Administration	-

Contributions	Adhere to best practice in the management of financial resources of the Supply Chain. Be actively involved in the performance feedback to operations and assist in the formulation of approaches and methods to attain and maintain the level of efficiency specified in the Supply Chain Strategy
Technical Competency(ies):	2.1 Manage procurement costs and budget<br>2.3 Manage tendering processes and supplier agreements

2.1 Manage procurement costs and budget

- » Ensure the organisation follows public procurement regulations
- » Ensure adherence to the approved procurement budget
- » Evaluate financial and management accounting practices within procurement
- » Analyse commercial factors that contribute toward cost reduction, price savings and value improvement opportunities
- » Analyse the principles of foreign exchange rates and how they impact prices

2.3 Manage tendering processes and supplier agreements

- » Evaluate when to the use of request for qualifications (RFQ), invitations to bid (ITB) and request for proposals (RFP) methods
- » Demonstrate in-depth knowledge of local and international tendering procedures and analyse the procurement strategy and methods appropriate to special commodities and contextual situations
- » Determine the detailed specifications for tenders
- » Provide operational guidance for high-value and politically-sensitive procurements
- » Facilitate the process of developing and managing contracts
- » Ensure adherence to the procurement, return and exchange policies
- » Ensure adherence to the appropriate legislation and policy in regard to procurement processes, and what is required to comply
- » Demonstrate ethical behaviour, abiding by conflict of interest policies
- » Evaluate tender bids based on the specification and evaluation criteria including capability of the supplier, problems relating to outcomes of the contract.
- » Evaluate the contract approval process, including contract negotiations
- » Determine some of the key issues relevant to negotiating supply contracts (e.g., volume usage over time, price volume agreements, alternate supplier

	clauses, duration of contract, period of review, terms for contract termination and renewal)<br>» Award and manage contracts, resolving any issues as they arise<br>» Analyse the process of debriefing unsuccessful suppliers<br>» Analyse the ways in which compliance with purchasing policies and procedures is monitored<br>» Analyse procurement planning including a yearly activities schedule and developing a wide range of briefs, specifications and commercial documentation<br>» Analyse strategic sourcing projects, capturing benefits, work requirements, impacts and cost savings<br>» Play a leading role in cross-functional teams handling strategic procurements<br>» Participate in effective internal and external procurement networks<br>» Analyse local and international health commodity prices and understand the factors affecting them<br>» Engage clients across the public sector in presentations demonstrating the benefits of a range of strategic projects designed to ensure cost savings and value for money for government procurement
Personal/Management Competency(ies):	5.6 Manage financial activities<br>6.1 Demonstrate generic skills (e.g., literacy, numeracy, technology)<br>6.1.1 Exhibit high understanding of literacy and numeracy<br>6.2 Demonstrate strong communication skills<br>6.2.1 Practice cultural awareness<br>6.4 Exhibit professional and ethical values<br>6.4.1 Demonstrate integrity<br>6.4.2 Engage in continuous professional development
5.6 Manage financial activities	» Determine rules for managing all resources with care, applying budgetary principles<br>» Take ownership for meeting budgets and donor requirements<br>» Analyse budgets (national, regional, etc.) as necessary for work (e.g., wages budget, touring budget, stationery budget, project budgets for using NGO funds)<br>» Produce timely and clear financial reports for funders and donors<br>» Keep records following government budgeting, accounting and financial practices<br>» Maintain an overview of multiple budgets from multiple sources<br>» Seek and use information on financial funding requirements<br>» Analyse the process of ensuring timely decision making with regard to financial shortfalls<br>» Analyse budget tracking for logistics activities according to established procedures<br>» Analyse financial statements and explain components of a balance sheet and income statement

- Create interactive decision support models that allow the development of multiple scenarios and demonstrate the sensitivity of multiple independent variables
- Determine the success or failure of a business using financial accounting
- Evaluate the calculations of the total system cost of delivering a product or service to the customer
- Analyse the key considerations for developing a business plan
- Evaluate the structure against which business performance will be monitored (e.g., cost centres, chart of accounts)
- Analyse key performance indicators used to monitor business performance (e.g., turnover, profitability)
- Determine strategies for minimising the risk of fraudulent activity (e.g., reconciliation of purchase orders, receipts and payment approvals; dual signatory arrangements for funds transfers)
- Evaluate investigative processes and options for confirming the existence of fraudulent activity (e.g., sample audits, forensic accounting services)
- Prepare funding applications for trusts and grants
- Demonstrate the process of ensuring financial transparency
- Evaluate the general monetary value of medicines and equipment
- Secure financing and manage budgets to support distribution operations
- Demonstrate the ability to translate financial information into SC language
- Demonstrate SC finance knowledge

6.1 Demonstrate generic skills (e.g., literacy, numeracy, technology)

6.1.1 Exhibit high understanding of literacy and numeracy

- Encourage speaking clearly and confidently, organizing information in a logical manner, and considering voice tone and pace
- Demonstrate responding to verbal messages and other cues (active listening)
- Demonstrate expressing ideas and opinions clearly in written and verbal form
- Demonstrate communicating information accurately, concisely and confidently in writing and verbally
- Demonstrate a level of mathematics suitable to the job held
- Demonstrate knowledge of visual presentation techniques, including charting, histograms and flow sheets
- Demonstrate reading and comprehending at a level necessary to properly complete duties of the position
- Demonstrate the process of interpreting written information in documents, such as reports, SOPs, LMIS forms, graphs, calendars, schedules, notices and directions
- Demonstrate paying attention to detail and identifying the main ideas, detecting inconsistencies and identifying missing information in documents
- Demonstrate computer skills — such as using Word, Excel, PowerPoint and the internet — suitable to the job level

	» Demonstrate the process of analysing and validating KPIs<br>» Participate in the evaluation of activities that act as standards against which the KPIs are measured<br>» Demonstrate the process of performing critical self-review on work before submission
6.2 Demonstrate strong communication skills	» Demonstrate being truthful and trustworthy, and supplying accurate information at all times<br>» Demonstrate working as part of a workplace team<br>» Determine vocabulary and communication style and form for both written and verbal communication that is appropriate for the situation, audience and material being communicated (e.g., avoid unnecessary jargon, clearly explain medical and SCM terminology)<br>» Demonstrate open communication within constraints of confidentiality<br>» Analyse communications about logistics<br>» Demonstrate participation in meetings, expressing one's opinions, being aware of others' needs, and being appropriately assertive when required<br>» Demonstrate perspective into the point of view of others, understanding their needs and goals<br>» Demonstrate the practise of building trust, rapport and credibility with others<br>» Determine the communication needs and concerns of others and respond to them<br>» Demonstrate avoiding conflict between work and personal interests<br>» Demonstrate the practice of meaningful two-way communication<br>» Demonstrate influence on others by persuasively presenting thoughts and ideas<br>» Demonstrate assertiveness skills to deal with unreasonable requests and/or refusals that would compromise practice or consumer care<br>» Demonstrate a positive, supportive and appreciative attitude<br>» Demonstrate the ability to balance the organisation's needs and the customer's needs<br>» Demonstrate self-control by maintaining composure and keeping emotions in check, even in difficult situations; deal calmly and effectively with stressful situations<br>» Demonstrate the expressing of opinions and providing information in written and/or verbal form in a manner that does not elicit concern, anger or other adverse response<br>» Demonstrate the use of a systematic process for following up that demonstrates written reports have been received and understood<br>» Evaluate the means by which responses to input to the work environment are monitored<br>» Seek practical ways to overcome barriers to communication<br>» Ensure language representation (e.g., interpreters) at meetings when appropriate

	» Demonstrate tackling difficult situations and resolving disputes between staff<br>» Demonstrate giving both positive and negative feedback sensitively<br>» Demonstrate maintaining and improving communication<br>» Demonstrate the process of giving and requesting feedback frequently<br>» Demonstrate the ability to communicate effectively with shop stewards and union representatives
6.2.1 Practice cultural awareness	» Demonstrate cultural awareness and sensitivity, treating all people with fairness, respect and dignity<br>» Demonstrate anti-discriminatory practices in the organisation, including HR and disciplinary procedures<br>» Demonstrate cultural sensitivity, equality and fairness at all levels of the organisation<br>» Demonstrate challenging discriminatory behaviour directly and sensitively<br>» Demonstrate acting in a non-discriminatory way toward individuals and groups<br>» Demonstrate the process of integrating cultural awareness in learning and development approaches<br>» Demonstrate avoiding stereotypical responses by examining one's own behaviour and bias<br>» Demonstrate managing cultural diversity in teams and make the most of differences<br>» Demonstrate the use of strategies and/or resources for communicating effectively with people from different cultural backgrounds<br>» Supply information relating to values, beliefs and cultural backgrounds of consumers that may influence the way professional services are provided
6.4 Exhibit professional and ethical values	
6.4.1 Demonstrate integrity	» Demonstrate accepting responsibility for work tasks and performance<br>» Demonstrate working within a framework of clearly understood humanitarian values and ethics<br>» Demonstrate standing by decisions and holding others accountable when necessary<br>» Demonstrate not abusing one's own power or position<br>» Demonstrate the process of managing team members to ensure they do not abuse their power or position<br>» Determine when individuals or the organisation is straying from organisation goals, and challenge them to uphold ethics<br>» Demonstrate resisting undue political pressure in decision making<br>» Demonstrate supporting staff in maintaining ethical stances<br>» Demonstrate making time in team for ethical inquiry and reflection<br>» Demonstrate showing consistency between expressed principles and behaviour

» Ensure that principles, values and ethics are embedded in policy

» Demonstrate acting without consideration of personal gain

» Demonstrate promoting transparency in decision making structures and processes

» Ensure programmes are acting with integrity, and recognise the impact of not doing so

» Demonstrate recognising one's own limitations and act upon them

» Demonstrate all standard operating procedures

» Demonstrate working in a safe and legal way

» Demonstrate respect, dignity and consideration for consumers

» Determine the impact of a no-blame culture on reporting and preventing recurrence of incidents

» Evaluate requests of colleagues that might be regarded as unreasonable

» Demonstrate communicating (verbally and by example) expectations of the desired standards and approaches to be adopted

» Determine roles and responsibilities in terms of the position statement/duty statement of the position held

» Demonstrate punctuality

» Demonstrate applying professional care and expertise to deliver high quality of services

» Demonstrate care and attention to detail in undertaking work activities

» Determine appropriate attire and presentation for the role and situation

» Demonstrate recognising and taking responsibility for emotions

» Demonstrate an environment in which others can talk and act without fear of repercussion

» Demonstrate keeping commitments and not letting people down

» Demonstrate being trusting and cooperative when working alongside others

» Demonstrate taking on various responsibilities within the department as the need arises

6.4.2 Engage in continuous professional development

» Demonstrate seeking out opportunities to learn, and integrating new knowledge and skills into work

» Determine gaps in knowledge and skills for completing specific tasks, and develop a plan to acquire them

» Demonstrate listening to and inviting feedback from others on one's own performance

» Demonstrate using lifelong learning (continuous striving to gain knowledge and maintain competence) in the context of career development and the professional's role in delivering healthcare services

» Analyse organisational systems for capturing learning and ensuring lessons learned

» Employ reflective learning within the team, in which lessons are captured and integrated into future projects

	» Promote continuous learning as an integral part of organisational performance<br>» Demonstrate being open to new ideas and different perspectives<br>» Demonstrate keeping up to date in the place of work with input from supervisors<br>» Demonstrate documenting continual professional development activities<br>» Demonstrate the use of multiple techniques to acquire new knowledge and skills; process and retain information; and identify when it is necessary to acquire new knowledge and skills.<br>» Convene professional organisations and/or committees<br>» Determine and/or demonstrate quality improvement and/or quality assurance activities participated in<br>» Demonstrate reflecting on performance and seeking what needs improvement<br>» Demonstrate the process of engaging in personal career development, identifying occupational interests, strengths, options and opportunities<br>» Demonstrate contributing to others' professional development<br>» Demonstrate sharing experiences and learning internally and externally<br>» Demonstrate seeking out challenging projects outside of core experience and achieve solid results
Basal Technology Competency(ies):	7.1 Data Science<br>7.5 Planning systems<br>7.6 ERP includes function of LMIS<br>7.12 Basic Office Skills<br>7.13 Have a command of technology
7.1 Data Science	» Analyse end to end MIS technology components that are inherent in advanced MIS systems and explore how these may be relevant in different supply chain contexts<br>» Determine emerging technological approaches to data visualization and analysis<br>» Analyse how data science can be applied within supply chain<br>» Demonstrate the ability to interact, provide guidance for data (dashboard) consolidation in various levels and use the tech tools<br>» Support movement of data into electronic form at lowest/remote SC level<br>» Demonstrate the ability to work with relational database
7.5 Planning systems	» Analyse blockchain and understand the building blocks for planning systems<br>» Evaluate planning systems as a value-add capability for relevant global health use cases

7.6 ERP includes function of LMIS	» Evaluate a Master Data Management System<br>» Demonstrate an understanding of the critical components of an ERP system<br>» Demonstrate an understanding of the meaning of common document types found within an ERP/LMIS<br>» Demonstrate an understanding of the logic of data visibility within an ERP<br>» Demonstrate the importance of data integrity, record management and information security
7.12 Basic Office Skills	» Demonstrate a good understanding of common presentation authoring packages<br>» Demonstrate a good understanding of common spreadsheet authoring packages<br>» Demonstrate a good understanding of common document authoring packages<br>» Demonstrate a good understanding of common email authoring packages<br>» Demonstrate a good understanding of common instant messaging packages<br>» Demonstrate a good understanding of common video conferencing authoring packages
7.13 Have a command of technology	» Demonstrate the use of technology suitable to the job held<br>» Monitor new developments and technologies in the sector<br>» Experiment with new technologies and recognising potential benefits for the sector<br>» Ensure that resources and support are provided across the organisation to enable colleagues to make the best use of available technology<br>» Determine what the organisations' strategy is for technology use<br>» Determine how to minimise environmental damage through technology use<br>» Analyse the use of technology to maximise effectiveness and efficiency<br>» Analyse the use of field-based technology (e.g., radio, general packet radio service (GPRS), satellite phone)
Advanced Technology Competency(ies):	7.2 Blockchain<br>7.7 Automation<br>7.10 Internet of things<br>7.11 Cloud Computing
7.2 Blockchain	» Evaluate blockchain as a value-add capability for relevant global health use cases
7.7 Automation	» Evaluate automation as a value-add capability for relevant global health use cases

7.10 Internet of things (IoT)	» Evaluate the opportunity of IoT used in conjunction with AI<br>» Evaluate the internet of things as a value-add capability for relevant global health use cases
7.11 Cloud Computing	» Evaluate cloud computing as a value-add capability for relevant global health use cases

Key Performance Indicators:

- » Supply Plan report; supplier performance report
- » Order accuracy - Net requirement versus order quantities in the planning horizon
- » Ratio between median price of pro- ducts procured and the international median reference value
- » Days payable/receivable outstanding
- » Documentation Accuracy
- » % of Faultless Invoices
- » Order Management Costs
- » Supply Chain Asset utilisation and efficiency
- » Maintain Supply Chain Assets Register
- » Maintain Supply Chain Assets Availability
- » Cash to Cash Cycle (Cash to Use Cycle)
- » Inventory Turns
- » Inventory Days of Supply
- » Expense Ratios
- » Average inventory holding cost
- » Incremental ordering cost

Training:	Qualifications Available:	Certifications Available:
» Basic legal process<br>» Basic Supply Chain Finance<br>» ERP Systems Training<br>» Business Ethics/Conduct training<br>» Accounting<br>» Accounts Receivable<br>» International Financial Reporting Standards<br>» Utilizing Finance Systems<br>» Financial Planning	» Advanced Diploma in Logistics<br>» Master of Commerce in Strategic Management Accounting and Finance<br>» Bachelor of Commerce in Strategic Management Accounting and Finance	» CIPS Level 5 Advanced Diploma in Procurement and Supply<br>» APICS Certified supply chain professional certificate<br>» CPA/ACCA professional qualification

Manager – Data and Use

Organisation

SC-focused Organisation

Competency Level:	Serial Number:	Primary Process:	Job Role:	Supervises:
Managerial	7-71-55-76-3	Enable – Data Information	Manager – Data and Use	Officer – Data and Use, Officer – Data Management

Contributions	Responsible for IT architecture within the supply chain organisation. Including LMIS, accounting and supply chain management systems.
Basal Technology Competency(ies):	7.1 Data Science<br>7.6 ERP includes function of LMIS<br>7.12 Basic Office Skills<br>7.13 Have a command of technology

7.1 Data Science

- Describe the end to end MIS technology components that are inherent in advanced MIS systems and explore how these may be relevant in different supply chain contexts
- Identify emerging technological approaches to data visualization and analysis
- Identify how data science can be applied within supply chain
- Ability to interact, provide guidance for data (dashboard) consolidation in various levels and use the tech tools
- Support movement of data into electronic form at lowest/remote SC level
- Ability to work with relational database

7.6 ERP includes function of LMIS

- Support a Master Data Management System
- Describe the critical components of an ERP system
- Describe the meaning of common document types found within an ERP/LMIS
- Describe the logic of data visibility within an ERP
- Describe the importance of data integrity, record management and information security

7.12 Basic Office Skills

- Have a good understanding of common presentation authoring packages
- Have a good understanding of common spreadsheet authoring packages
- Have a good understanding of common document authoring packages
- Have a good understanding of common email authoring packages
- Have a good understanding of common instant messaging packages
- Have a good understanding of common video conferencing authoring packages

7.13 Have a command of technology	» Apply the use of technology suitable to the job held<br>» Provide input on monitoring new developments and technologies in the sector<br>» Identify new technologies and recognising potential benefits for the sector<br>» Identify resources and support that are provided across the organisation to enable colleagues to make the best use of available technology<br>» Ensure that the organisation has a strategy for technology use<br>» Describe how to minimise environmental damage through technology use<br>» Use technology to maximise effectiveness and efficiency<br>» Use field-based technology (e.g., radio, general packet radio service (GPRS), satellite phone)

Key Performance Indicators:

- Number of personnel with the required Skill, Education, Training and Aptitude as per Supply Chain Strategy versus Total number of personnel.
- Establishes and maintains IT infrastructure within system availability standards as specified in the IT Service Level Agreement e.g. Up time % over a period.
- Response - Number of closed Help desk requests over a period versus Help Desk Requests
- Effectiveness - Time to resolutions within Service Agreement Parameters. Number of Help Desk Request saved within the SLA versus total help desk requests
- Identification of root cause for help desk request. Classification of helpdesk requests into root causes of request. Maintain a remediation log for further actions to reduce the number of requests
- Propose remediation regarding systems, modules, training to enable personnel to support the Supply Chain performance requirements.
- Ensure security is enabled on all systems. Measure and report on security breaches.

Training:	Qualifications Available:	Certifications Available:
» Basics of supply chain management<br>» IT infrastructure management<br>» Project Management		» Advanced Certificate in Information Technology Governance<br>» Advanced Certificate in Information Technology in Support Services<br>» Lean Six Sigma Certification in Finance green belt

Manager – QA/ QMS

Organisation:

SC-focused Organisation

Competency Level:	Serial Number:	Primary Process:	Job Role:	Supervises:
Managerial	2-243-25-28-4	Enable – Risk	Manager – QA/ QMS	Officer – QA/ QC

Contributions	Responsible for the establishment and maintenance of the Quality Management System for the Public Heal System Organisation.
Technical Competency(ies):	2.4 Undertake contract management and risk<br>2.4.3 Addresses risk and ensures quality management<br>2.5 Ensure quality of products<br>2.8 Prepare for product supply during disasters and emergencies<br>2.9 Undertake or manage manufacturing or compounding of products

2.4 Undertake contract management and risk

2.4.3 Addresses risk and ensures quality management	» Provide risk assessments for the procurement process (technical, commercial, administrative)<br>» Analyse the key performance indicators for assessing procurement and supplier performance and effectiveness<br>» Determine any problems with the procurement of supplies in the supply chain<br>» Evaluate the contingency plan for shortages<br>» Analyse recommended options for improving the performance of suppliers<br>» Demonstrate procurement portfolio analysis and risk assessment (supply positioning)<br>» Demonstrate the fundamentals of risk planning and assessment
2.5 Ensure quality of products	» Analyse the use and monitoring process for prequalification of suppliers<br>» Evaluate the process of ensuring that health commodities are not counterfeit and meet quality standards through certification (ISO, WHO GMP<br>» Implement, conduct and maintain a reporting system of pharmacovigilance (e.g., report adverse drug reactions, quality defects, batch recall) and equipment faults<br>» Analyse the process of inspecting products when delivered and during storage to catch defects or problems before they are given to, or needed by, clients<br>» Demonstrate compliance with good distribution and good storage practices (temperature monitoring in the warehouse, transportation and distribution)

	» Evaluate product-specific shelf-life by which the product are acceptable or rejected (e.g. no less than 1 year for most of drugs, and less for short shelf-life like laboratory)
2.8 Prepare for product supply during disasters and emergencies	» Analyse communications with the national disaster planning team<br>» Analyse the procurement and logistic requirements for emergency and disaster supply<br>» Assess the local capacity before the emergency or disaster supply<br>» Analyse the factors that may restrict emergency or disaster supply to take place
2.9 Undertake or manage manufacturing or compounding of products	» Ensure adherence to the good manufacturing practice for pharmaceutical (GMP) medicine when compounding<br>» Demonstrate compliance with relevant pharmaceutical policies and regulations
Personal/Management Competency(ies):	5.5 Manage and plan projects<br>5.5.2 Execute strategic decision making<br>5.5.3 Manage partnerships<br>6.1 Demonstrate generic skills (e.g., literacy, numeracy, technology)<br>6.1.1 Exhibit high understanding of literacy and numeracy<br>6.2 Demonstrate strong communication skills<br>6.2.1 Practice cultural awareness
5.5 Manage and plan projects	
5.5.2 Execute strategic decision making	» Determine how one can make decisions regarding one's own workload and area of responsibility<br>» Determine the process of prioritizing competing tasks and perform them quickly and efficiently according to their importance; finding new ways of organizing or planning work to accomplish it more efficiently<br>» Evaluate the proper time and space for consultation around decisions being made.<br>» Demonstrate the process of considering the input of resources including staff needed<br>» Analyse assumptions against facts<br>» Determine the key issues in a complex situation and come to the heart of the problem quickly<br>» Evaluate relevant information before making decisions<br>» Provide input to strategic decisions in the interest of the organisation's goals<br>» Evaluate when to analyse, evaluate and execute tough decisions when necessary<br>» Analyse the impact of decisions on others' work or team goals<br>» Communicate decisions and ensure they are incorporated into policies and processes<br>» Demonstrate inclusive and consultative strategic planning to establish strategic objectives

	» Evaluate emerging trends and practices<br>» Analyse the macro and long-term consequences of decisions
5.5.3 Manage partnerships	» Demonstrate the concepts of partnership working<br>» Determine the process of supporting implementation of partnership programmes<br>» Analyse the process of experimenting with and trialling new ideas with partners<br>» Analyse partnerships to deliver programme and increase impact<br>» Analyse innovation and creativity in partnership work<br>» Communicate key information with partner members<br>» Involve and value partners in all aspects of programming<br>» Analyse problems with supply chain relationships<br>» Demonstrate the process of communicating effectively with nurses, doctors and other members of the healthcare team<br>» Analyse the process of responding to consumer complaints or comments about services and/or advice received<br>» Analyse collaboration and consultation across the sector and encourage the team to work with others to gain market insight<br>» Encourage input from other key actors in the humanitarian sector and develop relationships with a cross-section of actors<br>» Analyse the process of meeting the reporting requirements of vertical programmes<br>» Analyse the structure of the health system at a national level and explain this to others<br>» Analyse the roles of other members of the healthcare team (including with consumers) in a way that engenders understanding and confidence in the team and its members<br>» Analyse the structure of the organisation, environment and/or service in which they work<br>» Demonstrate how vertical programmes work within the health system<br>» Evaluate the communication network established to achieve work outcomes<br>» Demonstrate knowledge of the structure of the health system at the provincial/regional level and explain this to others<br>» Analyse the roles and functions of government agencies in regulating and supporting supply chain organisations<br>» Demonstrate knowledge of change management (e.g. PtD Theory of Change)
6.1 Demonstrate generic skills (e.g., literacy, numeracy, technology)	

6.1.1 Exhibit high understanding of literacy and numeracy	» Encourage speaking clearly and confidently, organizing information in a logical manner, and considering voice tone and pace<br>» Demonstrate responding to verbal messages and other cues (active listening)<br>» Demonstrate expressing ideas and opinions clearly in written and verbal form<br>» Demonstrate communicating information accurately, concisely and confidently in writing and verbally<br>» Demonstrate a level of mathematics suitable to the job held<br>» Demonstrate knowledge of visual presentation techniques, including charting, histograms and flow sheets<br>» Demonstrate reading and comprehending at a level necessary to properly complete duties of the position<br>» Demonstrate the process of interpreting written information in documents, such as reports, SOPs, LMIS forms, graphs, calendars, schedules, notices and directions<br>» Demonstrate paying attention to detail and identifying the main ideas, detecting inconsistencies and identifying missing information in documents<br>» Demonstrate computer skills — such as using Word, Excel, PowerPoint and the internet — suitable to the job level<br>» Demonstrate the process of analysing and validating KPIs<br>» Participate in the evaluation of activities that act as standards against which the KPIs are measured<br>» Demonstrate the process of performing critical self-review on work before submission
6.2 Demonstrate strong communication skills	» Demonstrate being truthful and trustworthy, and supplying accurate information at all times<br>» Demonstrate working as part of a workplace team<br>» Determine vocabulary and communication style and form for both written and verbal communication that is appropriate for the situation, audience and material being communicated (e.g., avoid unnecessary jargon, clearly explain medical and SCM terminology)<br>» Demonstrate open communication within constraints of confidentiality<br>» Analyse communications about logistics<br>» Demonstrate participation in meetings, expressing one's opinions, being aware of others' needs, and being appropriately assertive when required<br>» Demonstrate perspective into the point of view of others, understanding their needs and goals<br>» Demonstrate the practise of building trust, rapport and credibility with others<br>» Determine the communication needs and concerns of others and respond to them<br>» Demonstrate avoiding conflict between work and personal interests<br>» Demonstrate the practice of meaningful two-way communication

- Demonstrate influence on others by persuasively presenting thoughts and ideas
- Demonstrate assertiveness skills to deal with unreasonable requests and/or refusals that would compromise practice or consumer care
- Demonstrate a positive, supportive and appreciative attitude
- Demonstrate the ability to balance the organisation's needs and the customer's needs
- Demonstrate self-control by maintaining composure and keeping emotions in check, even in difficult situations; deal calmly and effectively with stressful situations
- Demonstrate the expressing of opinions and providing information in written and/or verbal form in a manner that does not elicit concern, anger or other adverse response
- Demonstrate the use of a systematic process for following up that demonstrates written reports have been received and understood
- Evaluate the means by which responses to input to the work environment are monitored
- Seek practical ways to overcome barriers to communication
- Ensure language representation (e.g., interpreters) at meetings when appropriate
- Demonstrate tackling difficult situations and resolving disputes between staff
- Demonstrate giving both positive and negative feedback sensitively
- Demonstrate maintaining and improving communication
- Demonstrate the process of giving and requesting feedback frequently
- Demonstrate the ability to communicate effectively with shop stewards and union representatives

6.2.1 Practice cultural awareness

- Demonstrate cultural awareness and sensitivity, treating all people with fairness, respect and dignity
- Demonstrate anti-discriminatory practices in the organisation, including HR and disciplinary procedures
- Demonstrate cultural sensitivity, equality and fairness at all levels of the organisation
- Demonstrate challenging discriminatory behaviour directly and sensitively
- Demonstrate acting in a non-discriminatory way toward individuals and groups
- Demonstrate the process of integrating cultural awareness in learning and development approaches
- Demonstrate avoiding stereotypical responses by examining one's own behaviour and bias
- Demonstrate managing cultural diversity in teams and make the most of differences
- Demonstrate the use of strategies and/or resources for communicating effectively with people from different cultural backgrounds

	» Supply information relating to values, beliefs and cultural backgrounds of consumers that may influence the way professional services are provided
Basal Technology Competency(ies):	7.6 ERP includes function of LMIS<br>7.12 Basic Office Skills<br>7.13 Have a command of technology
**7.6 ERP includes function of LMIS**	» Evaluate a Master Data Management System<br>» Demonstrate an understanding of the critical components of an ERP system<br>» Demonstrate an understanding of the meaning of common document types found within an ERP/LMIS<br>» Demonstrate an understanding of the logic of data visibility within an ERP<br>» Demonstrate the importance of data integrity, record management and information security
**7.12 Basic Office Skills**	» Demonstrate a good understanding of common presentation authoring packages<br>» Demonstrate a good understanding of common spreadsheet authoring packages<br>» Demonstrate a good understanding of common document authoring packages<br>» Demonstrate a good understanding of common email authoring packages<br>» Demonstrate a good understanding of common instant messaging packages<br>» Demonstrate a good understanding of common video conferencing authoring packages
**7.13 Have a command of technology**	» Demonstrate the use of technology suitable to the job held<br>» Monitor new developments and technologies in the sector<br>» Experiment with new technologies and recognising potential benefits for the sector<br>» Ensure that resources and support are provided across the organisation to enable colleagues to make the best use of available technology<br>» Determine what the organisations' strategy is for technology use<br>» Determine how to minimise environmental damage through technology use<br>» Analyse the use of technology to maximise effectiveness and efficiency<br>» Analyse the use of field-based technology (e.g., radio, general packet radio service (GPRS), satellite phone)

Advanced Technology Competency(ies):	7.2 Blockchain<br>7.11 Cloud Computing
7.2 Blockchain	» Evaluate blockchain as a value-add capability for relevant global health use cases
7.11 Cloud Computing	» Evaluate cloud computing as a value-add capability for relevant global health use cases

Key Performance Indicators:

- Accuracy of Risk Register
- Cycle time of QA/QC report
- Overall value at risk
- Partner/Donor integration, policy, guidelines and regulatory reforms
- QA/QC report
- Risk / Mitigation Costs
- Time to Recovery (TTR)
- # Batch recall/quarantine with corrective action
- # Suppliers contracted with meeting required certifications
- % Shipment received at expected delivery time

Training:	Qualifications Available:	Certifications Available:
» Quality assurance & quality control<br>» Risk and exception management<br>» Process Auditing	» Advanced Diploma in Pharmaceutical Sciences<br>» Advanced Diploma in Shipping and Logistics<br>» Bachelor of in Pharmaceutical Sciences<br>» Bachelor of Science Honours: Chemistry, Quality Assurance and Drug Regulation	» SPSM3 TM (Enterprise-Wide Procurement Influence)<br>» APICS Certified in production and inventory management certificate<br>» CIPS Level 5 Advanced Diploma in Procurement and Supply<br>» Occupational Certificate: Health Information Manager<br>» Pharmaceutical Policy and Management

Manager – HR/ Workforce Development

Organisation:

SC-focused Organisation

Competency Level:	Serial Number:	Primary Process:	Job Role:	Supervises:
Managerial	5-571-572-573-4	Enable – Human Resources	Manager – HR/ Workforce Development	-

Contributions	Be familiar with the skills and competencies required to execute the Supply Chain Strategy of the Public Health System Organisation. Formulate a plan to ensure the skills and competency Human Resource base is kept up to date with the Supply Chain Requirements. Develop evaluation methodologies to validate the competency and skills installed base. Establish and maintain a catalogue of education and skill development resources to support the maintenance and enhancement of the Human Resource capital of the Supply Chain.
Personal/Management Competency(ies):	5.5 Manage and plan projects<br>5.5.2 Execute strategic decision making<br>5.5.3 Manage partnerships<br>5.5.4 Direct/participate in teamwork<br>5.7 Oversee/support human resources (e.g., recruitment, training, team management/ supervision)<br>5.7.1 Manage staff recruitment process<br>5.7.2 Train staff<br>5.7.3 Supervise staff<br>5.7.4 Assessing HR systems<br>6.1 Demonstrate generic skills (e.g., literacy, numeracy, technology)<br>6.1.1 Exhibit high understanding of literacy and numeracy<br>6.2 Demonstrate strong communication skills<br>6.2.1 Practice cultural awareness

5.5 Manage and plan projects

5.5.2 Execute strategic decision making	» Determine how one can make decisions regarding one's own workload and area of responsibility<br>» Determine the process of prioritizing competing tasks and perform them quickly and efficiently according to their importance; finding new ways of organizing or planning work to accomplish it more efficiently<br>» Evaluate the proper time and space for consultation around decisions being made.<br>» Demonstrate the process of considering the input of resources including staff needed

	» Analyse assumptions against facts<br>» Determine the key issues in a complex situation and come to the heart of the problem quickly<br>» Evaluate relevant information before making decisions<br>» Provide input to strategic decisions in the interest of the organisation's goals<br>» Evaluate when to analyse, evaluate and execute tough decisions when necessary<br>» Analyse the impact of decisions on others' work or team goals<br>» Communicate decisions and ensure they are incorporated into policies and processes<br>» Demonstrate inclusive and consultative strategic planning to establish strategic objectives<br>» Evaluate emerging trends and practices<br>» Analyse the macro and long-term consequences of decisions
5.5.3 Manage partnerships	» Demonstrate the concepts of partnership working<br>» Determine the process of supporting implementation of partnership programmes<br>» Analyse the process of experimenting with and trialling new ideas with partners<br>» Analyse partnerships to deliver programme and increase impact<br>» Analyse innovation and creativity in partnership work<br>» Communicate key information with partner members<br>» Involve and value partners in all aspects of programming<br>» Analyse problems with supply chain relationships<br>» Demonstrate the process of communicating effectively with nurses, doctors and other members of the healthcare team<br>» Analyse the process of responding to consumer complaints or comments about services and/or advice received<br>» Analyse collaboration and consultation across the sector and encourage the team to work with others to gain market insight<br>» Encourage input from other key actors in the humanitarian sector and develop relationships with a cross-section of actors<br>» Analyse the process of meeting the reporting requirements of vertical programmes<br>» Analyse the structure of the health system at a national level and explain this to others<br>» Analyse the roles of other members of the healthcare team (including with consumers) in a way that engenders understanding and confidence in the team and its members<br>» Analyse the structure of the organisation, environment and/or service in which they work<br>» Demonstrate how vertical programmes work within the health system<br>» Evaluate the communication network established to achieve work outcomes<br>» Demonstrate knowledge of the structure of the health system at the provincial/regional level and explain this to others

	» Analyse the roles and functions of government agencies in regulating and supporting supply chain organisations<br>» Demonstrate knowledge of change management (e.g. PtD Theory of Change)
5.5.4 Direct/participate in teamwork	» Work with team members within their area of authority to establish achievable goals and strategies that are consistent with the objectives established for the organisation as a whole<br>» Evaluate an organisational chart that shows the lines of reporting and responsibility among staff<br>» Determine a culture of actively contributing a perspective and making a positive contribution to team-based problem solving and decision making<br>» Determine a culture of providing feedback, encouragement and support to team members for progressing strategic goals<br>» Monitor team performance in relation to the organisation's mission and goals<br>» Enable the creation of a culture of teams thinking for themselves and resolving problems<br>» Demonstrate the process of encouraging and harnessing diversity within the team to boost team effectiveness<br>» Demonstrate the process of encouraging and supporting the team to work through its stages of development and perform well<br>» Determine roles and responsibilities in relation to employees' expertise and the expectations of collaborating team members<br>» Demonstrate where their position fits in the structure (organisation, SC, healthcare sector) , the responsibilities their position entails and the accountabilities linked to the position
5.7 Oversee/support human resources (e.g., recruitment, training, team management/ supervision)	
5.7.1 Manage staff recruitment process	» Demonstrate use of a system for staff recruitment, appraisals and monitoring<br>» Evaluate training and recruitment plans to fill any identified gaps and shortages<br>» Determine relevant and appropriate selection criteria for a defined role<br>» Analyse and demonstrate the standard selection documentation for recruitment<br>» Determine key issues impacting the size and membership of an interview panel (e.g., logistics, gender balance, absence of conflict of interest)<br>» Analyse requirements for a fair, defensible and balanced interview process<br>» Analyse and demonstrate the documentation maintained for each interviewed position<br>» Determine due process for formalising an appointment that minimises the opportunity for untoward effects (e.g., appeal, conflict, resignation)<br>» Analyse job descriptions to obtain and retain skilled staff

	» Consult with and involve relevant personnel in development and updating of role descriptions/duty statements that clarify the duties and responsibilities of positions<br>» Determine the process of orienting new staff to the workplace, explaining standard operating systems and procedures<br>» Evaluate human resource plans to meet the future staffing needs of the organisation<br>» Evaluate key features and measures used for monitoring a personnel retention strategy (e.g., recruitment costs, staff turnover, staff surveys)<br>» Determine the legislative issues impacting human resource policies and procedures<br>» Evaluate the process of maintaining employee master data including medicals, allergies to ensure safe working environment<br>» Analyse an employee opinion survey
5.7.2 Train staff	» Determine the process of maintaining a list of staff that require logistics system training (based on supportive supervision reports)<br>» Coordinate all training in managing the logistics system for all health facility staff<br>» Promote participation in relevant learning and development opportunities<br>» Demonstrate the process of assisting in training nurses and other health professionals in medicines ordering and storage procedures<br>» Demonstrate the process of orienting new doctors to the formulary and the systems and procedures of the medicines supply system<br>» Determine the process of providing on-the-job training to district health officials and hospital pharmacists as needed<br>» Demonstrate the process of ensuring staff have the necessary skills and understanding for safe practice in the event they need to fill a management role due to absence or illness<br>» Evaluate the process of staff handling hazardous materials receive training on safety & compliance regulations<br>» Evaluate any staff needs at all levels of the supply chain<br>» Analyse key factors important for initiating and/or sustaining the motivation of adult learners<br>» Evaluate the variable learning needs and styles or modalities in adult learners (e.g., visual versus auditory)<br>» Evaluate a range of strategies that could be used to reinforce and clarify educational content (e.g., tutorials or workshops, written materials, intranet- or internet-based resources)<br>» Evaluate the use of train-the-trainer to equipment trainers<br>» Analyse training effectiveness monitoring and training feedback systems
5.7.3 Supervise staff	» Determine the use of a performance management process with all personnel to ensure continuous improvement<br>» Determine an environment that is supportive of learning and professional development

	» Demonstrate the nature of the supervisory role, what is meant by direct supervision, and where responsibility for outputs and outcomes rests<br>» Plan and conduct supervisory tours of dependent facilities<br>» Analyse situations where supervised personnel are experiencing difficulties in completing work activities and/or where a mandatory notification obligation exists<br>» Analyse signs/cues from supervised personnel that indicate additional guidance or support is needed (e.g., hesitancy, distress, seeks clarification from less authoritative sources)<br>» Demonstrate a practise of providing feedback, encouragement and support to team members<br>» Analyse areas where performance should/could be improved and provide resources to support this<br>» Demonstrate the process of discussing expectations, achievements and contributions with personnel in a fair and equitable manner<br>» Support colleagues in creating a professional development plan and suggest ways the plan may be progressed through relevant training and/or experiential learning opportunities<br>» Demonstrate a culture of encouraging and supporting high-potential employees to accept growth challenges outside their own areas<br>» Demonstrate the process of providing progression opportunities for employees who have prepared themselves through development<br>» Evaluate the process of identifying and dealing with unproductive staff using an approved process in an ethical manner<br>» Evaluate and address work issues contributing to impairment of personnel (e.g., excessive workload, conflict)<br>» Determine the complaints management process<br>» Evaluate situations where additional expertise should be sought (e.g., counselling in the event of workplace violence or in situations of diminished performance due to drug or alcohol abuse)<br>» Demonstrate a culture of acknowledging and respecting different working styles
5.7.4 Assessing HR systems	» Ability to assess HR systems
6.1 Demonstrate generic skills (e.g., literacy, numeracy, technology)	
6.1.1 Exhibit high understanding of literacy and numeracy	» Encourage speaking clearly and confidently, organizing information in a logical manner, and considering voice tone and pace<br>» Demonstrate responding to verbal messages and other cues (active listening)<br>» Demonstrate expressing ideas and opinions clearly in written and verbal form<br>» Demonstrate communicating information accurately, concisely and confidently in writing and verbally<br>» Demonstrate a level of mathematics suitable to the job held<br>» Demonstrate knowledge of visual presentation techniques, including charting, histograms and flow sheets

» Demonstrate reading and comprehending at a level necessary to properly complete duties of the position

» Demonstrate the process of interpreting written information in documents, such as reports, SOPs, LMIS forms, graphs, calendars, schedules, notices and directions

» Demonstrate paying attention to detail and identifying the main ideas, detecting inconsistencies and identifying missing information in documents

» Demonstrate computer skills — such as using Word, Excel, PowerPoint and the internet — suitable to the job level

» Demonstrate the process of analysing and validating KPIs

» Participate in the evaluation of activities that act as standards against which the KPIs are measured

» Demonstrate the process of performing critical self-review on work before submission

6.2 Demonstrate strong communication skills

» Demonstrate being truthful and trustworthy, and supplying accurate information at all times

» Demonstrate working as part of a workplace team

» Determine vocabulary and communication style and form for both written and verbal communication that is appropriate for the situation, audience and material being communicated (e.g., avoid unnecessary jargon, clearly explain medical and SCM terminology)

» Demonstrate open communication within constraints of confidentiality

» Analyse communications about logistics

» Demonstrate participation in meetings, expressing one’s opinions, being aware of others’ needs, and being appropriately assertive when required

» Demonstrate perspective into the point of view of others, understanding their needs and goals

» Demonstrate the practise of building trust, rapport and credibility with others

» Determine the communication needs and concerns of others and respond to them

» Demonstrate avoiding conflict between work and personal interests

» Demonstrate the practice of meaningful two-way communication

» Demonstrate influence on others by persuasively presenting thoughts and ideas

» Demonstrate assertiveness skills to deal with unreasonable requests and/or refusals that would compromise practice or consumer care

» Demonstrate a positive, supportive and appreciative attitude

» Demonstrate the ability to balance the organisation’s needs and the customer’s needs

» Demonstrate self-control by maintaining composure and keeping emotions in check, even in difficult situations; deal calmly and effectively with stressful situations

» Demonstrate the expressing of opinions and providing information in written and/or verbal form in a manner that does not elicit concern, anger or other adverse response

	» Demonstrate the use of a systematic process for following up that demonstrates written reports have been received and understood<br>» Evaluate the means by which responses to input to the work environment are monitored<br>» Seek practical ways to overcome barriers to communication<br>» Ensure language representation (e.g., interpreters) at meetings when appropriate<br>» Demonstrate tackling difficult situations and resolving disputes between staff<br>» Demonstrate giving both positive and negative feedback sensitively<br>» Demonstrate maintaining and improving communication<br>» Demonstrate the process of giving and requesting feedback frequently<br>» Demonstrate the ability to communicate effectively with shop stewards and union representatives
6.2.1 Practice cultural awareness	» Demonstrate cultural awareness and sensitivity, treating all people with fairness, respect and dignity<br>» Demonstrate anti-discriminatory practices in the organisation, including HR and disciplinary procedures<br>» Demonstrate cultural sensitivity, equality and fairness at all levels of the organisation<br>» Demonstrate challenging discriminatory behaviour directly and sensitively<br>» Demonstrate acting in a non-discriminatory way toward individuals and groups<br>» Demonstrate the process of integrating cultural awareness in learning and development approaches<br>» Demonstrate avoiding stereotypical responses by examining one's own behaviour and bias<br>» Demonstrate managing cultural diversity in teams and make the most of differences<br>» Demonstrate the use of strategies and/or resources for communicating effectively with people from different cultural backgrounds<br>» Supply information relating to values, beliefs and cultural backgrounds of consumers that may influence the way professional services are provided
Basal Technology Competency(ies):	7.12 Basic Office Skills<br>7.13 Have a command of technology
7.12 Basic Office Skills	» Demonstrate a good understanding of common presentation authoring packages<br>» Demonstrate a good understanding of common spreadsheet authoring packages<br>» Demonstrate a good understanding of common document authoring packages<br>» Demonstrate a good understanding of common email authoring packages<br>» Demonstrate a good understanding of common instant messaging packages<br>» Demonstrate a good understanding of common video conferencing authoring packages

7.13 Have a command of technology	» Demonstrate the use of technology suitable to the job held<br>» Monitor new developments and technologies in the sector<br>» Experiment with new technologies and recognising potential benefits for the sector<br>» Ensure that resources and support are provided across the organisation to enable colleagues to make the best use of available technology<br>» Determine what the organisations' strategy is for technology use<br>» Determine how to minimise environmental damage through technology use<br>» Analyse the use of technology to maximise effectiveness and efficiency<br>» Analyse the use of field-based technology (e.g., radio, general packet radio service (GPRS), satellite phone)
Advanced Technology Competency(ies):	7.11 Cloud Computing
7.11 Cloud Computing	» Evaluate cloud computing as a value-add capability for relevant global health use cases

Key Performance Indicators:

- Planned skills upliftment plan - Number of personnel per competency versus actual skills upliftment number of personnel per competency.
- Employee engagement survey

Training:	Qualifications Available:	Certifications Available:
» Performance Management<br>» Contract Management<br>» EHS regulations<br>» Legislation and Standards<br>» Outsourcing	» Diploma in Management of Training<br>» Diploma: Human Resource Management	» Occupational Certificate: Health Information Manager

Lead – Compliance / Internal Audit

Organisation:

SC-focused Organisation

Competency Level:	Serial Number:	Primary Process:	Job Role:	Supervises:
Managerial	5-531-56-4	Enable – Regulatory (Individual) Compliance	Lead – Compliance / Internal Audit	-

Contributions	Be cognisant of the Supply Chain Strategy and Operating Model of the Public Health System Organisation. Ensure that the internal controls built into the processes deliver a reliable consistent quality result.
Personal/Management Competency(ies):	5.3 Implement risk management and monitoring and evaluation activities for the supply chain<br>5.3.1 Ensure monitoring and evaluation activities are completed<br>5.5 Manage and plan projects<br>5.5.4 Direct/participate in teamwork<br>5.6 Manage financial activities<br>6.1 Demonstrate generic skills (e.g., literacy, numeracy, technology)<br>6.1.1 Exhibit high understanding of literacy and numeracy<br>6.2 Demonstrate strong communication skills<br>6.2.1 Practice cultural awareness<br>6.4 Exhibit professional and ethical values<br>6.4.1 Demonstrate integrity<br>6.4.2 Engage in continuous professional development

5.3 Implement risk management and monitoring and evaluation activities for the supply chain

5.3.1 Ensure monitoring and evaluation activities are completed	» Perform periodic evaluations of pertinent information — such as problem symptoms from knowledgeable sources, carrying these through to the problems, potential causes and root causes of the problem<br>» Determine the process of quantifying the operational performance of similar companies and establish internal targets based on best-in-class results<br>» Analyse key performance measurements and continuous process improvement initiatives to improve process quality on a continual basis<br>» Analyse the results of the analyses provided

	» Determine the process of controlling and checking errors, taking corrective action so deviation from standards are minimised and the organisation's goals are achieved<br>» Analyse the use of appropriate technological developments to improve the system<br>» Demonstrate that continuous process improvement is an accepted way of organisational life<br>» Demonstrate ways of eliminating unnecessary steps in system design<br>» Analyse processes that strive to eliminate waste<br>» Review processes to encourage sustainability (e.g., reducing carbon footprint, establishing a paperless office, using renewable energy)<br>» Demonstrate the systematic approach used to close process or system performance gaps through streamlining and cycle time reduction, and identify and eliminate causes of quality below specifications, process variation and non-value-adding activities<br>» Determine the relationship between technology and process functionality<br>» Determine how policy, guidelines and regulatory reforms impact SC performance
5.5 Manage and plan projects	
5.5.4 Direct/participate in teamwork	» Work with team members within their area of authority to establish achievable goals and strategies that are consistent with the objectives established for the organisation as a whole<br>» Evaluate an organisational chart that shows the lines of reporting and responsibility among staff<br>» Determine a culture of actively contributing a perspective and making a positive contribution to team-based problem solving and decision making<br>» Determine a culture of providing feedback, encouragement and support to team members for progressing strategic goals<br>» Monitor team performance in relation to the organisation's mission and goals<br>» Enable the creation of a culture of teams thinking for themselves and resolving problems<br>» Demonstrate the process of encouraging and harnessing diversity within the team to boost team effectiveness<br>» Demonstrate the process of encouraging and supporting the team to work through its stages of development and perform well<br>» Determine roles and responsibilities in relation to employees' expertise and the expectations of collaborating team members<br>» Demonstrate where their position fits in the structure (organisation, SC, healthcare sector) , the responsibilities their position entails and the accountabilities linked to the position
5.6 Manage financial activities	» Determine rules for managing all resources with care, applying budgetary principles<br>» Take ownership for meeting budgets and donor requirements<br>» Analyse budgets (national, regional, etc.) as necessary for work (e.g., wages budget, touring budget, stationery budget, project budgets for using NGO funds)

- Produce timely and clear financial reports for funders and donors
- Keep records following government budgeting, accounting and financial practices
- Maintain an overview of multiple budgets from multiple sources
- Seek and use information on financial funding requirements
- Analyse the process of ensuring timely decision making with regard to financial shortfalls
- Analyse budget tracking for logistics activities according to established procedures
- Analyse financial statements and explain components of a balance sheet and income statement
- Create interactive decision support models that allow the development of multiple scenarios and demonstrate the sensitivity of multiple independent variables
- Determine the success or failure of a business using financial accounting
- Evaluate the calculations of the total system cost of delivering a product or service to the customer
- Analyse the key considerations for developing a business plan
- Evaluate the structure against which business performance will be monitored (e.g., cost centres, chart of accounts)
- Analyse key performance indicators used to monitor business performance (e.g., turnover, profitability)
- Determine strategies for minimising the risk of fraudulent activity (e.g., reconciliation of purchase orders, receipts and payment approvals; dual signatory arrangements for funds transfers)
- Evaluate investigative processes and options for confirming the existence of fraudulent activity (e.g., sample audits, forensic accounting services)
- Prepare funding applications for trusts and grants
- Demonstrate the process of ensuring financial transparency
- Evaluate the general monetary value of medicines and equipment
- Secure financing and manage budgets to support distribution operations
- Demonstrate the ability to translate financial information into SC language
- Demonstrate SC finance knowledge

6.1 Demonstrate generic skills (e.g., literacy, numeracy, technology)

6.1.1 Exhibit high understanding of literacy and numeracy

- Encourage speaking clearly and confidently, organizing information in a logical manner, and considering voice tone and pace
- Demonstrate responding to verbal messages and other cues (active listening)
- Demonstrate expressing ideas and opinions clearly in written and verbal form
- Demonstrate communicating information accurately, concisely and confidently in writing and verbally

» Demonstrate a level of mathematics suitable to the job held

» Demonstrate knowledge of visual presentation techniques, including charting, histograms and flow sheets

» Demonstrate reading and comprehending at a level necessary to properly complete duties of the position

» Demonstrate the process of interpreting written information in documents, such as reports, SOPs, LMIS forms, graphs, calendars, schedules, notices and directions

» Demonstrate paying attention to detail and identifying the main ideas, detecting inconsistencies and identifying missing information in documents

» Demonstrate computer skills — such as using Word, Excel, PowerPoint and the internet — suitable to the job level

» Demonstrate the process of analysing and validating KPIs

» Participate in the evaluation of activities that act as standards against which the KPIs are measured

» Demonstrate the process of performing critical self-review on work before submission

6.2 Demonstrate strong communication skills

» Demonstrate being truthful and trustworthy, and supplying accurate information at all times

» Demonstrate working as part of a workplace team

» Determine vocabulary and communication style and form for both written and verbal communication that is appropriate for the situation, audience and material being communicated (e.g., avoid unnecessary jargon, clearly explain medical and SCM terminology)

» Demonstrate open communication within constraints of confidentiality

» Analyse communications about logistics

» Demonstrate participation in meetings, expressing one's opinions, being aware of others' needs, and being appropriately assertive when required

» Demonstrate perspective into the point of view of others, understanding their needs and goals

» Demonstrate the practise of building trust, rapport and credibility with others

» Determine the communication needs and concerns of others and respond to them

» Demonstrate avoiding conflict between work and personal interests

» Demonstrate the practice of meaningful two-way communication

» Demonstrate influence on others by persuasively presenting thoughts and ideas

» Demonstrate assertiveness skills to deal with unreasonable requests and/or refusals that would compromise practice or consumer care

» Demonstrate a positive, supportive and appreciative attitude

» Demonstrate the ability to balance the organisation's needs and the customer's needs

» Demonstrate self-control by maintaining composure and keeping emotions in check, even in difficult situations; deal calmly and effectively with stressful situations

	» Demonstrate the expressing of opinions and providing information in written and/or verbal form in a manner that does not elicit concern, anger or other adverse response<br>» Demonstrate the use of a systematic process for following up that demonstrates written reports have been received and understood<br>» Evaluate the means by which responses to input to the work environment are monitored<br>» Seek practical ways to overcome barriers to communication<br>» Ensure language representation (e.g., interpreters) at meetings when appropriate<br>» Demonstrate tackling difficult situations and resolving disputes between staff<br>» Demonstrate giving both positive and negative feedback sensitively<br>» Demonstrate maintaining and improving communication<br>» Demonstrate the process of giving and requesting feedback frequently<br>» Demonstrate the ability to communicate effectively with shop stewards and union representatives
6.2.1 Practice cultural awareness	» Demonstrate cultural awareness and sensitivity, treating all people with fairness, respect and dignity<br>» Demonstrate anti-discriminatory practices in the organisation, including HR and disciplinary procedures<br>» Demonstrate cultural sensitivity, equality and fairness at all levels of the organisation<br>» Demonstrate challenging discriminatory behaviour directly and sensitively<br>» Demonstrate acting in a non-discriminatory way toward individuals and groups<br>» Demonstrate the process of integrating cultural awareness in learning and development approaches<br>» Demonstrate avoiding stereotypical responses by examining one’s own behaviour and bias<br>» Demonstrate managing cultural diversity in teams and make the most of differences<br>» Demonstrate the use of strategies and/or resources for communicating effectively with people from different cultural backgrounds<br>» Supply information relating to values, beliefs and cultural backgrounds of consumers that may influence the way professional services are provided
6.4 Exhibit professional and ethical values	
6.4.1 Demonstrate integrity	» Demonstrate accepting responsibility for work tasks and performance<br>» Demonstrate working within a framework of clearly understood humanitarian values and ethics<br>» Demonstrate standing by decisions and holding others accountable when necessary<br>» Demonstrate not abusing one’s own power or position

» Demonstrate the process of managing team members to ensure they do not abuse their power or position
» Determine when individuals or the organisation is straying from organisation goals, and challenge them to uphold ethics
» Demonstrate resisting undue political pressure in decision making
» Demonstrate supporting staff in maintaining ethical stances
» Demonstrate making time in team for ethical inquiry and reflection
» Demonstrate showing consistency between expressed principles and behaviour
» Ensure that principles, values and ethics are embedded in policy
» Demonstrate acting without consideration of personal gain
» Demonstrate promoting transparency in decision making structures and processes
» Ensure programmes are acting with integrity, and recognise the impact of not doing so
» Demonstrate recognising one's own limitations and act upon them
» Demonstrate all standard operating procedures
» Demonstrate working in a safe and legal way
» Demonstrate respect, dignity and consideration for consumers
» Determine the impact of a no-blame culture on reporting and preventing recurrence of incidents
» Evaluate requests of colleagues that might be regarded as unreasonable
» Demonstrate communicating (verbally and by example) expectations of the desired standards and approaches to be adopted
» Determine roles and responsibilities in terms of the position statement/duty statement of the position held
» Demonstrate punctuality
» Demonstrate applying professional care and expertise to deliver high quality of services
» Demonstrate care and attention to detail in undertaking work activities
» Determine appropriate attire and presentation for the role and situation
» Demonstrate recognising and taking responsibility for emotions
» Demonstrate an environment in which others can talk and act without fear of repercussion
» Demonstrate keeping commitments and not letting people down
» Demonstrate being trusting and cooperative when working alongside others
» Demonstrate taking on various responsibilities within the department as the need arises

6.4.2 Engage in continuous professional development

» Demonstrate seeking out opportunities to learn, and integrating new knowledge and skills into work
» Determine gaps in knowledge and skills for completing specific tasks, and develop a plan to acquire them

- » Demonstrate listening to and inviting feedback from others on one's own performance
- » Demonstrate using lifelong learning (continuous striving to gain knowledge and maintain competence) in the context of career development and the professional's role in delivering healthcare services
- » Analyse organisational systems for capturing learning and ensuring lessons learned
- » Employ reflective learning within the team, in which lessons are captured and integrated into future projects
- » Promote continuous learning as an integral part of organisational performance
- » Demonstrate being open to new ideas and different perspectives
- » Demonstrate keeping up to date in the place of work with input from supervisors
- » Demonstrate documenting continual professional development activities
- » Demonstrate the use of multiple techniques to acquire new knowledge and skills; process and retain information; and identify when it is necessary to acquire new knowledge and skills.
- » Convene professional organisations and/or committees
- » Determine and/or demonstrate quality improvement and/or quality assurance activities participated in
- » Demonstrate reflecting on performance and seeking what needs improvement
- » Demonstrate the process of engaging in personal career development, identifying occupational interests, strengths, options and opportunities
- » Demonstrate contributing to others' professional development
- » Demonstrate sharing experiences and learning internally and externally
- » Demonstrate seeking out challenging projects outside of core experience and achieve solid results

Basal Technology Competency(ies):	7.1 Data Science<br>7.5 Planning systems<br>7.6 ERP includes function of LMIS<br>7.12 Basic Office Skills

7.1 Data Science

- » Analyse end to end MIS technology components that are inherent in advanced MIS systems and explore how these may be relevant in different supply chain contexts
- » Determine emerging technological approaches to data visualization and analysis
- » Analyse how data science can be applied within supply chain
- » Demonstrate the ability to interact, provide guidance for data (dashboard) consolidation in various levels and use the tech tools
- » Support movement of data into electronic form at lowest/remote SC level
- » Demonstrate the ability to work with relational database

7.5 Planning systems

- » Analyse blockchain and understand the building blocks for planning systems

	» Evaluate planning systems as a value-add capability for relevant global health use cases
7.6 ERP includes function of LMIS	» Evaluate a Master Data Management System<br>» Demonstrate an understanding of the critical components of an ERP system<br>» Demonstrate an understanding of the meaning of common document types found within an ERP/LMIS<br>» Demonstrate an understanding of the logic of data visibility within an ERP<br>» Demonstrate the importance of data integrity, record management and information security
7.12 Basic Office Skills	» Demonstrate a good understanding of common presentation authoring packages<br>» Demonstrate a good understanding of common spreadsheet authoring packages<br>» Demonstrate a good understanding of common document authoring packages<br>» Demonstrate a good understanding of common email authoring packages<br>» Demonstrate a good understanding of common instant messaging packages<br>» Demonstrate a good understanding of common video conferencing authoring packages
Advanced Technology Competency(ies):	7.2 Blockchain<br>7.7 Automation<br>7.11 Cloud Computing
7.2 Blockchain	» Evaluate blockchain as a value-add capability for relevant global health use cases
7.7 Automation	» Evaluate automation as a value-add capability for relevant global health use cases
7.11 Cloud Computing	» Evaluate cloud computing as a value-add capability for relevant global health use cases

Key Performance Indicators:

- Assess Delivery Performance Cycle Time - Actual Time from assessed requirement to time delivered to end-user compared to committed Delivery Cycle Time as per Supply Chain strategy
- Assess Supplier Performance Cycle Time - Actual Supplier performance Cycle time versus Supplier commitment cycle time
- Inventory accuracy - Cycle count results
- Transportation lead time accuracy - Actual Transportation lead time versus Transportation promised lead time.

- Transport efficiency - Total cost of transport versus planned cost of transport by mode
- In-bound cost of acquisition - Actual total cost of acquisition versus Total Planned cost of acquisition including landed costs
- Manage performance cycle time
- Performance Report - Actual versus Plan reports for all metrics

Training:	Qualifications Available:	Certifications Available:
» Total Quality Management (TQM)<br>» Quality Management Systems<br>» Inventory Valuation/Financial Analysis<br>» Establishing Source and Evaluation Criteria	» Advanced Diploma in Quality Management<br>» Advanced Diploma: Financial Management<br>» Bachelor of Commerce in Financial Management	» SPSM3 TM (Enterprise-Wide Procurement Influence)<br>» SPSM Level 4 (External Procurement Influence)<br>» Lean Six Sigma Certification in Finance black belt

Manager – Warehouse and Distribution

Organisation: SC-focused Organisation				
Competency Level:	Serial Number:	Primary Process:	Job Role:	Supervises:
Managerial	3-31-34-513-3	Deliver	Manager – Warehouse and Distribution	Officer – Receiving, Manager – Customer and Sales, Head – Warehouse and Inventory Management, Officer – Order Processing/ Management, Head – Transport and Delivery Management, Lead – Waste and Returns Management, Head - Cold Chain Management
Contributions	Configure the warehouse to promote material flow. Use industries best practices in the warehouse to safeguard the material from damage, fire, obsolescence and shrinkage. Be accountable for the performance of all warehouse activities and performance metrics.			
Technical Competency(ies):	3.1 Undertake storage, warehousing and inventory management<br>3.1.1 Manage storage of commodities during emergency<br>3.2 Supply commodities to facilities<br>3.4 Manage transport for commodities<br>3.4.1 Manage transport for commodities during disaster			

3.1 Undertake storage, warehousing and inventory management

- Efficiently distribute products among suppliers, distribution centres, warehouses and customers through a logistics network
- Identify inputs to a total systems approach to designing and managing the entire flow of information, materials, and services
- Describe the choice of equipment or materials based on suitability for intended use, accuracy, safety of use and cost
- Maintain warehouses formal storage locations that identify the row, rack section, level and shelf location, typically with an alphanumeric location bar code or label
- Describe how medicines are appropriately stored, considering temperature, access and cleanliness
- Identify appropriate use of, and ability to maintain, the cold chain
- Describe the movement and storage of materials within a warehouse
- Describe how to secure the medical store and limit access to staff
- Describe organisation processes, instructions, rules and parameters for warehouse and inventory management and use of equipment (e.g., annual stock turns, expiry date tracking, stock procurement, rotation and retrieval, equipment maintenance)

» Describe policies and procedures for stock handling, distribution and withdrawal consistent with maintaining safety (e.g., policies for distribution of concentrated electrolyte solutions and cytotoxics, identification of cytotoxic drug products within the work environment, separation of like-named or like-packaged products)

» Identify requirements of information on the supplies being stored

» Describe the process of consolidating several items into larger units for fewer handlings

» Describe methods of stock rotation (e.g., first in first out, FIFO, or first to expire first out, FEFO)

» Describe the process of entering the quantity received, and updating the warehouse management system software with the stock on hand when each order is delivered

» Asses required levels of stock and/or equipment in specific situations

» Ensure accurate verification of rolling stocks

» Monitor the location and condition of the supplies being stored

» Collate and prepare information on the storage locations and facilities

» Identify any problems in using the storage locations and facilities

3.1.1 Manage storage of commodities during emergency

» Describe the types of warehouses available for emergency or disaster supply

» Describe the importance of the choice of storage site for emergency or disaster supply

» Describe the staff required for the storage of emergency or disaster supply

» Describe the equipment and material required for the warehousing of emergency or disaster supply

» Provide input to a rapid assessment of logistical needs in emergencies

3.2 Supply commodities to facilities

» Describe the various sources of distribution demand (customers)

» Describe the use of order policies for planned order generation

» Describe the calculation of re-supply quantities using a variety of inventory methods, including visual review, two bin, periodic review, order point and just in time

» Prepare and use order schedules

» Calculate safety stock

» Use economic order quantity (EOQ) principles in ordering

» Apply the use of pull and push inventory control systems

» Apply a regular, rather than urgent, order culture

» Apply order-filling priorities in relation to delivery opportunities and urgency

» Screen orders (modify order quantities on the basis of available stock, impact on service delivery, distance of facility from hospital)

» Describe assembling, checking, picking and packing of orders

» Describe making and using dispatch lists and invoices

	» Describe the balancing of supply with demand, considering both lead time and demand variability created by supply patterns not matching demand patterns<br>» Describe information on the storage locations and facilities<br>» Apply measures of customer satisfaction and identify loyal customers by using performance metrics taken from the customer perspective, with criteria such as on-time delivery, perception of quality, complaints and length of wait times
3.4 Manage transport for commodities	» Manage distribution activities, including a fleet of vehicles and distribution schedules, to deliver health commodities to facilities.<br>» Apply knowledge of policies and procedures related to handling and transporting special products such as vaccines, HIV/AIDS drugs, other high value products and narcotics<br>» Choose and compare shipping methods, considering trade-offs between costs and benefits<br>» Apply knowledge of all local and national laws/requirements for vehicle safety, driving regulations and licensing<br>» a<br>» Demonstrate maximizing freight loads while minimizing freight costs<br>» Describe the efficient use of transportation resources while meeting customers' needs<br>» Integrate movement demands with vehicle resources<br>» Apply quantitative techniques when solving logistics problems, such as designing routes and scheduling vehicles<br>» Describe the process of delivering products to hospitals and district health offices with the accompanying dispatch note and report and requisition forms<br>» Describe transport options for order delivery<br>» Ensure vehicle availability through developing and implementing a vehicle maintenance plan<br>» Describe information on distribution requirements<br>» Identify any problems in distribution requirements<br>» Integrate the process of equipping distribution sites<br>» Inform stakeholders for better coordination of distribution<br>» Integrate tracking devices (tools and indicators)<br>» Ensure training of drivers, van assistants, fleet administrators and expeditors<br>» Support investigations around missed OTIF and other failures
3.4.1 Manage transport for commodities during disaster	» Describe the different types of transport and their characteristics for emergency or disaster supply<br>» Develop a plan for the achievement of a rapid response logistics plan for deployment in the event of a medical emergency.
Personal/Management Competency(ies):	6.1 Demonstrate generic skills (e.g., literacy, numeracy, technology)<br>6.1.1 Exhibit high understanding of literacy and numeracy<br>6.2 Demonstrate strong communication skills

6.2.1 Practice cultural awareness

6.1 Demonstrate generic skills (e.g., literacy, numeracy, technology)	
6.1.1 Exhibit high understanding of literacy and numeracy	» Speak clearly and confidently, organize information in a logical manner, and consider voice tone and pace<br>» Respond to verbal messages and other cues (active listening)<br>» Express ideas and opinions clearly in written and verbal form<br>» Communicate information accurately, concisely and confidently in writing and verbally<br>» Practice a level of mathematics suitable to the job held<br>» Describe visual presentation techniques, including charting, histograms and flow sheets<br>» Read and comprehend at a level necessary to properly complete duties of the position<br>» Interpret written information in documents, such as reports, SOPs, LMIS forms, graphs, calendars, schedules, notices and directions<br>» Pay attention to detail and identify the main ideas, detect inconsistencies and identify missing information in documents<br>» Apply computer skills — such as using Word, Excel, PowerPoint and the internet — suitable to the job level<br>» Describe the process of analysing and validating KPIs<br>» Participate in the evaluation of activities that act as standards against which the KPIs are measured<br>» Apply the process of performing critical self-review on work before submission
6.2 Demonstrate strong communication skills	» Be truthful and trustworthy, and supply accurate information at all times<br>» Work as part of a workplace team<br>» Describe vocabulary and communication style and form for both written and verbal communication that is appropriate for the situation, audience and material being communicated (e.g., avoid unnecessary jargon, clearly explain medical and SCM terminology)<br>» Encourage open communication within constraints of confidentiality<br>» Organize communications about logistics<br>» Encourage participation in meetings, expressing one’s opinions, being aware of others’ needs, and being appropriately assertive when required<br>» Apply perspective into the point of view of others, understanding their needs and goals<br>» Encourage building trust, rapport and credibility with others<br>» Anticipate the communication needs and concerns of others and respond to them<br>» Avoid conflict between work and personal interests

	» Encourage meaningful two-way communication<br>» Influence others by persuasively presenting thoughts and ideas<br>» Apply assertiveness skills to deal with unreasonable requests and/or refusals that would compromise practice or consumer care<br>» Maintain a positive, supportive, and appreciative attitude<br>» Apply the ability to balance the organisation's needs and the customer's needs<br>» Use self-control by maintaining composure and keeping emotions in check, even in difficult situations, deal calmly and effectively with stressful situations<br>» Encourage the expressing of opinions and providing information in written and/or verbal form in a manner that does not elicit concern, anger or other adverse response<br>» Apply the use of a systematic process for following up that demonstrates written reports have been received and understood<br>» Describe the means by which responses to input to the work environment are monitored<br>» Seek practical ways to overcome barriers to communication<br>» Identify language representation (e.g., interpreters) at meetings when appropriate<br>» Tackle difficult situations and resolve disputes between staff<br>» Give both positive and negative feedback sensitively<br>» Maintain and improve communication<br>» Apply the process of giving and requesting feedback frequently<br>» Apply the ability to communicate effectively with shop stewards and union representatives
6.2.1 Practice cultural awareness	» Describe cultural awareness and sensitivity, treating all people with fairness, respect and dignity<br>» Describe anti-discriminatory practices in the organisation, including HR and disciplinary procedures<br>» Establish cultural sensitivity, equality and fairness at all levels of the organisation<br>» Challenge discriminatory behaviour directly and sensitively<br>» Act in a non-discriminatory way toward individuals and groups<br>» Integrate cultural awareness in learning and development approaches<br>» Avoid stereotypical responses by examining one's own behaviour and bias<br>» Manage cultural diversity in teams and make the most of differences<br>» Describe strategies and/or resources for communicating effectively with people from different cultural backgrounds<br>» Elicit information relating to values, beliefs and cultural backgrounds of consumers that may influence the way professional services are provided

Basal Technology Competency(ies):	7.4 Temperature and monitoring<br>7.6 ERP includes function of LMIS<br>7.12 Basic Office Skills<br>7.13 Have a command of technology
7.4 Temperature and monitoring	» Describe the current technology approaches for Temperature monitoring and sensors<br>» Describe what is required to implement this technology in a country context<br>» Describe infrastructure requirement for heat-sensitive items at peripheral ‘lower level’ of SC depending on volume and nature of items to handle;<br>» Define regular preventive maintenance and calibration of cold-chain equipment with biomedical engineers or logisticians<br>» Describe the policy for temperature monitoring tools during transportation and storage<br>» Describe the steps involved in health logistic management cycle: selection-order-delivery-reception-storage-distribution-inventory<br>» Describe hardware qualification and system validation of temperature monitoring<br>» Consider mass data management of temperature monitoring
7.6 ERP includes function of LMIS	» Support a Master Data Management System<br>» Describe the critical components of an ERP system<br>» Describe the meaning of common document types found within an ERP/LMIS<br>» Describe the logic of data visibility within an ERP<br>» Describe the importance of data integrity, record management and information security
7.12 Basic Office Skills	» Have a good understanding of common presentation authoring packages<br>» Have a good understanding of common spreadsheet authoring packages<br>» Have a good understanding of common document authoring packages<br>» Have a good understanding of common email authoring packages<br>» Have a good understanding of common instant messaging packages<br>» Have a good understanding of common video conferencing authoring packages
7.13 Have a command of technology	» Apply the use of technology suitable to the job held<br>» Provide input on monitoring new developments and technologies in the sector

	» Identify new technologies and recognising potential benefits for the sector<br>» Identify resources and support that are provided across the organisation to enable colleagues to make the best use of available technology<br>» Ensure that the organisation has a strategy for technology use<br>» Describe how to minimise environmental damage through technology use<br>» Use technology to maximise effectiveness and efficiency<br>» Use field-based technology (e.g., radio, general packet radio service (GPRS), satellite phone)
Advanced Technology Competency(ies):	7.7 Automation
7.7 Automation	» Describe automation as a value-add capability for relevant global health use cases

Key Performance Indicators:

- Safe product handling - Number of products handled during period out of conformance versus Number of products handled during period expressed as a % with prior period comparisons
- Number of order lines shipped with incorrectly captured Items - Number of order lines with incorrect items/SKU's versus total number of order lines shipped as a %
- Accuracy of Risk Register
- Cycle time of QA/QC report
- Overall value at risk
- Partner/Donor integration, policy, guidelines, and regulatory reforms
- QA/QC report
- Risk / Mitigation Costs
- Time to Recovery (TTR)
- Warehouse Efficiency - Time to Pick and Put away versus Planned Time to Pick and Put away. Presupposes the warehouse design promotes wave picking and flow
- Warehouse utilization - Volume used vs Volume Available
- Line Items SKU's handled vs Items / SKU's Planned over a planning horizon
- Number of warehouse locations used versus warehouse locations available (Pallet Positions may be used in larger warehouses)

Training:	Qualifications Available:	Certifications Available:
» Inventory Management<br>» Supply chain management<br>» Stores and warehousing	» Diploma in International Trade Management in Exports/Imports<br>» (CILT) International Diploma in Logistics & Transport<br>» Advanced Diploma in Logistics	» APICS Certified in logistics transportation and distribution certificate<br>» SCPro™ Certification<br>» Lean Six Sigma Certification in Warehousing green belt

Manager – Customer and Sales

Organisation:

SC-focused Organisation

Competency Level:	Serial Number:	Primary Process:	Job Role:	Supervises:
Managerial	3-35-351-3	Deliver	Manager – Customer and Sales	-

Contributions	Maintain a relationship with end-users and provide feedback to operations around the user experience. Formulate interventions to align the Supply Chain operations to the needs of the end-user. Feedback to end-users on queries and changes to the delivery process. Engage end-users to establish demand over the planning horizon and feedback to demand planners. Be consulted and informed about the delivery performance metrics.
Technical Competency(ies):	1.4 Forecast and quantify product needs<br>2.2 Build and maintain supplier relationships<br>3.2 Supply commodities to facilities<br>3.8 Customer relationship management

1.4 Forecast and quantify product needs

- Identify the factors that affect usage patterns of medications and equipment and how this affects ordering (e.g., disease outbreaks), using national policies as a guide and to ensure consistent application
- Apply principles and applications of demand forecasting
- Apply the critical requirements for effective forecasting: establishing time horizons, level of detail and use of data
- Describe policies and procedures for forecasting
- Quantify product requirements using a variety of methods
- Organize and summarize consumption data at the national level for various country programmes
- Describe the use different types of forecasts
- Apply VEN or ABC analysis to programme requirements for national level procurement
- Calculate average monthly dispensed-to-user quantities for all service delivery points nationally
- Calculate storage space requirements for all levels in the supply chain
- Calculate the months of supply on hand for each commodity at the national level
- Describe the maximum and minimum stock levels for each level in a programme
- Use various tools used in quantification (e.g., quantification software)
- Describe the use of various data sources for commodity forecasting (e.g., consumption data, services data, demographic data)
- Establish key performance indicators of forecast accuracy

	» Establish the degree of error when using forecasting methods
2.2 Build and maintain supplier relationships	» Describe factors in the analysis of the market<br>» Use and monitor the processes for prequalification and tender contracting<br>» Define of a good supplier<br>» Describe the process of locating and sourcing key suppliers, keeping in mind the total cost associated with procuring an item or service<br>» Describe supplier relationship management plans (e.g., sourcing, frequency of meetings, negotiate and monitor benchmarks for performance)<br>» Educate suppliers to create value for customers by streamlining processes in the value chain<br>» Explain the difference between strategic and non-strategic suppliers and the associated supplier management principles<br>» Describe commercial factors that contribute toward cost reduction, price savings and value improvement opportunities<br>» Ensure the strategic sourcing practices drive down cost across consecutive tenders<br>» Work with suppliers to agree cost saving opportunities in the whole value chain, with an appropriate cost sharing mechanism applied to ensure a “win-win” outcome<br>» Manage supplier risk<br>» Apply ethical sourcing
3.2 Supply commodities to facilities	» Describe the various sources of distribution demand (customers)<br>» Describe the use of order policies for planned order generation<br>» Describe the calculation of re-supply quantities using a variety of inventory methods, including visual review, two bin, periodic review, order point and just in time<br>» Prepare and use order schedules<br>» Calculate safety stock<br>» Use economic order quantity (EOQ) principles in ordering<br>» Apply the use of pull and push inventory control systems<br>» Apply a regular, rather than urgent, order culture<br>» Apply order-filling priorities in relation to delivery opportunities and urgency<br>» Screen orders (modify order quantities on the basis of available stock, impact on service delivery, distance of facility from hospital)<br>» Describe assembling, checking, picking and packing of orders<br>» Describe making and using dispatch lists and invoices<br>» Describe the balancing of supply with demand, considering both lead time and demand variability created by supply patterns not matching demand patterns<br>» Describe information on the storage locations and facilities

	» Apply measures of customer satisfaction and identify loyal customers by using performance metrics taken from the customer perspective, with criteria such as on-time delivery, perception of quality, complaints and length of wait times
3.8 Customer relationship management	» Identify different product strategies in line with market and demand source segmentation strategies.<br>» Establishing relationships with new demand sources and managing relationships with existing demand sources.<br>» Describe the alignment of demand sources with demand segmentation strategies
Personal/Management Competency(ies):	6.1 Demonstrate generic skills (e.g., literacy, numeracy, technology)<br>6.1.1 Exhibit high understanding of literacy and numeracy<br>6.2 Demonstrate strong communication skills<br>6.2.1 Practice cultural awareness
6.1 Demonstrate generic skills (e.g., literacy, numeracy, technology)	
6.1.1 Exhibit high understanding of literacy and numeracy	» Speak clearly and confidently, organize information in a logical manner, and consider voice tone and pace<br>» Respond to verbal messages and other cues (active listening)<br>» Express ideas and opinions clearly in written and verbal form<br>» Communicate information accurately, concisely and confidently in writing and verbally<br>» Practice a level of mathematics suitable to the job held<br>» Describe visual presentation techniques, including charting, histograms and flow sheets<br>» Read and comprehend at a level necessary to properly complete duties of the position<br>» Interpret written information in documents, such as reports, SOPs, LMIS forms, graphs, calendars, schedules, notices and directions<br>» Pay attention to detail and identify the main ideas, detect inconsistencies and identify missing information in documents<br>» Apply computer skills — such as using Word, Excel, PowerPoint and the internet — suitable to the job level<br>» Describe the process of analysing and validating KPIs<br>» Participate in the evaluation of activities that act as standards against which the KPIs are measured<br>» Apply the process of performing critical self-review on work before submission
6.2 Demonstrate strong communication skills	» Be truthful and trustworthy, and supply accurate information at all times<br>» Work as part of a workplace team<br>» Describe vocabulary and communication style and form for both written and verbal communication that is appropriate for the situation, audience

and material being communicated (e.g., avoid unnecessary jargon, clearly explain medical and SCM terminology)

» Encourage open communication within constraints of confidentiality
» Organize communications about logistics
» Encourage participation in meetings, expressing one's opinions, being aware of others' needs, and being appropriately assertive when required
» Apply perspective into the point of view of others, understanding their needs and goals
» Encourage building trust, rapport and credibility with others
» Anticipate the communication needs and concerns of others and respond to them
» Avoid conflict between work and personal interests
» Encourage meaningful two-way communication
» Influence others by persuasively presenting thoughts and ideas
» Apply assertiveness skills to deal with unreasonable requests and/or refusals that would compromise practice or consumer care
» Maintain a positive, supportive and appreciative attitude
» Apply the ability to balance the organisation's needs and the customer's needs
» Use self-control by maintaining composure and keeping emotions in check, even in difficult situations; deal calmly and effectively with stressful situations
» Encourage the expressing of opinions and providing information in written and/or verbal form in a manner that does not elicit concern, anger or other adverse response
» Apply the use of a systematic process for following up that demonstrates written reports have been received and understood
» Describe the means by which responses to input to the work environment are monitored
» Seek practical ways to overcome barriers to communication
» Identify language representation (e.g., interpreters) at meetings when appropriate
» Tackle difficult situations and resolve disputes between staff
» Give both positive and negative feedback sensitively
» Maintain and improve communication
» Apply the process of giving and requesting feedback frequently
» Apply the ability to communicate effectively with shop stewards and union representatives

6.2.1 Practice cultural awareness

» Describe cultural awareness and sensitivity, treating all people with fairness, respect and dignity
» Describe anti-discriminatory practices in the organisation, including HR and disciplinary procedures

	» Establish cultural sensitivity, equality and fairness at all levels of the organisation<br>» Challenge discriminatory behaviour directly and sensitively<br>» Act in a non-discriminatory way toward individuals and groups<br>» Integrate cultural awareness in learning and development approaches<br>» Avoid stereotypical responses by examining one's own behaviour and bias<br>» Manage cultural diversity in teams and make the most of differences<br>» Describe strategies and/or resources for communicating effectively with people from different cultural backgrounds<br>» Elicit information relating to values, beliefs and cultural backgrounds of consumers that may influence the way professional services are provided
Basal Technology Competency(ies):	7.6 ERP includes function of LMIS<br>7.12 Basic Office Skills<br>7.13 Have a command of technology
7.6 ERP includes function of LMIS	» Support a Master Data Management System<br>» Describe the critical components of an ERP system<br>» Describe the meaning of common document types found within an ERP/LMIS<br>» Describe the logic of data visibility within an ERP<br>» Describe the importance of data integrity, record management and information security
7.12 Basic Office Skills	» Have a good understanding of common presentation authoring packages<br>» Have a good understanding of common spreadsheet authoring packages<br>» Have a good understanding of common document authoring packages<br>» Have a good understanding of common email authoring packages<br>» Have a good understanding of common instant messaging packages<br>» Have a good understanding of common video conferencing authoring packages
7.13 Have a command of technology	» Apply the use of technology suitable to the job held<br>» Provide input on monitoring new developments and technologies in the sector<br>» Identify new technologies and recognising potential benefits for the sector<br>» Identify resources and support that are provided across the organisation to enable colleagues to make the best use of available technology

» Ensure that the organisation has a strategy for technology use

» Describe how to minimise environmental damage through technology use

» Use technology to maximise effectiveness and efficiency

» Use field-based technology (e.g., radio, general packet radio service (GPRS), satellite phone)

Advanced Technology Competency(ies):	7.7 Automation
7.7 Automation	» Describe automation as a value-add capability for relevant global health use cases

Key Performance Indicators:

» Availability - Stock out rate - Number of missed line item quantity deliveries due to stock out versus total line item quantity deliveries

» Availability - Stock levels - Current on hand stock versus Planned on hand stock

» Safe product handling - Number of products handled during period out of conformance versus Number of products handled during period expressed as a % with prior period comparisons

» Customer Incidents Report number of incidents by month/week/day comparison with previous periods

» No of Order Lines shipped with incorrectly captured Items - Number of order lines with incorrect items/SKU's versus total number of order lines shipped as a %

» Waste Management - Existence of supply chain management protocols for disposal of medical waste and management of unusable products

» Number of order lines delivered to incorrect end-user - Number of orders incorrectly addressed versus number of orders processed expressed as a %

» Number of order lines delivered out of Quality Specifications - Number of Item/SKU delivered out of specification e.g. Cold Chain infractions, Expired products versus total Number of order lines delivered as a %. A comparative graph would be an appropriate tool for the measurement of this metric.

» % Product Transferred On-Time to Demand Requirement. Actual Number of products transferred outside of the Time Limits vs Number of products Transferred.

» Transfer Product Cycle Time - Actual Transfer Cycle Time versus Planned Transferred Cycle Time

» Accurate pick - Picking Accuracy number of order lines picked inaccurately divided by the total order lines picked (SKU and Quantity)

» On-time delivery - Actual Delivery Date & Time Vs Promised Delivery Data and Time

» Customer order fulfilment cycle time & rate - Actual Order Fulfilment Cycle Time versus Promised Customer Order Fulfilment Cycle time

» Warehousing and Inventory Management OTIF - On time and in full

» Regulatory - % of procured products that meet Stringent Regulatory Authority (SRA) or WHO standards

Training:	Qualifications Available:	Certifications Available:

» Supply chain management<br>» Barcoding<br>» Transport Management	» Bachelor of Business Administration	» Higher Certificate in Export Management<br>» Lean Six Sigma Certification in Warehousing green belt<br>» Lean Six Sigma Certification in Warehousing yellow belt<br>» Occupational Certificate: Maintenance Planner

Head - Cold Chain Management

Organisation:

SC-focused Organisation

Competency Level:	Serial Number:	Primary Process:	Job Role:	Supervises:
Managerial	3-31-25-74-3	Enable – Performance	Head - Cold Chain Management	-

Contributions	Is responsible for making sure that storage capacity in correct temperature environment is sufficient for current and future need, that cold chain equipment is functional and providing a safe storage environment.
Technical Competency(ies):	1.3 List any special considerations for the product (e.g., temperature requirements, size, implications for infrastructure)<br>2.5 Ensure quality of products<br>3.1 Undertake storage, warehousing and inventory management<br>3.1.1 Manage storage of commodities during emergency

1.3 List any special considerations for the product (e.g., temperature requirements, size, implications for infrastructure)

- Describe the principles and processes of category management, including market segmentation principles
- List any specific considerations in the quantification of programme-specific products (e.g., ARVs, family planning commodities, vaccines)
- Describe current international trends in commodity availability
- Define regulatory requirements for the product

2.5 Ensure quality of products

- Use and monitor the processes for prequalification of suppliers
- Ensure health commodities are not counterfeit and meet quality standards through certification (ISO, WHO GMP)
- Describe the process of implementing, conducting, and maintaining a reporting system of pharmacovigilance (e.g. report adverse drug reactions, quality defects, batch recall) and equipment faults
- Describe the process of inspecting products when delivered and during storage to catch defects or problems before they are given to, or needed by, clients
- Ensure compliance with good distribution and good storage practices (temperature monitoring in the warehouse, transportation and distribution)
- Define product-specific shelf-life by which the product are acceptable or rejected (e.g. no less than 1 year for most of drugs, and less for short shelf-life like laboratory)

3.1 Undertake storage, warehousing and inventory management

- Efficiently distribute products among suppliers, distribution centres, warehouses and customers through a logistics network
- Identify inputs to a total systems approach to designing and managing the entire flow of information, materials, and services
- Describe the choice of equipment or materials based on suitability for intended use, accuracy, safety of use and cost

	» Maintain warehouses formal storage locations that identify the row, rack section, level and shelf location, typically with an alphanumeric location bar code or label<br>» Describe how medicines are appropriately stored, considering temperature, access and cleanliness<br>» Identify appropriate use of, and ability to maintain, the cold chain<br>» Describe the movement and storage of materials within a warehouse<br>» Describe how to secure the medical store and limit access to staff<br>» Describe organisation processes, instructions, rules and parameters for warehouse and inventory management and use of equipment (e.g., annual stock turns, expiry date tracking, stock procurement, rotation and retrieval, equipment maintenance)<br>» Describe policies and procedures for stock handling, distribution and withdrawal consistent with maintaining safety (e.g., policies for distribution of concentrated electrolyte solutions and cytotoxics, identification of cytotoxic drug products within the work environment, separation of like-named or like-packaged products)<br>» Identify requirements of information on the supplies being stored<br>» Describe the process of consolidating several items into larger units for fewer handlings<br>» Describe methods of stock rotation (e.g., first in first out, FIFO, or first to expire first out, FEFO)<br>» Describe the process of entering the quantity received, and updating the warehouse management system software with the stock on hand when each order is delivered<br>» Asses required levels of stock and/or equipment in specific situations<br>» Ensure accurate verification of rolling stocks<br>» Monitor the location and condition of the supplies being stored<br>» Collate and prepare information on the storage locations and facilities<br>» Identify any problems in using the storage locations and facilities
3.1.1 Manage storage of commodities during emergency	» Describe the types of warehouses available for emergency or disaster supply<br>» Describe the importance of the choice of storage site for emergency or disaster supply<br>» Describe the staff required for the storage of emergency or disaster supply<br>» Describe the equipment and material required for the warehousing of emergency or disaster supply<br>» Provide input to a rapid assessment of logistical needs in emergencies
Personal/Management Competency(ies):	5.5 Manage and plan projects<br>5.5.4 Direct/participate in teamwork<br>6.1 Demonstrate generic skills (e.g., literacy, numeracy, technology)<br>6.1.1 Exhibit high understanding of literacy and numeracy

5.5 Manage and plan projects	
5.5.4 Direct/participate in teamwork	» Describe the process of working with team members within their area of authority to establish achievable goals and strategies that are consistent with the objectives established for the organisation as a whole<br>» Describe an organisational chart that shows the lines of reporting and responsibility among staff<br>» Actively contribute a perspective and make a positive contribution to team-based problem solving and decision making<br>» Provide feedback, encouragement and support to team members for progressing strategic goals<br>» Define team performance in relation to the organisation's mission and goals<br>» Encourage teams to think for themselves and resolve problems<br>» Encourage and harness diversity within the team to boost team effectiveness<br>» Encourage and support the team to work through its stages of development and perform well<br>» Describe roles and responsibilities in relation to employees' expertise and the expectations of collaborating team members<br>» Describe where their position fits in the structure (organisation, SC, healthcare sector) , the responsibilities their position entails and the accountabilities linked to the position
6.1 Demonstrate generic skills (e.g., literacy, numeracy, technology)	
6.1.1 Exhibit high understanding of literacy and numeracy	» Speak clearly and confidently, organize information in a logical manner, and consider voice tone and pace<br>» Respond to verbal messages and other cues (active listening)<br>» Express ideas and opinions clearly in written and verbal form<br>» Communicate information accurately, concisely and confidently in writing and verbally<br>» Practice a level of mathematics suitable to the job held<br>» Describe visual presentation techniques, including charting, histograms and flow sheets<br>» Read and comprehend at a level necessary to properly complete duties of the position<br>» Interpret written information in documents, such as reports, SOPs, LMIS forms, graphs, calendars, schedules, notices and directions<br>» Pay attention to detail and identify the main ideas, detect inconsistencies and identify missing information in documents<br>» Apply computer skills — such as using Word, Excel, PowerPoint and the internet — suitable to the job level<br>» Describe the process of analysing and validating KPIs

	» Participate in the evaluation of activities that act as standards against which the KPIs are measured<br>» Apply the process of performing critical self-review on work before submission
Basal Technology Competency(ies):	7.4 Temperature and monitoring<br>7.6 ERP includes function of LMIS<br>7.12 Basic Office Skills
7.4 Temperature and monitoring	» Describe the current technology approaches for temperature monitoring and sensors<br>» Describe what is required to implement this technology in a country context<br>» Describe infrastructure requirement for heat-sensitive items at peripheral 'lower level' of SC depending on volume and nature of items to handle;<br>» Define regular preventive maintenance and calibration of cold-chain equipment with biomedical engineers or logisticians<br>» Describe the policy for temperature monitoring tools during transportation and storage<br>» Describe the steps involved in health logistic management cycle: selection-order-delivery-reception-storage-distribution-inventory<br>» Describe hardware qualification and system validation of temperature monitoring<br>» Consider mass data management of temperature monitoring
7.6 ERP includes function of LMIS	» Support a Master Data Management System<br>» Describe the critical components of an ERP system<br>» Describe the meaning of common document types found within an ERP/LMIS<br>» Describe the logic of data visibility within an ERP<br>» Describe the importance of data integrity, record management and information security
7.12 Basic Office Skills	» Have a good understanding of common presentation authoring packages<br>» Have a good understanding of common spreadsheet authoring packages<br>» Have a good understanding of common document authoring packages<br>» Have a good understanding of common email authoring packages<br>» Have a good understanding of common instant messaging packages<br>» Have a good understanding of common video conferencing authoring packages

Key Performance Indicators:
» % Number of items failing Cold Chain Parameters. Total number of items deviated from temperature within recommended versus total items stored under temperature-controlled environment<br>» Adherence to Cold Chain infrastructure inspection maintenance requirements - Report on infrastructure % inspection and or maintenance compliance versus total inspections.<br>» % Infrastructure available. Infrastructure in useable state versus total infrastructure by type<br>» Utilisation of temperature-controlled infrastructure capacity vs actual used over a specified period<br>» Efficiency of temperature-controlled infrastructure - Total running costs of temperature-controlled infrastructure versus planned running cost per infrastructure component<br>» Temperature controlled infrastructure asset register accuracy. Asset Count accuracy report<br>» % of active cold chain having passive cold chain (contingency plan) versus total infrastructure

Training:	Qualifications Available:	Certifications Available:
» Warehouse design<br>» Cold chain management	» Diploma in International Trade Management in Exports/Imports<br>» (CILT) International Diploma in Logistics & Transport	» APICS Certified in logistics transportation and distribution certificate<br>» CILT/LLA-Medical logistics<br>» International Certificate in Logistics & Transport

Manager - Contract and 3PLs

Organisation:

SC-focused Organisation

Competency Level:	Serial Number:	Primary Process:	Job Role:	Supervises:
**Managerial**	**5-54-22-23-3**	**Enable – Performance**	**Manager - Contract and 3PLs**	**-**

Contributions	Responsible for creation and management of contracts between the supply chain organisation and third-party logistics to provide transportation services across the healthcare supply chain.
Technical Competency(ies):	2.2 Build and maintain supplier relationships<br>2.3 Manage tendering processes and supplier agreements

2.2 Build and maintain supplier relationships

- Describe factors in the analysis of the market
- Use and monitor the processes for prequalification and tender contracting
- Define of a good supplier
- Describe the process of locating and sourcing key suppliers, keeping in mind the total cost associated with procuring an item or service
- Describe supplier relationship management plans (e.g., sourcing, frequency of meetings, negotiate and monitor benchmarks for performance)
- Educate suppliers to create value for customers by streamlining processes in the value chain
- Explain the difference between strategic and non-strategic suppliers and the associated supplier management principles
- Describe commercial factors that contribute toward cost reduction, price savings and value improvement opportunities
- Ensure the strategic sourcing practices drive down cost across consecutive tenders
- Work with suppliers to agree cost saving opportunities in the whole value chain, with an appropriate cost sharing mechanism applied to ensure a “win-win” outcome
- Manage supplier risk
- Apply ethical sourcing

2.3 Manage tendering processes and supplier agreements

- Demonstrate the correct use of request for qualifications (RFQ), invitations to bid (ITB) and request for proposals (RFP) methods
- Demonstrate knowledge of local and international tendering procedures (e.g., open competitive bidding, restricted tender, competitive negotiation, direct procurement)
- Write detailed specifications for tenders

	» Describe the high-level guidance required for high-value and politically sensitive procurements<br>» Describe the process of developing and managing contracts<br>» Describe the procurement, return and exchange policies<br>» List appropriate legislation and policy in regard to procurement processes, and what is required to comply<br>» Ensure ethical behaviour, abiding by conflict of interest policies<br>» Describe the process of evaluating tender bids based on the specification and evaluation criteria including capability of the supplier, problems relating to outcomes of the contract.<br>» Follow contract approval process, including contract negotiations<br>» Discuss some of the key issues relevant to negotiating supply contracts (e.g., volume usage over time, price volume agreements, alternate supplier clauses, duration of contract, period of review, terms for contract termination and renewal)<br>» Describe the process of awarding and managing contracts, resolving any issues as they arise<br>» Describe the process of debriefing unsuccessful suppliers<br>» Describe the ways in which compliance with purchasing policies and procedures is monitored<br>» Participate in procurement planning including a yearly activities schedule and developing a wide range of briefs, specifications and commercial documentation<br>» Identify strategic sourcing projects, capturing benefits, work requirements, impacts and cost savings<br>» Participate in cross-functional teams handling strategic procurements<br>» Describe effective internal and external procurement networks<br>» List local and international health commodity prices and understand the factors affecting them<br>» Describe the process of engaging clients across the public sector in presentations demonstrating the benefits of a range of strategic projects designed to ensure cost savings and value for money for government procurement
Personal/Management Competency(ies):	5.4 Manage outsourcing of SCM functions<br>6.1 Demonstrate generic skills (e.g., literacy, numeracy, technology)<br>6.1.1 Exhibit high understanding of literacy and numeracy
5.4 Manage outsourcing of SCM functions	» Provide input on negotiating and compiling contracts with outside parties for delivery of logistics services
6.1 Demonstrate generic skills (e.g., literacy, numeracy, technology)	
6.1.1 Exhibit high understanding of literacy and numeracy	» Speak clearly and confidently, organize information in a logical manner, and consider voice tone and pace<br>» Respond to verbal messages and other cues (active listening)

- Express ideas and opinions clearly in written and verbal form
- Communicate information accurately, concisely and confidently in writing and verbally
- Practice a level of mathematics suitable to the job held
- Describe visual presentation techniques, including charting, histograms and flow sheets
- Read and comprehend at a level necessary to properly complete duties of the position
- Interpret written information in documents, such as reports, SOPs, LMIS forms, graphs, calendars, schedules, notices and directions
- Pay attention to detail and identify the main ideas, detect inconsistencies and identify missing information in documents
- Apply computer skills — such as using Word, Excel, PowerPoint and the internet — suitable to the job level
- Describe the process of analysing and validating KPIs
- Participate in the evaluation of activities that act as standards against which the KPIs are measured
- Apply the process of performing critical self-review on work before submission

Basal Technology Competency(ies):	7.4 Temperature and monitoring<br>7.6 ERP includes function of LMIS<br>7.12 Basic Office Skills

7.4 Temperature and monitoring

- Describe the current technology approaches for Temperature monitoring and sensors
- Describe what is required to implement this technology in a country context
- Describe infrastructure requirement for heat-sensitive items at peripheral 'lower level' of SC depending on volume and nature of items to handle;
- Define regular preventive maintenance and calibration of cold-chain equipment with biomedical engineers or logisticians
- Describe the policy for temperature monitoring tools during transportation and storage
- Describe the steps involved in health logistic management cycle: selection-order-delivery-reception-storage-distribution-inventory
- Describe hardware qualification and system validation of temperature monitoring
- Consider mass data management of temperature monitoring

7.6 ERP includes function of LMIS

- Support a Master Data Management System
- Describe the critical components of an ERP system
- Describe the meaning of common document types found within an ERP/LMIS

	» Describe the logic of data visibility within an ERP<br>» Describe the importance of data integrity, record management and information security
7.12 Basic Office Skills	» Have a good understanding of common presentation authoring packages<br>» Have a good understanding of common spreadsheet authoring packages<br>» Have a good understanding of common document authoring packages<br>» Have a good understanding of common email authoring packages<br>» Have a good understanding of common instant messaging packages<br>» Have a good understanding of common video conferencing authoring packages

Key Performance Indicators:

- Item Master Data Accuracy
- Inbound Transportation Master Data Accuracy by Item SKU
- Item SKU Lead Time Accuracy
- Risk avoidance, elimination and or Mitigation Costs
- % Adherence to Service Level Agreement (SLA) - Number of 3PLS's utilised versus 3PLS's with SLA's formalised
- SLA Performance by agreement by suppliers' report
- 3PL actual cost versus Planned cost

Training:	Qualifications Available:	Certifications Available:
» Negotiation<br>» Transport Management<br>» Third party logistics dynamics	» (CILT) International Diploma in Logistics & Transport<br>» Bachelor of Commerce in Logistics Management	

Lead - Partners Integration

Organisation:

SC-focused Organisation

Competency Level:	Serial Number:	Primary Process:	Job Role:	Supervises:
Managerial	5-55-66-4	Enable – Regulatory (Individual) Compliance	Lead - Partners Integration	-

Contributions	Responsible for liaising and coordinating with partner organisations, understanding partner organisation drivers and finding win-win situations between them.
Personal/Management Competency(ies):	5.5 Manage and plan projects<br>5.5.1 Develop and direct project plans<br>5.5.2 Execute strategic decision making<br>5.5.3 Manage partnerships<br>5.5.4 Direct/participate in teamwork<br>6.1 Demonstrate generic skills (e.g., literacy, numeracy, technology)<br>6.1.1 Exhibit high understanding of literacy and numeracy<br>6.2 Demonstrate strong communication skills<br>6.2.1 Practice cultural awareness<br>6.6 Abide by rules / laws / legislation

5.5 Manage and plan projects

5.5.1 Develop and direct project plans

- » Evaluate obstacles and critical events for ensuring the six rights of logistics management (right quantity, product, condition, place, time and cost) and develop contingency plans to address them; monitor progress and take necessary corrective action when needed
- » Determine an operational plan that is consistent with the strategic plan, links specific goals and strategies, and identifies the performance indicators to be used to measure achievements
- » Demonstrate the use of information gathering techniques from stakeholders, analysing situations and identifying implications to make correct decisions
- » Review the questions, “Where are we going?” and “How are we going to get there?” and create a specific and purposeful path to achieve this.
- » Demonstrate the process reference model to managing supply chain processes (i.e., integrating business process re-engineering, benchmarking and process measurement)
- » Analyse the current state of a process and derive the desired future state

	» Demonstrate the process of selecting and applying tools or technological solutions to frequently encountered problems<br>» Analyse the process of providing staff with the necessary tools to carry out plans<br>» Demonstrate the ability to monitor progress and make changes as required<br>» Convene meetings for planning, organisation and monitoring logistics activities<br>» Ensure staff are aware that they are accountable for achieving the desired results<br>» Determine how planning, organising and managing resources will bring about the successful completion of projects<br>» Determine the key features of a successful change management strategy
5.5.2 Execute strategic decision making	» Determine how one can make decisions regarding one's own workload and area of responsibility<br>» Determine the process of prioritizing competing tasks and perform them quickly and efficiently according to their importance; finding new ways of organizing or planning work to accomplish it more efficiently<br>» Evaluate the proper time and space for consultation around decisions being made.<br>» Demonstrate the process of considering the input of resources including staff needed<br>» Analyse assumptions against facts<br>» Determine the key issues in a complex situation and come to the heart of the problem quickly<br>» Evaluate relevant information before making decisions<br>» Provide input to strategic decisions in the interest of the organisation's goals<br>» Evaluate when to analyse, evaluate and execute tough decisions when necessary<br>» Analyse the impact of decisions on others' work or team goals<br>» Communicate decisions and ensure they are incorporated into policies and processes<br>» Demonstrate inclusive and consultative strategic planning to establish strategic objectives<br>» Evaluate emerging trends and practices<br>» Analyse the macro and long-term consequences of decisions
5.5.3 Manage partnerships	» Demonstrate the concepts of partnership working<br>» Determine the process of supporting implementation of partnership programmes<br>» Analyse the process of experimenting with and trialling new ideas with partners<br>» Analyse partnerships to deliver programme and increase impact

- Analyse innovation and creativity in partnership work
- Communicate key information with partner members
- Involve and value partners in all aspects of programming
- Analyse problems with supply chain relationships
- Demonstrate the process of communicating effectively with nurses, doctors and other members of the healthcare team
- Analyse the process of responding to consumer complaints or comments about services and/or advice received
- Analyse collaboration and consultation across the sector and encourage the team to work with others to gain market insight
- Encourage input from other key actors in the humanitarian sector and develop relationships with a cross-section of actors
- Analyse the process of meeting the reporting requirements of vertical programmes
- Analyse the structure of the health system at a national level and explain this to others
- Analyse the roles of other members of the healthcare team (including with consumers) in a way that engenders understanding and confidence in the team and its members
- Analyse the structure of the organisation, environment and/or service in which they work
- Demonstrate how vertical programmes work within the health system
- Evaluate the communication network established to achieve work outcomes
- Demonstrate knowledge of the structure of the health system at the provincial/regional level and explain this to others
- Analyse the roles and functions of government agencies in regulating and supporting supply chain organisations
- Demonstrate knowledge of change management (e.g. PtD Theory of Change)

5.5.4 Direct/participate in teamwork

- Work with team members within their area of authority to establish achievable goals and strategies that are consistent with the objectives established for the organisation as a whole
- Evaluate an organisational chart that shows the lines of reporting and responsibility among staff
- Determine a culture of actively contributing a perspective and making a positive contribution to team-based problem solving and decision making
- Determine a culture of providing feedback, encouragement and support to team members for progressing strategic goals
- Monitor team performance in relation to the organisation’s mission and goals
- Enable the creation of a culture of teams thinking for themselves and resolving problems
- Demonstrate the process of encouraging and harnessing diversity within the team to boost team effectiveness

- Demonstrate the process of encouraging and supporting the team to work through its stages of development and perform well
- Determine roles and responsibilities in relation to employees' expertise and the expectations of collaborating team members
- Demonstrate where their position fits in the structure (organisation, SC, healthcare sector) , the responsibilities their position entails and the accountabilities linked to the position

6.1 Demonstrate generic skills (e.g., literacy, numeracy, technology)

6.1.1 Exhibit high understanding of literacy and numeracy

- Encourage speaking clearly and confidently, organizing information in a logical manner, and considering voice tone and pace
- Demonstrate responding to verbal messages and other cues (active listening)
- Demonstrate expressing ideas and opinions clearly in written and verbal form
- Demonstrate communicating information accurately, concisely and confidently in writing and verbally
- Demonstrate a level of mathematics suitable to the job held
- Demonstrate knowledge of visual presentation techniques, including charting, histograms and flow sheets
- Demonstrate reading and comprehending at a level necessary to properly complete duties of the position
- Demonstrate the process of interpreting written information in documents, such as reports, SOPs, LMIS forms, graphs, calendars, schedules, notices and directions
- Demonstrate paying attention to detail and identifying the main ideas, detecting inconsistencies and identifying missing information in documents
- Demonstrate computer skills — such as using Word, Excel, PowerPoint and the internet — suitable to the job level
- Demonstrate the process of analysing and validating KPIs
- Participate in the evaluation of activities that act as standards against which the KPIs are measured
- Demonstrate the process of performing critical self-review on work before submission

6.2 Demonstrate strong communication skills

- Demonstrate being truthful and trustworthy, and supplying accurate information at all times
- Demonstrate working as part of a workplace team
- Determine vocabulary and communication style and form for both written and verbal communication that is appropriate for the situation, audience and material being communicated (e.g., avoid unnecessary jargon, clearly explain medical and SCM terminology)
- Demonstrate open communication within constraints of confidentiality
- Analyse communications about logistics

	» Demonstrate participation in meetings, expressing one's opinions, being aware of others' needs, and being appropriately assertive when required<br>» Demonstrate perspective into the point of view of others, understanding their needs and goals<br>» Demonstrate the practise of building trust, rapport and credibility with others<br>» Determine the communication needs and concerns of others and respond to them<br>» Demonstrate avoiding conflict between work and personal interests<br>» Demonstrate the practice of meaningful two-way communication<br>» Demonstrate influence on others by persuasively presenting thoughts and ideas<br>» Demonstrate assertiveness skills to deal with unreasonable requests and/or refusals that would compromise practice or consumer care<br>» Demonstrate a positive, supportive and appreciative attitude<br>» Demonstrate the ability to balance the organisation's needs and the customer's needs<br>» Demonstrate self-control by maintaining composure and keeping emotions in check, even in difficult situations; deal calmly and effectively with stressful situations<br>» Demonstrate the expressing of opinions and providing information in written and/or verbal form in a manner that does not elicit concern, anger or other adverse response<br>» Demonstrate the use of a systematic process for following up that demonstrates written reports have been received and understood<br>» Evaluate the means by which responses to input to the work environment are monitored<br>» Seek practical ways to overcome barriers to communication<br>» Ensure language representation (e.g., interpreters) at meetings when appropriate<br>» Demonstrate tackling difficult situations and resolving disputes between staff<br>» Demonstrate giving both positive and negative feedback sensitively<br>» Demonstrate maintaining and improving communication<br>» Demonstrate the process of giving and requesting feedback frequently<br>» Demonstrate the ability to communicate effectively with shop stewards and union representatives
6.2.1 Practice cultural awareness	» Demonstrate cultural awareness and sensitivity, treating all people with fairness, respect and dignity<br>» Demonstrate anti-discriminatory practices in the organisation, including HR and disciplinary procedures<br>» Demonstrate cultural sensitivity, equality and fairness at all levels of the organisation<br>» Demonstrate challenging discriminatory behaviour directly and sensitively

- Demonstrate acting in a non-discriminatory way toward individuals and groups
- Demonstrate the process of integrating cultural awareness in learning and development approaches
- Demonstrate avoiding stereotypical responses by examining one's own behaviour and bias
- Demonstrate managing cultural diversity in teams and make the most of differences
- Demonstrate the use of strategies and/or resources for communicating effectively with people from different cultural backgrounds
- Supply information relating to values, beliefs and cultural backgrounds of consumers that may influence the way professional services are provided

6.6 Abide by rules / laws / legislation

- Demonstrate compliance with the legislation that covers the practice of public health supply chains and healthcare, and describe its purpose
- Analyse requirements of professional codes, guidelines and standards adopted as part of the legislative framework
- Demonstrate keeping up to date with changes in legal instruments, as informed by national-level managers
- Demonstrate compliance with international regulations in decision making for the distribution system, including customs regulations on import and export requirements (e.g., trade tariffs and duties on imported goods) and security regulations (e.g., 2007 SAFE Ports Act, NAFTA or European Union trade agreements)
- Demonstrate applying and understanding regulatory affairs and the key aspects of pharmaceutical registration and legislation
- Demonstrate knowledge of the principles of business economics and intellectual property rights, including the basics of patent interpretation
- Demonstrate knowledge of applicable procurement law and the practical effects of a contract's terms and conditions
- Evaluate current industry and government regulations governing sustainability
- Demonstrate a working knowledge of key hazardous materials handling laws
- Determine policies and procedures relating to workplace safety that are consistent with agreed or recognised standards
- Analyse professional standards and conventions, as well as workplace policies and procedures, for preparing pharmaceutical products
- Demonstrate all standard operating procedures
- Evaluate the process of complying with data privacy and data protection laws

Basal Technology Competency(ies):	7.12 Basic Office Skills<br>7.13 Have a command of technology

7.12 Basic Office Skills	» Demonstrate a good understanding of common presentation authoring packages<br>» Demonstrate a good understanding of common spreadsheet authoring packages<br>» Demonstrate a good understanding of common document authoring packages<br>» Demonstrate a good understanding of common email authoring packages<br>» Demonstrate a good understanding of common instant messaging packages<br>» Demonstrate a good understanding of common video conferencing authoring packages
7.13 Have a command of technology	» Demonstrate the use of technology suitable to the job held<br>» Monitor new developments and technologies in the sector<br>» Experiment with new technologies and recognising potential benefits for the sector<br>» Ensure that resources and support are provided across the organisation to enable colleagues to make the best use of available technology<br>» Determine what the organisations' strategy is for technology use<br>» Determine how to minimise environmental damage through technology use<br>» Analyse the use of technology to maximise effectiveness and efficiency<br>» Analyse the use of field-based technology (e.g., radio, general packet radio service (GPRS), satellite phone)

Key Performance Indicators:

- % of formalized agreements between partners and donors. Actual number of partner and donor agreements versus formalized agreements
- Regular Feedback to Donors and Partners on performance of programmes and or donations

Training:	Qualifications Available:	Certifications Available:
» Legislation and Standards<br>» International trade regulation<br>» Partnership Management	» Bachelor of Commerce in Philosophy, Politics and Economics<br>» Bachelor of Science Honours: Pharmacology<br>» Bachelor of Accounting Honours	» APICS Certified in production and inventory management certificate<br>» Lean Six Sigma Certification in Healthcare black belt

Manager - Logistics Planning

Organisation: SC-focused Organisation				
Competency Level:	Serial Number:	Primary Process:	Job Role:	Supervises:
Managerial	3-34-15-14-3	Plan – Deliver	Manager - Logistics Planning	Officer - Logistics Planning

Contributions	Responsible for identifying the required levels of customer service and subsequently developing cost effective methods of delivering those required service levels. Provides a consolidating role in the development of an end-to-end Supply Chain Plan/Strategy.
Technical Competency(ies):	1.4 Forecast and quantify product needs<br>1.5 Supply planning<br>3.4 Manage transport for commodities<br>3.4.1 Manage transport for commodities during disaster

1.4 Forecast and quantify product needs

- Identify the factors that affect usage patterns of medications and equipment and how this affects ordering (e.g., disease outbreaks), using national policies as a guide and to ensure consistent application
- Apply principles and applications of demand forecasting
- Apply the critical requirements for effective forecasting: establishing time horizons, level of detail and use of data
- Describe policies and procedures for forecasting
- Quantify product requirements using a variety of methods
- Organize and summarize consumption data at the national level for various country programmes
- Describe the use different types of forecasts
- Apply VEN or ABC analysis to programme requirements for national level procurement
- Calculate average monthly dispensed-to-user quantities for all service delivery points nationally
- Calculate storage space requirements for all levels in the supply chain
- Calculate the months of supply on hand for each commodity at the national level
- Describe the maximum and minimum stock levels for each level in a programme
- Use various tools used in quantification (e.g., quantification software)
- Describe the use of various data sources for commodity forecasting (e.g., consumption data, services data, demographic data)
- Establish key performance indicators of forecast accuracy
- Establish the degree of error when using forecasting methods

1.5 Supply planning

- Describe a purchasing and supply plan in line with national priorities
- Balance supply chain resources and trigger activities to correct any imbalances

	» Explain the formalisation of sales and operations plans through sales and operations meeting.<br>» Translate sales and operations plan into executable plans to ensure dispatch of goods in line with overall priorities.<br>» Describe the national commodity pipeline
3.4 Manage transport for commodities	» Manage distribution activities, including a fleet of vehicles and distribution schedules, to deliver health commodities to facilities.<br>» Apply knowledge of policies and procedures related to handling and transporting special products such as vaccines, HIV/AIDS drugs, other high value products and narcotics<br>» Choose and compare shipping methods, considering trade-offs between costs and benefits<br>» Apply knowledge of all local and national laws/requirements for vehicle safety, driving regulations and licensing<br>» a<br>» Demonstrate maximizing freight loads while minimizing freight costs<br>» Describe the efficient use of transportation resources while meeting customers' needs<br>» Integrate movement demands with vehicle resources<br>» Apply quantitative techniques when solving logistics problems, such as designing routes and scheduling vehicles<br>» Describe the process of delivering products to hospitals and district health offices with the accompanying dispatch note and report and requisition forms<br>» Describe transport options for order delivery<br>» Ensure vehicle availability through developing and implementing a vehicle maintenance plan<br>» Describe information on distribution requirements<br>» Identify any problems in distribution requirements<br>» Integrate the process of equipping distribution sites<br>» Inform stakeholders for better coordination of distribution<br>» Integrate tracking devices (tools and indicators)<br>» Ensure training of drivers, van assistants, fleet administrators and expeditors<br>» Support investigations around missed OTIF and other failures
3.4.1 Manage transport for commodities during disaster	Describe the different types of transport and their characteristics for emergency or disaster supply<br>Develop a plan for the achievement of a rapid response logistics plan for deployment in the event of a medical emergency.
Personal/Management Competency(ies):	6.1 Demonstrate generic skills (e.g., literacy, numeracy, technology)<br>6.1.1 Exhibit high understanding of literacy and numeracy<br>6.2 Demonstrate strong communication skills<br>6.2.1 Practice cultural awareness

6.1 Demonstrate generic skills (e.g., literacy, numeracy, technology)	
6.1.1 Exhibit high understanding of literacy and numeracy	» Speak clearly and confidently, organize information in a logical manner, and consider voice tone and pace<br>» Respond to verbal messages and other cues (active listening)<br>» Express ideas and opinions clearly in written and verbal form<br>» Communicate information accurately, concisely and confidently in writing and verbally<br>» Practice a level of mathematics suitable to the job held<br>» Describe visual presentation techniques, including charting, histograms and flow sheets<br>» Read and comprehend at a level necessary to properly complete duties of the position<br>» Interpret written information in documents, such as reports, SOPs, LMIS forms, graphs, calendars, schedules, notices and directions<br>» Pay attention to detail and identify the main ideas, detect inconsistencies and identify missing information in documents<br>» Apply computer skills — such as using Word, Excel, PowerPoint and the internet — suitable to the job level<br>» Describe the process of analysing and validating KPIs<br>» Participate in the evaluation of activities that act as standards against which the KPIs are measured<br>» Apply the process of performing critical self-review on work before submission
6.2 Demonstrate strong communication skills	» Be truthful and trustworthy, and supply accurate information at all times<br>» Work as part of a workplace team<br>» Describe vocabulary and communication style and form for both written and verbal communication that is appropriate for the situation, audience and material being communicated (e.g., avoid unnecessary jargon, clearly explain medical and SCM terminology)<br>» Encourage open communication within constraints of confidentiality<br>» Organize communications about logistics<br>» Encourage participation in meetings, expressing one's opinions, being aware of others' needs, and being appropriately assertive when required<br>» Apply perspective into the point of view of others, understanding their needs and goals<br>» Encourage building trust, rapport and credibility with others<br>» Anticipate the communication needs and concerns of others and respond to them<br>» Avoid conflict between work and personal interests<br>» Encourage meaningful two-way communication<br>» Influence others by persuasively presenting thoughts and ideas

» Apply assertiveness skills to deal with unreasonable requests and/or refusals that would compromise practice or consumer care

» Maintain a positive, supportive, and appreciative attitude

» Apply the ability to balance the organisation's needs and the customer's needs

» Use self-control by maintaining composure and keeping emotions in check, even in difficult situations, deal calmly and effectively with stressful situations

» Encourage the expressing of opinions and providing information in written and/or verbal form in a manner that does not elicit concern, anger or other adverse response

» Apply the use of a systematic process for following up that demonstrates written reports have been received and understood

» Describe the means by which responses to input to the work environment are monitored

» Seek practical ways to overcome barriers to communication

» Identify language representation (e.g., interpreters) at meetings when appropriate

» Tackle difficult situations and resolve disputes between staff

» Give both positive and negative feedback sensitively

» Maintain and improve communication

» Apply the process of giving and requesting feedback frequently

» Apply the ability to communicate effectively with shop stewards and union representatives

6.2.1 Practice cultural awareness

» Describe cultural awareness and sensitivity, treating all people with fairness, respect and dignity

» Describe anti-discriminatory practices in the organisation, including HR and disciplinary procedures

» Establish cultural sensitivity, equality and fairness at all levels of the organisation

» Challenge discriminatory behaviour directly and sensitively

» Act in a non-discriminatory way toward individuals and groups

» Integrate cultural awareness in learning and development approaches

» Avoid stereotypical responses by examining one's own behaviour and bias

» Manage cultural diversity in teams and make the most of differences

» Describe strategies and/or resources for communicating effectively with people from different cultural backgrounds

» Elicit information relating to values, beliefs and cultural backgrounds of consumers that may influence the way professional services are provided

Basal Technology Competency(ies):	7.4 Temperature and monitoring<br>7.6 ERP includes function of LMIS<br>7.12 Basic Office Skills<br>7.13 Have a command of technology

7.4 Temperature and monitoring	» Describe the current technology approaches for Temperature monitoring and sensors<br>» Describe what is required to implement this technology in a country context<br>» Describe infrastructure requirement for heat-sensitive items at peripheral ‘lower level’ of SC depending on volume and nature of items to handle;<br>» Define regular preventive maintenance and calibration of cold-chain equipment with biomedical engineers or logisticians<br>» Describe the policy for temperature monitoring tools during transportation and storage<br>» Describe the steps involved in health logistic management cycle: selection-order-delivery-reception-storage-distribution-inventory<br>» Describe hardware qualification and system validation of temperature monitoring<br>» Consider mass data management of temperature monitoring
7.6 ERP includes function of LMIS	» Support a Master Data Management System<br>» Describe the critical components of an ERP system<br>» Describe the meaning of common document types found within an ERP/LMIS<br>» Describe the logic of data visibility within an ERP<br>» Describe the importance of data integrity, record management and information security
7.12 Basic Office Skills	» Have a good understanding of common presentation authoring packages<br>» Have a good understanding of common spreadsheet authoring packages<br>» Have a good understanding of common document authoring packages<br>» Have a good understanding of common email authoring packages<br>» Have a good understanding of common instant messaging packages<br>» Have a good understanding of common video conferencing authoring packages
7.13 Have a command of technology	» Apply the use of technology suitable to the job held<br>» Provide input on monitoring new developments and technologies in the sector<br>» Identify new technologies and recognising potential benefits for the sector<br>» Identify resources and support that are provided across the organisation to enable colleagues to make the best use of available technology<br>» Ensure that the organisation has a strategy for technology use<br>» Describe how to minimise environmental damage through technology use<br>» Use technology to maximise effectiveness and efficiency<br>» Use field-based technology (e.g., radio, general packet radio service (GPRS), satellite phone)
Advanced Technology Competency(ies):	7.2 Blockchain<br>7.3 Unmanned Aerial Vehicles (UAVs)

	7.7 Automation<br>7.9 Additive Manufacturing<br>7.11 Cloud Computing<br>7.14 eProcurement
7.2 Blockchain	» Describe blockchain as a value-add capability for relevant global health use cases
7.3 Unmanned Aerial Vehicles (UAVs)	» Describe the current UAV landscape, their application and the enabling environment required to support country implementation<br>» Describe UAVs as a value-add capability for relevant global health use cases<br>» Describe the laws/ regulations around the use of UAV's
7.7 Automation	» Describe automation as a value-add capability for relevant global health use cases
7.9 Additive Manufacturing	» Describe additive manufacturing as a value-add capability for relevant global health use cases
7.11 Cloud Computing	» Describe cloud computing as a value-add capability for relevant global health use cases
7.14 eProcurement	Describe how to transact with an eProcurement system where necessary<br>Describe eProcurement as a value-add capability for relevant global health use cases<br>Apply the benefits and limitations of eProcurement to best utilise the system

Key Performance Indicators:

- Customer order fulfilment cycle time & rate - Actual Order Fulfilment Cycle Time versus Promised Customer Order Fulfilment Cycle time
- %Cubic Metres delivered vs Cubic Metres planned by Location delivered
- Cost per Cubic Metres delivered vs Cost per Cubic Metres planned by Location delivered
- Transportation Lead time planned versus Actual Transportation Lead Time
- Route efficiency - Actual Routing costs versus Standard / Planned Routing costs
- On-time and In-Full (OTIF)
- 0 reroutes
- Routing costs incurred and the service level achieved

Training:	Qualifications Available:	Certifications Available:
» Forecasting software training<br>» Systems training<br>» Risk and exception management<br>» Inventory management, Min-Max Replenishment<br>» ABC/VEN Inventory Classification<br>» Transport Management<br>» Cold chain management<br>» Inventory planning certification	» Bachelor of Business Administration in Logistics and Supply Chain Management<br>» National Diploma: Freight Handling Logistics	» International Certificate in Logistics & Transport

Manager – Demand and Supply Planning

Organisation:

SC-focused Organisation

Competency Level:	Serial Number:	Primary Process:	Job Role:	Supervises:
Managerial	1-14-15-3	Plan – Procurement	Manager – Demand and Supply Planning	Head – Demand and Supply Planning, Officer – Inventory and Demand Planning, Lead - Demand and Supply Planning, Manager - Logistics Planning

Contributions	Articulates the Supply Chain Strategy to the Procurement functionaries. Ensures that the Procurement department is enabled to supply the Supply Chain in line with the Supply Chain Materials and Resource Plan., Facilitates the execution of the Supply Strategy, Implements best practices to extract the most efficiency from each process and guarantee supply accuracy, lead time and cost diminution as well as data accuracy.
Technical Competency(ies):	1.3 List any special considerations for the product (e.g., temperature requirements, size, implications for infrastructure)<br>1.4 Forecast and quantify product needs<br>1.5 Supply planning
1.3 List any special considerations for the product (e.g., temperature requirements, size, implications for infrastructure)	» Describe the principles and processes of category management, including market segmentation principles<br>» List any specific considerations in the quantification of programme-specific products (e.g., ARVs, family planning commodities, vaccines)<br>» Describe current international trends in commodity availability<br>» Define regulatory requirements for the product
1.4 Forecast and quantify product needs	» Identify the factors that affect usage patterns of medications and equipment and how this affects ordering (e.g., disease outbreaks), using national policies as a guide and to ensure consistent application<br>» Apply principles and applications of demand forecasting<br>» Apply the critical requirements for effective forecasting: establishing time horizons, level of detail and use of data<br>» Describe policies and procedures for forecasting<br>» Quantify product requirements using a variety of methods<br>» Organize and summarize consumption data at the national level for various country programmes<br>» Describe the use different types of forecasts<br>» Apply VEN or ABC analysis to programme requirements for national level procurement

	» Calculate average monthly dispensed-to-user quantities for all service delivery points nationally<br>» Calculate storage space requirements for all levels in the supply chain<br>» Calculate the months of supply on hand for each commodity at the national level<br>» Describe the maximum and minimum stock levels for each level in a programme<br>» Use various tools used in quantification (e.g., quantification software)<br>» Describe the use of various data sources for commodity forecasting (e.g., consumption data, services data, demographic data)<br>» Establish key performance indicators of forecast accuracy<br>» Establish the degree of error when using forecasting methods
1.5 Supply planning	» Describe a purchasing and supply plan in line with national priorities<br>» Balance supply chain resources and trigger activities to correct any imbalances<br>» Explain the formalisation of sales and operations plans through sales and operations meeting.<br>» Translate sales and operations plan into executable plans to ensure dispatch of goods in line with overall priorities.<br>» Describe the national commodity pipeline
Personal/Management Competency(ies):	5.3 Implement risk management and monitoring and evaluation activities for the supply chain<br>5.3.1 Ensure monitoring and evaluation activities are completed<br>5.6 Manage financial activities<br>6.1 Demonstrate generic skills (e.g., literacy, numeracy, technology)<br>6.1.1 Exhibit high understanding of literacy and numeracy<br>6.2 Demonstrate strong communication skills<br>6.2.1 Practice cultural awareness<br>6.3 Utilize problem-solving skills<br>6.3.3 Take risk into account and implement security measures<br>6.4 Exhibit professional and ethical values<br>6.4.1 Demonstrate integrity
5.3 Implement risk management and monitoring and evaluation activities for the supply chain	
5.3.1 Ensure monitoring and evaluation activities are completed	» Describe the periodic evaluations to maintain processes by gathering pertinent information — such as problem symptoms from knowledgeable sources, carrying these through to the problems, potential causes and root causes of the problem<br>» Quantify the operational performance of similar companies and establish internal targets based on best-in-class results

» Apply key performance measurements and continuous process improvement initiatives to improve process quality on a continual basis

» Provide results of the analyses to colleagues

» Control and check errors, taking corrective action so deviation from standards are minimised and the organisation's goals are achieved

» Identify the use of appropriate technological developments to improve the system

» Describe that continuous process improvement is an accepted way of organisational life

» Execute ways of eliminating unnecessary steps in system design

» Apply processes that strive to eliminate waste

» Apply processes to encourage sustainability (e.g., reducing carbon footprint, establishing a paperless office, using renewable energy)

» Describe the systematic approach used to close process or system performance gaps through streamlining and cycle time reduction, and identify and eliminate causes of quality below specifications, process variation and non-value-adding activities

» Outline the relationship between technology and process functionality

» Describe how policy, guidelines and regulatory reforms impact SC performance

5.6 Manage financial activities

» Manage all resources with care, applying budgetary principles

» Participate in meeting budgets and donor requirements

» Recognise budgets (national, regional, etc.) as necessary for work (e.g., wages budget, touring budget, stationery budget, project budgets for using NGO funds)

» Describe the process of producing timely and clear financial reports for funders and donors

» Describe the process of keeping records following government budgeting, accounting and financial practices

» Describe the process of maintaining an overview of multiple budgets from multiple sources

» Providing input during seeking and using information on financial funding requirements

» Ensure timely decision making with regard to financial shortfalls

» Provide budget tracking for logistics activities according to established procedures

» Provide input during the analysis of financial statements and explain components of a balance sheet and income statement

» Provide input on creating interactive decision support models that allow the development of multiple scenarios and demonstrating the sensitivity of multiple independent variables

» Describe the success or failure of a business using financial accounting

» Calculate the total system cost of delivering a product or service to the customer

» Describe the key considerations for developing a business plan

» Describe the structure against which business performance will be monitored (e.g., cost centres, chart of accounts)
» List key performance indicators used to monitor business performance (e.g., turnover, profitability)
» List strategies for minimising the risk of fraudulent activity (e.g., reconciliation of purchase orders, receipts and payment approvals; dual signatory arrangements for funds transfers)
» Describe investigative processes and options for confirming the existence of fraudulent activity (e.g., sample audits, forensic accounting services)
» Provide input on preparing funding applications for trusts and grants
» Apply the process of ensuring financial transparency
» Describe the general monetary value of medicines and equipment
» Provide input to securing financing and managing budgets to support distribution operations
» Apply the ability to translate financial information into SC language
» Apply SC finance knowledge

6.1 Demonstrate generic skills (e.g., literacy, numeracy, technology)

6.1.1 Exhibit high understanding of literacy and numeracy

» Speak clearly and confidently, organize information in a logical manner, and consider voice tone and pace
» Respond to verbal messages and other cues (active listening)
» Express ideas and opinions clearly in written and verbal form
» Communicate information accurately, concisely and confidently in writing and verbally
» Practice a level of mathematics suitable to the job held
» Describe visual presentation techniques, including charting, histograms and flow sheets
» Read and comprehend at a level necessary to properly complete duties of the position
» Interpret written information in documents, such as reports, SOPs, LMIS forms, graphs, calendars, schedules, notices and directions
» Pay attention to detail and identify the main ideas, detect inconsistencies and identify missing information in documents
» Apply computer skills — such as using Word, Excel, PowerPoint and the internet — suitable to the job level
» Describe the process of analysing and validating KPIs
» Participate in the evaluation of activities that act as standards against which the KPIs are measured
» Apply the process of performing critical self-review on work before submission

6.2 Demonstrate strong communication skills	» Be truthful and trustworthy, and supply accurate information at all times<br>» Work as part of a workplace team<br>» Describe vocabulary and communication style and form for both written and verbal communication that is appropriate for the situation, audience and material being communicated (e.g., avoid unnecessary jargon, clearly explain medical and SCM terminology)<br>» Encourage open communication within constraints of confidentiality<br>» Organize communications about logistics<br>» Encourage participation in meetings, expressing one's opinions, being aware of others' needs, and being appropriately assertive when required<br>» Apply perspective into the point of view of others, understanding their needs and goals<br>» Encourage building trust, rapport and credibility with others<br>» Anticipate the communication needs and concerns of others and respond to them<br>» Avoid conflict between work and personal interests<br>» Encourage meaningful two-way communication<br>» Influence others by persuasively presenting thoughts and ideas<br>» Apply assertiveness skills to deal with unreasonable requests and/or refusals that would compromise practice or consumer care<br>» Maintain a positive, supportive and appreciative attitude<br>» Apply the ability to balance the organisation's needs and the customer's needs<br>» Use self-control by maintaining composure and keeping emotions in check, even in difficult situations; deal calmly and effectively with stressful situations<br>» Encourage the expressing of opinions and providing information in written and/or verbal form in a manner that does not elicit concern, anger or other adverse response<br>» Apply the use of a systematic process for following up that demonstrates written reports have been received and understood<br>» Describe the means by which responses to input to the work environment are monitored<br>» Seek practical ways to overcome barriers to communication<br>» Identify language representation (e.g., interpreters) at meetings when appropriate<br>» Tackle difficult situations and resolve disputes between staff<br>» Give both positive and negative feedback sensitively<br>» Maintain and improve communication<br>» Apply the process of giving and requesting feedback frequently<br>» Apply the ability to communicate effectively with shop stewards and union representatives
6.2.1 Practice cultural awareness	» Describe cultural awareness and sensitivity, treating all people with fairness, respect and dignity

» Describe anti-discriminatory practices in the organisation, including HR and disciplinary procedures
» Establish cultural sensitivity, equality and fairness at all levels of the organisation
» Challenge discriminatory behaviour directly and sensitively
» Act in a non-discriminatory way toward individuals and groups
» Integrate cultural awareness in learning and development approaches
» Avoid stereotypical responses by examining one's own behaviour and bias
» Manage cultural diversity in teams and make the most of differences
» Describe strategies and/or resources for communicating effectively with people from different cultural backgrounds
» Elicit information relating to values, beliefs and cultural backgrounds of consumers that may influence the way professional services are provided

6.3 Utilize problem-solving skills

» Identify information to solve problems in a sensitive and ethical manner
» Manage day to day and complex problems in a timely manner
» Explain problem situations and their step-by-step transformation based on planning and reasoning, without apportioning blame
» Critically review, analyse, synthesize, compare and interpret information; draw conclusions from relevant and/or missing information; and understand the relationship among facts and apply this understanding when solving problems
» Describe a range of possible approaches/strategies that are effective for resolving conflict in the workplace (e.g., negotiation, collaborative problem-solving, mediation, arbitration)
» Use an open environment that encourages people to work together
» Resolve conflicts as they arise
» Use creativity through questioning, attempting to improve on ideas, applying other experiences and working toward action in problem solving
» Break down facts and thoughts into strengths and weaknesses
» Think in a careful way to solve problems, analyse data, and recall and apply information, involving others
» Practice goal-directed thinking and action in situations in which no routine solutions exist
» Maintain appropriate ethical and moral standards in resolving problems
» Choose between alternative courses of action, using cognitive processes such as memory, thinking and evaluation
» Map likely consequences of decisions to choose the best course of action
» Ask other people to help with solving problems
» Use difficult or unusual situations to develop unique approaches and useful solutions

» Commit to a solution in a timely manner, and develop a realistic approach for applying the chosen solution; evaluate the outcome of the solution to see if further action is needed, and identify lessons learned
» Describe the impact of conflict in the workplace (e.g., tension, low morale, absenteeism, system or service failure, aggressive or uncooperative behaviours)
» Follow up on problems to ensure they are fixed
» Describe situations where referral is warranted (e.g., severe emotional distress, intractable dispute)

6.3.3 Take risk into account and implement security measures

» Follow security guidelines, plans and standard operating procedures
» Provide input into security planning and reviews
» Ensure compliance with legal, regulatory, ethical and social requirements in humanitarian settings
» Carry out responsibilities and follow instructions (e.g., completing a personal risk assessment and filling in travel plans)
» Ensure personal behaviour does not impact personal or organisational security

6.4 Exhibit professional and ethical values

6.4.1 Demonstrate integrity

» Accept responsibility for one's own work tasks and performance
» Work within a framework of clearly understood humanitarian values and ethics
» Stand by decisions and hold others accountable when necessary
» Does not abuse one's own power or position
» Manage team members to ensure they do not abuse their power or position
» Identify when individuals or the organisation is straying from organisation goals, and challenge them to uphold ethics
» Resist undue political pressure in decision making
» Support staff in maintaining ethical stances
» Make time in team for ethical inquiry and reflection
» Show consistency between expressed principles and behaviour
» Provide input to ensure that principles, values and ethics are embedded in policy
» Act without consideration of personal gain
» Promote transparency in decision making structures and processes
» Provide input on ensuring programmes are acting with integrity, and recognising the impact of not doing so
» Recognise one's own limitations and act upon them
» Apply all standard operating procedures
» Work in a safe and legal way
» Demonstrate respect, dignity and consideration for consumers

	» Discuss the impact of a no-blame culture on reporting and preventing recurrence of incidents<br>» Describe requests of colleagues that might be regarded as unreasonable<br>» Communicate (verbally and by example) expectations of the desired standards and approaches to be adopted<br>» Describe roles and responsibilities in terms of the position statement/duty statement of the position held<br>» Demonstrate punctuality<br>» Explain the obligation to apply professional care and expertise to deliver high quality of services<br>» Demonstrate care and attention to detail in undertaking work activities<br>» Describe appropriate attire and presentation for the role and situation<br>» Recognise and take responsibility for emotions<br>» Describe an environment in which others can talk and act without fear of repercussion<br>» Seek to keep commitments and not let people down<br>» Be trusting and cooperative when working alongside others<br>» Take on various responsibilities within the department as the need arises
Basal Technology Competency(ies):	7.6 ERP includes function of LMIS<br>7.12 Basic Office Skills<br>7.13 Have a command of technology
7.6 ERP includes function of LMIS	» Support a Master Data Management System<br>» Describe the critical components of an ERP system<br>» Describe the meaning of common document types found within an ERP/LMIS<br>» Describe the logic of data visibility within an ERP<br>» Describe the importance of data integrity, record management and information security
7.12 Basic Office Skills	» Have a good understanding of common presentation authoring packages<br>» Have a good understanding of common spreadsheet authoring packages<br>» Have a good understanding of common document authoring packages<br>» Have a good understanding of common email authoring packages<br>» Have a good understanding of common instant messaging packages<br>» Have a good understanding of common video conferencing authoring packages
7.13 Have a command of technology	» Apply the use of technology suitable to the job held<br>» Provide input on monitoring new developments and technologies in the sector

» Identify new technologies and recognising potential benefits for the sector

» Identify resources and support that are provided across the organisation to enable colleagues to make the best use of available technology

» Ensure that the organisation has a strategy for technology use

» Describe how to minimise environmental damage through technology use

» Use technology to maximise effectiveness and efficiency

» Use field-based technology (e.g., radio, general packet radio service (GPRS), satellite phone)

Advanced Technology Competency(ies):	7.14 eProcurement
7.14 eProcurement	Describe how to transact with an eProcurement system where necessary<br>Describe eProcurement as a value-add capability for relevant global health use cases<br>Apply the benefits and limitations of eProcurement to best utilise the system

Key Performance Indicators:

» % of emergency orders issued in the last 12 months

» Availability - Stock out rate - Number of missed line item quantity deliveries due to stock out versus total line item quantity deliveries

» Availability - Stock levels - Current on hand stock versus Planned on hand stock

» Data accuracy - Vendor Master data accuracy, item / SKU Master Data accuracy

» Forecast accuracy, measured and reported on.

» Inventory Days of Supply - Actual inventory - Planned Demand over the planning horizon divided by the average daily usage

» Order accuracy - Net requirement versus order quantities in the planning horizon

» Supply adequacy - Net requirements are reflected in the Procurement pipeline to secure sufficient future supply.

» Supply Chain Planning Cost recorded and measured against required Supply China Planning Cycle times to accomplish Supply Chain Strategic objectives.

» Supply Plan report; supplier performance report

» Vendor on time delivery (VOTD)

» Transport efficiency - Total cost of transport versus planned cost of transport by mode

» Forecast bias.

» Forecast accuracy measured based on absolute percentage error (MAPE)

» Transportation lead time accuracy - Actual Transportation lead time versus Transportation promised lead time

Training:	Qualifications Available:	Certifications Available:

» Risk and exception management<br>» Import & export regulations for products<br>» Inventory management, Min-Max Replenishment<br>» ABC/VEN Inventory Classification	» Diploma in International Trade Management in Exports/Imports<br>» Bachelor of Business Science	» Occupational Certificate: Procurement Officer<br>» CIPS Level 4 Diploma in Procurement and Supply<br>» SPSM2® (Global Procurement Management)

Manager – Performance Management

Organisation:<br>**SC-focused Organisation**				
Competency Level:	Serial Number:	Primary Process:	Job Role:	Supervises:
Managerial	5-531-532-3	Enable – SC Performance	Manager – Performance Management	-
Contributions	Creates and Maintains the data collection framework to enable the measurement of the metrics prescribed in the Public Health System Organisation, Operating Model and Supply Chain Strategy			
Personal/Management Competency(ies):	5.5 Manage and plan projects<br>5.5.1 Develop and direct project plans<br>5.5.2 Execute strategic decision making<br>5.5.3 Manage partnerships<br>5.5.4 Direct/participate in teamwork<br>5.6 Manage financial activities<br>6.1 Demonstrate generic skills (e.g., literacy, numeracy, technology)<br>6.1.1 Exhibit high understanding of literacy and numeracy<br>6.2 Demonstrate strong communication skills<br>6.2.1 Practice cultural awareness			

5.5 Manage and plan projects

5.5.1 Develop and direct project plans	» Anticipate obstacles and critical events for ensuring the six rights of logistics management (right quantity, product, condition, place, time and cost) and develop contingency plans to address them; monitor progress and take necessary corrective action when needed<br>» Describe an operational plan that is consistent with the strategic plan, links specific goals and strategies, and identifies the performance indicators to be used to measure achievements<br>» Apply the use of information gathering techniques from stakeholders, analysing situations and identifying implications to make correct decisions<br>» Answer the questions, “Where are we going?” and “How are we going to get there?” and create a specific and purposeful path to achieve this.<br>» Apply the process reference model to managing supply chain processes (i.e., integrating business process re-engineering, benchmarking and process measurement)<br>» Capture the current state of a process and derive the desired future state<br>» Select and apply tools or technological solutions to frequently encountered problems<br>» Provide staff with the necessary tools to carry out plans

	» Describe the process of monitoring progress and making changes as required<br>» Facilitate meetings for planning, organisation and monitoring logistics activities<br>» Describe the process of ensuring staff are aware that they are accountable for achieving the desired results<br>» Describe how planning, organising and managing resources will bring about the successful completion of projects<br>» Describe the key features of a successful change management strategy
5.5.2 Execute strategic decision making	» Make decisions regarding one's own workload and area of responsibility<br>» Prioritize competing tasks and perform them quickly and efficiently according to their importance; find new ways of organizing or planning work to accomplish it more efficiently<br>» Ensure proper time and space for consultation around decisions being made.<br>» Consider the input of resources including staff needed<br>» Check assumptions against facts<br>» Identify the key issues in a complex situation and come to the heart of the problem quickly<br>» Gather relevant information before making decisions<br>» Describe the process of providing input for making strategic decisions in the interest of the organisation's goals<br>» Describe when to analyse, evaluate and execute tough decisions when necessary<br>» Consider the impact of decisions on others' work or team goals<br>» Describe the process to communicate decisions and ensure they are incorporated into policies and processes<br>» Describe inclusive and consultative strategic planning to establish strategic objectives<br>» Identify and support emerging trends and practices<br>» Describe the macro and long-term consequences of decisions
5.5.3 Manage partnerships	» Apply the concepts of partnership working<br>» Support implementation of partnership programmes<br>» Describe the process of experimenting with and trialling new ideas with partners<br>» Identify partnerships to deliver programme and increase impact<br>» Use innovation and creativity in partnership work<br>» Describe the process of communicating key information with partner members<br>» Describe the process of involving and valuing partners in all aspects of programming<br>» Identify problems with supply chain relationships

	» Communicate effectively with nurses, doctors and other members of the healthcare team<br>» Respond to consumer complaints or comments about services and/or advice received<br>» Foster collaboration and consultation across the sector and encourage the team to work with others to gain market insight<br>» Assist in sharing input from other key actors in the humanitarian sector and develop relationships with a cross-section of actors<br>» Meet the reporting requirements of vertical programmes<br>» Outline the structure of the health system at a national level<br>» Discuss the role of other members of the healthcare team (including with consumers) in a way that engenders understanding and confidence in the team and its members<br>» Describe the structure of the organisation, environment and/or service in which they work<br>» Describe how vertical programmes work within the health system<br>» Describe the communication network established to achieve work outcomes<br>» Describe the structure of the health system at the provincial/regional level and explain this to others<br>» List the roles and functions of government agencies in regulating and supporting supply chain organisations<br>» Describe knowledge of change management (e.g. PtD Theory of Change)
5.5.4 Direct/participate in teamwork	» Describe the process of working with team members within their area of authority to establish achievable goals and strategies that are consistent with the objectives established for the organisation as a whole<br>» Describe an organisational chart that shows the lines of reporting and responsibility among staff<br>» Actively contribute a perspective and make a positive contribution to team-based problem solving and decision making<br>» Provide feedback, encouragement and support to team members for progressing strategic goals<br>» Define team performance in relation to the organisation's mission and goals<br>» Encourage teams to think for themselves and resolve problems<br>» Encourage and harness diversity within the team to boost team effectiveness<br>» Encourage and support the team to work through its stages of development and perform well<br>» Describe roles and responsibilities in relation to employees' expertise and the expectations of collaborating team members<br>» Describe where their position fits in the structure (organisation, SC, healthcare sector) , the responsibilities their position entails and the accountabilities linked to the position

5.6 Manage financial activities	» Manage all resources with care, applying budgetary principles<br>» Participate in meeting budgets and donor requirements<br>» Recognise budgets (national, regional, etc.) as necessary for work (e.g., wages budget, touring budget, stationery budget, project budgets for using NGO funds)<br>» Describe the process of producing timely and clear financial reports for funders and donors<br>» Describe the process of keeping records following government budgeting, accounting and financial practices<br>» Describe the process of maintaining an overview of multiple budgets from multiple sources<br>» Providing input during seeking and using information on financial funding requirements<br>» Ensure timely decision making with regard to financial shortfalls<br>» Provide budget tracking for logistics activities according to established procedures<br>» Provide input during the analysis of financial statements and explain components of a balance sheet and income statement<br>» Provide input on creating interactive decision support models that allow the development of multiple scenarios and demonstrating the sensitivity of multiple independent variables<br>» Describe the success or failure of a business using financial accounting<br>» Calculate the total system cost of delivering a product or service to the customer<br>» Describe the key considerations for developing a business plan<br>» Describe the structure against which business performance will be monitored (e.g., cost centres, chart of accounts)<br>» List key performance indicators used to monitor business performance (e.g., turnover, profitability)<br>» List strategies for minimising the risk of fraudulent activity (e.g., reconciliation of purchase orders, receipts and payment approvals; dual signatory arrangements for funds transfers)<br>» Describe investigative processes and options for confirming the existence of fraudulent activity (e.g., sample audits, forensic accounting services)<br>» Provide input on preparing funding applications for trusts and grants<br>» Apply the process of ensuring financial transparency<br>» Describe the general monetary value of medicines and equipment<br>» Provide input to securing financing and managing budgets to support distribution operations<br>» Apply the ability to translate financial information into SC language<br>» Apply SC finance knowledge
6.1 Demonstrate generic skills (e.g., literacy, numeracy, technology)	

6.1.1 Exhibit high understanding of literacy and numeracy	» Speak clearly and confidently, organize information in a logical manner, and consider voice tone and pace<br>» Respond to verbal messages and other cues (active listening)<br>» Express ideas and opinions clearly in written and verbal form<br>» Communicate information accurately, concisely and confidently in writing and verbally<br>» Practice a level of mathematics suitable to the job held<br>» Describe visual presentation techniques, including charting, histograms and flow sheets<br>» Read and comprehend at a level necessary to properly complete duties of the position<br>» Interpret written information in documents, such as reports, SOPs, LMIS forms, graphs, calendars, schedules, notices and directions<br>» Pay attention to detail and identify the main ideas, detect inconsistencies and identify missing information in documents<br>» Apply computer skills — such as using Word, Excel, PowerPoint and the internet — suitable to the job level<br>» Describe the process of analysing and validating KPIs<br>» Participate in the evaluation of activities that act as standards against which the KPIs are measured<br>» Apply the process of performing critical self-review on work before submission
6.2 Demonstrate strong communication skills	» Be truthful and trustworthy, and supply accurate information at all times<br>» Work as part of a workplace team<br>» Describe vocabulary and communication style and form for both written and verbal communication that is appropriate for the situation, audience and material being communicated (e.g., avoid unnecessary jargon, clearly explain medical and SCM terminology)<br>» Encourage open communication within constraints of confidentiality<br>» Organize communications about logistics<br>» Encourage participation in meetings, expressing one's opinions, being aware of others' needs, and being appropriately assertive when required<br>» Apply perspective into the point of view of others, understanding their needs and goals<br>» Encourage building trust, rapport and credibility with others<br>» Anticipate the communication needs and concerns of others and respond to them<br>» Avoid conflict between work and personal interests<br>» Encourage meaningful two-way communication<br>» Influence others by persuasively presenting thoughts and ideas<br>» Apply assertiveness skills to deal with unreasonable requests and/or refusals that would compromise practice or consumer care<br>» Maintain a positive, supportive and appreciative attitude

» Apply the ability to balance the organisation’s needs and the customer’s needs
» Use self-control by maintaining composure and keeping emotions in check, even in difficult situations; deal calmly and effectively with stressful situations
» Encourage the expressing of opinions and providing information in written and/or verbal form in a manner that does not elicit concern, anger or other adverse response
» Apply the use of a systematic process for following up that demonstrates written reports have been received and understood
» Describe the means by which responses to input to the work environment are monitored
» Seek practical ways to overcome barriers to communication
» Identify language representation (e.g., interpreters) at meetings when appropriate
» Tackle difficult situations and resolve disputes between staff
» Give both positive and negative feedback sensitively
» Maintain and improve communication
» Apply the process of giving and requesting feedback frequently
» Apply the ability to communicate effectively with shop stewards and union representatives

6.2.1 Practice cultural awareness

» Describe cultural awareness and sensitivity, treating all people with fairness, respect and dignity
» Describe anti-discriminatory practices in the organisation, including HR and disciplinary procedures
» Establish cultural sensitivity, equality and fairness at all levels of the organisation
» Challenge discriminatory behaviour directly and sensitively
» Act in a non-discriminatory way toward individuals and groups
» Integrate cultural awareness in learning and development approaches
» Avoid stereotypical responses by examining one’s own behaviour and bias
» Manage cultural diversity in teams and make the most of differences
» Describe strategies and/or resources for communicating effectively with people from different cultural backgrounds
» Elicit information relating to values, beliefs and cultural backgrounds of consumers that may influence the way professional services are provided

Key Performance Indicators:

» Assess Delivery Performance Cycle Time - Actual Time from assessed requirement to time delivered to end-user compared to committed Delivery Cycle Time as per Supply Chain strategy
» Assess Supplier Performance Cycle Time - Actual Supplier performance Cycle time versus Supplier commitment cycle time
» Inventory accuracy - Cycle count results

- Transportation lead time accuracy - Actual Transportation lead time versus Transportation promised lead time.
- Transport efficiency - Total cost of transport versus planned cost of transport by mode
- In-bound cost of acquisition - Actual total cost of acquisition versus Total Planned cost of acquisition including landed costs
- Manage performance cycle time
- Performance Report - Actual versus Plan reports for all metrics

Training:	Qualifications Available:	Certifications Available:
» Basic Finance<br>» Benchmarking<br>» Lean Manufacturing<br>» Performance Management<br>» Optimization<br>» Quality Management<br>» Six Sigma<br>» Supply Chain Performance Measurements<br>» Total Quality Management (TQM)<br>» Data management<br>» ERP Systems<br>» Installed base management<br>» Interpreting Specifications<br>» Office automation tools<br>» Product Information Management (Product Data Management)	» National Diploma: Financial Markets<br>» National Diploma: Transport Economics	

Manager – SC Data and Analytics

Organisation:

SC-focused Organisation

Competency Level:	Serial Number:	Primary Process:	Job Role:	Supervises:
Managerial	5-56-551-3	Enable – Data Information	Manager – SC Data and Analytics	Officer – SC Data Quality and Use

Contributions	Building of the data gathering infrastructure to support the Supply Chain organisation by rapidly generating relevant and vital metrics as well as their associated KPIs. The ability to build data structures attached to the transaction systems capable of providing data from source transactions. The ability to mine this data, use it to build analytic engines as well as leverage the data to build predictability models for risk mitigation and improved planning.
Personal/Management Competency(ies):	5.5 Manage and plan projects<br>5.5.1 Develop and direct project plans<br>5.5.2 Execute strategic decision making<br>5.5.4 Direct/participate in teamwork<br>5.6 Manage financial activities<br>6.1 Demonstrate generic skills (e.g., literacy, numeracy, technology)<br>6.1.1 Exhibit high understanding of literacy and numeracy<br>6.2 Demonstrate strong communication skills<br>6.2.1 Practice cultural awareness

5.5 Manage and plan projects

5.5.1 Develop and direct project plans

- » Anticipate obstacles and critical events for ensuring the six rights of logistics management (right quantity, product, condition, place, time and cost) and develop contingency plans to address them; monitor progress and take necessary corrective action when needed
- » Describe an operational plan that is consistent with the strategic plan, links specific goals and strategies, and identifies the performance indicators to be used to measure achievements
- » Apply the use of information gathering techniques from stakeholders, analysing situations and identifying implications to make correct decisions
- » Answer the questions, “Where are we going?” and “How are we going to get there?” and create a specific and purposeful path to achieve this.
- » Apply the process reference model to managing supply chain processes (i.e., integrating business process re-engineering, benchmarking and process measurement)
- » Capture the current state of a process and derive the desired future state
- » Select and apply tools or technological solutions to frequently encountered problems
- » Provide staff with the necessary tools to carry out plans

	» Describe the process of monitoring progress and making changes as required<br>» Facilitate meetings for planning, organisation and monitoring logistics activities<br>» Describe the process of ensuring staff are aware that they are accountable for achieving the desired results<br>» Describe how planning, organising and managing resources will bring about the successful completion of projects<br>» Describe the key features of a successful change management strategy
5.5.2 Execute strategic decision making	» Make decisions regarding one's own workload and area of responsibility<br>» Prioritize competing tasks and perform them quickly and efficiently according to their importance; find new ways of organizing or planning work to accomplish it more efficiently<br>» Ensure proper time and space for consultation around decisions being made.<br>» Consider the input of resources including staff needed<br>» Check assumptions against facts<br>» Identify the key issues in a complex situation and come to the heart of the problem quickly<br>» Gather relevant information before making decisions<br>» Describe the process of providing input for making strategic decisions in the interest of the organisation's goals<br>» Describe when to analyse, evaluate and execute tough decisions when necessary<br>» Consider the impact of decisions on others' work or team goals<br>» Describe the process to communicate decisions and ensure they are incorporated into policies and processes<br>» Describe inclusive and consultative strategic planning to establish strategic objectives<br>» Identify and support emerging trends and practices<br>» Describe the macro and long-term consequences of decisions
5.5.4 Direct/participate in teamwork	» Describe the process of working with team members within their area of authority to establish achievable goals and strategies that are consistent with the objectives established for the organisation as a whole<br>» Describe an organisational chart that shows the lines of reporting and responsibility among staff<br>» Actively contribute a perspective and make a positive contribution to team-based problem solving and decision making<br>» Provide feedback, encouragement and support to team members for progressing strategic goals<br>» Define team performance in relation to the organisation's mission and goals<br>» Encourage teams to think for themselves and resolve problems

	» Encourage and harness diversity within the team to boost team effectiveness<br>» Encourage and support the team to work through its stages of development and perform well<br>» Describe roles and responsibilities in relation to employees' expertise and the expectations of collaborating team members<br>» Describe where their position fits in the structure (organisation, SC, healthcare sector) , the responsibilities their position entails and the accountabilities linked to the position
5.6 Manage financial activities	» Manage all resources with care, applying budgetary principles<br>» Participate in meeting budgets and donor requirements<br>» Recognise budgets (national, regional, etc.) as necessary for work (e.g., wages budget, touring budget, stationery budget, project budgets for using NGO funds)<br>» Describe the process of producing timely and clear financial reports for funders and donors<br>» Describe the process of keeping records following government budgeting, accounting and financial practices<br>» Describe the process of maintaining an overview of multiple budgets from multiple sources<br>» Providing input during seeking and using information on financial funding requirements<br>» Ensure timely decision making with regard to financial shortfalls<br>» Provide budget tracking for logistics activities according to established procedures<br>» Provide input during the analysis of financial statements and explain components of a balance sheet and income statement<br>» Provide input on creating interactive decision support models that allow the development of multiple scenarios and demonstrating the sensitivity of multiple independent variables<br>» Describe the success or failure of a business using financial accounting<br>» Calculate the total system cost of delivering a product or service to the customer<br>» Describe the key considerations for developing a business plan<br>» Describe the structure against which business performance will be monitored (e.g., cost centres, chart of accounts)<br>» List key performance indicators used to monitor business performance (e.g., turnover, profitability)<br>» List strategies for minimising the risk of fraudulent activity (e.g., reconciliation of purchase orders, receipts and payment approvals; dual signatory arrangements for funds transfers)<br>» Describe investigative processes and options for confirming the existence of fraudulent activity (e.g., sample audits, forensic accounting services)<br>» Provide input on preparing funding applications for trusts and grants<br>» Apply the process of ensuring financial transparency

	» Describe the general monetary value of medicines and equipment<br>» Provide input to securing financing and managing budgets to support distribution operations<br>» Apply the ability to translate financial information into SC language<br>» Apply SC finance knowledge
6.1 Demonstrate generic skills (e.g., literacy, numeracy, technology)	
6.1.1 Exhibit high understanding of literacy and numeracy	» Speak clearly and confidently, organize information in a logical manner, and consider voice tone and pace<br>» Respond to verbal messages and other cues (active listening)<br>» Express ideas and opinions clearly in written and verbal form<br>» Communicate information accurately, concisely and confidently in writing and verbally<br>» Practice a level of mathematics suitable to the job held<br>» Describe visual presentation techniques, including charting, histograms and flow sheets<br>» Read and comprehend at a level necessary to properly complete duties of the position<br>» Interpret written information in documents, such as reports, SOPs, LMIS forms, graphs, calendars, schedules, notices and directions<br>» Pay attention to detail and identify the main ideas, detect inconsistencies and identify missing information in documents<br>» Apply computer skills — such as using Word, Excel, PowerPoint and the internet — suitable to the job level<br>» Describe the process of analysing and validating KPIs<br>» Participate in the evaluation of activities that act as standards against which the KPIs are measured<br>» Apply the process of performing critical self-review on work before submission
6.2 Demonstrate strong communication skills	» Be truthful and trustworthy, and supply accurate information at all times<br>» Work as part of a workplace team<br>» Describe vocabulary and communication style and form for both written and verbal communication that is appropriate for the situation, audience and material being communicated (e.g., avoid unnecessary jargon, clearly explain medical and SCM terminology)<br>» Encourage open communication within constraints of confidentiality<br>» Organize communications about logistics<br>» Encourage participation in meetings, expressing one’s opinions, being aware of others’ needs, and being appropriately assertive when required<br>» Apply perspective into the point of view of others, understanding their needs and goals

» Encourage building trust, rapport and credibility with others
» Anticipate the communication needs and concerns of others and respond to them
» Avoid conflict between work and personal interests
» Encourage meaningful two-way communication
» Influence others by persuasively presenting thoughts and ideas
» Apply assertiveness skills to deal with unreasonable requests and/or refusals that would compromise practice or consumer care
» Maintain a positive, supportive and appreciative attitude
» Apply the ability to balance the organisation's needs and the customer's needs
» Use self-control by maintaining composure and keeping emotions in check, even in difficult situations; deal calmly and effectively with stressful situations
» Encourage the expressing of opinions and providing information in written and/or verbal form in a manner that does not elicit concern, anger or other adverse response
» Apply the use of a systematic process for following up that demonstrates written reports have been received and understood
» Describe the means by which responses to input to the work environment are monitored
» Seek practical ways to overcome barriers to communication
» Identify language representation (e.g., interpreters) at meetings when appropriate
» Tackle difficult situations and resolve disputes between staff
» Give both positive and negative feedback sensitively
» Maintain and improve communication
» Apply the process of giving and requesting feedback frequently
» Apply the ability to communicate effectively with shop stewards and union representatives

6.2.1 Practice cultural awareness

» Describe cultural awareness and sensitivity, treating all people with fairness, respect and dignity
» Describe anti-discriminatory practices in the organisation, including HR and disciplinary procedures
» Establish cultural sensitivity, equality and fairness at all levels of the organisation
» Challenge discriminatory behaviour directly and sensitively
» Act in a non-discriminatory way toward individuals and groups
» Integrate cultural awareness in learning and development approaches
» Avoid stereotypical responses by examining one's own behaviour and bias
» Manage cultural diversity in teams and make the most of differences
» Describe strategies and/or resources for communicating effectively with people from different cultural backgrounds

» Elicit information relating to values, beliefs and cultural backgrounds of consumers that may influence the way professional services are provided

Basal Technology Competency(ies):	7.1 Data Science<br>7.4 Temperature and monitoring<br>7.5 Planning systems<br>7.6 ERP includes function of LMIS<br>7.12 Basic Office Skills<br>7.13 Have a command of technology
**7.1 Data Science**	» Describe the end to end MIS technology components that are inherent in advanced MIS systems and explore how these may be relevant in different supply chain contexts<br>» Identify emerging technological approaches to data visualization and analysis<br>» Identify how data science can be applied within supply chain<br>» Ability to interact, provide guidance for data (dashboard) consolidation in various levels and use the tech tools<br>» Support movement of data into electronic form at lowest/remote SC level<br>» Ability to work with relational database
**7.4 Temperature and monitoring**	» Describe the current technology approaches for Temperature Monitoring Sensors<br>» Describe what is required to implement this technology in a country context<br>» Describe infrastructure requirement for heat-sensitive items at peripheral ‘lower level’ of SC depending on volume and nature of items to handle;<br>» Define regular preventive maintenance and calibration of cold-chain equipment with biomedical engineers or logisticians<br>» Describe the policy for temperature monitoring tools during transportation and storage<br>» Describe the steps involved in health logistic management cycle: selection-order-delivery-reception-storage-distribution-inventory<br>» Describe hardware qualification and system validation of temperature monitoring<br>» Consider mass data management of temperature monitoring
**7.5 Planning systems**	» Describe blockchain and understand the building blocks for planning systems<br>» Describe planning systems as a value-add capability for relevant global health use cases
**7.6 ERP includes function of LMIS**	» Support a Master Data Management System<br>» Describe the critical components of an ERP system

	» Describe the meaning of common document types found within an ERP/LMIS<br>» Describe the logic of data visibility within an ERP<br>» Describe the importance of data integrity, record management and information security
7.12 Basic Office Skills	» Have a good understanding of common presentation authoring packages<br>» Have a good understanding of common spreadsheet authoring packages<br>» Have a good understanding of common document authoring packages<br>» Have a good understanding of common email authoring packages<br>» Have a good understanding of common instant messaging packages<br>» Have a good understanding of common video conferencing authoring packages
7.13 Have a command of technology	» Apply the use of technology suitable to the job held<br>» Provide input on monitoring new developments and technologies in the sector<br>» Identify new technologies and recognising potential benefits for the sector<br>» Identify resources and support that are provided across the organisation to enable colleagues to make the best use of available technology<br>» Ensure that the organisation has a strategy for technology use<br>» Describe how to minimise environmental damage through technology use<br>» Use technology to maximise effectiveness and efficiency<br>» Use field-based technology (e.g., radio, general packet radio service (GPRS), satellite phone)
Advanced Technology Competency(ies):	7.2 Blockchain<br>7.3 Unmanned Aerial Vehicles (UAVs)<br>7.7 Automation<br>7.8 Artificial Intelligence<br>7.9 Additive Manufacturing<br>7.10 Internet of things<br>7.11 Cloud Computing<br>7.14 eProcurement
7.2 Blockchain	» Describe blockchain as a value-add capability for relevant global health use cases
7.3 Unmanned Aerial Vehicles (UAVs)	» Describe the current UAV landscape, their application and the enabling environment required to support country implementation<br>» Describe UAVs as a value-add capability for relevant global health use cases

	» Describe the laws/ regulations around the use of UAV's
7.7 Automation	» Describe automation as a value-add capability for relevant global health use cases
7.8 Artificial Intelligence	» Describe artificial intelligence as a value-add capability for relevant global health use cases
7.9 Additive Manufacturing	» Describe additive manufacturing as a value-add capability for relevant global health use cases
7.10 Internet of things (IoT)	» Understand the opportunity of IoT used in conjunction with AI<br>» Describe the internet of things as a value-add capability for relevant global health use cases
7.11 Cloud Computing	» Describe cloud computing as a value-add capability for relevant global health use cases
7.14 eProcurement	» Describe how to transact with an eProcurement system where necessary<br>» Describe eProcurement as a value-add capability for relevant global health use cases<br>» Apply the benefits and limitations of eProcurement to best utilise the system

Key Performance Indicators:

- Assess Delivery Performance Cycle Time - Actual Time from assessed requirement to time delivered to end-user compared to committed Delivery Cycle Time as per Supply Chain strategy
- Assess Supplier Performance Cycle Time - Actual Supplier performance Cycle time versus Supplier commitment cycle time
- Inventory accuracy - Cycle count results
- Transportation lead time accuracy - Actual Transportation lead time versus Transportation promised lead time.
- Transport efficiency - Total cost of transport versus planned cost of transport by mode
- In-bound cost of acquisition - Actual total cost of acquisition versus Total Planned cost of acquisition including landed costs
- Manage performance cycle time
- Performance Report - Actual versus Plan reports for all metrics

Training:	Qualifications Available:	Certifications Available:
» Data management<br>» ERP Systems<br>» Installed base management<br>» Interpreting Specifications<br>» Office automation tools<br>» Product Information Management (Product Data Management)	» National Diploma: Financial Markets<br>» National Diploma: Transport Economics<br>» Bachelor of Commerce in Finance<br>» Bachelor of Commerce in Transport Economics<br>» Bachelor of Commerce in Information Systems	» Certified Supply Chain Analyst (CSCA-ISCEA)

Manager - HR/ Workforce Development

Organisation: SC-focused Organisation				
Competency Level:	Serial Number:	Primary Process:	Job Role:	Supervises:
Managerial	5-571-572-4	Enable – Human Resources	Manager - HR/ Workforce Development	Lead - HR/ Workforce Development

Contributions	Maintains and enhances the Human Resources Enabling capability of the Public Health System Organisation. Coordinates with Executive and Operations to ensure that the Human Resource capacity is capable of supporting the Public Health Supply Chain Strategy.
Personal/Management Competency(ies):	5.5 Manage and plan projects<br>5.5.2 Execute strategic decision making<br>5.7 Oversee/support human resources (e.g., recruitment, training, team management/ supervision)<br>5.7.1 Manage staff recruitment process<br>5.7.2 Train staff<br>5.7.3 Supervise staff<br>5.7.4 Assessing HR systems<br>6.5 Prove leadership abilities<br>6.5.1 Demonstrate resilience and ability to manage stress<br>6.6 Abide by rules / laws / legislation

5.5 Manage and plan projects

5.5.2 Execute strategic decision making

» Determine how one can make decisions regarding one's own workload and area of responsibility

» Determine the process of prioritizing competing tasks and perform them quickly and efficiently according to their importance; finding new ways of organizing or planning work to accomplish it more efficiently

» Evaluate the proper time and space for consultation around decisions being made.

» Demonstrate the process of considering the input of resources including staff needed

» Analyse assumptions against facts

» Determine the key issues in a complex situation and come to the heart of the problem quickly

» Evaluate relevant information before making decisions

» Provide input to strategic decisions in the interest of the organisation's goals

	» Evaluate when to analyse, evaluate and execute tough decisions when necessary<br>» Analyse the impact of decisions on others' work or team goals<br>» Communicate decisions and ensure they are incorporated into policies and processes<br>» Demonstrate inclusive and consultative strategic planning to establish strategic objectives<br>» Evaluate emerging trends and practices<br>» Analyse the macro and long-term consequences of decisions
5.7 Oversee/support human resources (e.g., recruitment, training, team management/ supervision)	
5.7.1 Manage staff recruitment process	» Demonstrate use of a system for staff recruitment, appraisals and monitoring<br>» Evaluate training and recruitment plans to fill any identified gaps and shortages<br>» Determine relevant and appropriate selection criteria for a defined role<br>» Analyse and demonstrate the standard selection documentation for recruitment<br>» Determine key issues impacting the size and membership of an interview panel (e.g., logistics, gender balance, absence of conflict of interest)<br>» Analyse requirements for a fair, defensible and balanced interview process<br>» Analyse and demonstrate the documentation maintained for each interviewed position<br>» Determine due process for formalising an appointment that minimises the opportunity for untoward effects (e.g., appeal, conflict, resignation)<br>» Analyse job descriptions to obtain and retain skilled staff<br>» Consult with and involve relevant personnel in development and updating of role descriptions/duty statements that clarify the duties and responsibilities of positions<br>» Determine the process of orienting new staff to the workplace, explaining standard operating systems and procedures<br>» Evaluate human resource plans to meet the future staffing needs of the organisation<br>» Evaluate key features and measures used for monitoring a personnel retention strategy (e.g., recruitment costs, staff turnover, staff surveys)<br>» Determine the legislative issues impacting human resource policies and procedures<br>» Evaluate the process of maintaining employee master data including medicals, allergies to ensure safe working environment<br>» Analyse an employee opinion survey

5.7.2 Train staff

- Determine the process of maintaining a list of staff that require logistics system training (based on supportive supervision reports)
- Coordinate all training in managing the logistics system for all health facility staff
- Promote participation in relevant learning and development opportunities
- Demonstrate the process of assisting in training nurses and other health professionals in medicines ordering and storage procedures
- Demonstrate the process of orienting new doctors to the formulary and the systems and procedures of the medicines supply system
- Determine the process of providing on-the-job training to district health officials and hospital pharmacists as needed
- Demonstrate the process of ensuring staff have the necessary skills and understanding for safe practice in the event they need to fill a management role due to absence or illness
- Evaluate the process of staff handling hazardous materials receive training on safety & compliance regulations
- Evaluate any staff needs at all levels of the supply chain
- Analyse key factors important for initiating and/or sustaining the motivation of adult learners
- Evaluate the variable learning needs and styles or modalities in adult learners (e.g., visual versus auditory)
- Evaluate a range of strategies that could be used to reinforce and clarify educational content (e.g., tutorials or workshops, written materials, intranet- or internet-based resources)
- Evaluate the use of train-the-trainer to equipment trainers
- Analyse training effectiveness monitoring and training feedback systems

5.7.3 Supervise staff

- Determine the use of a performance management process with all personnel to ensure continuous improvement
- Determine an environment that is supportive of learning and professional development
- Demonstrate the nature of the supervisory role, what is meant by direct supervision, and where responsibility for outputs and outcomes rests
- Plan and conduct supervisory tours of dependent facilities
- Analyse situations where supervised personnel are experiencing difficulties in completing work activities and/or where a mandatory notification obligation exists
- Analyse signs/cues from supervised personnel that indicate additional guidance or support is needed (e.g., hesitancy, distress, seeks clarification from less authoritative sources)
- Demonstrate a practise of providing feedback, encouragement and support to team members
- Analyse areas where performance should/could be improved and provide resources to support this
- Demonstrate the process of discussing expectations, achievements and contributions with personnel in a fair and equitable manner

	» Support colleagues in creating a professional development plan and suggest ways the plan may be progressed through relevant training and/or experiential learning opportunities<br>» Demonstrate a culture of encouraging and supporting high-potential employees to accept growth challenges outside their own areas<br>» Demonstrate the process of providing progression opportunities for employees who have prepared themselves through development<br>» Evaluate the process of identifying and dealing with unproductive staff using an approved process in an ethical manner<br>» Evaluate and address work issues contributing to impairment of personnel (e.g., excessive workload, conflict)<br>» Determine the complaints management process<br>» Evaluate situations where additional expertise should be sought (e.g., counselling in the event of workplace violence or in situations of diminished performance due to drug or alcohol abuse)<br>» Demonstrate a culture of acknowledging and respecting different working styles
5.7.4 Assessing HR systems	» Ability to assess HR systems
6.5 Prove leadership abilities	» Analyse input to meetings and programme development<br>» Look for future trends and issues, and assist the organisation in meeting the challenges<br>» Contribute to a collaborative working environment<br>» Demonstrate a drive for change and improvement develops opportunities for the organisation and sector<br>» Demonstrate channelling energy and ideas toward resolving issues<br>» Demonstrate the practise of communicating with influence<br>» Demonstrate showing courage to take an unpopular stance when needed<br>» Demonstrate steering and implementing change organisationally<br>» Demonstrate anticipating and resolving conflict<br>» Demonstrate showing initiative in working methods<br>» Exemplify personal drive and integrity<br>» Demonstrate serving as a role model for others to follow<br>» Demonstrate leadership and practice management skills, initiative and efficiency<br>» Demonstrate inspiring others through professional excellence, innovation and communication<br>» Determine situations in which a change in leadership style would be warranted (e.g., directive versus consultative style in the event of a crisis)
6.5.1 Demonstrate resilience and ability to manage stress	» Demonstrate identifying symptoms of stress and take steps to reduce stress<br>» Demonstrate seeing the bigger picture and help others to

	» Demonstrate recovering quickly from setbacks<br>» Demonstrate drawing on previous experience and support mechanisms to reduce the impact of stress on self and others<br>» Determine a working environment that aims to minimise pressure and stress<br>» Demonstrate coping well under pressure, particularly in difficult environments<br>» Demonstrate acting as a role model for others and display courage under difficult circumstances<br>» Demonstrate recognising the limitations of staff and taking action to limit their exposure to harm when needed<br>» Demonstrate making use of personal support mechanisms<br>» Demonstrate helping others identify personal support mechanisms<br>» Determine organisational policy to support self-care in agencies
6.6 Abide by rules / laws / legislation	» Demonstrate compliance with the legislation that covers the practice of public health supply chains and healthcare, and describe its purpose<br>» Analyse requirements of professional codes, guidelines and standards adopted as part of the legislative framework<br>» Demonstrate keeping up to date with changes in legal instruments, as informed by national-level managers<br>» Demonstrate compliance with international regulations in decision making for the distribution system, including customs regulations on import and export requirements (e.g., trade tariffs and duties on imported goods) and security regulations (e.g., 2007 SAFE Ports Act, NAFTA or European Union trade agreements)<br>» Demonstrate applying and understanding regulatory affairs and the key aspects of pharmaceutical registration and legislation<br>» Demonstrate knowledge of the principles of business economics and intellectual property rights, including the basics of patent interpretation<br>» Demonstrate knowledge of applicable procurement law and the practical effects of a contract’s terms and conditions<br>» Evaluate current industry and government regulations governing sustainability<br>» Demonstrate a working knowledge of key hazardous materials handling laws<br>» Determine policies and procedures relating to workplace safety that are consistent with agreed or recognised standards<br>» Analyse professional standards and conventions, as well as workplace policies and procedures, for preparing pharmaceutical products<br>» Demonstrate all standard operating procedures<br>» Evaluate the process of complying with data privacy and data protection laws
Basal Technology Competency(ies):	7.12 Basic Office Skills<br>7.13 Have a command of technology

7.12 Basic Office Skills	» Demonstrate a good understanding of common presentation authoring packages<br>» Demonstrate a good understanding of common spreadsheet authoring packages<br>» Demonstrate a good understanding of common document authoring packages<br>» Demonstrate a good understanding of common email authoring packages<br>» Demonstrate a good understanding of common instant messaging packages<br>» Demonstrate a good understanding of common video conferencing authoring packages
7.13 Have a command of technology	» Demonstrate the use of technology suitable to the job held<br>» Monitor new developments and technologies in the sector<br>» Experiment with new technologies and recognising potential benefits for the sector<br>» Ensure that resources and support are provided across the organisation to enable colleagues to make the best use of available technology<br>» Determine what the organisations' strategy is for technology use<br>» Determine how to minimise environmental damage through technology use<br>» Analyse the use of technology to maximise effectiveness and efficiency<br>» Analyse the use of field-based technology (e.g., radio, general packet radio service (GPRS), satellite phone)
Advanced Technology Competency(ies):	7.11 Cloud Computing
7.11 Cloud Computing	» Evaluate cloud computing as a value-add capability for relevant global health use cases

Key Performance Indicators:

» Competency compliance. No of personnel evaluated and found to competent versus plan.

» Number of personnel with the required Skill, Education, Training and Aptitude as per Supply Chain Strategy versus Total number of personnel.

» Number of personnel progressed professionally versus Number of personnel that were targeted for progress.

» Planned skills upliftment plan - Number of personnel per competency versus actual skills upliftment Number of personnel per competency.

» HR4SCM Competency Requirements & Plan

» Creation of the HR4SCM Competency Requirements & Plan based on the Supply Chain Requirements

» Number of personnel with the required Skill, Education, Training and Aptitude as per Supply Chain Strategy versus Total number of personnel.

» Number of personnel progressed professionally versus Number of personnel that were targeted for progress.
» Planned skills upliftment plan - Number of personnel per competency versus actual skills upliftment Number of personnel per competency.
» Competency compliance. No of personnel evaluated and found to competent versus plan.
» HR4SCM Competency Requirements & Plan
» Direct Labour Cost
» Human Resource Contract Management
» EHS regulations
» Legislation and Standards
» Performance Management

Training:	Qualifications Available:	Certifications Available:
» Performance Management<br>» Contract Management<br>» EHS regulations<br>» Legislation and Standards<br>» Outsourcing	» Diploma in Human Resource Management	» Occupational Certificate: Health Information Manager

Manager – Contracts and Agreements

Organisation:

SC-focused Organisation

Competency Level:	Serial Number:	Primary Process:	Job Role:	Supervises:
Managerial	2-242-23-3	Enable – Contracts Agreements	Manager – Contracts and Agreements	Manager - Contract and 3PLs, Specialist – Contracts

Contributions	Maintains and enhances the Contractual capacity of the Public Health System Organisation. Ensures that the Enable processes are adhered to as prescribed in the Public Health System Organisation's Operating Model. Maintains contract and agreement records current and relevant and deploys appropriate governance methodology to each contract or agreement.
Technical Competency(ies):	2.3 Manage tendering processes and supplier agreements<br>2.4 Undertake contract management and risk<br>2.4.1 Place commodity orders<br>2.4.2 Manage contracts<br>2.4.3 Addresses risk and ensures quality management

2.3 Manage tendering processes and supplier agreements

- Demonstrate the correct use of request for qualifications (RFQ), invitations to bid (ITB) and request for proposals (RFP) methods
- Demonstrate knowledge of local and international tendering procedures (e.g., open competitive bidding, restricted tender, competitive negotiation, direct procurement)
- Write detailed specifications for tenders
- Describe the high-level guidance required for high-value and politically sensitive procurements
- Describe the process of developing and managing contracts
- Describe the procurement, return and exchange policies
- List appropriate legislation and policy in regard to procurement processes, and what is required to comply
- Ensure ethical behaviour, abiding by conflict of interest policies
- Describe the process of evaluating tender bids based on the specification and evaluation criteria including capability of the supplier, problems relating to outcomes of the contract.
- Follow contract approval process, including contract negotiations
- Discuss some of the key issues relevant to negotiating supply contracts (e.g., volume usage over time, price volume agreements, alternate supplier clauses, duration of contract, period of review, terms for contract termination and renewal)
- Describe the process of awarding and managing contracts, resolving any issues as they arise
- Describe the process of debriefing unsuccessful suppliers

- Describe the ways in which compliance with purchasing policies and procedures is monitored
- Participate in procurement planning including a yearly activities schedule and developing a wide range of briefs, specifications and commercial documentation
- Identify strategic sourcing projects, capturing benefits, work requirements, impacts and cost savings
- Participate in cross-functional teams handling strategic procurements
- Describe effective internal and external procurement networks
- List local and international health commodity prices and understand the factors affecting them
- Describe the process of engaging clients across the public sector in presentations demonstrating the benefits of a range of strategic projects designed to ensure cost savings and value for money for government procurement

2.4 Undertake contract management and risk

2.4.1 Place commodity orders

- Obtain authorisations to administer the contracts
- Describe the type of supplies required
- Obtain reference sources and supplier catalogues to clarify required product and its availability
- Comply with policies and procedures to order required stock and equipment
- Confirm the orders with suppliers
- Identify any problems in placing orders
- Comply with procedures for placing orders
- Identify any problems with order delivery and recommend options for progressing order delivery
- Ensure effective mechanisms for checking invoices exist and take action to adjust payments accordingly
- Receive or raise requisitions and arrange purchase orders and payments

2.4.2 Manage contracts

- Conduct reviews of performance against agreed key performance indicators
- Provide timely and expert guidance to remediate procurement performance issues
- Describe contract responsiveness and negotiate changes to a contract (contract variations)
- Manage contract disputes and terminate contracts legally and appropriately if necessary
- Describe the importance of supplier control mechanisms, contract administration and supplier management

2.4.3 Addresses risk and ensures quality management

- Describe the process of providing risk assessments for the procurement process (technical, commercial, administrative)

- Use key performance indicators for assessing procurement and supplier performance and effectiveness
- Identify any problems with the procurement of supplies in the supply chain
- Identify the contingency plan for shortages
- Describe recommended options for improving the performance of suppliers
- Apply procurement portfolio analysis and risk assessment (supply positioning)
- Describe the fundamentals of risk planning and assessment

Personal/Management Competency(ies):	6.1 Demonstrate generic skills (e.g., literacy, numeracy, technology)<br>6.1.1 Exhibit high understanding of literacy and numeracy<br>6.2 Demonstrate strong communication skills<br>6.2.1 Practice cultural awareness

6.1 Demonstrate generic skills (e.g., literacy, numeracy, technology)

6.1.1 Exhibit high understanding of literacy and numeracy

- Speak clearly and confidently, organize information in a logical manner, and consider voice tone and pace
- Respond to verbal messages and other cues (active listening)
- Express ideas and opinions clearly in written and verbal form
- Communicate information accurately, concisely and confidently in writing and verbally
- Practice a level of mathematics suitable to the job held
- Describe visual presentation techniques, including charting, histograms and flow sheets
- Read and comprehend at a level necessary to properly complete duties of the position
- Interpret written information in documents, such as reports, SOPs, LMIS forms, graphs, calendars, schedules, notices and directions
- Pay attention to detail and identify the main ideas, detect inconsistencies and identify missing information in documents
- Apply computer skills — such as using Word, Excel, PowerPoint and the internet — suitable to the job level
- Describe the process of analysing and validating KPIs
- Participate in the evaluation of activities that act as standards against which the KPIs are measured
- Apply the process of performing critical self-review on work before submission

6.2 Demonstrate strong communication skills

- Be truthful and trustworthy, and supply accurate information at all times
- Work as part of a workplace team

	» Describe vocabulary and communication style and form for both written and verbal communication that is appropriate for the situation, audience and material being communicated (e.g., avoid unnecessary jargon, clearly explain medical and SCM terminology)<br>» Encourage open communication within constraints of confidentiality<br>» Organize communications about logistics<br>» Encourage participation in meetings, expressing one's opinions, being aware of others' needs, and being appropriately assertive when required<br>» Apply perspective into the point of view of others, understanding their needs and goals<br>» Encourage building trust, rapport and credibility with others<br>» Anticipate the communication needs and concerns of others and respond to them<br>» Avoid conflict between work and personal interests<br>» Encourage meaningful two-way communication<br>» Influence others by persuasively presenting thoughts and ideas<br>» Apply assertiveness skills to deal with unreasonable requests and/or refusals that would compromise practice or consumer care<br>» Maintain a positive, supportive and appreciative attitude<br>» Apply the ability to balance the organisation's needs and the customer's needs<br>» Use self-control by maintaining composure and keeping emotions in check, even in difficult situations; deal calmly and effectively with stressful situations<br>» Encourage the expressing of opinions and providing information in written and/or verbal form in a manner that does not elicit concern, anger or other adverse response<br>» Apply the use of a systematic process for following up that demonstrates written reports have been received and understood<br>» Describe the means by which responses to input to the work environment are monitored<br>» Seek practical ways to overcome barriers to communication<br>» Identify language representation (e.g., interpreters) at meetings when appropriate<br>» Tackle difficult situations and resolve disputes between staff<br>» Give both positive and negative feedback sensitively<br>» Maintain and improve communication<br>» Apply the process of giving and requesting feedback frequently<br>» Apply the ability to communicate effectively with shop stewards and union representatives
6.2.1 Practice cultural awareness	» Describe cultural awareness and sensitivity, treating all people with fairness, respect and dignity<br>» Describe anti-discriminatory practices in the organisation, including HR and disciplinary procedures

	» Establish cultural sensitivity, equality and fairness at all levels of the organisation<br>» Challenge discriminatory behaviour directly and sensitively<br>» Act in a non-discriminatory way toward individuals and groups<br>» Integrate cultural awareness in learning and development approaches<br>» Avoid stereotypical responses by examining one's own behaviour and bias<br>» Manage cultural diversity in teams and make the most of differences<br>» Describe strategies and/or resources for communicating effectively with people from different cultural backgrounds<br>» Elicit information relating to values, beliefs and cultural backgrounds of consumers that may influence the way professional services are provided
Basal Technology Competency(ies):	7.6 ERP includes function of LMIS<br>7.12 Basic Office Skills<br>7.13 Have a command of technology
7.6 ERP includes function of LMIS	» Support a Master Data Management System<br>» Describe the critical components of an ERP system<br>» Describe the meaning of common document types found within an ERP/LMIS<br>» Describe the logic of data visibility within an ERP<br>» Describe the importance of data integrity, record management and information security
7.12 Basic Office Skills	» Have a good understanding of common presentation authoring packages<br>» Have a good understanding of common spreadsheet authoring packages<br>» Have a good understanding of common document authoring packages<br>» Have a good understanding of common email authoring packages<br>» Have a good understanding of common instant messaging packages<br>» Have a good understanding of common video conferencing authoring packages
7.13 Have a command of technology	» Apply the use of technology suitable to the job held<br>» Provide input on monitoring new developments and technologies in the sector<br>» Identify new technologies and recognising potential benefits for the sector<br>» Identify resources and support that are provided across the organisation to enable colleagues to make the best use of available technology<br>» Ensure that the organisation has a strategy for technology use

	» Describe how to minimise environmental damage through technology use<br>» Use technology to maximise effectiveness and efficiency<br>» Use field-based technology (e.g., radio, general packet radio service (GPRS), satellite phone)
Advanced Technology Competency(ies):	7.2 Blockchain<br>7.11 Cloud Computing
7.2 Blockchain	» Describe blockchain as a value-add capability for relevant global health use cases
7.11 Cloud Computing	» Describe cloud computing as a value-add capability for relevant global health use cases

Key Performance Indicators:

» On Time Closure of Tenders
» Procurement cycle time
» Tender creation cycle time
» Accuracy of tender documents
» Quality of Supplier Relationship
» Tenders adjudication and allocation on Time performance
» Tenders allocated in accordance with Business Rules
» On Time Closure of Tenders
» Vendor/Supplier Master Data Accuracy
» Item Master Data Accuracy
» Risk avoidance, elimination and or Mitigation Costs
» inbound Transportation Master Data Accuracy by Item SKU
» Item SKU Lead Time Accuracy

Training:	Qualifications Available:	Certifications Available:
» Performance Management<br>» Contract Management<br>» Outsourcing<br>» Legal Frameworks<br>» Basic Transportation Management<br>» Consignment Agreement Development		» CIPS Level 4 Diploma in Procurement and Supply<br>» SPSM2® (Global Procurement Management)<br>» SPSM ® (Essential Procurement Skills)

» Customer/Supplier Communication<br>» Intellectual Property/Proprietary Data<br>» International Trade<br>» Procurement<br>» Subcontracting Types (FFP, CP, CPAF, Performance Based)<br>» Supplier Relationship Management (SRM)		

Manager – Programmes (Donations) and Global Partnerships

Organisation:

SC-focused Organisation

Competency Level:	Serial Number:	Primary Process:	Job Role:	Supervises:
Managerial	2-26-27-4	Procure	Manager – Programmes (Donations) and Global Partnerships	Officer – Programmes (Donations) Procurement

Contributions	Maintains productive relationships with the donor organisations and global partners. Monitors the established metrics for the supply of goods and services by donors and global partners. Seeks to improve the efficiency of the donor and global partnerships by using the Source and Procure processes as defined in the Public Health System Organisation's Operating Model.
Technical Competency(ies):	2.6 Manage import and export of products<br>2.7 Manage donations of products

2.6 Manage import and export of products

- » Ensure adherence to the import, export and customs procedures and requirements
- » Determine import or export requirements for the supplies
- » Evaluate import or export documentation for the supplies
- » Evaluate the import and export procedures
- » Evaluate any problems with the import and export procedures and requirements
- » Demonstrate knowledge about international business processes (e.g. shipping, air cargo, clearing and forwarding, financial transactions, business ethics)

2.7 Manage donations of products		» Follow the national donations policy, referring to the appropriate Ministry and customs office for advice<br>» Support policies and procedures that enable the ability to say no to donations that are not consistent with national donation policy<br>» Demonstrate the ability to manage donation plan in emergency situations
Personal/Management Competency(ies):	5.5 Manage and plan projects<br>5.5.2 Execute strategic decision making<br>6.5 Prove leadership abilities<br>6.5.1 Demonstrate resilience and ability to manage stress<br>6.6 Abide by rules / laws / legislation	
5.5 Manage and plan projects		
5.5.2 Execute strategic decision making		» Determine how one can make decisions regarding one's own workload and area of responsibility<br>» Determine the process of prioritizing competing tasks and perform them quickly and efficiently according to their importance; finding new ways of organizing or planning work to accomplish it more efficiently<br>» Evaluate the proper time and space for consultation around decisions being made.<br>» Demonstrate the process of considering the input of resources including staff needed<br>» Analyse assumptions against facts<br>» Determine the key issues in a complex situation and come to the heart of the problem quickly<br>» Evaluate relevant information before making decisions<br>» Provide input to strategic decisions in the interest of the organisation's goals<br>» Evaluate when to analyse, evaluate and execute tough decisions when necessary<br>» Analyse the impact of decisions on others' work or team goals<br>» Communicate decisions and ensure they are incorporated into policies and processes<br>» Demonstrate inclusive and consultative strategic planning to establish strategic objectives<br>» Evaluate emerging trends and practices<br>» Analyse the macro and long-term consequences of decisions
6.5 Prove leadership abilities		» Analyse input to meetings and programme development<br>» Look for future trends and issues, and assist the organisation in meeting the challenges<br>» Contribute to a collaborative working environment<br>» Demonstrate a drive for change and improvement develops opportunities for the organisation and sector

	» Demonstrate channelling energy and ideas toward resolving issues<br>» Demonstrate the practise of communicating with influence<br>» Demonstrate showing courage to take an unpopular stance when needed<br>» Demonstrate steering and implementing change organisationally<br>» Demonstrate anticipating and resolving conflict<br>» Demonstrate showing initiative in working methods<br>» Exemplify personal drive and integrity<br>» Demonstrate serving as a role model for others to follow<br>» Demonstrate leadership and practice management skills, initiative and efficiency<br>» Demonstrate inspiring others through professional excellence, innovation and communication<br>» Determine situations in which a change in leadership style would be warranted (e.g., directive versus consultative style in the event of a crisis)
6.5.1 Demonstrate resilience and ability to manage stress	» Demonstrate identifying symptoms of stress and take steps to reduce stress<br>» Demonstrate seeing the bigger picture and help others to<br>» Demonstrate recovering quickly from setbacks<br>» Demonstrate drawing on previous experience and support mechanisms to reduce the impact of stress on self and others<br>» Determine a working environment that aims to minimise pressure and stress<br>» Demonstrate coping well under pressure, particularly in difficult environments<br>» Demonstrate acting as a role model for others and display courage under difficult circumstances<br>» Demonstrate recognising the limitations of staff and taking action to limit their exposure to harm when needed<br>» Demonstrate making use of personal support mechanisms<br>» Demonstrate helping others identify personal support mechanisms<br>» Determine organisational policy to support self-care in agencies
6.6 Abide by rules / laws / legislation	» Demonstrate compliance with the legislation that covers the practice of public health supply chains and healthcare, and describe its purpose<br>» Analyse requirements of professional codes, guidelines and standards adopted as part of the legislative framework<br>» Demonstrate keeping up to date with changes in legal instruments, as informed by national-level managers<br>» Demonstrate compliance with international regulations in decision making for the distribution system, including customs regulations on import and export requirements (e.g., trade tariffs and duties on imported

	goods) and security regulations (e.g., 2007 SAFE Ports Act, NAFTA or European Union trade agreements)<br>» Demonstrate applying and understanding regulatory affairs and the key aspects of pharmaceutical registration and legislation<br>» Demonstrate knowledge of the principles of business economics and intellectual property rights, including the basics of patent interpretation<br>» Demonstrate knowledge of applicable procurement law and the practical effects of a contract’s terms and conditions<br>» Evaluate current industry and government regulations governing sustainability<br>» Demonstrate a working knowledge of key hazardous materials handling laws<br>» Determine policies and procedures relating to workplace safety that are consistent with agreed or recognised standards<br>» Analyse professional standards and conventions, as well as workplace policies and procedures, for preparing pharmaceutical products<br>» Demonstrate all standard operating procedures<br>» Evaluate the process of complying with data privacy and data protection laws
Basal Technology Competency(ies):	7.6 ERP includes function of LMIS<br>7.12 Basic Office Skills<br>7.13 Have a command of technology
7.6 ERP includes function of LMIS	» Evaluate a Master Data Management System<br>» Demonstrate an understanding of the critical components of an ERP system<br>» Demonstrate an understanding of the meaning of common document types found within an ERP/LMIS<br>» Demonstrate an understanding of the logic of data visibility within an ERP<br>» Demonstrate the importance of data integrity, record management and information security
7.12 Basic Office Skills	» Demonstrate a good understanding of common presentation authoring packages<br>» Demonstrate a good understanding of common spreadsheet authoring packages<br>» Demonstrate a good understanding of common document authoring packages<br>» Demonstrate a good understanding of common email authoring packages<br>» Demonstrate a good understanding of common instant messaging packages<br>» Demonstrate a good understanding of common video conferencing authoring packages

7.13 Have a command of technology	» Demonstrate the use of technology suitable to the job held<br>» Monitor new developments and technologies in the sector<br>» Experiment with new technologies and recognising potential benefits for the sector<br>» Ensure that resources and support are provided across the organisation to enable colleagues to make the best use of available technology<br>» Determine what the organisations' strategy is for technology use<br>» Determine how to minimise environmental damage through technology use<br>» Analyse the use of technology to maximise effectiveness and efficiency<br>» Analyse the use of field-based technology (e.g., radio, general packet radio service (GPRS), satellite phone)
Advanced Technology Competency(ies):	7.2 Blockchain<br>7.11 Cloud Computing
7.2 Blockchain	» Evaluate blockchain as a value-add capability for relevant global health use cases
7.11 Cloud Computing	» Evaluate cloud computing as a value-add capability for relevant global health use cases

Key Performance Indicators:

- Accuracy of Risk Register
- Cycle time of QA/QC report
- Overall value at risk
- Partner/Donor integration, policy, guidelines and regulatory reforms
- QA/QC report
- Risk / Mitigation Costs
- Time to Recovery (TTR)
- Partner/Donor feedback on donations - actual reach of programmes versus planned reach
- Partner Donation yield - Partner/Donor feedback on donations - number and quantity of items / SKU donated versus number of items and quantity wasted.

Training:	Qualifications Available:	Certifications Available:
» Contract Management<br>» Consignment Agreement Development<br>» Customer/Supplier Communication	» Bachelor of Commerce in Philosophy, Politics and Economics<br>» Bachelor of Science Honours: Pharmacology	» SPSM3 TM (Enterprise-Wide Procurement Influence)<br>» APICS Certified in production and inventory management certificate

» International Trade<br>» Procurement<br>» Subcontracting Types (FFP, CP, CPAF, Performance Based)<br>» Supplier Relationship Management (SRM)	» Bachelor of Accounting Honours	

Operational
Officer– Procurement

Organisation:
SC-focused Organisation

Competency Level:	Serial Number:	Primary Process:	Job Role:	Supervises:
Operational	2-21-22-2	Enable – Procurement	Officer– Procurement	Officer – Procurement and Supply

Contributions	Maintains the procurement process, to ensure that materials and resources are available when needed and within specifications stipulate in the Procurement business rules.
Technical Competency(ies):	2.1 Manage procurement costs and budget<br>2.2 Build and maintain supplier relationships

2.1 Manage procurement costs and budget

- Understand public procurement regulations
- Understand the importance of budget approval before initiating procurement
- Understanding the importance of financial and management accounting practices within procurement
- Understand the commercial factors that contribute toward cost reduction, price savings and value improvement opportunities
- Understand the principles of foreign exchange rates and how they impact prices

2.2 Build and maintain supplier relationships

- Understand the factors in the analysis of the market
- Understand the processes for prequalification and tender contracting
- Understand the factors that define a good supplier
- Understand that key suppliers must be located and that there is a total cost associated with procuring an item or service
- Understand supplier relationship management plans (e.g., sourcing, frequency of meetings, negotiate and monitor benchmarks for performance)
- Understand the process of educating suppliers to create value for customers by streamlining processes in the value chain
- Understand the difference between strategic and non-strategic suppliers and the associated supplier management principles
- Understand commercial factors that contribute toward cost reduction, price savings and value improvement opportunities
- Understand the process of strategic sourcing practices drive down cost across consecutive tenders
- Compare the process of working with suppliers to agree cost saving opportunities in the whole value chain, with an appropriate cost sharing mechanism applied to ensure a “win-win” outcome
- Understand the process of managing supplier risk

	» Understand ethical sourcing
Personal/Management Competency(ies):	6.1 Demonstrate generic skills (e.g., literacy, numeracy, technology)<br>6.1.1 Exhibit high understanding of literacy and numeracy<br>6.2 Demonstrate strong communication skills<br>6.2.1 Practice cultural awareness
6.1 Demonstrate generic skills (e.g., literacy, numeracy, technology)	
6.1.1 Exhibit high understanding of literacy and numeracy	» Speak clearly and confidently, organize information in a logical manner, and consider voice tone and pace<br>» Respond to verbal messages and other cues (active listening)<br>» Express ideas and opinions clearly in written and verbal form<br>» Communicate information accurately, concisely and confidently in writing and verbally<br>» Practice a level of mathematics suitable to the job held<br>» Compare visual presentation techniques, including charting, histograms and flow sheets<br>» Read and comprehend at a level necessary to properly complete duties of the position<br>» Understand the need to interpret written information in documents, such as reports, SOPs, LMIS forms, graphs, calendars, schedules, notices and directions<br>» Understand the need to pay attention to detail and identify the main ideas, detect inconsistencies and identify missing information in documents<br>» Enable computer skills — such as using Word, Excel, PowerPoint and the internet — suitable to the job level<br>» Understand the process of analysing and validating KPIs<br>» Understand how the evaluation process of activities that act as standards against which the KPIs are measured is carried out<br>» Examine the process of performing critical self-review on work before submission
6.2 Demonstrate strong communication skills	» Be truthful and trustworthy, and supply accurate information at all times<br>» Enable working as part of a workplace team<br>» Understand vocabulary and communication style and form for both written and verbal communication that is appropriate for the situation, audience and material being communicated (e.g., avoid unnecessary jargon, clearly explain medical and SCM terminology)<br>» Enable open communication within constraints of confidentiality<br>» Participate in the organisation of communications about logistics<br>» Enable participation in meetings, expressing one's opinions, being aware of others' needs, and being appropriately assertive when required

- Show perspective into the point of view of others, understanding their needs and goals
- Enable the practise of building trust, rapport and credibility with others
- Understand the communication needs and concerns of others and respond to them
- Understand the need to avoid conflict between work and personal interests
- Enable meaningful two-way communication
- Show influence on others by persuasively presenting thoughts and ideas
- Show assertiveness skills to deal with unreasonable requests and/or refusals that would compromise practice or consumer care
- Maintain a positive, supportive and appreciative attitude
- Show the ability to balance the organisation's needs and the customer's needs
- Show self-control by maintaining composure and keeping emotions in check, even in difficult situations; deal calmly and effectively with stressful situations
- Enable expressing of opinions and providing information in written and/or verbal form in a manner that does not elicit concern, anger or other adverse response
- Enable the use of a systematic process for following up that demonstrates written reports have been received and understood
- Understand the means by which responses to input to the work environment are monitored
- Seek practical ways to overcome barriers to communication
- Understand the need to ensuring language representation (e.g., interpreters) at meetings when appropriate
- Interpret difficult situations and disputes between staff that need to be tackled and resolved
- Enable giving both positive and negative feedback sensitively
- Understand the need to maintain and improve communication
- Examine the process of giving and requesting feedback frequently
- Examine effective communication with shop stewards and union representatives

6.2.1 Practice cultural awareness

- Understand the need for cultural awareness and sensitivity, treating all people with fairness, respect and dignity
- Enable anti-discriminatory practices in the organisation, including HR and disciplinary procedures
- Enable cultural sensitivity, equality and fairness at all levels of the organisation
- Challenge discriminatory behaviour directly and sensitively
- Act in a non-discriminatory way toward individuals and groups
- Enable integrating cultural awareness in learning and development approaches
- Avoid stereotypical responses by examining one's own behaviour and bias

	» Enable managing cultural diversity in teams and make the most of differences<br>» Compare strategies and/or resources for communicating effectively with people from different cultural backgrounds<br>» Enable the process to elicit information relating to values, beliefs and cultural backgrounds of consumers that may influence the way professional services are provided
Basal Technology Competency(ies):	7.4 Temperature and monitoring<br>7.6 ERP includes function of LMIS<br>7.12 Basic Office Skills<br>7.13 Have a command of technology
7.4 Temperature and monitoring	» Compare the current technology approaches for Temperature monitoring and sensors<br>» Consider what is required to implement this technology in a country context<br>» Compare infrastructure requirement for heat-sensitive items at peripheral 'lower level' of SC depending on volume and nature of items to handle;<br>» Examine regular preventive maintenance and calibration of cold-chain equipment with biomedical engineers or logisticians<br>» Understand the policy for temperature monitoring tools during transportation and storage<br>» Understand the steps involved in health logistic management cycle: selection-order-delivery-reception-storage-distribution-inventory<br>» Understand hardware qualification and system validation of temperature monitoring<br>» Understand mass data management of temperature monitoring
7.6 ERP includes function of LMIS	» Enable a Master Data Management System<br>» Understand the critical components of an ERP system<br>» Understand the meaning of common document types found within an ERP/LMIS<br>» Understand the logic of data visibility within an ERP<br>» Understand the importance of data integrity, record management and information security
7.12 Basic Office Skills	» Have a good understanding of common presentation authoring packages<br>» Have a good understanding of common spreadsheet authoring packages<br>» Have a good understanding of common document authoring packages<br>» Have a good understanding of common email authoring packages<br>» Have a good understanding of common instant messaging packages<br>» Have a good understanding of common video conferencing authoring packages

7.13 Have a command of technology	» Enable the use of technology suitable to the job held<br>» Understand the need of monitoring new developments and technologies in the sector<br>» Compare new technologies and recognising potential benefits for the sector<br>» Understand that resources and support are provided across the organisation to enable colleagues to make the best use of available technology<br>» Understand that the organisation has a strategy for technology use<br>» Compare how to minimise environmental damage through technology use<br>» Enable the use of technology to maximise effectiveness and efficiency<br>» Enable the use of field-based technology (e.g., radio, general packet radio service (GPRS), satellite phone)
Advanced Technology Competency(ies):	7.2 Blockchain<br>7.3 Unmanned Aerial Vehicles (UAVs)<br>7.7 Automation<br>7.9 Additive Manufacturing<br>7.11 Cloud Computing<br>7.14 eProcurement
7.2 Blockchain	» Examine blockchain as a value-add capability for relevant global health use cases
7.3 Unmanned Aerial Vehicles (UAVs)	» Understand the current UAV landscape, their application and the enabling environment required to support country implementation<br>» Understand UAVs as a value-add capability for relevant global health use cases<br>» Understand the laws/ regulations around the use of UAV’s
7.7 Automation	» Understand automation as a value-add capability for relevant global health use cases
7.9 Additive Manufacturing	» Understand additive manufacturing as a value-add capability for relevant global health use cases
7.11 Cloud Computing	» Understand cloud computing as a value-add capability for relevant global health use cases

7.14 eProcurement	» Understand how to transact with an eProcurement system where necessary<br>» Understand eProcurement as a value-add capability for relevant global health use cases<br>» Understand the benefits and limitations of eProcurement to best utilise the system

Key Performance Indicators:

- % of emergency orders issued in the last 12 months
- % of product selection based on National Essential Medicines List
- % of procured products that meet Stringent Regulatory Authority (SRA) or WHO standards
- Supply adequacy - Net requirements are reflected in the Procurement pipeline to secure sufficient future supply.
- Supply Plan report; supplier performance report
- Fill Rate vs Planned fill rate
- Identify, Prioritize, and Aggregate Supply Chain Requirements Cycle Time - Actual Time taken to determine net supply chain requirements by product and SKU vs Planned Time
- Order accuracy - Net requirement versus order quantities in the planning horizon
- Ratio between median price of pro- ducts procured and the international median reference value
- Availability - Stock out rate - Number of missed line item quantity deliveries due to stock out versus total line item quantity deliveries
- Availability - Stock levels - Current on hand stock versus Planned on hand stock
- Data accuracy - Vendor Master data accuracy, item / sku Master Data accuracy
- Demand analysis - Actual Demand versus Planned Demand
- Forecast Accuracy - Using various measures of Forecast Error e.g. MAD
- Inventory Days of Supply versus Planned Inventory days of Supply
- Reports submitted on time (for all and separate reports)

Training:	Qualifications Available:	Certifications Available:
» Contract Management<br>» Supplier Relationship Management (SRM)<br>» Analytical Skills<br>» Competitive Billing<br>» Negotiation<br>» Procurement/ RFQ Management		» CIPS Level 3 Advanced Certificate in Procurement and Supply Operations

Officer – Product Selection Use

Organisation: **SC-focused Organisation**				
Competency Level:	Serial Number:	Primary Process:	Job Role:	Supervises:
Operational	1-11-12-15-2	Plan – Procurement	Officer – Product Selection Use	-

Contributions	Responsible for the procurement of specific products, timeous communications of demand to suppliers, expediting
Technical Competency(ies):	1.1 Select the appropriate product<br>1.2 Define the specifications of the product, including product quality<br>1.5 Supply planning
1.1 Select the appropriate product	» Compare the broad concepts of the national medicines policy, essential medicines lists, essential equipment lists, standard treatment guidelines and "dangerous drug" (DDA) policy<br>» Understand the government system required to add and subtract items from the essential medicines list and the essential equipment list<br>» Understand that the government system required to alter standard treatment guidelines, dangerous drug policy and national medicines policy<br>» Compare the type of supplies and services that are required<br>» Provide input for regularly scheduled coordination meetings with stakeholders involved in financing, procuring or distributing commodities<br>» Understand the steps needed to bring a medicinal product to the market, including the safety, quality, efficacy and pharmacoeconomic assessments of the product
1.2 Define the specifications of the product, including product quality	» Understand specifications for procurement<br>» Compare the characteristics of a good specification<br>» Compare the types of specifications<br>» Name the advantages of functional and performance specifications<br>» Examine the contents of a specification<br>» Name the procurement staff responsibilities in the specification process<br>» Compare the quality standard of product
1.5 Supply planning	» Examine a purchasing and supply plan in line with national priorities<br>» Understand the importance of balancing of supply chain resources and trigger activities to correct any imbalances<br>» Understand the formalisation of sales and operations plans through sales and operations meeting.<br>» Understand that the sales and operations plan must be translated into executable plans to ensure dispatch of goods in line with overall priorities.<br>» Understand the national commodity pipeline

Personal/Management Competency(ies):	6.1 Demonstrate generic skills (e.g., literacy, numeracy, technology)<br>6.1.1 Exhibit high understanding of literacy and numeracy<br>6.2 Demonstrate strong communication skills<br>6.2.1 Practice cultural awareness
6.1 Demonstrate generic skills (e.g., literacy, numeracy, technology)	
6.1.1 Exhibit high understanding of literacy and numeracy	» Speak clearly and confidently, organize information in a logical manner, and consider voice tone and pace<br>» Respond to verbal messages and other cues (active listening)<br>» Express ideas and opinions clearly in written and verbal form<br>» Communicate information accurately, concisely and confidently in writing and verbally<br>» Practice a level of mathematics suitable to the job held<br>» Compare visual presentation techniques, including charting, histograms and flow sheets<br>» Read and comprehend at a level necessary to properly complete duties of the position<br>» Understand the need to interpret written information in documents, such as reports, SOPs, LMIS forms, graphs, calendars, schedules, notices and directions<br>» Understand the need to pay attention to detail and identify the main ideas, detect inconsistencies and identify missing information in documents<br>» Enable computer skills — such as using Word, Excel, PowerPoint and the internet — suitable to the job level<br>» Understand the process of analysing and validating KPIs<br>» Understand how the evaluation process of activities that act as standards against which the KPIs are measured is carried out<br>» Examine the process of performing critical self-review on work before submission
6.2 Demonstrate strong communication skills	» Be truthful and trustworthy, and supply accurate information at all times<br>» Enable working as part of a workplace team<br>» Understand vocabulary and communication style and form for both written and verbal communication that is appropriate for the situation, audience and material being communicated (e.g., avoid unnecessary jargon, clearly explain medical and SCM terminology)<br>» Enable open communication within constraints of confidentiality<br>» Participate in the organisation of communications about logistics<br>» Enable participation in meetings, expressing one's opinions, being aware of others' needs, and being appropriately assertive when required<br>» Show perspective into the point of view of others, understanding their needs and goals<br>» Enable the practise of building trust, rapport and credibility with others

- Understand the communication needs and concerns of others and respond to them
- Understand the need to avoid conflict between work and personal interests
- Enable meaningful two-way communication
- Show influence on others by persuasively presenting thoughts and ideas
- Show assertiveness skills to deal with unreasonable requests and/or refusals that would compromise practice or consumer care
- Maintain a positive, supportive and appreciative attitude
- Show the ability to balance the organisation's needs and the customer's needs
- Show self-control by maintaining composure and keeping emotions in check, even in difficult situations; deal calmly and effectively with stressful situations
- Enable expressing of opinions and providing information in written and/or verbal form in a manner that does not elicit concern, anger or other adverse response
- Enable the use of a systematic process for following up that demonstrates written reports have been received and understood
- Understand the means by which responses to input to the work environment are monitored
- Seek practical ways to overcome barriers to communication
- Understand the need to ensuring language representation (e.g., interpreters) at meetings when appropriate
- Interpret difficult situations and disputes between staff that need to be tackled and resolved
- Enable giving both positive and negative feedback sensitively
- Understand the need to maintain and improve communication
- Examine the process of giving and requesting feedback frequently
- Examine effective communication with shop stewards and union representatives

6.2.1 Practice cultural awareness

- Understand the need for cultural awareness and sensitivity, treating all people with fairness, respect and dignity
- Enable anti-discriminatory practices in the organisation, including HR and disciplinary procedures
- Enable cultural sensitivity, equality and fairness at all levels of the organisation
- Challenge discriminatory behaviour directly and sensitively
- Act in a non-discriminatory way toward individuals and groups
- Enable integrating cultural awareness in learning and development approaches
- Avoid stereotypical responses by examining one's own behaviour and bias
- Enable managing cultural diversity in teams and make the most of differences
- Compare strategies and/or resources for communicating effectively with people from different cultural backgrounds
- Enable the process to elicit information relating to values, beliefs and cultural backgrounds of consumers that may influence the way professional services are provided

Basal Technology Competency(ies):	7.6 ERP includes function of LMIS<br>7.12 Basic Office Skills<br>7.13 Have a command of technology
7.6 ERP includes function of LMIS	» Enable a Master Data Management System<br>» Understand the critical components of an ERP system<br>» Understand the meaning of common document types found within an ERP/LMIS<br>» Understand the logic of data visibility within an ERP<br>» Understand the importance of data integrity, record management and information security
7.12 Basic Office Skills	» Have a good understanding of common presentation authoring packages<br>» Have a good understanding of common spreadsheet authoring packages<br>» Have a good understanding of common document authoring packages<br>» Have a good understanding of common email authoring packages<br>» Have a good understanding of common instant messaging packages<br>» Have a good understanding of common video conferencing authoring packages
7.13 Have a command of technology	» Enable the use of technology suitable to the job held<br>» Understand the need of monitoring new developments and technologies in the sector<br>» Compare new technologies and recognising potential benefits for the sector<br>» Understand that resources and support are provided across the organisation to enable colleagues to make the best use of available technology<br>» Understand that the organisation has a strategy for technology use<br>» Compare how to minimise environmental damage through technology use<br>» Enable the use of technology to maximise effectiveness and efficiency<br>» Enable the use of field-based technology (e.g., radio, general packet radio service (GPRS), satellite phone)
Advanced Technology Competency(ies):	7.7 Automation
7.7 Automation	Understand automation as a value-add capability for relevant global health use cases

Key Performance Indicators:

- Forecast Accuracy
- Number of items whose replenishment is flagged past due date (Identify, Prioritize, and Aggregate Supply Chain Requirements (materials) within required Cycle Time) versus total item requirements.
- Inventory Days of Supply versus Planned Inventory as stipulated in the Operations Plan
- Fill Rate versus Planned Fill Rate
- Actual Procurement cycle time versus Planned Procurement Cycle Time
- Days Payable Outstanding due to incorrect Master Data, incorrect process steps, incorrect documentation, incorrect items supplied, incorrect items ordered
- Cost to Purchase versus Planned Cost to Purchase
- % Schedules Changed within Supplier's Lead Time
- Stock out rate due to failure in procurement
- Stock levels versus Planned Stock levels
- Ratio between median price of products procured and the international median reference value
- Order accuracy - Order accuracy measures how many orders were correctly picked and shipped / packed correctly to the customer/client (e.g. health facility or district store).
- Vendor on time delivery (VOTD)
- % of emergency orders issued in the last 12 months
- % of product selection based on National Essential Medicines List
- % of procured products that meet Stringent Regulatory Authority (SRA) or WHO standards
- Reports submitted on time (for all and separate reports) in accordance with business rules (reporting calendar)
- Data accuracy Number of Procurement Transaction failures due to data inaccuracy versus Total number of Procurement Transactions
- Supply adequacy - Net requirements are reflected in the Procurement pipeline to secure sufficient future supply.
- Supply Plan report; supplier performance report
- Stock out Rate - Number of items Actually stocked out or Expected to be stocked out based on inadequate Supply adequacy.
- Stock Levels - Actual stock levels versus Planned Stock Levels

Training:	Qualifications Available:	Certifications Available:
» Inventory Management<br>» Demand Management<br>» MPS Methodologies and Techniques<br>» S & OP Plan Communication<br>» Quantification of health products		» CIPS Level 3 Advanced Certificate in Procurement and Supply Operations<br>» SCM Principles On Demand Course on Basics of Inventory Management

Officer – Supply Planning/ Supplier Management

Organisation:

SC-focused Organisation

Competency Level:	Serial Number:	Primary Process:	Job Role:	Supervises:
Operational	1-15-22-13-2	Plan – Procurement	Officer – Supply Planning/ Supplier Management	-

Contributions	Manages the relationship between the Public Health System Organisation and its Suppliers. Measures their performance and liaises with them about performance targets
Technical Competency(ies):	1.2 Define the specifications of the product, including product quality<br>1.3 List any special considerations for the product (e.g., temperature requirements, size, implications for infrastructure)<br>1.4 Forecast and quantify product needs<br>1.5 Supply planning<br>2.2 Build and maintain supplier relationships
1.2 Define the specifications of the product, including product quality	» Understand specifications for procurement<br>» Compare the characteristics of a good specification<br>» Compare the types of specifications<br>» Name the advantages of functional and performance specifications<br>» Examine the contents of a specification<br>» Name the procurement staff responsibilities in the specification process<br>» Compare the quality standard of product<br>» Not Applicable
1.3 List any special considerations for the product (e.g., temperature requirements, size, implications for infrastructure)	» Compare the principles and processes of category management, including market segmentation principles<br>» Examine any specific considerations in the quantification of programme-specific products (e.g., ARVs, family planning commodities, vaccines)<br>» Examine current international trends in commodity availability<br>» Compare regulatory requirements for the product
1.4 Forecast and quantify product needs	» Examine the factors that affect usage patterns of medications and equipment and how this affects ordering (e.g., disease outbreaks), using national policies as a guide and to ensure consistent application<br>» Understand principles and applications of demand forecasting<br>» Compare the critical requirements for effective forecasting: establishing time horizons, level of detail and use of data<br>» Understand policies and procedures for forecasting<br>» Understand the process of quantifying product requirements using a variety of methods

- Examine consumption data at the national level for various country programmes
- Compare and reconcile different types of forecasts
- Understand the process of applying VEN or ABC analysis to programme requirements for national level procurement
- Understand the importance of calculating average monthly dispensed-to-user quantities for all service delivery points nationally
- Understand the importance of calculating storage space requirements for all levels in the supply chain
- Understand the importance of calculating the months of supply on hand for each commodity at the national level
- Examine the maximum and minimum stock levels for each level in a programme
- Understand various tools used in quantification (e.g., quantification software)
- Understand the use of various data sources for commodity forecasting (e.g., consumption data, services data, demographic data)
- Compare key performance indicators of forecast accuracy
- Examine the degree of error when using forecasting methods

1.5 Supply planning

- Examine a purchasing and supply plan in line with national priorities
- Understand the importance of balancing of supply chain resources and trigger activities to correct any imbalances
- Understand the formalisation of sales and operations plans through sales and operations meeting.
- Understand that the sales and operations plan must be translated into executable plans to ensure dispatch of goods in line with overall priorities.
- Understand the national commodity pipeline

2.2 Build and maintain supplier relationships

- Understand the factors in the analysis of the market
- Understand the processes for prequalification and tender contracting
- Understand the factors that define a good supplier
- Understand that key suppliers must be located and that there is a total cost associated with procuring an item or service
- Understand supplier relationship management plans (e.g., sourcing, frequency of meetings, negotiate and monitor benchmarks for performance)
- Understand the process of educating suppliers to create value for customers by streamlining processes in the value chain
- Understand the difference between strategic and non-strategic suppliers and the associated supplier management principles
- Understand commercial factors that contribute toward cost reduction, price savings and value improvement opportunities
- Understand the process of strategic sourcing practices drive down cost across consecutive tenders
- Compare the process of working with suppliers to agree cost saving opportunities in the whole value chain, with an appropriate cost sharing mechanism applied to ensure a “win-win” outcome
- Understand the process of managing supplier risk
- Understand ethical sourcing

Personal/Management Competency(ies):	6.1 Demonstrate generic skills (e.g., literacy, numeracy, technology)<br>6.1.1 Exhibit high understanding of literacy and numeracy<br>6.2 Demonstrate strong communication skills<br>6.2.1 Practice cultural awareness
**6.1 Demonstrate generic skills (e.g., literacy, numeracy, technology)**	
**6.1.1 Exhibit high understanding of literacy and numeracy**	» Speak clearly and confidently, organize information in a logical manner, and consider voice tone and pace<br>» Respond to verbal messages and other cues (active listening)<br>» Express ideas and opinions clearly in written and verbal form<br>» Communicate information accurately, concisely and confidently in writing and verbally<br>» Practice a level of mathematics suitable to the job held<br>» Compare visual presentation techniques, including charting, histograms and flow sheets<br>» Read and comprehend at a level necessary to properly complete duties of the position<br>» Understand the need to interpret written information in documents, such as reports, SOPs, LMIS forms, graphs, calendars, schedules, notices and directions<br>» Understand the need to pay attention to detail and identify the main ideas, detect inconsistencies and identify missing information in documents<br>» Enable computer skills — such as using Word, Excel, PowerPoint and the internet — suitable to the job level<br>» Understand the process of analysing and validating KPIs<br>» Understand how the evaluation process of activities that act as standards against which the KPIs are measured is carried out<br>» Examine the process of performing critical self-review on work before submission
**6.2 Demonstrate strong communication skills**	» Be truthful and trustworthy, and supply accurate information at all times<br>» Enable working as part of a workplace team<br>» Understand vocabulary and communication style and form for both written and verbal communication that is appropriate for the situation, audience and material being communicated (e.g., avoid unnecessary jargon, clearly explain medical and SCM terminology)<br>» Enable open communication within constraints of confidentiality<br>» Participate in the organisation of communications about logistics<br>» Enable participation in meetings, expressing one’s opinions, being aware of others’ needs, and being appropriately assertive when required

» Show perspective into the point of view of others, understanding their needs and goals

» Enable the practise of building trust, rapport and credibility with others

» Understand the communication needs and concerns of others and respond to them

» Understand the need to avoid conflict between work and personal interests

» Enable meaningful two-way communication

» Show influence on others by persuasively presenting thoughts and ideas

» Show assertiveness skills to deal with unreasonable requests and/or refusals that would compromise practice or consumer care

» Maintain a positive, supportive and appreciative attitude

» Show the ability to balance the organisation’s needs and the customer’s needs

» Show self-control by maintaining composure and keeping emotions in check, even in difficult situations; deal calmly and effectively with stressful situations

» Enable expressing of opinions and providing information in written and/or verbal form in a manner that does not elicit concern, anger or other adverse response

» Enable the use of a systematic process for following up that demonstrates written reports have been received and understood

» Understand the means by which responses to input to the work environment are monitored

» Seek practical ways to overcome barriers to communication

» Understand the need to ensuring language representation (e.g., interpreters) at meetings when appropriate

» Interpret difficult situations and disputes between staff that need to be tackled and resolved

» Enable giving both positive and negative feedback sensitively

» Understand the need to maintain and improve communication

» Examine the process of giving and requesting feedback frequently

» Examine effective communication with shop stewards and union representatives

6.2.1 Practice cultural awareness

» Understand the need for cultural awareness and sensitivity, treating all people with fairness, respect and dignity

» Enable anti-discriminatory practices in the organisation, including HR and disciplinary procedures

» Enable cultural sensitivity, equality and fairness at all levels of the organisation

» Challenge discriminatory behaviour directly and sensitively

» Act in a non-discriminatory way toward individuals and groups

» Enable integrating cultural awareness in learning and development approaches

» Avoid stereotypical responses by examining one’s own behaviour and bias

» Enable managing cultural diversity in teams and make the most of differences

» Compare strategies and/or resources for communicating effectively with people from different cultural backgrounds

	» Enable the process to elicit information relating to values, beliefs and cultural backgrounds of consumers that may influence the way professional services are provided
Basal Technology Competency(ies):	7.4 Temperature and monitoring<br>7.6 ERP includes function of LMIS<br>7.12 Basic Office Skills<br>7.13 Have a command of technology
7.4 Temperature and monitoring	» Compare the current technology approaches for Temperature monitoring and sensors<br>» Consider what is required to implement this technology in a country context<br>» Compare infrastructure requirement for heat-sensitive items at peripheral 'lower level' of SC depending on volume and nature of items to handle;<br>» Examine regular preventive maintenance and calibration of cold-chain equipment with biomedical engineers or logisticians<br>» Understand the policy for temperature monitoring tools during transportation and storage<br>» Understand the steps involved in health logistic management cycle: selection-order-delivery-reception-storage-distribution-inventory<br>» Understand hardware qualification and system validation of temperature monitoring<br>» Understand mass data management of temperature monitoring
7.6 ERP includes function of LMIS	» Enable a Master Data Management System<br>» Understand the critical components of an ERP system<br>» Understand the meaning of common document types found within an ERP/LMIS<br>» Understand the logic of data visibility within an ERP<br>» Understand the importance of data integrity, record management and information security
7.12 Basic Office Skills	» Have a good understanding of common presentation authoring packages<br>» Have a good understanding of common spreadsheet authoring packages<br>» Have a good understanding of common document authoring packages<br>» Have a good understanding of common email authoring packages<br>» Have a good understanding of common instant messaging packages<br>» Have a good understanding of common video conferencing authoring packages
7.13 Have a command of technology	» Enable the use of technology suitable to the job held<br>» Understand the need of monitoring new developments and technologies in the sector<br>» Compare new technologies and recognising potential benefits for the sector

	» Understand that resources and support are provided across the organisation to enable colleagues to make the best use of available technology<br>» Understand that the organisation has a strategy for technology use<br>» Compare how to minimise environmental damage through technology use<br>» Enable the use of technology to maximise effectiveness and efficiency<br>» Enable the use of field-based technology (e.g., radio, general packet radio service (GPRS), satellite phone)
Advanced Technology Competency(ies):	7.2 Blockchain<br>7.3 Unmanned Aerial Vehicles (UAVs)<br>7.7 Automation<br>7.9 Additive Manufacturing<br>7.11 Cloud Computing<br>7.14 eProcurement
7.2 Blockchain	» Examine blockchain as a value-add capability for relevant global health use cases
7.3 Unmanned Aerial Vehicles (UAVs)	» Understand the current UAV landscape, their application and the enabling environment required to support country implementation<br>» Understand UAVs as a value-add capability for relevant global health use cases<br>» Understand the laws/ regulations around the use of UAV’s
7.7 Automation	» Understand automation as a value-add capability for relevant global health use cases
7.9 Additive Manufacturing	» Understand additive manufacturing as a value-add capability for relevant global health use cases
7.11 Cloud Computing	» Understand cloud computing as a value-add capability for relevant global health use cases
7.14 eProcurement	» Understand how to transact with an eProcurement system where necessary<br>» Understand eProcurement as a value-add capability for relevant global health use cases<br>» Understand the benefits and limitations of eProcurement to best utilise the system

Key Performance Indicators:

- Vendor on time delivery (VOTD)
- % of emergency orders issued in the last 12 months
- % of product selection based on National Essential Medicines List
- % of procured products that meet Stringent Regulatory Authority (SRA) or WHO standards
- Supply adequacy - Net requirements are reflected in the Procurement pipeline to secure sufficient future supply.
- Supply Plan report; supplier performance report
- Fill Rate vs Planned fill rate
- Identify, Prioritize, and Aggregate Supply Chain Requirements Cycle Time - Actual Time taken to determine net supply chain requirements by product and SKU vs Planned Time
- Order accuracy - Net requirement versus order quantities in the planning horizon
- Ratio between median price of pro- ducts procured and the international median reference value
- Availability - Stock out rate - number of missed line item quantity deliveries due to stock out versus total line item quantity deliveries
- Availability - Stock levels - Current on hand stock versus Planned on hand stock
- Data accuracy - Vendor Master data accuracy, item / SKU Master Data accuracy
- Demand analysis - Actual Demand versus Planned Demand
- Forecast Accuracy - Using various measures of Forecast Error e.g. MAD
- Inventory Days of Supply versus Planned Inventory days of Supply
- Reports submitted on time (for all and separate reports)
- Availability of KPI Dashboards for Critical Supplier
- Percentage of Certified Suppliers vs Developing
- Supplier Awards Programme

Training:	Qualifications Available:	Certifications Available:
» Inventory Management<br>» Demand Management<br>» MPS Methodologies and Techniques<br>» S & OP Plan Communication<br>» Good Distribution Practice training		» CIPS Level 3 Advanced Certificate in Procurement and Supply Operations

Officer – Inventory and Demand Planning

Organisation:

SC-focused Organisation

Competency Level:	Serial Number:	Primary Process:	Job Role:	Supervises:
Operational	1-14-15-31-2	Plan – Procurement	Officer – Inventory and Demand Planning	-

Contributions	Regular monitoring of inventory levels and usage rates to calculate the procurement levels based on the Demand Plan.
Technical Competency(ies):	1.1 Select the appropriate product<br>1.5 Supply planning<br>2.4 Undertake contract management and risk<br>2.4.3 Addresses risk and ensures quality management<br>3.1 Undertake storage, warehousing and inventory management<br>3.1.1 Manage storage of commodities during emergency

1.1 Select the appropriate product

» Compare the broad concepts of the national medicines policy, essential medicines lists, essential equipment lists, standard treatment guidelines and "dangerous drug" (DDA) policy

» Understand the government system required to add and subtract items from the essential medicines list and the essential equipment list

» Understand that the government system required to alter standard treatment guidelines, dangerous drug policy and national medicines policy

» Compare the type of supplies and services that are required

» Provide input for regularly scheduled coordination meetings with stakeholders involved in financing, procuring or distributing commodities

» Understand the steps needed to bring a medicinal product to the market, including the safety, quality, efficacy and pharmacoeconomic assessments of the product

1.5 Supply planning

» Examine a purchasing and supply plan in line with national priorities

» Understand the importance of balancing of supply chain resources and trigger activities to correct any imbalances

» Understand the formalisation of sales and operations plans through sales and operations meeting.

» Understand that the sales and operations plan must be translated into executable plans to ensure dispatch of goods in line with overall priorities.

» Understand the national commodity pipeline

2.4 Undertake contract management and risk

2.4.3 Addresses risk and ensures quality management	» Understand the risk assessments for the procurement process (technical, commercial, administrative)<br>» Understand the key performance indicators for assessing procurement and supplier performance and effectiveness<br>» Examine any problems with the procurement of supplies in the supply chain<br>» Understand the contingency plan for shortages<br>» Compare recommended options for improving the performance of suppliers<br>» Understand procurement portfolio analysis and risk assessment (supply positioning)<br>» Understand the fundamentals of risk planning and assessment<br>»
3.1 Undertake storage, warehousing and inventory management	» Enable distribution of products among suppliers, distribution centres, warehouses and customers through a logistics network<br>» Enable a total systems approach to designing and managing the entire flow of information, materials, and services<br>» Understand the choice of equipment or materials based on suitability for intended use, accuracy, safety of use and cost<br>» Understand the importance of warehouses to have formal storage locations that identify the row, rack section, level and shelf location, typically with an alphanumeric location bar code or label<br>» Understand that medicines are to be stored appropriately, considering temperature, access and cleanliness<br>» Enable use of, and ability to maintain, the cold chain<br>» Understand the movement and storage of materials within a warehouse<br>» Understand the need to secure the medical store and limit access to staff<br>» Understand and follow organisation processes, instructions, rules and parameters for warehouse and inventory management and use of equipment (e.g., annual stock turns, expiry date tracking, stock procurement, rotation and retrieval, equipment maintenance)<br>» Enable and understand policies and procedures for stock handling, distribution and withdrawal consistent with maintaining safety (e.g., policies for distribution of concentrated electrolyte solutions and cytotoxics, identification of cytotoxic drug products within the work environment, separation of like-named or like-packaged products)<br>» Understand the information on the supplies being stored<br>» Enable the process of consolidating several items into larger units for fewer handlings<br>» Enable methods of stock rotation (e.g., first in first out, FIFO, or first to expire first out, FEFO)<br>» Understand the process of entering the quantity received and update the warehouse/inventory/LMIS system software with the stock on hand when each order is delivered according to requisite SOP's

	» Understand that there are required levels of stock and/or equipment in specific situations<br>» Understand the importance of accurate verification of rolling stocks<br>» Enable the process of monitoring the location and condition of the supplies being stored<br>» Understand the importance of the information on the storage locations and facilities<br>» Examine any problems in using the storage locations and facilities
3.1.1 Manage storage of commodities during emergency	» Understand the types of warehouses with those available for emergency or disaster supply<br>» Understanding the importance of the choice of storage site for emergency or disaster supply<br>» Understanding of the staff required for the storage of emergency or disaster supply<br>» Compare the equipment and material required for the warehousing of emergency or disaster supply<br>» Enable the conduct of a rapid assessment of logistical needs in emergencies
Personal/Management Competency(ies):	6.1 Demonstrate generic skills (e.g., literacy, numeracy, technology)<br>6.1.1 Exhibit high understanding of literacy and numeracy<br>6.2 Demonstrate strong communication skills<br>6.2.1 Practice cultural awareness
6.1 Demonstrate generic skills (e.g., literacy, numeracy, technology)	
6.1.1 Exhibit high understanding of literacy and numeracy	» Speak clearly and confidently, organize information in a logical manner, and consider voice tone and pace<br>» Respond to verbal messages and other cues (active listening)<br>» Express ideas and opinions clearly in written and verbal form<br>» Communicate information accurately, concisely and confidently in writing and verbally<br>» Practice a level of mathematics suitable to the job held<br>» Compare visual presentation techniques, including charting, histograms and flow sheets<br>» Read and comprehend at a level necessary to properly complete duties of the position<br>» Understand the need to interpret written information in documents, such as reports, SOPs, LMIS forms, graphs, calendars, schedules, notices and directions<br>» Understand the need to pay attention to detail and identify the main ideas, detect inconsistencies and identify missing information in documents

	» Enable computer skills — such as using Word, Excel, PowerPoint and the internet — suitable to the job level<br>» Understand the process of analysing and validating KPIs<br>» Understand how the evaluation process of activities that act as standards against which the KPIs are measured is carried out<br>» Examine the process of performing critical self-review on work before submission
6.2 Demonstrate strong communication skills	» Be truthful and trustworthy, and supply accurate information at all times<br>» Enable working as part of a workplace team<br>» Understand vocabulary and communication style and form for both written and verbal communication that is appropriate for the situation, audience and material being communicated (e.g., avoid unnecessary jargon, clearly explain medical and SCM terminology)<br>» Enable open communication within constraints of confidentiality<br>» Participate in the organisation of communications about logistics<br>» Enable participation in meetings, expressing one's opinions, being aware of others' needs, and being appropriately assertive when required<br>» Show perspective into the point of view of others, understanding their needs and goals<br>» Enable the practise of building trust, rapport and credibility with others<br>» Understand the communication needs and concerns of others and respond to them<br>» Understand the need to avoid conflict between work and personal interests<br>» Enable meaningful two-way communication<br>» Show influence on others by persuasively presenting thoughts and ideas<br>» Show assertiveness skills to deal with unreasonable requests and/or refusals that would compromise practice or consumer care<br>» Maintain a positive, supportive and appreciative attitude<br>» Show the ability to balance the organisation's needs and the customer's needs<br>» Show self-control by maintaining composure and keeping emotions in check, even in difficult situations; deal calmly and effectively with stressful situations<br>» Enable expressing of opinions and providing information in written and/or verbal form in a manner that does not elicit concern, anger or other adverse response<br>» Enable the use of a systematic process for following up that demonstrates written reports have been received and understood<br>» Understand the means by which responses to input to the work environment are monitored<br>» Seek practical ways to overcome barriers to communication<br>» Understand the need to ensuring language representation (e.g., interpreters) at meetings when appropriate

- Interpret difficult situations and disputes between staff that need to be tackled and resolved
- Enable giving both positive and negative feedback sensitively
- Understand the need to maintain and improve communication
- Examine the process of giving and requesting feedback frequently
- Examine effective communication with shop stewards and union representatives

6.2.1 Practice cultural awareness

- Understand the need for cultural awareness and sensitivity, treating all people with fairness, respect and dignity
- Enable anti-discriminatory practices in the organisation, including HR and disciplinary procedures
- Enable cultural sensitivity, equality and fairness at all levels of the organisation
- Challenge discriminatory behaviour directly and sensitively
- Act in a non-discriminatory way toward individuals and groups
- Enable integrating cultural awareness in learning and development approaches
- Avoid stereotypical responses by examining one's own behaviour and bias
- Enable managing cultural diversity in teams and make the most of differences
- Compare strategies and/or resources for communicating effectively with people from different cultural backgrounds
- Enable the process to elicit information relating to values, beliefs and cultural backgrounds of consumers that may influence the way professional services are provided

Key Performance Indicators:

- % of emergency orders issued in the last 12 months
- Supply adequacy - Net requirements are reflected in the Procurement pipeline to secure sufficient future supply.
- Fill Rate vs Planned fill rate
- Identify, Prioritize, and Aggregate Supply Chain Requirements Cycle Time - Actual Time taken to determine net supply chain requirements by product and SKU vs Planned Time
- Availability - Stock out rate - Number of missed line item quantity deliveries due to stock out versus total line item quantity deliveries
- Availability - Stock levels - Current on hand stock versus Planned on hand stock
- Data accuracy - Vendor Master data accuracy, item / sku Master Data accuracy
- Demand analysis - Actual Demand versus Planned Demand
- Forecast Accuracy - Using various measures of Forecast Error e.g. MAD
- Inventory Days of Supply versus Planned Inventory days of Supply
- Reports submitted on time (for all and separate reports)
- Warehouse Utilization - Volume used vs Volume Available

- Line Items SKU's handled vs Items / SKU's Planned over a planning horizon
- Level of expired stock
- Value of expired stock write-offs
- Number of warehouse locations used versus warehouse locations available (Pallet Positions may be used in larger warehouses)

Training:	Qualifications Available:	Certifications Available:
» Forecasting software training<br>» Inventory Management<br>» Demand Management<br>» MPS Methodologies and Techniques<br>» S & OP Plan Communication		» CIPS Level 3 Advanced Certificate in Procurement and Supply Operations

Officer – Network Analyst

Organisation:

SC-focused Organisation

Competency Level:	Serial Number:	Primary Process:	Job Role:	Supervises:
Operational	7-79-32-61-2	Enable – SC Networking	Officer – Network Analyst	-

Contributions	Calculation of network cost, gathering and analysis of network performance data
Technical Competency(ies):	3.2 Supply commodities to facilities

3.2 Supply commodities to facilities

- List the sources of distribution demand (customers)
- Understand the use of order policies for planned order generation
- Explain the calculation of re-supply quantities using a variety of inventory methods, including visual review, two bin, periodic review, order point and just in time
- Use order schedules
- Understand safety stock calculations
- Understand the principles of EOQ in the ordering process
- Understand the use of pull and push inventory control systems
- Enable a regular, rather than urgent, order culture
- Enable order-filling priorities in relation to delivery opportunities and urgency
- Understand that orders must be screened (modify order quantities on the basis of available stock, impact on service delivery, distance of facility from hospital)
- Enable assembling, checking, picking and packing of orders
- Understand making and using dispatch lists and invoices
- Enable the balancing of supply with demand, considering both lead time and demand variability created by supply patterns not matching demand patterns
- Understand information on the storage locations and facilities
- Understand measures of customer satisfaction by using performance metrics taken from the customer perspective, with criteria such as on-time delivery, perception of quality, complaints and length of wait times

Personal/Management Competency(ies):	6.1 Demonstrate generic skills (e.g., literacy, numeracy, technology)<br>6.1.1 Exhibit high understanding of literacy and numeracy<br>6.2 Demonstrate strong communication skills<br>6.2.1 Practice cultural awareness

6.1 Demonstrate generic skills (e.g., literacy, numeracy, technology)	
6.1.1 Exhibit high understanding of literacy and numeracy	» Speak clearly and confidently, organize information in a logical manner, and consider voice tone and pace<br>» Respond to verbal messages and other cues (active listening)<br>» Express ideas and opinions clearly in written and verbal form<br>» Communicate information accurately, concisely and confidently in writing and verbally<br>» Practice a level of mathematics suitable to the job held<br>» Compare visual presentation techniques, including charting, histograms and flow sheets<br>» Read and comprehend at a level necessary to properly complete duties of the position<br>» Understand the need to interpret written information in documents, such as reports, SOPs, LMIS forms, graphs, calendars, schedules, notices and directions<br>» Understand the need to pay attention to detail and identify the main ideas, detect inconsistencies and identify missing information in documents<br>» Enable computer skills — such as using Word, Excel, PowerPoint and the internet — suitable to the job level<br>» Understand the process of analysing and validating KPIs<br>» Understand how the evaluation process of activities that act as standards against which the KPIs are measured is carried out<br>» Examine the process of performing critical self-review on work before submission
6.2 Demonstrate strong communication skills	» Be truthful and trustworthy, and supply accurate information at all times<br>» Enable working as part of a workplace team<br>» Understand vocabulary and communication style and form for both written and verbal communication that is appropriate for the situation, audience and material being communicated (e.g., avoid unnecessary jargon, clearly explain medical and SCM terminology)<br>» Enable open communication within constraints of confidentiality<br>» Participate in the organisation of communications about logistics<br>» Enable participation in meetings, expressing one’s opinions, being aware of others’ needs, and being appropriately assertive when required<br>» Show perspective into the point of view of others, understanding their needs and goals<br>» Enable the practise of building trust, rapport and credibility with others<br>» Understand the communication needs and concerns of others and respond to them

	» Understand the need to avoid conflict between work and personal interests<br>» Enable meaningful two-way communication<br>» Show influence on others by persuasively presenting thoughts and ideas<br>» Show assertiveness skills to deal with unreasonable requests and/or refusals that would compromise practice or consumer care<br>» Maintain a positive, supportive and appreciative attitude<br>» Show the ability to balance the organisation's needs and the customer's needs<br>» Show self-control by maintaining composure and keeping emotions in check, even in difficult situations; deal calmly and effectively with stressful situations<br>» Enable expressing of opinions and providing information in written and/or verbal form in a manner that does not elicit concern, anger or other adverse response<br>» Enable the use of a systematic process for following up that demonstrates written reports have been received and understood<br>» Understand the means by which responses to input to the work environment are monitored<br>» Seek practical ways to overcome barriers to communication<br>» Understand the need to ensuring language representation (e.g., interpreters) at meetings when appropriate<br>» Interpret difficult situations and disputes between staff that need to be tackled and resolved<br>» Enable giving both positive and negative feedback sensitively<br>» Understand the need to maintain and improve communication<br>» Examine the process of giving and requesting feedback frequently<br>» Examine effective communication with shop stewards and union representatives
6.2.1 Practice cultural awareness	» Understand the need for cultural awareness and sensitivity, treating all people with fairness, respect and dignity<br>» Enable anti-discriminatory practices in the organisation, including HR and disciplinary procedures<br>» Enable cultural sensitivity, equality and fairness at all levels of the organisation<br>» Challenge discriminatory behaviour directly and sensitively<br>» Act in a non-discriminatory way toward individuals and groups<br>» Enable integrating cultural awareness in learning and development approaches<br>» Avoid stereotypical responses by examining one's own behaviour and bias<br>» Enable managing cultural diversity in teams and make the most of differences

- Compare strategies and/or resources for communicating effectively with people from different cultural backgrounds
- Enable the process to elicit information relating to values, beliefs and cultural backgrounds of consumers that may influence the way professional services are provided

Basal Technology Competency(ies):	7.13 Have a command of technology
7.13 Have a command of technology	» Enable the use of technology suitable to the job held<br>» Understand the need of monitoring new developments and technologies in the sector<br>» Compare new technologies and recognising potential benefits for the sector<br>» Understand that resources and support are provided across the organisation to enable colleagues to make the best use of available technology<br>» Understand that the organisation has a strategy for technology use<br>» Compare how to minimise environmental damage through technology use<br>» Enable the use of technology to maximise effectiveness and efficiency<br>» Enable the use of field-based technology (e.g., radio, general packet radio service (GPRS), satellite phone)

Key Performance Indicators:

- Assess Delivery Performance Cycle Time - Actual Time from assessed requirement to time delivered to end-user compared to committed Delivery Cycle Time as per Supply Chain strategy
- Assess Supplier Performance Cycle Time - Actual Supplier performance Cycle time versus Supplier commitment cycle time
- Inventory accuracy - Cycle count results
- Transportation lead time accuracy - Actual Transportation lead time versus Transportation promised lead time.
- Transport efficiency - Total cost of transport versus planned cost of transport by mode
- In-bound cost of acquisition - Actual total cost of acquisition versus Total Planned cost of acquisition including landed costs
- Manage performance cycle time
- Performance Report - Actual versus Plan reports for all metrics

Training:	Qualifications Available:	Certifications Available:
» Supply Chain Performance Measurements<br>» Outsourcing<br>» Prioritization<br>» Asset Management		» Lean Six Sigma Certification in Information Technology white belt

» Capacity Planning/Management<br>» Logistics network modelling<br>» Supply Chain Management		» Certified Supply Chain Analyst (CSCA-ISCEA)

Officer – Strategic Procurement

Organisation

SC-focused Organisation

Competency Level:	Serial Number:	Primary Process:	Job Role:	Supervises:
Operational	2-22-23-2	Enable – Procurement	Officer – Strategic Procurement	-

Contributions	Negotiation and evaluation of strategic items and suppliers. Certification of preferred, approved and available vendors. Determination and allocation of volumes to suppliers to maintain a fault tolerant network of suppliers.
Technical Competency(ies):	2.2 Build and maintain supplier relationships<br>2.3 Manage tendering processes and supplier agreements<br>2.4 Undertake contract management and risk<br>2.4.2 Manage contracts

2.2 Build and maintain supplier relationships

- Understand the factors in the analysis of the market
- Understand the processes for prequalification and tender contracting
- Understand the factors that define a good supplier
- Understand that key suppliers must be located and that there is a total cost associated with procuring an item or service
- Understand supplier relationship management plans (e.g., sourcing, frequency of meetings, negotiate and monitor benchmarks for performance)
- Understand the process of educating suppliers to create value for customers by streamlining processes in the value chain
- Understand the difference between strategic and non-strategic suppliers and the associated supplier management principles
- Understand commercial factors that contribute toward cost reduction, price savings and value improvement opportunities
- Understand the process of strategic sourcing practices drive down cost across consecutive tenders
- Compare the process of working with suppliers to agree cost saving opportunities in the whole value chain, with an appropriate cost sharing mechanism applied to ensure a “win-win” outcome
- Understand the process of managing supplier risk
- Understand ethical sourcing

2.3 Manage tendering processes and supplier agreements

- Understand the use of request for qualifications (RFQ), invitations to bid (ITB) and request for proposals (RFP) methods and when to choose which method
- Understand the local and international tendering procedures (e.g., open competitive bidding, restricted tender, competitive negotiation, direct procurement)
- Understand the detailed specifications for tenders

	» Understand the need for high-level guidance for high-value and politically sensitive procurements<br>» Understand the process of developing and managing contracts<br>» Understand the procurement, return and exchange policies<br>» Understand appropriate legislation and policy in regard to procurement processes, and what is required to comply<br>» Understand what constitutes ethical behaviour, abiding by conflict of interest policies<br>» Understand the process of evaluating tender bids based on the specification and evaluation criteria including capability of the supplier, problems relating to outcomes of the contract.<br>» Understand that there is a contract approval process, including contract negotiations<br>» Understand some of the key issues relevant to negotiating supply contracts (e.g., volume usage over time, price volume agreements, alternate supplier clauses, duration of contract, period of review, terms for contract termination and renewal)<br>» Understand the process of awarding and managing contracts, resolving any issues as they arise<br>» Understand the process of debriefing unsuccessful suppliers<br>» Understand the ways in which compliance with purchasing policies and procedures is monitored<br>» Understand the process of procurement planning including a yearly activities schedule and developing a wide range of briefs, specifications and commercial documentation<br>» Examine strategic sourcing projects, capturing benefits, work requirements, impacts and cost savings<br>» Provide input to cross-functional teams handling strategic procurements<br>» Compare internal and external procurement networks<br>» Compare local and international health commodity prices and understand the factors affecting them<br>» Understand the process of engaging clients across the public sector in presentations demonstrating the benefits of a range of strategic projects designed to ensure cost savings and value for money for government procurement
2.4 Undertake contract management and risk	»
2.4.2 Manage contracts	» Understand the process of conducting reviews of performance against agreed key performance indicators<br>» Understand the need for providing timely and expert guidance to remediate procurement performance issues<br>» Compare contract responsiveness and negotiating changes to a contract (contract variations)<br>» Understand the process of managing contract disputes and terminating contracts legally and appropriately if necessary

	» Understand the importance of supplier control mechanisms, contract administration and supplier management
Personal/Management Competency(ies):	6.1 Demonstrate generic skills (e.g., literacy, numeracy, technology)<br>6.1.1 Exhibit high understanding of literacy and numeracy<br>6.2 Demonstrate strong communication skills<br>6.2.1 Practice cultural awareness
**6.1 Demonstrate generic skills (e.g., literacy, numeracy, technology)**	
**6.1.1 Exhibit high understanding of literacy and numeracy**	» Speak clearly and confidently, organize information in a logical manner, and consider voice tone and pace<br>» Respond to verbal messages and other cues (active listening)<br>» Express ideas and opinions clearly in written and verbal form<br>» Communicate information accurately, concisely and confidently in writing and verbally<br>» Practice a level of mathematics suitable to the job held<br>» Compare visual presentation techniques, including charting, histograms and flow sheets<br>» Read and comprehend at a level necessary to properly complete duties of the position<br>» Understand the need to interpret written information in documents, such as reports, SOPs, LMIS forms, graphs, calendars, schedules, notices and directions<br>» Understand the need to pay attention to detail and identify the main ideas, detect inconsistencies and identify missing information in documents<br>» Enable computer skills — such as using Word, Excel, PowerPoint and the internet — suitable to the job level<br>» Understand the process of analysing and validating KPIs<br>» Understand how the evaluation process of activities that act as standards against which the KPIs are measured is carried out<br>» Examine the process of performing critical self-review on work before submission
**6.2 Demonstrate strong communication skills**	» Be truthful and trustworthy, and supply accurate information at all times<br>» Enable working as part of a workplace team<br>» Understand vocabulary and communication style and form for both written and verbal communication that is appropriate for the situation, audience and material being communicated (e.g., avoid unnecessary jargon, clearly explain medical and SCM terminology)<br>» Enable open communication within constraints of confidentiality<br>» Participate in the organisation of communications about logistics

- Enable participation in meetings, expressing one's opinions, being aware of others' needs, and being appropriately assertive when required
- Show perspective into the point of view of others, understanding their needs and goals
- Enable the practise of building trust, rapport and credibility with others
- Understand the communication needs and concerns of others and respond to them
- Understand the need to avoid conflict between work and personal interests
- Enable meaningful two-way communication
- Show influence on others by persuasively presenting thoughts and ideas
- Show assertiveness skills to deal with unreasonable requests and/or refusals that would compromise practice or consumer care
- Maintain a positive, supportive and appreciative attitude
- Show the ability to balance the organisation's needs and the customer's needs
- Show self-control by maintaining composure and keeping emotions in check, even in difficult situations; deal calmly and effectively with stressful situations
- Enable expressing of opinions and providing information in written and/or verbal form in a manner that does not elicit concern, anger or other adverse response
- Enable the use of a systematic process for following up that demonstrates written reports have been received and understood
- Understand the means by which responses to input to the work environment are monitored
- Seek practical ways to overcome barriers to communication
- Understand the need to ensuring language representation (e.g., interpreters) at meetings when appropriate
- Interpret difficult situations and disputes between staff that need to be tackled and resolved
- Enable giving both positive and negative feedback sensitively
- Understand the need to maintain and improve communication
- Examine the process of giving and requesting feedback frequently
- Examine effective communication with shop stewards and union representatives

6.2.1 Practice cultural awareness

- Understand the need for cultural awareness and sensitivity, treating all people with fairness, respect and dignity
- Enable anti-discriminatory practices in the organisation, including HR and disciplinary procedures
- Enable cultural sensitivity, equality and fairness at all levels of the organisation
- Challenge discriminatory behaviour directly and sensitively
- Act in a non-discriminatory way toward individuals and groups
- Enable integrating cultural awareness in learning and development approaches
- Avoid stereotypical responses by examining one's own behaviour and bias
- Enable managing cultural diversity in teams and make the most of differences

	» Compare strategies and/or resources for communicating effectively with people from different cultural backgrounds<br>» Enable the process to elicit information relating to values, beliefs and cultural backgrounds of consumers that may influence the way professional services are provided
Basal Technology Competency(ies):	7.4 Temperature and monitoring<br>7.6 ERP includes function of LMIS<br>7.12 Basic Office Skills<br>7.13 Have a command of technology
7.4 Temperature and monitoring	» Compare the current technology approaches for Temperature monitoring and sensors<br>» Consider what is required to implement this technology in a country context<br>» Compare infrastructure requirement for heat-sensitive items at peripheral 'lower level' of SC depending on volume and nature of items to handle;<br>» Examine regular preventive maintenance and calibration of cold-chain equipment with biomedical engineers or logisticians<br>» Understand the policy for temperature monitoring tools during transportation and storage<br>» Understand the steps involved in health logistic management cycle: selection-order-delivery-reception-storage-distribution-inventory<br>» Understand hardware qualification and system validation of temperature monitoring<br>» Understand mass data management of temperature monitoring
7.6 ERP includes function of LMIS	» Enable a Master Data Management System<br>» Understand the critical components of an ERP system<br>» Understand the meaning of common document types found within an ERP/LMIS<br>» Understand the logic of data visibility within an ERP<br>» Understand the importance of data integrity, record management and information security
7.12 Basic Office Skills	» Have a good understanding of common presentation authoring packages<br>» Have a good understanding of common spreadsheet authoring packages<br>» Have a good understanding of common document authoring packages<br>» Have a good understanding of common email authoring packages<br>» Have a good understanding of common instant messaging packages<br>» Have a good understanding of common video conferencing authoring packages

7.13 Have a command of technology	» Enable the use of technology suitable to the job held<br>» Understand the need of monitoring new developments and technologies in the sector<br>» Compare new technologies and recognising potential benefits for the sector<br>» Understand that resources and support are provided across the organisation to enable colleagues to make the best use of available technology<br>» Understand that the organisation has a strategy for technology use<br>» Compare how to minimise environmental damage through technology use<br>» Enable the use of technology to maximise effectiveness and efficiency<br>» Enable the use of field-based technology (e.g., radio, general packet radio service (GPRS), satellite phone)
Advanced Technology Competency(ies):	7.2 Blockchain<br>7.3 Unmanned Aerial Vehicles (UAVs)<br>7.7 Automation<br>7.9 Additive Manufacturing<br>7.11 Cloud Computing<br>7.14 eProcurement
7.2 Blockchain	» Examine blockchain as a value-add capability for relevant global health use cases
7.3 Unmanned Aerial Vehicles (UAVs)	» Understand the current UAV landscape, their application and the enabling environment required to support country implementation<br>» Understand UAVs as a value-add capability for relevant global health use cases<br>» Understand the laws/ regulations around the use of UAV's
7.7 Automation	» Understand automation as a value-add capability for relevant global health use cases
7.9 Additive Manufacturing	» Understand additive manufacturing as a value-add capability for relevant global health use cases
7.11 Cloud Computing	» Understand cloud computing as a value-add capability for relevant global health use cases
7.14 eProcurement	» Understand how to transact with an eProcurement system where necessary

» Understand eProcurement as a value-add capability for relevant global health use cases

» Understand the benefits and limitations of eProcurement to best utilise the system

Key Performance Indicators:

» Procurement cycle time

» Accuracy of tender documents

» Quality of Supplier Relationship

» Tenders adjudication and allocation on Time performance

» Tenders allocated in accordance with Business Rules

» Vendor/Supplier Master Data Accuracy

» Item Master Data Accuracy

» inbound Transportation Master Data Accuracy by Item SKU

» Item SKU Lead Time Accuracy

Training:	Qualifications Available:	Certifications Available:
» Contract Management<br>» Supplier Relationship Management (SRM)<br>» Analytical Skills<br>» Competitive Billing<br>» Negotiation<br>» Procurement/ RFQ Management		» CIPS Level 3 Advanced Certificate in Procurement and Supply Operations

Officer – Tender Management

Organisation:				
**SC-focused Organisation**				
Competency Level:	Serial Number:	Primary Process:	Job Role:	Supervises:
Operational	2-23-64-2	Enable – Procurement	Officer – Tender Management	-
Contributions	Preparation of Tender documents. Workflow of the tender documentation and process.			
Technical Competency(ies):	2.3 Manage tendering processes and supplier agreements			

2.3 Manage tendering processes and supplier agreements

- Understand the use of request for qualifications (RFQ), invitations to bid (ITB) and request for proposals (RFP) methods and when to choose which method
- Understand the local and international tendering procedures (e.g., open competitive bidding, restricted tender, competitive negotiation, direct procurement)
- Understand the detailed specifications for tenders
- Understand the need for high-level guidance for high-value and politically sensitive procurements
- Understand the process of developing and managing contracts
- Understand the procurement, return and exchange policies
- Understand appropriate legislation and policy in regard to procurement processes, and what is required to comply
- Understand what constitutes ethical behaviour, abiding by conflict of interest policies
- Understand the process of evaluating tender bids based on the specification and evaluation criteria including capability of the supplier, problems relating to outcomes of the contract.
- Understand that there is a contract approval process, including contract negotiations
- Understand some of the key issues relevant to negotiating supply contracts (e.g., volume usage over time, price volume agreements, alternate supplier clauses, duration of contract, period of review, terms for contract termination and renewal)
- Understand the process of awarding and managing contracts, resolving any issues as they arise
- Understand the process of debriefing unsuccessful suppliers
- Understand the ways in which compliance with purchasing policies and procedures is monitored
- Understand the process of procurement planning including a yearly activities schedule and developing a wide range of briefs, specifications and commercial documentation

	» Examine strategic sourcing projects, capturing benefits, work requirements, impacts and cost savings<br>» Provide input to cross-functional teams handling strategic procurements<br>» Compare internal and external procurement networks<br>» Compare local and international health commodity prices and understand the factors affecting them<br>» Understand the process of engaging clients across the public sector in presentations demonstrating the benefits of a range of strategic projects designed to ensure cost savings and value for money for government procurement
Personal/Management Competency(ies):	6.1 Demonstrate generic skills (e.g., literacy, numeracy, technology)<br>6.1.1 Exhibit high understanding of literacy and numeracy<br>6.2 Demonstrate strong communication skills<br>6.2.1 Practice cultural awareness<br>6.4 Exhibit professional and ethical values<br>6.4.1 Demonstrate integrity<br>6.4.2 Engage in continuous professional development
6.1 Demonstrate generic skills (e.g., literacy, numeracy, technology)	
6.1.1 Exhibit high understanding of literacy and numeracy	» Speak clearly and confidently, organize information in a logical manner, and consider voice tone and pace<br>» Respond to verbal messages and other cues (active listening)<br>» Express ideas and opinions clearly in written and verbal form<br>» Communicate information accurately, concisely and confidently in writing and verbally<br>» Practice a level of mathematics suitable to the job held<br>» Compare visual presentation techniques, including charting, histograms and flow sheets<br>» Read and comprehend at a level necessary to properly complete duties of the position<br>» Understand the need to interpret written information in documents, such as reports, SOPs, LMIS forms, graphs, calendars, schedules, notices and directions<br>» Understand the need to pay attention to detail and identify the main ideas, detect inconsistencies and identify missing information in documents<br>» Enable computer skills — such as using Word, Excel, PowerPoint and the internet — suitable to the job level<br>» Understand the process of analysing and validating KPIs<br>» Understand how the evaluation process of activities that act as standards against which the KPIs are measured is carried out<br>» Examine the process of performing critical self-review on work before submission

6.2 Demonstrate strong communication skills	» Be truthful and trustworthy, and supply accurate information at all times<br>» Enable working as part of a workplace team<br>» Understand vocabulary and communication style and form for both written and verbal communication that is appropriate for the situation, audience and material being communicated (e.g., avoid unnecessary jargon, clearly explain medical and SCM terminology)<br>» Enable open communication within constraints of confidentiality<br>» Participate in the organisation of communications about logistics<br>» Enable participation in meetings, expressing one's opinions, being aware of others' needs, and being appropriately assertive when required<br>» Show perspective into the point of view of others, understanding their needs and goals<br>» Enable the practise of building trust, rapport and credibility with others<br>» Understand the communication needs and concerns of others and respond to them<br>» Understand the need to avoid conflict between work and personal interests<br>» Enable meaningful two-way communication<br>» Show influence on others by persuasively presenting thoughts and ideas<br>» Show assertiveness skills to deal with unreasonable requests and/or refusals that would compromise practice or consumer care<br>» Maintain a positive, supportive and appreciative attitude<br>» Show the ability to balance the organisation's needs and the customer's needs<br>» Show self-control by maintaining composure and keeping emotions in check, even in difficult situations; deal calmly and effectively with stressful situations<br>» Enable expressing of opinions and providing information in written and/or verbal form in a manner that does not elicit concern, anger or other adverse response<br>» Enable the use of a systematic process for following up that demonstrates written reports have been received and understood<br>» Understand the means by which responses to input to the work environment are monitored<br>» Seek practical ways to overcome barriers to communication<br>» Understand the need to ensuring language representation (e.g., interpreters) at meetings when appropriate<br>» Interpret difficult situations and disputes between staff that need to be tackled and resolved<br>» Enable giving both positive and negative feedback sensitively<br>» Understand the need to maintain and improve communication<br>» Examine the process of giving and requesting feedback frequently<br>» Examine effective communication with shop stewards and union representatives

6.2.1 Practice cultural awareness	» Understand the need for cultural awareness and sensitivity, treating all people with fairness, respect and dignity<br>» Enable anti-discriminatory practices in the organisation, including HR and disciplinary procedures<br>» Enable cultural sensitivity, equality and fairness at all levels of the organisation<br>» Challenge discriminatory behaviour directly and sensitively<br>» Act in a non-discriminatory way toward individuals and groups<br>» Enable integrating cultural awareness in learning and development approaches<br>» Avoid stereotypical responses by examining one's own behaviour and bias<br>» Enable managing cultural diversity in teams and make the most of differences<br>» Compare strategies and/or resources for communicating effectively with people from different cultural backgrounds<br>» Enable the process to elicit information relating to values, beliefs and cultural backgrounds of consumers that may influence the way professional services are provided
**6.4 Exhibit professional and ethical values**	
6.4.1 Demonstrate integrity	» Accept responsibility for one's own work tasks and performance<br>» Enable working within a framework of clearly understood humanitarian values and ethics<br>» Stand by decisions and hold others accountable when necessary<br>» Does not abuse one's own power or position<br>» Enable the process of managing team members to ensure they do not abuse their power or position<br>» Examine when individuals or the organisation is straying from organisation goals, and challenge them to uphold ethics<br>» Understand the need to resist undue political pressure in decision making<br>» Understand the need to support staff in maintaining ethical stances<br>» Enable the process of making time in team for ethical inquiry and reflection<br>» Understand the need to show consistency between expressed principles and behaviour<br>» Understand that principles, values and ethics are embedded in policy<br>» Understand the need to act without consideration of personal gain<br>» Enable promoting transparency in decision making structures and processes<br>» Understand the need to ensure programmes are acting with integrity, and recognising the impact of not doing so

- Recognise one's own limitations and act upon them
- Follow all standard operating procedures
- Follow the practise of working in a safe and legal way
- Demonstrate respect, dignity and consideration for consumers
- Compare the impact of a no-blame culture on reporting and preventing recurrence of incidents
- Interpret requests of colleagues that might be regarded as unreasonable
- Understand the need to communicate (verbally and by example) expectations of the desired standards and approaches to be adopted
- Compare roles and responsibilities in terms of the position statement/duty statement of the position held
- Demonstrate punctuality
- Understand the obligation to apply professional care and expertise to deliver high quality of services
- Demonstrate care and attention to detail in undertaking work activities
- Demonstrate appropriate attire and presentation for the role and situation
- Recognise and take responsibility for emotions
- Enable an environment in which others can talk and act without fear of repercussion
- Seek to keep commitments and not let people down
- Be trusting and cooperative when working alongside others
- Take on various responsibilities within the department as the need arises

6.4.2 Engage in continuous professional development

- Seek out opportunities to learn, and integrate new knowledge and skills into work
- Examine gaps in knowledge and skills for completing specific tasks, and develop a plan to acquire them
- Listen to and invite feedback from others on one's own performance
- Use lifelong learning (continuous striving to gain knowledge and maintain competence) in the context of career development and the professional's role in delivering healthcare services
- Enable organisational systems for capturing learning and ensuring lessons learned
- Enable reflective learning within the team, in which lessons are captured and integrated into future projects
- Enable continuous learning as an integral part of organisational performance
- Be open to new ideas and different perspectives
- Keep up to date in the place of work with input from supervisors
- Follow and enable the practise of documenting continual professional development activities
- Enable the use of multiple techniques to acquire new knowledge and skills; process and retain information; and identify when it is necessary to acquire new knowledge and skills.

	» Attend professional organisations and/or committees<br>» Understand and/or enable quality improvement and/or quality assurance activities participated in<br>» Reflect on performance and seek what needs improvement<br>» Enable the process of engaging in personal career development, identifying occupational interests, strengths, options and opportunities<br>» Understand the need to contribute to others' professional development<br>» Enable sharing experiences and learning internally and externally<br>» Understand the need to seek out challenging projects outside of core experience and achieve solid results
Basal Technology Competency(ies):	7.6 ERP includes function of LMIS<br>7.12 Basic Office Skills<br>7.13 Have a command of technology
7.6 ERP includes function of LMIS	» Enable a Master Data Management System<br>» Understand the critical components of an ERP system<br>» Understand the meaning of common document types found within an ERP/LMIS<br>» Understand the logic of data visibility within an ERP<br>» Understand the importance of data integrity, record management and information security
7.12 Basic Office Skills	» Have a good understanding of common presentation authoring packages<br>» Have a good understanding of common spreadsheet authoring packages<br>» Have a good understanding of common document authoring packages<br>» Have a good understanding of common email authoring packages<br>» Have a good understanding of common instant messaging packages<br>» Have a good understanding of common video conferencing authoring packages
7.13 Have a command of technology	» Enable the use of technology suitable to the job held<br>» Understand the need of monitoring new developments and technologies in the sector<br>» Compare new technologies and recognising potential benefits for the sector<br>» Understand that resources and support are provided across the organisation to enable colleagues to make the best use of available technology<br>» Understand that the organisation has a strategy for technology use<br>» Compare how to minimise environmental damage through technology use<br>» Enable the use of technology to maximise effectiveness and efficiency

	» Enable the use of field-based technology (e.g., radio, general packet radio service (GPRS), satellite phone)
Advanced Technology Competency(ies):	7.2 Blockchain<br>7.7 Automation<br>7.9 Additive Manufacturing<br>7.11 Cloud Computing<br>7.14 eProcurement
7.2 Blockchain	» Examine blockchain as a value-add capability for relevant global health use cases
7.7 Automation	» Understand automation as a value-add capability for relevant global health use cases
7.9 Additive Manufacturing	» Understand additive manufacturing as a value-add capability for relevant global health use cases
7.11 Cloud Computing	» Understand cloud computing as a value-add capability for relevant global health use cases
7.14 eProcurement	» Understand how to transact with an eProcurement system where necessary<br>» Understand eProcurement as a value-add capability for relevant global health use cases<br>» Understand the benefits and limitations of eProcurement to best utilise the system

Key Performance Indicators:

- Procurement cycle time
- Accuracy of tender documents
- Quality of Supplier Relationship
- Tenders adjudication and allocation on Time performance
- Tenders allocated in accordance with Business Rules
- Vendor/Supplier Master Data Accuracy
- Item Master Data Accuracy
- inbound Transportation Master Data Accuracy by Item SKU
- Item SKU Lead Time Accuracy
- Tender creation cycle time
- On Time Closure of Tenders
- Risk avoidance, elimination and or Mitigation Costs

Training:	Qualifications Available:	Certifications Available:
» Contract Management<br>» Supplier Relationship Management (SRM)<br>» Analytical Skills<br>» Competitive Bidding<br>» Negotiation<br>» Procurement/ RFQ Management		» CIPS Level 3 Advanced Certificate in Procurement and Supply Operations

Officer – Contracts

Organisation: SC-focused Organisation				
Competency Level:	Serial Number:	Primary Process:	Job Role:	Supervises:
Operational	2-242-64-2	Enable – Contracts	Officer – Contracts	-
Contributions	Monitoring of awarded contracts for on-time delivery, cost and quality of supplies. Alerting strategic procurement when contracts are about to expire within adequate lead time to re-establish continuation of supply.			
Technical Competency(ies):	2.4 Undertake contract management and risk<br>2.4.1 Place commodity orders<br>2.4.2 Manage contracts<br>2.4.3 Addresses risk and ensures quality management			

2.4 Undertake contract management and risk

2.4.1 Place commodity orders

- » Understand that authorisations are required to administer the contracts
- » Understand the type of supplies required
- » Understand the process of obtaining reference sources and supplier catalogues to clarify required product and its availability
- » Understand the process to comply with policies and procedures to order required stock and equipment
- » Understand the process of confirming the orders with suppliers
- » Understand the process of identifying any problems in placing orders
- » Understand the procedures for placing orders
- » Examine any problems with order delivery and compare recommended options for progressing order delivery
- » Enable the execution of effective mechanisms for checking invoices exist and take action to adjust payments accordingly
- » Understand the process of receiving or raising requisitions and arranging purchase orders and payments

2.4.2 Manage contracts

- » Understand the process of conducting reviews of performance against agreed key performance indicators
- » Understand the need for providing timely and expert guidance to remediate procurement performance issues
- » Compare contract responsiveness and negotiating changes to a contract (contract variations)
- » Understand the process of managing contract disputes and terminating contracts legally and appropriately if necessary

	» Understand the importance of supplier control mechanisms, contract administration and supplier management
2.4.3 Addresses risk and ensures quality management	» Understand the risk assessments for the procurement process (technical, commercial, administrative)<br>» Understand the key performance indicators for assessing procurement and supplier performance and effectiveness<br>» Examine any problems with the procurement of supplies in the supply chain<br>» Understand the contingency plan for shortages<br>» Compare recommended options for improving the performance of suppliers<br>» Understand procurement portfolio analysis and risk assessment (supply positioning)<br>» Understand the fundamentals of risk planning and assessment
Personal/Management Competency(ies):	6.1 Demonstrate generic skills (e.g., literacy, numeracy, technology)<br>6.1.1 Exhibit high understanding of literacy and numeracy<br>6.2 Demonstrate strong communication skills<br>6.2.1 Practice cultural awareness<br>6.4 Exhibit professional and ethical values<br>6.4.1 Demonstrate integrity<br>6.4.2 Engage in continuous professional development
6.1 Demonstrate generic skills (e.g., literacy, numeracy, technology)	»
6.1.1 Exhibit high understanding of literacy and numeracy	» Speak clearly and confidently, organize information in a logical manner, and consider voice tone and pace<br>» Respond to verbal messages and other cues (active listening)<br>» Express ideas and opinions clearly in written and verbal form<br>» Communicate information accurately, concisely and confidently in writing and verbally<br>» Practice a level of mathematics suitable to the job held<br>» Compare visual presentation techniques, including charting, histograms and flow sheets<br>» Read and comprehend at a level necessary to properly complete duties of the position<br>» Understand the need to interpret written information in documents, such as reports, SOPs, LMIS forms, graphs, calendars, schedules, notices and directions<br>» Understand the need to pay attention to detail and identify the main ideas, detect inconsistencies and identify missing information in documents<br>» Enable computer skills — such as using Word, Excel, PowerPoint and the internet — suitable to the job level<br>» Understand the process of analysing and validating KPIs

	» Understand how the evaluation process of activities that act as standards against which the KPIs are measured is carried out<br>» Examine the process of performing critical self-review on work before submission
6.2 Demonstrate strong communication skills	» Be truthful and trustworthy, and supply accurate information at all times<br>» Enable working as part of a workplace team<br>» Understand vocabulary and communication style and form for both written and verbal communication that is appropriate for the situation, audience and material being communicated (e.g., avoid unnecessary jargon, clearly explain medical and SCM terminology)<br>» Enable open communication within constraints of confidentiality<br>» Participate in the organisation of communications about logistics<br>» Enable participation in meetings, expressing one's opinions, being aware of others' needs, and being appropriately assertive when required<br>» Show perspective into the point of view of others, understanding their needs and goals<br>» Enable the practise of building trust, rapport and credibility with others<br>» Understand the communication needs and concerns of others and respond to them<br>» Understand the need to avoid conflict between work and personal interests<br>» Enable meaningful two-way communication<br>» Show influence on others by persuasively presenting thoughts and ideas<br>» Show assertiveness skills to deal with unreasonable requests and/or refusals that would compromise practice or consumer care<br>» Maintain a positive, supportive and appreciative attitude<br>» Show the ability to balance the organisation's needs and the customer's needs<br>» Show self-control by maintaining composure and keeping emotions in check, even in difficult situations; deal calmly and effectively with stressful situations<br>» Enable expressing of opinions and providing information in written and/or verbal form in a manner that does not elicit concern, anger or other adverse response<br>» Enable the use of a systematic process for following up that demonstrates written reports have been received and understood<br>» Understand the means by which responses to input to the work environment are monitored<br>» Seek practical ways to overcome barriers to communication<br>» Understand the need to ensuring language representation (e.g., interpreters) at meetings when appropriate<br>» Interpret difficult situations and disputes between staff that need to be tackled and resolved<br>» Enable giving both positive and negative feedback sensitively<br>» Understand the need to maintain and improve communication<br>» Examine the process of giving and requesting feedback frequently

	» Examine effective communication with shop stewards and union representatives
6.2.1 Practice cultural awareness	» Understand the need for cultural awareness and sensitivity, treating all people with fairness, respect and dignity<br>» Enable anti-discriminatory practices in the organisation, including HR and disciplinary procedures<br>» Enable cultural sensitivity, equality and fairness at all levels of the organisation<br>» Challenge discriminatory behaviour directly and sensitively<br>» Act in a non-discriminatory way toward individuals and groups<br>» Enable integrating cultural awareness in learning and development approaches<br>» Avoid stereotypical responses by examining one's own behaviour and bias<br>» Enable managing cultural diversity in teams and make the most of differences<br>» Compare strategies and/or resources for communicating effectively with people from different cultural backgrounds<br>» Enable the process to elicit information relating to values, beliefs and cultural backgrounds of consumers that may influence the way professional services are provided
6.4 Exhibit professional and ethical values	
6.4.1 Demonstrate integrity	» Accept responsibility for one's own work tasks and performance<br>» Enable working within a framework of clearly understood humanitarian values and ethics<br>» Stand by decisions and hold others accountable when necessary<br>» Does not abuse one's own power or position<br>» Enable the process of managing team members to ensure they do not abuse their power or position<br>» Examine when individuals or the organisation is straying from organisation goals, and challenge them to uphold ethics<br>» Understand the need to resist undue political pressure in decision making<br>» Understand the need to support staff in maintaining ethical stances<br>» Enable the process of making time in team for ethical inquiry and reflection<br>» Understand the need to show consistency between expressed principles and behaviour<br>» Understand that principles, values and ethics are embedded in policy<br>» Understand the need to act without consideration of personal gain<br>» Enable promoting transparency in decision making structures and processes<br>» Understand the need to ensure programmes are acting with integrity, and recognising the impact of not doing so

	» Recognise one's own limitations and act upon them<br>» Follow all standard operating procedures<br>» Follow the practise of working in a safe and legal way<br>» Demonstrate respect, dignity and consideration for consumers<br>» Compare the impact of a no-blame culture on reporting and preventing recurrence of incidents<br>» Interpret requests of colleagues that might be regarded as unreasonable<br>» Understand the need to communicate (verbally and by example) expectations of the desired standards and approaches to be adopted<br>» Compare roles and responsibilities in terms of the position statement/duty statement of the position held<br>» Demonstrate punctuality<br>» Understand the obligation to apply professional care and expertise to deliver high quality of services<br>» Demonstrate care and attention to detail in undertaking work activities<br>» Demonstrate appropriate attire and presentation for the role and situation<br>» Recognise and take responsibility for emotions<br>» Enable an environment in which others can talk and act without fear of repercussion<br>» Seek to keep commitments and not let people down<br>» Be trusting and cooperative when working alongside others<br>» Take on various responsibilities within the department as the need arises
6.4.2 Engage in continuous professional development	» Seek out opportunities to learn, and integrate new knowledge and skills into work<br>» Examine gaps in knowledge and skills for completing specific tasks, and develop a plan to acquire them<br>» Listen to and invite feedback from others on one's own performance<br>» Use lifelong learning (continuous striving to gain knowledge and maintain competence) in the context of career development and the professional's role in delivering healthcare services<br>» Enable organisational systems for capturing learning and ensuring lessons learned<br>» Enable reflective learning within the team, in which lessons are captured and integrated into future projects<br>» Enable continuous learning as an integral part of organisational performance<br>» Be open to new ideas and different perspectives<br>» Keep up to date in the place of work with input from supervisors<br>» Follow and enable the practise of documenting continual professional development activities<br>» Enable the use of multiple techniques to acquire new knowledge and skills; process and retain information; and identify when it is necessary to acquire new knowledge and skills.<br>» Attend professional organisations and/or committees

- Understand and/or enable quality improvement and/or quality assurance activities participated in
- Reflect on performance and seek what needs improvement
- Enable the process of engaging in personal career development, identifying occupational interests, strengths, options and opportunities
- Understand the need to contribute to others' professional development
- Enable sharing experiences and learning internally and externally
- Understand the need to seek out challenging projects outside of core experience and achieve solid results

Basal Technology Competency(ies):	7.6 ERP includes function of LMIS<br>7.12 Basic Office Skills<br>7.13 Have a command of technology
7.6 ERP includes function of LMIS	» Enable a Master Data Management System<br>» Understand the critical components of an ERP system<br>» Understand the meaning of common document types found within an ERP/LMIS<br>» Understand the logic of data visibility within an ERP<br>» Understand the importance of data integrity, record management and information security
7.12 Basic Office Skills	» Have a good understanding of common presentation authoring packages<br>» Have a good understanding of common spreadsheet authoring packages<br>» Have a good understanding of common document authoring packages<br>» Have a good understanding of common email authoring packages<br>» Have a good understanding of common instant messaging packages<br>» Have a good understanding of common video conferencing authoring packages
7.13 Have a command of technology	» Enable the use of technology suitable to the job held<br>» Understand the need of monitoring new developments and technologies in the sector<br>» Compare new technologies and recognising potential benefits for the sector<br>» Understand that resources and support are provided across the organisation to enable colleagues to make the best use of available technology<br>» Understand that the organisation has a strategy for technology use<br>» Compare how to minimise environmental damage through technology use<br>» Enable the use of technology to maximise effectiveness and efficiency<br>» Enable the use of field-based technology (e.g., radio, general packet radio service (GPRS), satellite phone)

Advanced Technology Competency(ies):	7.2 Blockchain<br>7.11 Cloud Computing
7.2 Blockchain	» Examine blockchain as a value-add capability for relevant global health use cases
7.11 Cloud Computing	» Understand cloud computing as a value-add capability for relevant global health use cases

Key Performance Indicators:

- Procurement cycle time from requirement assessment to goods received in good order and condition according to specifications
- Tender creation cycle time - from needs assessment to Tender flotation
- Quality of Supplier Relationship - Number of miscommunications and misunderstandings between the SC Focused organisation and the suppliers in a given period of time. Measured by Number of shipments late or early, incorrect products and or sku's and incorrect quantity and or dosages
- Tenders adjudication and allocation on Time performance as per business rules. Number of times Tenders fail to be adjudicated in accordance with the business rules as a % of Total Tenders
- Tenders allocated in accordance with Business Rules Number of times Tenders fail to be allocated to suppliers in accordance with the business rules as a % of Total Tenders allocated.
- Number of elapsed days since Tenders that ought to be closed as per business rules and process fail to be closed versus Total tenders that ought to be closed.
- Procurement cycle time - Elapsed time from Tender award to contract award to goods received.
- Quality of Supplier Relationship - Number of misunderstandings regarding timing, products, sky's, quality, quantity, dosage and quantity.

Training:	Qualifications Available:	Certifications Available:
» Contract Management<br>» Supplier Relationship Management (SRM)<br>» Analytical Skills<br>» Competitive Billing<br>» Negotiation<br>» Procurement/ RFQ Management		» CIPS Level 3 Advanced Certificate in Procurement and Supply Operations

Officer – Data and Use

Organisation:

SC-focused Organisation

Competency Level:	Serial Number:	Primary Process:	Job Role:	Supervises:
Operational	7-71-55-76-2	Enable – Data Information	Officer – Data and Use	-

Contributions	Responsible for supporting IT architecture within the supply chain organisation. Including LMIS, accounting and supply chain management systems.
Personal/Management Competency(ies):	5.5 Manage and plan projects<br>5.5.1 Develop and direct project plans<br>5.5.2 Execute strategic decision making<br>5.5.3 Manage partnerships<br>5.5.4 Direct/participate in teamwork

5.5 Manage and plan projects

5.5.1 Develop and direct project plans

- » Understand the process to anticipate obstacles and critical events for ensuring the six rights of logistics management (right quantity, product, condition, place, time and cost) and the contingency plans to address them; as well as the need to monitor progress and take necessary corrective action when needed
- » Understand an operational plan that is consistent with the strategic plan, links specific goals and strategies, and identifies the performance indicators to be used to measure achievements
- » Enable the use of information gathering techniques from stakeholders, analysing situations and identifying implications to make correct decisions
- » Examine of the questions, “Where are we going?” and “How are we going to get there?” and the need to create a specific and purposeful path to achieve this.
- » Enable the process reference model to managing supply chain processes (i.e., integrating business process re-engineering, benchmarking and process measurement)
- » Understand the process of capturing the current state of a process and deriving the desired future state
- » Enable the process of selecting and applying tools or technological solutions to frequently encountered problems
- » Understand the need to provide staff with the necessary tools to carry out plans
- » Understand the need to monitor progress and make changes as required
- » Provide input at meetings for planning, organisation and monitoring logistics activities
- » Understand that the staff are accountable for achieving the desired results
- » Understand that planning, organising and managing resources bring about the successful completion of projects
- » Compare the key features of a successful change management strategy

5.5.2 Execute strategic decision making	» Understand that one can make decisions regarding one's own workload and area of responsibility<br>» Understand of need to prioritize competing tasks and perform them quickly and efficiently according to their importance; finding new ways of organizing or planning work to accomplish it more efficiently<br>» Understand the need to ensure proper time and space for consultation around decisions being made.<br>» Follow the process of considering the input of resources including staff needed<br>» Understand the need to check assumptions against facts<br>» Examine the key issues in a complex situation and come to the heart of the problem quickly<br>» Examine relevant information before making decisions<br>» Understand that strategic decisions are made in the interest of the organisation's goals<br>» Understand when to analyse, evaluate and execute tough decisions when necessary<br>» Understand the impact of decisions on others' work or team goals<br>» Understand the process to communicate decisions and ensure they are incorporated into policies and processes<br>» Understand inclusive and consultative strategic planning to establish strategic objectives<br>» Examine and enable emerging trends and practices<br>» Understand the macro and long-term consequences of decisions
5.5.3 Manage partnerships	» Enable the concepts of partnership working<br>» Understand the process of supporting implementation of partnership programmes<br>» Understand the process of experimenting with and trialling new ideas with partners<br>» Understand the need to build partnerships to deliver programme and increase impact<br>» Enable innovation and creativity in partnership work<br>» Understand the need to communicate key information with partner members<br>» Understand the need to involve and value partners in all aspects of programming<br>» Examine problems with supply chain relationships<br>» Understand the need to communicate effectively with nurses, doctors and other members of the healthcare team<br>» Understand the need to respond to consumer complaints or comments about services and/or advice received<br>» Understand the need for collaboration and consultation across the sector and encourage the team to work with others to gain market insight<br>» Understand the need to encourage input from other key actors in the humanitarian sector and develop relationships with a cross-section of actors

	» Understand the need to meet the reporting requirements of vertical programmes<br>» Understand the structure of the health system at a national level and explain this to others<br>» Compare the role of other members of the healthcare team (including with consumers) in a way that engenders understanding and confidence in the team and its members<br>» Understand the structure of the organisation, environment and/or service in which they work<br>» Understand how vertical programmes work within the health system<br>» Understand the communication network established to achieve work outcomes<br>» Understand the structure of the health system at the provincial/regional level and explain this to others<br>» Understand the roles and functions of government agencies in regulating and supporting supply chain organisations<br>» Compare knowledge of change management (e.g. PtD Theory of Change)
5.5.4 Direct/participate in teamwork	» Understand the need to work with team members within their area of authority to establish achievable goals and strategies that are consistent with the objectives established for the organisation as a whole<br>» Interpret an organisational chart that shows the lines of reporting and responsibility among staff<br>» Understand the need to actively contribute a perspective and making a positive contribution to team-based problem solving and decision making<br>» Understand the need to provide feedback, encouragement and support to team members for progressing strategic goals<br>» Understand the need to monitor team performance in relation to the organisation's mission and goals<br>» Understand the need for teams to think for themselves and resolve problems<br>» Understand the need to encourage and harness diversity within the team to boost team effectiveness<br>» Understand the need to encourage and support the team to work through its stages of development and perform well<br>» Understand roles and responsibilities in relation to employees' expertise and the expectations of collaborating team members<br>» Understand where their position fits in the structure (organisation, SC, healthcare sector) , the responsibilities their position entails and the accountabilities linked to the position
Basal Technology Competency(ies):	7.1 Data Science<br>7.6 ERP includes function of LMIS<br>7.12 Basic Office Skills<br>7.13 Have a command of technology

7.1 Data Science	» Understand end to end MIS technology components that are inherent in advanced MIS systems and explore how these may be relevant in different supply chain contexts<br>» Compare emerging technological approaches to data visualization and analysis<br>» Awareness of analytics (data interpretation and decision making)<br>» Enable the ability to interact, provide guidance for data (dashboard) consolidation in various levels and use the tech tools<br>» Support movement of data into electronic form at lowest/remote SC level<br>» Enable the ability to work with relational database
7.6 ERP includes function of LMIS	» Enable a Master Data Management System<br>» Understand the critical components of an ERP system<br>» Understand the meaning of common document types found within an ERP/LMIS<br>» Understand the logic of data visibility within an ERP<br>» Understand the importance of data integrity, record management and information security
7.12 Basic Office Skills	» Have a good understanding of common presentation authoring packages<br>» Have a good understanding of common spreadsheet authoring packages<br>» Have a good understanding of common document authoring packages<br>» Have a good understanding of common email authoring packages<br>» Have a good understanding of common instant messaging packages<br>» Have a good understanding of common video conferencing authoring packages
7.13 Have a command of technology	» Enable the use of technology suitable to the job held<br>» Understand the need of monitoring new developments and technologies in the sector<br>» Compare new technologies and recognising potential benefits for the sector<br>» Understand that resources and support are provided across the organisation to enable colleagues to make the best use of available technology<br>» Understand that the organisation has a strategy for technology use<br>» Compare how to minimise environmental damage through technology use<br>» Enable the use of technology to maximise effectiveness and efficiency<br>» Enable the use of field-based technology (e.g., radio, general packet radio service (GPRS), satellite phone)

Key Performance Indicators:

- Number of personnel with the required Skill, Education, Training and Aptitude as per Supply Chain Strategy versus Total number of personnel.
- Establishes and maintains IT infrastructure within system availability standards as specified in the IT Service Level Agreement e.g. Up time % over a period.
- Response - Number of closed Help desk requests over a period versus Help Desk Requests

- Effectiveness - Time to resolutions within Service Agreement Parameters. Number of Help Desk Request saved within the SLA versus total help desk requests
- Identification of root cause for help desk request. Classification of helpdesk requests into root causes of request. Maintain a remediation log for further actions to reduce the number of requests
- Propose remediation regarding systems, modules, training to enable personnel to support the Supply Chain performance requirements.
- Ensure security is enabled on all systems. Measure and report on security breaches.

Training:	Qualifications Available:	Certifications Available:
» IT infrastructure management<br>» Project Management<br>» Database management		» Advanced Certificate in Information Technology Governance<br>» Advanced Certificate in Information Technology in Support Services<br>» Lean Six Sigma Certification in Finance green belt

Officer – Data Management

Organisation:<br>**SC-focused Organisation**				
Competency Level:	Serial Number:	Primary Process:	Job Role:	Supervises:
Operational	7-71-76-2	Enable – Data Information	Officer – Data Management	-
Contributions	Responsible for supporting the Manager - Data use in providing access and data analysis tools for use across the supply chain organisation and creation of the data quality report			
Basal Technology Competency(ies):	7.1 Data Science<br>7.6 ERP includes function of LMIS<br>7.12 Basic Office Skills<br>7.13 Have a command of technology			

7.1 Data Science

- » Understand end to end MIS technology components that are inherent in advanced MIS systems and explore how these may be relevant in different supply chain contexts
- » Compare emerging technological approaches to data visualization and analysis
- » Awareness of analytics (data interpretation and decision making)
- » Enable the ability to interact, provide guidance for data (dashboard) consolidation in various levels and use the tech tools
- » Support movement of data into electronic form at lowest/remote SC level
- » Enable the ability to work with relational database

7.6 ERP includes function of LMIS

- » Enable a Master Data Management System
- » Understand the critical components of an ERP system
- » Understand the meaning of common document types found within an ERP/LMIS
- » Understand the logic of data visibility within an ERP
- » Understand the importance of data integrity, record management and information security

7.12 Basic Office Skills

- » Have a good understanding of common presentation authoring packages
- » Have a good understanding of common spreadsheet authoring packages
- » Have a good understanding of common document authoring packages
- » Have a good understanding of common email authoring packages
- » Have a good understanding of common instant messaging packages
- » Have a good understanding of common video conferencing authoring packages

7.13 Have a command of technology	» Enable the use of technology suitable to the job held<br>» Understand the need of monitoring new developments and technologies in the sector<br>» Compare new technologies and recognising potential benefits for the sector<br>» Understand that resources and support are provided across the organisation to enable colleagues to make the best use of available technology<br>» Understand that the organisation has a strategy for technology use<br>» Compare how to minimise environmental damage through technology use<br>» Enable the use of technology to maximise effectiveness and efficiency<br>» Enable the use of field-based technology (e.g., radio, general packet radio service (GPRS), satellite phone)

Key Performance Indicators:

- » Number of personnel with the required Skill, Education, Training and Aptitude as per Supply Chain Strategy versus Total number of personnel.
- » Number of personnel progressed professionally versus Number of personnel that were targeted for progress.
- » Establishes and maintains IT infrastructure within system availability standards as specified in the IT Service Level Agreement e.g. Up time % over a period.
- » Response - Number of closed Help desk requests over a period versus Help Desk Requests
- » Effectiveness - Time to resolutions within Service Agreement Parameters. Number of Help Desk Request saved within the SLA versus total help desk requests
- » Identification of root cause for help desk request. Classification of helpdesk requests into root causes of request. Maintain a remediation log for further actions to reduce the number of requests
- » Propose remediation regarding systems, modules, training to enable personnel to support the Supply Chain performance requirements.
- » Ensure security is enabled on all systems. Measure and report on security breaches.

Training:	Qualifications Available:	Certifications Available:
» Basics of supply chain management<br>» IT infrastructure management<br>» Database management		» Lean Six Sigma Certification in Information Technology white belt<br>» Further Education and Training Certificate: Business Systems Operations: End User (ERP)

Officer – QA/QC

Organisation:<br>**SC-focused Organisation**				
Competency Level:	Serial Number:	Primary Process:	Job Role:	Supervises:
Operational	2-243-25-28-2	Enable – Risk	Officer – QA/ QC	-

Contributions	Monitoring the feedback obtained from the operational data pertaining to the quality of products and or services. Alerting the correct personnel should a breach be sensed. Formulating mitigation actions or processes to eliminate or mitigate the risk from occurring
Technical Competency(ies):	2.4 Undertake contract management and risk<br>2.4.3 Addresses risk and ensures quality management<br>2.5 Ensure quality of products<br>2.8 Prepare for product supply during disasters and emergencies<br>2.9 Undertake or manage manufacturing or compounding of products

2.4 Undertake contract management and risk

2.4.3 Addresses risk and ensures quality management	» Understand the risk assessments for the procurement process (technical, commercial, administrative)<br>» Understand the key performance indicators for assessing procurement and supplier performance and effectiveness<br>» Examine any problems with the procurement of supplies in the supply chain<br>» Understand the contingency plan for shortages<br>» Compare recommended options for improving the performance of suppliers<br>» Understand procurement portfolio analysis and risk assessment (supply positioning)<br>» Understand the fundamentals of risk planning and assessment
2.5 Ensure quality of products	» Understand the process of using and monitoring for prequalification of suppliers<br>» Understand the process of ensuring that health commodities are not counterfeit and meet quality standards through certification (ISO, WHO GMP<br>» Understand the process of implementing, conducting and maintaining a reporting system of pharmacovigilance (e.g. report adverse drug reactions, quality defects, batch recall) and equipment faults<br>» Examine the process of inspecting products when delivered and during storage to catch defects or problems before they are given to, or needed by, clients<br>» Compare compliance with good distribution and good storage practices (temperature monitoring in the warehouse, transportation and distribution)

	» Understand product-specific shelf-life by which the product are acceptable or rejected (e.g. no less than 1 year for most of drugs, and less for short shelf-life like laboratory)
2.8 Prepare for product supply during disasters and emergencies	» Understand the need to communicate with the national disaster planning team<br>» Understand the procurement and logistic requirements for emergency and disaster supply<br>» Understand the importance of the assessment of local capacity before the emergency or disaster supply<br>» Understand the factors that may restrict emergency or disaster supply to take place
2.9 Undertake or manage manufacturing or compounding of products	» Understand the good manufacturing practice for pharmaceutical (GMP) medicine<br>» Compare compliance with relevant pharmaceutical policies and regulations
Personal/Management Competency(ies):	6.1 Demonstrate generic skills (e.g., literacy, numeracy, technology)<br>6.1.1 Exhibit high understanding of literacy and numeracy<br>6.2 Demonstrate strong communication skills<br>6.2.1 Practice cultural awareness
6.1 Demonstrate generic skills (e.g., literacy, numeracy, technology)	»
6.1.1 Exhibit high understanding of literacy and numeracy	» Speak clearly and confidently, organize information in a logical manner, and consider voice tone and pace<br>» Respond to verbal messages and other cues (active listening)<br>» Express ideas and opinions clearly in written and verbal form<br>» Communicate information accurately, concisely and confidently in writing and verbally<br>» Practice a level of mathematics suitable to the job held<br>» Compare visual presentation techniques, including charting, histograms and flow sheets<br>» Read and comprehend at a level necessary to properly complete duties of the position<br>» Understand the need to interpret written information in documents, such as reports, SOPs, LMIS forms, graphs, calendars, schedules, notices and directions<br>» Understand the need to pay attention to detail and identify the main ideas, detect inconsistencies and identify missing information in documents<br>» Enable computer skills — such as using Word, Excel, PowerPoint and the internet — suitable to the job level<br>» Understand the process of analysing and validating KPIs

	» Understand how the evaluation process of activities that act as standards against which the KPIs are measured is carried out<br>» Examine the process of performing critical self-review on work before submission
6.2 Demonstrate strong communication skills	» Be truthful and trustworthy, and supply accurate information at all times<br>» Enable working as part of a workplace team<br>» Understand vocabulary and communication style and form for both written and verbal communication that is appropriate for the situation, audience and material being communicated (e.g., avoid unnecessary jargon, clearly explain medical and SCM terminology)<br>» Enable open communication within constraints of confidentiality<br>» Participate in the organisation of communications about logistics<br>» Enable participation in meetings, expressing one's opinions, being aware of others' needs, and being appropriately assertive when required<br>» Show perspective into the point of view of others, understanding their needs and goals<br>» Enable the practise of building trust, rapport and credibility with others<br>» Understand the communication needs and concerns of others and respond to them<br>» Understand the need to avoid conflict between work and personal interests<br>» Enable meaningful two-way communication<br>» Show influence on others by persuasively presenting thoughts and ideas<br>» Show assertiveness skills to deal with unreasonable requests and/or refusals that would compromise practice or consumer care<br>» Maintain a positive, supportive and appreciative attitude<br>» Show the ability to balance the organisation's needs and the customer's needs<br>» Show self-control by maintaining composure and keeping emotions in check, even in difficult situations; deal calmly and effectively with stressful situations<br>» Enable expressing of opinions and providing information in written and/or verbal form in a manner that does not elicit concern, anger or other adverse response<br>» Enable the use of a systematic process for following up that demonstrates written reports have been received and understood<br>» Understand the means by which responses to input to the work environment are monitored<br>» Seek practical ways to overcome barriers to communication<br>» Understand the need to ensuring language representation (e.g., interpreters) at meetings when appropriate<br>» Interpret difficult situations and disputes between staff that need to be tackled and resolved<br>» Enable giving both positive and negative feedback sensitively<br>» Understand the need to maintain and improve communication

	» Examine the process of giving and requesting feedback frequently<br>» Examine effective communication with shop stewards and union representatives
6.2.1 Practice cultural awareness	» Understand the need for cultural awareness and sensitivity, treating all people with fairness, respect and dignity<br>» Enable anti-discriminatory practices in the organisation, including HR and disciplinary procedures<br>» Enable cultural sensitivity, equality and fairness at all levels of the organisation<br>» Challenge discriminatory behaviour directly and sensitively<br>» Act in a non-discriminatory way toward individuals and groups<br>» Enable integrating cultural awareness in learning and development approaches<br>» Avoid stereotypical responses by examining one's own behaviour and bias<br>» Enable managing cultural diversity in teams and make the most of differences<br>» Compare strategies and/or resources for communicating effectively with people from different cultural backgrounds<br>» Enable the process to elicit information relating to values, beliefs and cultural backgrounds of consumers that may influence the way professional services are provided
Basal Technology Competency(ies):	7.6 ERP includes function of LMIS<br>7.12 Basic Office Skills<br>7.13 Have a command of technology
7.6 ERP includes function of LMIS	» Enable a Master Data Management System<br>» Understand the critical components of an ERP system<br>» Understand the meaning of common document types found within an ERP/LMIS<br>» Understand the logic of data visibility within an ERP<br>» Understand the importance of data integrity, record management and information security
7.12 Basic Office Skills	» Have a good understanding of common presentation authoring packages<br>» Have a good understanding of common spreadsheet authoring packages<br>» Have a good understanding of common document authoring packages<br>» Have a good understanding of common email authoring packages<br>» Have a go& understanding of common instant messaging packages<br>» Have a good understanding of common video conferencing authoring packages
7.13 Have a command of technology	» Enable the use of technology suitable to the job held<br>» Understand the need of monitoring new developments and technologies in the sector

	» Compare new technologies and recognising potential benefits for the sector<br>» Understand that resources and support are provided across the organisation to enable colleagues to make the best use of available technology<br>» Understand that the organisation has a strategy for technology use<br>» Compare how to minimise environmental damage through technology use<br>» Enable the use of technology to maximise effectiveness and efficiency<br>» Enable the use of field-based technology (e.g., radio, general packet radio service (GPRS), satellite phone)
Advanced Technology Competency(ies):	7.2 Blockchain<br>7.11 Cloud Computing
7.2 Blockchain	» Examine blockchain as a value-add capability for relevant global health use cases
7.11 Cloud Computing	» Understand cloud computing as a value-add capability for relevant global health use cases

Key Performance Indicators:

- » Accuracy of Risk Register
- » Cycle time of QA/QC report
- » Overall value at risk
- » Partner/Donor integration, policy, guidelines and regulatory reforms
- » QA/QC report
- » Risk / Mitigation Costs
- » Time to Recovery (TTR)

Training:	Qualifications Available:	Certifications Available:
» Risk and exception management<br>» Process Auditing<br>» Basics of Supply Chain Management		» CIPS Level 3 Advanced Certificate in Procurement and Supply Operations<br>» APICS Certified in production and inventory management certificate<br>» Further Education and Training Certificate: Archives and Records Management<br>» Further Education and Training Certificate: Production Technology<br>» Lean Six Sigma Certification in Human Resources white belt<br>» Lean Six Sigma Certification in Information Technology white belt<br>» Manage Logistics Operations skills programme<br>» Apply fundamental concepts and principles of supply chain management skills programme

Officer – Import and Clearance

Organisation:

SC-focused Organisation

Competency Level:	Serial Number:	Primary Process:	Job Role:	Supervises:
Operational	2-26-61-3	Procure	Officer – Import and Clearance	-

Contributions	Be knowledgeable of Customs and Excise regulations, be knowledgeable of INCO Terms, their application and consequences. Have a sound relationship with Clearing Houses and Custom and Excise authorities. Expedite inbound and outbound shipments to support the Supply Chain Strategy.
Technical Competency(ies):	2.6 Manage import and export of products

2.6 Manage import and export of products

- » Comply with import, export and customs procedures and requirements
- » Identify import or export requirements for the supplies
- » Describe import or export documentation for the supplies
- » Describe the completion of import and export procedures
- » Identify any problems with the import and export procedures and requirements
- » Apply knowledge about international business processes (e.g. shipping, air cargo, clearing and forwarding, financial transactions, business ethics)

Personal/Management Competency(ies):	6.1 Demonstrate generic skills (e.g., literacy, numeracy, technology)<br>6.1.1 Exhibit high understanding of literacy and numeracy<br>6.2 Demonstrate strong communication skills<br>6.2.1 Practice cultural awareness

6.1 Demonstrate generic skills (e.g., literacy, numeracy, technology)

6.1.1 Exhibit high understanding of literacy and numeracy

- » Speak clearly and confidently, organize information in a logical manner, and consider voice tone and pace
- » Respond to verbal messages and other cues (active listening)
- » Express ideas and opinions clearly in written and verbal form
- » Communicate information accurately, concisely and confidently in writing and verbally
- » Practice a level of mathematics suitable to the job held
- » Describe visual presentation techniques, including charting, histograms and flow sheets

» Read and comprehend at a level necessary to properly complete duties of the position

» Interpret written information in documents, such as reports, SOPs, LMIS forms, graphs, calendars, schedules, notices and directions

» Pay attention to detail and identify the main ideas, detect inconsistencies and identify missing information in documents

» Apply computer skills — such as using Word, Excel, PowerPoint and the internet — suitable to the job level

» Describe the process of analysing and validating KPIs

» Participate in the evaluation of activities that act as standards against which the KPIs are measured

» Apply the process of performing critical self-review on work before submission

6.2 Demonstrate strong communication skills

» Be truthful and trustworthy, and supply accurate information at all times

» Work as part of a workplace team

» Describe vocabulary and communication style and form for both written and verbal communication that is appropriate for the situation, audience and material being communicated (e.g., avoid unnecessary jargon, clearly explain medical and SCM terminology)

» Encourage open communication within constraints of confidentiality

» Organize communications about logistics

» Encourage participation in meetings, expressing one's opinions, being aware of others' needs, and being appropriately assertive when required

» Apply perspective into the point of view of others, understanding their needs and goals

» Encourage building trust, rapport and credibility with others

» Anticipate the communication needs and concerns of others and respond to them

» Avoid conflict between work and personal interests

» Encourage meaningful two-way communication

» Influence others by persuasively presenting thoughts and ideas

» Apply assertiveness skills to deal with unreasonable requests and/or refusals that would compromise practice or consumer care

» Maintain a positive, supportive and appreciative attitude

» Apply the ability to balance the organisation's needs and the customer's needs

» Use self-control by maintaining composure and keeping emotions in check, even in difficult situations; deal calmly and effectively with stressful situations

» Encourage the expressing of opinions and providing information in written and/or verbal form in a manner that does not elicit concern, anger or other adverse response

» Apply the use of a systematic process for following up that demonstrates written reports have been received and understood

	» Describe the means by which responses to input to the work environment are monitored<br>» Seek practical ways to overcome barriers to communication<br>» Identify language representation (e.g., interpreters) at meetings when appropriate<br>» Tackle difficult situations and resolve disputes between staff<br>» Give both positive and negative feedback sensitively<br>» Maintain and improve communication<br>» Apply the process of giving and requesting feedback frequently<br>» Apply the ability to communicate effectively with shop stewards and union representatives
6.2.1 Practice cultural awareness	» Describe cultural awareness and sensitivity, treating all people with fairness, respect and dignity<br>» Describe anti-discriminatory practices in the organisation, including HR and disciplinary procedures<br>» Establish cultural sensitivity, equality and fairness at all levels of the organisation<br>» Challenge discriminatory behaviour directly and sensitively<br>» Act in a non-discriminatory way toward individuals and groups<br>» Integrate cultural awareness in learning and development approaches<br>» Avoid stereotypical responses by examining one's own behaviour and bias<br>» Manage cultural diversity in teams and make the most of differences<br>» Describe strategies and/or resources for communicating effectively with people from different cultural backgrounds<br>» Elicit information relating to values, beliefs and cultural backgrounds of consumers that may influence the way professional services are provided
Basal Technology Competency(ies):	7.6 ERP includes function of LMIS<br>7.12 Basic Office Skills<br>7.13 Have a command of technology
7.6 ERP includes function of LMIS	» Support a Master Data Management System<br>» Describe the critical components of an ERP system<br>» Describe the meaning of common document types found within an ERP/LMIS<br>» Describe the logic of data visibility within an ERP<br>» Describe the importance of data integrity, record management and information security
7.12 Basic Office Skills	» Have a good understanding of common presentation authoring packages<br>» Have a good understanding of common spreadsheet authoring packages<br>» Have a good understanding of common document authoring packages

	» Have a good understanding of common email authoring packages<br>» Have a good understanding of common instant messaging packages<br>» Have a good understanding of common video conferencing authoring packages
7.13 Have a command of technology	» Apply the use of technology suitable to the job held<br>» Provide input on monitoring new developments and technologies in the sector<br>» Identify new technologies and recognising potential benefits for the sector<br>» Identify resources and support that are provided across the organisation to enable colleagues to make the best use of available technology<br>» Ensure that the organisation has a strategy for technology use<br>» Describe how to minimise environmental damage through technology use<br>» Use technology to maximise effectiveness and efficiency<br>» Use field-based technology (e.g., radio, general packet radio service (GPRS), satellite phone)
Advanced Technology Competency(ies):	7.2 Blockchain<br>7.11 Cloud Computing
7.2 Blockchain	» Describe blockchain as a value-add capability for relevant global health use cases
7.11 Cloud Computing	» Describe cloud computing as a value-add capability for relevant global health use cases

Key Performance Indicators:

» Accuracy of Risk Register
» Cycle time of QA/QC report
» Overall value at risk
» Partner/Donor integration, policy, guidelines and regulatory reforms
» QA/QC report
» Risk / Mitigation Costs
» Time to Recovery (TTR)
» Number of Shipments Cleared first Time versus total Shipments cleared
» Number of Procurement instructions with invalid Uniform Custom codes vs Number of Procurement documents
» Number of Procurement instructions with invalid Inco Terms vs Number of Procurement documents
» Number of Procurement instructions with invalid custom tariffs versus Number of Procurement Documents

Training:	Qualifications Available:	Certifications Available:

» Procurement<br>» Controls and Compliance<br>» Import/Export Regulations<br>» Logistics & Distribution training	» Diploma in International Trade Management in Exports/Imports<br>» Diploma in Export Management	» Higher Certificate in Export Management<br>» Manage Logistics Operations skills programme<br>» Apply fundamental concepts and principles of supply chain management skills programme<br>» APICS Certified in Logistics, Transportation and Distribution (CLTD)

Officer – Receiving

Organisation:<br>**SC-focused Organisation**				
Competency Level:<br>**Operational**	Serial Number:<br>**3-31-311-34-2**	Primary Process:<br>**Procure**	Job Role:<br>**Officer – Receiving**	Supervises:<br>**Assistant – Receiving**
Contributions	Have knowledge of receiving protocols and process. Establish a workflow to ensure speedy and accurate receipt of products. Maintain a staff complement that understands and is comfortable with the execution of the workflow.			
Technical Competency(ies):	3.1 Undertake storage, warehousing and inventory management<br>3.1.1 Manage storage of commodities during emergency<br>3.4 Manage transport for commodities<br>3.4.1 Manage transport for commodities during disaster			

3.1 Undertake storage, warehousing and inventory management

» Enable distribution of products among suppliers, distribution centres, warehouses and customers through a logistics network

» Enable a total systems approach to designing and managing the entire flow of information, materials, and services

» Understand the choice of equipment or materials based on suitability for intended use, accuracy, safety of use and cost

» Understand the importance of warehouses to have formal storage locations that identify the row, rack section, level and shelf location, typically with an alphanumeric location bar code or label

» Understand that medicines are to be stored appropriately, considering temperature, access and cleanliness

» Enable use of, and ability to maintain, the cold chain

» Understand the movement and storage of materials within a warehouse

» Understand the need to secure the medical store and limit access to staff

» Understand and follow organisation processes, instructions, rules and parameters for warehouse and inventory management and use of equipment (e.g., annual stock turns, expiry date tracking, stock procurement, rotation and retrieval, equipment maintenance)

» Enable and understand policies and procedures for stock handling, distribution and withdrawal consistent with maintaining safety (e.g., policies for distribution of concentrated electrolyte solutions and cytotoxics, identification of cytotoxic drug products within the work environment, separation of like-named or like-packaged products)

» Understand the information on the supplies being stored

» Enable the process of consolidating several items into larger units for fewer handlings

	» Enable methods of stock rotation (e.g., first in first out, FIFO, or first to expire first out, FEFO)<br>» Understand the process of entering the quantity received and update the warehouse/inventory/LMIS system software with the stock on hand when each order is delivered according to requisite SOP's<br>» Understand that there are required levels of stock and/or equipment in specific situations<br>» Understand the importance of accurate verification of rolling stocks<br>» Enable the process of monitoring the location and condition of the supplies being stored<br>» Understand the importance of the information on the storage locations and facilities<br>» Examine any problems in using the storage locations and facilities
3.1.1 Manage storage of commodities during emergency	» Understand the types of warehouses with those available for emergency or disaster supply<br>» Understanding the importance of the choice of storage site for emergency or disaster supply<br>» Understanding of the staff required for the storage of emergency or disaster supply<br>» Compare the equipment and material required for the warehousing of emergency or disaster supply<br>» Enable the conduct of a rapid assessment of logistical needs in emergencies
3.4 Manage transport for commodities	» Understand distribution activities, including a fleet of vehicles and distribution schedules, to deliver health commodities to facilities.<br>» Understand the policies and procedures related to handling and transporting special products such as vaccines, HIV/AIDS drugs, other high value products and narcotics<br>» Understands shipping methods, considering trade-offs between costs and benefits<br>» Understand local and national laws/requirements for vehicle safety, driving regulations and licensing<br>» Understand proper packing and labelling methods for transportation of hazardous materials<br>» Understand the process of maximizing freight loads while minimizing freight costs<br>» Enable efficient use of transportation resources while meeting customers' needs<br>» Examine movement demands with vehicle resources<br>» Understand quantitative techniques when solving logistics problems, such as designing routes and scheduling vehicles<br>» Understand the process of delivering products to hospitals and district health offices with the accompanying dispatch note and report and requisition forms<br>» Coordinate transport options for order delivery<br>» Understand that vehicle availability is due to a vehicle maintenance plan

	» Interpret information on distribution requirements<br>» Examine any problems in distribution requirements<br>» Enable the process of equipping distribution sites<br>» Understand the need to inform stakeholders for better coordination of distribution<br>» Compare tracking devices (tools and indicators)<br>» Examine the process of training of drivers, van assistants, fleet administrators and expeditors<br>» Examine the process of supporting investigations around missed OTIF and other failures
3.4.1 Manage transport for commodities during disaster	» Be able to compare of the different types of transport and their characteristics for emergency or disaster supply<br>» Understand the process of developing a plan for the achievement of a rapid response logistics plan for deployment in the event of a medical emergency.
Personal/Management Competency(ies):	6.1 Demonstrate generic skills (e.g., literacy, numeracy, technology)<br>6.1.1 Exhibit high understanding of literacy and numeracy<br>6.2 Demonstrate strong communication skills<br>6.2.1 Practice cultural awareness
6.1 Demonstrate generic skills (e.g., literacy, numeracy, technology)	
6.1.1 Exhibit high understanding of literacy and numeracy	» Speak clearly and confidently, organize information in a logical manner, and consider voice tone and pace<br>» Respond to verbal messages and other cues (active listening)<br>» Express ideas and opinions clearly in written and verbal form<br>» Communicate information accurately, concisely and confidently in writing and verbally<br>» Practice a level of mathematics suitable to the job held<br>» Compare visual presentation techniques, including charting, histograms and flow sheets<br>» Read and comprehend at a level necessary to properly complete duties of the position<br>» Understand the need to interpret written information in documents, such as reports, SOPs, LMIS forms, graphs, calendars, schedules, notices and directions<br>» Understand the need to pay attention to detail and identify the main ideas, detect inconsistencies and identify missing information in documents<br>» Enable computer skills — such as using Word, Excel, PowerPoint and the internet — suitable to the job level<br>» Understand the process of analysing and validating KPIs<br>» Understand how the evaluation process of activities that act as standards against which the KPIs are measured is carried out

	» Examine the process of performing critical self-review on work before submission
6.2 Demonstrate strong communication skills	» Be truthful and trustworthy, and supply accurate information at all times<br>» Enable working as part of a workplace team<br>» Understand vocabulary and communication style and form for both written and verbal communication that is appropriate for the situation, audience and material being communicated (e.g., avoid unnecessary jargon, clearly explain medical and SCM terminology)<br>» Enable open communication within constraints of confidentiality<br>» Participate in the organisation of communications about logistics<br>» Enable participation in meetings, expressing one's opinions, being aware of others' needs, and being appropriately assertive when required<br>» Show perspective into the point of view of others, understanding their needs and goals<br>» Enable the practise of building trust, rapport and credibility with others<br>» Understand the communication needs and concerns of others and respond to them<br>» Understand the need to avoid conflict between work and personal interests<br>» Enable meaningful two-way communication<br>» Show influence on others by persuasively presenting thoughts and ideas<br>» Show assertiveness skills to deal with unreasonable requests and/or refusals that would compromise practice or consumer care<br>» Maintain a positive, supportive and appreciative attitude<br>» Show the ability to balance the organisation's needs and the customer's needs<br>» Show self-control by maintaining composure and keeping emotions in check, even in difficult situations; deal calmly and effectively with stressful situations<br>» Enable expressing of opinions and providing information in written and/or verbal form in a manner that does not elicit concern, anger or other adverse response<br>» Enable the use of a systematic process for following up that demonstrates written reports have been received and understood<br>» Understand the means by which responses to input to the work environment are monitored<br>» Seek practical ways to overcome barriers to communication<br>» Understand the need to ensuring language representation (e.g., interpreters) at meetings when appropriate<br>» Interpret difficult situations and disputes between staff that need to be tackled and resolved<br>» Enable giving both positive and negative feedback sensitively<br>» Understand the need to maintain and improve communication<br>» Examine the process of giving and requesting feedback frequently

» Examine effective communication with shop stewards and union representatives

6.2.1 Practice cultural awareness	» Understand the need for cultural awareness and sensitivity, treating all people with fairness, respect and dignity<br>» Enable anti-discriminatory practices in the organisation, including HR and disciplinary procedures<br>» Enable cultural sensitivity, equality and fairness at all levels of the organisation<br>» Challenge discriminatory behaviour directly and sensitively<br>» Act in a non-discriminatory way toward individuals and groups<br>» Enable integrating cultural awareness in learning and development approaches<br>» Avoid stereotypical responses by examining one's own behaviour and bias<br>» Enable managing cultural diversity in teams and make the most of differences<br>» Compare strategies and/or resources for communicating effectively with people from different cultural backgrounds<br>» Enable the process to elicit information relating to values, beliefs and cultural backgrounds of consumers that may influence the way professional services are provided
Basal Technology Competency(ies):	7.4 Temperature and monitoring<br>7.6 ERP includes function of LMIS<br>7.12 Basic Office Skills<br>7.13 Have a command of technology
7.4 Temperature and monitoring	» Compare the current technology approaches for temperature monitoring and sensors<br>» Consider what is required to implement this technology in a country context<br>» Compare infrastructure requirement for heat-sensitive items at peripheral 'lower level' of SC depending on volume and nature of items to handle;<br>» Examine regular preventive maintenance and calibration of cold-chain equipment with biomedical engineers or logisticians<br>» Understand the policy for temperature monitoring tools during transportation and storage<br>» Understand the steps involved in health logistic management cycle: selection-order-delivery-reception-storage-distribution-inventory<br>» Understand hardware qualification and system validation of temperature monitoring<br>» Understand mass data management of temperature monitoring
7.6 ERP includes function of LMIS	» Enable a Master Data Management System<br>» Understand the critical components of an ERP system

	» Understand the meaning of common document types found within an ERP/LMIS<br>» Understand the logic of data visibility within an ERP<br>» Understand the importance of data integrity, record management and information security
7.12 Basic Office Skills	» Have a good understanding of common presentation authoring packages<br>» Have a good understanding of common spreadsheet authoring packages<br>» Have a good understanding of common document authoring packages<br>» Have a good understanding of common email authoring packages<br>» Have a good understanding of common instant messaging packages<br>» Have a good understanding of common video conferencing authoring packages
7.13 Have a command of technology	» Enable the use of technology suitable to the job held<br>» Understand the need of monitoring new developments and technologies in the sector<br>» Compare new technologies and recognising potential benefits for the sector<br>» Understand that resources and support are provided across the organisation to enable colleagues to make the best use of available technology<br>» Understand that the organisation has a strategy for technology use<br>» Compare how to minimise environmental damage through technology use<br>» Enable the use of technology to maximise effectiveness and efficiency<br>» Enable the use of field-based technology (e.g., radio, general packet radio service (GPRS), satellite phone)
Advanced Technology Competency(ies):	7.2 Blockchain<br>7.11 Cloud Computing
7.2 Blockchain	» Examine blockchain as a value-add capability for relevant global health use cases
7.11 Cloud Computing	» Understand cloud computing as a value-add capability for relevant global health use cases

Key Performance Indicators:
» % of Order lines received accurately<br>» % Orders/ Lines Processed Complete<br>» % Orders/ Lines Received On-Time to Demand Requirement<br>» % Orders/ lines received with correct packaging<br>» % Orders/ Lines Received with Correct Shipping Documents<br>» % of Order put-away within Put-Away cycle Time

Training:	Qualifications Available:	Certifications Available:
» Data management<br>» ERP Systems<br>» Legislation and Standards<br>» Discrepancy Reporting and Resolution<br>» ID & Damage Inspection<br>» Inventory Management<br>» Property Control and Disposition<br>» Return Management<br>» MSDS/CoC/BoL/Environmental Interpretation<br>» Logistics Management<br>» Bar Code Handling/RFID (if available)		» Further Education and Training Certificate: Archives and Records Management<br>» Receive stock in a DC warehouse skills programme<br>» Further Education and Training Certificate: Freight Forwarding and Customs Compliance

Head – Warehouse and Inventory Management

Organisation:

SC-focused Organisation

Competency Level:	Serial Number:	Primary Process:	Job Role:	Supervises:
Operational	3-311-351--2	Deliver	Head – Warehouse and Inventory Management	Assistant – Storage, Assistant – Inventory

Contributions	Accountable for the safe keeping and order lines of the warehouse. Ensures that best practices are used to ensure inventory accuracy, minimize obsolescence, shrinkage. Ensure that material handling equipment, space and human resource levels are available for the efficient operation of the warehouse function.
Technical Competency(ies):	3.1 Undertake storage, warehousing and inventory management<br>3.1.1 Manage storage of commodities during emergency<br>3.5 Manage disposal of products (e.g. expired, damaged, redundant products)<br>3.5.1 Define and direct process for managing redundant and returned stock<br>3.5.2 Manage process for disposal of returned stock

3.1 Undertake storage, warehousing and inventory management

» Enable distribution of products among suppliers, distribution centres, warehouses and customers through a logistics network

» Enable a total systems approach to designing and managing the entire flow of information, materials, and services

» Understand the choice of equipment or materials based on suitability for intended use, accuracy, safety of use and cost

» Understand the importance of warehouses to have formal storage locations that identify the row, rack section, level and shelf location, typically with an alphanumeric location bar code or label

» Understand that medicines are to be stored appropriately, considering temperature, access and cleanliness

» Enable use of, and ability to maintain, the cold chain

» Understand the movement and storage of materials within a warehouse

» Understand the need to secure the medical store and limit access to staff

» Understand and follow organisation processes, instructions, rules and parameters for warehouse and inventory management and use of equipment (e.g., annual stock turns, expiry date tracking, stock procurement, rotation and retrieval, equipment maintenance)

» Enable and understand policies and procedures for stock handling, distribution and withdrawal consistent with maintaining safety (e.g., policies for distribution of concentrated electrolyte solutions and cytotoxics, identification of cytotoxic drug products within the work environment, separation of like-named or like-packaged products)

	» Understand the information on the supplies being stored<br>» Enable the process of consolidating several items into larger units for fewer handlings<br>» Enable methods of stock rotation (e.g., first in first out, FIFO, or first to expire first out, FEFO)<br>» Understand the process of entering the quantity received and update the warehouse/inventory/LMIS system software with the stock on hand when each order is delivered according to requisite SOP's<br>» Understand that there are required levels of stock and/or equipment in specific situations<br>» Understand the importance of accurate verification of rolling stocks<br>» Enable the process of monitoring the location and condition of the supplies being stored<br>» Understand the importance of the information on the storage locations and facilities<br>» Examine any problems in using the storage locations and facilities
3.1.1 Manage storage of commodities during emergency	» Understand the types of warehouses with those available for emergency or disaster supply<br>» Understanding the importance of the choice of storage site for emergency or disaster supply<br>» Understanding of the staff required for the storage of emergency or disaster supply<br>» Compare the equipment and material required for the warehousing of emergency or disaster supply<br>» Enable the conduct of a rapid assessment of logistical needs in emergencies
3.5 Manage disposal of products (e.g. expired, damaged, redundant products)	
3.5.1 Define and direct process for managing redundant and returned stock	» Understand information on the supplies being returned<br>» Understand the flow of returned supplies<br>» Interpret any problems with the flow of returned supplies<br>» Compare options for improving the flow of returned supplies<br>» Understand procedures for monitoring the flow of returned supplies<br>» Understand the recall procedures to be used in response to a product recall notice or to access the information promptly.<br>» Understand issues relevant to the urgency and scope of action required in response to a product recall notice<br>» Explain the importance of reporting and the procedures for following up on medication incidents<br>» Understand follow-up strategies likely to be effective in preventing recurrence (e.g., root cause analysis)<br>» Enable an integrated healthcare waste management plan

	» Enable guides and procedures for collection, sorting, transport and disposal<br>» Examine pre-disposal inspections<br>» Understand the prerequisites for products disposal<br>» Understand product disposal report
3.5.2 Manage process for disposal of returned stock	» Enable the disposal of expired medications and/or medical equipment according to national policy<br>» Understand the recording system for disposal<br>» Enable the disposal of specific individual items of greatest risk with appropriate care (e.g., oncology medicine)
Personal/Management Competency(ies):	6.1 Demonstrate generic skills (e.g., literacy, numeracy, technology)<br>6.1.1 Exhibit high understanding of literacy and numeracy<br>6.2 Demonstrate strong communication skills<br>6.2.1 Practice cultural awareness
6.1 Demonstrate generic skills (e.g., literacy, numeracy, technology)	
6.1.1 Exhibit high understanding of literacy and numeracy	» Speak clearly and confidently, organize information in a logical manner, and consider voice tone and pace<br>» Respond to verbal messages and other cues (active listening)<br>» Express ideas and opinions clearly in written and verbal form<br>» Communicate information accurately, concisely and confidently in writing and verbally<br>» Practice a level of mathematics suitable to the job held<br>» Compare visual presentation techniques, including charting, histograms and flow sheets<br>» Read and comprehend at a level necessary to properly complete duties of the position<br>» Understand the need to interpret written information in documents, such as reports, SOPs, LMIS forms, graphs, calendars, schedules, notices and directions<br>» Understand the need to pay attention to detail and identify the main ideas, detect inconsistencies and identify missing information in documents<br>» Enable computer skills — such as using Word, Excel, PowerPoint and the internet — suitable to the job level<br>» Understand the process of analysing and validating KPIs<br>» Understand how the evaluation process of activities that act as standards against which the KPIs are measured is carried out<br>» Examine the process of performing critical self-review on work before submission
6.2 Demonstrate strong communication skills	» Be truthful and trustworthy, and supply accurate information at all times<br>» Enable working as part of a workplace team

» Understand vocabulary and communication style and form for both written and verbal communication that is appropriate for the situation, audience and material being communicated (e.g., avoid unnecessary jargon, clearly explain medical and SCM terminology)

» Enable open communication within constraints of confidentiality

» Participate in the organisation of communications about logistics

» Enable participation in meetings, expressing one's opinions, being aware of others' needs, and being appropriately assertive when required

» Show perspective into the point of view of others, understanding their needs and goals

» Enable the practise of building trust, rapport and credibility with others

» Understand the communication needs and concerns of others and respond to them

» Understand the need to avoid conflict between work and personal interests

» Enable meaningful two-way communication

» Show influence on others by persuasively presenting thoughts and ideas

» Show assertiveness skills to deal with unreasonable requests and/or refusals that would compromise practice or consumer care

» Maintain a positive, supportive and appreciative attitude

» Show the ability to balance the organisation's needs and the customer's needs

» Show self-control by maintaining composure and keeping emotions in check, even in difficult situations; deal calmly and effectively with stressful situations

» Enable expressing of opinions and providing information in written and/or verbal form in a manner that does not elicit concern, anger or other adverse response

» Enable the use of a systematic process for following up that demonstrates written reports have been received and understood

» Understand the means by which responses to input to the work environment are monitored

» Seek practical ways to overcome barriers to communication

» Understand the need to ensuring language representation (e.g., interpreters) at meetings when appropriate

» Interpret difficult situations and disputes between staff that need to be tackled and resolved

» Enable giving both positive and negative feedback sensitively

» Understand the need to maintain and improve communication

» Examine the process of giving and requesting feedback frequently

» Examine effective communication with shop stewards and union representatives

6.2.1 Practice cultural awareness

- Understand the need for cultural awareness and sensitivity, treating all people with fairness, respect and dignity
- Enable anti-discriminatory practices in the organisation, including HR and disciplinary procedures
- Enable cultural sensitivity, equality and fairness at all levels of the organisation
- Challenge discriminatory behaviour directly and sensitively
- Act in a non-discriminatory way toward individuals and groups
- Enable integrating cultural awareness in learning and development approaches
- Avoid stereotypical responses by examining one's own behaviour and bias
- Enable managing cultural diversity in teams and make the most of differences
- Compare strategies and/or resources for communicating effectively with people from different cultural backgrounds
- Enable the process to elicit information relating to values, beliefs and cultural backgrounds of consumers that may influence the way professional services are provided

Basal Technology Competency(ies):	7.4 Temperature and monitoring<br>7.5 Planning systems<br>7.6 ERP includes function of LMIS<br>7.12 Basic Office Skills

7.4 Temperature and monitoring

- Compare the current technology approaches for Temperature monitoring and sensors
- Consider what is required to implement this technology in a country context
- Compare infrastructure requirement for heat-sensitive items at peripheral 'lower level' of SC depending on volume and nature of items to handle;
- Examine regular preventive maintenance and calibration of cold-chain equipment with biomedical engineers or logisticians
- Understand the policy for temperature monitoring tools during transportation and storage
- Understand the steps involved in health logistic management cycle: selection-order-delivery-reception-storage-distribution-inventory
- Understand hardware qualification and system validation of temperature monitoring
- Understand mass data management of temperature monitoring

7.5 Planning systems

- Examine blockchain and understand the building blocks for planning systems
- Examine planning systems as a value-add capability for relevant global health use cases

7.6 ERP includes function of LMIS	» Enable a Master Data Management System<br>» Understand the critical components of an ERP system<br>» Understand the meaning of common document types found within an ERP/LMIS<br>» Understand the logic of data visibility within an ERP<br>» Understand the importance of data integrity, record management and information security
7.12 Basic Office Skills	» Have a good understanding of common presentation authoring packages<br>» Have a good understanding of common spreadsheet authoring packages<br>» Have a good understanding of common document authoring packages<br>» Have a good understanding of common email authoring packages<br>» Have a good understanding of common instant messaging packages<br>» Have a good understanding of common video conferencing authoring packages
Advanced Technology Competency(ies):	7.7 Automation<br>7.11 Cloud Computing
7.7 Automation	» Understand automation as a value-add capability for relevant global health use cases
7.11 Cloud Computing	» Understand cloud computing as a value-add capability for relevant global health use cases

Key Performance Indicators:

- No of Order Lines shipped with incorrectly captured Items - Number of order lines with incorrect items/sku's versus total number of order lines shipped as a %
- Accuracy of Risk Register
- Cycle time of QA/QC report
- Overall value at risk
- Partner/Donor integration, policy, guidelines and regulatory reforms
- QA/QC report
- Risk / Mitigation Costs
- Time to Recovery (TTR)

- Warehouse Efficiency - Time to Pick and Put away versus Planned Time to Pick and Put away. Presupposes the warehouse design promotes wave picking and flow
- Warehouse utilization - Volume used vs Volume Available
- Line Items SKU's handled vs Items / SKU's Planned over a planning horizon
- Number of warehouse locations used versus warehouse locations available (Pallet Positions may be used in larger warehouses)
- Safe product handling - Number of products handled during period out of conformance versus Number of products handled during period expressed as a % with prior period comparisons

Training:	Qualifications Available:	Certifications Available:
» Inventory Management<br>» Supply chain management<br>» Stores and warehousing<br>» Barcoding	»	» Manage Logistics Operations skills programme<br>» SAPICS Basics of stores and stock control certificate

Officer – Order Processing/ Management

Organisation: **SC-focused Organisation**				
Competency Level:	Serial Number:	Primary Process:	Job Role:	Supervises:
Operational	3-32-61-62-2	Deliver	Officer – Order Processing/ Management	Assistant – Pick, Pack

Contributions	Create the platform for all demand provident from end-users is timeously and accurately recorded. Feedback to the end-user when the promised date will likely deviate from the expected deliver date.
Technical Competency(ies):	3.2 Supply commodities to facilities
3.2 Supply commodities to facilities	» List the sources of distribution demand (customers)<br>» Understand the use of order policies for planned order generation<br>» Explain the calculation of re-supply quantities using a variety of inventory methods, including visual review, two bin, periodic review, order point and just in time<br>» Use order schedules<br>» Understand safety stock calculations<br>» Understand the principles of EOQ in the ordering process<br>» Understand the use of pull and push inventory control systems<br>» Enable a regular, rather than urgent, order culture<br>» Enable order-filling priorities in relation to delivery opportunities and urgency<br>» Understand that orders must be screened (modify order quantities on the basis of available stock, impact on service delivery, distance of facility from hospital)<br>» Enable assembling, checking, picking and packing of orders<br>» Understand making and using dispatch lists and invoices<br>» Enable the balancing of supply with demand, considering both lead time and demand variability created by supply patterns not matching demand patterns<br>» Understand information on the storage locations and facilities<br>» Understand measures of customer satisfaction by using performance metrics taken from the customer perspective, with criteria such as on-time delivery, perception of quality, complaints and length of wait times
Personal/Management Competency(ies):	6.1 Demonstrate generic skills (e.g., literacy, numeracy, technology)<br>6.1.1 Exhibit high understanding of literacy and numeracy<br>6.2 Demonstrate strong communication skills<br>6.2.1 Practice cultural awareness

6.1 Demonstrate generic skills (e.g., literacy, numeracy, technology)	
6.1.1 Exhibit high understanding of literacy and numeracy	» Speak clearly and confidently, organize information in a logical manner, and consider voice tone and pace<br>» Respond to verbal messages and other cues (active listening)<br>» Express ideas and opinions clearly in written and verbal form<br>» Communicate information accurately, concisely and confidently in writing and verbally<br>» Practice a level of mathematics suitable to the job held<br>» Compare visual presentation techniques, including charting, histograms and flow sheets<br>» Read and comprehend at a level necessary to properly complete duties of the position<br>» Understand the need to interpret written information in documents, such as reports, SOPs, LMIS forms, graphs, calendars, schedules, notices and directions<br>» Understand the need to pay attention to detail and identify the main ideas, detect inconsistencies and identify missing information in documents<br>» Enable computer skills — such as using Word, Excel, PowerPoint and the internet — suitable to the job level<br>» Understand the process of analysing and validating KPIs<br>» Understand how the evaluation process of activities that act as standards against which the KPIs are measured is carried out<br>» Examine the process of performing critical self-review on work before submission
6.2 Demonstrate strong communication skills	» Be truthful and trustworthy, and supply accurate information at all times<br>» Enable working as part of a workplace team<br>» Understand vocabulary and communication style and form for both written and verbal communication that is appropriate for the situation, audience and material being communicated (e.g., avoid unnecessary jargon, clearly explain medical and SCM terminology)<br>» Enable open communication within constraints of confidentiality<br>» Participate in the organisation of communications about logistics<br>» Enable participation in meetings, expressing one's opinions, being aware of others' needs, and being appropriately assertive when required<br>» Show perspective into the point of view of others, understanding their needs and goals<br>» Enable the practise of building trust, rapport and credibility with others<br>» Understand the communication needs and concerns of others and respond to them<br>» Understand the need to avoid conflict between work and personal interests<br>» Enable meaningful two-way communication<br>» Show influence on others by persuasively presenting thoughts and ideas

	» Show assertiveness skills to deal with unreasonable requests and/or refusals that would compromise practice or consumer care<br>» Maintain a positive, supportive and appreciative attitude<br>» Show the ability to balance the organisation's needs and the customer's needs<br>» Show self-control by maintaining composure and keeping emotions in check, even in difficult situations; deal calmly and effectively with stressful situations<br>» Enable expressing of opinions and providing information in written and/or verbal form in a manner that does not elicit concern, anger or other adverse response<br>» Enable the use of a systematic process for following up that demonstrates written reports have been received and understood<br>» Understand the means by which responses to input to the work environment are monitored<br>» Seek practical ways to overcome barriers to communication<br>» Understand the need to ensuring language representation (e.g., interpreters) at meetings when appropriate<br>» Interpret difficult situations and disputes between staff that need to be tackled and resolved<br>» Enable giving both positive and negative feedback sensitively<br>» Understand the need to maintain and improve communication<br>» Examine the process of giving and requesting feedback frequently<br>» Examine effective communication with shop stewards and union representatives
6.2.1 Practice cultural awareness	» Understand the need for cultural awareness and sensitivity, treating all people with fairness, respect and dignity<br>» Enable anti-discriminatory practices in the organisation, including HR and disciplinary procedures<br>» Enable cultural sensitivity, equality and fairness at all levels of the organisation<br>» Challenge discriminatory behaviour directly and sensitively<br>» Act in a non-discriminatory way toward individuals and groups<br>» Enable integrating cultural awareness in learning and development approaches<br>» Avoid stereotypical responses by examining one's own behaviour and bias<br>» Enable managing cultural diversity in teams and make the most of differences<br>» Compare strategies and/or resources for communicating effectively with people from different cultural backgrounds<br>» Enable the process to elicit information relating to values, beliefs and cultural backgrounds of consumers that may influence the way professional services are provided
Basal Technology Competency(ies):	7.5 Planning systems<br>7.6 ERP includes function of LMIS<br>7.12 Basic Office Skills<br>7.13 Have a command of technology

7.5 Planning systems	» Examine blockchain and understand the building blocks for planning systems<br>» Examine planning systems as a value-add capability for relevant global health use cases
7.6 ERP includes function of LMIS	» Enable a Master Data Management System<br>» Understand the critical components of an ERP system<br>» Understand the meaning of common document types found within an ERP/LMIS<br>» Understand the logic of data visibility within an ERP<br>» Understand the importance of data integrity, record management and information security
7.12 Basic Office Skills	» Have a good understanding of common presentation authoring packages<br>» Have a good understanding of common spreadsheet authoring packages<br>» Have a good understanding of common document authoring packages<br>» Have a good understanding of common email authoring packages<br>» Have a good understanding of common instant messaging packages<br>» Have a good understanding of common video conferencing authoring packages
7.13 Have a command of technology	» Enable the use of technology suitable to the job held<br>» Understand the need of monitoring new developments and technologies in the sector<br>» Compare new technologies and recognising potential benefits for the sector<br>» Understand that resources and support are provided across the organisation to enable colleagues to make the best use of available technology<br>» Understand that the organisation has a strategy for technology use<br>» Compare how to minimise environmental damage through technology use<br>» Enable the use of technology to maximise effectiveness and efficiency<br>» Enable the use of field-based technology (e.g., radio, general packet radio service (GPRS), satellite phone)

Key Performance Indicators:

- Customer order fulfilment cycle time & rate - Actual Order Fulfilment Cycle Time versus Promised Customer Order Fulfilment Cycle time
- Number of Order Lines rejected on Pick due to unavailability of stock
- Number of Order Lines delivered out of Quality Specifications
- Number of Order Lines delivered outside the promised date
- Number of Line Items short or excess delivered due to incorrect Order capture
- Accurate pick - Picking Accuracy number of order lines picked inaccurately divided by the total order lines picked (SKU and Quantity)
- Customer Incidents Report number of incidents by month/week/day comparison with previous periods

- No of order lines shipped with incorrectly captured Items - Number of order lines with incorrect items/SKU's versus total number of order lines shipped as a %
- Number of order lines delivered to incorrect end-user - Number of orders incorrectly addressed versus number of orders processed expressed as a %

Training:	Qualifications Available:	Certifications Available:
» Order Management<br>» Supply chain management<br>» Barcoding		» Manage Logistics Operations skills programme<br>» SAPICS Basics of stores and stock control certificate

Head – Transport and Delivery Management

Organisation:

SC-focused Organisation

Competency Level:	Serial Number:	Primary Process:	Job Role:	Supervises:
Operational	3-341-61-62-2	Deliver	Head – Transport and Delivery Management	Assistant – Delivery

Contributions	Create a platform to enable the most efficient delivery method, while complying to the Supply Chain Strategy. Ensure timely allocation of transport resources, select the most cost-efficient transport mode but ensure that delivery lead times are honoured to the end-user. Involved in outsourcing of transport resources, transport engineering focusing upon vehicle maintenance and ensuring cold chain integrity
Technical Competency(ies):	3.1 Undertake storage, warehousing and inventory management<br>3.1.1 Manage storage of commodities during emergency<br>3.4 Manage transport for commodities<br>3.4.1 Manage transport for commodities during disaster

3.1 Undertake storage, warehousing and inventory management

- Enable distribution of products among suppliers, distribution centres, warehouses and customers through a logistics network
- Enable a total systems approach to designing and managing the entire flow of information, materials, and services
- Understand the choice of equipment or materials based on suitability for intended use, accuracy, safety of use and cost
- Understand the importance of warehouses to have formal storage locations that identify the row, rack section, level and shelf location, typically with an alphanumeric location bar code or label
- Understand that medicines are to be stored appropriately, considering temperature, access and cleanliness
- Enable use of, and ability to maintain, the cold chain
- Understand the movement and storage of materials within a warehouse
- Understand the need to secure the medical store and limit access to staff
- Understand and follow organisation processes, instructions, rules and parameters for warehouse and inventory management and use of equipment (e.g., annual stock turns, expiry date tracking, stock procurement, rotation and retrieval, equipment maintenance)
- Enable and understand policies and procedures for stock handling, distribution and withdrawal consistent with maintaining safety (e.g., policies for distribution of concentrated electrolyte solutions and cytotoxics, identification of cytotoxic drug products within the work environment, separation of like-named or like-packaged products)
- Understand the information on the supplies being stored

	» Enable the process of consolidating several items into larger units for fewer handlings<br>» Enable methods of stock rotation (e.g., first in first out, FIFO, or first to expire first out, FEFO)<br>» Understand the process of entering the quantity received and update the warehouse/inventory/LMIS system software with the stock on hand when each order is delivered according to requisite SOP's<br>» Understand that there are required levels of stock and/or equipment in specific situations<br>» Understand the importance of accurate verification of rolling stocks<br>» Enable the process of monitoring the location and condition of the supplies being stored<br>» Understand the importance of the information on the storage locations and facilities<br>» Examine any problems in using the storage locations and facilities
3.1.1 Manage storage of commodities during emergency	» Understand the types of warehouses with those available for emergency or disaster supply<br>» Understanding the importance of the choice of storage site for emergency or disaster supply<br>» Understanding of the staff required for the storage of emergency or disaster supply<br>» Compare the equipment and material required for the warehousing of emergency or disaster supply<br>» Enable the conduct of a rapid assessment of logistical needs in emergencies
3.4 Manage transport for commodities	» Understand distribution activities, including a fleet of vehicles and distribution schedules, to deliver health commodities to facilities.<br>» Understand the policies and procedures related to handling and transporting special products such as vaccines, HIV/AIDS drugs, other high value products and narcotics<br>» Understands shipping methods, considering trade-offs between costs and benefits<br>» Understand local and national laws/requirements for vehicle safety, driving regulations and licensing<br>» Understand proper packing and labelling methods for transportation of hazardous materials<br>» Understand the process of maximizing freight loads while minimizing freight costs<br>» Enable efficient use of transportation resources while meeting customers' needs<br>» Examine movement demands with vehicle resources<br>» Understand quantitative techniques when solving logistics problems, such as designing routes and scheduling vehicles<br>» Understand the process of delivering products to hospitals and district health offices with the accompanying dispatch note and report and requisition forms<br>» Coordinate transport options for order delivery<br>» Understand that vehicle availability is due to a vehicle maintenance plan

	» Interpret information on distribution requirements<br>» Examine any problems in distribution requirements<br>» Enable the process of equipping distribution sites<br>» Understand the need to inform stakeholders for better coordination of distribution<br>» Compare tracking devices (tools and indicators)<br>» Examine the process of training of drivers, van assistants, fleet administrators and expeditors<br>» Examine the process of supporting investigations around missed OTIF and other failures
3.4.1 Manage transport for commodities during disaster	» Be able to compare of the different types of transport and their characteristics for emergency or disaster supply<br>» Understand the process of developing a plan for the achievement of a rapid response logistics plan for deployment in the event of a medical emergency.
Personal/Management Competency(ies):	5.1 Design and implement supply chain system and strategies<br>5.5 Manage and plan projects<br>5.5.2 Execute strategic decision making<br>6.1 Demonstrate generic skills (e.g., literacy, numeracy, technology)<br>6.1.1 Exhibit high understanding of literacy and numeracy<br>6.2 Demonstrate strong communication skills<br>6.2.1 Practice cultural awareness
5.1 Design and implement supply chain system and strategies	» Enable a responsive, agile and efficient supply chain that has the ability to meet the changing needs of customers and deliver high-quality products with short lead times at low cost<br>» Examine the interrelationship of organisations, people, technology, activities, information, and resources involved in moving a product from supplier to customer<br>» Understand the management activities carried out in the course of running an organisation, including controlling, leading, monitoring, adjusting, organising and planning<br>» Understand the characteristics unique to public health, demonstrate awareness of the factors that could contribute to or hinder the delivery of logistics services<br>» Examine emerging concepts and principles in public health logistics; adapt current practices to incorporate new developments in the field<br>» Compare which type of inventory control system will be most effective for a particular programme or country<br>» Compare the difference between continuous and period review systems of inventory control<br>» Enable and follow a formal logistics strategy and plan
5.5 Manage and plan projects	

5.5.2 Execute strategic decision making	» Understand that one can make decisions regarding one's own workload and area of responsibility<br>» Understand of need to prioritize competing tasks and perform them quickly and efficiently according to their importance; finding new ways of organizing or planning work to accomplish it more efficiently<br>» Understand the need to ensure proper time and space for consultation around decisions being made.<br>» Follow the process of considering the input of resources including staff needed<br>» Understand the need to check assumptions against facts<br>» Examine the key issues in a complex situation and come to the heart of the problem quickly<br>» Examine relevant information before making decisions<br>» Understand that strategic decisions are made in the interest of the organisation's goals<br>» Understand when to analyse, evaluate and execute tough decisions when necessary<br>» Understand the impact of decisions on others' work or team goals<br>» Understand the process to communicate decisions and ensure they are incorporated into policies and processes<br>» Understand inclusive and consultative strategic planning to establish strategic objectives<br>» Examine and enable emerging trends and practices<br>» Understand the macro and long-term consequences of decisions
6.1 Demonstrate generic skills (e.g., literacy, numeracy, technology)	
6.1.1 Exhibit high understanding of literacy and numeracy	» Speak clearly and confidently, organize information in a logical manner, and consider voice tone and pace<br>» Respond to verbal messages and other cues (active listening)<br>» Express ideas and opinions clearly in written and verbal form<br>» Communicate information accurately, concisely and confidently in writing and verbally<br>» Practice a level of mathematics suitable to the job held<br>» Compare visual presentation techniques, including charting, histograms and flow sheets<br>» Read and comprehend at a level necessary to properly complete duties of the position<br>» Understand the need to interpret written information in documents, such as reports, SOPs, LMIS forms, graphs, calendars, schedules, notices and directions<br>» Understand the need to pay attention to detail and identify the main ideas, detect inconsistencies and identify missing information in documents

- Enable computer skills — such as using Word, Excel, PowerPoint and the internet — suitable to the job level
- Understand the process of analysing and validating KPIs
- Understand how the evaluation process of activities that act as standards against which the KPIs are measured is carried out
- Examine the process of performing critical self-review on work before submission

6.2 Demonstrate strong communication skills	» Be truthful and trustworthy, and supply accurate information at all times<br>» Enable working as part of a workplace team<br>» Understand vocabulary and communication style and form for both written and verbal communication that is appropriate for the situation, audience and material being communicated (e.g., avoid unnecessary jargon, clearly explain medical and SCM terminology)<br>» Enable open communication within constraints of confidentiality<br>» Participate in the organisation of communications about logistics<br>» Enable participation in meetings, expressing one's opinions, being aware of others' needs, and being appropriately assertive when required<br>» Show perspective into the point of view of others, understanding their needs and goals<br>» Enable the practise of building trust, rapport and credibility with others<br>» Understand the communication needs and concerns of others and respond to them<br>» Understand the need to avoid conflict between work and personal interests<br>» Enable meaningful two-way communication<br>» Show influence on others by persuasively presenting thoughts and ideas<br>» Show assertiveness skills to deal with unreasonable requests and/or refusals that would compromise practice or consumer care<br>» Maintain a positive, supportive and appreciative attitude<br>» Show the ability to balance the organisation's needs and the customer's needs<br>» Show self-control by maintaining composure and keeping emotions in check, even in difficult situations; deal calmly and effectively with stressful situations<br>» Enable expressing of opinions and providing information in written and/or verbal form in a manner that does not elicit concern, anger or other adverse response<br>» Enable the use of a systematic process for following up that demonstrates written reports have been received and understood<br>» Understand the means by which responses to input to the work environment are monitored<br>» Seek practical ways to overcome barriers to communication<br>» Understand the need to ensuring language representation (e.g., interpreters) at meetings when appropriate<br>» Interpret difficult situations and disputes between staff that need to be tackled and resolved<br>» Enable giving both positive and negative feedback sensitively

	» Understand the need to maintain and improve communication<br>» Examine the process of giving and requesting feedback frequently<br>» Examine effective communication with shop stewards and union representatives
6.2.1 Practice cultural awareness	» Understand the need for cultural awareness and sensitivity, treating all people with fairness, respect and dignity<br>» Enable anti-discriminatory practices in the organisation, including HR and disciplinary procedures<br>» Enable cultural sensitivity, equality and fairness at all levels of the organisation<br>» Challenge discriminatory behaviour directly and sensitively<br>» Act in a non-discriminatory way toward individuals and groups<br>» Enable integrating cultural awareness in learning and development approaches<br>» Avoid stereotypical responses by examining one's own behaviour and bias<br>» Enable managing cultural diversity in teams and make the most of differences<br>» Compare strategies and/or resources for communicating effectively with people from different cultural backgrounds<br>» Enable the process to elicit information relating to values, beliefs and cultural backgrounds of consumers that may influence the way professional services are provided
Basal Technology Competency(ies):	7.5 Planning systems<br>7.6 ERP includes function of LMIS<br>7.12 Basic Office Skills<br>7.15 Global positioning system (GPS)
7.5 Planning systems	» Examine blockchain and understand the building blocks for planning systems<br>» Examine planning systems as a value-add capability for relevant global health use cases
7.6 ERP includes function of LMIS	» Enable a Master Data Management System<br>» Understand the critical components of an ERP system<br>» Understand the meaning of common document types found within an ERP/LMIS<br>» Understand the logic of data visibility within an ERP<br>» Understand the importance of data integrity, record management and information security
7.12 Basic Office Skills	» Have a good understanding of common presentation authoring packages<br>» Have a good understanding of common spreadsheet authoring packages<br>» Have a good understanding of common document authoring packages<br>» Have a good understanding of common email authoring packages

	» Have a good understanding of common instant messaging packages<br>» Have a good understanding of common video conferencing authoring packages
7.15 Global positioning system (GPS)	» Understand GPS as a value-add capability for relevant global health use cases
Advanced Technology Competency(ies):	7.3 Unmanned Aerial Vehicles (UAVs)<br>7.7 Automation
7.3 Unmanned Aerial Vehicles (UAVs)	» Understand the current UAV landscape, their application and the enabling environment required to support country implementation<br>» Understand UAVs as a value-add capability for relevant global health use cases<br>» Understand the laws/ regulations around the use of UAV's
7.7 Automation	» Understand automation as a value-add capability for relevant global health use cases

Key Performance Indicators:

» Customer Incidents Report number of incidents by month/week/day comparison with previous periods

» No of Order Lines shipped with incorrectly captured Items - Number of order lines with incorrect items/sku's versus total number of order lines shipped as a %

» % Product Transferred On-Time to Demand Requirement. Actual Number of products transferred outside of the Time Limits vs Number of products Transferred.

» Transfer Product Cycle Time - Actual Transfer Cycle Time versus Planned Transferred Cycle Time

» On-time delivery - Actual Delivery Date & Time Vs Promised Delivery Data and Time

» Warehousing and Inventory Management OTIF - On time and in full under the individual's control

» Vehicle utilisation based on time and capacity in cubic metres

» Vehicle downtime incorporating a range of non-availability parameters e.g. routine maintenance, unscheduled repairs, accident damage and driver illness

» Number of cold chain infringements

» Driver time utilisation incorporating a range of activity parameters e.g. driving time, unloading time and yard/hub time

Training:	Qualifications Available:	Certifications Available:
» Radio Frequency Identification (RFID)<br>» vehicle routing<br>» scheduling systems<br>» asset management systems<br>» Supply chain management<br>» Barcoding<br>» Transport Management<br>» Cold chain management		» Manage Logistics Operations skills programme<br>» Apply fundamental concepts and principles of supply chain management skills programme<br>» International Diploma in Logistics & Transport

Lead – Waste and Returns Management

Organisation:

SC-focused Organisation

Competency Level:	Serial Number:	Primary Process:	Job Role:	Supervises:
Operational	3-35-61-62-4	Return	Lead – Waste and Returns Management	-

Contributions	Execute on the Green process elements of the Return Processes Is cognisant of the Green requirements as per the Supply Chain Strategy of the Public Health System Organisation.
Technical Competency(ies):	3.5 Manage disposal of products (e.g. expired, damaged, redundant products)<br>3.5.1 Define and direct process for managing redundant and returned stock<br>3.5.2 Manage process for disposal of returned stock

3.5 Manage disposal of products (e.g. expired, damaged, redundant products)

3.5.1 Define and direct process for managing redundant and returned stock	» Evaluate information on the supplies being returned<br>» Collate, prepare and analyse data on the flow of returned supplies<br>» Resolve any problems with the flow of returned supplies<br>» Recommend options for improving the flow of returned supplies<br>» Analyse procedures for monitoring the flow of returned supplies<br>» Determine recall procedures to be used in response to a product recall notice or to access the information promptly.<br>» Demonstrate knowledge of issues relevant to the urgency and scope of action required in response to a product recall notice

	» Analyse the information provided in the reports when following up on medication incidents<br>» Evaluate follow-up strategies likely to be effective in preventing recurrence (e.g., root cause analysis)<br>» Evaluate an integrated healthcare waste management plan<br>» Evaluate guides and procedures for collection, sorting, transport and disposal<br>» Evaluate pre-disposal inspections<br>» Evaluate the prerequisites for products disposal<br>» Evaluate product disposal report
3.5.2 Manage process for disposal of returned stock	» Analyse the process of disposing of expired medications and/or medical equipment according to national policy<br>» Evaluate an appropriate recording system for disposal<br>» Analyse the process of disposal for specific individual items of greatest risk with appropriate care (e.g., oncology medicine)
Personal/Management Competency(ies):	5.5 Manage and plan projects<br>5.5.2 Execute strategic decision making<br>6.5 Prove leadership abilities<br>6.5.1 Demonstrate resilience and ability to manage stress<br>6.6 Abide by rules / laws / legislation
5.5 Manage and plan projects	»
5.5.2 Execute strategic decision making	» Determine how one can make decisions regarding one's own workload and area of responsibility<br>» Determine the process of prioritizing competing tasks and perform them quickly and efficiently according to their importance; finding new ways of organizing or planning work to accomplish it more efficiently<br>» Evaluate the proper time and space for consultation around decisions being made.<br>» Demonstrate the process of considering the input of resources including staff needed<br>» Analyse assumptions against facts<br>» Determine the key issues in a complex situation and come to the heart of the problem quickly<br>» Evaluate relevant information before making decisions<br>» Provide input to strategic decisions in the interest of the organisation's goals<br>» Evaluate when to analyse, evaluate and execute tough decisions when necessary<br>» Analyse the impact of decisions on others' work or team goals<br>» Communicate decisions and ensure they are incorporated into policies and processes

- Demonstrate inclusive and consultative strategic planning to establish strategic objectives
- Evaluate emerging trends and practices
- Analyse the macro and long-term consequences of decisions

6.5 Prove leadership abilities

- Analyse input to meetings and programme development
- Look for future trends and issues, and assist the organisation in meeting the challenges
- Contribute to a collaborative working environment
- Demonstrate a drive for change and improvement develops opportunities for the organisation and sector
- Demonstrate channelling energy and ideas toward resolving issues
- Demonstrate the practise of communicating with influence
- Demonstrate showing courage to take an unpopular stance when needed
- Demonstrate steering and implementing change organisationally
- Demonstrate anticipating and resolving conflict
- Demonstrate showing initiative in working methods
- Exemplify personal drive and integrity
- Demonstrate serving as a role model for others to follow
- Demonstrate leadership and practice management skills, initiative and efficiency
- Demonstrate inspiring others through professional excellence, innovation and communication
- Determine situations in which a change in leadership style would be warranted (e.g., directive versus consultative style in the event of a crisis)

6.5.1 Demonstrate resilience and ability to manage stress

- Demonstrate identifying symptoms of stress and take steps to reduce stress
- Demonstrate seeing the bigger picture and help others to
- Demonstrate recovering quickly from setbacks
- Demonstrate drawing on previous experience and support mechanisms to reduce the impact of stress on self and others
- Determine a working environment that aims to minimise pressure and stress
- Demonstrate coping well under pressure, particularly in difficult environments
- Demonstrate acting as a role model for others and display courage under difficult circumstances
- Demonstrate recognising the limitations of staff and taking action to limit their exposure to harm when needed
- Demonstrate making use of personal support mechanisms
- Demonstrate helping others identify personal support mechanisms

	» Determine organisational policy to support self-care in agencies
**6.6 Abide by rules / laws / legislation**	» Demonstrate compliance with the legislation that covers the practice of public health supply chains and healthcare, and describe its purpose<br>» Analyse requirements of professional codes, guidelines and standards adopted as part of the legislative framework<br>» Demonstrate keeping up to date with changes in legal instruments, as informed by national-level managers<br>» Demonstrate compliance with international regulations in decision making for the distribution system, including customs regulations on import and export requirements (e.g., trade tariffs and duties on imported goods) and security regulations (e.g., 2007 SAFE Ports Act, NAFTA or European Union trade agreements)<br>» Demonstrate applying and understanding regulatory affairs and the key aspects of pharmaceutical registration and legislation<br>» Demonstrate knowledge of the principles of business economics and intellectual property rights, including the basics of patent interpretation<br>» Demonstrate knowledge of applicable procurement law and the practical effects of a contract's terms and conditions<br>» Evaluate current industry and government regulations governing sustainability<br>» Demonstrate a working knowledge of key hazardous materials handling laws<br>» Determine policies and procedures relating to workplace safety that are consistent with agreed or recognised standards<br>» Analyse professional standards and conventions, as well as workplace policies and procedures, for preparing pharmaceutical products<br>» Demonstrate all standard operating procedures<br>» Evaluate the process of complying with data privacy and data protection laws
Basal Technology Competency(ies):	7.4 Temperature and monitoring<br>7.6 ERP includes function of LMIS<br>7.12 Basic Office Skills<br>7.13 Have a command of technology
**7.4 Temperature and monitoring**	» Analyse the current technology approaches for Temperature monitoring and sensors<br>» Evaluate what is required to implement this technology in a country context<br>» Evaluate infrastructure requirement for heat-sensitive items at peripheral 'lower level' of SC depending on volume and nature of items to handle;<br>» Analyse regular preventive maintenance and calibration of cold-chain equipment with biomedical engineers or logisticians<br>» Evaluate the policy for temperature monitoring tools during transportation and storage

	» Demonstrate the steps involved in health logistic management cycle: selection-order-delivery-reception-storage-distribution-inventory<br>» Determine hardware qualification and system validation of temperature monitoring<br>» Evaluate mass data management of temperature monitoring
7.6 ERP includes function of LMIS	» Evaluate a Master Data Management System<br>» Demonstrate an understanding of the critical components of an ERP system<br>» Demonstrate an understanding of the meaning of common document types found within an ERP/LMIS<br>» Demonstrate an understanding of the logic of data visibility within an ERP<br>» Demonstrate the importance of data integrity, record management and information security
7.12 Basic Office Skills	» Demonstrate a good understanding of common presentation authoring packages<br>» Demonstrate a good understanding of common spreadsheet authoring packages<br>» Demonstrate a good understanding of common document authoring packages<br>» Demonstrate a good understanding of common email authoring packages<br>» Demonstrate a good understanding of common instant messaging packages<br>» Demonstrate a good understanding of common video conferencing authoring packages
7.13 Have a command of technology	» Demonstrate the use of technology suitable to the job held<br>» Monitor new developments and technologies in the sector<br>» Experiment with new technologies and recognising potential benefits for the sector<br>» Ensure that resources and support are provided across the organisation to enable colleagues to make the best use of available technology<br>» Determine what the organisations' strategy is for technology use<br>» Determine how to minimise environmental damage through technology use<br>» Analyse the use of technology to maximise effectiveness and efficiency<br>» Analyse the use of field-based technology (e.g., radio, general packet radio service (GPRS), satellite phone)
Advanced Technology Competency(ies):	7.7 Automation

7.7 Automation	» Evaluate automation as a value-add capability for relevant global health use cases

Key Performance Indicators:

- Availability - Stock out rate - Number of missed line item quantity deliveries due to stock out versus total line item quantity deliveries
- Safe product handling - Number of products handled during period out of conformance versus Number of products handled during period expressed as a % with prior period comparisons
- Customer Incidents Report number of incidents by month/week/day comparison with previous periods
- No of Order Lines shipped with incorrectly captured Items - Number of order lines with incorrect items/SKU's versus total number of order lines shipped as a %
- % Product Transferred On-Time to Demand Requirement. Actual Number of products transferred outside of the Time Limits vs Number of products Transferred.
- Transfer Product Cycle Time - Actual Transfer Cycle Time versus Planned Transferred Cycle Time
- Accurate pick - Picking Accuracy number of order lines picked inaccurately divided by the total order lines picked (SKU and Quantity)
- On-time delivery - Actual Delivery Date & Time Vs Promised Delivery Data and Time
- Customer order fulfilment cycle time & rate - Actual Order Fulfilment Cycle Time versus Promised Customer Order Fulfilment Cycle time

Training:	Qualifications Available:	Certifications Available:
» Regulatory Policy Management<br>» Warranty process and policy<br>» Lot Tracking	» Bachelor of Commerce in Logistics Management<br>» Bachelor of Business Administration in Logistics and Supply Chain Management<br>» Bachelor of Commerce	» APICS Certified in logistics transportation and distribution certificate<br>» Lean Six Sigma Certification in Healthcare black belt

Manager - Occupational Safety

Organisation:

SC-focused Organisation

Competency Level:	Serial Number:	Primary Process:	Job Role:	Supervises:
Operational	3-39-554-641-3	Enable – Regulatory (Individual) Compliance	Manager - Occupational Safety	-

Contributions	Responsible for compliance to the health and safety guidelines for the organisation. Promoting a safe and healthy working environment in the organisation. Responsible to reduce the number of work-related accidents and diseases in the organisation. Responsible to provide equitable compensation benefits to those who may get injured in work-related accidents or contract occupational diseases. Ensures the surveillance of the working environment
Technical Competency(ies):	3.9 Facility design

3.9 Facility design

- Provide inputs to the design and layout of the size and configuration of the facility/building.
- Describe the flow of product through the facility to making sure it is achievable with the selection of material handling equipment
- Achieve the balance of the health and safety requirements of the facility with its performance requirements

Personal/Management Competency(ies):

- 5.5 Manage and plan projects
- 5.5.4 Direct/participate in teamwork
- 6.1 Demonstrate generic skills (e.g., literacy, numeracy, technology)
- 6.1.1 Exhibit high understanding of literacy and numeracy
- 6.2 Demonstrate strong communication skills
- 6.2.1 Practice cultural awareness
- 6.4 Exhibit professional and ethical values
- 6.4.1 Demonstrate integrity

5.5 Manage and plan projects

5.5.4 Direct/participate in teamwork

- Describe the process of working with team members within their area of authority to establish achievable goals and strategies that are consistent with the objectives established for the organisation as a whole
- Describe an organisational chart that shows the lines of reporting and responsibility among staff
- Actively contribute a perspective and make a positive contribution to team-based problem solving and decision making
- Provide feedback, encouragement and support to team members for progressing strategic goals

	» Define team performance in relation to the organisation's mission and goals<br>» Encourage teams to think for themselves and resolve problems<br>» Encourage and harness diversity within the team to boost team effectiveness<br>» Encourage and support the team to work through its stages of development and perform well<br>» Describe roles and responsibilities in relation to employees' expertise and the expectations of collaborating team members<br>» Describe where their position fits in the structure (organisation, SC, healthcare sector) , the responsibilities their position entails and the accountabilities linked to the position
6.1 Demonstrate generic skills (e.g., literacy, numeracy, technology)	
6.1.1 Exhibit high understanding of literacy and numeracy	» Speak clearly and confidently, organize information in a logical manner, and consider voice tone and pace<br>» Respond to verbal messages and other cues (active listening)<br>» Express ideas and opinions clearly in written and verbal form<br>» Communicate information accurately, concisely and confidently in writing and verbally<br>» Practice a level of mathematics suitable to the job held<br>» Describe visual presentation techniques, including charting, histograms and flow sheets<br>» Read and comprehend at a level necessary to properly complete duties of the position<br>» Interpret written information in documents, such as reports, SOPs, LMIS forms, graphs, calendars, schedules, notices and directions<br>» Pay attention to detail and identify the main ideas, detect inconsistencies and identify missing information in documents<br>» Apply computer skills — such as using Word, Excel, PowerPoint and the internet — suitable to the job level<br>» Describe the process of analysing and validating KPIs<br>» Participate in the evaluation of activities that act as standards against which the KPIs are measured<br>» Apply the process of performing critical self-review on work before submission
6.2 Demonstrate strong communication skills	» Be truthful and trustworthy, and supply accurate information at all times<br>» Work as part of a workplace team<br>» Describe vocabulary and communication style and form for both written and verbal communication that is appropriate for the situation, audience and material being communicated (e.g., avoid unnecessary jargon, clearly explain medical and SCM terminology)<br>» Encourage open communication within constraints of confidentiality

	» Organize communications about logistics<br>» Encourage participation in meetings, expressing one's opinions, being aware of others' needs, and being appropriately assertive when required<br>» Apply perspective into the point of view of others, understanding their needs and goals<br>» Encourage building trust, rapport and credibility with others<br>» Anticipate the communication needs and concerns of others and respond to them<br>» Avoid conflict between work and personal interests<br>» Encourage meaningful two-way communication<br>» Influence others by persuasively presenting thoughts and ideas<br>» Apply assertiveness skills to deal with unreasonable requests and/or refusals that would compromise practice or consumer care<br>» Maintain a positive, supportive and appreciative attitude<br>» Apply the ability to balance the organisation's needs and the customer's needs<br>» Use self-control by maintaining composure and keeping emotions in check, even in difficult situations; deal calmly and effectively with stressful situations<br>» Encourage the expressing of opinions and providing information in written and/or verbal form in a manner that does not elicit concern, anger or other adverse response<br>» Apply the use of a systematic process for following up that demonstrates written reports have been received and understood<br>» Describe the means by which responses to input to the work environment are monitored<br>» Seek practical ways to overcome barriers to communication<br>» Identify language representation (e.g., interpreters) at meetings when appropriate<br>» Tackle difficult situations and resolve disputes between staff<br>» Give both positive and negative feedback sensitively<br>» Maintain and improve communication<br>» Apply the process of giving and requesting feedback frequently<br>» Apply the ability to communicate effectively with shop stewards and union representatives
6.2.1 Practice cultural awareness	» Describe cultural awareness and sensitivity, treating all people with fairness, respect and dignity<br>» Describe anti-discriminatory practices in the organisation, including HR and disciplinary procedures<br>» Establish cultural sensitivity, equality and fairness at all levels of the organisation<br>» Challenge discriminatory behaviour directly and sensitively<br>» Act in a non-discriminatory way toward individuals and groups

- Integrate cultural awareness in learning and development approaches
- Avoid stereotypical responses by examining one's own behaviour and bias
- Manage cultural diversity in teams and make the most of differences
- Describe strategies and/or resources for communicating effectively with people from different cultural backgrounds
- Elicit information relating to values, beliefs and cultural backgrounds of consumers that may influence the way professional services are provided

6.4 Exhibit professional and ethical values

6.4.1 Demonstrate integrity

- Accept responsibility for one's own work tasks and performance
- Work within a framework of clearly understood humanitarian values and ethics
- Stand by decisions and hold others accountable when necessary
- Does not abuse one's own power or position
- Manage team members to ensure they do not abuse their power or position
- Identify when individuals or the organisation is straying from organisation goals, and challenge them to uphold ethics
- Resist undue political pressure in decision making
- Support staff in maintaining ethical stances
- Make time in team for ethical inquiry and reflection
- Show consistency between expressed principles and behaviour
- Provide input to ensure that principles, values and ethics are embedded in policy
- Act without consideration of personal gain
- Promote transparency in decision making structures and processes
- Provide input on ensuring programmes are acting with integrity, and recognising the impact of not doing so
- Recognise one's own limitations and act upon them
- Apply all standard operating procedures
- Work in a safe and legal way
- Demonstrate respect, dignity and consideration for consumers
- Discuss the impact of a no-blame culture on reporting and preventing recurrence of incidents
- Describe requests of colleagues that might be regarded as unreasonable
- Communicate (verbally and by example) expectations of the desired standards and approaches to be adopted
- Describe roles and responsibilities in terms of the position statement/duty statement of the position held
- Demonstrate punctuality

- Explain the obligation to apply professional care and expertise to deliver high quality of services
- Demonstrate care and attention to detail in undertaking work activities
- Describe appropriate attire and presentation for the role and situation
- Recognise and take responsibility for emotions
- Describe an environment in which others can talk and act without fear of repercussion
- Seek to keep commitments and not let people down
- Be trusting and cooperative when working alongside others
- Take on various responsibilities within the department as the need arises

Key Performance Indicators:
» Safety statics - Number of cases due to human error ranked by seriousness this period versus previous periods. A graphical representation would be indicated.<br>» Safety statics - Number of cases due to faulty equipment, ranked by seriousness of injury this period versus previous periods. A graphical representation would be indicated.<br>» Audit of safety equipment results this period versus previous periods<br>» Audit of Occupational safety signage and posters. This period versus previous periods<br>» Occupational safety educational awareness programmes delivered. Actual this period versus Planned this period<br>» Root cause analysis reports per reported incident. This period versus Last Periods

Training:	Qualifications Available:	Certifications Available:
» SHE compliance training<br>» Reporting standards training	»	» APICS Principles of operations management series certificate<br>» Logistics and Supply Chain Management MIT<br>» SCPro™ Certification<br>» Occupational Certificate: Maintenance Planner<br>» Occupational Certificate: Compliance Officer

Lead - Demand and Supply Planning

Organisation: SC-focused Organisation				
Competency Level:	Serial Number:	Primary Process:	Job Role:	Supervises:
Operational	1-14-15-2	Plan – Supply Chain	Lead – Demand and Supply Planning	Officer – Demand and Supply Planning, Officer – Inventory and Demand Planning

Contributions	Responsible for assisting managers in receiving deliver and procure plans and balancing the supply chain with regards to incoming stock, stock on hand and capacity
Technical Competency(ies):	1.1 Select the appropriate product<br>1.2 Define the specifications of the product, including product quality<br>1.3 List any special considerations for the product (e.g., temperature requirements, size, implications for infrastructure)<br>1.4 Forecast and quantify product needs<br>1.5 Supply planning
1.1 Select the appropriate product	» Compare the broad concepts of the national medicines policy, essential medicines lists, essential equipment lists, standard treatment guidelines and “dangerous drug” (DDA) policy<br>» Understand the government system required to add and subtract items from the essential medicines list and the essential equipment list<br>» Understand that the government system required to alter standard treatment guidelines, dangerous drug policy and national medicines policy<br>» Compare the type of supplies and services that are required<br>» Provide input for regularly scheduled coordination meetings with stakeholders involved in financing, procuring or distributing commodities<br>» Understand the steps needed to bring a medicinal product to the market, including the safety, quality, efficacy and pharmacoeconomic assessments of the product
1.2 Define the specifications of the product, including product quality	» Understand specifications for procurement<br>» Compare the characteristics of a good specification<br>» Compare the types of specifications<br>» Name the advantages of functional and performance specifications<br>» Examine the contents of a specification<br>» Name the procurement staff responsibilities in the specification process<br>» Compare the quality standard of product<br>» Not Applicable
1.3 List any special considerations for the	» Compare the principles and processes of category management, including market segmentation principles

product (e.g., temperature requirements, size, implications for infrastructure)	» Examine any specific considerations in the quantification of programme-specific products (e.g., ARVs, family planning commodities, vaccines)<br>» Examine current international trends in commodity availability<br>» Compare regulatory requirements for the product
1.4 Forecast and quantify product needs	» Examine the factors that affect usage patterns of medications and equipment and how this affects ordering (e.g., disease outbreaks), using national policies as a guide and to ensure consistent application<br>» Understand principles and applications of demand forecasting<br>» Compare the critical requirements for effective forecasting: establishing time horizons, level of detail and use of data<br>» Understand policies and procedures for forecasting<br>» Understand the process of quantifying product requirements using a variety of methods<br>» Examine consumption data at the national level for various country programmes<br>» Compare and reconcile different types of forecasts<br>» Understand the process of applying VEN or ABC analysis to programme requirements for national level procurement<br>» Understand the importance of calculating average monthly dispensed-to-user quantities for all service delivery points nationally<br>» Understand the importance of calculating storage space requirements for all levels in the supply chain<br>» Understand the importance of calculating the months of supply on hand for each commodity at the national level<br>» Examine the maximum and minimum stock levels for each level in a programme<br>» Understand various tools used in quantification (e.g., quantification software)<br>» Understand the use of various data sources for commodity forecasting (e.g., consumption data, services data, demographic data)<br>» Compare key performance indicators of forecast accuracy<br>» Examine the degree of error when using forecasting methods
1.5 Supply planning	» Examine a purchasing and supply plan in line with national priorities<br>» Understand the importance of balancing of supply chain resources and trigger activities to correct any imbalances<br>» Understand the formalisation of sales and operations plans through sales and operations meeting.<br>» Understand that the sales and operations plan must be translated into executable plans to ensure dispatch of goods in line with overall priorities.<br>» Understand the national commodity pipeline
Personal/Management Competency(ies):	6.1 Demonstrate generic skills (e.g., literacy, numeracy, technology)<br>6.1.1 Exhibit high understanding of literacy and numeracy<br>6.2 Demonstrate strong communication skills<br>6.2.1 Practice cultural awareness

6.1 Demonstrate generic skills (e.g., literacy, numeracy, technology)	»
6.1.1 Exhibit high understanding of literacy and numeracy	» Speak clearly and confidently, organize information in a logical manner, and consider voice tone and pace<br>» Respond to verbal messages and other cues (active listening)<br>» Express ideas and opinions clearly in written and verbal form<br>» Communicate information accurately, concisely and confidently in writing and verbally<br>» Practice a level of mathematics suitable to the job held<br>» Compare visual presentation techniques, including charting, histograms and flow sheets<br>» Read and comprehend at a level necessary to properly complete duties of the position<br>» Understand the need to interpret written information in documents, such as reports, SOPs, LMIS forms, graphs, calendars, schedules, notices and directions<br>» Understand the need to pay attention to detail and identify the main ideas, detect inconsistencies and identify missing information in documents<br>» Enable computer skills — such as using Word, Excel, PowerPoint and the internet — suitable to the job level<br>» Understand the process of analysing and validating KPIs<br>» Understand how the evaluation process of activities that act as standards against which the KPIs are measured is carried out<br>» Examine the process of performing critical self-review on work before submission
6.2 Demonstrate strong communication skills	» Be truthful and trustworthy, and supply accurate information at all times<br>» Enable working as part of a workplace team<br>» Understand vocabulary and communication style and form for both written and verbal communication that is appropriate for the situation, audience and material being communicated (e.g., avoid unnecessary jargon, clearly explain medical and SCM terminology)<br>» Enable open communication within constraints of confidentiality<br>» Participate in the organisation of communications about logistics<br>» Enable participation in meetings, expressing one’s opinions, being aware of others’ needs, and being appropriately assertive when required<br>» Show perspective into the point of view of others, understanding their needs and goals<br>» Enable the practise of building trust, rapport and credibility with others<br>» Understand the communication needs and concerns of others and respond to them<br>» Understand the need to avoid conflict between work and personal interests

- Enable meaningful two-way communication
- Show influence on others by persuasively presenting thoughts and ideas
- Show assertiveness skills to deal with unreasonable requests and/or refusals that would compromise practice or consumer care
- Maintain a positive, supportive and appreciative attitude
- Show the ability to balance the organisation's needs and the customer's needs
- Show self-control by maintaining composure and keeping emotions in check, even in difficult situations; deal calmly and effectively with stressful situations
- Enable expressing of opinions and providing information in written and/or verbal form in a manner that does not elicit concern, anger or other adverse response
- Enable the use of a systematic process for following up that demonstrates written reports have been received and understood
- Understand the means by which responses to input to the work environment are monitored
- Seek practical ways to overcome barriers to communication
- Understand the need to ensuring language representation (e.g., interpreters) at meetings when appropriate
- Interpret difficult situations and disputes between staff that need to be tackled and resolved
- Enable giving both positive and negative feedback sensitively
- Understand the need to maintain and improve communication
- Examine the process of giving and requesting feedback frequently
- Examine effective communication with shop stewards and union representatives

6.2.1 Practice cultural awareness

- Understand the need for cultural awareness and sensitivity, treating all people with fairness, respect and dignity
- Enable anti-discriminatory practices in the organisation, including HR and disciplinary procedures
- Enable cultural sensitivity, equality and fairness at all levels of the organisation
- Challenge discriminatory behaviour directly and sensitively
- Act in a non-discriminatory way toward individuals and groups
- Enable integrating cultural awareness in learning and development approaches
- Avoid stereotypical responses by examining one's own behaviour and bias
- Enable managing cultural diversity in teams and make the most of differences
- Compare strategies and/or resources for communicating effectively with people from different cultural backgrounds
- Enable the process to elicit information relating to values, beliefs and cultural backgrounds of consumers that may influence the way professional services are provided

Basal Technology Competency(ies):	7.4 Temperature and monitoring<br>7.6 ERP includes function of LMIS<br>7.12 Basic Office Skills<br>7.13 Have a command of technology
**7.4 Temperature and monitoring**	» Compare the current technology approaches for Temperature monitoring and sensors<br>» Consider what is required to implement this technology in a country context<br>» Compare infrastructure requirement for heat-sensitive items at peripheral 'lower level' of SC depending on volume and nature of items to handle;<br>» Examine regular preventive maintenance and calibration of cold-chain equipment with biomedical engineers or logisticians<br>» Understand the policy for temperature monitoring tools during transportation and storage<br>» Understand the steps involved in health logistic management cycle: selection-order-delivery-reception-storage-distribution-inventory<br>» Understand hardware qualification and system validation of temperature monitoring<br>» Understand mass data management of temperature monitoring
**7.6 ERP includes function of LMIS**	» Enable a Master Data Management System<br>» Understand the critical components of an ERP system<br>» Understand the meaning of common document types found within an ERP/LMIS<br>» Understand the logic of data visibility within an ERP<br>» Understand the importance of data integrity, record management and information security
**7.12 Basic Office Skills**	» Have a good understanding of common presentation authoring packages<br>» Have a good understanding of common spreadsheet authoring packages<br>» Have a good understanding of common document authoring packages<br>» Have a good understanding of common email authoring packages<br>» Have a good understanding of common instant messaging packages<br>» Have a good understanding of common video conferencing authoring packages
**7.13 Have a command of technology**	» Enable the use of technology suitable to the job held<br>» Understand the need of monitoring new developments and technologies in the sector<br>» Compare new technologies and recognising potential benefits for the sector<br>» Understand that resources and support are provided across the organisation to enable colleagues to make the best use of available technology

	» Understand that the organisation has a strategy for technology use<br>» Compare how to minimise environmental damage through technology use<br>» Enable the use of technology to maximise effectiveness and efficiency<br>» Enable the use of field-based technology (e.g., radio, general packet radio service (GPRS), satellite phone)
Advanced Technology Competency(ies):	7.2 Blockchain<br>7.3 Unmanned Aerial Vehicles (UAVs)<br>7.7 Automation<br>7.9 Additive Manufacturing<br>7.11 Cloud Computing<br>7.14 eProcurement
7.2 Blockchain	» Examine blockchain as a value-add capability for relevant global health use cases
7.3 Unmanned Aerial Vehicles (UAVs)	» Understand the current UAV landscape, their application and the enabling environment required to support country implementation<br>» Understand UAVs as a value-add capability for relevant global health use cases<br>» Understand the laws/ regulations around the use of UAV’s
7.7 Automation	» Understand automation as a value-add capability for relevant global health use cases
7.9 Additive Manufacturing	» Understand additive manufacturing as a value-add capability for relevant global health use cases
7.11 Cloud Computing	» Understand cloud computing as a value-add capability for relevant global health use cases
7.14 eProcurement	» Understand how to transact with an eProcurement system where necessary<br>» Understand eProcurement as a value-add capability for relevant global health use cases<br>» Understand the benefits and limitations of eProcurement to best utilise the system

Key Performance Indicators:
» Vendor on time delivery (VOTD)<br>» % of emergency orders issued in the last 12 months<br>» % of product selection based on National Essential Medicines List<br>» % of procured products that meet Stringent Regulatory Authority (SRA) or WHO standards<br>» Reports submitted on time (for all and separate reports) in accordance with business rules (reporting calendar)<br>» Supply adequacy - Net requirements are reflected in the Procurement pipeline to secure sufficient future supply.<br>» Supply Plan report; supplier performance report<br>» Forecasting and Supply Planning done within the specified cycle time to support the Supply Chain performance requirements.<br>» Data accuracy - Vendor Master data accuracy, item / SKU Master Data accuracy<br>» Establish Product Demand and Resources Cycle Time and recorded.in the "ERP" system against the products and resources.<br>» Establish Supply Plan Cycle Times and recorded in the "ERP" system.<br>» Fill Rate vs Planned fill rate<br>» Forecast accuracy, measured and reported on.<br>» Identify, Prioritize, and Aggregate Supply Chain Requirements Cycle Time - Actual Time taken to determine net supply chain requirements by product and SKU vs Planned Time<br>» Inventory Days of Supply - Actual inventory - Planned Demand over the planning horizon divided by the average daily usage<br>» Material and Resource Plan valid. Number of valid actions performed versus number of actions performed. (Exception messages trigger due to invalid schedules)<br>» Order accuracy - Net requirement versus order quantities in the planning horizon<br>» Procurement planning Cycle Time, by product and resource. Actual Overall planning cycle versus required Overall planning cycle time to achieve Supply Chain performance requirements.<br>» Ratio between median price of pro- ducts procured and the international median reference value<br>» Supply Chain Planning Cost recorded and measured against required Supply China Planning Cycle times to accomplish Supply Chain Strategic objectives.<br>» Availability - Stock out rate - Number of missed line item quantity deliveries due to stock out versus total line item quantity deliveries<br>» Availability - Stock levels - Current on hand stock versus Planned on hand stock

Training:	Qualifications Available:	Certifications Available:
» Risk and exception management<br>» Import & export regulations for products<br>» Inventory management, Min-Max Replenishment<br>» ABC/VEN Inventory Classification		» CIPS Level 3 Advanced Certificate in Procurement and Supply Operations<br>» Manage Logistics Operations skills programme

Lead - Returns Management

Organisation:

SC-focused Organisation

Competency Level:	Serial Number:	Primary Process:	Job Role:	Supervises:
Operational	2-22-351-352-3	Return	Lead - Returns Management	-

Contributions	Manages the return to supplier process. Liaises with suppliers when returns are required to be disposed of through the supplier or if information about the product needs to be communicated backwards along the supply chain.
Technical Competency(ies):	2.2 Build and maintain supplier relationships<br>3.5 Manage disposal of products (e.g. expired, damaged, redundant products)<br>3.5.1 Define and direct process for managing redundant and returned stock<br>3.5.2 Manage process for disposal of returned stock

2.2 Build and maintain supplier relationships

- Describe factors in the analysis of the market
- Use and monitor the processes for prequalification and tender contracting
- Define of a good supplier
- Describe the process of locating and sourcing key suppliers, keeping in mind the total cost associated with procuring an item or service
- Describe supplier relationship management plans (e.g., sourcing, frequency of meetings, negotiate and monitor benchmarks for performance)
- Educate suppliers to create value for customers by streamlining processes in the value chain
- Explain the difference between strategic and non-strategic suppliers and the associated supplier management principles
- Describe commercial factors that contribute toward cost reduction, price savings and value improvement opportunities
- Ensure the strategic sourcing practices drive down cost across consecutive tenders
- Work with suppliers to agree cost saving opportunities in the whole value chain, with an appropriate cost sharing mechanism applied to ensure a “win-win” outcome
- Manage supplier risk
- Apply ethical sourcing

3.5 Manage disposal of products (e.g. expired, damaged, redundant products)	
3.5.1 Define and direct process for managing redundant and returned stock	» Confirm information on the supplies being returned<br>» Describe the flow of returned supplies<br>» Identify any problems with the flow of returned supplies<br>» Integrate options for improving the flow of returned supplies<br>» Apply procedures for monitoring the flow of returned supplies<br>» Describe and/or demonstrate recall procedures to be used in response to a product recall notice or to access the information promptly.<br>» Identify issues relevant to the urgency and scope of action required in response to a product recall notice<br>» Identify the information required for reporting and procedures with regards to following up on medication incidents<br>» Identify follow-up strategies likely to be effective in preventing recurrence (e.g., root cause analysis)<br>» Describe an integrated healthcare waste management plan<br>» Describe guides and procedures for collection, sorting, transport and disposal<br>» Conduct pre-disposal inspections<br>» Describe the prerequisites for products disposal<br>» Generate product disposal report
3.5.2 Manage process for disposal of returned stock	» Dispose of expired medications and/or medical equipment according to national policy<br>» Describe and/or use an appropriate recording system for disposal<br>» Describe the disposal of specific individual items of greatest risk with appropriate care (e.g., oncology medicine)
Personal/Management Competency(ies):	6.1 Demonstrate generic skills (e.g., literacy, numeracy, technology)<br>6.1.1 Exhibit high understanding of literacy and numeracy<br>6.2 Demonstrate strong communication skills<br>6.2.1 Practice cultural awareness
6.1 Demonstrate generic skills (e.g., literacy, numeracy, technology)	
6.1.1 Exhibit high understanding of literacy and numeracy	» Speak clearly and confidently, organize information in a logical manner, and consider voice tone and pace<br>» Respond to verbal messages and other cues (active listening)<br>» Express ideas and opinions clearly in written and verbal form<br>» Communicate information accurately, concisely and confidently in writing and verbally<br>» Practice a level of mathematics suitable to the job held

	» Describe visual presentation techniques, including charting, histograms and flow sheets<br>» Read and comprehend at a level necessary to properly complete duties of the position<br>» Interpret written information in documents, such as reports, SOPs, LMIS forms, graphs, calendars, schedules, notices and directions<br>» Pay attention to detail and identify the main ideas, detect inconsistencies and identify missing information in documents<br>» Apply computer skills — such as using Word, Excel, PowerPoint and the internet — suitable to the job level<br>» Describe the process of analysing and validating KPIs<br>» Participate in the evaluation of activities that act as standards against which the KPIs are measured<br>» Apply the process of performing critical self-review on work before submission
6.2 Demonstrate strong communication skills	» Be truthful and trustworthy, and supply accurate information at all times<br>» Work as part of a workplace team<br>» Describe vocabulary and communication style and form for both written and verbal communication that is appropriate for the situation, audience and material being communicated (e.g., avoid unnecessary jargon, clearly explain medical and SCM terminology)<br>» Encourage open communication within constraints of confidentiality<br>» Organize communications about logistics<br>» Encourage participation in meetings, expressing one’s opinions, being aware of others’ needs, and being appropriately assertive when required<br>» Apply perspective into the point of view of others, understanding their needs and goals<br>» Encourage building trust, rapport and credibility with others<br>» Anticipate the communication needs and concerns of others and respond to them<br>» Avoid conflict between work and personal interests<br>» Encourage meaningful two-way communication<br>» Influence others by persuasively presenting thoughts and ideas<br>» Apply assertiveness skills to deal with unreasonable requests and/or refusals that would compromise practice or consumer care<br>» Maintain a positive, supportive and appreciative attitude<br>» Apply the ability to balance the organisation’s needs and the customer’s needs<br>» Use self-control by maintaining composure and keeping emotions in check, even in difficult situations; deal calmly and effectively with stressful situations<br>» Encourage the expressing of opinions and providing information in written and/or verbal form in a manner that does not elicit concern, anger or other adverse response

- Apply the use of a systematic process for following up that demonstrates written reports have been received and understood
- Describe the means by which responses to input to the work environment are monitored
- Seek practical ways to overcome barriers to communication
- Identify language representation (e.g., interpreters) at meetings when appropriate
- Tackle difficult situations and resolve disputes between staff
- Give both positive and negative feedback sensitively
- Maintain and improve communication
- Apply the process of giving and requesting feedback frequently
- Apply the ability to communicate effectively with shop stewards and union representatives

6.2.1 Practice cultural awareness

- Describe cultural awareness and sensitivity, treating all people with fairness, respect and dignity
- Describe anti-discriminatory practices in the organisation, including HR and disciplinary procedures
- Establish cultural sensitivity, equality and fairness at all levels of the organisation
- Challenge discriminatory behaviour directly and sensitively
- Act in a non-discriminatory way toward individuals and groups
- Integrate cultural awareness in learning and development approaches
- Avoid stereotypical responses by examining one's own behaviour and bias
- Manage cultural diversity in teams and make the most of differences
- Describe strategies and/or resources for communicating effectively with people from different cultural backgrounds
- Elicit information relating to values, beliefs and cultural backgrounds of consumers that may influence the way professional services are provided

Basal Technology Competency(ies):

7.6 ERP includes function of LMIS

7.12 Basic Office Skills

7.13 Have a command of technology

7.6 ERP includes function of LMIS

- Support a Master Data Management System
- Describe the critical components of an ERP system
- Describe the meaning of common document types found within an ERP/LMIS
- Describe the logic of data visibility within an ERP
- Describe the importance of data integrity, record management and information security

7.12 Basic Office Skills	» Have a good understanding of common presentation authoring packages<br>» Have a good understanding of common spreadsheet authoring packages<br>» Have a good understanding of common document authoring packages<br>» Have a good understanding of common email authoring packages<br>» Have a good understanding of common instant messaging packages<br>» Have a good understanding of common video conferencing authoring packages
7.13 Have a command of technology	» Apply the use of technology suitable to the job held<br>» Provide input on monitoring new developments and technologies in the sector<br>» Identify new technologies and recognising potential benefits for the sector<br>» Identify resources and support that are provided across the organisation to enable colleagues to make the best use of available technology<br>» Ensure that the organisation has a strategy for technology use<br>» Describe how to minimise environmental damage through technology use<br>» Use technology to maximise effectiveness and efficiency<br>» Use field-based technology (e.g., radio, general packet radio service (GPRS), satellite phone)

Advanced Technology Competency(ies):	7.7 Automation
7.7 Automation	» Describe automation as a value-add capability for relevant global health use cases

Key Performance Indicators:

» Safe product handling - Number of products handled during period out of conformance versus Number of products handled during period expressed as a % with prior period comparisons

» Customer Incidents Report number of incidents by month/week/day comparison with previous periods

» No of Order Lines shipped with incorrectly captured Items - Number of order lines with incorrect items/SKU's versus total number of order lines shipped as a %

» Regulatory - % of procured products that meet Stringent Regulatory Authority (SRA) or WHO standards

» Waste Management - Existence of supply chain management protocols for disposal of medical waste and management of unusable products

» Number of Order Lines delivered to incorrect end-user - Number of orders incorrectly addressed versus number of orders processed expressed as a %

» Number of Order Lines delivered out of Quality Specifications - Number of Item/SKU delivered out of specification e.g. Cold Chain infractions, Expired products versus total Number of order lines delivered as a %. A comparative graph would be an appropriate tool for the measurement of this metric.

Training:	Qualifications Available:	Certifications Available:
» SC data analysis<br>» ERP Systems<br>» Inventory Management<br>» Regulatory Policy Management<br>» Logistics Management		» CIPS Level 4 Diploma in Procurement and Supply<br>» APICS Principles of operations management series certificate<br>» Logistics and Supply Chain Management MIT

Officer - Logistics Planning

Organisation:

SC-focused Organisation

Competency Level:	Serial Number:	Primary Process:	Job Role:	Supervises:
Operational	3-34-15-14-2	Plan – Deliver	Officer - Logistics Planning	-

Contributions	Assists the Manager - Logistics in balancing demand with capacity of logistics systems. Provides input to the total supply chain plan
Technical Competency(ies):	1.4 Forecast and quantify product needs<br>1.5 Supply planning<br>3.4 Manage transport for commodities<br>3.4.1 Manage transport for commodities during disaster

1.4 Forecast and quantify product needs

- Examine the factors that affect usage patterns of medications and equipment and how this affects ordering (e.g., disease outbreaks), using national policies as a guide and to ensure consistent application
- Understand principles and applications of demand forecasting
- Compare the critical requirements for effective forecasting: establishing time horizons, level of detail and use of data
- Understand policies and procedures for forecasting
- Understand the process of quantifying product requirements using a variety of methods
- Examine consumption data at the national level for various country programmes
- Compare and reconcile different types of forecasts
- Understand the process of applying VEN or ABC analysis to programme requirements for national level procurement
- Understand the importance of calculating average monthly dispensed-to-user quantities for all service delivery points nationally
- Understand the importance of calculating storage space requirements for all levels in the supply chain
- Understand the importance of calculating the months of supply on hand for each commodity at the national level
- Examine the maximum and minimum stock levels for each level in a programme
- Understand various tools used in quantification (e.g., quantification software)
- Understand the use of various data sources for commodity forecasting (e.g., consumption data, services data, demographic data)
- Compare key performance indicators of forecast accuracy

	» Examine the degree of error when using forecasting methods
1.5 Supply planning	» Examine a purchasing and supply plan in line with national priorities<br>» Understand the importance of balancing of supply chain resources and trigger activities to correct any imbalances<br>» Understand the formalisation of sales and operations plans through sales and operations meeting.<br>» Understand that the sales and operations plan must be translated into executable plans to ensure dispatch of goods in line with overall priorities.<br>» Understand the national commodity pipeline
3.4 Manage transport for commodities	» Understand distribution activities, including a fleet of vehicles and distribution schedules, to deliver health commodities to facilities.<br>» Understand the policies and procedures related to handling and transporting special products such as vaccines, HIV/AIDS drugs, other high value products and narcotics<br>» Understands shipping methods, considering trade-offs between costs and benefits<br>» Understand local and national laws/requirements for vehicle safety, driving regulations and licensing<br>» Understand proper packing and labelling methods for transportation of hazardous materials<br>» Understand the process of maximizing freight loads while minimizing freight costs<br>» Enable efficient use of transportation resources while meeting customers' needs<br>» Examine movement demands with vehicle resources<br>» Understand quantitative techniques when solving logistics problems, such as designing routes and scheduling vehicles<br>» Understand the process of delivering products to hospitals and district health offices with the accompanying dispatch note and report and requisition forms<br>» Coordinate transport options for order delivery<br>» Understand that vehicle availability is due to a vehicle maintenance plan<br>» Interpret information on distribution requirements<br>» Examine any problems in distribution requirements<br>» Enable the process of equipping distribution sites<br>» Understand the need to inform stakeholders for better coordination of distribution<br>» Compare tracking devices (tools and indicators)<br>» Examine the process of training of drivers, van assistants, fleet administrators and expeditors<br>» Examine the process of supporting investigations around missed OTIF and other failures

3.4.1 Manage transport for commodities during disaster	» Be able to compare of the different types of transport and their characteristics for emergency or disaster supply<br>» Understand the process of developing a plan for the achievement of a rapid response logistics plan for deployment in the event of a medical emergency.
Personal/Management Competency(ies):	6.1 Demonstrate generic skills (e.g., literacy, numeracy, technology)<br>6.1.1 Exhibit high understanding of literacy and numeracy<br>6.2 Demonstrate strong communication skills<br>6.2.1 Practice cultural awareness
6.1 Demonstrate generic skills (e.g., literacy, numeracy, technology)	
6.1.1 Exhibit high understanding of literacy and numeracy	» Speak clearly and confidently, organize information in a logical manner, and consider voice tone and pace<br>» Respond to verbal messages and other cues (active listening)<br>» Express ideas and opinions clearly in written and verbal form<br>» Communicate information accurately, concisely and confidently in writing and verbally<br>» Practice a level of mathematics suitable to the job held<br>» Compare visual presentation techniques, including charting, histograms and flow sheets<br>» Read and comprehend at a level necessary to properly complete duties of the position<br>» Understand the need to interpret written information in documents, such as reports, SOPs, LMIS forms, graphs, calendars, schedules, notices and directions<br>» Understand the need to pay attention to detail and identify the main ideas, detect inconsistencies and identify missing information in documents<br>» Enable computer skills — such as using Word, Excel, PowerPoint and the internet — suitable to the job level<br>» Understand the process of analysing and validating KPIs<br>» Understand how the evaluation process of activities that act as standards against which the KPIs are measured is carried out<br>» Examine the process of performing critical self-review on work before submission
6.2 Demonstrate strong communication skills	» Be truthful and trustworthy, and supply accurate information at all times<br>» Enable working as part of a workplace team<br>» Understand vocabulary and communication style and form for both written and verbal communication that is appropriate for the situation, audience and material being communicated (e.g., avoid unnecessary jargon, clearly explain medical and SCM terminology)<br>» Enable open communication within constraints of confidentiality

» Participate in the organisation of communications about logistics
» Enable participation in meetings, expressing one's opinions, being aware of others' needs, and being appropriately assertive when required
» Show perspective into the point of view of others, understanding their needs and goals
» Enable the practise of building trust, rapport and credibility with others
» Understand the communication needs and concerns of others and respond to them
» Understand the need to avoid conflict between work and personal interests
» Enable meaningful two-way communication
» Show influence on others by persuasively presenting thoughts and ideas
» Show assertiveness skills to deal with unreasonable requests and/or refusals that would compromise practice or consumer care
» Maintain a positive, supportive and appreciative attitude
» Show the ability to balance the organisation's needs and the customer's needs
» Show self-control by maintaining composure and keeping emotions in check, even in difficult situations; deal calmly and effectively with stressful situations
» Enable expressing of opinions and providing information in written and/or verbal form in a manner that does not elicit concern, anger or other adverse response
» Enable the use of a systematic process for following up that demonstrates written reports have been received and understood
» Understand the means by which responses to input to the work environment are monitored
» Seek practical ways to overcome barriers to communication
» Understand the need to ensuring language representation (e.g., interpreters) at meetings when appropriate
» Interpret difficult situations and disputes between staff that need to be tackled and resolved
» Enable giving both positive and negative feedback sensitively
» Understand the need to maintain and improve communication
» Examine the process of giving and requesting feedback frequently
» Examine effective communication with shop stewards and union representatives

6.2.1 Practice cultural awareness

» Understand the need for cultural awareness and sensitivity, treating all people with fairness, respect and dignity
» Enable anti-discriminatory practices in the organisation, including HR and disciplinary procedures
» Enable cultural sensitivity, equality and fairness at all levels of the organisation

	» Challenge discriminatory behaviour directly and sensitively<br>» Act in a non-discriminatory way toward individuals and groups<br>» Enable integrating cultural awareness in learning and development approaches<br>» Avoid stereotypical responses by examining one's own behaviour and bias<br>» Enable managing cultural diversity in teams and make the most of differences<br>» Compare strategies and/or resources for communicating effectively with people from different cultural backgrounds<br>» Enable the process to elicit information relating to values, beliefs and cultural backgrounds of consumers that may influence the way professional services are provided
Basal Technology Competency(ies):	7.4 Temperature and monitoring<br>7.6 ERP includes function of LMIS<br>7.12 Basic Office Skills<br>7.13 Have a command of technology
7.4 Temperature and monitoring	» Compare the current technology approaches for Temperature monitoring and sensors<br>» Consider what is required to implement this technology in a country context<br>» Compare infrastructure requirement for heat-sensitive items at peripheral 'lower level' of SC depending on volume and nature of items to handle;<br>» Examine regular preventive maintenance and calibration of cold-chain equipment with biomedical engineers or logisticians<br>» Understand the policy for temperature monitoring tools during transportation and storage<br>» Understand the steps involved in health logistic management cycle: selection-order-delivery-reception-storage-distribution-inventory<br>» Understand hardware qualification and system validation of temperature monitoring<br>» Understand mass data management of temperature monitoring
7.6 ERP includes function of LMIS	» Enable a Master Data Management System<br>» Understand the critical components of an ERP system<br>» Understand the meaning of common document types found within an ERP/LMIS<br>» Understand the logic of data visibility within an ERP<br>» Understand the importance of data integrity, record management and information security

7.12 Basic Office Skills	» Have a good understanding of common presentation authoring packages<br>» Have a good understanding of common spreadsheet authoring packages<br>» Have a good understanding of common document authoring packages<br>» Have a good understanding of common email authoring packages<br>» Have a good understanding of common instant messaging packages<br>» Have a good understanding of common video conferencing authoring packages
7.13 Have a command of technology	» Enable the use of technology suitable to the job held<br>» Understand the need of monitoring new developments and technologies in the sector<br>» Compare new technologies and recognising potential benefits for the sector<br>» Understand that resources and support are provided across the organisation to enable colleagues to make the best use of available technology<br>» Understand that the organisation has a strategy for technology use<br>» Compare how to minimise environmental damage through technology use<br>» Enable the use of technology to maximise effectiveness and efficiency<br>» Enable the use of field-based technology (e.g., radio, general packet radio service (GPRS), satellite phone)
Advanced Technology Competency(ies):	7.2 Blockchain<br>7.3 Unmanned Aerial Vehicles (UAVs)<br>7.7 Automation<br>7.9 Additive Manufacturing<br>7.11 Cloud Computing<br>7.14 eProcurement
7.2 Blockchain	» Examine blockchain as a value-add capability for relevant global health use cases
7.3 Unmanned Aerial Vehicles (UAVs)	» Understand the current UAV landscape, their application and the enabling environment required to support country implementation<br>» Understand UAVs as a value-add capability for relevant global health use cases<br>» Understand the laws/ regulations around the use of UAV's

7.7 Automation	» Understand automation as a value-add capability for relevant global health use cases
7.9 Additive Manufacturing	» Understand additive manufacturing as a value-add capability for relevant global health use cases
7.11 Cloud Computing	» Understand cloud computing as a value-add capability for relevant global health use cases
7.14 eProcurement	» Understand how to transact with an eProcurement system where necessary<br>» Understand eProcurement as a value-add capability for relevant global health use cases<br>» Understand the benefits and limitations of eProcurement to best utilise the system

Key Performance Indicators:

- %Cubic Metres delivered vs Cubic Metres planned by Location delivered
- Cost per Cubic Metres delivered vs Cost per Cubic Metres planned by Location delivered
- Transportation Lead time planned versus Actual Transportation Lead Time
- Warehouse Utilization - Volume used vs Volume Available
- Line Items SKU's handled vs Items / SKU's Planned over a planning horizon
- Route efficiency - Actual Routing costs versus Standard / Planned Routing costs
- On-time and In-Full (OTIF (assume same as %Cubic Metres delivered vs Cubic Metres planned)
- Zero delivery failures due to non-compliance (broken cold chain, contamination, wet stock, broken seals, side curtain vehicles, co-loading with other products, spills etc)
- Pharmaceutical product not staying over at unlicensed TSP for longer than 48 hours
- Cold Chain Maintained

Training:	Qualifications Available:	Certifications Available:
» Risk and exception management<br>» Inventory management, Min-Max Replenishment<br>» ABC/VEN Inventory Classification<br>» Transport Management<br>» Inventory planning certification		» Manage Logistics Operations skills programme<br>» Apply fundamental concepts and principles of supply chain management skills programme<br>» APICS Certified in Logistics, Transportation and Distribution (CLTD)

» Good Distribution Practice training		

Specialist – Contracts

Organisation:

SC-focused Organisation

Competency Level:	Serial Number:	Primary Process:	Job Role:	Supervises:
**Operational**	**2-242-23-2**	**Enable – Contracts Agreements**	**Specialist – Contracts**	**-**

Contributions	Adheres to the Enable processes for specified contract types as prescribed in the Public Health System Organisation's Operating Model. Maintains contract and agreement records current and relevant and utilizes appropriate governance methodology to each contract or agreement of a specified type.
Technical Competency(ies):	2.3 Manage tendering processes and supplier agreements<br>2.4 Undertake contract management and risk<br>2.4.1 Place commodity orders<br>2.4.2 Manage contracts<br>2.4.3 Addresses risk and ensures quality management

2.3 Manage tendering processes and supplier agreements

- Understand the use of request for qualifications (RFQ), invitations to bid (ITB) and request for proposals (RFP) methods and when to choose which method
- Understand the local and international tendering procedures (e.g., open competitive bidding, restricted tender, competitive negotiation, direct procurement)
- Understand the detailed specifications for tenders
- Understand the need for high-level guidance for high-value and politically sensitive procurements
- Understand the process of developing and managing contracts
- Understand the procurement, return and exchange policies
- Understand appropriate legislation and policy in regard to procurement processes, and what is required to comply
- Understand what constitutes ethical behaviour, abiding by conflict of interest policies
- Understand the process of evaluating tender bids based on the specification and evaluation criteria including capability of the supplier, problems relating to outcomes of the contract.
- Understand that there is a contract approval process, including contract negotiations
- Understand some of the key issues relevant to negotiating supply contracts (e.g., volume usage over time, price volume agreements,

	alternate supplier clauses, duration of contract, period of review, terms for contract termination and renewal)<br>» Understand the process of awarding and managing contracts, resolving any issues as they arise<br>» Understand the process of debriefing unsuccessful suppliers<br>» Understand the ways in which compliance with purchasing policies and procedures is monitored<br>» Understand the process of procurement planning including a yearly activities schedule and developing a wide range of briefs, specifications and commercial documentation<br>» Examine strategic sourcing projects, capturing benefits, work requirements, impacts and cost savings<br>» Provide input to cross-functional teams handling strategic procurements<br>» Compare internal and external procurement networks<br>» Compare local and international health commodity prices and understand the factors affecting them<br>» Understand the process of engaging clients across the public sector in presentations demonstrating the benefits of a range of strategic projects designed to ensure cost savings and value for money for government procurement
2.4 Undertake contract management and risk	
2.4.1 Place commodity orders	» Understand that authorisations are required to administer the contracts<br>» Understand the type of supplies required<br>» Understand the process of obtaining reference sources and supplier catalogues to clarify required product and its availability<br>» Understand the process to comply with policies and procedures to order required stock and equipment<br>» Understand the process of confirming the orders with suppliers<br>» Understand the process of identifying any problems in placing orders<br>» Understand the procedures for placing orders<br>» Examine any problems with order delivery and compare recommended options for progressing order delivery<br>» Enable the execution of effective mechanisms for checking invoices exist and take action to adjust payments accordingly<br>» Understand the process of receiving or raising requisitions and arranging purchase orders and payments
2.4.2 Manage contracts	» Understand the process of conducting reviews of performance against agreed key performance indicators<br>» Understand the need for providing timely and expert guidance to remediate procurement performance issues<br>» Compare contract responsiveness and negotiating changes to a contract (contract variations)<br>» Understand the process of managing contract disputes and terminating contracts legally and appropriately if necessary

	» Understand the importance of supplier control mechanisms, contract administration and supplier management
2.4.3 Addresses risk and ensures quality management	» Understand the risk assessments for the procurement process (technical, commercial, administrative)<br>» Understand the key performance indicators for assessing procurement and supplier performance and effectiveness<br>» Examine any problems with the procurement of supplies in the supply chain<br>» Understand the contingency plan for shortages<br>» Compare recommended options for improving the performance of suppliers<br>» Understand procurement portfolio analysis and risk assessment (supply positioning)<br>» Understand the fundamentals of risk planning and assessment
Personal/Management Competency(ies):	6.1 Demonstrate generic skills (e.g., literacy, numeracy, technology)<br>6.1.1 Exhibit high understanding of literacy and numeracy<br>6.2 Demonstrate strong communication skills<br>6.2.1 Practice cultural awareness
6.1 Demonstrate generic skills (e.g., literacy, numeracy, technology)	
6.1.1 Exhibit high understanding of literacy and numeracy	» Speak clearly and confidently, organize information in a logical manner, and consider voice tone and pace<br>» Respond to verbal messages and other cues (active listening)<br>» Express ideas and opinions clearly in written and verbal form<br>» Communicate information accurately, concisely and confidently in writing and verbally<br>» Practice a level of mathematics suitable to the job held<br>» Compare visual presentation techniques, including charting, histograms and flow sheets<br>» Read and comprehend at a level necessary to properly complete duties of the position<br>» Understand the need to interpret written information in documents, such as reports, SOPs, LMIS forms, graphs, calendars, schedules, notices and directions<br>» Understand the need to pay attention to detail and identify the main ideas, detect inconsistencies and identify missing information in documents<br>» Enable computer skills — such as using Word, Excel, PowerPoint and the internet — suitable to the job level<br>» Understand the process of analysing and validating KPIs<br>» Understand how the evaluation process of activities that act as standards against which the KPIs are measured is carried out

- Examine the process of performing critical self-review on work before submission

6.2 Demonstrate strong communication skills	» Be truthful and trustworthy, and supply accurate information at all times<br>» Enable working as part of a workplace team<br>» Understand vocabulary and communication style and form for both written and verbal communication that is appropriate for the situation, audience and material being communicated (e.g., avoid unnecessary jargon, clearly explain medical and SCM terminology)<br>» Enable open communication within constraints of confidentiality<br>» Participate in the organisation of communications about logistics<br>» Enable participation in meetings, expressing one's opinions, being aware of others' needs, and being appropriately assertive when required<br>» Show perspective into the point of view of others, understanding their needs and goals<br>» Enable the practise of building trust, rapport and credibility with others<br>» Understand the communication needs and concerns of others and respond to them<br>» Understand the need to avoid conflict between work and personal interests<br>» Enable meaningful two-way communication<br>» Show influence on others by persuasively presenting thoughts and ideas<br>» Show assertiveness skills to deal with unreasonable requests and/or refusals that would compromise practice or consumer care<br>» Maintain a positive, supportive and appreciative attitude<br>» Show the ability to balance the organisation's needs and the customer's needs<br>» Show self-control by maintaining composure and keeping emotions in check, even in difficult situations; deal calmly and effectively with stressful situations<br>» Enable expressing of opinions and providing information in written and/or verbal form in a manner that does not elicit concern, anger or other adverse response<br>» Enable the use of a systematic process for following up that demonstrates written reports have been received and understood<br>» Understand the means by which responses to input to the work environment are monitored<br>» Seek practical ways to overcome barriers to communication<br>» Understand the need to ensuring language representation (e.g., interpreters) at meetings when appropriate<br>» Interpret difficult situations and disputes between staff that need to be tackled and resolved<br>» Enable giving both positive and negative feedback sensitively<br>» Understand the need to maintain and improve communication<br>» Examine the process of giving and requesting feedback frequently

	» Examine effective communication with shop stewards and union representatives
6.2.1 Practice cultural awareness	» Understand the need for cultural awareness and sensitivity, treating all people with fairness, respect and dignity<br>» Enable anti-discriminatory practices in the organisation, including HR and disciplinary procedures<br>» Enable cultural sensitivity, equality and fairness at all levels of the organisation<br>» Challenge discriminatory behaviour directly and sensitively<br>» Act in a non-discriminatory way toward individuals and groups<br>» Enable integrating cultural awareness in learning and development approaches<br>» Avoid stereotypical responses by examining one's own behaviour and bias<br>» Enable managing cultural diversity in teams and make the most of differences<br>» Compare strategies and/or resources for communicating effectively with people from different cultural backgrounds<br>» Enable the process to elicit information relating to values, beliefs and cultural backgrounds of consumers that may influence the way professional services are provided
Basal Technology Competency(ies):	7.6 ERP includes function of LMIS<br>7.12 Basic Office Skills<br>7.13 Have a command of technology
7.6 ERP includes function of LMIS	» Enable a Master Data Management System<br>» Understand the critical components of an ERP system<br>» Understand the meaning of common document types found within an ERP/LMIS<br>» Understand the logic of data visibility within an ERP<br>» Understand the importance of data integrity, record management and information security
7.12 Basic Office Skills	» Have a good understanding of common presentation authoring packages<br>» Have a good understanding of common spreadsheet authoring packages<br>» Have a good understanding of common document authoring packages<br>» Have a good understanding of common email authoring packages<br>» Have a good understanding of common instant messaging packages<br>» Have a good understanding of common video conferencing authoring packages
7.13 Have a command of technology	» Enable the use of technology suitable to the job held<br>» Understand the need of monitoring new developments and technologies in the sector

	» Compare new technologies and recognising potential benefits for the sector<br>» Understand that resources and support are provided across the organisation to enable colleagues to make the best use of available technology<br>» Understand that the organisation has a strategy for technology use<br>» Compare how to minimise environmental damage through technology use<br>» Enable the use of technology to maximise effectiveness and efficiency<br>» Enable the use of field-based technology (e.g., radio, general packet radio service (GPRS), satellite phone)
Advanced Technology Competency(ies):	7.2 Blockchain<br>7.11 Cloud Computing
7.2 Blockchain	» Examine blockchain as a value-add capability for relevant global health use cases
7.11 Cloud Computing	» Understand cloud computing as a value-add capability for relevant global health use cases

Key Performance Indicators:

- Accuracy of tender documents
- Quality of Supplier Relationship
- Tenders adjudication and allocation on Time performance
- Tenders allocated in accordance with Business Rules
- Vendor/Supplier Master Data Accuracy
- Item Master Data Accuracy
- inbound Transportation Master Data Accuracy by Item SKU
- Item SKU Lead Time Accuracy
- Tender creation cycle time
- On Time Closure of Tenders
- Risk avoidance, elimination and or Mitigation Costs

Training:	Qualifications Available:	Certifications Available:
» Logistics and procurement<br>» Contract Management<br>» Consignment Agreement Development<br>» Customer/Supplier Communication<br>» International Trade<br>» Procurement		» CIPS Level 3 Advanced Certificate in Procurement and Supply Operations<br>» =-

» Subcontracting Types (FFP, CP, CPAF, Performance Based)<br>» Supplier Relationship Management (SRM)		

Officer – Performance Monitoring

Organisation: **SC-focused Organisation**				
Competency Level:	Serial Number:	Primary Process:	Job Role:	Supervises:
Operational	5-531-532-52-2	Enable – SC Performance	Officer – Performance Monitoring	-

Contributions	Ensures the timely and accurate recording of transactional data prescribed by the Supply Chain Process Metrics at the appropriate transaction points, Creates and maintains the appropriate reporting infrastructure to disseminate the performance metric information in accordance with the Supply Chain RACI's
Personal/Management Competency(ies):	5.3 Implement risk management and monitoring and evaluation activities for the supply chain<br>5.3.1 Ensure monitoring and evaluation activities are completed<br>5.3.2 Implement risk management activities<br>6.1 Demonstrate generic skills (e.g., literacy, numeracy, technology)<br>6.1.1 Exhibit high understanding of literacy and numeracy<br>6.2 Demonstrate strong communication skills<br>6.2.1 Practice cultural awareness

5.3 Implement risk management and monitoring and evaluation activities for the supply chain

5.3.1 Ensure monitoring and evaluation activities are completed

- Understand the periodic evaluations to maintain processes by gathering pertinent information — such as problem symptoms from knowledgeable sources, carrying these through to the problems, potential causes and root causes of the problem
- Understand the process of quantifying the operational performance of similar companies and establish internal targets based on best-in-class results
- Enable key performance measurements and continuous process improvement initiatives to improve process quality on a continual basis
- Understand the need to provide results of the analyses to colleagues
- Understand of the need to control and check errors, taking corrective action so deviation from standards are minimised and the organisation’s goals are achieved
- Examine the use of appropriate technological developments to improve the system
- Understand that continuous process improvement is an accepted way of organisational life
- Examine ways of eliminating unnecessary steps in system design

	» Enable processes that strive to eliminate waste<br>» Enable processes to encourage sustainability (e.g., reducing carbon footprint, establishing a paperless office, using renewable energy)<br>» Understand the systematic approach used to close process or system performance gaps through streamlining and cycle time reduction, and identify and eliminate causes of quality below specifications, process variation and non-value-adding activities<br>» Examine the relationship between technology and process functionality<br>» Understand how policy, guidelines and regulatory reforms impact SC performance
5.3.2 Implement risk management activities	» Examine risks affecting supply, transformation, delivery and customer demand<br>» Understand what constitutes unsafe working conditions and how to take prompt, corrective action; stay alert to and take preventive action against hazards and threats; follow the process of recommending measures to protect employees from hazardous working conditions ; follow protocol for reporting safety violations<br>» Follow training programmes/drills in which personnel participate (e.g., fire and armed hold-up drills, safe manual handling practices, use of mandatory safety clothing)<br>» Enable systems of review (e.g., audit) to check compliance with policies and procedures intended to maintain workplace safety (e.g., manual handling techniques, correct use of protective clothing)<br>» Ensure equipment and tools are operating to prescribed standards<br>» Enable key security systems for the workplace (e.g., for cash, narcotics and other controlled substances, investigational drugs, consumer records, entry and exit points) and levels of access and/or authority applicable to each<br>» Follow the prevention strategies adopted (e.g., protocols, security barriers, fixed or personal duress alarms)<br>» Understand the need to document critical incidents<br>» Understand contingency planning and the need to manage operational continuity<br>» Understand what constitutes a safe working environment and engender a shared commitment from all personnel to creating one<br>» Understand relevant safety and health laws and regulations; comply with safety codes, standards and guidelines; adhere to worksite safety programmes; understand emergency response plans when they exist<br>» Understand how to handle hazardous commodities and first aid procedures
6.1 Demonstrate generic skills (e.g., literacy, numeracy, technology)	
6.1.1 Exhibit high understanding of literacy and numeracy	» Speak clearly and confidently, organize information in a logical manner, and consider voice tone and pace<br>» Respond to verbal messages and other cues (active listening)

	» Express ideas and opinions clearly in written and verbal form<br>» Communicate information accurately, concisely and confidently in writing and verbally<br>» Practice a level of mathematics suitable to the job held<br>» Compare visual presentation techniques, including charting, histograms and flow sheets<br>» Read and comprehend at a level necessary to properly complete duties of the position<br>» Understand the need to interpret written information in documents, such as reports, SOPs, LMIS forms, graphs, calendars, schedules, notices and directions<br>» Understand the need to pay attention to detail and identify the main ideas, detect inconsistencies and identify missing information in documents<br>» Enable computer skills — such as using Word, Excel, PowerPoint and the internet — suitable to the job level<br>» Understand the process of analysing and validating KPIs<br>» Understand how the evaluation process of activities that act as standards against which the KPIs are measured is carried out<br>» Examine the process of performing critical self-review on work before submission
6.2 Demonstrate strong communication skills	» Be truthful and trustworthy, and supply accurate information at all times<br>» Enable working as part of a workplace team<br>» Understand vocabulary and communication style and form for both written and verbal communication that is appropriate for the situation, audience and material being communicated (e.g., avoid unnecessary jargon, clearly explain medical and SCM terminology)<br>» Enable open communication within constraints of confidentiality<br>» Participate in the organisation of communications about logistics<br>» Enable participation in meetings, expressing one's opinions, being aware of others' needs, and being appropriately assertive when required<br>» Show perspective into the point of view of others, understanding their needs and goals<br>» Enable the practise of building trust, rapport and credibility with others<br>» Understand the communication needs and concerns of others and respond to them<br>» Understand the need to avoid conflict between work and personal interests<br>» Enable meaningful two-way communication<br>» Show influence on others by persuasively presenting thoughts and ideas<br>» Show assertiveness skills to deal with unreasonable requests and/or refusals that would compromise practice or consumer care<br>» Maintain a positive, supportive and appreciative attitude<br>» Show the ability to balance the organisation's needs and the customer's needs

	» Show self-control by maintaining composure and keeping emotions in check, even in difficult situations; deal calmly and effectively with stressful situations<br>» Enable expressing of opinions and providing information in written and/or verbal form in a manner that does not elicit concern, anger or other adverse response<br>» Enable the use of a systematic process for following up that demonstrates written reports have been received and understood<br>» Understand the means by which responses to input to the work environment are monitored<br>» Seek practical ways to overcome barriers to communication<br>» Understand the need to ensuring language representation (e.g., interpreters) at meetings when appropriate<br>» Interpret difficult situations and disputes between staff that need to be tackled and resolved<br>» Enable giving both positive and negative feedback sensitively<br>» Understand the need to maintain and improve communication<br>» Examine the process of giving and requesting feedback frequently<br>» Examine effective communication with shop stewards and union representatives
6.2.1 Practice cultural awareness	» Understand the need for cultural awareness and sensitivity, treating all people with fairness, respect and dignity<br>» Enable anti-discriminatory practices in the organisation, including HR and disciplinary procedures<br>» Enable cultural sensitivity, equality and fairness at all levels of the organisation<br>» Challenge discriminatory behaviour directly and sensitively<br>» Act in a non-discriminatory way toward individuals and groups<br>» Enable integrating cultural awareness in learning and development approaches<br>» Avoid stereotypical responses by examining one's own behaviour and bias<br>» Enable managing cultural diversity in teams and make the most of differences<br>» Compare strategies and/or resources for communicating effectively with people from different cultural backgrounds<br>» Enable the process to elicit information relating to values, beliefs and cultural backgrounds of consumers that may influence the way professional services are provided
Basal Technology Competency(ies):	7.6 ERP includes function of LMIS<br>7.12 Basic Office Skills<br>7.13 Have a command of technology
7.6 ERP includes function of LMIS	» Enable a Master Data Management System<br>» Understand the critical components of an ERP system<br>» Understand the meaning of common document types found within an ERP/LMIS

	» Understand the logic of data visibility within an ERP<br>» Understand the importance of data integrity, record management and information security
7.12 Basic Office Skills	» Have a good understanding of common presentation authoring packages<br>» Have a good understanding of common spreadsheet authoring packages<br>» Have a good understanding of common document authoring packages<br>» Have a good understanding of common email authoring packages<br>» Have a good understanding of common instant messaging packages<br>» Have a good understanding of common video conferencing authoring packages
7.13 Have a command of technology	» Enable the use of technology suitable to the job held<br>» Understand the need of monitoring new developments and technologies in the sector<br>» Compare new technologies and recognising potential benefits for the sector<br>» Understand that resources and support are provided across the organisation to enable colleagues to make the best use of available technology<br>» Understand that the organisation has a strategy for technology use<br>» Compare how to minimise environmental damage through technology use<br>» Enable the use of technology to maximise effectiveness and efficiency<br>» Enable the use of field-based technology (e.g., radio, general packet radio service (GPRS), satellite phone)
Advanced Technology Competency(ies):	7.2 Blockchain<br>7.7 Automation<br>7.9 Additive Manufacturing<br>7.10 Internet of things<br>7.14 eProcurement
7.2 Blockchain	» Examine blockchain as a value-add capability for relevant global health use cases
7.7 Automation	» Understand automation as a value-add capability for relevant global health use cases
7.9 Additive Manufacturing	» Understand additive manufacturing as a value-add capability for relevant global health use cases

7.10 Internet of things (IoT)	»	Understand the opportunity of IoT used in conjunction with AI
	»	Understand the internet of things as a value-add capability for relevant global health use cases
7.14 eProcurement	»	Understand how to transact with an eProcurement system where necessary
	»	Understand eProcurement as a value-add capability for relevant global health use cases
	»	Understand the benefits and limitations of eProcurement to best utilise the system

Key Performance Indicators:

- Assess Delivery Performance Cycle Time - Actual Time from assessed requirement to time delivered to end-user compared to committed Delivery Cycle Time as per Supply Chain strategy
- Assess Supplier Performance Cycle Time - Actual Supplier performance Cycle time versus Supplier commitment cycle time
- Inventory accuracy - Cycle count results
- Transportation lead time accuracy - Actual Transportation lead time versus Transportation promised lead time.
- Transport efficiency - Total cost of transport versus planned cost of transport by mode
- In-bound cost of acquisition - Actual total cost of acquisition versus Total Planned cost of acquisition including landed costs
- Manage performance cycle time
- Performance Report - Actual versus Plan reports for all metrics
- Work/activities executed in alignment with SOP’s
- Reports available on time

Training:	Qualifications Available:	Certifications Available:
» RP Systems<br>» Basic Finance<br>» Benchmarking<br>» Lean Manufacturing<br>» Performance Management<br>» Optimization<br>» Quality Management<br>» Six Sigma<br>» Supply Chain Performance Measurements		» Manage Logistics Operations skills programme<br>» Apply fundamental concepts and principles of supply chain management skills programme

» Total Quality Management (TQM)

» Risk Management

» Monitoring and Evaluation

Officer – SC Data Quality and Use

Organisation: SC-focused Organisation				
Competency Level:	Serial Number:	Primary Process:	Job Role:	Supervises:
Operational	5-56-551-2	Enable – Data Information	Officer – SC Data Quality and Use	-
Contributions	Building of the data gathering infrastructure to support the metrics of the Supply Chain organisation to enable the timeliness of metric generation and the ability to build data structures attached to the transaction systems capable of providing data from source transactions to build analytic models and data mining data with a view to build predictability models for risk mitigation and improved planning.			
Personal/Management Competency(ies):	5.5 Manage and plan projects<br>5.5.1 Develop and direct project plans<br>5.5.2 Execute strategic decision making<br>5.5.3 Manage partnerships<br>5.5.4 Direct/participate in teamwork<br>5.6 Manage financial activities<br>6.1 Demonstrate generic skills (e.g., literacy, numeracy, technology)<br>6.1.1 Exhibit high understanding of literacy and numeracy<br>6.2 Demonstrate strong communication skills<br>6.2.1 Practice cultural awareness			

5.5 Manage and plan projects	
5.5.1 Develop and direct project plans	» Understand the process to anticipate obstacles and critical events for ensuring the six rights of logistics management (right quantity, product, condition, place, time and cost) and the contingency plans to address

them; as well as the need to monitor progress and take necessary corrective action when needed

» Understand an operational plan that is consistent with the strategic plan, links specific goals and strategies, and identifies the performance indicators to be used to measure achievements

» Enable the use of information gathering techniques from stakeholders, analysing situations and identifying implications to make correct decisions

» Examine of the questions, “Where are we going?” and “How are we going to get there?” and the need to create a specific and purposeful path to achieve this.

» Enable the process reference model to managing supply chain processes (i.e., integrating business process re-engineering, benchmarking and process measurement)

» Understand the process of capturing the current state of a process and deriving the desired future state

» Enable the process of selecting and applying tools or technological solutions to frequently encountered problems

» Understand the need to provide staff with the necessary tools to carry out plans

» Understand the need to monitor progress and make changes as required

» Provide input at meetings for planning, organisation and monitoring logistics activities

» Understand that the staff are accountable for achieving the desired results

» Understand that planning, organising and managing resources bring about the successful completion of projects

» Compare the key features of a successful change management strategy

5.5.2 Execute strategic decision making

» Understand that one can make decisions regarding one’s own workload and area of responsibility

» Understand of need to prioritize competing tasks and perform them quickly and efficiently according to their importance; finding new ways of organizing or planning work to accomplish it more efficiently

» Understand the need to ensure proper time and space for consultation around decisions being made.

» Follow the process of considering the input of resources including staff needed

» Understand the need to check assumptions against facts

» Examine the key issues in a complex situation and come to the heart of the problem quickly

» Examine relevant information before making decisions

» Understand that strategic decisions are made in the interest of the organisation’s goals

- Understand when to analyse, evaluate and execute tough decisions when necessary
- Understand the impact of decisions on others' work or team goals
- Understand the process to communicate decisions and ensure they are incorporated into policies and processes
- Understand inclusive and consultative strategic planning to establish strategic objectives
- Examine and enable emerging trends and practices
- Understand the macro and long-term consequences of decisions

5.5.3 Manage partnerships

- Enable the concepts of partnership working
- Understand the process of supporting implementation of partnership programmes
- Understand the process of experimenting with and trialling new ideas with partners
- Understand the need to build partnerships to deliver programme and increase impact
- Enable innovation and creativity in partnership work
- Understand the need to communicate key information with partner members
- Understand the need to involve and value partners in all aspects of programming
- Examine problems with supply chain relationships
- Understand the need to communicate effectively with nurses, doctors and other members of the healthcare team
- Understand the need to respond to consumer complaints or comments about services and/or advice received
- Understand the need for collaboration and consultation across the sector and encourage the team to work with others to gain market insight
- Understand the need to encourage input from other key actors in the humanitarian sector and develop relationships with a cross-section of actors
- Understand the need to meet the reporting requirements of vertical programmes
- Understand the structure of the health system at a national level and explain this to others
- Compare the role of other members of the healthcare team (including with consumers) in a way that engenders understanding and confidence in the team and its members
- Understand the structure of the organisation, environment and/or service in which they work
- Understand how vertical programmes work within the health system

	» Understand the communication network established to achieve work outcomes<br>» Understand the structure of the health system at the provincial/regional level and explain this to others<br>» Understand the roles and functions of government agencies in regulating and supporting supply chain organisations<br>» Compare knowledge of change management (e.g. PtD Theory of Change)
5.5.4 Direct/participate in teamwork	» Understand the need to work with team members within their area of authority to establish achievable goals and strategies that are consistent with the objectives established for the organisation as a whole<br>» Interpret an organisational chart that shows the lines of reporting and responsibility among staff<br>» Understand the need to actively contribute a perspective and making a positive contribution to team-based problem solving and decision making<br>» Understand the need to provide feedback, encouragement and support to team members for progressing strategic goals<br>» Understand the need to monitor team performance in relation to the organisation's mission and goals<br>» Understand the need for teams to think for themselves and resolve problems<br>» Understand the need to encourage and harness diversity within the team to boost team effectiveness<br>» Understand the need to encourage and support the team to work through its stages of development and perform well<br>» Understand roles and responsibilities in relation to employees' expertise and the expectations of collaborating team members<br>» Understand where their position fits in the structure (organisation, SC, healthcare sector) , the responsibilities their position entails and the accountabilities linked to the position
5.6 Manage financial activities	» Follow rules for managing all resources with care, applying budgetary principles<br>» Understand the need to take overall responsibility for meeting budgets and donor requirements<br>» Understand budgets (national, regional, etc.) as necessary for work (e.g., wages budget, touring budget, stationery budget, project budgets for using NGO funds)<br>» Understand the process of producing timely and clear financial reports for funders and donors<br>» Understand the process of keeping records following government budgeting, accounting and financial practices<br>» Understand the process of maintaining an overview of multiple budgets from multiple sources

	» Understand the process of seeking and using information on financial funding requirements<br>» Understand the need for timely decision making with regard to financial shortfalls<br>» Understand the need for budget tracking for logistics activities according to established procedures<br>» Understand the need for analysing of financial statements and explaining components of a balance sheet and income statement<br>» Understand interactive decision support models that allow the development of multiple scenarios and demonstrating the sensitivity of multiple independent variables<br>» Examine the success or failure of a business using financial accounting<br>» Understand the calculations of the total system cost of delivering a product or service to the customer<br>» Compare the key considerations for developing a business plan<br>» Examine the structure against which business performance will be monitored (e.g., cost centres, chart of accounts)<br>» Interpret key performance indicators used to monitor business performance (e.g., turnover, profitability)<br>» Compare strategies for minimising the risk of fraudulent activity (e.g., reconciliation of purchase orders, receipts and payment approvals; dual signatory arrangements for funds transfers)<br>» Understand investigative processes and options for confirming the existence of fraudulent activity (e.g., sample audits, forensic accounting services)<br>» Understand the process of preparing funding applications for trusts and grants<br>» Understand the need to ensure financial transparency<br>» Compare the general monetary value of medicines and equipment<br>» Understand the need to secure financing and managing budgets to support distribution operations<br>» Compare the ability to translate financial information into SC language<br>» Understand SC finance
6.1 Demonstrate generic skills (e.g., literacy, numeracy, technology)	
6.1.1 Exhibit high understanding of literacy and numeracy	» Speak clearly and confidently, organize information in a logical manner, and consider voice tone and pace<br>» Respond to verbal messages and other cues (active listening)<br>» Express ideas and opinions clearly in written and verbal form<br>» Communicate information accurately, concisely and confidently in writing and verbally<br>» Practice a level of mathematics suitable to the job held

	» Compare visual presentation techniques, including charting, histograms and flow sheets<br>» Read and comprehend at a level necessary to properly complete duties of the position<br>» Understand the need to interpret written information in documents, such as reports, SOPs, LMIS forms, graphs, calendars, schedules, notices and directions<br>» Understand the need to pay attention to detail and identify the main ideas, detect inconsistencies and identify missing information in documents<br>» Enable computer skills — such as using Word, Excel, PowerPoint and the internet — suitable to the job level<br>» Understand the process of analysing and validating KPIs<br>» Understand how the evaluation process of activities that act as standards against which the KPIs are measured is carried out<br>» Examine the process of performing critical self-review on work before submission
6.2 Demonstrate strong communication skills	» Be truthful and trustworthy, and supply accurate information at all times<br>» Enable working as part of a workplace team<br>» Understand vocabulary and communication style and form for both written and verbal communication that is appropriate for the situation, audience and material being communicated (e.g., avoid unnecessary jargon, clearly explain medical and SCM terminology)<br>» Enable open communication within constraints of confidentiality<br>» Participate in the organisation of communications about logistics<br>» Enable participation in meetings, expressing one's opinions, being aware of others' needs, and being appropriately assertive when required<br>» Show perspective into the point of view of others, understanding their needs and goals<br>» Enable the practise of building trust, rapport and credibility with others<br>» Understand the communication needs and concerns of others and respond to them<br>» Understand the need to avoid conflict between work and personal interests<br>» Enable meaningful two-way communication<br>» Show influence on others by persuasively presenting thoughts and ideas<br>» Show assertiveness skills to deal with unreasonable requests and/or refusals that would compromise practice or consumer care<br>» Maintain a positive, supportive and appreciative attitude<br>» Show the ability to balance the organisation's needs and the customer's needs

- Show self-control by maintaining composure and keeping emotions in check, even in difficult situations; deal calmly and effectively with stressful situations
- Enable expressing of opinions and providing information in written and/or verbal form in a manner that does not elicit concern, anger or other adverse response
- Enable the use of a systematic process for following up that demonstrates written reports have been received and understood
- Understand the means by which responses to input to the work environment are monitored
- Seek practical ways to overcome barriers to communication
- Understand the need to ensuring language representation (e.g., interpreters) at meetings when appropriate
- Interpret difficult situations and disputes between staff that need to be tackled and resolved
- Enable giving both positive and negative feedback sensitively
- Understand the need to maintain and improve communication
- Examine the process of giving and requesting feedback frequently
- Examine effective communication with shop stewards and union representatives

6.2.1 Practice cultural awareness

- Understand the need for cultural awareness and sensitivity, treating all people with fairness, respect and dignity
- Enable anti-discriminatory practices in the organisation, including HR and disciplinary procedures
- Enable cultural sensitivity, equality and fairness at all levels of the organisation
- Challenge discriminatory behaviour directly and sensitively
- Act in a non-discriminatory way toward individuals and groups
- Enable integrating cultural awareness in learning and development approaches
- Avoid stereotypical responses by examining one's own behaviour and bias
- Enable managing cultural diversity in teams and make the most of differences
- Compare strategies and/or resources for communicating effectively with people from different cultural backgrounds
- Enable the process to elicit information relating to values, beliefs and cultural backgrounds of consumers that may influence the way professional services are provided

Basal Technology Competency(ies):	7.1 Data Science<br>7.4 Temperature and monitoring<br>7.5 Planning systems

	7.6 ERP includes function of LMIS<br>7.12 Basic Office Skills<br>7.13 Have a command of technology
7.1 Data Science	» Understand end to end MIS technology components that are inherent in advanced MIS systems and explore how these may be relevant in different supply chain contexts<br>» Compare emerging technological approaches to data visualization and analysis<br>» Awareness of analytics (data interpretation and decision making)<br>» Enable the ability to interact, provide guidance for data (dashboard) consolidation in various levels and use the tech tools<br>» Support movement of data into electronic form at lowest/remote SC level<br>» Enable the ability to work with relational database
7.4 Temperature and monitoring	» Compare the current technology approaches for Temperature Monitoring Sensors<br>» Consider what is required to implement this technology in a country context<br>» Compare infrastructure requirement for heat-sensitive items at peripheral 'lower level' of SC depending on volume and nature of items to handle;<br>» Examine regular preventive maintenance and calibration of cold-chain equipment with biomedical engineers or logisticians<br>» Understand the policy for temperature monitoring tools during transportation and storage<br>» Understand the steps involved in health logistic management cycle: selection-order-delivery-reception-storage-distribution-inventory<br>» Understand hardware qualification and system validation of temperature monitoring<br>» Understand mass data management of temperature monitoring
7.5 Planning systems	» Examine blockchain and understand the building blocks for planning systems<br>» Examine planning systems as a value-add capability for relevant global health use cases
7.6 ERP includes function of LMIS	» Enable a Master Data Management System<br>» Understand the critical components of an ERP system<br>» Understand the meaning of common document types found within an ERP/LMIS<br>» Understand the logic of data visibility within an ERP

	» Understand the importance of data integrity, record management and information security
7.12 Basic Office Skills	» Have a good understanding of common presentation authoring packages<br>» Have a good understanding of common spreadsheet authoring packages<br>» Have a good understanding of common document authoring packages<br>» Have a good understanding of common email authoring packages<br>» Have a good understanding of common instant messaging packages<br>» Have a good understanding of common video conferencing authoring packages
7.13 Have a command of technology	» Enable the use of technology suitable to the job held<br>» Understand the need of monitoring new developments and technologies in the sector<br>» Compare new technologies and recognising potential benefits for the sector<br>» Understand that resources and support are provided across the organisation to enable colleagues to make the best use of available technology<br>» Understand that the organisation has a strategy for technology use<br>» Compare how to minimise environmental damage through technology use<br>» Enable the use of technology to maximise effectiveness and efficiency<br>» Enable the use of field-based technology (e.g., radio, general packet radio service (GPRS), satellite phone)
Advanced Technology Competency(ies):	7.2 Blockchain<br>7.3 Unmanned Aerial Vehicles (UAVs)<br>7.7 Automation<br>7.8 Artificial Intelligence<br>7.9 Additive Manufacturing<br>7.10 Internet of things<br>7.11 Cloud Computing<br>7.14 eProcurement
7.2 Blockchain	» Examine blockchain as a value-add capability for relevant global health use cases

7.3 Unmanned Aerial Vehicles (UAVs)	» Understand the current UAV landscape, their application and the enabling environment required to support country implementation<br>» Understand UAVs as a value-add capability for relevant global health use cases<br>» Understand the laws/ regulations around the use of UAV's
7.7 Automation	» Understand automation as a value-add capability for relevant global health use cases
7.8 Artificial Intelligence	» Understand artificial intelligence as a value-add capability for relevant global health use cases
7.9 Additive Manufacturing	» Understand additive manufacturing as a value-add capability for relevant global health use cases
7.10 Internet of things (IoT)	» Understand the opportunity of IoT used in conjunction with AI<br>» Understand the internet of things as a value-add capability for relevant global health use cases
7.11 Cloud Computing	» Understand cloud computing as a value-add capability for relevant global health use cases
7.14 eProcurement	» Understand how to transact with an eProcurement system where necessary<br>» Understand eProcurement as a value-add capability for relevant global health use cases<br>» Understand the benefits and limitations of eProcurement to best utilise the system

Key Performance Indicators:

» Assess Delivery Performance Cycle Time - Actual Time from assessed requirement to time delivered to end-user compared to committed Delivery Cycle Time as per Supply Chain strategy

» Assess Supplier Performance Cycle Time - Actual Supplier performance Cycle time versus Supplier commitment cycle time

» Inventory accuracy - Cycle count results

» Transportation lead time accuracy - Actual Transportation lead time versus Transportation promised lead time.

» Transport efficiency - Total cost of transport versus planned cost of transport by mode

» In-bound cost of acquisition - Actual total cost of acquisition versus Total Planned cost of acquisition including landed costs

- Manage performance cycle time
- Performance Report - Actual versus Plan reports for all metrics

Training:	Qualifications Available:	Certifications Available:
» Data management<br>» ERP Systems<br>» Installed base management<br>» Interpreting Specifications<br>» Office automation tools<br>» Product Information Management (Product Data Management)		» Manage Logistics Operations skills programme<br>» Lean Six Sigma Certification in Finance white belt

Officer - Human Resources Specialist

Organisation: SC-focused Organisation				
Competency Level:	Serial Number:	Primary Process:	Job Role:	Supervises:
Operational	5-571-572-2	Enable – Human Resources	Officer - Human Resources Specialist	Assistant - Human Resources Assistant

Contributions	Manages specific human resources requirements, recruitments within certain competencies within the supply chain and identifies training for those individuals.
Personal/Management Competency(ies):	5.7 Oversee/support human resources (e.g., recruitment, training, team management/ supervision)<br>5.7.1 Manage staff recruitment process<br>5.7.2 Train staff<br>5.7.3 Supervise staff<br>5.7.4 Assessing HR systems<br>6.1 Demonstrate generic skills (e.g., literacy, numeracy, technology)<br>6.1.1 Exhibit high understanding of literacy and numeracy<br>6.2 Demonstrate strong communication skills<br>6.2.1 Practice cultural awareness

5.7 Oversee/support human resources (e.g., recruitment, training, team management/ supervision)

5.7.1 Manage staff recruitment process

- » Enable use of a system for staff recruitment, appraisals and monitoring
- » Follow training and recruitment plans to fill any identified gaps and shortages
- » Compare relevant and appropriate selection criteria for a defined role
- » Understand and follow the standard selection documentation for recruitment
- » Understand key issues impacting the size and membership of an interview panel (e.g., logistics, gender balance, absence of conflict of interest)
- » Compare requirements for a fair, defensible and balanced interview process
- » Understand and follow the documentation maintained for each interviewed position
- » Understand due process for formalising an appointment that minimises the opportunity for untoward effects (e.g., appeal, conflict, resignation)
- » Understand job descriptions to obtain and retain skilled staff

	» Understand the process of consulting with and involving relevant personnel in development and updating of role descriptions/duty statements that clarify the duties and responsibilities of positions<br>» Understand the process of orienting new staff to the workplace, explaining standard operating systems and procedures<br>» Examine human resource plans to meet the future staffing needs of the organisation<br>» Examine key features and measures used for monitoring a personnel retention strategy (e.g., recruitment costs, staff turnover, staff surveys)<br>» Understand the legislative issues impacting human resource policies and procedures<br>» Understand the process of maintaining employee master data including medicals, allergies to ensure safe working environment<br>» Compare an employee opinion survey
5.7.2 Train staff	» Follow the process of maintaining a list of staff that require logistics system training (based on supportive supervision reports)<br>» Understand the process of coordinating all training in managing the logistics system for all health facility staff<br>» Enable a culture of promoting participation in relevant learning and development opportunities<br>» Understand the process of assisting in training nurses and other health professionals in medicines ordering and storage procedures<br>» Understand the process of orienting new doctors to the formulary and the systems and procedures of the medicines supply system<br>» Understand the process of providing on-the-job training to district health officials and hospital pharmacists as needed<br>» Understand the need to ensure staff have the necessary skills and understanding for safe practice in the event they need to fill a management role due to absence or illness<br>» Understand the need to ensure all staff handling hazardous materials receive training on safety & compliance regulations<br>» Examine any staff needs at all levels of the supply chain<br>» Examine key factors important for initiating and/or sustaining the motivation of adult learners<br>» Compare the variable learning needs and styles or modalities in adult learners (e.g., visual versus auditory)<br>» Compare a range of strategies that could be used to reinforce and clarify educational content (e.g., tutorials or workshops, written materials, intranet- or internet-based resources)<br>» Understand the use train-the-trainer to equipment trainers<br>» Examine training effectiveness monitoring and training feedback systems
5.7.3 Supervise staff	» Enable the use of a performance management process with all personnel to ensure continuous improvement

	» Understand the need for an environment that is supportive of learning and professional development<br>» Interpret the nature of the supervisory role, what is meant by direct supervision, and where responsibility for outputs and outcomes rests<br>» Understand the need to plan and conduct supervisory tours of dependent facilities<br>» Understand the need to identify situations where supervised personnel are experiencing difficulties in completing work activities and/or where a mandatory notification obligation exists<br>» Interpret signs/cues from supervised personnel that indicate additional guidance or support is needed (e.g., hesitancy, distress, seeks clarification from less authoritative sources)<br>» Understand the need to provide feedback, encouragement and support to team members<br>» Understand the need to discuss, in a constructive manner, areas where performance should/could be improved and provide resources to support this<br>» Understand the need to discuss expectations, achievements and contributions with personnel in a fair and equitable manner<br>» Understand the need to support colleagues in creating a professional development plan and suggesting ways the plan may be progressed through relevant training and/or experiential learning opportunities<br>» Understand the need to encourage and support high-potential employees to accept growth challenges outside their own areas<br>» Understand the progression opportunities for employees who have prepared themselves through development<br>» Understand the need to identify and deal with unproductive staff using an approved process in an ethical manner<br>» Understand the need to identify and address work issues contributing to impairment of personnel (e.g., excessive workload, conflict)<br>» Understand the complaints management process<br>» Examine situations where additional expertise should be sought (e.g., counselling in the event of workplace violence or in situations of diminished performance due to drug or alcohol abuse)<br>» Understand different working styles
5.7.4 Assessing HR systems	» Understand the need to assess HR systems
6.1 Demonstrate generic skills (e.g., literacy, numeracy, technology)	
6.1.1 Exhibit high understanding of literacy and numeracy	» Speak clearly and confidently, organize information in a logical manner, and consider voice tone and pace<br>» Respond to verbal messages and other cues (active listening)<br>» Express ideas and opinions clearly in written and verbal form<br>» Communicate information accurately, concisely and confidently in writing and verbally

» Practice a level of mathematics suitable to the job held

» Compare visual presentation techniques, including charting, histograms and flow sheets

» Read and comprehend at a level necessary to properly complete duties of the position

» Understand the need to interpret written information in documents, such as reports, SOPs, LMIS forms, graphs, calendars, schedules, notices and directions

» Understand the need to pay attention to detail and identify the main ideas, detect inconsistencies and identify missing information in documents

» Enable computer skills — such as using Word, Excel, PowerPoint and the internet — suitable to the job level

» Understand the process of analysing and validating KPIs

» Understand how the evaluation process of activities that act as standards against which the KPIs are measured is carried out

» Examine the process of performing critical self-review on work before submission

6.2 Demonstrate strong communication skills

» Be truthful and trustworthy, and supply accurate information at all times

» Enable working as part of a workplace team

» Understand vocabulary and communication style and form for both written and verbal communication that is appropriate for the situation, audience and material being communicated (e.g., avoid unnecessary jargon, clearly explain medical and SCM terminology)

» Enable open communication within constraints of confidentiality

» Participate in the organisation of communications about logistics

» Enable participation in meetings, expressing one's opinions, being aware of others' needs, and being appropriately assertive when required

» Show perspective into the point of view of others, understanding their needs and goals

» Enable the practise of building trust, rapport and credibility with others

» Understand the communication needs and concerns of others and respond to them

» Understand the need to avoid conflict between work and personal interests

» Enable meaningful two-way communication

» Show influence on others by persuasively presenting thoughts and ideas

» Show assertiveness skills to deal with unreasonable requests and/or refusals that would compromise practice or consumer care

» Maintain a positive, supportive and appreciative attitude

» Show the ability to balance the organisation's needs and the customer's needs

» Show self-control by maintaining composure and keeping emotions in check, even in difficult situations; deal calmly and effectively with stressful situations

	» Enable expressing of opinions and providing information in written and/or verbal form in a manner that does not elicit concern, anger or other adverse response<br>» Enable the use of a systematic process for following up that demonstrates written reports have been received and understood<br>» Understand the means by which responses to input to the work environment are monitored<br>» Seek practical ways to overcome barriers to communication<br>» Understand the need to ensuring language representation (e.g., interpreters) at meetings when appropriate<br>» Interpret difficult situations and disputes between staff that need to be tackled and resolved<br>» Enable giving both positive and negative feedback sensitively<br>» Understand the need to maintain and improve communication<br>» Examine the process of giving and requesting feedback frequently<br>» Examine effective communication with shop stewards and union representatives
6.2.1 Practice cultural awareness	» Understand the need for cultural awareness and sensitivity, treating all people with fairness, respect and dignity<br>» Enable anti-discriminatory practices in the organisation, including HR and disciplinary procedures<br>» Enable cultural sensitivity, equality and fairness at all levels of the organisation<br>» Challenge discriminatory behaviour directly and sensitively<br>» Act in a non-discriminatory way toward individuals and groups<br>» Enable integrating cultural awareness in learning and development approaches<br>» Avoid stereotypical responses by examining one's own behaviour and bias<br>» Enable managing cultural diversity in teams and make the most of differences<br>» Compare strategies and/or resources for communicating effectively with people from different cultural backgrounds<br>» Enable the process to elicit information relating to values, beliefs and cultural backgrounds of consumers that may influence the way professional services are provided
Basal Technology Competency(ies):	7.12 Basic Office Skills<br>7.13 Have a command of technology
7.12 Basic Office Skills	» Have a good understanding of common presentation authoring packages

	» Have a good understanding of common spreadsheet authoring packages<br>» Have a good understanding of common document authoring packages<br>» Have a good understanding of common email authoring packages<br>» Have a good understanding of common instant messaging packages<br>» Have a good understanding of common video conferencing authoring packages
7.13 Have a command of technology	» Enable the use of technology suitable to the job held<br>» Understand the need of monitoring new developments and technologies in the sector<br>» Compare new technologies and recognising potential benefits for the sector<br>» Understand that resources and support are provided across the organisation to enable colleagues to make the best use of available technology<br>» Understand that the organisation has a strategy for technology use<br>» Compare how to minimise environmental damage through technology use<br>» Enable the use of technology to maximise effectiveness and efficiency<br>» Enable the use of field-based technology (e.g., radio, general packet radio service (GPRS), satellite phone)
Advanced Technology Competency(ies):	7.11 Cloud Computing
7.11 Cloud Computing	» Understand cloud computing as a value-add capability for relevant global health use cases

Key Performance Indicators:

- Competency compliance. No of personnel evaluated and found to competent versus plan.
- Number of personnel with the required Skill, Education, Training and Aptitude as per Supply Chain Strategy versus Total number of personnel.
- Number of personnel progressed professionally versus Number of personnel that were targeted for progress.
- Planned skills upliftment plan – Number of personnel per competency versus actual skills upliftment number of personnel per competency.
- HR4SCM Competency Requirements & Plan
- Creation of the HR4SCM Competency Requirements & Plan based on the Supply Chain Requirements
- Number of personnel with the required Skill, Education, Training and Aptitude as per Supply Chain Strategy versus Total number of personnel.
- Number of personnel progressed professionally versus Number of personnel that were targeted for progress.
- Planned skills upliftment plan - Number of personnel per competency versus actual skills upliftment number of personnel per competency.

- Competency compliance. No of personnel evaluated and found to competent versus plan.
- HR4SCM Competency Requirements & Plan
- Direct Labour Cost
- Human Resource Contract Management
- EHS regulations
- Legislation and Standards
- Performance Management
- 100% completion of Training Plan as scheduled
- >80% score on Employee Opinion Survey (EOS)

Training:	Qualifications Available:	Certifications Available:
» Legislation and Standards<br>» Human resources for supply chain<br>» Introductory Organisational and Industrial Psychology<br>» Train-the-trainer	» Diploma: Human Resource Management	

Officer – Programmes (Donations) Procurement

Organisation:

SC-focused Organisation

Competency Level:	Serial Number:	Primary Process:	Job Role:	Supervises:
Operational	2-26-27-2	Procure	Officer – Programmes (Donations) Procurement	-

Contributions	Administers the programmes established by the Donors and Global Partners, ensures adherence to protocols as stipulated in the agreements. Manages the timeous procurement of materials and services to support the Supply Chain Strategy of the Public Health System Organisation.
Technical Competency(ies):	2.6 Manage import and export of products<br>2.7 Manage donations of products

2.6 Manage import and export of products

- » Examine compliance with import, export and customs procedures and requirements
- » Compare import or export requirements for the supplies
- » Examine import or export documentation for the supplies
- » Examine the completion of import and export procedures
- » Understand the importance to identify any problems with the import and export procedures and requirements
- » Understand international business processes (e.g. shipping, air cargo, clearing and forwarding, financial transactions, business ethics)

2.7 Manage donations of products

- » Understand the national donations policy, referring to the appropriate Ministry and customs office for advice
- » Understand to say no to donations that are not consistent with national donation policy
- » Understand how to manage donation plan in emergency situations

Personal/Management Competency(ies):	6.1 Demonstrate generic skills (e.g., literacy, numeracy, technology)<br>6.1.1 Exhibit high understanding of literacy and numeracy<br>6.2 Demonstrate strong communication skills<br>6.2.1 Practice cultural awareness<br>6.3 Utilize problem-solving skills<br>6.3.1 Negotiate

6.1 Demonstrate generic skills (e.g., literacy, numeracy, technology)

6.1.1 Exhibit high understanding of literacy and numeracy	» Speak clearly and confidently, organize information in a logical manner, and consider voice tone and pace<br>» Respond to verbal messages and other cues (active listening)<br>» Express ideas and opinions clearly in written and verbal form<br>» Communicate information accurately, concisely and confidently in writing and verbally<br>» Practice a level of mathematics suitable to the job held<br>» Compare visual presentation techniques, including charting, histograms and flow sheets<br>» Read and comprehend at a level necessary to properly complete duties of the position<br>» Understand the need to interpret written information in documents, such as reports, SOPs, LMIS forms, graphs, calendars, schedules, notices and directions<br>» Understand the need to pay attention to detail and identify the main ideas, detect inconsistencies and identify missing information in documents<br>» Enable computer skills — such as using Word, Excel, PowerPoint and the internet — suitable to the job level<br>» Understand the process of analysing and validating KPIs<br>» Understand how the evaluation process of activities that act as standards against which the KPIs are measured is carried out<br>» Examine the process of performing critical self-review on work before submission
6.2 Demonstrate strong communication skills	» Be truthful and trustworthy, and supply accurate information at all times<br>» Enable working as part of a workplace team<br>» Understand vocabulary and communication style and form for both written and verbal communication that is appropriate for the situation, audience and material being communicated (e.g., avoid unnecessary jargon, clearly explain medical and SCM terminology)<br>» Enable open communication within constraints of confidentiality<br>» Participate in the organisation of communications about logistics<br>» Enable participation in meetings, expressing one’s opinions, being aware of others’ needs, and being appropriately assertive when required<br>» Show perspective into the point of view of others, understanding their needs and goals<br>» Enable the practise of building trust, rapport and credibility with others<br>» Understand the communication needs and concerns of others and respond to them<br>» Understand the need to avoid conflict between work and personal interests<br>» Enable meaningful two-way communication<br>» Show influence on others by persuasively presenting thoughts and ideas

	» Show assertiveness skills to deal with unreasonable requests and/or refusals that would compromise practice or consumer care<br>» Maintain a positive, supportive and appreciative attitude<br>» Show the ability to balance the organisation's needs and the customer's needs<br>» Show self-control by maintaining composure and keeping emotions in check, even in difficult situations; deal calmly and effectively with stressful situations<br>» Enable expressing of opinions and providing information in written and/or verbal form in a manner that does not elicit concern, anger or other adverse response<br>» Enable the use of a systematic process for following up that demonstrates written reports have been received and understood<br>» Understand the means by which responses to input to the work environment are monitored<br>» Seek practical ways to overcome barriers to communication<br>» Understand the need to ensuring language representation (e.g., interpreters) at meetings when appropriate<br>» Interpret difficult situations and disputes between staff that need to be tackled and resolved<br>» Enable giving both positive and negative feedback sensitively<br>» Understand the need to maintain and improve communication<br>» Examine the process of giving and requesting feedback frequently<br>» Examine effective communication with shop stewards and union representatives
6.2.1 Practice cultural awareness	» Understand the need for cultural awareness and sensitivity, treating all people with fairness, respect and dignity<br>» Enable anti-discriminatory practices in the organisation, including HR and disciplinary procedures<br>» Enable cultural sensitivity, equality and fairness at all levels of the organisation<br>» Challenge discriminatory behaviour directly and sensitively<br>» Act in a non-discriminatory way toward individuals and groups<br>» Enable integrating cultural awareness in learning and development approaches<br>» Avoid stereotypical responses by examining one's own behaviour and bias<br>» Enable managing cultural diversity in teams and make the most of differences<br>» Compare strategies and/or resources for communicating effectively with people from different cultural backgrounds<br>» Enable the process to elicit information relating to values, beliefs and cultural backgrounds of consumers that may influence the way professional services are provided
6.3 Utilize problem-solving skills	» Examine information to solve problems in a sensitive and ethical manner

- Understand the need to manage day to day and complex problems in a timely manner
- Understand the need to explain problem situations and their step-by-step transformation based on planning and reasoning, without apportioning blame
- Understand the need to critically review, analyse, synthesize, compare and interpret information; draw conclusions from relevant and/or missing information; and understand the relationship among facts and apply this understanding when solving problems
- Compare a range of possible approaches/strategies that are effective for resolving conflict in the workplace (e.g., negotiation, collaborative problem-solving, mediation, arbitration)
- Enable an open environment that encourages people to work together
- Understand the need to resolve conflicts as they arise
- Enable creativity through questioning, attempting to improve on ideas, applying other experiences and working toward action in problem solving
- Understand that one can break down facts and thoughts into strengths and weaknesses
- Enable thinking in a careful way to solve problems, analyse data, and recall and apply information, involving others
- Enable goal-directed thinking and action in situations in which no routine solutions exist
- Understand the need to maintain appropriate ethical and moral standards in resolving problems
- Compare alternative courses of action, using cognitive processes such as memory, thinking and evaluation
- Understand how to map likely consequences of decisions to choose the best course of action
- Understand how to ask other people to help with solving problems
- Examine difficult or unusual situations to develop unique approaches and useful solutions
- Understand the need to commit to a solution in a timely manner, and develop a realistic approach for applying the chosen solution; evaluate the outcome of the solution to see if further action is needed, and identify lessons learned
- Examine the impact of conflict in the workplace (e.g., tension, low morale, absenteeism, system or service failure, aggressive or uncooperative behaviours)
- Enable the practise of following up on problems to ensure they are fixed
- Understand situations where referral is warranted (e.g., severe emotional distress, intractable dispute)

6.3.1 Negotiate

- Understand the principles of negotiation and aim for a win-win outcome
- Understand the need to manage and resolve relationship issues within and outside the team
- Understand solution-focussed approaches to further the organisation's mission

	» Understand how to build consensus among parties<br>» Compare alternative ways of doing things<br>» Examine where fair approaches are being utilised to resolve issues<br>» Understand how to seek to reach constructive solutions while maintaining positive working relationships<br>» Understand how to adapt style to take into account cultural differences regarding negotiation<br>» Understand how to build consensus at a high level for the benefit of all parties
Basal Technology Competency(ies):	7.6 ERP includes function of LMIS<br>7.12 Basic Office Skills<br>7.13 Have a command of technology
7.6 ERP includes function of LMIS	» Enable a Master Data Management System<br>» Understand the critical components of an ERP system<br>» Understand the meaning of common document types found within an ERP/LMIS<br>» Understand the logic of data visibility within an ERP<br>» Understand the importance of data integrity, record management and information security
7.12 Basic Office Skills	» Have a good understanding of common presentation authoring packages<br>» Have a good understanding of common spreadsheet authoring packages<br>» Have a good understanding of common document authoring packages<br>» Have a good understanding of common email authoring packages<br>» Have a good understanding of common instant messaging packages<br>» Have a good understanding of common video conferencing authoring packages
7.13 Have a command of technology	» Enable the use of technology suitable to the job held<br>» Understand the need of monitoring new developments and technologies in the sector<br>» Compare new technologies and recognising potential benefits for the sector<br>» Understand that resources and support are provided across the organisation to enable colleagues to make the best use of available technology<br>» Understand that the organisation has a strategy for technology use<br>» Compare how to minimise environmental damage through technology use<br>» Enable the use of technology to maximise effectiveness and efficiency

» Enable the use of field-based technology (e.g., radio, general packet radio service (GPRS), satellite phone)

Advanced Technology Competency(ies):	7.2 Blockchain<br>7.11 Cloud Computing
7.2 Blockchain	» Examine blockchain as a value-add capability for relevant global health use cases
7.11 Cloud Computing	» Understand cloud computing as a value-add capability for relevant global health use cases

Key Performance Indicators:

» Accuracy of Risk Register

» Cycle time of QA/QC report

» Overall value at risk

» Partner/Donor integration, policy, guidelines and regulatory reforms

» QA/QC report

» Risk / Mitigation Costs

» Time to Recovery (TTR)

Training:	Qualifications Available:	Certifications Available:
» Import & export regulations for products<br>» Inventory management, Min-Max Replenishment		» CIPS Level 3 Advanced Certificate in Procurement and Supply Operations

Officer – SC Data and Analytics

Organisation: SC-focused Organisation				
Competency Level:	Serial Number:	Primary Process:	Job Role:	Supervises:
Operational	5-56-551-52-2	Enable – Data Information	Officer – SC Data and Analytics	-

Contributions	Ensures that the Metrics monitoring systems are performing as designed, troubleshoot where and when the operators have queries with the reporting.
Personal/Management Competency(ies):	5.2 Oversee operating of a logistics management information system<br>5.5 Manage and plan projects<br>5.5.1 Develop and direct project plans<br>5.5.2 Execute strategic decision making<br>5.5.3 Manage partnerships<br>5.5.4 Direct/participate in teamwork<br>5.6 Manage financial activities<br>6.1 Demonstrate generic skills (e.g., literacy, numeracy, technology)<br>6.1.1 Exhibit high understanding of literacy and numeracy<br>6.2 Demonstrate strong communication skills<br>6.2.1 Practice cultural awareness

5.2 Oversee operating of a logistics management information system

» Enable information visibility in inventory levels, production and materials in transit

» Understand appropriate recording systems. (e.g., stock cards, order forms, computer systems)

» Understand the process of aggregating, analysing, and interpreting logistics data to produce reports on logistics system performance

» Understand that reports are disseminated up and down the supply chain to all appropriate stakeholders

» Understand the need to communicate directly with facilities for receipt, review and approval of reports and/or orders; follow up on missing reports/orders; and generate feedback reports for the facilities

» Understand the need to monitor the supply pipeline and assessing stock status

» Enable a logical system for secure storage of records

» Understand the urgency of required information

» Follow the process of assessing specific requirements for undertaking work (e.g., information, stock, equipment, access to specific expertise) and ensuring those requirements are or can be met

» Show knowledge of the national medicines supply information system (electronic or manual)

5.5 Manage and plan projects

5.5.1 Develop and direct project plans

- Understand the process to anticipate obstacles and critical events for ensuring the six rights of logistics management (right quantity, product, condition, place, time and cost) and the contingency plans to address them; as well as the need to monitor progress and take necessary corrective action when needed
- Understand an operational plan that is consistent with the strategic plan, links specific goals and strategies, and identifies the performance indicators to be used to measure achievements
- Enable the use of information gathering techniques from stakeholders, analysing situations and identifying implications to make correct decisions
- Examine of the questions, “Where are we going?” and “How are we going to get there?” and the need to create a specific and purposeful path to achieve this.
- Enable the process reference model to managing supply chain processes (i.e., integrating business process re-engineering, benchmarking and process measurement)
- Understand the process of capturing the current state of a process and deriving the desired future state
- Enable the process of selecting and applying tools or technological solutions to frequently encountered problems
- Understand the need to provide staff with the necessary tools to carry out plans
- Understand the need to monitor progress and make changes as required
- Provide input at meetings for planning, organisation and monitoring logistics activities
- Understand that the staff are accountable for achieving the desired results
- Understand that planning, organising and managing resources bring about the successful completion of projects
- Compare the key features of a successful change management strategy

5.5.2 Execute strategic decision making

- Understand that one can make decisions regarding one’s own workload and area of responsibility
- Understand of need to prioritize competing tasks and perform them quickly and efficiently according to their importance; finding new ways of organizing or planning work to accomplish it more efficiently
- Understand the need to ensure proper time and space for consultation around decisions being made.
- Follow the process of considering the input of resources including staff needed
- Understand the need to check assumptions against facts
- Examine the key issues in a complex situation and come to the heart of the problem quickly
- Examine relevant information before making decisions

	» Understand that strategic decisions are made in the interest of the organisation's goals<br>» Understand when to analyse, evaluate and execute tough decisions when necessary<br>» Understand the impact of decisions on others' work or team goals<br>» Understand the process to communicate decisions and ensure they are incorporated into policies and processes<br>» Understand inclusive and consultative strategic planning to establish strategic objectives<br>» Examine and enable emerging trends and practices<br>» Understand the macro and long-term consequences of decisions
5.5.3 Manage partnerships	» Enable the concepts of partnership working<br>» Understand the process of supporting implementation of partnership programmes<br>» Understand the process of experimenting with and trialling new ideas with partners<br>» Understand the need to build partnerships to deliver programme and increase impact<br>» Enable innovation and creativity in partnership work<br>» Understand the need to communicate key information with partner members<br>» Understand the need to involve and value partners in all aspects of programming<br>» Examine problems with supply chain relationships<br>» Understand the need to communicate effectively with nurses, doctors and other members of the healthcare team<br>» Understand the need to respond to consumer complaints or comments about services and/or advice received<br>» Understand the need for collaboration and consultation across the sector and encourage the team to work with others to gain market insight<br>» Understand the need to encourage input from other key actors in the humanitarian sector and develop relationships with a cross-section of actors<br>» Understand the need to meet the reporting requirements of vertical programmes<br>» Understand the structure of the health system at a national level and explain this to others<br>» Compare the role of other members of the healthcare team (including with consumers) in a way that engenders understanding and confidence in the team and its members<br>» Understand the structure of the organisation, environment and/or service in which they work<br>» Understand how vertical programmes work within the health system

» Understand the communication network established to achieve work outcomes

» Understand the structure of the health system at the provincial/regional level and explain this to others

» Understand the roles and functions of government agencies in regulating and supporting supply chain organisations

» Compare knowledge of change management (e.g. PtD Theory of Change)

5.5.4 Direct/participate in teamwork

» Understand the need to work with team members within their area of authority to establish achievable goals and strategies that are consistent with the objectives established for the organisation as a whole

» Interpret an organisational chart that shows the lines of reporting and responsibility among staff

» Understand the need to actively contribute a perspective and making a positive contribution to team-based problem solving and decision making

» Understand the need to provide feedback, encouragement and support to team members for progressing strategic goals

» Understand the need to monitor team performance in relation to the organisation's mission and goals

» Understand the need for teams to think for themselves and resolve problems

» Understand the need to encourage and harness diversity within the team to boost team effectiveness

» Understand the need to encourage and support the team to work through its stages of development and perform well

» Understand roles and responsibilities in relation to employees' expertise and the expectations of collaborating team members

» Understand where their position fits in the structure (organisation, SC, healthcare sector) , the responsibilities their position entails and the accountabilities linked to the position

5.6 Manage financial activities

» Follow rules for managing all resources with care, applying budgetary principles

» Understand the need to take overall responsibility for meeting budgets and donor requirements

» Understand budgets (national, regional, etc.) as necessary for work (e.g., wages budget, touring budget, stationery budget, project budgets for using NGO funds)

» Understand the process of producing timely and clear financial reports for funders and donors

» Understand the process of keeping records following government budgeting, accounting and financial practices

» Understand the process of maintaining an overview of multiple budgets from multiple sources

» Understand the process of seeking and using information on financial funding requirements

- Understand the need for timely decision making with regard to financial shortfalls
- Understand the need for budget tracking for logistics activities according to established procedures
- Understand the need for analysing of financial statements and explaining components of a balance sheet and income statement
- Understand interactive decision support models that allow the development of multiple scenarios and demonstrating the sensitivity of multiple independent variables
- Examine the success or failure of a business using financial accounting
- Understand the calculations of the total system cost of delivering a product or service to the customer
- Compare the key considerations for developing a business plan
- Examine the structure against which business performance will be monitored (e.g., cost centres, chart of accounts)
- Interpret key performance indicators used to monitor business performance (e.g., turnover, profitability)
- Compare strategies for minimising the risk of fraudulent activity (e.g., reconciliation of purchase orders, receipts and payment approvals; dual signatory arrangements for funds transfers)
- Understand investigative processes and options for confirming the existence of fraudulent activity (e.g., sample audits, forensic accounting services)
- Understand the process of preparing funding applications for trusts and grants
- Understand the need to ensure financial transparency
- Compare the general monetary value of medicines and equipment
- Understand the need to secure financing and managing budgets to support distribution operations
- Compare the ability to translate financial information into SC language
- Understand SC finance

6.1 Demonstrate generic skills (e.g., literacy, numeracy, technology)

6.1.1 Exhibit high understanding of literacy and numeracy

- Speak clearly and confidently, organize information in a logical manner, and consider voice tone and pace
- Respond to verbal messages and other cues (active listening)
- Express ideas and opinions clearly in written and verbal form
- Communicate information accurately, concisely and confidently in writing and verbally
- Practice a level of mathematics suitable to the job held
- Compare visual presentation techniques, including charting, histograms and flow sheets
- Read and comprehend at a level necessary to properly complete duties of the position

	» Understand the need to interpret written information in documents, such as reports, SOPs, LMIS forms, graphs, calendars, schedules, notices and directions<br>» Understand the need to pay attention to detail and identify the main ideas, detect inconsistencies and identify missing information in documents<br>» Enable computer skills — such as using Word, Excel, PowerPoint and the internet — suitable to the job level<br>» Understand the process of analysing and validating KPIs<br>» Understand how the evaluation process of activities that act as standards against which the KPIs are measured is carried out<br>» Examine the process of performing critical self-review on work before submission
6.2 Demonstrate strong communication skills	» Be truthful and trustworthy, and supply accurate information at all times<br>» Enable working as part of a workplace team<br>» Understand vocabulary and communication style and form for both written and verbal communication that is appropriate for the situation, audience and material being communicated (e.g., avoid unnecessary jargon, clearly explain medical and SCM terminology)<br>» Enable open communication within constraints of confidentiality<br>» Participate in the organisation of communications about logistics<br>» Enable participation in meetings, expressing one's opinions, being aware of others' needs, and being appropriately assertive when required<br>» Show perspective into the point of view of others, understanding their needs and goals<br>» Enable the practise of building trust, rapport and credibility with others<br>» Understand the communication needs and concerns of others and respond to them<br>» Understand the need to avoid conflict between work and personal interests<br>» Enable meaningful two-way communication<br>» Show influence on others by persuasively presenting thoughts and ideas<br>» Show assertiveness skills to deal with unreasonable requests and/or refusals that would compromise practice or consumer care<br>» Maintain a positive, supportive and appreciative attitude<br>» Show the ability to balance the organisation's needs and the customer's needs<br>» Show self-control by maintaining composure and keeping emotions in check, even in difficult situations; deal calmly and effectively with stressful situations<br>» Enable expressing of opinions and providing information in written and/or verbal form in a manner that does not elicit concern, anger or other adverse response<br>» Enable the use of a systematic process for following up that demonstrates written reports have been received and understood

	» Understand the means by which responses to input to the work environment are monitored<br>» Seek practical ways to overcome barriers to communication<br>» Understand the need to ensuring language representation (e.g., interpreters) at meetings when appropriate<br>» Interpret difficult situations and disputes between staff that need to be tackled and resolved<br>» Enable giving both positive and negative feedback sensitively<br>» Understand the need to maintain and improve communication<br>» Examine the process of giving and requesting feedback frequently<br>» Examine effective communication with shop stewards and union representatives
6.2.1 Practice cultural awareness	» Understand the need for cultural awareness and sensitivity, treating all people with fairness, respect and dignity<br>» Enable anti-discriminatory practices in the organisation, including HR and disciplinary procedures<br>» Enable cultural sensitivity, equality and fairness at all levels of the organisation<br>» Challenge discriminatory behaviour directly and sensitively<br>» Act in a non-discriminatory way toward individuals and groups<br>» Enable integrating cultural awareness in learning and development approaches<br>» Avoid stereotypical responses by examining one's own behaviour and bias<br>» Enable managing cultural diversity in teams and make the most of differences<br>» Compare strategies and/or resources for communicating effectively with people from different cultural backgrounds<br>» Enable the process to elicit information relating to values, beliefs and cultural backgrounds of consumers that may influence the way professional services are provided
Basal Technology Competency(ies):	7.1 Data Science<br>7.4 Temperature and monitoring<br>7.5 Planning systems<br>7.6 ERP includes function of LMIS<br>7.12 Basic Office Skills<br>7.13 Have a command of technology
7.1 Data Science	» Understand end to end MIS technology components that are inherent in advanced MIS systems and explore how these may be relevant in different supply chain contexts<br>» Compare emerging technological approaches to data visualization and analysis

	» Awareness of analytics (data interpretation and decision making)<br>» Enable the ability to interact, provide guidance for data (dashboard) consolidation in various levels and use the tech tools<br>» Support movement of data into electronic form at lowest/remote SC level<br>» Enable the ability to work with relational database
7.4 Temperature and monitoring	» Compare the current technology approaches for Temperature Monitoring Sensors<br>» Consider what is required to implement this technology in a country context<br>» Compare infrastructure requirement for heat-sensitive items at peripheral ‘lower level’ of SC depending on volume and nature of items to handle;<br>» Examine regular preventive maintenance and calibration of cold-chain equipment with biomedical engineers or logisticians<br>» Understand the policy for temperature monitoring tools during transportation and storage<br>» Understand the steps involved in health logistic management cycle: selection-order-delivery-reception-storage-distribution-inventory<br>» Understand hardware qualification and system validation of temperature monitoring<br>» Understand mass data management of temperature monitoring
7.5 Planning systems	» Examine blockchain and understand the building blocks for planning systems<br>» Examine planning systems as a value-add capability for relevant global health use cases
7.6 ERP includes function of LMIS	» Enable a Master Data Management System<br>» Understand the critical components of an ERP system<br>» Understand the meaning of common document types found within an ERP/LMIS<br>» Understand the logic of data visibility within an ERP<br>» Understand the importance of data integrity, record management and information security
7.12 Basic Office Skills	» Have a good understanding of common presentation authoring packages<br>» Have a good understanding of common spreadsheet authoring packages<br>» Have a good understanding of common document authoring packages<br>» Have a good understanding of common email authoring packages<br>» Have a good understanding of common instant messaging packages

	» Have a good understanding of common video conferencing authoring packages
**7.13 Have a command of technology**	» Enable the use of technology suitable to the job held<br>» Understand the need of monitoring new developments and technologies in the sector<br>» Compare new technologies and recognising potential benefits for the sector<br>» Understand that resources and support are provided across the organisation to enable colleagues to make the best use of available technology<br>» Understand that the organisation has a strategy for technology use<br>» Compare how to minimise environmental damage through technology use<br>» Enable the use of technology to maximise effectiveness and efficiency<br>» Enable the use of field-based technology (e.g., radio, general packet radio service (GPRS), satellite phone)
Advanced Technology Competency(ies):	7.2 Blockchain<br>7.3 Unmanned Aerial Vehicles (UAVs)<br>7.7 Automation<br>7.8 Artificial Intelligence<br>7.9 Additive Manufacturing<br>7.10 Internet of things<br>7.11 Cloud Computing<br>7.14 eProcurement
**7.2 Blockchain**	» Examine blockchain as a value-add capability for relevant global health use cases
**7.3 Unmanned Aerial Vehicles (UAVs)**	» Understand the current UAV landscape, their application and the enabling environment required to support country implementation<br>» Understand UAVs as a value-add capability for relevant global health use cases<br>» Understand the laws/ regulations around the use of UAV's
**7.7 Automation**	» Understand automation as a value-add capability for relevant global health use cases
**7.8 Artificial Intelligence**	» Understand artificial intelligence as a value-add capability for relevant global health use cases

7.9 Additive Manufacturing	» Understand additive manufacturing as a value-add capability for relevant global health use cases
7.10 Internet of things (IoT)	» Understand the opportunity of IoT used in conjunction with AI<br>» Understand the internet of things as a value-add capability for relevant global health use cases
7.11 Cloud Computing	» Understand cloud computing as a value-add capability for relevant global health use cases
7.14 eProcurement	» Understand how to transact with an eProcurement system where necessary<br>» Understand eProcurement as a value-add capability for relevant global health use cases<br>» Understand the benefits and limitations of eProcurement to best utilise the system

Key Performance Indicators:

- » Assess Delivery Performance Cycle Time - Actual Time from assessed requirement to time delivered to end-user compared to committed Delivery Cycle Time as per Supply Chain strategy
- » Assess Supplier Performance Cycle Time - Actual Supplier performance Cycle time versus Supplier commitment cycle time
- » Inventory accuracy - Cycle count results
- » Transportation lead time accuracy - Actual Transportation lead time versus Transportation promised lead time.
- » Transport efficiency - Total cost of transport versus planned cost of transport by mode
- » In-bound cost of acquisition - Actual total cost of acquisition versus Total Planned cost of acquisition including landed costs
- » Manage performance cycle time
- » Performance Report - Actual versus Plan reports for all metric

Training:	Qualifications Available:	Certifications Available:
» Data management<br>» ERP Systems<br>» Installed base management<br>» Interpreting Specifications<br>» Office automation tools<br>» Product Information Management (Product Data Management)		» Manage Logistics Operations skills programme<br>» Lean Six Sigma Certification in Finance white belt

Tactical
Assistant – Storage

Organisation: SC-focused Organisation				
Competency Level:	Serial Number:	Primary Process:	Job Role:	Supervises:
Tactical	3-33-25-1	Deliver	Assistant – Storage	-
Contributions	Performs the actual receiving, storage and disposition in accordance with the protocols governing the items being returned.			
Technical Competency(ies):	3.1 Undertake storage, warehousing and inventory management<br>3.1.1 Manage storage of commodities during emergency<br>3.3 Supply commodities to sections within a facility			

3.1 Undertake storage, warehousing and inventory management

» Execute distribution of products among suppliers, distribution centres, warehouses and customers through a logistics network

» Be aware of a total systems approach to designing and managing the entire flow of information, materials, and services

» Awareness of the choice of equipment or materials based on suitability for intended use, accuracy, safety of use and cost

» Awareness of the formal storage locations in the warehouse that identify the row, rack section, level and shelf location, typically with an alphanumeric location bar code or label

» Store medicines appropriately, considering temperature, access and cleanliness

» Ability to maintain the cold chain

» Execute the movement and storage of materials within a warehouse

» Be aware of the need to secure the medical store and limit access to staff

» Follow organisation processes, instructions, rules and parameters for warehouse and inventory management and use of equipment (e.g., annual stock turns, expiry date tracking, stock procurement, rotation and retrieval, equipment maintenance)

» Awareness of policies and procedures for stock handling, distribution and withdrawal consistent with maintaining safety (e.g., policies for distribution of concentrated electrolyte solutions and cytotoxics, identification of cytotoxic drug products within the work environment, separation of like-named or like-packaged products)

» Confirm information on the supplies being stored

» Awareness that one must consolidate several items into larger units for fewer handlings

» Awareness of methods of stock rotation (e.g., first in first out, FIFO, or first to expire first out, FEFO)

	» Enter the quantity received and update the warehouse/inventory/LMIS system software with the stock on hand when each order is delivered according to requisite SOP's<br>» Awareness that there are required levels of stock and/or equipment in specific situations<br>» Awareness of the importance of accurate verification of rolling stocks<br>» Awareness of the importance of monitoring the location and condition of the supplies being stored<br>» Awareness of the importance of the information on the storage locations and facilities<br>» Awareness of the need to communicate any problems in using the storage locations and facilities
3.1.1 Manage storage of commodities during emergency	» Awareness of the types of warehouses available for emergency or disaster supply<br>» Awareness of the choice of storage site for emergency or disaster supply<br>» Awareness of the staff required for the storage of emergency or disaster supply<br>» Awareness of the equipment and material required for the warehousing of emergency or disaster supply<br>» Awareness of the need for a rapid assessment of logistical needs in emergencies
3.3 Supply commodities to sections within a facility	» Awareness of the use of an imprest list<br>» Awareness of the process of validating the requisition from different health care services within the facility
Personal/Management Competency(ies):	6.1 Demonstrate generic skills (e.g., literacy, numeracy, technology)<br>6.1.1 Exhibit high understanding of literacy and numeracy<br>6.2 Demonstrate strong communication skills<br>6.2.1 Practice cultural awareness
6.1 Demonstrate generic skills (e.g., literacy, numeracy, technology)	
6.1.1 Exhibit high understanding of literacy and numeracy	» Speak clearly and confidently, organize information in a logical manner, and consider voice tone and pace<br>» Respond to verbal messages and other cues (active listening)<br>» Express ideas and opinions clearly in written and verbal form<br>» Communicate information accurately, concisely and confidently in writing and verbally<br>» Practice a level of mathematics suitable to the job held<br>» Awareness of visual presentation techniques, including charting, histograms and flow sheets<br>» Read and comprehend at a level necessary to properly complete duties of the position

	» Awareness of the need to interpret written information in documents, such as reports, SOPs, LMIS forms, graphs, calendars, schedules, notices and directions<br>» Awareness of the need to paying attention to detail and identifying the main ideas, detecting inconsistencies and identifying missing information in documents<br>» Execute computer skills — such as using Word, Excel, PowerPoint and the internet — suitable to the job level<br>» Operational knowledge of analysing and validating KPIs<br>» Awareness of how the evaluation process of activities that act as standards against which the KPIs are measured is carried out<br>» Execute the process of performing critical self-review on work before submission
6.2 Demonstrate strong communication skills	» Be truthful and trustworthy, and supply accurate information at all times<br>» Realize the need to work as part of a workplace team<br>» Awareness of vocabulary and communication style and form for both written and verbal communication that is appropriate for the situation, audience and material being communicated (e.g., avoid unnecessary jargon, clearly explain medical and SCM terminology)<br>» Awareness of open communication within constraints of confidentiality<br>» Awareness of communications about logistics<br>» Participate in meetings, expressing one's opinions, being aware of others' needs, and being appropriately assertive when required<br>» Awareness of perspective into the point of view of others, understanding their needs and goals<br>» Build trust, rapport and credibility with others<br>» Awareness of the communication needs and concerns of others and respond to them<br>» Awareness of the need to avoid conflict between work and personal interests<br>» Practice meaningful two-way communication<br>» Realize that others are influenced by persuasively presenting thoughts and ideas<br>» Awareness of the need for assertiveness skills to deal with unreasonable requests and/or refusals that would compromise practice or consumer care<br>» Maintain a positive, supportive and appreciative attitude<br>» Awareness of the need to balance the organisation's needs and the customer's needs<br>» Execute self-control by maintaining composure and keeping emotions in check, even in difficult situations; deal calmly and effectively with stressful situations<br>» Express opinions and provide information in written and/or verbal form in a manner that does not elicit concern, anger or other adverse response<br>» Basic operational knowledge of a systematic process for following up that demonstrates written reports have been received and understood

	» Basic operational knowledge of the means by which responses to input to the work environment are monitored<br>» Seek practical ways to overcome barriers to communication<br>» Awareness of the need for language representation (e.g., interpreters) at meetings when appropriate<br>» Realize that difficult situations and disputes between staff need to be tackled and resolved<br>» Realize the need to give both positive and negative feedback sensitively<br>» Awareness of the need to maintain and improve communication<br>» Execute the process of giving and requesting feedback frequently<br>» Execute effective communication with shop stewards and union representatives
6.2.1 Practice cultural awareness	» Awareness of the need for cultural awareness and sensitivity, treating all people with fairness, respect and dignity<br>» Awareness of the anti-discriminatory practices in the organisation, including HR and disciplinary procedures<br>» Awareness of the need for cultural sensitivity, equality and fairness at all levels of the organisation<br>» Challenge discriminatory behaviour directly and sensitively<br>» Act in a non-discriminatory way toward individuals and groups<br>» Awareness of the need for integrating cultural awareness in learning and development approaches<br>» Avoid stereotypical responses by examining one's own behaviour and bias<br>» Awareness of the need to manage cultural diversity in teams and make the most of differences<br>» Awareness of strategies and/or resources for communicating effectively with people from different cultural backgrounds<br>» Awareness of the need to elicit information relating to values, beliefs and cultural backgrounds of consumers that may influence the way professional services are provided
Basal Technology Competency(ies):	7.6 ERP includes function of LMIS<br>7.13 Have a command of technology
7.6 ERP includes function of LMIS	» Awareness of a Master Data Management System<br>» Awareness of the critical components of an ERP system<br>» Awareness of the meaning of common document types found within an ERP/LMIS<br>» Awareness of the logic of data visibility within an ERP<br>» Awareness of the importance of data integrity, record management and information security
7.13 Have a command of technology	» Execute the use of technology suitable to the job held<br>» Awareness of the need to monitor new developments and technologies in the sector

- Awareness of new technologies and recognising potential benefits for the sector
- Awareness that resources and support are provided across the organisation to enable colleagues to make the best use of available technology
- Awareness of the fact that the organisation has a strategy for technology use
- Awareness of how to minimise environmental damage through technology use
- Basic operational knowledge of technology to maximise effectiveness and efficiency
- Basic operational knowledge of field-based technology (e.g., radio, general packet radio service (GPRS), satellite phone)

Advanced Technology Competency(ies):	7.7 Automation
7.7 Automation	» Awareness of automation as a value-add capability for relevant global health use cases

Key Performance Indicators:

- Availability - Stock out rate - Number of missed line item quantity deliveries due to stock out versus total line item quantity deliveries
- Availability - Stock levels - Current on hand stock versus Planned on hand stock
- Safe product handling - Number of products handled during period out of conformance versus Number of products handled during period expressed as a % with prior period comparisons
- Customer Incidents Report number of incidents by month/week/day comparison with previous periods
- No of Order Lines shipped with incorrectly captured Items - Number of order lines with incorrect items/SKU's versus total number of order lines shipped as a %
- Regulatory - % of procured products that meet Stringent Regulatory Authority (SRA) or WHO standards
- Waste Management - Existence of supply chain management protocols for disposal of medical waste and management of unusable products
- Number of order lines delivered to incorrect end-user - Number of orders incorrectly addressed versus number of orders processed expressed as a %
- Number of order lines delivered out of Quality Specifications - Number of Item/SKU delivered out of specification e.g. Cold Chain infractions, Expired products versus total Number of order lines delivered as a %. A comparative graph would be an appropriate tool for the measurement of this metric.
- % Product Transferred On-Time to Demand Requirement. Actual Number of products transferred outside of the Time Limits vs Number of products Transferred.
- Transfer Product Cycle Time - Actual Transfer Cycle Time versus Planned Transferred Cycle Time
- Accurate pick - Picking Accuracy number of order lines picked inaccurately divided by the total order lines picked (SKU and Quantity)
- On-time delivery - Actual Delivery Date & Time Vs Promised Delivery Data and Time

- Customer order fulfilment cycle time & rate - Actual Order Fulfilment Cycle Time versus Promised Customer Order Fulfilment Cycle time
- Warehousing and Inventory Management OTIF - On time and in full

Training:	Qualifications Available:	Certifications Available:
» Material handling equipment usage		» Occupational Certificate: Store Person<br>» Describe the use of stores and warehousing in operations skills programme<br>» Occupational Certificate: Dispatching and Receiving Clerk

Assistant – Delivery

Organisation: SC-focused Organisation				
Competency Level:	Serial Number:	Primary Process:	Job Role:	Supervises:
Tactical	3-34-61-62-1	Deliver	Assistant – Delivery	-

Contributions	Responsible for dispatching stock from warehouses and storage areas and completing all paperwork required for a successful dispatch according the organisations standard operating procedures.
Technical Competency(ies):	3.4 Manage transport for commodities<br>3.4.1 Manage transport for commodities during disaster

3.4 Manage transport for commodities

» Execute distribution activities, including a fleet of vehicles and distribution schedules, to deliver health commodities to facilities.

» Execute the policies and procedures related to handling and transporting special products such as vaccines, HIV/AIDS drugs, other high value products and narcotics

» Basic operational knowledge of shipping methods, considering trade-offs between costs and benefits

» Awareness of local and national laws/requirements for vehicle safety, driving regulations and licensing

» Execute proper packing and labelling methods for transportation of hazardous materials

» Awareness of the process of maximizing freight loads while minimizing freight costs

» Execute efficient use of transportation resources while meeting customers’ needs

» Execute movement demands with vehicle resources

» Basic operational knowledge of quantitative techniques when solving logistics problems, such as designing routes and scheduling vehicles

» Execute the delivery of products to hospitals and district health offices with the accompanying dispatch note and report and requisition forms

» Execute transport options for order delivery

» Awareness that vehicle availability is due to a vehicle maintenance plan

» List information on distribution requirements

» Awareness of any problems in distribution requirements

» Execute the process of equipping distribution sites

» Awareness of the importance of informing stakeholders for better coordination of distribution

» Awareness of tracking devices (tools and indicators)

» Awareness of the process of training of drivers, van assistants, fleet administrators and expeditors

	» Execute the process of supporting investigations around missed OTIF and other failures
3.4.1 Manage transport for commodities during disaster	» List knowledge of the different types of transport and their characteristics for emergency or disaster supply<br>» Awareness of the process of developing a plan for the achievement of a rapid response logistics plan for deployment in the event of a medical emergency.
Personal/Management Competency(ies):	6.1 Demonstrate generic skills (e.g., literacy, numeracy, technology)<br>6.1.1 Exhibit high understanding of literacy and numeracy<br>6.2 Demonstrate strong communication skills<br>6.2.1 Practice cultural awareness
6.1 Demonstrate generic skills (e.g., literacy, numeracy, technology)	
6.1.1 Exhibit high understanding of literacy and numeracy	» Speak clearly and confidently, organize information in a logical manner, and consider voice tone and pace<br>» Respond to verbal messages and other cues (active listening)<br>» Express ideas and opinions clearly in written and verbal form<br>» Communicate information accurately, concisely and confidently in writing and verbally<br>» Practice a level of mathematics suitable to the job held<br>» Awareness of visual presentation techniques, including charting, histograms and flow sheets<br>» Read and comprehend at a level necessary to properly complete duties of the position<br>» Awareness of the need to interpret written information in documents, such as reports, SOPs, LMIS forms, graphs, calendars, schedules, notices and directions<br>» Awareness of the need to paying attention to detail and identifying the main ideas, detecting inconsistencies and identifying missing information in documents<br>» Execute computer skills — such as using Word, Excel, PowerPoint and the internet — suitable to the job level<br>» Operational knowledge of analysing and validating KPIs<br>» Awareness of how the evaluation process of activities that act as standards against which the KPIs are measured is carried out<br>» Execute the process of performing critical self-review on work before submission
6.2 Demonstrate strong communication skills	» Be truthful and trustworthy, and supply accurate information at all times<br>» Realize the need to work as part of a workplace team<br>» Awareness of vocabulary and communication style and form for both written and verbal communication that is appropriate for the situation,

audience and material being communicated (e.g., avoid unnecessary jargon, clearly explain medical and SCM terminology)

- Awareness of open communication within constraints of confidentiality
- Awareness of communications about logistics
- Participate in meetings, expressing one's opinions, being aware of others' needs, and being appropriately assertive when required
- Awareness of perspective into the point of view of others, understanding their needs and goals
- Build trust, rapport and credibility with others
- Awareness of the communication needs and concerns of others and respond to them
- Awareness of the need to avoid conflict between work and personal interests
- Practice meaningful two-way communication
- Realize that others are influenced by persuasively presenting thoughts and ideas
- Awareness of the need for assertiveness skills to deal with unreasonable requests and/or refusals that would compromise practice or consumer care
- Maintain a positive, supportive and appreciative attitude
- Awareness of the need to balance the organisation's needs and the customer's needs
- Execute self-control by maintaining composure and keeping emotions in check, even in difficult situations; deal calmly and effectively with stressful situations
- Express opinions and provide information in written and/or verbal form in a manner that does not elicit concern, anger or other adverse response
- Basic operational knowledge of a systematic process for following up that demonstrates written reports have been received and understood
- Basic operational knowledge of the means by which responses to input to the work environment are monitored
- Seek practical ways to overcome barriers to communication
- Awareness of the need for language representation (e.g., interpreters) at meetings when appropriate
- Realize that difficult situations and disputes between staff need to be tackled and resolved
- Realize the need to give both positive and negative feedback sensitively
- Awareness of the need to maintain and improve communication
- Execute the process of giving and requesting feedback frequently
- Execute effective communication with shop stewards and union representatives

6.2.1 Practice cultural awareness

- Awareness of the need for cultural awareness and sensitivity, treating all people with fairness, respect and dignity

	» Awareness of the anti-discriminatory practices in the organisation, including HR and disciplinary procedures<br>» Awareness of the need for cultural sensitivity, equality and fairness at all levels of the organisation<br>» Challenge discriminatory behaviour directly and sensitively<br>» Act in a non-discriminatory way toward individuals and groups<br>» Awareness of the need for integrating cultural awareness in learning and development approaches<br>» Avoid stereotypical responses by examining one's own behaviour and bias<br>» Awareness of the need to manage cultural diversity in teams and make the most of differences<br>» Awareness of strategies and/or resources for communicating effectively with people from different cultural backgrounds<br>» Awareness of the need to elicit information relating to values, beliefs and cultural backgrounds of consumers that may influence the way professional services are provided
Basal Technology Competency(ies):	7.4 Temperature and monitoring<br>7.6 ERP includes function of LMIS<br>7.12 Basic Office Skills<br>7.13 Have a command of technology
**7.4 Temperature and monitoring**	» Awareness of the current technology approaches for Temperature monitoring and sensors<br>» Awareness of what is required to implement this technology in a country context<br>» Awareness of infrastructure requirement for heat-sensitive items at peripheral 'lower level' of SC depending on volume and nature of items to handle;<br>» Awareness of regular preventive maintenance and calibration of cold-chain equipment with biomedical engineers or logisticians<br>» Awareness of the policy for temperature monitoring tools during transportation and storage<br>» Awareness of the steps involved in health logistic management cycle: selection-order-delivery-reception-storage-distribution-inventory<br>» Awareness of hardware qualification and system validation of temperature monitoring<br>» Basic operational knowledge mass data management of temperature monitoring
**7.6 ERP includes function of LMIS**	» Awareness of a Master Data Management System<br>» Awareness of the critical components of an ERP system<br>» Awareness of the meaning of common document types found within an ERP/LMIS<br>» Awareness of the logic of data visibility within an ERP

	» Awareness of the importance of data integrity, record management and information security
**7.12 Basic Office Skills**	» Have a good understanding of common presentation authoring packages<br>» Have a good understanding of common spreadsheet authoring packages<br>» Have a good understanding of common document authoring packages<br>» Have a good understanding of common email authoring packages<br>» Have a good understanding of common instant messaging packages<br>» Have a good understanding of common video conferencing authoring packages
**7.13 Have a command of technology**	» Execute the use of technology suitable to the job held<br>» Awareness of the need to monitor new developments and technologies in the sector<br>» Awareness of new technologies and recognising potential benefits for the sector<br>» Awareness that resources and support are provided across the organisation to enable colleagues to make the best use of available technology<br>» Awareness of the fact that the organisation has a strategy for technology use<br>» Awareness of how to minimise environmental damage through technology use<br>» Basic operational knowledge of technology to maximise effectiveness and efficiency<br>» Basic operational knowledge of field-based technology (e.g., radio, general packet radio service (GPRS), satellite phone)
Advanced Technology Competency(ies):	7.7 Automation
**7.7 Automation**	» Awareness of automation as a value-add capability for relevant global health use cases

Key Performance Indicators:

» Availability - Stock out rate - Number of missed line item quantity deliveries due to stock out versus total line item quantity deliveries

»

» Availability - Stock levels - Current on hand stock versus Planned on hand stock

» Safe product handling - Number of products handled during period out of conformance versus Number of products handled during period expressed as a % with prior period comparisons

» Customer Incidents Report number of incidents by month/week/day comparison with previous periods

» No of Order Lines shipped with incorrectly captured Items - Number of order lines with incorrect items/SKU's versus total number of order lines shipped as a %

» Regulatory - % of procured products that meet Stringent Regulatory Authority (SRA) or WHO standards

» Waste Management - Existence of supply chain management protocols for disposal of medical waste and management of unusable products

» Number of order lines delivered to incorrect end-user - Number of orders incorrectly addressed versus number of orders processed expressed as a %

» Number of Order Lines delivered out of Quality Specifications - Number of Item/SKU delivered out of specification e.g. Cold Chain infractions, Expired products versus total Number of order lines delivered as a %. A comparative graph would be an appropriate tool for the measurement of this metric.

» % Product Transferred On-Time to Demand Requirement. Actual Number of products transferred outside of the Time Limits vs Number of products Transferred.

» Transfer Product Cycle Time - Actual Transfer Cycle Time versus Planned Transferred Cycle Time

» Accurate pick - Picking Accuracy number of order lines picked inaccurately divided by the total order lines picked (SKU and Quantity)

» On-time delivery - Actual Delivery Date & Time Vs Promised Delivery Data and Time

» Customer order fulfilment cycle time & rate - Actual Order Fulfilment Cycle Time versus Promised Customer Order Fulfilment Cycle time

» Warehousing and Inventory Management OTIF - On time and in full

Training:	Qualifications Available:	Certifications Available:
» Vehicle operation<br>» Material handling	» Diploma in Supply Chain Management and Logistics	» Describe the use of stores and warehousing in operations skills programme

Assistant – Inventory

Organisation:

SC-focused Organisation

Competency Level:	Serial Number:	Primary Process:	Job Role:	Supervises:
Tactical	3-31-32-61-62-1	Deliver	Assistant – Inventory	-

Contributions	Responsible for carrying out cycle counts within storage areas and reporting deviations from standard to supervisors
Technical Competency(ies):	3.1 Undertake storage, warehousing and inventory management<br>3.1.1 Manage storage of commodities during emergency<br>3.2 Supply commodities to facilities

3.1 Undertake storage, warehousing and inventory management

- Execute distribution of products among suppliers, distribution centres, warehouses and customers through a logistics network
- Be aware of a total systems approach to designing and managing the entire flow of information, materials, and services
- Awareness of the choice of equipment or materials based on suitability for intended use, accuracy, safety of use and cost
- Awareness of the formal storage locations in the warehouse that identify the row, rack section, level and shelf location, typically with an alphanumeric location bar code or label
- Store medicines appropriately, considering temperature, access and cleanliness
- Ability to maintain the cold chain
- Execute the movement and storage of materials within a warehouse
- Be aware of the need to secure the medical store and limit access to staff
- Follow organisation processes, instructions, rules and parameters for warehouse and inventory management and use of equipment (e.g., annual stock turns, expiry date tracking, stock procurement, rotation and retrieval, equipment maintenance)
- Awareness of policies and procedures for stock handling, distribution and withdrawal consistent with maintaining safety (e.g., policies for distribution of concentrated electrolyte solutions and cytotoxics, identification of cytotoxic drug products within the work environment, separation of like-named or like-packaged products)
- Confirm information on the supplies being stored
- Awareness that one must consolidate several items into larger units for fewer handlings
- Awareness of methods of stock rotation (e.g., first in first out, FIFO, or first to expire first out, FEFO)
- Enter the quantity received and update the warehouse/inventory/LMIS system software with the stock on hand when each order is delivered according to requisite SOP's

	» Awareness that there are required levels of stock and/or equipment in specific situations<br>» Awareness of the importance of accurate verification of rolling stocks<br>» Awareness of the importance of monitoring the location and condition of the supplies being stored<br>» Awareness of the importance of the information on the storage locations and facilities<br>» Awareness of the need to communicate any problems in using the storage locations and facilities
3.1.1 Manage storage of commodities during emergency	» Awareness of the types of warehouses available for emergency or disaster supply<br>» Awareness of the choice of storage site for emergency or disaster supply<br>» Awareness of the staff required for the storage of emergency or disaster supply<br>» Awareness of the equipment and material required for the warehousing of emergency or disaster supply<br>» Awareness of the need for a rapid assessment of logistical needs in emergencies
3.2 Supply commodities to facilities	» Working knowledge of the various sources of distribution demand (customers)<br>» Basic operational knowledge of order policies for planned order generation<br>» Execute re-supply quantities using a variety of inventory methods, including visual review, two bin, periodic review, order point and just in time<br>» Basic knowledge of the use of order schedules<br>» Basic operational knowledge of safety stocks<br>» Awareness of the principles of EOQ in the ordering process<br>» Awareness of pull and push inventory control systems<br>» Execute a regular, rather than urgent, order culture<br>» Execute order-filling priorities in relation to delivery opportunities and urgency<br>» Awareness that orders must be screened (modify order quantities on the basis of available stock, impact on service delivery, distance of facility from hospital)<br>» Assemble, check, pick and pack orders<br>» Execute dispatch lists and invoices<br>» Awareness of the importance of the balancing of supply with demand, considering both lead time and demand variability created by supply patterns not matching demand patterns<br>» Basic operational knowledge on the storage locations and facilities<br>» Awareness of the measures of customer satisfaction by using performance metrics taken from the customer perspective, with criteria such as on-time delivery, perception of quality, complaints and length of wait times
Personal/Management Competency(ies):	6.1 Demonstrate generic skills (e.g., literacy, numeracy, technology)<br>6.1.1 Exhibit high understanding of literacy and numeracy<br>6.2 Demonstrate strong communication skills<br>6.2.1 Practice cultural awareness

6.1 Demonstrate generic skills (e.g., literacy, numeracy, technology)	
6.1.1 Exhibit high understanding of literacy and numeracy	» Speak clearly and confidently, organize information in a logical manner, and consider voice tone and pace<br>» Respond to verbal messages and other cues (active listening)<br>» Express ideas and opinions clearly in written and verbal form<br>» Communicate information accurately, concisely and confidently in writing and verbally<br>» Practice a level of mathematics suitable to the job held<br>» Awareness of visual presentation techniques, including charting, histograms and flow sheets<br>» Read and comprehend at a level necessary to properly complete duties of the position<br>» Awareness of the need to interpret written information in documents, such as reports, SOPs, LMIS forms, graphs, calendars, schedules, notices and directions<br>» Awareness of the need to paying attention to detail and identifying the main ideas, detecting inconsistencies and identifying missing information in documents<br>» Execute computer skills — such as using Word, Excel, PowerPoint and the internet — suitable to the job level<br>» Operational knowledge of analysing and validating KPIs<br>» Awareness of how the evaluation process of activities that act as standards against which the KPIs are measured is carried out<br>» Execute the process of performing critical self-review on work before submission
6.2 Demonstrate strong communication skills	» Be truthful and trustworthy, and supply accurate information at all times<br>» Realize the need to work as part of a workplace team<br>» Awareness of vocabulary and communication style and form for both written and verbal communication that is appropriate for the situation, audience and material being communicated (e.g., avoid unnecessary jargon, clearly explain medical and SCM terminology)<br>» Awareness of open communication within constraints of confidentiality<br>» Awareness of communications about logistics<br>» Participate in meetings, expressing one's opinions, being aware of others' needs, and being appropriately assertive when required<br>» Awareness of perspective into the point of view of others, understanding their needs and goals<br>» Build trust, rapport and credibility with others<br>» Awareness of the communication needs and concerns of others and respond to them<br>» Awareness of the need to avoid conflict between work and personal interests<br>» Practice meaningful two-way communication<br>» Realize that others are influenced by persuasively presenting thoughts and ideas

	» Awareness of the need for assertiveness skills to deal with unreasonable requests and/or refusals that would compromise practice or consumer care<br>» Maintain a positive, supportive and appreciative attitude<br>» Awareness of the need to balance the organisation's needs and the customer's needs<br>» Execute self-control by maintaining composure and keeping emotions in check, even in difficult situations; deal calmly and effectively with stressful situations<br>» Express opinions and provide information in written and/or verbal form in a manner that does not elicit concern, anger or other adverse response<br>» Basic operational knowledge of a systematic process for following up that demonstrates written reports have been received and understood<br>» Basic operational knowledge of the means by which responses to input to the work environment are monitored<br>» Seek practical ways to overcome barriers to communication<br>» Awareness of the need for language representation (e.g., interpreters) at meetings when appropriate<br>» Realize that difficult situations and disputes between staff need to be tackled and resolved<br>» Realize the need to give both positive and negative feedback sensitively<br>» Awareness of the need to maintain and improve communication<br>» Execute the process of giving and requesting feedback frequently<br>» Execute effective communication with shop stewards and union representatives
6.2.1 Practice cultural awareness	» Awareness of the need for cultural awareness and sensitivity, treating all people with fairness, respect and dignity<br>» Awareness of the anti-discriminatory practices in the organisation, including HR and disciplinary procedures<br>» Awareness of the need for cultural sensitivity, equality and fairness at all levels of the organisation<br>» Challenge discriminatory behaviour directly and sensitively<br>» Act in a non-discriminatory way toward individuals and groups<br>» Awareness of the need for integrating cultural awareness in learning and development approaches<br>» Avoid stereotypical responses by examining one's own behaviour and bias<br>» Awareness of the need to manage cultural diversity in teams and make the most of differences<br>» Awareness of strategies and/or resources for communicating effectively with people from different cultural backgrounds<br>» Awareness of the need to elicit information relating to values, beliefs and cultural backgrounds of consumers that may influence the way professional services are provided
Basal Technology Competency(ies):	7.6 ERP includes function of LMIS<br>7.13 Have a command of technology

7.6 ERP includes function of LMIS	» Awareness of a Master Data Management System<br>» Awareness of the critical components of an ERP system<br>» Awareness of the meaning of common document types found within an ERP/LMIS<br>» Awareness of the logic of data visibility within an ERP<br>» Awareness of the importance of data integrity, record management and information security
7.13 Have a command of technology	» Execute the use of technology suitable to the job held<br>» Awareness of the need to monitor new developments and technologies in the sector<br>» Awareness of new technologies and recognising potential benefits for the sector<br>» Awareness that resources and support are provided across the organisation to enable colleagues to make the best use of available technology<br>» Awareness of the fact that the organisation has a strategy for technology use<br>» Awareness of how to minimise environmental damage through technology use<br>» Basic operational knowledge of technology to maximise effectiveness and efficiency<br>» Basic operational knowledge of field-based technology (e.g., radio, general packet radio service (GPRS), satellite phone)
Advanced Technology Competency(ies):	7.7 Automation
7.7 Automation	» Awareness of automation as a value-add capability for relevant global health use cases

Key Performance Indicators:

- » Safe product handling - Number of products handled during period out of conformance versus Number of products handled during period expressed as a % with prior period comparisons
- » Customer Incidents Report number of incidents by month/week/day comparison with previous periods
- » No of Order Lines shipped with incorrectly captured Items - Number of order lines with incorrect items/SKU's versus total number of order lines shipped as a %
- » Number of Order Lines delivered to incorrect end-user -Number of orders incorrectly addressed versus number of orders processed expressed as a %
- » Number of Order Lines delivered out of Quality Specifications - Number of Item/SKU delivered out of specification e.g. Cold Chain infractions, Expired products versus total Number of order lines delivered as a %. A comparative graph would be an appropriate tool for the measurement of this metric.
- » Competency compliance. No of personnel evaluated and found to competent versus plan.
- » % Product Transferred On-Time to Demand Requirement. Actual Number of products transferred outside of the Time Limits vs Number of products Transferred.
- » Transfer Product Cycle Time - Actual Transfer Cycle Time versus Planned Transferred Cycle Time
- » Accurate pick - Picking Accuracy number of order lines picked inaccurately divided by the total order lines picked (SKU and Quantity)

» On-time delivery - Actual Delivery Date & Time Vs Promised Delivery Data and Time
» Customer order fulfilment cycle time & rate - Actual Order Fulfilment Cycle Time versus Promised Customer Order Fulfilment Cycle time
» Availability - Stock out rate - Number of missed line item quantity deliveries due to stock out versus total line item quantity deliveries
» Availability - Stock levels - Current on hand stock versus Planned on hand stock
» Regulatory - % of procured products that meet Stringent Regulatory Authority (SRA) or WHO standards
» Warehousing and Inventory Management OTIF - On time and in full
» Waste Management - Existence of supply chain management protocols for disposal of medical waste and management of unusable products
» Line Items SKU's handled vs Items / SKU's Planned over a planning horizon
» Number of warehouse locations used versus warehouse locations available (Pallet Positions may be used in larger warehouses)
» Warehouse Utilization - Volume used vs Volume Available

Training:	Qualifications Available:	Certifications Available:
» Inventory Management<br>» Inventory housekeeping<br>» Cycle counting		» Occupational Certificate: Store Person<br>» Occupational Certificate: Dispatching and Receiving Clerk<br>» Discuss the role of stocktaking in ensuring inventory accuracy skills programme

Assistant – Pick, Pack

Organisation: **SC-focused Organisation**				
Competency Level:	Serial Number:	Primary Process:	Job Role:	Supervises:
Tactical	3-32-61-62-1	Deliver	Assistant – Pick, Pack	-

Contributions	Perform Pick, Pack operations in accordance with work instructions, accurately and timeously. Record transactions speedily.
Technical Competency(ies):	3.2 Supply commodities to facilities

3.2 Supply commodities to facilities

- Working knowledge of the various sources of distribution demand (customers)
- Basic operational knowledge of order policies for planned order generation
- Execute re-supply quantities using a variety of inventory methods, including visual review, two bin, periodic review, order point and just in time
- Basic knowledge of the use of order schedules
- Basic operational knowledge of safety stocks
- Awareness of the principles of EOQ in the ordering process
- Awareness of pull and push inventory control systems
- Execute a regular, rather than urgent, order culture
- Execute order-filling priorities in relation to delivery opportunities and urgency
- Awareness that orders must be screened (modify order quantities on the basis of available stock, impact on service delivery, distance of facility from hospital)
- Assemble, check, pick and pack orders
- Execute dispatch lists and invoices
- Awareness of the importance of the balancing of supply with demand, considering both lead time and demand variability created by supply patterns not matching demand patterns
- Basic operational knowledge on the storage locations and facilities
- Awareness of the measures of customer satisfaction by using performance metrics taken from the customer perspective, with criteria such as on-time delivery, perception of quality, complaints, and length of wait times

Personal/Management Competency(ies):	6.1 Demonstrate generic skills (e.g., literacy, numeracy, technology)<br>6.1.1 Exhibit high understanding of literacy and numeracy<br>6.2 Demonstrate strong communication skills<br>6.2.1 Practice cultural awareness

6.1 Demonstrate generic skills (e.g., literacy, numeracy, technology)

6.1.1 Exhibit high understanding of literacy and numeracy	» Speak clearly and confidently, organize information in a logical manner, and consider voice tone and pace<br>» Respond to verbal messages and other cues (active listening)<br>» Express ideas and opinions clearly in written and verbal form<br>» Communicate information accurately, concisely and confidently in writing and verbally<br>» Practice a level of mathematics suitable to the job held<br>» Awareness of visual presentation techniques, including charting, histograms and flow sheets<br>» Read and comprehend at a level necessary to properly complete duties of the position<br>» Awareness of the need to interpret written information in documents, such as reports, SOPs, LMIS forms, graphs, calendars, schedules, notices and directions<br>» Awareness of the need to paying attention to detail and identifying the main ideas, detecting inconsistencies and identifying missing information in documents<br>» Execute computer skills — such as using Word, Excel, PowerPoint and the internet — suitable to the job level<br>» Operational knowledge of analysing and validating KPIs<br>» Awareness of how the evaluation process of activities that act as standards against which the KPIs are measured is carried out<br>» Execute the process of performing critical self-review on work before submission
6.2 Demonstrate strong communication skills	» Be truthful and trustworthy, and supply accurate information at all times<br>» Realize the need to work as part of a workplace team<br>» Awareness of vocabulary and communication style and form for both written and verbal communication that is appropriate for the situation, audience and material being communicated (e.g., avoid unnecessary jargon, clearly explain medical and SCM terminology)<br>» Awareness of open communication within constraints of confidentiality<br>» Awareness of communications about logistics<br>» Participate in meetings, expressing one's opinions, being aware of others' needs, and being appropriately assertive when required<br>» Awareness of perspective into the point of view of others, understanding their needs and goals<br>» Build trust, rapport and credibility with others<br>» Awareness of the communication needs and concerns of others and respond to them<br>» Awareness of the need to avoid conflict between work and personal interests<br>» Practice meaningful two-way communication<br>» Realize that others are influenced by persuasively presenting thoughts and ideas

	» Awareness of the need for assertiveness skills to deal with unreasonable requests and/or refusals that would compromise practice or consumer care<br>» Maintain a positive, supportive and appreciative attitude<br>» Awareness of the need to balance the organisation's needs and the customer's needs<br>» Execute self-control by maintaining composure and keeping emotions in check, even in difficult situations; deal calmly and effectively with stressful situations<br>» Express opinions and provide information in written and/or verbal form in a manner that does not elicit concern, anger or other adverse response<br>» Basic operational knowledge of a systematic process for following up that demonstrates written reports have been received and understood<br>» Basic operational knowledge of the means by which responses to input to the work environment are monitored<br>» Seek practical ways to overcome barriers to communication<br>» Awareness of the need for language representation (e.g., interpreters) at meetings when appropriate<br>» Realize that difficult situations and disputes between staff need to be tackled and resolved<br>» Realize the need to give both positive and negative feedback sensitively<br>» Awareness of the need to maintain and improve communication<br>» Execute the process of giving and requesting feedback frequently<br>» Execute effective communication with shop stewards and union representatives
6.2.1 Practice cultural awareness	» Awareness of the need for cultural awareness and sensitivity, treating all people with fairness, respect and dignity<br>» Awareness of the anti-discriminatory practices in the organisation, including HR and disciplinary procedures<br>» Awareness of the need for cultural sensitivity, equality and fairness at all levels of the organisation<br>» Challenge discriminatory behaviour directly and sensitively<br>» Act in a non-discriminatory way toward individuals and groups<br>» Awareness of the need for integrating cultural awareness in learning and development approaches<br>» Avoid stereotypical responses by examining one's own behaviour and bias<br>» Awareness of the need to manage cultural diversity in teams and make the most of differences<br>» Awareness of strategies and/or resources for communicating effectively with people from different cultural backgrounds<br>» Awareness of the need to elicit information relating to values, beliefs and cultural backgrounds of consumers that may influence the way professional services are provided
Basal Technology Competency(ies):	7.6 ERP includes function of LMIS<br>7.13 Have a command of technology

7.6 ERP includes function of LMIS	» Awareness of a Master Data Management System<br>» Awareness of the critical components of an ERP system<br>» Awareness of the meaning of common document types found within an ERP/LMIS<br>» Awareness of the logic of data visibility within an ERP<br>» Awareness of the importance of data integrity, record management and information security
7.13 Have a command of technology	» Execute the use of technology suitable to the job held<br>» Awareness of the need to monitor new developments and technologies in the sector<br>» Awareness of new technologies and recognising potential benefits for the sector<br>» Awareness that resources and support are provided across the organisation to enable colleagues to make the best use of available technology<br>» Awareness of the fact that the organisation has a strategy for technology use<br>» Awareness of how to minimise environmental damage through technology use<br>» Basic operational knowledge of technology to maximise effectiveness and efficiency<br>» Basic operational knowledge of field-based technology (e.g., radio, general packet radio service (GPRS), satellite phone)
Advanced Technology Competency(ies):	7.7 Automation
7.7 Automation	» Awareness of automation as a value-add capability for relevant global health use cases

Key Performance Indicators:

- Customer Incidents Report number of incidents by month/week/day comparison with previous periods
- No of Order Lines shipped with incorrectly captured Items - Number of order lines with incorrect items/SKU's versus total number of order lines shipped as a %
- Accurate pick - Picking Accuracy number of order lines picked inaccurately divided by the total order lines picked (SKU and Quantity)
- On-time delivery - Actual Delivery Date & Time Vs Promised Delivery Data and Time
- Customer order fulfilment cycle time & rate - Actual Order Fulfilment Cycle Time versus Promised Customer Order Fulfilment Cycle time

Training:	Qualifications Available:	Certifications Available:
» Order Management<br>» Material Handling<br>» Picking<br>» Packing		» Occupational Certificate: Store Person<br>» Pick stock in a DC warehouse skills programme

Assistant – Receiving

Organisation: SC-focused Organisation				
Competency Level:	Serial Number:	Primary Process:	Job Role:	Supervises:
Tactical	3-31-311-34-1	Procure	Assistant – Receiving	-

Contributions	Accepts goods received and performs put away operations in compliance with the rules governing the organisation of inventory. Assists the Officer - Receiving with executing the receiving process within the regulatory and ethical constraints
Technical Competency(ies):	3.1 Undertake storage, warehousing and inventory management<br>3.1.1 Manage storage of commodities during emergency<br>3.4 Manage transport for commodities<br>3.4.1 Manage transport for commodities during disaster

3.1 Undertake storage, warehousing and inventory management

- Execute distribution of products among suppliers, distribution centres, warehouses and customers through a logistics network
- Be aware of a total systems approach to designing and managing the entire flow of information, materials, and services
- Awareness of the choice of equipment or materials based on suitability for intended use, accuracy, safety of use and cost
- Awareness of the formal storage locations in the warehouse that identify the row, rack section, level and shelf location, typically with an alphanumeric location bar code or label
- Store medicines appropriately, considering temperature, access and cleanliness
- Ability to maintain the cold chain
- Execute the movement and storage of materials within a warehouse
- Be aware of the need to secure the medical store and limit access to staff
- Follow organisation processes, instructions, rules and parameters for warehouse and inventory management and use of equipment (e.g., annual stock turns, expiry date tracking, stock procurement, rotation and retrieval, equipment maintenance)
- Awareness of policies and procedures for stock handling, distribution and withdrawal consistent with maintaining safety (e.g., policies for distribution of concentrated electrolyte solutions and cytotoxics, identification of cytotoxic drug products within the work environment, separation of like-named or like-packaged products)
- Confirm information on the supplies being stored

	» Awareness that one must consolidate several items into larger units for fewer handlings<br>» Awareness of methods of stock rotation (e.g., first in first out, FIFO, or first to expire first out, FEFO)<br>» Enter the quantity received and update the warehouse/inventory/LMIS system software with the stock on hand when each order is delivered according to requisite SOP's<br>» Awareness that there are required levels of stock and/or equipment in specific situations<br>» Awareness of the importance of accurate verification of rolling stocks<br>» Awareness of the importance of monitoring the location and condition of the supplies being stored<br>» Awareness of the importance of the information on the storage locations and facilities<br>» Awareness of the need to communicate any problems in using the storage locations and facilities
3.1.1 Manage storage of commodities during emergency	» Awareness of the types of warehouses available for emergency or disaster supply<br>» Awareness of the choice of storage site for emergency or disaster supply<br>» Awareness of the staff required for the storage of emergency or disaster supply<br>» Awareness of the equipment and material required for the warehousing of emergency or disaster supply<br>» Awareness of the need for a rapid assessment of logistical needs in emergencies
3.4 Manage transport for commodities	» Execute distribution activities, including a fleet of vehicles and distribution schedules, to deliver health commodities to facilities.<br>» Execute the policies and procedures related to handling and transporting special products such as vaccines, HIV/AIDS drugs, other high value products and narcotics<br>» Basic operational knowledge of shipping methods, considering trade-offs between costs and benefits<br>» Awareness of local and national laws/requirements for vehicle safety, driving regulations and licensing<br>» Execute proper packing and labelling methods for transportation of hazardous materials<br>» Awareness of the process of maximizing freight loads while minimizing freight costs<br>» Execute efficient use of transportation resources while meeting customers' needs<br>» Execute movement demands with vehicle resources<br>» Basic operational knowledge of quantitative techniques when solving logistics problems, such as designing routes and scheduling vehicles

	» Execute the delivery of products to hospitals and district health offices with the accompanying dispatch note and report and requisition forms<br>» Execute transport options for order delivery<br>» Awareness that vehicle availability is due to a vehicle maintenance plan<br>» List information on distribution requirements<br>» Awareness of any problems in distribution requirements<br>» Execute the process of equipping distribution sites<br>» Awareness of the importance of informing stakeholders for better coordination of distribution<br>» Awareness of tracking devices (tools and indicators)<br>» Awareness of the process of training of drivers, van assistants, fleet administrators and expeditors<br>» Execute the process of supporting investigations around missed OTIF and other failures
3.4.1 Manage transport for commodities during disaster	» List knowledge of the different types of transport and their characteristics for emergency or disaster supply<br>» Awareness of the process of developing a plan for the achievement of a rapid response logistics plan for deployment in the event of a medical emergency.
Personal/Management Competency(ies):	6.1 Demonstrate generic skills (e.g., literacy, numeracy, technology)<br>6.1.1 Exhibit high understanding of literacy and numeracy<br>6.2 Demonstrate strong communication skills<br>6.2.1 Practice cultural awareness
6.1 Demonstrate generic skills (e.g., literacy, numeracy, technology)	
6.1.1 Exhibit high understanding of literacy and numeracy	» Speak clearly and confidently, organize information in a logical manner, and consider voice tone and pace<br>» Respond to verbal messages and other cues (active listening)<br>» Express ideas and opinions clearly in written and verbal form<br>» Communicate information accurately, concisely and confidently in writing and verbally<br>» Practice a level of mathematics suitable to the job held<br>» Awareness of visual presentation techniques, including charting, histograms and flow sheets<br>» Read and comprehend at a level necessary to properly complete duties of the position<br>» Awareness of the need to interpret written information in documents, such as reports, SOPs, LMIS forms, graphs, calendars, schedules, notices and directions

	» Awareness of the need to paying attention to detail and identifying the main ideas, detecting inconsistencies and identifying missing information in documents<br>» Execute computer skills — such as using Word, Excel, PowerPoint and the internet — suitable to the job level<br>» Operational knowledge of analysing and validating KPIs<br>» Awareness of how the evaluation process of activities that act as standards against which the KPIs are measured is carried out<br>» Execute the process of performing critical self-review on work before submission
6.2 Demonstrate strong communication skills	» Be truthful and trustworthy, and supply accurate information at all times<br>» Realize the need to work as part of a workplace team<br>» Awareness of vocabulary and communication style and form for both written and verbal communication that is appropriate for the situation, audience and material being communicated (e.g., avoid unnecessary jargon, clearly explain medical and SCM terminology)<br>» Awareness of open communication within constraints of confidentiality<br>» Awareness of communications about logistics<br>» Participate in meetings, expressing one's opinions, being aware of others' needs, and being appropriately assertive when required<br>» Awareness of perspective into the point of view of others, understanding their needs and goals<br>» Build trust, rapport and credibility with others<br>» Awareness of the communication needs and concerns of others and respond to them<br>» Awareness of the need to avoid conflict between work and personal interests<br>» Practice meaningful two-way communication<br>» Realize that others are influenced by persuasively presenting thoughts and ideas<br>» Awareness of the need for assertiveness skills to deal with unreasonable requests and/or refusals that would compromise practice or consumer care<br>» Maintain a positive, supportive and appreciative attitude<br>» Awareness of the need to balance the organisation's needs and the customer's needs<br>» Execute self-control by maintaining composure and keeping emotions in check, even in difficult situations; deal calmly and effectively with stressful situations<br>» Express opinions and provide information in written and/or verbal form in a manner that does not elicit concern, anger or other adverse response

- Basic operational knowledge of a systematic process for following up that demonstrates written reports have been received and understood
- Basic operational knowledge of the means by which responses to input to the work environment are monitored
- Seek practical ways to overcome barriers to communication
- Awareness of the need for language representation (e.g., interpreters) at meetings when appropriate
- Realize that difficult situations and disputes between staff need to be tackled and resolved
- Realize the need to give both positive and negative feedback sensitively
- Awareness of the need to maintain and improve communication
- Execute the process of giving and requesting feedback frequently
- Execute effective communication with shop stewards and union representatives

6.2.1 Practice cultural awareness

- Awareness of the need for cultural awareness and sensitivity, treating all people with fairness, respect and dignity
- Awareness of the anti-discriminatory practices in the organisation, including HR and disciplinary procedures
- Awareness of the need for cultural sensitivity, equality and fairness at all levels of the organisation
- Challenge discriminatory behaviour directly and sensitively
- Act in a non-discriminatory way toward individuals and groups
- Awareness of the need for integrating cultural awareness in learning and development approaches
- Avoid stereotypical responses by examining one's own behaviour and bias
- Awareness of the need to manage cultural diversity in teams and make the most of differences
- Awareness of strategies and/or resources for communicating effectively with people from different cultural backgrounds
- Awareness of the need to elicit information relating to values, beliefs and cultural backgrounds of consumers that may influence the way professional services are provided

Basal Technology Competency(ies):

7.4 Temperature and monitoring

7.6 ERP includes function of LMIS

7.12 Basic Office Skills

7.13 Have a command of technology

7.4 Temperature and monitoring

- Awareness of the current technology approaches for Temperature monitoring and sensors

	» Awareness of what is required to implement this technology in a country context<br>» Awareness of infrastructure requirement for heat-sensitive items at peripheral ‘lower level’ of SC depending on volume and nature of items to handle;<br>» Awareness of regular preventive maintenance and calibration of cold-chain equipment with biomedical engineers or logisticians<br>» Awareness of the policy for temperature monitoring tools during transportation and storage<br>» Awareness of the steps involved in health logistic management cycle: selection-order-delivery-reception-storage-distribution-inventory<br>» Awareness of hardware qualification and system validation of temperature monitoring<br>» Basic operational knowledge mass data management of temperature monitoring
7.6 ERP includes function of LMIS	» Awareness of a Master Data Management System<br>» Awareness of the critical components of an ERP system<br>» Awareness of the meaning of common document types found within an ERP/LMIS<br>» Awareness of the logic of data visibility within an ERP<br>» Awareness of the importance of data integrity, record management and information security
7.12 Basic Office Skills	» Have a good understanding of common presentation authoring packages<br>» Have a good understanding of common spreadsheet authoring packages<br>» Have a good understanding of common document authoring packages<br>» Have a good understanding of common email authoring packages<br>» Have a good understanding of common instant messaging packages<br>» Have a good understanding of common video conferencing authoring packages
7.13 Have a command of technology	» Execute the use of technology suitable to the job held<br>» Awareness of the need to monitor new developments and technologies in the sector<br>» Awareness of new technologies and recognising potential benefits for the sector<br>» Awareness that resources and support are provided across the organisation to enable colleagues to make the best use of available technology

	» Awareness of the fact that the organisation has a strategy for technology use<br>» Awareness of how to minimise environmental damage through technology use<br>» Basic operational knowledge of technology to maximise effectiveness and efficiency<br>» Basic operational knowledge of field-based technology (e.g., radio, general packet radio service (GPRS), satellite phone)
Advanced Technology Competency(ies):	7.2 Blockchain<br>7.11 Cloud Computing
7.2 Blockchain	» Awareness of blockchain as a value-add capability for relevant global health use cases
7.11 Cloud Computing	» Awareness of cloud computing as a value-add capability for relevant global health use cases

Key Performance Indicators:

- % of Order lines received accurately
- % Orders/ Lines Processed Complete
- % Orders/ Lines Received On-Time to Demand Requirement
- % Orders/ lines received with correct packaging
- % Orders/ Lines Received with Correct Shipping Documents
- % of Order lines received accurately
- % Orders/ Lines Processed Complete
- % Orders/ Lines Received On-Time to Demand Requirement
- % Orders/ lines received with correct packaging
- % Orders/ Lines Received with Correct Shipping Documents
- % of Order put-away within Put-Away cycle Time

Training:	Qualifications Available:	Certifications Available:
» Data management<br>» ERP Systems<br>» Legislation and Standards<br>» Discrepancy Reporting and Resolution		» Occupational Certificate: Store Person<br>» Occupational Certificate: Dispatching and Receiving Clerk

» ID & Damage Inspection<br>» Inventory Management<br>» Property Control and Disposition<br>» Return Management<br>» MSDS/CoC/BoL/Environmental Interpretation<br>» Logistics Management<br>» Bar Code Handling/RFID (if available)		» Receive stock in a DC warehouse skills programme

Assistant - Human Resources Assistant

**Organisation**: SC-focused Organisation				
Competency Level:	Serial Number:	Primary Process:	Job Role:	Supervises:
Tactical	5-571-572-1	Enable – Human Resources	Assistant - Human Resources Assistant	-

Contributions	Assists Human resources managerial staff with day to day activities in recruitment and training of staff
Personal/Management Competency(ies):	5.7 Oversee/support human resources (e.g., recruitment, training, team management/ supervision)<br>5.7.1 Manage staff recruitment process<br>5.7.2 Train staff<br>5.7.3 Supervise staff<br>5.7.4 Assessing HR systems<br>6.1 Demonstrate generic skills (e.g., literacy, numeracy, technology)<br>6.1.1 Exhibit high understanding of literacy and numeracy<br>6.2 Demonstrate strong communication skills<br>6.2.1 Practice cultural awareness

5.7 Oversee/support human resources (e.g., recruitment, training, team management/ supervision)

5.7.1 Manage staff recruitment process

- Awareness of a system for staff recruitment, appraisals and monitoring
- Awareness of training and recruitment plans to fill any identified gaps and shortages
- Awareness of relevant and appropriate selection criteria for a defined role
- Awareness of the standard selection documentation for recruitment
- Awareness of key issues impacting the size and membership of an interview panel (e.g., logistics, gender balance, absence of conflict of interest)
- Awareness of requirements for a fair, defensible and balanced interview process
- Awareness of the documentation maintained for each interviewed position
- Awareness of due process for formalising an appointment that minimises the opportunity for untoward effects (e.g., appeal, conflict, resignation)

- Awareness of job descriptions to obtain and retain skilled staff
- Awareness of the need to consult with and involve relevant personnel in development and updating of role descriptions/duty statements that clarify the duties and responsibilities of positions
- Awareness of the need to orient new staff to the workplace, explaining standard operating systems and procedures
- Awareness of human resource plans to meet the future staffing needs of the organisation
- Awareness of key features and measures used for monitoring a personnel retention strategy (e.g., recruitment costs, staff turnover, staff surveys)
- Awareness of the legislative issues impacting human resource policies and procedures
- Awareness the process of maintaining employee master data including medicals, allergies to ensure safe working environment
- Execute an employee opinion survey

5.7.2 Train staff

- Awareness of the need to maintain a list of staff that require logistics system training (based on supportive supervision reports)
- Awareness of the need to coordinate all training in managing the logistics system for all health facility staff
- Awareness of the need for participation in relevant learning and development opportunities
- Awareness of the need to assist in training nurses and other health professionals in medicines ordering and storage procedures
- Awareness of the need to orient new doctors to the formulary and the systems and procedures of the medicines supply system
- Awareness of the need to provide on-the-job training to district health officials and hospital pharmacists as needed
- Awareness of the need to ensure staff have the necessary skills and understanding for safe practice in the event they need to fill a management role due to absence or illness
- Awareness of the need to ensure all staff handling hazardous materials receive training on safety & compliance regulations
- Awareness of any staff needs at all levels of the supply chain
- Awareness of key factors important for initiating and/or sustaining the motivation of adult learners
- Awareness of the variable learning needs and styles or modalities in adult learners (e.g., visual versus auditory)
- Observe a range of strategies that could be used to reinforce and clarify educational content (e.g., tutorials or workshops, written materials, intranet- or internet-based resources)
- Basic operational knowledge of the use train-the-trainer to equipment trainers
- Execute training effectiveness monitoring and training feedback systems

5.7.3 Supervise staff	» Awareness of a performance management process with all personnel to ensure continuous improvement<br>» Awareness of the need for an environment that is supportive of learning and professional development<br>» Awareness of the nature of the supervisory role, what is meant by direct supervision, and where responsibility for outputs and outcomes rests<br>» Awareness of the need to plan and conduct supervisory tours of dependent facilities<br>» Awareness of the need to identify situations where supervised personnel are experiencing difficulties in completing work activities and/or where a mandatory notification obligation exists<br>» Awareness of signs/cues from supervised personnel that indicate additional guidance or support is needed (e.g., hesitancy, distress, seeks clarification from less authoritative sources)<br>» Awareness of the need to provide feedback, encouragement and support to team members<br>» Awareness of the need to discuss, in a constructive manner, areas where performance should/could be improved and provide resources to support this<br>» Awareness of the need to discuss expectations, achievements and contributions with personnel in a fair and equitable manner<br>» Awareness of the need to support colleagues in creating a professional development plan and suggesting ways the plan may be progressed through relevant training and/or experiential learning opportunities<br>» Awareness of the need to encourage and support high-potential employees to accept growth challenges outside their own areas<br>» Awareness of the progression opportunities for employees who have prepared themselves through development<br>» Awareness of the need to identify and deal with unproductive staff using an approved process in an ethical manner<br>» Awareness of the need to identify and address work issues contributing to impairment of personnel (e.g., excessive workload, conflict)<br>» Basic operational knowledge of the complaints management process<br>» Awareness of the fact that there may be situations where additional expertise should be sought (e.g., counselling in the event of workplace violence or in situations of diminished performance due to drug or alcohol abuse)<br>» Awareness of different working styles
5.7.4 Assessing HR systems	» Awareness of the need to assess HR systems
6.1 Demonstrate generic skills (e.g., literacy, numeracy, technology)	
6.1.1 Exhibit high understanding of literacy and numeracy	» Speak clearly and confidently, organize information in a logical manner, and consider voice tone and pace<br>» Respond to verbal messages and other cues (active listening)

- Express ideas and opinions clearly in written and verbal form
- Communicate information accurately, concisely and confidently in writing and verbally
- Practice a level of mathematics suitable to the job held
- Awareness of visual presentation techniques, including charting, histograms and flow sheets
- Read and comprehend at a level necessary to properly complete duties of the position
- Awareness of the need to interpret written information in documents, such as reports, SOPs, LMIS forms, graphs, calendars, schedules, notices and directions
- Awareness of the need to paying attention to detail and identifying the main ideas, detecting inconsistencies and identifying missing information in documents
- Execute computer skills — such as using Word, Excel, PowerPoint and the internet — suitable to the job level
- Operational knowledge of analysing and validating KPIs
- Awareness of how the evaluation process of activities that act as standards against which the KPIs are measured is carried out
- Execute the process of performing critical self-review on work before submission

6.2 Demonstrate strong communication skills

- Be truthful and trustworthy, and supply accurate information at all times
- Realize the need to work as part of a workplace team
- Awareness of vocabulary and communication style and form for both written and verbal communication that is appropriate for the situation, audience and material being communicated (e.g., avoid unnecessary jargon, clearly explain medical and SCM terminology)
- Awareness of open communication within constraints of confidentiality
- Awareness of communications about logistics
- Participate in meetings, expressing one’s opinions, being aware of others’ needs, and being appropriately assertive when required
- Awareness of perspective into the point of view of others, understanding their needs and goals
- Build trust, rapport and credibility with others
- Awareness of the communication needs and concerns of others and respond to them
- Awareness of the need to avoid conflict between work and personal interests
- Practice meaningful two-way communication
- Realize that others are influenced by persuasively presenting thoughts and ideas
- Awareness of the need for assertiveness skills to deal with unreasonable requests and/or refusals that would compromise practice or consumer care
- Maintain a positive, supportive and appreciative attitude

	» Awareness of the need to balance the organisation's needs and the customer's needs<br>» Execute self-control by maintaining composure and keeping emotions in check, even in difficult situations; deal calmly and effectively with stressful situations<br>» Express opinions and provide information in written and/or verbal form in a manner that does not elicit concern, anger or other adverse response<br>» Basic operational knowledge of a systematic process for following up that demonstrates written reports have been received and understood<br>» Basic operational knowledge of the means by which responses to input to the work environment are monitored<br>» Seek practical ways to overcome barriers to communication<br>» Awareness of the need for language representation (e.g., interpreters) at meetings when appropriate<br>» Realize that difficult situations and disputes between staff need to be tackled and resolved<br>» Realize the need to give both positive and negative feedback sensitively<br>» Awareness of the need to maintain and improve communication<br>» Execute the process of giving and requesting feedback frequently<br>» Execute effective communication with shop stewards and union representatives
6.2.1 Practice cultural awareness	» Awareness of the need for cultural awareness and sensitivity, treating all people with fairness, respect and dignity<br>» Awareness of the anti-discriminatory practices in the organisation, including HR and disciplinary procedures<br>» Awareness of the need for cultural sensitivity, equality and fairness at all levels of the organisation<br>» Challenge discriminatory behaviour directly and sensitively<br>» Act in a non-discriminatory way toward individuals and groups<br>» Awareness of the need for integrating cultural awareness in learning and development approaches<br>» Avoid stereotypical responses by examining one's own behaviour and bias<br>» Awareness of the need to manage cultural diversity in teams and make the most of differences<br>» Awareness of strategies and/or resources for communicating effectively with people from different cultural backgrounds<br>» Awareness of the need to elicit information relating to values, beliefs and cultural backgrounds of consumers that may influence the way professional services are provided
Basal Technology Competency(ies):	7.12 Basic Office Skills<br>7.13 Have a command of technology
7.12 Basic Office Skills	» Have a good understanding of common presentation authoring packages

	» Have a good understanding of common spreadsheet authoring packages<br>» Have a good understanding of common document authoring packages<br>» Have a good understanding of common email authoring packages<br>» Have a good understanding of common instant messaging packages<br>» Have a good understanding of common video conferencing authoring packages
7.13 Have a command of technology	» Execute the use of technology suitable to the job held<br>» Awareness of the need to monitor new developments and technologies in the sector<br>» Awareness of new technologies and recognising potential benefits for the sector<br>» Awareness that resources and support are provided across the organisation to enable colleagues to make the best use of available technology<br>» Awareness of the fact that the organisation has a strategy for technology use<br>» Awareness of how to minimise environmental damage through technology use<br>» Basic operational knowledge of technology to maximise effectiveness and efficiency<br>» Basic operational knowledge of field-based technology (e.g., radio, general packet radio service (GPRS), satellite phone)
Advanced Technology Competency(ies):	7.11 Cloud Computing
7.11 Cloud Computing	» Awareness of cloud computing as a value-add capability for relevant global health use cases

Key Performance Indicators:

- Competency compliance. No of personnel evaluated and found to competent versus plan.
- Creation of the HR4SCM Competency Requirements & Plan based on the Supply Chain Requirements
- Number of personnel with the required Skill, Education, Training and Aptitude as per Supply Chain Strategy versus Total number of personnel.
- Number of personnel progressed professionally versus Number of personnel that were targeted for progress.
- Planned skills upliftment plan - Number of personnel per competency versus actual skills upliftment Number of personnel per competency.
- HR4SCM Competency Requirements & Plan
- Direct Labour Cost
- Human Resource Contract Management
- EHS regulations
- Legislation and Standards

» Performance Management		
**Training:**	**Qualifications Available:**	**Certifications Available:**
» Legislation and Standards<br>» Human resources for supply chain		» National Certificate: Business Studies: FET Phase

Public Health Organisation – Intermediate Level

Strategic

Lead – SC Governance

Organisation: **Public Health Organisation – Intermediate Level**				
Competency Level:	Serial Number:	Primary Process:	Job Role:	Supervises:
Strategic	5-532-64-4	Plan – Supply Chain	Lead – SC Governance	Lead – Compliance / Internal Audit, Lead – Compliance and Risk Management
Contributions	Ensures that the Governance Enabling process is current and appropriate to the execution of the Public Health System Organisation. Supports the Public Health Care Supply Chain Strategy by participating in the Strengthening of the Governance process			
Personal/Management Competency(ies):	5.3 Implement risk management and monitoring and evaluation activities for the supply chain<br>5.3.2 Implement risk management activities<br>6.1 Demonstrate generic skills (e.g., literacy, numeracy, technology)<br>6.1.1 Exhibit high understanding of literacy and numeracy<br>6.2 Demonstrate strong communication skills<br>6.2.1 Practice cultural awareness<br>6.4 Exhibit professional and ethical values<br>6.4.1 Demonstrate integrity<br>6.4.2 Engage in continuous professional development			

5.3 Implement risk management and monitoring and evaluation activities for the supply chain	
5.3.2 Implement risk management activities	» Determine risks affecting supply, transformation, delivery and customer demand<br>» Determine what constitutes an unsafe working conditions and how to take prompt, corrective action; stay alert to and take preventive action against hazards and threats; recommend measures to protect employees from hazardous working conditions; follow protocol for reporting safety violations; and investigate accidents to determine how to prevent them in the future

	» Demonstrate training programmes/drills in which personnel participate (e.g., fire and armed hold-up drills, safe manual handling practices, use of mandatory safety clothing)<br>» Analyse systems of review (e.g., audit) to check compliance with policies and procedures intended to maintain workplace safety (e.g., manual handling techniques, correct use of protective clothing)<br>» Determine the process of ensuring equipment and tools are operating to prescribed standards<br>» Determine key security systems for the workplace (e.g., for cash, narcotics and other controlled substances, investigational drugs, consumer records, entry and exit points) and levels of access and/or authority applicable to each<br>» Demonstrate the prevention strategies adopted (e.g., protocols, security barriers, fixed or personal duress alarms)<br>» Evaluate documented critical incidents<br>» Engage in contingency planning and managing operational continuity<br>» Ensure that there is an understanding of what a safe working environment is and engender a shared commitment from all personnel to creating one<br>» Demonstrate knowledge of relevant safety and health laws and regulations; comply with safety codes, standards and guidelines; adhere to worksite safety programmes; understand emergency response plans when they exist<br>» Demonstrate knowledge of how to handle hazardous commodities and first aid procedures
6.1 Demonstrate generic skills (e.g., literacy, numeracy, technology)	
6.1.1 Exhibit high understanding of literacy and numeracy	» Encourage speaking clearly and confidently, organizing information in a logical manner, and considering voice tone and pace<br>» Demonstrate responding to verbal messages and other cues (active listening)<br>» Demonstrate expressing ideas and opinions clearly in written and verbal form<br>» Demonstrate communicating information accurately, concisely and confidently in writing and verbally<br>» Demonstrate a level of mathematics suitable to the job held<br>» Demonstrate knowledge of visual presentation techniques, including charting, histograms and flow sheets<br>» Demonstrate reading and comprehending at a level necessary to properly complete duties of the position<br>» Demonstrate the process of interpreting written information in documents, such as reports, SOPs, LMIS forms, graphs, calendars, schedules, notices and directions<br>» Demonstrate paying attention to detail and identifying the main ideas, detecting inconsistencies and identifying missing information in documents

	» Demonstrate computer skills — such as using Word, Excel, PowerPoint and the internet — suitable to the job level<br>» Demonstrate the process of analysing and validating KPIs<br>» Participate in the evaluation of activities that act as standards against which the KPIs are measured<br>» Demonstrate the process of performing critical self-review on work before submission
6.2 Demonstrate strong communication skills	» Demonstrate being truthful and trustworthy, and supplying accurate information at all times<br>» Demonstrate working as part of a workplace team<br>» Determine vocabulary and communication style and form for both written and verbal communication that is appropriate for the situation, audience and material being communicated (e.g., avoid unnecessary jargon, clearly explain medical and SCM terminology)<br>» Demonstrate open communication within constraints of confidentiality<br>» Analyse communications about logistics<br>» Demonstrate participation in meetings, expressing one's opinions, being aware of others' needs, and being appropriately assertive when required<br>» Demonstrate perspective into the point of view of others, understanding their needs and goals<br>» Demonstrate the practise of building trust, rapport and credibility with others<br>» Determine the communication needs and concerns of others and respond to them<br>» Demonstrate avoiding conflict between work and personal interests<br>» Demonstrate the practice of meaningful two-way communication<br>» Demonstrate influence on others by persuasively presenting thoughts and ideas<br>» Demonstrate assertiveness skills to deal with unreasonable requests and/or refusals that would compromise practice or consumer care<br>» Demonstrate a positive, supportive and appreciative attitude<br>» Demonstrate the ability to balance the organisation's needs and the customer's needs<br>» Demonstrate self-control by maintaining composure and keeping emotions in check, even in difficult situations; deal calmly and effectively with stressful situations<br>» Demonstrate the expressing of opinions and providing information in written and/or verbal form in a manner that does not elicit concern, anger or other adverse response<br>» Demonstrate the use of a systematic process for following up that demonstrates written reports have been received and understood<br>» Evaluate the means by which responses to input to the work environment are monitored<br>» Seek practical ways to overcome barriers to communication

	» Ensure language representation (e.g., interpreters) at meetings when appropriate<br>» Demonstrate tackling difficult situations and resolving disputes between staff<br>» Demonstrate giving both positive and negative feedback sensitively<br>» Demonstrate maintaining and improving communication<br>» Demonstrate the process of giving and requesting feedback frequently<br>» Demonstrate the ability to communicate effectively with shop stewards and union representatives
6.2.1 Practice cultural awareness	» Demonstrate cultural awareness and sensitivity, treating all people with fairness, respect and dignity<br>» Demonstrate anti-discriminatory practices in the organisation, including HR and disciplinary procedures<br>» Demonstrate cultural sensitivity, equality and fairness at all levels of the organisation<br>» Demonstrate challenging discriminatory behaviour directly and sensitively<br>» Demonstrate acting in a non-discriminatory way toward individuals and groups<br>» Demonstrate the process of integrating cultural awareness in learning and development approaches<br>» Demonstrate avoiding stereotypical responses by examining one's own behaviour and bias<br>» Demonstrate managing cultural diversity in teams and make the most of differences<br>» Demonstrate the use of strategies and/or resources for communicating effectively with people from different cultural backgrounds<br>» Supply information relating to values, beliefs and cultural backgrounds of consumers that may influence the way professional services are provided
6.4 Exhibit professional and ethical values	
6.4.1 Demonstrate integrity	» Demonstrate accepting responsibility for work tasks and performance<br>» Demonstrate working within a framework of clearly understood humanitarian values and ethics<br>» Demonstrate standing by decisions and holding others accountable when necessary<br>» Demonstrate not abusing one's own power or position<br>» Demonstrate the process of managing team members to ensure they do not abuse their power or position<br>» Determine when individuals or the organisation is straying from organisation goals, and challenge them to uphold ethics<br>» Demonstrate resisting undue political pressure in decision making

	» Demonstrate supporting staff in maintaining ethical stances<br>» Demonstrate making time in team for ethical inquiry and reflection<br>» Demonstrate showing consistency between expressed principles and behaviour<br>» Ensure that principles, values and ethics are embedded in policy<br>» Demonstrate acting without consideration of personal gain<br>» Demonstrate promoting transparency in decision making structures and processes<br>» Ensure programmes are acting with integrity, and recognise the impact of not doing so<br>» Demonstrate recognising one's own limitations and act upon them<br>» Demonstrate all standard operating procedures<br>» Demonstrate working in a safe and legal way<br>» Demonstrate respect, dignity and consideration for consumers<br>» Determine the impact of a no-blame culture on reporting and preventing recurrence of incidents<br>» Evaluate requests of colleagues that might be regarded as unreasonable<br>» Demonstrate communicating (verbally and by example) expectations of the desired standards and approaches to be adopted<br>» Determine roles and responsibilities in terms of the position statement/duty statement of the position held<br>» Demonstrate punctuality<br>» Demonstrate applying professional care and expertise to deliver high quality of services<br>» Demonstrate care and attention to detail in undertaking work activities<br>» Determine appropriate attire and presentation for the role and situation<br>» Demonstrate recognising and taking responsibility for emotions<br>» Demonstrate an environment in which others can talk and act without fear of repercussion<br>» Demonstrate keeping commitments and not letting people down<br>» Demonstrate being trusting and cooperative when working alongside others<br>» Demonstrate taking on various responsibilities within the department as the need arises
6.4.2 Engage in continuous professional development	» Demonstrate seeking out opportunities to learn, and integrating new knowledge and skills into work<br>» Determine gaps in knowledge and skills for completing specific tasks, and develop a plan to acquire them<br>» Demonstrate listening to and inviting feedback from others on one's own performance<br>» Demonstrate using lifelong learning (continuous striving to gain knowledge and maintain competence) in the context of career development and the professional's role in delivering healthcare services

- Analyse organisational systems for capturing learning and ensuring lessons learned
- Employ reflective learning within the team, in which lessons are captured and integrated into future projects
- Promote continuous learning as an integral part of organisational performance
- Demonstrate being open to new ideas and different perspectives
- Demonstrate keeping up to date in the place of work with input from supervisors
- Demonstrate documenting continual professional development activities
- Demonstrate the use of multiple techniques to acquire new knowledge and skills; process and retain information; and identify when it is necessary to acquire new knowledge and skills.
- Convene professional organisations and/or committees
- Determine and/or demonstrate quality improvement and/or quality assurance activities participated in
- Demonstrate reflecting on performance and seeking what needs improvement
- Demonstrate the process of engaging in personal career development, identifying occupational interests, strengths, options and opportunities
- Demonstrate contributing to others' professional development
- Demonstrate sharing experiences and learning internally and externally
- Demonstrate seeking out challenging projects outside of core experience and achieve solid results

Basal Technology Competency(ies):	7.5 Planning systems<br>7.6 ERP includes function of LMIS<br>7.12 Basic Office Skills<br>7.13 Have a command of technology

7.5 Planning systems

- Analyse blockchain and understand the building blocks for planning systems
- Evaluate planning systems as a value-add capability for relevant global health use cases

7.6 ERP includes function of LMIS

- Evaluate a Master Data Management System
- Demonstrate an understanding of the critical components of an ERP system
- Demonstrate an understanding of the meaning of common document types found within an ERP/LMIS
- Demonstrate an understanding of the logic of data visibility within an ERP
- Demonstrate the importance of data integrity, record management and information security

7.12 Basic Office Skills	» Demonstrate a good understanding of common presentation authoring packages<br>» Demonstrate a good understanding of common spreadsheet authoring packages<br>» Demonstrate a good understanding of common document authoring packages<br>» Demonstrate a good understanding of common email authoring packages<br>» Demonstrate a good understanding of common instant messaging packages<br>» Demonstrate a good understanding of common video conferencing authoring packages
7.13 Have a command of technology	» Demonstrate the use of technology suitable to the job held<br>» Monitor new developments and technologies in the sector<br>» Experiment with new technologies and recognising potential benefits for the sector<br>» Ensure that resources and support are provided across the organisation to enable colleagues to make the best use of available technology<br>» Determine what the organisations' strategy is for technology use<br>» Determine how to minimise environmental damage through technology use<br>» Analyse the use of technology to maximise effectiveness and efficiency<br>» Analyse the use of field-based technology (e.g., radio, general packet radio service (GPRS), satellite phone)
Advanced Technology Competency(ies):	7.2 Blockchain<br>7.7 Automation<br>7.11 Cloud Computing
7.2 Blockchain	» Evaluate blockchain as a value-add capability for relevant global health use cases
7.7 Automation	» Evaluate automation as a value-add capability for relevant global health use cases
7.11 Cloud Computing	» Evaluate cloud computing as a value-add capability for relevant global health use cases

Key Performance Indicators:

- Assess Delivery Performance Cycle Time - Actual Time from assessed requirement to time delivered to end-user compared to committed Delivery Cycle Time as per Supply Chain strategy
- Assess Supplier Performance Cycle Time - Actual Supplier performance Cycle time versus Supplier commitment cycle time
- Inventory accuracy - Cycle count results
- Transportation lead time accuracy - Actual Transportation lead time versus Transportation promised lead time.
- Transport efficiency - Total cost of transport versus planned cost of transport by mode
- In-bound cost of acquisition - Actual total cost of acquisition versus Total Planned cost of acquisition including landed costs
- Manage performance cycle time
- Performance Report - Actual versus Plan reports for all metrics
- Order fill rate (quantity and item lines fulfilled according to the DP requisitions)
- Stock according to plan/ to show overstock or understock
- Availability of health commodities/Stock out
- Expiration of health products (total value of expired products/Value of inventory + value of expiries)
- Inventory accuracy for warehouse management system vs physical count

Training:	Qualifications Available:	Certifications Available:
» Risk and exception management<br>» Process Auditing	» Bachelor of Business Administration<br>» Bachelor of Supply Chain Management	» SPSM3 TM (Enterprise-Wide Procurement Influence)<br>» Occupational Certificate: Health Information Manager<br>» APICS Certified supply chain professional certificate

Managerial
Head – Demand and Supply Planning

Organisation:

Public Health Organisation – Intermediate Level

Competency Level:	Serial Number:	Primary Process:	Job Role:	Supervises:
Managerial	1-14-15-13-3	Plan – Procurement	Head – Demand and Supply Planning	Officer – Demand and Supply Planning

Contributions	Articulates the Supply Chain Strategy to the Procurement functionality within the context. Ensures that the Sourcing/Procurement department is enabled to supply the public health system in line with the Supply Chain Materials and Resource Plan., Facilitates the execution of the Supply Strategy in the context, Implements best practices to extract the most efficiency from each process and guarantee supply accuracy, lead time and cost diminution as well as data accuracy.
Technical Competency(ies):	1.1 Select the appropriate product<br>1.2 Define the specifications of the product, including product quality<br>1.3 List any special considerations for the product (e.g., temperature requirements, size, implications for infrastructure)<br>1.4 Forecast and quantify product needs<br>1.5 Supply planning
1.1 Select the appropriate product	» Describe the broad concepts of the national medicines policy, essential medicines lists, essential equipment lists, standard treatment guidelines and “dangerous drug” (DDA) policy<br>» Use the government system required to add and subtract items from the essential medicines list and the essential equipment list<br>» Follow the government system required to alter standard treatment guidelines, dangerous drug policy and national medicines policy<br>» Confirm the type of supplies and services that are required<br>» Participate in regularly scheduled coordination meetings with stakeholders involved in financing, procuring or distributing commodities<br>» Describe the steps needed to bring a medicinal product to the market, including the safety, quality, efficacy and pharmacoeconomic assessments of the product
1.2 Define the specifications of the product, including product quality	» Define specifications for procurement<br>» Describe the characteristics of a good specification<br>» Discuss the types of specifications<br>» Identify the advantages of functional and performance specifications<br>» List the contents of a specification<br>» Identify the procurement staff responsibilities in the specification process<br>» Describe the quality standard of product<br>» Ensure that solution meets specification requirements
1.3 List any special considerations for the product (e.g., temperature requirements, size, implications for infrastructure)	» Describe the principles and processes of category management, including market segmentation principles<br>» List any specific considerations in the quantification of programme-specific products (e.g., ARVs, family planning commodities, vaccines)<br>» Describe current international trends in commodity availability<br>» Define regulatory requirements for the product
1.4 Forecast and quantify product needs	» Identify the factors that affect usage patterns of medications and equipment and how this affects ordering (e.g., disease outbreaks), using national policies as a guide and to ensure consistent application<br>» Apply principles and applications of demand forecasting

	» Apply the critical requirements for effective forecasting: establishing time horizons, level of detail and use of data<br>» Describe policies and procedures for forecasting<br>» Quantify product requirements using a variety of methods<br>» Organize and summarize consumption data at the national level for various country programmes<br>» Describe the use different types of forecasts<br>» Apply VEN or ABC analysis to programme requirements for national level procurement<br>» Calculate average monthly dispensed-to-user quantities for all service delivery points nationally<br>» Calculate storage space requirements for all levels in the supply chain<br>» Calculate the months of supply on hand for each commodity at the national level<br>» Describe the maximum and minimum stock levels for each level in a programme<br>» Use various tools used in quantification (e.g., quantification software)<br>» Describe the use of various data sources for commodity forecasting (e.g., consumption data, services data, demographic data)<br>» Establish key performance indicators of forecast accuracy<br>» Establish the degree of error when using forecasting methods
1.5 Supply planning	» Describe a purchasing and supply plan in line with national priorities<br>» Balance supply chain resources and trigger activities to correct any imbalances<br>» Explain the formalisation of sales and operations plans through sales and operations meeting.<br>» Translate sales and operations plan into executable plans to ensure dispatch of goods in line with overall priorities.<br>» Describe the national commodity pipeline
Personal/Management Competency(ies):	6.1 Demonstrate generic skills (e.g., literacy, numeracy, technology)<br>6.1.1 Exhibit high understanding of literacy and numeracy<br>6.2 Demonstrate strong communication skills<br>6.2.1 Practice cultural awareness
6.1 Demonstrate generic skills (e.g., literacy, numeracy, technology)	»
6.1.1 Exhibit high understanding of literacy and numeracy	» Speak clearly and confidently, organize information in a logical manner, and consider voice tone and pace<br>» Respond to verbal messages and other cues (active listening)<br>» Express ideas and opinions clearly in written and verbal form<br>» Communicate information accurately, concisely and confidently in writing and verbally<br>» Practice a level of mathematics suitable to the job held

- Describe visual presentation techniques, including charting, histograms and flow sheets
- Read and comprehend at a level necessary to properly complete duties of the position
- Interpret written information in documents, such as reports, SOPs, LMIS forms, graphs, calendars, schedules, notices and directions
- Pay attention to detail and identify the main ideas, detect inconsistencies and identify missing information in documents
- Apply computer skills — such as using Word, Excel, PowerPoint and the internet — suitable to the job level
- Describe the process of analysing and validating KPIs
- Participate in the evaluation of activities that act as standards against which the KPIs are measured
- Apply the process of performing critical self-review on work before submission

6.2 Demonstrate strong communication skills

- Be truthful and trustworthy, and supply accurate information at all times
- Work as part of a workplace team
- Describe vocabulary and communication style and form for both written and verbal communication that is appropriate for the situation, audience and material being communicated (e.g., avoid unnecessary jargon, clearly explain medical and SCM terminology)
- Encourage open communication within constraints of confidentiality
- Organize communications about logistics
- Encourage participation in meetings, expressing one's opinions, being aware of others' needs, and being appropriately assertive when required
- Apply perspective into the point of view of others, understanding their needs and goals
- Encourage building trust, rapport and credibility with others
- Anticipate the communication needs and concerns of others and respond to them
- Avoid conflict between work and personal interests
- Encourage meaningful two-way communication
- Influence others by persuasively presenting thoughts and ideas
- Apply assertiveness skills to deal with unreasonable requests and/or refusals that would compromise practice or consumer care
- Maintain a positive, supportive and appreciative attitude
- Apply the ability to balance the organisation's needs and the customer's needs
- Use self-control by maintaining composure and keeping emotions in check, even in difficult situations; deal calmly and effectively with stressful situations
- Encourage the expressing of opinions and providing information in written and/or verbal form in a manner that does not elicit concern, anger or other adverse response
- Apply the use of a systematic process for following up that demonstrates written reports have been received and understood

	» Describe the means by which responses to input to the work environment are monitored<br>» Seek practical ways to overcome barriers to communication<br>» Identify language representation (e.g., interpreters) at meetings when appropriate<br>» Tackle difficult situations and resolve disputes between staff<br>» Give both positive and negative feedback sensitively<br>» Maintain and improve communication<br>» Apply the process of giving and requesting feedback frequently<br>» Apply the ability to communicate effectively with shop stewards and union representatives
6.2.1 Practice cultural awareness	» Describe cultural awareness and sensitivity, treating all people with fairness, respect and dignity<br>» Describe anti-discriminatory practices in the organisation, including HR and disciplinary procedures<br>» Establish cultural sensitivity, equality and fairness at all levels of the organisation<br>» Challenge discriminatory behaviour directly and sensitively<br>» Act in a non-discriminatory way toward individuals and groups<br>» Integrate cultural awareness in learning and development approaches<br>» Avoid stereotypical responses by examining one's own behaviour and bias<br>» Manage cultural diversity in teams and make the most of differences<br>» Describe strategies and/or resources for communicating effectively with people from different cultural backgrounds<br>» Elicit information relating to values, beliefs and cultural backgrounds of consumers that may influence the way professional services are provided
Basal Technology Competency(ies):	7.4 Temperature and monitoring<br>7.6 ERP includes function of LMIS<br>7.12 Basic Office Skills<br>7.13 Have a command of technology
7.4 Temperature and monitoring	» Describe the current technology approaches for Temperature monitoring and sensors<br>» Describe what is required to implement this technology in a country context<br>» Describe infrastructure requirement for heat-sensitive items at peripheral 'lower level' of SC depending on volume and nature of items to handle;<br>» Define regular preventive maintenance and calibration of cold-chain equipment with biomedical engineers or logisticians<br>» Describe the policy for temperature monitoring tools during transportation and storage<br>» Describe the steps involved in health logistic management cycle: selection-order-delivery-reception-storage-distribution-inventory

	» Describe hardware qualification and system validation of temperature monitoring<br>» Consider mass data management of temperature monitoring
7.6 ERP includes function of LMIS	» Support a Master Data Management System<br>» Describe the critical components of an ERP system<br>» Describe the meaning of common document types found within an ERP/LMIS<br>» Describe the logic of data visibility within an ERP<br>» Describe the importance of data integrity, record management and information security
7.12 Basic Office Skills	» Have a good understanding of common presentation authoring packages<br>» Have a good understanding of common spreadsheet authoring packages<br>» Have a good understanding of common document authoring packages<br>» Have a good understanding of common email authoring packages<br>» Have a good understanding of common instant messaging packages<br>» Have a good understanding of common video conferencing authoring packages
7.13 Have a command of technology	» Apply the use of technology suitable to the job held<br>» Provide input on monitoring new developments and technologies in the sector<br>» Identify new technologies and recognising potential benefits for the sector<br>» Identify resources and support that are provided across the organisation to enable colleagues to make the best use of available technology<br>» Ensure that the organisation has a strategy for technology use<br>» Describe how to minimise environmental damage through technology use<br>» Use technology to maximise effectiveness and efficiency<br>» Use field-based technology (e.g., radio, general packet radio service (GPRS), satellite phone)
Advanced Technology Competency(ies):	7.2 Blockchain<br>7.3 Unmanned Aerial Vehicles (UAVs)<br>7.7 Automation<br>7.9 Additive Manufacturing<br>7.11 Cloud Computing<br>7.14 eProcurement
7.2 Blockchain	» Describe blockchain as a value-add capability for relevant global health use cases

7.3 Unmanned Aerial Vehicles (UAVs)	» Describe the current UAV landscape, their application and the enabling environment required to support country implementation<br>» Describe UAVs as a value-add capability for relevant global health use cases<br>» Describe the laws/ regulations around the use of UAV's
7.7 Automation	» Describe automation as a value-add capability for relevant global health use cases
7.9 Additive Manufacturing	» Describe additive manufacturing as a value-add capability for relevant global health use cases
7.11 Cloud Computing	» Describe cloud computing as a value-add capability for relevant global health use cases
7.14 eProcurement	» Describe how to transact with an eProcurement system where necessary<br>» Describe eProcurement as a value-add capability for relevant global health use cases<br>» Apply the benefits and limitations of eProcurement to best utilise the system

Key Performance Indicators:

- Vendor on time delivery (VOTD)
- % of emergency orders issued in the last 12 months
- % of product selection based on National Essential Medicines List
- % of procured products that meet Stringent Regulatory Authority (SRA) or WHO standards
- Supply adequacy - Net requirements are reflected in the Procurement pipeline to secure sufficient future supply.
- Supply Plan report; supplier performance report
- Data accuracy - Vendor Master data accuracy, item / SKU Master Data accuracy
- Fill Rate vs Planned fill rate
- Forecast accuracy, measured and reported on.
- Identify, Prioritize, and Aggregate Supply Chain Requirements Cycle Time - Actual Time taken to determine net supply chain requirements by product and SKU vs Planned Time
- Inventory Days of Supply - Actual inventory - Planned Demand over the planning horizon divided by the average daily usage
- Order accuracy - Net requirement versus order quantities in the planning horizon
- Ratio between median price of pro- ducts procured and the international median reference value

- Supply Chain Planning Cost recorded and measured against required Supply China Planning Cycle times to accomplish Supply Chain Strategic objectives.
- Availability - Stock out rate - Number of missed line item quantity deliveries due to stock out versus total line item quantity deliveries
- Availability - Stock levels - Current on hand stock versus Planned on hand stock
- Forecasting and Supply Planning done within the specified cycle time to support the Supply Chain performance requirements.
- Establish Product Demand and Resources Cycle Time and recorded.in the "ERP" system against the products and resources.
- Establish Supply Plan Cycle Times and recorded in the "ERP" system.
- Material and Resource Plan valid. Number of valid actions performed versus number of actions performed. (Exception messages trigger due to invalid schedules)
- Procurement planning Cycle Time, by product and resource. Actual Overall planning cycle versus required Overall planning cycle time to achieve Supply Chain performance requirements.
- Reports submitted on time (for all and separate reports) in accordance with business rules (reporting calendar)

Training:	Qualifications Available:	Certifications Available:
» Risk and exception management<br>» Import & export regulations for products<br>» Inventory management, Min-Max Replenishment<br>» ABC/VEN Inventory Classification	» Diploma in International Trade Management in Exports/Imports	» Occupational Certificate: Procurement Officer<br>» CIPS Level 4 Diploma in Procurement and Supply<br>» SPSM2® (Global Procurement Management)

Lead - Procurement

Organisation: **Public Health Organisation – Intermediate Level**				
Competency Level:	Serial Number:	Primary Process:	Job Role:	Supervises:
Managerial	2-21-22-4	Enable – Procurement	Lead - Procurement	Officer– Procurement, Lead – Returns and Disposal, Officer – Procurement and Supply
Contributions	Leads the procurement department within context to enable the procurement processes as defined in the Supply Chain design. Ensures that Procurement Master Data is adequate to eliminate bottlenecks in the procurement process.			
Technical Competency(ies):	2.1 Manage procurement costs and budget<br>2.2 Build and maintain supplier relationships			

2.1 Manage procurement costs and budget	» Ensure the organisation follows public procurement regulations<br>» Ensure adherence to the approved procurement budget<br>» Evaluate financial and management accounting practices within procurement<br>» Analyse commercial factors that contribute toward cost reduction, price savings and value improvement opportunities<br>» Analyse the principles of foreign exchange rates and how they impact prices
2.2 Build and maintain supplier relationships	» Analyse the market<br>» Analyse adherence to the processes for prequalification and tender contracting<br>» Determine the factors that define a good supplier<br>» Effectively locate and source key suppliers, while analysing the total cost associated with procuring an item or service<br>» Analyse supplier relationship management plans (e.g., sourcing, frequency of meetings, negotiate and monitor benchmarks for performance)<br>» Analyse of the process of educating suppliers to create value for customers by streamlining processes in the value chain<br>» Analyse the difference between strategic and non-strategic suppliers and the associated supplier management principles<br>» Analyse commercial factors that contribute toward cost reduction, price savings and value improvement opportunities<br>» Ensure the strategic sourcing practices drive down cost across consecutive tenders

	» Analyse the process of working with suppliers to agree cost saving opportunities in the whole value chain, with an appropriate cost sharing mechanism applied to ensure a “win-win” outcome<br>» Evaluate the process of managing supplier risk<br>» Demonstrate ethical sourcing
Personal/Management Competency(ies):	5.5 Manage and plan projects<br>5.5.2 Execute strategic decision making<br>5.5.3 Manage partnerships<br>6.1 Demonstrate generic skills (e.g., literacy, numeracy, technology)<br>6.1.1 Exhibit high understanding of literacy and numeracy<br>6.2 Demonstrate strong communication skills<br>6.2.1 Practice cultural awareness<br>6.3 Utilize problem-solving skills<br>6.3.1 Negotiate

5.5 Manage and plan projects

**5.5.2 Execute strategic decision making**	» Determine how one can make decisions regarding one’s own workload and area of responsibility<br>» Determine the process of prioritizing competing tasks and perform them quickly and efficiently according to their importance; finding new ways of organizing or planning work to accomplish it more efficiently<br>» Evaluate the proper time and space for consultation around decisions being made.<br>» Demonstrate the process of considering the input of resources including staff needed<br>» Analyse assumptions against facts<br>» Determine the key issues in a complex situation and come to the heart of the problem quickly<br>» Evaluate relevant information before making decisions<br>» Provide input to strategic decisions in the interest of the organisation’s goals<br>» Evaluate when to analyse, evaluate and execute tough decisions when necessary<br>» Analyse the impact of decisions on others’ work or team goals<br>» Communicate decisions and ensure they are incorporated into policies and processes<br>» Demonstrate inclusive and consultative strategic planning to establish strategic objectives<br>» Evaluate emerging trends and practices<br>» Analyse the macro and long-term consequences of decisions
**5.5.3 Manage partnerships**	» Demonstrate the concepts of partnership working<br>» Determine the process of supporting implementation of partnership programmes

- Analyse the process of experimenting with and trialling new ideas with partners
- Analyse partnerships to deliver programme and increase impact
- Analyse innovation and creativity in partnership work
- Communicate key information with partner members
- Involve and value partners in all aspects of programming
- Analyse problems with supply chain relationships
- Demonstrate the process of communicating effectively with nurses, doctors and other members of the healthcare team
- Analyse the process of responding to consumer complaints or comments about services and/or advice received
- Analyse collaboration and consultation across the sector and encourage the team to work with others to gain market insight
- Encourage input from other key actors in the humanitarian sector and develop relationships with a cross-section of actors
- Analyse the process of meeting the reporting requirements of vertical programmes
- Analyse the structure of the health system at a national level and explain this to others
- Analyse the roles of other members of the healthcare team (including with consumers) in a way that engenders understanding and confidence in the team and its members
- Analyse the structure of the organisation, environment and/or service in which they work
- Demonstrate how vertical programmes work within the health system
- Evaluate the communication network established to achieve work outcomes
- Demonstrate knowledge of the structure of the health system at the provincial/regional level and explain this to others
- Analyse the roles and functions of government agencies in regulating and supporting supply chain organisations
- Demonstrate knowledge of change management (e.g. PtD Theory of Change)

6.1 Demonstrate generic skills (e.g., literacy, numeracy, technology)

6.1.1 Exhibit high understanding of literacy and numeracy

- Encourage speaking clearly and confidently, organizing information in a logical manner, and considering voice tone and pace
- Demonstrate responding to verbal messages and other cues (active listening)
- Demonstrate expressing ideas and opinions clearly in written and verbal form
- Demonstrate communicating information accurately, concisely and confidently in writing and verbally
- Demonstrate a level of mathematics suitable to the job held
- Demonstrate knowledge of visual presentation techniques, including charting, histograms and flow sheets

- Demonstrate reading and comprehending at a level necessary to properly complete duties of the position
- Demonstrate the process of interpreting written information in documents, such as reports, SOPs, LMIS forms, graphs, calendars, schedules, notices and directions
- Demonstrate paying attention to detail and identifying the main ideas, detecting inconsistencies and identifying missing information in documents
- Demonstrate computer skills — such as using Word, Excel, PowerPoint and the internet — suitable to the job level
- Demonstrate the process of analysing and validating KPIs
- Participate in the evaluation of activities that act as standards against which the KPIs are measured
- Demonstrate the process of performing critical self-review on work before submission

6.2 Demonstrate strong communication skills

- Demonstrate being truthful and trustworthy, and supplying accurate information at all times
- Demonstrate working as part of a workplace team
- Determine vocabulary and communication style and form for both written and verbal communication that is appropriate for the situation, audience and material being communicated (e.g., avoid unnecessary jargon, clearly explain medical and SCM terminology)
- Demonstrate open communication within constraints of confidentiality
- Analyse communications about logistics
- Demonstrate participation in meetings, expressing one's opinions, being aware of others' needs, and being appropriately assertive when required
- Demonstrate perspective into the point of view of others, understanding their needs and goals
- Demonstrate the practise of building trust, rapport and credibility with others
- Determine the communication needs and concerns of others and respond to them
- Demonstrate avoiding conflict between work and personal interests
- Demonstrate the practice of meaningful two-way communication
- Demonstrate influence on others by persuasively presenting thoughts and ideas
- Demonstrate assertiveness skills to deal with unreasonable requests and/or refusals that would compromise practice or consumer care
- Demonstrate a positive, supportive and appreciative attitude
- Demonstrate the ability to balance the organisation's needs and the customer's needs
- Demonstrate self-control by maintaining composure and keeping emotions in check, even in difficult situations; deal calmly and effectively with stressful situations
- Demonstrate the expressing of opinions and providing information in written and/or verbal form in a manner that does not elicit concern, anger or other adverse response

	» Demonstrate the use of a systematic process for following up that demonstrates written reports have been received and understood<br>» Evaluate the means by which responses to input to the work environment are monitored<br>» Seek practical ways to overcome barriers to communication<br>» Ensure language representation (e.g., interpreters) at meetings when appropriate<br>» Demonstrate tackling difficult situations and resolving disputes between staff<br>» Demonstrate giving both positive and negative feedback sensitively<br>» Demonstrate maintaining and improving communication<br>» Demonstrate the process of giving and requesting feedback frequently<br>» Demonstrate the ability to communicate effectively with shop stewards and union representatives
6.2.1 Practice cultural awareness	» Demonstrate cultural awareness and sensitivity, treating all people with fairness, respect and dignity<br>» Demonstrate anti-discriminatory practices in the organisation, including HR and disciplinary procedures<br>» Demonstrate cultural sensitivity, equality and fairness at all levels of the organisation<br>» Demonstrate challenging discriminatory behaviour directly and sensitively<br>» Demonstrate acting in a non-discriminatory way toward individuals and groups<br>» Demonstrate the process of integrating cultural awareness in learning and development approaches<br>» Demonstrate avoiding stereotypical responses by examining one's own behaviour and bias<br>» Demonstrate managing cultural diversity in teams and make the most of differences<br>» Demonstrate the use of strategies and/or resources for communicating effectively with people from different cultural backgrounds<br>» Supply information relating to values, beliefs and cultural backgrounds of consumers that may influence the way professional services are provided
6.3 Utilize problem-solving skills	» Evaluate information to solve problems in a sensitive and ethical manner<br>» Demonstrate managing day to day and complex problems in a timely manner<br>» Evaluate problem situations and their step-by-step transformation based on planning and reasoning, without apportioning blame<br>» Demonstrate the process of critically reviewing, analysing, synthesizing, comparing and interpreting information; drawing conclusions from relevant and/or missing information; and understanding the relationship among facts and apply this understanding when solving problems<br>» Evaluate a range of possible approaches/strategies that are effective for resolving conflict in the workplace (e.g., negotiation, collaborative problem-solving, mediation, arbitration)<br>» Evaluate an open environment that encourages people to work together<br>» Demonstrate resolving conflicts as they arise

- Demonstrate creativity through questioning, attempting to improve on ideas, applying other experiences and working toward action in problem solving
- Analyse facts and thoughts into strengths and weaknesses
- Demonstrate thinking in a careful way to solve problems, analyse data, and recall and apply information, involving others
- Demonstrate goal-directed thinking and action in situations in which no routine solutions exist
- Demonstrate maintaining appropriate ethical and moral standards in resolving problems
- Analyse alternative courses of action, using cognitive processes such as memory, thinking and evaluation
- Demonstrate mapping likely consequences of decisions to choose the best course of action
- Demonstrate asking other people to help with solving problems
- Evaluate difficult or unusual situations to develop unique approaches and useful solutions
- Demonstrate committing to a solution in a timely manner, and developing a realistic approach for applying the chosen solution; evaluating the outcome of the solution to see if further action is needed, and identify lessons learned
- Evaluate the impact of conflict in the workplace (e.g., tension, low morale, absenteeism, system or service failure, aggressive or uncooperative behaviours)
- Demonstrate the practise of following up on problems to ensure they are fixed
- Determine situations where referral is warranted (e.g., severe emotional distress, intractable dispute)

6.3.1 Negotiate

- Demonstrate the principles of negotiation and aim for a win-win outcome
- Demonstrating managing and resolving relationship issues within and outside the team
- Determine solution-focussed approaches to further the organisation's mission
- Demonstrate building consensus among parties
- Evaluate alternative ways of doing things
- Determine where fair approaches are being utilised to resolve issues
- Demonstrate seeking to reach constructive solutions while maintaining positive working relationships
- Demonstrate adapting style to take into account cultural differences regarding negotiation
- Demonstrate building consensus at a high level for the benefit of all parties

Basal Technology Competency(ies):

7.4 Temperature and monitoring

7.6 ERP includes function of LMIS

7.12 Basic Office Skills

7.13 Have a command of technology

7.4 Temperature and monitoring	» Analyse the current technology approaches for Temperature monitoring and sensors<br>» Evaluate what is required to implement this technology in a country context<br>» Evaluate infrastructure requirement for heat-sensitive items at peripheral 'lower level' of SC depending on volume and nature of items to handle;<br>» Analyse regular preventive maintenance and calibration of cold-chain equipment with biomedical engineers or logisticians<br>» Evaluate the policy for temperature monitoring tools during transportation and storage<br>» Demonstrate the steps involved in health logistic management cycle: selection-order-delivery-reception-storage-distribution-inventory<br>» Determine hardware qualification and system validation of temperature monitoring<br>» Evaluate mass data management of temperature monitoring
7.6 ERP includes function of LMIS	» Evaluate a Master Data Management System<br>» Demonstrate an understanding of the critical components of an ERP system<br>» Demonstrate an understanding of the meaning of common document types found within an ERP/LMIS<br>» Demonstrate an understanding of the logic of data visibility within an ERP<br>» Demonstrate the importance of data integrity, record management and information security
7.12 Basic Office Skills	Demonstrate a good understanding of common presentation authoring packages<br>Demonstrate a good understanding of common spreadsheet authoring packages<br>Demonstrate a good understanding of common document authoring packages<br>Demonstrate a good understanding of common email authoring packages<br>Demonstrate a good understanding of common instant messaging packages<br>Demonstrate a good understanding of common video conferencing authoring packages
7.13 Have a command of technology	» Demonstrate the use of technology suitable to the job held<br>» Monitor new developments and technologies in the sector<br>» Experiment with new technologies and recognising potential benefits for the sector<br>» Ensure that resources and support are provided across the organisation to enable colleagues to make the best use of available technology<br>» Determine what the organisations' strategy is for technology use<br>» Determine how to minimise environmental damage through technology use<br>» Analyse the use of technology to maximise effectiveness and efficiency<br>» Analyse the use of field-based technology (e.g., radio, general packet radio service (GPRS), satellite phone)

Key Performance Indicators:		
» Adherence to medicines budget<br>» Cost savings delivery<br>» Medicine availability<br>» Adherence to strategic sourcing principles for different categories		
**Training:**	**Qualifications Available:**	**Certifications Available:**
» Contract Management<br>» Analytical Skills<br>» Competitive Billing<br>» Negotiation	» Bachelor of Business Science<br>» Bachelor of Accounting Honours	» Occupational Certificate: Supply and Distribution Manager<br>» SPSM3 TM (Enterprise-Wide Procurement Influence)<br>» CIPS Level 5 Advanced Diploma in Procurement and Supply

Lead – Receiving and Inventory Management

Organisation:				
**Public Health Organisation – Intermediate Level**				
Competency Level:	Serial Number:	Primary Process:	Job Role:	Supervises:
Managerial	3-33-37-4	Deliver	Lead – Receiving and Inventory Management	Officer – Receiving and Inventory Management, Clerk – Receiving and Storage

Contributions	Manages the receiving team in the context department, manages the warehouse personnel to perform according to best practices as defined by the Supply Chain model of the Public Health Supply Chain. Monitors the order cycle metrics for receiving and put away, inventory put away accuracy.
Technical Competency(ies):	2.5 Ensure quality of products<br>3.1 Undertake storage, warehousing and inventory management<br>3.1.1 Manage storage of commodities during emergency<br>3.3 Supply commodities to sections within a facility<br>3.5 Manage disposal of products (e.g. expired, damaged, redundant products)<br>3.5.1 Define and direct process for managing redundant and returned stock<br>3.7 Manage re-packing of products

2.5 Ensure quality of products	» Analyse the use and monitoring process for prequalification of suppliers<br>» Evaluate the process of ensuring that health commodities are not counterfeit and meet quality standards through certification (ISO, WHO GMP<br>» Implement, conduct and maintain a reporting system of pharmacovigilance (e.g., report adverse drug reactions, quality defects, batch recall) and equipment faults<br>» Analyse the process of inspecting products when delivered and during storage to catch defects or problems before they are given to, or needed by, clients<br>» Demonstrate compliance with good distribution and good storage practices (temperature monitoring in the warehouse, transportation and distribution)<br>» Evaluate product-specific shelf-life by which the product are acceptable or rejected (e.g. no less than 1 year for most of drugs, and less for short shelf-life like laboratory)
3.1 Undertake storage, warehousing and inventory management	» Analyse methods to distribute products among suppliers, distribution centres, warehouses and customers through a logistics network<br>» Analyse a total systems approach to designing and managing the entire flow of information, materials, and services<br>» Determine the choice of equipment or materials based on suitability for intended use, accuracy, safety of use and cost<br>» Analyse the warehouse configuration to have formal storage locations that identify the row, rack section, level and shelf location, typically with an alphanumeric location bar code or label

	» Evaluate how medicines are appropriately stored, considering temperature, access and cleanliness<br>» Determine the appropriate use of, and ability to maintain, the cold chain<br>» Analyse the movement and storage of materials within a warehouse<br>» Analyse the process of securing the medical store and limiting access to staff<br>» Analyse organisation processes, instructions, rules and parameters for warehouse and inventory management and use of equipment (e.g., annual stock turns, expiry date tracking, stock procurement, rotation and retrieval, equipment maintenance)<br>» Analyse policies and procedures for stock handling, distribution and withdrawal consistent with maintaining safety (e.g., policies for distribution of concentrated electrolyte solutions and cytotoxics, identification of cytotoxic drug products within the work environment, separation of like-named or like-packaged products)<br>» Analyse requirements for information on the supplies being stored<br>» Analyse processes to consolidate several items into larger units for fewer handlings<br>» Analyse methods of stock rotation (e.g., first in first out, FIFO, or first to expire first out, FEFO)<br>» Analyse the process of entering the quantity received and updating the warehouse management system software with the stock on hand when each order is delivered<br>» Determine required levels of stock and/or equipment in specific situations<br>» Measure the accuracy of verification of rolling stocks<br>» Implement the methodology for monitoring the location and condition of the supplies being stored<br>» Analyse information on the storage locations and facilities<br>» Analyse any problems in using the storage locations and facilities
3.1.1 Manage storage of commodities during emergency	» Analyse the different types of warehouses available for emergency or disaster supply<br>» Analyse the choice of storage site for emergency or disaster supply<br>» Evaluate the staff required for the storage of emergency or disaster supply<br>» Determine the equipment and material required for the warehousing of emergency or disaster supply<br>» Conduct a rapid assessment of logistical needs in emergencies
3.3 Supply commodities to sections within a facility	» Review imprest list and their implementations in the context<br>» Analyse and validate the requisition from different health care services within the facility
3.5 Manage disposal of products (e.g. expired, damaged, redundant products)	
3.5.1 Define and direct process for	» Evaluate information on the supplies being returned

managing redundant and returned stock	» Collate, prepare and analyse data on the flow of returned supplies<br>» Resolve any problems with the flow of returned supplies<br>» Recommend options for improving the flow of returned supplies<br>» Analyse procedures for monitoring the flow of returned supplies<br>» Determine recall procedures to be used in response to a product recall notice or to access the information promptly.<br>» Demonstrate knowledge of issues relevant to the urgency and scope of action required in response to a product recall notice<br>» Analyse the information provided in the reports when following up on medication incidents<br>» Evaluate follow-up strategies likely to be effective in preventing recurrence (e.g., root cause analysis)<br>» Evaluate an integrated healthcare waste management plan<br>» Evaluate guides and procedures for collection, sorting, transport and disposal<br>» Evaluate pre-disposal inspections<br>» Evaluate the prerequisites for products disposal<br>» Evaluate product disposal report
3.7 Manage re-packing of products	» Determine when re-packing or pre-packs are required<br>» Determine a safe system for repacking

Personal/Management Competency(ies):	5.5 Manage and plan projects<br>5.5.2 Execute strategic decision making<br>5.5.3 Manage partnerships<br>6.1 Demonstrate generic skills (e.g., literacy, numeracy, technology)<br>6.1.1 Exhibit high understanding of literacy and numeracy<br>6.2 Demonstrate strong communication skills<br>6.2.1 Practice cultural awareness

5.5 Manage and plan projects	
5.5.2 Execute strategic decision making	» Determine how one can make decisions regarding one's own workload and area of responsibility<br>» Determine the process of prioritizing competing tasks and perform them quickly and efficiently according to their importance; finding new ways of organizing or planning work to accomplish it more efficiently<br>» Evaluate the proper time and space for consultation around decisions being made.<br>» Demonstrate the process of considering the input of resources including staff needed<br>» Analyse assumptions against facts<br>» Determine the key issues in a complex situation and come to the heart of the problem quickly<br>» Evaluate relevant information before making decisions

» Provide input to strategic decisions in the interest of the organisation's goals

» Evaluate when to analyse, evaluate and execute tough decisions when necessary

» Analyse the impact of decisions on others' work or team goals

» Communicate decisions and ensure they are incorporated into policies and processes

» Demonstrate inclusive and consultative strategic planning to establish strategic objectives

» Evaluate emerging trends and practices

» Analyse the macro and long-term consequences of decisions

5.5.3 Manage partnerships

» Demonstrate the concepts of partnership working

» Determine the process of supporting implementation of partnership programmes

» Analyse the process of experimenting with and trialling new ideas with partners

» Analyse partnerships to deliver programme and increase impact

» Analyse innovation and creativity in partnership work

» Communicate key information with partner members

» Involve and value partners in all aspects of programming

» Analyse problems with supply chain relationships

» Demonstrate the process of communicating effectively with nurses, doctors and other members of the healthcare team

» Analyse the process of responding to consumer complaints or comments about services and/or advice received

» Analyse collaboration and consultation across the sector and encourage the team to work with others to gain market insight

» Encourage input from other key actors in the humanitarian sector and develop relationships with a cross-section of actors

» Analyse the process of meeting the reporting requirements of vertical programmes

» Analyse the structure of the health system at a national level and explain this to others

» Analyse the roles of other members of the healthcare team (including with consumers) in a way that engenders understanding and confidence in the team and its members

» Analyse the structure of the organisation, environment and/or service in which they work

» Demonstrate how vertical programmes work within the health system

» Evaluate the communication network established to achieve work outcomes

» Demonstrate knowledge of the structure of the health system at the provincial/regional level and explain this to others

» Analyse the roles and functions of government agencies in regulating and supporting supply chain organisations

» Demonstrate knowledge of change management (e.g. PtD Theory of Change)

6.1 Demonstrate generic skills (e.g., literacy, numeracy, technology)	
6.1.1 Exhibit high understanding of literacy and numeracy	» Encourage speaking clearly and confidently, organizing information in a logical manner, and considering voice tone and pace<br>» Demonstrate responding to verbal messages and other cues (active listening)<br>» Demonstrate expressing ideas and opinions clearly in written and verbal form<br>» Demonstrate communicating information accurately, concisely and confidently in writing and verbally<br>» Demonstrate a level of mathematics suitable to the job held<br>» Demonstrate knowledge of visual presentation techniques, including charting, histograms and flow sheets<br>» Demonstrate reading and comprehending at a level necessary to properly complete duties of the position<br>» Demonstrate the process of interpreting written information in documents, such as reports, SOPs, LMIS forms, graphs, calendars, schedules, notices and directions<br>» Demonstrate paying attention to detail and identifying the main ideas, detecting inconsistencies and identifying missing information in documents<br>» Demonstrate computer skills — such as using Word, Excel, PowerPoint and the internet — suitable to the job level<br>» Demonstrate the process of analysing and validating KPIs<br>» Participate in the evaluation of activities that act as standards against which the KPIs are measured<br>» Demonstrate the process of performing critical self-review on work before submission
6.2 Demonstrate strong communication skills	» Demonstrate being truthful and trustworthy, and supplying accurate information at all times<br>» Demonstrate working as part of a workplace team<br>» Determine vocabulary and communication style and form for both written and verbal communication that is appropriate for the situation, audience and material being communicated (e.g., avoid unnecessary jargon, clearly explain medical and SCM terminology)<br>» Demonstrate open communication within constraints of confidentiality<br>» Analyse communications about logistics<br>» Demonstrate participation in meetings, expressing one's opinions, being aware of others' needs, and being appropriately assertive when required<br>» Demonstrate perspective into the point of view of others, understanding their needs and goals<br>» Demonstrate the practise of building trust, rapport and credibility with others<br>» Determine the communication needs and concerns of others and respond to them

» Demonstrate avoiding conflict between work and personal interests
» Demonstrate the practice of meaningful two-way communication
» Demonstrate influence on others by persuasively presenting thoughts and ideas
» Demonstrate assertiveness skills to deal with unreasonable requests and/or refusals that would compromise practice or consumer care
» Demonstrate a positive, supportive and appreciative attitude
» Demonstrate the ability to balance the organisation's needs and the customer's needs
» Demonstrate self-control by maintaining composure and keeping emotions in check, even in difficult situations; deal calmly and effectively with stressful situations
» Demonstrate the expressing of opinions and providing information in written and/or verbal form in a manner that does not elicit concern, anger or other adverse response
» Demonstrate the use of a systematic process for following up that demonstrates written reports have been received and understood
» Evaluate the means by which responses to input to the work environment are monitored
» Seek practical ways to overcome barriers to communication
» Ensure language representation (e.g., interpreters) at meetings when appropriate
» Demonstrate tackling difficult situations and resolving disputes between staff
» Demonstrate giving both positive and negative feedback sensitively
» Demonstrate maintaining and improving communication
» Demonstrate the process of giving and requesting feedback frequently
» Demonstrate the ability to communicate effectively with shop stewards and union representatives

6.2.1 Practice cultural awareness

» Demonstrate cultural awareness and sensitivity, treating all people with fairness, respect and dignity
» Demonstrate anti-discriminatory practices in the organisation, including HR and disciplinary procedures
» Demonstrate cultural sensitivity, equality and fairness at all levels of the organisation
» Demonstrate challenging discriminatory behaviour directly and sensitively
» Demonstrate acting in a non-discriminatory way toward individuals and groups
» Demonstrate the process of integrating cultural awareness in learning and development approaches
» Demonstrate avoiding stereotypical responses by examining one's own behaviour and bias
» Demonstrate managing cultural diversity in teams and make the most of differences

	» Demonstrate the use of strategies and/or resources for communicating effectively with people from different cultural backgrounds<br>» Supply information relating to values, beliefs and cultural backgrounds of consumers that may influence the way professional services are provided
Basal Technology Competency(ies):	7.4 Temperature and monitoring<br>7.6 ERP includes function of LMIS<br>7.12 Basic Office Skills<br>7.13 Have a command of technology
7.4 Temperature and monitoring	» Analyse the current technology approaches for Temperature monitoring and sensors<br>» Evaluate what is required to implement this technology in a country context<br>» Evaluate infrastructure requirement for heat-sensitive items at peripheral 'lower level' of SC depending on volume and nature of items to handle;<br>» Analyse regular preventive maintenance and calibration of cold-chain equipment with biomedical engineers or logisticians<br>» Evaluate the policy for temperature monitoring tools during transportation and storage<br>» Demonstrate the steps involved in health logistic management cycle: selection-order-delivery-reception-storage-distribution-inventory<br>» Determine hardware qualification and system validation of temperature monitoring<br>» Evaluate mass data management of temperature monitoring
7.6 ERP includes function of LMIS	» Evaluate a Master Data Management System<br>» Demonstrate an understanding of the critical components of an ERP system<br>» Demonstrate an understanding of the meaning of common document types found within an ERP/LMIS<br>» Demonstrate an understanding of the logic of data visibility within an ERP<br>» Demonstrate the importance of data integrity, record management and information security
7.12 Basic Office Skills	» Demonstrate a good understanding of common presentation authoring packages<br>» Demonstrate a good understanding of common spreadsheet authoring packages<br>» Demonstrate a good understanding of common document authoring packages<br>» Demonstrate a good understanding of common email authoring packages<br>» Demonstrate a good understanding of common instant messaging packages<br>» Demonstrate a good understanding of common video conferencing authoring packages
7.13 Have a command of technology	» Demonstrate the use of technology suitable to the job held<br>» Monitor new developments and technologies in the sector<br>» Experiment with new technologies and recognising potential benefits for the sector

- Ensure that resources and support are provided across the organisation to enable colleagues to make the best use of available technology
- Determine what the organisations' strategy is for technology use
- Determine how to minimise environmental damage through technology use
- Analyse the use of technology to maximise effectiveness and efficiency
- Analyse the use of field-based technology (e.g., radio, general packet radio service (GPRS), satellite phone)

Advanced Technology Competency(ies):	7.7 Automation
**7.7 Automation**	» Evaluate automation as a value-add capability for relevant global health use cases

Key Performance Indicators:

- Availability - Stock out rate - Number of missed line item quantity deliveries due to stock out versus total line item quantity deliveries
- Availability - Stock levels - Current on hand stock versus Planned on hand stock
- Safe product handling - Number of products handled during period out of conformance versus Number of products handled during period expressed as a % with prior period comparisons
- % Product Transferred On-Time to Demand Requirement. Actual Number of products transferred outside of the Time Limits vs Number of products Transferred.
- Transfer Product Cycle Time - Actual Transfer Cycle Time versus Planned Transferred Cycle Time
- Accurate pick - Picking Accuracy number of order lines picked inaccurately divided by the total order lines picked (SKU and Quantity)
- On-time delivery - Actual Delivery Date & Time Vs Promised Delivery Data and Time
- Customer order fulfilment cycle time & rate - Actual Order Fulfilment Cycle Time versus Promised Customer Order Fulfilment Cycle time
- Customer Incidents Report number of incidents by month/week/day comparison with previous periods
- No of Order Lines shipped with incorrectly captured Items - Number of order lines with incorrect items/SKU's versus total number of order lines shipped as a %
- Regulatory - % of procured products that meet Stringent Regulatory Authority (SRA) or WHO standards
- Warehousing and Inventory Management OTIF - On time and in full
- Waste Management - Existence of supply chain management protocols for disposal of medical waste and management of unusable products
- Number of order lines delivered to incorrect end-user - Number of orders incorrectly addressed versus number of orders processed expressed as a %
- Number of order lines delivered out of Quality Specifications - Number of Item/SKU delivered out of specification e.g. Cold Chain infractions, Expired products versus total Number of order lines delivered as a %. A comparative graph would be an appropriate tool for the measurement of this metric.
- Warehouse Utilization - Volume used vs Volume Available
- Line Items SKU's handled vs Items / SKU's Planned over a planning horizon
- Number of warehouse locations used versus warehouse locations available (Pallet Positions may be used in larger warehouses)

» Warehouse Utilization - Volume used vs Volume Available

Training:	Qualifications Available:	Certifications Available:
» Data management<br>» ERP Systems<br>» Legislation and Standards<br>» Supplier Relationship Management (SRM)<br>» 3-way Receiving Match<br>» Code Handling/RFID (if available)<br>» Cross Docking<br>» Discrepancy Reporting and Resolution<br>» ID & Damage Inspection<br>» Inventory Management<br>» MSDS/Environmental Interpretation<br>» Property Control and Disposition<br>» Return Management<br>» Pharmacovigilance	» Bachelor of Commerce in Logistics Management<br>» Bachelor of Business Administration in Logistics and Supply Chain Management<br>» Bachelor of Business Management	» Occupational Certificate: Health Information Manager<br>» APICS Certified in logistics transportation and distribution certificate<br>» SPSM Level 4 (External Procurement Influence)

Lead – Returns and Disposal

Organisation:

Public Health Organisation – Intermediate Level

Competency Level:	Serial Number:	Primary Process:	Job Role:	Supervises:
Managerial	3-351-352-3	Return	Lead – Returns and Disposal	-

Contributions	Ensures regulatory compliance of returns. Manages the quarantine requirements for material returns and disposition of return materials. Ensures that the material being returned is accounted for at all times.
Technical Competency(ies):	3.5 Manage disposal of products (e.g. expired, damaged, redundant products)<br>3.5.1 Define and direct process for managing redundant and returned stock<br>3.5.2 Manage process for disposal of returned stock

3.5 Manage disposal of products (e.g. expired, damaged, redundant products)

3.5.1 Define and direct process for managing redundant and returned stock

- Confirm information on the supplies being returned
- Describe the flow of returned supplies
- Identify any problems with the flow of returned supplies
- Integrate options for improving the flow of returned supplies
- Apply procedures for monitoring the flow of returned supplies
- Describe and/or demonstrate recall procedures to be used in response to a product recall notice or to access the information promptly.
- Identify issues relevant to the urgency and scope of action required in response to a product recall notice
- Identify the information required for reporting and procedures with regards to following up on medication incidents
- Identify follow-up strategies likely to be effective in preventing recurrence (e.g., root cause analysis)
- Describe an integrated healthcare waste management plan
- Describe guides and procedures for collection, sorting, transport and disposal
- Conduct pre-disposal inspections
- Describe the prerequisites for products disposal
- Generate product disposal report

3.5.2 Manage process for disposal of returned stock	» Dispose of expired medications and/or medical equipment according to national policy<br>» Describe and/or use an appropriate recording system for disposal<br>» Describe the disposal of specific individual items of greatest risk with appropriate care (e.g., oncology medicine)
Personal/Management Competency(ies):	6.1 Demonstrate generic skills (e.g., literacy, numeracy, technology)<br>6.1.1 Exhibit high understanding of literacy and numeracy<br>6.2 Demonstrate strong communication skills<br>6.2.1 Practice cultural awareness
6.1 Demonstrate generic skills (e.g., literacy, numeracy, technology)	
6.1.1 Exhibit high understanding of literacy and numeracy	» Speak clearly and confidently, organize information in a logical manner, and consider voice tone and pace<br>» Respond to verbal messages and other cues (active listening)<br>» Express ideas and opinions clearly in written and verbal form<br>» Communicate information accurately, concisely and confidently in writing and verbally<br>» Practice a level of mathematics suitable to the job held<br>» Describe visual presentation techniques, including charting, histograms and flow sheets<br>» Read and comprehend at a level necessary to properly complete duties of the position<br>» Interpret written information in documents, such as reports, SOPs, LMIS forms, graphs, calendars, schedules, notices and directions<br>» Pay attention to detail and identify the main ideas, detect inconsistencies and identify missing information in documents<br>» Apply computer skills — such as using Word, Excel, PowerPoint and the internet — suitable to the job level<br>» Describe the process of analysing and validating KPIs<br>» Participate in the evaluation of activities that act as standards against which the KPIs are measured<br>» Apply the process of performing critical self-review on work before submission
6.2 Demonstrate strong communication skills	» Be truthful and trustworthy, and supply accurate information at all times<br>» Work as part of a workplace team<br>» Describe vocabulary and communication style and form for both written and verbal communication that is appropriate for the situation, audience and material being communicated (e.g., avoid unnecessary jargon, clearly explain medical and SCM terminology)<br>» Encourage open communication within constraints of confidentiality<br>» Organize communications about logistics

	» Encourage participation in meetings, expressing one's opinions, being aware of others' needs, and being appropriately assertive when required<br>» Apply perspective into the point of view of others, understanding their needs and goals<br>» Encourage building trust, rapport and credibility with others<br>» Anticipate the communication needs and concerns of others and respond to them<br>» Avoid conflict between work and personal interests<br>» Encourage meaningful two-way communication<br>» Influence others by persuasively presenting thoughts and ideas<br>» Apply assertiveness skills to deal with unreasonable requests and/or refusals that would compromise practice or consumer care<br>» Maintain a positive, supportive and appreciative attitude<br>» Apply the ability to balance the organisation's needs and the customer's needs<br>» Use self-control by maintaining composure and keeping emotions in check, even in difficult situations; deal calmly and effectively with stressful situations<br>» Encourage the expressing of opinions and providing information in written and/or verbal form in a manner that does not elicit concern, anger or other adverse response<br>» Apply the use of a systematic process for following up that demonstrates written reports have been received and understood<br>» Describe the means by which responses to input to the work environment are monitored<br>» Seek practical ways to overcome barriers to communication<br>» Identify language representation (e.g., interpreters) at meetings when appropriate<br>» Tackle difficult situations and resolve disputes between staff<br>» Give both positive and negative feedback sensitively<br>» Maintain and improve communication<br>» Apply the process of giving and requesting feedback frequently<br>» Apply the ability to communicate effectively with shop stewards and union representatives
6.2.1 Practice cultural awareness	» Describe cultural awareness and sensitivity, treating all people with fairness, respect and dignity<br>» Describe anti-discriminatory practices in the organisation, including HR and disciplinary procedures<br>» Establish cultural sensitivity, equality and fairness at all levels of the organisation<br>» Challenge discriminatory behaviour directly and sensitively<br>» Act in a non-discriminatory way toward individuals and groups<br>» Integrate cultural awareness in learning and development approaches<br>» Avoid stereotypical responses by examining one's own behaviour and bias<br>» Manage cultural diversity in teams and make the most of differences

- Describe strategies and/or resources for communicating effectively with people from different cultural backgrounds
- Elicit information relating to values, beliefs and cultural backgrounds of consumers that may influence the way professional services are provided

Basal Technology Competency(ies):	7.6 ERP includes function of LMIS<br>7.12 Basic Office Skills<br>7.13 Have a command of technology
7.6 ERP includes function of LMIS	» Support a Master Data Management System<br>» Describe the critical components of an ERP system<br>» Describe the meaning of common document types found within an ERP/LMIS<br>» Describe the logic of data visibility within an ERP<br>» Describe the importance of data integrity, record management and information security
7.12 Basic Office Skills	» Have a good understanding of common presentation authoring packages<br>» Have a good understanding of common spreadsheet authoring packages<br>» Have a good understanding of common document authoring packages<br>» Have a good understanding of common email authoring packages<br>» Have a good understanding of common instant messaging packages<br>» Have a good understanding of common video conferencing authoring packages
7.13 Have a command of technology	» Apply the use of technology suitable to the job held<br>» Provide input on monitoring new developments and technologies in the sector<br>» Identify new technologies and recognising potential benefits for the sector<br>» Identify resources and support that are provided across the organisation to enable colleagues to make the best use of available technology<br>» Ensure that the organisation has a strategy for technology use<br>» Describe how to minimise environmental damage through technology use<br>» Use technology to maximise effectiveness and efficiency<br>» Use field-based technology (e.g., radio, general packet radio service (GPRS), satellite phone)
Advanced Technology Competency(ies):	7.7 Automation
7.7 Automation	» Describe automation as a value-add capability for relevant global health use cases

Key Performance Indicators:

- Availability - Stock out rate - Number of missed line item quantity deliveries due to stock out versus total line item quantity deliveries
- Availability - Stock levels - Current on hand stock versus Planned on hand stock
- Safe product handling - Number of products handled during period out of conformance versus Number of products handled during period expressed as a % with prior period comparisons
- Customer Incidents Report number of incidents by month/week/day comparison with previous periods
- No of Order Lines shipped with incorrectly captured Items - Number of order lines with incorrect items/SKU's versus total number of order lines shipped as a %
- Waste Management - Existence of supply chain management protocols for disposal of medical waste and management of unusable products
- Number of Order Lines delivered to incorrect end-user - Number of orders incorrectly addressed versus number of orders processed expressed as a %
- Number of Order Lines delivered out of Quality Specifications - Number of Item/SKU delivered out of specification e.g. Cold Chain infractions, Expired products versus total Number of order lines delivered as a %. A comparative graph would be an appropriate tool for the measurement of this metric.
- % Product Transferred On-Time to Demand Requirement. Actual Number of products transferred outside of the Time Limits vs Number of products Transferred.
- Transfer Product Cycle Time - Actual Transfer Cycle Time versus Planned Transferred Cycle Time
- Accurate pick - Picking Accuracy number of order lines picked inaccurately divided by the total order lines picked (SKU and Quantity)
- On-time delivery - Actual Delivery Date & Time Vs Promised Delivery Data and Time
- Customer order fulfilment cycle time & rate - Actual Order Fulfilment Cycle Time versus Promised Customer Order Fulfilment Cycle time
- Warehousing and Inventory Management OTIF - On time and in full
- Regulatory - % of procured products that meet Stringent Regulatory Authority (SRA) or WHO standards

Training:	Qualifications Available:	Certifications Available:
» ERP Systems<br>» Inventory Management<br>» Return Management<br>» Regulatory Policy Management<br>» Logistics Management	» Bachelor of Commerce in Logistics Management<br>» Bachelor of Business Administration in Logistics and Supply Chain Management	» Occupational Certificate: Maintenance Planner

Manager – Performance Management

Organisation: Public Health Organisation – Intermediate Level				
Competency Level:	Serial Number:	Primary Process:	Job Role:	Supervises:
Managerial	5-531-532-3	Enable – SC Performance	Manager – Performance Management	-

Contributions	Creates and Maintains the data collection framework to enable the measurement of the metrics prescribed in the Public Health System Organisation, Operating Model and Supply Chain Strategy
Personal/Management Competency(ies):	5.5 Manage and plan projects<br>5.5.1 Develop and direct project plans<br>5.5.2 Execute strategic decision making<br>5.5.3 Manage partnerships<br>5.5.4 Direct/participate in teamwork<br>5.6 Manage financial activities<br>6.1 Demonstrate generic skills (e.g., literacy, numeracy, technology)<br>6.1.1 Exhibit high understanding of literacy and numeracy<br>6.2 Demonstrate strong communication skills<br>6.2.1 Practice cultural awareness

5.5 Manage and plan projects

5.5.1 Develop and direct project plans

- Anticipate obstacles and critical events for ensuring the six rights of logistics management (right quantity, product, condition, place, time and cost) and develop contingency plans to address them; monitor progress and take necessary corrective action when needed
- Describe an operational plan that is consistent with the strategic plan, links specific goals and strategies, and identifies the performance indicators to be used to measure achievements
- Apply the use of information gathering techniques from stakeholders, analysing situations and identifying implications to make correct decisions
- Answer the questions, “Where are we going?” and “How are we going to get there?” and create a specific and purposeful path to achieve this.
- Apply the process reference model to managing supply chain processes (i.e., integrating business process re-engineering, benchmarking and process measurement)
- Capture the current state of a process and derive the desired future state
- Select and apply tools or technological solutions to frequently encountered problems
- Provide staff with the necessary tools to carry out plans

	» Describe the process of monitoring progress and making changes as required<br>» Facilitate meetings for planning, organisation and monitoring logistics activities<br>» Describe the process of ensuring staff are aware that they are accountable for achieving the desired results<br>» Describe how planning, organising and managing resources will bring about the successful completion of projects<br>» Describe the key features of a successful change management strategy
5.5.2 Execute strategic decision making	» Make decisions regarding one's own workload and area of responsibility<br>» Prioritize competing tasks and perform them quickly and efficiently according to their importance; find new ways of organizing or planning work to accomplish it more efficiently<br>» Ensure proper time and space for consultation around decisions being made.<br>» Consider the input of resources including staff needed<br>» Check assumptions against facts<br>» Identify the key issues in a complex situation and come to the heart of the problem quickly<br>» Gather relevant information before making decisions<br>» Describe the process of providing input for making strategic decisions in the interest of the organisation's goals<br>» Describe when to analyse, evaluate and execute tough decisions when necessary<br>» Consider the impact of decisions on others' work or team goals<br>» Describe the process to communicate decisions and ensure they are incorporated into policies and processes<br>» Describe inclusive and consultative strategic planning to establish strategic objectives<br>» Identify and support emerging trends and practices<br>» Describe the macro and long-term consequences of decisions
5.5.3 Manage partnerships	» Apply the concepts of partnership working<br>» Support implementation of partnership programmes<br>» Describe the process of experimenting with and trialling new ideas with partners<br>» Identify partnerships to deliver programme and increase impact<br>» Use innovation and creativity in partnership work<br>» Describe the process of communicating key information with partner members<br>» Describe the process of involving and valuing partners in all aspects of programming<br>» Identify problems with supply chain relationships

	» Communicate effectively with nurses, doctors and other members of the healthcare team<br>» Respond to consumer complaints or comments about services and/or advice received<br>» Foster collaboration and consultation across the sector and encourage the team to work with others to gain market insight<br>» Assist in sharing input from other key actors in the humanitarian sector and develop relationships with a cross-section of actors<br>» Meet the reporting requirements of vertical programmes<br>» Outline the structure of the health system at a national level<br>» Discuss the role of other members of the healthcare team (including with consumers) in a way that engenders understanding and confidence in the team and its members<br>» Describe the structure of the organisation, environment and/or service in which they work<br>» Describe how vertical programmes work within the health system<br>» Describe the communication network established to achieve work outcomes<br>» Describe the structure of the health system at the provincial/regional level and explain this to others<br>» List the roles and functions of government agencies in regulating and supporting supply chain organisations<br>» Describe knowledge of change management (e.g. PtD Theory of Change)
5.5.4 Direct/participate in teamwork	» Describe the process of working with team members within their area of authority to establish achievable goals and strategies that are consistent with the objectives established for the organisation as a whole<br>» Describe an organisational chart that shows the lines of reporting and responsibility among staff<br>» Actively contribute a perspective and make a positive contribution to team-based problem solving and decision making<br>» Provide feedback, encouragement and support to team members for progressing strategic goals<br>» Define team performance in relation to the organisation’s mission and goals<br>» Encourage teams to think for themselves and resolve problems<br>» Encourage and harness diversity within the team to boost team effectiveness<br>» Encourage and support the team to work through its stages of development and perform well<br>» Describe roles and responsibilities in relation to employees’ expertise and the expectations of collaborating team members<br>» Describe where their position fits in the structure (organisation, SC, healthcare sector) , the responsibilities their position entails and the accountabilities linked to the position

5.6 Manage financial activities	» Manage all resources with care, applying budgetary principles<br>» Participate in meeting budgets and donor requirements<br>» Recognise budgets (national, regional, etc.) as necessary for work (e.g., wages budget, touring budget, stationery budget, project budgets for using NGO funds)<br>» Describe the process of producing timely and clear financial reports for funders and donors<br>» Describe the process of keeping records following government budgeting, accounting and financial practices<br>» Describe the process of maintaining an overview of multiple budgets from multiple sources<br>» Providing input during seeking and using information on financial funding requirements<br>» Ensure timely decision making with regard to financial shortfalls<br>» Provide budget tracking for logistics activities according to established procedures<br>» Provide input during the analysis of financial statements and explain components of a balance sheet and income statement<br>» Provide input on creating interactive decision support models that allow the development of multiple scenarios and demonstrating the sensitivity of multiple independent variables<br>» Describe the success or failure of a business using financial accounting<br>» Calculate the total system cost of delivering a product or service to the customer<br>» Describe the key considerations for developing a business plan<br>» Describe the structure against which business performance will be monitored (e.g., cost centres, chart of accounts)<br>» List key performance indicators used to monitor business performance (e.g., turnover, profitability)<br>» List strategies for minimising the risk of fraudulent activity (e.g., reconciliation of purchase orders, receipts and payment approvals; dual signatory arrangements for funds transfers)<br>» Describe investigative processes and options for confirming the existence of fraudulent activity (e.g., sample audits, forensic accounting services)<br>» Provide input on preparing funding applications for trusts and grants<br>» Apply the process of ensuring financial transparency<br>» Describe the general monetary value of medicines and equipment<br>» Provide input to securing financing and managing budgets to support distribution operations<br>» Apply the ability to translate financial information into SC language<br>» Apply SC finance knowledge
6.1 Demonstrate generic skills (e.g., literacy, numeracy, technology)	

6.1.1 Exhibit high understanding of literacy and numeracy	»	Speak clearly and confidently, organize information in a logical manner, and consider voice tone and pace
	»	Respond to verbal messages and other cues (active listening)
	»	Express ideas and opinions clearly in written and verbal form
	»	Communicate information accurately, concisely and confidently in writing and verbally
	»	Practice a level of mathematics suitable to the job held
	»	Describe visual presentation techniques, including charting, histograms and flow sheets
	»	Read and comprehend at a level necessary to properly complete duties of the position
	»	Interpret written information in documents, such as reports, SOPs, LMIS forms, graphs, calendars, schedules, notices and directions
	»	Pay attention to detail and identify the main ideas, detect inconsistencies and identify missing information in documents
	»	Apply computer skills — such as using Word, Excel, PowerPoint and the internet — suitable to the job level
	»	Describe the process of analysing and validating KPIs
	»	Participate in the evaluation of activities that act as standards against which the KPIs are measured
	»	Apply the process of performing critical self-review on work before submission
6.2 Demonstrate strong communication skills	»	Be truthful and trustworthy, and supply accurate information at all times
	»	Work as part of a workplace team
	»	Describe vocabulary and communication style and form for both written and verbal communication that is appropriate for the situation, audience and material being communicated (e.g., avoid unnecessary jargon, clearly explain medical and SCM terminology)
	»	Encourage open communication within constraints of confidentiality
	»	Organize communications about logistics
	»	Encourage participation in meetings, expressing one's opinions, being aware of others' needs, and being appropriately assertive when required
	»	Apply perspective into the point of view of others, understanding their needs and goals
	»	Encourage building trust, rapport and credibility with others
	»	Anticipate the communication needs and concerns of others and respond to them
	»	Avoid conflict between work and personal interests
	»	Encourage meaningful two-way communication
	»	Influence others by persuasively presenting thoughts and ideas
	»	Apply assertiveness skills to deal with unreasonable requests and/or refusals that would compromise practice or consumer care

» Maintain a positive, supportive and appreciative attitude
» Apply the ability to balance the organisation's needs and the customer's needs
» Use self-control by maintaining composure and keeping emotions in check, even in difficult situations; deal calmly and effectively with stressful situations
» Encourage the expressing of opinions and providing information in written and/or verbal form in a manner that does not elicit concern, anger or other adverse response
» Apply the use of a systematic process for following up that demonstrates written reports have been received and understood
» Describe the means by which responses to input to the work environment are monitored
» Seek practical ways to overcome barriers to communication
» Identify language representation (e.g., interpreters) at meetings when appropriate
» Tackle difficult situations and resolve disputes between staff
» Give both positive and negative feedback sensitively
» Maintain and improve communication
» Apply the process of giving and requesting feedback frequently
» Apply the ability to communicate effectively with shop stewards and union representatives

6.2.1 Practice cultural awareness

» Describe cultural awareness and sensitivity, treating all people with fairness, respect and dignity
» Describe anti-discriminatory practices in the organisation, including HR and disciplinary procedures
» Establish cultural sensitivity, equality and fairness at all levels of the organisation
» Challenge discriminatory behaviour directly and sensitively
» Act in a non-discriminatory way toward individuals and groups
» Integrate cultural awareness in learning and development approaches
» Avoid stereotypical responses by examining one's own behaviour and bias
» Manage cultural diversity in teams and make the most of differences
» Describe strategies and/or resources for communicating effectively with people from different cultural backgrounds
» Elicit information relating to values, beliefs and cultural backgrounds of consumers that may influence the way professional services are provided

Key Performance Indicators:

- Assess Delivery Performance Cycle Time - Actual Time from assessed requirement to time delivered to end-user compared to committed Delivery Cycle Time as per Supply Chain strategy
- Assess Supplier Performance Cycle Time - Actual Supplier performance Cycle time versus Supplier commitment cycle time
- Inventory accuracy - Cycle count results
- Transportation lead time accuracy - Actual Transportation lead time versus Transportation promised lead time.
- Transport efficiency - Total cost of transport versus planned cost of transport by mode
- In-bound cost of acquisition - Actual total cost of acquisition versus Total Planned cost of acquisition including landed costs
- Manage performance cycle time
- Performance Report - Actual versus Plan reports for all metrics

Training:	Qualifications Available:	Certifications Available:
» Basic Finance<br>» Benchmarking<br>» Lean Manufacturing<br>» Performance Management<br>» Optimization<br>» Quality Management<br>» Six Sigma<br>» Supply Chain Performance Measurements<br>» Total Quality Management (TQM)<br>» Data management<br>» ERP Systems<br>» Installed base management<br>» Interpreting Specifications<br>» Office automation tools<br>» Product Information Management (Product Data Management)	» National Diploma: Financial Markets<br>» National Diploma: Transport Economics	

Lead – Compliance and Risk Management

Organisation:

Public Health Organisation – Intermediate Level

Competency Level:	Serial Number:	Primary Process:	Job Role:	Supervises:
Managerial	5-531-542-3	Enable – Regulatory (Individual) Compliance	Lead – Compliance and Risk Management	-

Contributions	Participates in the Audit and Risk Committee's environmental scanning for Risk. Alerts the committee and relevant members to the probability of risk occurring, investigates and determines possible risk mitigation strategies and actions. Ensures that the Risk enabler process is followed as per the Public Health System Organisation's Operating Model. Updates the risk register. Tests the process for compliance and monitors the risk and compliance metrics for results that are out of the performance bands.
Personal/Management Competency(ies):	5.3 Implement risk management and monitoring and evaluation activities for the supply chain<br>5.3.1 Ensure monitoring and evaluation activities are completed<br>5.4 Manage outsourcing of SCM functions<br>6.1 Demonstrate generic skills (e.g., literacy, numeracy, technology)<br>6.1.1 Exhibit high understanding of literacy and numeracy<br>6.2 Demonstrate strong communication skills<br>6.2.1 Practice cultural awareness

5.3 Implement risk management and monitoring and evaluation activities for the supply chain

5.3.1 Ensure monitoring and evaluation activities are completed

» Describe the periodic evaluations to maintain processes by gathering pertinent information — such as problem symptoms from knowledgeable sources, carrying these through to the problems, potential causes and root causes of the problem

» Quantify the operational performance of similar companies and establish internal targets based on best-in-class results

» Apply key performance measurements and continuous process improvement initiatives to improve process quality on a continual basis

» Provide results of the analyses to colleagues

» Control and check errors, taking corrective action so deviation from standards are minimised and the organisation’s goals are achieved

» Identify the use of appropriate technological developments to improve the system

	» Describe that continuous process improvement is an accepted way of organisational life<br>» Execute ways of eliminating unnecessary steps in system design<br>» Apply processes that strive to eliminate waste<br>» Apply processes to encourage sustainability (e.g., reducing carbon footprint, establishing a paperless office, using renewable energy)<br>» Describe the systematic approach used to close process or system performance gaps through streamlining and cycle time reduction, and identify and eliminate causes of quality below specifications, process variation and non-value-adding activities<br>» Outline the relationship between technology and process functionality<br>» Describe how policy, guidelines and regulatory reforms impact SC performance
5.4 Manage outsourcing of SCM functions	» Provide input on negotiating and compiling contracts with outside parties for delivery of logistics services
6.1 Demonstrate generic skills (e.g., literacy, numeracy, technology)	
6.1.1 Exhibit high understanding of literacy and numeracy	» Speak clearly and confidently, organize information in a logical manner, and consider voice tone and pace<br>» Respond to verbal messages and other cues (active listening)<br>» Express ideas and opinions clearly in written and verbal form<br>» Communicate information accurately, concisely and confidently in writing and verbally<br>» Practice a level of mathematics suitable to the job held<br>» Describe visual presentation techniques, including charting, histograms and flow sheets<br>» Read and comprehend at a level necessary to properly complete duties of the position<br>» Interpret written information in documents, such as reports, SOPs, LMIS forms, graphs, calendars, schedules, notices and directions<br>» Pay attention to detail and identify the main ideas, detect inconsistencies and identify missing information in documents<br>» Apply computer skills — such as using Word, Excel, PowerPoint and the internet — suitable to the job level<br>» Describe the process of analysing and validating KPIs<br>» Participate in the evaluation of activities that act as standards against which the KPIs are measured<br>» Apply the process of performing critical self-review on work before submission
6.2 Demonstrate strong communication skills	» Be truthful and trustworthy, and supply accurate information at all times<br>» Work as part of a workplace team

» Describe vocabulary and communication style and form for both written and verbal communication that is appropriate for the situation, audience and material being communicated (e.g., avoid unnecessary jargon, clearly explain medical and SCM terminology)

» Encourage open communication within constraints of confidentiality

» Organize communications about logistics

» Encourage participation in meetings, expressing one's opinions, being aware of others' needs, and being appropriately assertive when required

» Apply perspective into the point of view of others, understanding their needs and goals

» Encourage building trust, rapport and credibility with others

» Anticipate the communication needs and concerns of others and respond to them

» Avoid conflict between work and personal interests

» Encourage meaningful two-way communication

» Influence others by persuasively presenting thoughts and ideas

» Apply assertiveness skills to deal with unreasonable requests and/or refusals that would compromise practice or consumer care

» Maintain a positive, supportive and appreciative attitude

» Apply the ability to balance the organisation's needs and the customer's needs

» Use self-control by maintaining composure and keeping emotions in check, even in difficult situations; deal calmly and effectively with stressful situations

» Encourage the expressing of opinions and providing information in written and/or verbal form in a manner that does not elicit concern, anger or other adverse response

» Apply the use of a systematic process for following up that demonstrates written reports have been received and understood

» Describe the means by which responses to input to the work environment are monitored

» Seek practical ways to overcome barriers to communication

» Identify language representation (e.g., interpreters) at meetings when appropriate

» Tackle difficult situations and resolve disputes between staff

» Give both positive and negative feedback sensitively

» Maintain and improve communication

» Apply the process of giving and requesting feedback frequently

» Apply the ability to communicate effectively with shop stewards and union representatives

6.2.1 Practice cultural awareness

» Describe cultural awareness and sensitivity, treating all people with fairness, respect and dignity

» Describe anti-discriminatory practices in the organisation, including HR and disciplinary procedures

	» Establish cultural sensitivity, equality and fairness at all levels of the organisation<br>» Challenge discriminatory behaviour directly and sensitively<br>» Act in a non-discriminatory way toward individuals and groups<br>» Integrate cultural awareness in learning and development approaches<br>» Avoid stereotypical responses by examining one's own behaviour and bias<br>» Manage cultural diversity in teams and make the most of differences<br>» Describe strategies and/or resources for communicating effectively with people from different cultural backgrounds<br>» Elicit information relating to values, beliefs and cultural backgrounds of consumers that may influence the way professional services are provided
Basal Technology Competency(ies):	7.1 Data Science<br>7.5 Planning systems<br>7.6 ERP includes function of LMIS<br>7.12 Basic Office Skills
7.1 Data Science	» Describe the end to end MIS technology components that are inherent in advanced MIS systems and explore how these may be relevant in different supply chain contexts<br>» Identify emerging technological approaches to data visualization and analysis<br>» Identify how data science can be applied within supply chain<br>» Ability to interact, provide guidance for data (dashboard) consolidation in various levels and use the tech tools<br>» Support movement of data into electronic form at lowest/remote SC level<br>» Ability to work with relational database
7.5 Planning systems	» Describe blockchain and understand the building blocks for planning systems<br>» Describe planning systems as a value-add capability for relevant global health use cases
7.6 ERP includes function of LMIS	» Support a Master Data Management System<br>» Describe the critical components of an ERP system<br>» Describe the meaning of common document types found within an ERP/LMIS<br>» Describe the logic of data visibility within an ERP<br>» Describe the importance of data integrity, record management and information security

7.12 Basic Office Skills	» Have a good understanding of common presentation authoring packages<br>» Have a good understanding of common spreadsheet authoring packages<br>» Have a good understanding of common document authoring packages<br>» Have a good understanding of common email authoring packages<br>» Have a good understanding of common instant messaging packages<br>» Have a good understanding of common video conferencing authoring packages
Advanced Technology Competency(ies):	7.2 Blockchain<br>7.7 Automation<br>7.11 Cloud Computing
7.2 Blockchain	» Describe blockchain as a value-add capability for relevant global health use cases
7.7 Automation	» Describe automation as a value-add capability for relevant global health use cases
7.11 Cloud Computing	» Describe cloud computing as a value-add capability for relevant global health use cases

Key Performance Indicators:

- Evaluates and updates Risk Register, Risk Register up-to-date following Risk and Audit Committee meetings
- Number of breaches in compliance to business rules versus total number of transactions
- Audits or reviews audit reports for Cold Supply Chain, Cycle counts, Supplier Performance, Transportation Performance, Delivery Performance and flags items out of performance bands for further investigation. Number of investigations versus Number of deviations
- % number of transactions breaching legislation versus number of transactions in a period.
- Number of security and access breaches and reports thereon. Launches root cause analysis investigations and proposes corrective actions.
- Evaluates Risk Mitigation, Avoidance and Elimination efforts. Number of times risk occurs and Mitigation, Avoidance and or Elimination failed. Total Failed occurrences versus total Items on Risk Register

Training:	Qualifications Available:	Certifications Available:
» Risk and exception management	» (CILT) International Diploma in Logistics Transport	

» Supply Chain Performance Measurements<br>» Risk Assessment<br>» Risk Identification<br>» Risk Mitigation<br>» Risk Response Planning		

Lead - HR/ Workforce Development

Organisation: Public Health Organisation – Intermediate Level				
Competency Level:	Serial Number:	Primary Process:	Job Role:	Supervises:
Managerial	5-571-572-3	Enable – Human Resources	Lead - HR/ Workforce Development	Officer - Human Resources Specialist

Contributions	Implement a Human Resources platform to support the Human Resources Enablers specified in the Public Health System
Personal/Management Competency(ies):	5.5 Manage and plan projects<br>5.5.4 Direct/participate in teamwork<br>5.7 Oversee/support human resources (e.g., recruitment, training, team management/ supervision)<br>5.7.1 Manage staff recruitment process<br>5.7.2 Train staff<br>5.7.3 Supervise staff<br>5.7.4 Assessing HR systems<br>6.1 Demonstrate generic skills (e.g., literacy, numeracy, technology)<br>6.1.1 Exhibit high understanding of literacy and numeracy<br>6.2 Demonstrate strong communication skills<br>6.2.1 Practice cultural awareness

5.5 Manage and plan projects

5.5.4 Direct/participate in teamwork

- Describe the process of working with team members within their area of authority to establish achievable goals and strategies that are consistent with the objectives established for the organisation as a whole
- Describe an organisational chart that shows the lines of reporting and responsibility among staff
- Actively contribute a perspective and make a positive contribution to team-based problem solving and decision making
- Provide feedback, encouragement and support to team members for progressing strategic goals
- Define team performance in relation to the organisation's mission and goals
- Encourage teams to think for themselves and resolve problems
- Encourage and harness diversity within the team to boost team effectiveness
- Encourage and support the team to work through its stages of development and perform well
- Describe roles and responsibilities in relation to employees' expertise and the expectations of collaborating team members

	» Describe where their position fits in the structure (organisation, SC, healthcare sector) , the responsibilities their position entails and the accountabilities linked to the position
5.7 Oversee/support human resources (e.g., recruitment, training, team management/ supervision)	
5.7.1 Manage staff recruitment process	» Apply use of a system for staff recruitment, appraisals and monitoring<br>» Apply training and recruitment plans to fill any identified gaps and shortages<br>» Identify relevant and appropriate selection criteria for a defined role<br>» Describe and use the standard selection documentation for recruitment<br>» List key issues impacting the size and membership of an interview panel (e.g., logistics, gender balance, absence of conflict of interest)<br>» Describe requirements for a fair, defensible and balanced interview process<br>» Describe and use the documentation maintained for each interviewed position<br>» List due process for formalising an appointment that minimises the opportunity for untoward effects (e.g., appeal, conflict, resignation)<br>» Use job descriptions to obtain and retain skilled staff<br>» Describe the process of consulting with and involving relevant personnel in development and updating of role descriptions/duty statements that clarify the duties and responsibilities of positions<br>» Orient new staff to the workplace, explaining standard operating systems and procedures<br>» Describe human resource plans to meet the future staffing needs of the organisation<br>» Explain key features and measures used for monitoring a personnel retention strategy (e.g., recruitment costs, staff turnover, staff surveys)<br>» Describe the legislative issues impacting human resource policies and procedures<br>» Maintain employee master data including medicals, allergies to ensure safe working environment<br>» Apply an employee opinion survey
5.7.2 Train staff	» Maintain a list of staff that require logistics system training (based on supportive supervision reports)<br>» Describe the process of coordinating all training in managing the logistics system for all health facility staff<br>» Apply a culture of promoting participation in relevant learning and development opportunities<br>» Assist in training nurses and other health professionals in medicines ordering and storage procedures

» Orient new doctors to the formulary and the systems and procedures of the medicines supply system

» Provide on-the-job training to district health officials and hospital pharmacists as needed

» Ensure staff have the necessary skills and understanding for safe practice in the event they need to fill a management role due to absence or illness

» Ensure all staff handling hazardous materials receive training on safety & compliance regulations

» Identify any staff needs at all levels of the supply chain

» Discuss key factors important for initiating and/or sustaining the motivation of adult learners

» Discuss the variable learning needs and styles or modalities in adult learners (e.g., visual versus auditory)

» Describe a range of strategies that could be used to reinforce and clarify educational content (e.g., tutorials or workshops, written materials, intranet- or internet-based resources)

» Apply the use train-the-trainer to equipment trainers

» Apply training effectiveness monitoring and training feedback systems

5.7.3 Supervise staff

» Use a performance management process with all personnel to ensure continuous improvement

» Influence an environment that is supportive of learning and professional development

» Describe the nature of the supervisory role, what is meant by direct supervision, and where responsibility for outputs and outcomes rests

» Describe the process of planning and conducting supervisory tours of dependent facilities

» Identify situations where supervised personnel are experiencing difficulties in completing work activities and/or where a mandatory notification obligation exists

» Describe signs/cues from supervised personnel that indicate additional guidance or support is needed (e.g., hesitancy, distress, seeks clarification from less authoritative sources)

» Provide feedback, encouragement and support to team members

» Discuss, in a constructive manner, areas where performance should/could be improved and provide resources to support this

» Discuss expectations, achievements and contributions with personnel in a fair and equitable manner

» Apply the process of supporting colleagues in creating a professional development plan and suggesting ways the plan may be progressed through relevant training and/or experiential learning opportunities

» Encourage and support high-potential employees to accept growth challenges outside their own areas

» Provide progression opportunities for employees who have prepared themselves through development

» Identify and deal with unproductive staff using an approved process in an ethical manner

	» Identify and address work issues contributing to impairment of personnel (e.g., excessive workload, conflict)<br>» Explain the complaints management process<br>» Describe situations where additional expertise should be sought (e.g., counselling in the event of workplace violence or in situations of diminished performance due to drug or alcohol abuse)<br>» Acknowledge and respect different working styles
5.7.4 Assessing HR systems	» Provide input on assessing HR systems
6.1 Demonstrate generic skills (e.g., literacy, numeracy, technology)	
6.1.1 Exhibit high understanding of literacy and numeracy	» Speak clearly and confidently, organize information in a logical manner, and consider voice tone and pace<br>» Respond to verbal messages and other cues (active listening)<br>» Express ideas and opinions clearly in written and verbal form<br>» Communicate information accurately, concisely and confidently in writing and verbally<br>» Practice a level of mathematics suitable to the job held<br>» Describe visual presentation techniques, including charting, histograms and flow sheets<br>» Read and comprehend at a level necessary to properly complete duties of the position<br>» Interpret written information in documents, such as reports, SOPs, LMIS forms, graphs, calendars, schedules, notices and directions<br>» Pay attention to detail and identify the main ideas, detect inconsistencies and identify missing information in documents<br>» Apply computer skills — such as using Word, Excel, PowerPoint and the internet — suitable to the job level<br>» Describe the process of analysing and validating KPIs<br>» Participate in the evaluation of activities that act as standards against which the KPIs are measured<br>» Apply the process of performing critical self-review on work before submission
6.2 Demonstrate strong communication skills	» Be truthful and trustworthy, and supply accurate information at all times<br>» Work as part of a workplace team<br>» Describe vocabulary and communication style and form for both written and verbal communication that is appropriate for the situation, audience and material being communicated (e.g., avoid unnecessary jargon, clearly explain medical and SCM terminology)<br>» Encourage open communication within constraints of confidentiality<br>» Organize communications about logistics

	» Encourage participation in meetings, expressing one's opinions, being aware of others' needs, and being appropriately assertive when required<br>» Apply perspective into the point of view of others, understanding their needs and goals<br>» Encourage building trust, rapport and credibility with others<br>» Anticipate the communication needs and concerns of others and respond to them<br>» Avoid conflict between work and personal interests<br>» Encourage meaningful two-way communication<br>» Influence others by persuasively presenting thoughts and ideas<br>» Apply assertiveness skills to deal with unreasonable requests and/or refusals that would compromise practice or consumer care<br>» Maintain a positive, supportive and appreciative attitude<br>» Apply the ability to balance the organisation's needs and the customer's needs<br>» Use self-control by maintaining composure and keeping emotions in check, even in difficult situations; deal calmly and effectively with stressful situations<br>» Encourage the expressing of opinions and providing information in written and/or verbal form in a manner that does not elicit concern, anger or other adverse response<br>» Apply the use of a systematic process for following up that demonstrates written reports have been received and understood<br>» Describe the means by which responses to input to the work environment are monitored<br>» Seek practical ways to overcome barriers to communication<br>» Identify language representation (e.g., interpreters) at meetings when appropriate<br>» Tackle difficult situations and resolve disputes between staff<br>» Give both positive and negative feedback sensitively<br>» Maintain and improve communication<br>» Apply the process of giving and requesting feedback frequently<br>» Apply the ability to communicate effectively with shop stewards and union representatives
6.2.1 Practice cultural awareness	» Describe cultural awareness and sensitivity, treating all people with fairness, respect and dignity<br>» Describe anti-discriminatory practices in the organisation, including HR and disciplinary procedures<br>» Establish cultural sensitivity, equality and fairness at all levels of the organisation<br>» Challenge discriminatory behaviour directly and sensitively<br>» Act in a non-discriminatory way toward individuals and groups<br>» Integrate cultural awareness in learning and development approaches<br>» Avoid stereotypical responses by examining one's own behaviour and bias<br>» Manage cultural diversity in teams and make the most of differences

- Describe strategies and/or resources for communicating effectively with people from different cultural backgrounds
- Elicit information relating to values, beliefs and cultural backgrounds of consumers that may influence the way professional services are provided

Basal Technology Competency(ies):	7.12 Basic Office Skills<br>7.13 Have a command of technology

7.12 Basic Office Skills

- Have a good understanding of common presentation authoring packages
- Have a good understanding of common spreadsheet authoring packages
- Have a good understanding of common document authoring packages
- Have a good understanding of common email authoring packages
- Have a good understanding of common instant messaging packages
- Have a good understanding of common video conferencing authoring packages

7.13 Have a command of technology

- Apply the use of technology suitable to the job held
- Provide input on monitoring new developments and technologies in the sector
- Identify new technologies and recognising potential benefits for the sector
- Identify resources and support that are provided across the organisation to enable colleagues to make the best use of available technology
- Ensure that the organisation has a strategy for technology use
- Describe how to minimise environmental damage through technology use
- Use technology to maximise effectiveness and efficiency
- Use field-based technology (e.g., radio, general packet radio service (GPRS), satellite phone)

Advanced Technology Competency(ies):	7.11 Cloud Computing

7.11 Cloud Computing

- Describe cloud computing as a value-add capability for relevant global health use cases

Key Performance Indicators:

- Competency compliance. No of personnel evaluated and found to competent versus plan.
- Number of personnel with the required Skill, Education, Training and Aptitude as per Supply Chain Strategy versus Total number of personnel.
- Number of personnel progressed professionally versus Number of personnel that were targeted for progress.
- Planned skills upliftment plan - Number of personnel per competency versus actual skills upliftment Number of personnel per competency.
- HR4SCM Competency Requirements & Plan
- Creation of the HR4SCM Competency Requirements & Plan based on the Supply Chain Requirements
- Number of personnel with the required Skill, Education, Training and Aptitude as per Supply Chain Strategy versus Total number of personnel.
- Number of personnel progressed professionally versus Number of personnel that were targeted for progress.
- Planned skills upliftment plan - Number of personnel per competency versus actual skills upliftment Number of personnel per competency.
- Competency compliance. No of personnel evaluated and found to competent versus plan.
- HR4SCM Competency Requirements & Plan
- Direct Labour Cost
- Human Resource Contract Management
- EHS regulations
- Legislation and Standards
- Performance Management

Training:	Qualifications Available:	Certifications Available:
» Performance Management<br>» Contract Management<br>» EHS regulations<br>» Legislation and Standards<br>» Outsourcing	» Diploma in Human Resource Management<br>» Diploma in Management of Training<br>» Diploma: Human Resource Management	

Lead – Health SC System Strengthening

Organisation:

Public Health Organisation – Intermediate Level

Competency Level:	Serial Number:	Primary Process:	Job Role:	Supervises:
Managerial	3-31-32-3	Enable – Performance	Lead – Health SC System	-

Strengthening	
Contributions	Performs a support function to assist the process owners to get to the root-cause of performance deviations or process failures.
Technical Competency(ies):	3.1 Undertake storage, warehousing and inventory management<br>3.1.1 Manage storage of commodities during emergency<br>3.2 Supply commodities to facilities<br>3.3 Supply commodities to sections within a facility
3.1 Undertake storage, warehousing and inventory management	» Efficiently distribute products among suppliers, distribution centres, warehouses and customers through a logistics network<br>» Identify inputs to a total systems approach to designing and managing the entire flow of information, materials, and services<br>» Describe the choice of equipment or materials based on suitability for intended use, accuracy, safety of use and cost<br>» Maintain warehouses formal storage locations that identify the row, rack section, level and shelf location, typically with an alphanumeric location bar code or label<br>» Describe how medicines are appropriately stored, considering temperature, access and cleanliness<br>» Identify appropriate use of, and ability to maintain, the cold chain<br>» Describe the movement and storage of materials within a warehouse<br>» Describe how to secure the medical store and limit access to staff<br>» Describe organisation processes, instructions, rules and parameters for warehouse and inventory management and use of equipment (e.g., annual stock turns, expiry date tracking, stock procurement, rotation and retrieval, equipment maintenance)<br>» Describe policies and procedures for stock handling, distribution and withdrawal consistent with maintaining safety (e.g., policies for distribution of concentrated electrolyte solutions and cytotoxics, identification of cytotoxic drug products within the work environment, separation of like-named or like-packaged products)<br>» Identify requirements of information on the supplies being stored<br>» Describe the process of consolidating several items into larger units for fewer handlings<br>» Describe methods of stock rotation (e.g., first in first out, FIFO, or first to expire first out, FEFO)<br>» Describe the process of entering the quantity received, and updating the warehouse management system software with the stock on hand when each order is delivered<br>» Asses required levels of stock and/or equipment in specific situations<br>» Ensure accurate verification of rolling stocks<br>» Monitor the location and condition of the supplies being stored<br>» Collate and prepare information on the storage locations and facilities<br>» Identify any problems in using the storage locations and facilities
3.1.1 Manage storage of	» Describe the types of warehouses available for emergency or disaster supply

commodities during emergency	» Describe the importance of the choice of storage site for emergency or disaster supply<br>» Describe the staff required for the storage of emergency or disaster supply<br>» Describe the equipment and material required for the warehousing of emergency or disaster supply<br>» Provide input to a rapid assessment of logistical needs in emergencies
3.2 Supply commodities to facilities	» Describe the various sources of distribution demand (customers)<br>» Describe the use of order policies for planned order generation<br>» Describe the calculation of re-supply quantities using a variety of inventory methods, including visual review, two bin, periodic review, order point and just in time<br>» Prepare and use order schedules<br>» Calculate safety stock<br>» Use economic order quantity (EOQ) principles in ordering<br>» Apply the use of pull and push inventory control systems<br>» Apply a regular, rather than urgent, order culture<br>» Apply order-filling priorities in relation to delivery opportunities and urgency<br>» Screen orders (modify order quantities on the basis of available stock, impact on service delivery, distance of facility from hospital)<br>» Describe assembling, checking, picking and packing of orders<br>» Describe making and using dispatch lists and invoices<br>» Describe the balancing of supply with demand, considering both lead time and demand variability created by supply patterns not matching demand patterns<br>» Describe information on the storage locations and facilities<br>» Apply measures of customer satisfaction and identify loyal customers by using performance metrics taken from the customer perspective, with criteria such as on-time delivery, perception of quality, complaints and length of wait times
3.3 Supply commodities to sections within a facility	» Review imprest quantities regularly and assist in the development of the list in conjunction with doctors and nurses<br>» Validate the requisition from different health care services within the facility

Personal/Management Competency(ies):	5.1 Design and implement supply chain system and strategies<br>5.3 Implement risk management and monitoring and evaluation activities for the supply chain<br>5.3.1 Ensure monitoring and evaluation activities are completed<br>5.3.2 Implement risk management activities<br>5.5 Manage and plan projects<br>5.5.2 Execute strategic decision making<br>5.5.3 Manage partnerships<br>6.2 Demonstrate strong communication skills<br>6.2.1 Practice cultural awareness<br>6.3 Utilize problem-solving skills

	6.3.1 Negotiate<br>6.5 Prove leadership abilities<br>6.5.1 Demonstrate resilience and ability to manage stress<br>6.6 Abide by rules / laws / legislation
5.1 Design and implement supply chain system and strategies	» Apply a responsive, agile and efficient supply chain that has the ability to meet the changing needs of customers and deliver high-quality products with short lead times at low cost<br>» Describe the interrelationship of organisations, people, technology, activities, information, and resources involved in moving a product from supplier to customer<br>» List the management activities carried out in the course of running an organisation, including controlling, leading, monitoring, adjusting, organising and planning<br>» Describe the characteristics unique to public health, demonstrate awareness of the factors that could contribute to or hinder the delivery of logistics services<br>» Describe emerging concepts and principles in public health logistics; adapt current practices to incorporate new developments in the field<br>» Describe which type of inventory control system will be most effective for a particular programme or country<br>» Explain the difference between continuous and period review systems of inventory control<br>» Define and apply a formal logistics strategy and plan
5.3 Implement risk management and monitoring and evaluation activities for the supply chain	
5.3.1 Ensure monitoring and evaluation activities are completed	» Describe the periodic evaluations to maintain processes by gathering pertinent information — such as problem symptoms from knowledgeable sources, carrying these through to the problems, potential causes and root causes of the problem<br>» Quantify the operational performance of similar companies and establish internal targets based on best-in-class results<br>» Apply key performance measurements and continuous process improvement initiatives to improve process quality on a continual basis<br>» Provide results of the analyses to colleagues<br>» Control and check errors, taking corrective action so deviation from standards are minimised and the organisation’s goals are achieved<br>» Identify the use of appropriate technological developments to improve the system<br>» Describe that continuous process improvement is an accepted way of organisational life<br>» Execute ways of eliminating unnecessary steps in system design<br>» Apply processes that strive to eliminate waste<br>» Apply processes to encourage sustainability (e.g., reducing carbon footprint, establishing a paperless office, using renewable energy)

	» Describe the systematic approach used to close process or system performance gaps through streamlining and cycle time reduction, and identify and eliminate causes of quality below specifications, process variation and non-value-adding activities<br>» Outline the relationship between technology and process functionality<br>» Describe how policy, guidelines and regulatory reforms impact SC performance
5.3.2 Implement risk management activities	» Identify risks affecting supply, transformation, delivery and customer demand<br>» Identify unsafe working conditions and take prompt, corrective action; stay alert to and take preventive action against hazards and threats; recommend measures to protect employees from hazardous working conditions; follow protocol for reporting safety violations; and investigate accidents to determine how to prevent them in the future<br>» Apply training programmes/drills in which personnel participate (e.g., fire and armed hold-up drills, safe manual handling practices, use of mandatory safety clothing)<br>» Apply systems of review (e.g., audit) to check compliance with policies and procedures intended to maintain workplace safety (e.g., manual handling techniques, correct use of protective clothing)<br>» Apply the process of ensuring equipment and tools are operating to prescribed standards<br>» Apply key security systems for the workplace (e.g., for cash, narcotics and other controlled substances, investigational drugs, consumer records, entry and exit points) and levels of access and/or authority applicable to each<br>» Describe the prevention strategies adopted (e.g., protocols, security barriers, fixed or personal duress alarms)<br>» Document critical incidents<br>» Provide input when engaging in contingency planning and managing operational continuity<br>» Increase understanding of a safe working environment and engender a shared commitment from all personnel to creating one<br>» Describe relevant safety and health laws and regulations; comply with safety codes, standards and guidelines; adhere to worksite safety programmes; understand emergency response plans when they exist<br>» Describe how to handle hazardous commodities and first aid procedures
5.5 Manage and plan projects	
5.5.2 Execute strategic decision making	» Make decisions regarding one's own workload and area of responsibility<br>» Prioritize competing tasks and perform them quickly and efficiently according to their importance; find new ways of organizing or planning work to accomplish it more efficiently<br>» Ensure proper time and space for consultation around decisions being made.<br>» Consider the input of resources including staff needed<br>» Check assumptions against facts<br>» Identify the key issues in a complex situation and come to the heart of the problem quickly<br>» Gather relevant information before making decisions

» Describe the process of providing input for making strategic decisions in the interest of the organisation's goals
» Describe when to analyse, evaluate and execute tough decisions when necessary
» Consider the impact of decisions on others' work or team goals
» Describe the process to communicate decisions and ensure they are incorporated into policies and processes
» Describe inclusive and consultative strategic planning to establish strategic objectives
» Identify and support emerging trends and practices
» Describe the macro and long-term consequences of decisions

5.5.3 Manage partnerships

» Apply the concepts of partnership working
» Support implementation of partnership programmes
» Describe the process of experimenting with and trialling new ideas with partners
» Identify partnerships to deliver programme and increase impact
» Use innovation and creativity in partnership work
» Describe the process of communicating key information with partner members
» Describe the process of involving and valuing partners in all aspects of programming
» Identify problems with supply chain relationships
» Communicate effectively with nurses, doctors and other members of the healthcare team
» Respond to consumer complaints or comments about services and/or advice received
» Foster collaboration and consultation across the sector and encourage the team to work with others to gain market insight
» Assist in sharing input from other key actors in the humanitarian sector and develop relationships with a cross-section of actors
» Meet the reporting requirements of vertical programmes
» Outline the structure of the health system at a national level
» Discuss the role of other members of the healthcare team (including with consumers) in a way that engenders understanding and confidence in the team and its members
» Describe the structure of the organisation, environment and/or service in which they work
» Describe how vertical programmes work within the health system
» Describe the communication network established to achieve work outcomes
» Describe the structure of the health system at the provincial/regional level and explain this to others
» List the roles and functions of government agencies in regulating and supporting supply chain organisations
» Describe knowledge of change management (e.g. PtD Theory of Change)

6.2 Demonstrate strong communication skills	» Be truthful and trustworthy, and supply accurate information at all times<br>» Work as part of a workplace team<br>» Describe vocabulary and communication style and form for both written and verbal communication that is appropriate for the situation, audience and material being communicated (e.g., avoid unnecessary jargon, clearly explain medical and SCM terminology)<br>» Encourage open communication within constraints of confidentiality<br>» Organize communications about logistics<br>» Encourage participation in meetings, expressing one's opinions, being aware of others' needs, and being appropriately assertive when required<br>» Apply perspective into the point of view of others, understanding their needs and goals<br>» Encourage building trust, rapport and credibility with others<br>» Anticipate the communication needs and concerns of others and respond to them<br>» Avoid conflict between work and personal interests<br>» Encourage meaningful two-way communication<br>» Influence others by persuasively presenting thoughts and ideas<br>» Apply assertiveness skills to deal with unreasonable requests and/or refusals that would compromise practice or consumer care<br>» Maintain a positive, supportive and appreciative attitude<br>» Apply the ability to balance the organisation's needs and the customer's needs<br>» Use self-control by maintaining composure and keeping emotions in check, even in difficult situations; deal calmly and effectively with stressful situations<br>» Encourage the expressing of opinions and providing information in written and/or verbal form in a manner that does not elicit concern, anger or other adverse response<br>» Apply the use of a systematic process for following up that demonstrates written reports have been received and understood<br>» Describe the means by which responses to input to the work environment are monitored<br>» Seek practical ways to overcome barriers to communication<br>» Identify language representation (e.g., interpreters) at meetings when appropriate<br>» Tackle difficult situations and resolve disputes between staff<br>» Give both positive and negative feedback sensitively<br>» Maintain and improve communication<br>» Apply the process of giving and requesting feedback frequently<br>» Apply the ability to communicate effectively with shop stewards and union representatives
6.2.1 Practice cultural awareness	» Describe cultural awareness and sensitivity, treating all people with fairness, respect and dignity<br>» Describe anti-discriminatory practices in the organisation, including HR and disciplinary procedures<br>» Establish cultural sensitivity, equality and fairness at all levels of the organisation

- Challenge discriminatory behaviour directly and sensitively
- Act in a non-discriminatory way toward individuals and groups
- Integrate cultural awareness in learning and development approaches
- Avoid stereotypical responses by examining one's own behaviour and bias
- Manage cultural diversity in teams and make the most of differences
- Describe strategies and/or resources for communicating effectively with people from different cultural backgrounds
- Elicit information relating to values, beliefs and cultural backgrounds of consumers that may influence the way professional services are provided

6.3 Utilize problem-solving skills

- Identify information to solve problems in a sensitive and ethical manner
- Manage day to day and complex problems in a timely manner
- Explain problem situations and their step-by-step transformation based on planning and reasoning, without apportioning blame
- Critically review, analyse, synthesize, compare and interpret information; draw conclusions from relevant and/or missing information; and understand the relationship among facts and apply this understanding when solving problems
- Describe a range of possible approaches/strategies that are effective for resolving conflict in the workplace (e.g., negotiation, collaborative problem-solving, mediation, arbitration)
- Use an open environment that encourages people to work together
- Resolve conflicts as they arise
- Use creativity through questioning, attempting to improve on ideas, applying other experiences and working toward action in problem solving
- Break down facts and thoughts into strengths and weaknesses
- Think in a careful way to solve problems, analyse data, and recall and apply information, involving others
- Practice goal-directed thinking and action in situations in which no routine solutions exist
- Maintain appropriate ethical and moral standards in resolving problems
- Choose between alternative courses of action, using cognitive processes such as memory, thinking and evaluation
- Map likely consequences of decisions to choose the best course of action
- Ask other people to help with solving problems
- Use difficult or unusual situations to develop unique approaches and useful solutions
- Commit to a solution in a timely manner, and develop a realistic approach for applying the chosen solution; evaluate the outcome of the solution to see if further action is needed, and identify lessons learned
- Describe the impact of conflict in the workplace (e.g., tension, low morale, absenteeism, system or service failure, aggressive or uncooperative behaviours)
- Follow up on problems to ensure they are fixed
- Describe situations where referral is warranted (e.g., severe emotional distress, intractable dispute)

6.3.1 Negotiate	» Apply the principles of negotiation and aim for a win-win outcome<br>» Manage and resolve relationship issues within and outside the team<br>» Model solution-focussed approaches to further the organisation's mission<br>» Build consensus among parties<br>» Present or propose alternative ways of doing things<br>» Identify where fair approaches are being utilised to resolve issues<br>» Seek to reach constructive solutions while maintaining positive working relationships<br>» Adapt style to take into account cultural differences regarding negotiation<br>» Build consensus at a high level for the benefit of all parties
6.5 Prove leadership abilities	» Provide input to meetings and programme development<br>» Identify future trends and issues, and assist the organisation in meeting the challenges<br>» Contribute to a collaborative working environment<br>» Describe how change and improvement develops opportunities for the organisation and sector<br>» Channel energy and ideas toward resolving issues<br>» Communicate with influence<br>» Value the practise of showing courage to take an unpopular stance when needed<br>» Steer and implement change organisationally<br>» Anticipate and resolve conflict<br>» Show initiative in working methods<br>» Exemplify personal drive and integrity<br>» Serve as a role model for others to follow<br>» Demonstrate leadership and practice management skills, initiative and efficiency<br>» Inspire others through professional excellence, innovation and communication<br>» Discuss situations in which a change in leadership style would be warranted (e.g., directive versus consultative style in the event of a crisis)
6.5.1 Demonstrate resilience and ability to manage stress	» Identify symptoms of stress and take steps to reduce stress<br>» See the bigger picture and help others to<br>» Recover quickly from setbacks<br>» Draw on previous experience and support mechanisms to reduce the impact of stress on self and others<br>» Describe a working environment that aims to minimise pressure and stress<br>» Cope well under pressure, particularly in difficult environments<br>» Act as a role model for others and display courage under difficult circumstances<br>» Recognise the limitations of staff and take action to limit their exposure to harm when needed

- Identify and make use of personal support mechanisms
- Help others identify personal support mechanisms
- Influence organisational policy to support self-care in agencies

6.6 Abide by rules / laws / legislation

- List and comply with the legislation that covers the practice of public health supply chains and healthcare, and describe its purpose
- Describe requirements of professional codes, guidelines and standards adopted as part of the legislative framework
- Keep up to date with changes in legal instruments, as informed by national-level managers
- Comply with international regulations in decision making for the distribution system, including customs regulations on import and export requirements (e.g., trade tariffs and duties on imported goods) and security regulations (e.g., 2007 SAFE Ports Act, NAFTA or European Union trade agreements)
- Apply and understand regulatory affairs and the key aspects of pharmaceutical registration and legislation
- Apply knowledge of the principles of business economics and intellectual property rights, including the basics of patent interpretation
- Apply knowledge of applicable procurement law and the practical effects of a contract's terms and conditions
- Describe current industry and government regulations governing sustainability
- Apply a working knowledge of key hazardous materials handling laws
- Apply policies and procedures relating to workplace safety that are consistent with agreed or recognised standards
- Describe professional standards and conventions, as well as workplace policies and procedures, for preparing pharmaceutical products
- Apply all standard operating procedures
- Comply with data privacy and data protection laws

Basal Technology Competency(ies):	7.4 Temperature and monitoring<br>7.6 ERP includes function of LMIS<br>7.12 Basic Office Skills<br>7.13 Have a command of technology

7.4 Temperature and monitoring

- Describe the current technology approaches for Temperature monitoring and sensors
- Describe what is required to implement this technology in a country context
- Describe infrastructure requirement for heat-sensitive items at peripheral 'lower level' of SC depending on volume and nature of items to handle;
- Define regular preventive maintenance and calibration of cold-chain equipment with biomedical engineers or logisticians

	» Describe the policy for temperature monitoring tools during transportation and storage<br>» Describe the steps involved in health logistic management cycle: selection-order-delivery-reception-storage-distribution-inventory<br>» Describe hardware qualification and system validation of temperature monitoring<br>» Consider mass data management of temperature monitoring
7.6 ERP includes function of LMIS	» Support a Master Data Management System<br>» Describe the critical components of an ERP system<br>» Describe the meaning of common document types found within an ERP/LMIS<br>» Describe the logic of data visibility within an ERP<br>» Describe the importance of data integrity, record management and information security
7.12 Basic Office Skills	» Have a good understanding of common presentation authoring packages<br>» Have a good understanding of common spreadsheet authoring packages<br>» Have a good understanding of common document authoring packages<br>» Have a good understanding of common email authoring packages<br>» Have a good understanding of common instant messaging packages<br>» Have a good understanding of common video conferencing authoring packages
7.13 Have a command of technology	» Apply the use of technology suitable to the job held<br>» Provide input on monitoring new developments and technologies in the sector<br>» Identify new technologies and recognising potential benefits for the sector<br>» Identify resources and support that are provided across the organisation to enable colleagues to make the best use of available technology<br>» Ensure that the organisation has a strategy for technology use<br>» Describe how to minimise environmental damage through technology use<br>» Use technology to maximise effectiveness and efficiency<br>» Use field-based technology (e.g., radio, general packet radio service (GPRS), satellite phone)
Advanced Technology Competency(ies):	7.2 Blockchain<br>7.11 Cloud Computing
7.2 Blockchain	» Describe blockchain as a value-add capability for relevant global health use cases
7.11 Cloud Computing	» Describe cloud computing as a value-add capability for relevant global health use cases

Key Performance Indicators:

- Risk / Mitigation Costs
- Time to Recovery (TTR)
- Launch Projects to Evaluate adequacy of Supply Chain practices and Implement best practices to enable Supply Chain reliability and agility. Cause a benefit analysis to be conducted to validate assumed benefits.
- Engage in root cause analysis to eradicate or minimize process errors and suggest process, practices or education and training interventions. Audit results and report.
- Maintain a project portfolio for all projects envisaged targeted at improving Supply Chain Performance. Track progress and benefits derived from the implementations

Training:	Qualifications Available:	Certifications Available:
» SC Optimization<br>» Scenario Planning<br>» Integrated Business/ SC Planning<br>» Supply chain Risk<br>» Executive training<br>» Advanced Supply Chain Management<br>» System Improvement<br>» Process Improvement	» Diploma in International Trade Management in Exports/Imports<br>» (CILT) International Diploma in Logistics & Transport	» Higher Certificate in Export Management<br>» SCPro™ Certification<br>» Lean Six Sigma Certification in Warehousing green belt<br>» Lean Six Sigma Certification in Warehousing yellow belt

Operational

Officer – Demand and Supply Planning

Organisation:

Public Health Organisation – Intermediate Level

Competency Level:	Serial Number:	Primary Process:	Job Role:	Supervises:
Operational	1-11-12-2	Plan – Procurement	Officer – Demand and Supply Planning	-

Contributions	Executes the Procurement process as prescribed in the Public Health System Organisation Operating Model as it relates to the context.
Technical Competency(ies):	1.1 Select the appropriate product<br>1.2 Define the specifications of the product, including product quality<br>1.3 List any special considerations for the product (e.g., temperature requirements, size, implications for infrastructure)<br>1.4 Forecast and quantify product needs<br>1.5 Supply planning
1.1 Select the appropriate product	» Compare the broad concepts of the national medicines policy, essential medicines lists, essential equipment lists, standard treatment guidelines and "dangerous drug" (DDA) policy<br>» Understand the government system required to add and subtract items from the essential medicines list and the essential equipment list<br>» Understand that the government system required to alter standard treatment guidelines, dangerous drug policy and national medicines policy<br>» Compare the type of supplies and services that are required<br>» Provide input for regularly scheduled coordination meetings with stakeholders involved in financing, procuring or distributing commodities<br>» Understand the steps needed to bring a medicinal product to the market, including the safety, quality, efficacy and pharmacoeconomic assessments of the product
1.2 Define the specifications of the product, including product quality	» Understand specifications for procurement<br>» Compare the characteristics of a good specification<br>» Compare the types of specifications<br>» Name the advantages of functional and performance specifications<br>» Examine the contents of a specification<br>» Name the procurement staff responsibilities in the specification process<br>» Compare the quality standard of product<br>» Not Applicable

1.3 List any special considerations for the product (e.g., temperature requirements, size, implications for infrastructure)	» Compare the principles and processes of category management, including market segmentation principles<br>» Examine any specific considerations in the quantification of programme-specific products (e.g., ARVs, family planning commodities, vaccines)<br>» Examine current international trends in commodity availability<br>» Compare regulatory requirements for the product
1.4 Forecast and quantify product needs	» Examine the factors that affect usage patterns of medications and equipment and how this affects ordering (e.g., disease outbreaks), using national policies as a guide and to ensure consistent application<br>» Understand principles and applications of demand forecasting<br>» Compare the critical requirements for effective forecasting: establishing time horizons, level of detail and use of data<br>» Understand policies and procedures for forecasting<br>» Understand the process of quantifying product requirements using a variety of methods<br>» Examine consumption data at the national level for various country programmes<br>» Compare and reconcile different types of forecasts<br>» Understand the process of applying VEN or ABC analysis to programme requirements for national level procurement<br>» Understand the importance of calculating average monthly dispensed-to-user quantities for all service delivery points nationally<br>» Understand the importance of calculating storage space requirements for all levels in the supply chain<br>» Understand the importance of calculating the months of supply on hand for each commodity at the national level<br>» Examine the maximum and minimum stock levels for each level in a programme<br>» Understand various tools used in quantification (e.g., quantification software)<br>» Understand the use of various data sources for commodity forecasting (e.g., consumption data, services data, demographic data)<br>» Compare key performance indicators of forecast accuracy<br>» Examine the degree of error when using forecasting methods
1.5 Supply planning	» Examine a purchasing and supply plan in line with national priorities<br>» Understand the importance of balancing of supply chain resources and trigger activities to correct any imbalances<br>» Understand the formalisation of sales and operations plans through sales and operations meeting.<br>» Understand that the sales and operations plan must be translated into executable plans to ensure dispatch of goods in line with overall priorities.<br>» Understand the national commodity pipeline

Personal/Management Competency(ies):	6.1 Demonstrate generic skills (e.g., literacy, numeracy, technology)<br>6.1.1 Exhibit high understanding of literacy and numeracy

6.1 Demonstrate generic skills (e.g., literacy, numeracy, technology)

6.1.1 Exhibit high understanding of literacy and numeracy

» Speak clearly and confidently, organize information in a logical manner, and consider voice tone and pace

» Respond to verbal messages and other cues (active listening)

» Express ideas and opinions clearly in written and verbal form

» Communicate information accurately, concisely and confidently in writing and verbally

» Practice a level of mathematics suitable to the job held

» Compare visual presentation techniques, including charting, histograms and flow sheets

» Read and comprehend at a level necessary to properly complete duties of the position

» Understand the need to interpret written information in documents, such as reports, SOPs, LMIS forms, graphs, calendars, schedules, notices and directions

» Understand the need to pay attention to detail and identify the main ideas, detect inconsistencies and identify missing information in documents

» Enable computer skills — such as using Word, Excel, PowerPoint and the internet — suitable to the job level

» Understand the process of analysing and validating KPIs

» Understand how the evaluation process of activities that act as standards against which the KPIs are measured is carried out

» Examine the process of performing critical self-review on work before submission

Basal Technology Competency(ies):	7.6 ERP includes function of LMIS<br>7.12 Basic Office Skills<br>7.13 Have a command of technology

7.6 ERP includes function of LMIS

» Enable a Master Data Management System

» Understand the critical components of an ERP system

» Understand the meaning of common document types found within an ERP/LMIS

» Understand the logic of data visibility within an ERP

» Understand the importance of data integrity, record management and information security

7.12 Basic Office Skills

» Have a good understanding of common presentation authoring packages

	» Have a good understanding of common spreadsheet authoring packages<br>» Have a good understanding of common document authoring packages<br>» Have a good understanding of common email authoring packages<br>» Have a good understanding of common instant messaging packages<br>» Have a good understanding of common video conferencing authoring packages
7.13 Have a command of technology	» Enable the use of technology suitable to the job held<br>» Understand the need of monitoring new developments and technologies in the sector<br>» Compare new technologies and recognising potential benefits for the sector<br>» Understand that resources and support are provided across the organisation to enable colleagues to make the best use of available technology<br>» Understand that the organisation has a strategy for technology use<br>» Compare how to minimise environmental damage through technology use<br>» Enable the use of technology to maximise effectiveness and efficiency<br>» Enable the use of field-based technology (e.g., radio, general packet radio service (GPRS), satellite phone)
Advanced Technology Competency(ies):	7.7 Automation
7.7 Automation	» Understand automation as a value-add capability for relevant global health use cases

Key Performance Indicators:

- Vendor on time delivery (VOTD)
- % of emergency orders issued in the last 12 months
- % of product selection based on National Essential Medicines List
- % of procured products that meet Stringent Regulatory Authority (SRA) or WHO standards
- Reports submitted on time (for all and separate reports) in accordance with business rules (reporting calendar)
- Supply adequacy - Net requirements are reflected in the Procurement pipeline to secure sufficient future supply.
- Supply Plan report; supplier performance report
- Stock out rate - Number of missed line item quantity deliveries due to stock out versus total line item quantity deliveries
- Stock levels - Current on hand stock versus Planned on hand stock
- Forecasting and Supply Planning done within the specified cycle time to support the Supply Chain performance requirements.
- Data accuracy - Vendor Master data accuracy, item / SKU Master Data accuracy

- Establish Product Demand and Resources Cycle Time and recorded.in the "ERP" system against the products and resources.
- Establish Supply Plan Cycle Times and recorded in the "ERP" system.
- Fill Rate vs Planned fill rate
- Forecast accuracy, measured and reported on.
- Identify, Prioritize, and Aggregate Supply Chain Requirements Cycle Time - Actual Time taken to determine net supply chain requirements by product and SKU vs Planned Time
- Inventory Days of Supply - Actual inventory - Planned Demand over the planning horizon divided by the average daily usage
- Material and Resource Plan valid. Number of valid actions performed versus number of actions performed. (Exception messages trigger due to invalid schedules)
- Order accuracy - Net requirement versus order quantities in the planning horizon
- Procurement planning Cycle Time, by product and resource. Actual Overall planning cycle versus required Overall planning cycle time to achieve Supply Chain performance requirements.
- Ratio between median price of pro- ducts procured and the international median reference value
- Supply Chain Planning Cost recorded and measured against required Supply China Planning Cycle times to accomplish Supply Chain Strategic objectives.

Training:	Qualifications Available:	Certifications Available:
» Risk and exception management<br>» Import & export regulations for products<br>» Inventory management, Min-Max Replenishment<br>» ABC/VEN Inventory Classification		» CIPS Level 3 Advanced Certificate in Procurement and Supply Operations

Officer– Procurement

Organisation:

Public Health Organisation – Intermediate Level

Competency Level:	Serial Number:	Primary Process:	Job Role:	Supervises:
Operational	2-21-22-2	Enable – Procurement	Officer– Procurement	Officer – Procurement and Supply

Contributions	Maintains the procurement process, to ensure that materials and resources are available when needed and within specifications stipulate in the Procurement business rules.
Technical Competency(ies):	2.1 Manage procurement costs and budget<br>2.2 Build and maintain supplier relationships

2.1 Manage procurement costs and budget

- Understand public procurement regulations
- Understand the importance of budget approval before initiating procurement
- Understanding the importance of financial and management accounting practices within procurement
- Understand the commercial factors that contribute toward cost reduction, price savings and value improvement opportunities
- Understand the principles of foreign exchange rates and how they impact prices

2.2 Build and maintain supplier relationships

- Understand the factors in the analysis of the market
- Understand the processes for prequalification and tender contracting
- Understand the factors that define a good supplier
- Understand that key suppliers must be located and that there is a total cost associated with procuring an item or service
- Understand supplier relationship management plans (e.g., sourcing, frequency of meetings, negotiate and monitor benchmarks for performance)
- Understand the process of educating suppliers to create value for customers by streamlining processes in the value chain
- Understand the difference between strategic and non-strategic suppliers and the associated supplier management principles
- Understand commercial factors that contribute toward cost reduction, price savings and value improvement opportunities
- Understand the process of strategic sourcing practices drive down cost across consecutive tenders
- Compare the process of working with suppliers to agree cost saving opportunities in the whole value chain, with an appropriate cost sharing mechanism applied to ensure a “win-win” outcome
- Understand the process of managing supplier risk
- Understand ethical sourcing

Personal/Management Competency(ies):	6.1 Demonstrate generic skills (e.g., literacy, numeracy, technology)<br>6.1.1 Exhibit high understanding of literacy and numeracy<br>6.2 Demonstrate strong communication skills<br>6.2.1 Practice cultural awareness

6.1 Demonstrate generic skills (e.g., literacy, numeracy, technology)

6.1.1 Exhibit high understanding of literacy and numeracy

» Speak clearly and confidently, organize information in a logical manner, and consider voice tone and pace

» Respond to verbal messages and other cues (active listening)

» Express ideas and opinions clearly in written and verbal form

» Communicate information accurately, concisely and confidently in writing and verbally

» Practice a level of mathematics suitable to the job held

» Compare visual presentation techniques, including charting, histograms and flow sheets

» Read and comprehend at a level necessary to properly complete duties of the position

» Understand the need to interpret written information in documents, such as reports, SOPs, LMIS forms, graphs, calendars, schedules, notices and directions

» Understand the need to pay attention to detail and identify the main ideas, detect inconsistencies and identify missing information in documents

» Enable computer skills — such as using Word, Excel, PowerPoint and the internet — suitable to the job level

» Understand the process of analysing and validating KPIs

» Understand how the evaluation process of activities that act as standards against which the KPIs are measured is carried out

» Examine the process of performing critical self-review on work before submission

6.2 Demonstrate strong communication skills

» Be truthful and trustworthy, and supply accurate information at all times

» Enable working as part of a workplace team

» Understand vocabulary and communication style and form for both written and verbal communication that is appropriate for the situation, audience and material being communicated (e.g., avoid unnecessary jargon, clearly explain medical and SCM terminology)

» Enable open communication within constraints of confidentiality

» Participate in the organisation of communications about logistics

» Enable participation in meetings, expressing one's opinions, being aware of others' needs, and being appropriately assertive when required

» Show perspective into the point of view of others, understanding their needs and goals

» Enable the practise of building trust, rapport and credibility with others
» Understand the communication needs and concerns of others and respond to them
» Understand the need to avoid conflict between work and personal interests
» Enable meaningful two-way communication
» Show influence on others by persuasively presenting thoughts and ideas
» Show assertiveness skills to deal with unreasonable requests and/or refusals that would compromise practice or consumer care
» Maintain a positive, supportive and appreciative attitude
» Show the ability to balance the organisation's needs and the customer's needs
» Show self-control by maintaining composure and keeping emotions in check, even in difficult situations; deal calmly and effectively with stressful situations
» Enable expressing of opinions and providing information in written and/or verbal form in a manner that does not elicit concern, anger or other adverse response
» Enable the use of a systematic process for following up that demonstrates written reports have been received and understood
» Understand the means by which responses to input to the work environment are monitored
» Seek practical ways to overcome barriers to communication
» Understand the need to ensuring language representation (e.g., interpreters) at meetings when appropriate
» Interpret difficult situations and disputes between staff that need to be tackled and resolved
» Enable giving both positive and negative feedback sensitively
» Understand the need to maintain and improve communication
» Examine the process of giving and requesting feedback frequently
» Examine effective communication with shop stewards and union representatives

6.2.1 Practice cultural awareness

» Understand the need for cultural awareness and sensitivity, treating all people with fairness, respect and dignity
» Enable anti-discriminatory practices in the organisation, including HR and disciplinary procedures
» Enable cultural sensitivity, equality and fairness at all levels of the organisation
» Challenge discriminatory behaviour directly and sensitively
» Act in a non-discriminatory way toward individuals and groups
» Enable integrating cultural awareness in learning and development approaches
» Avoid stereotypical responses by examining one's own behaviour and bias
» Enable managing cultural diversity in teams and make the most of differences

	» Compare strategies and/or resources for communicating effectively with people from different cultural backgrounds<br>» Enable the process to elicit information relating to values, beliefs and cultural backgrounds of consumers that may influence the way professional services are provided
Basal Technology Competency(ies):	7.4 Temperature and monitoring<br>7.6 ERP includes function of LMIS<br>7.12 Basic Office Skills<br>7.13 Have a command of technology
7.4 Temperature and monitoring	» Compare the current technology approaches for Temperature monitoring and sensors<br>» Consider what is required to implement this technology in a country context<br>» Compare infrastructure requirement for heat-sensitive items at peripheral 'lower level' of SC depending on volume and nature of items to handle;<br>» Examine regular preventive maintenance and calibration of cold-chain equipment with biomedical engineers or logisticians<br>» Understand the policy for temperature monitoring tools during transportation and storage<br>» Understand the steps involved in health logistic management cycle: selection-order-delivery-reception-storage-distribution-inventory<br>» Understand hardware qualification and system validation of temperature monitoring<br>» Understand mass data management of temperature monitoring
7.6 ERP includes function of LMIS	» Enable a Master Data Management System<br>» Understand the critical components of an ERP system<br>» Understand the meaning of common document types found within an ERP/LMIS<br>» Understand the logic of data visibility within an ERP<br>» Understand the importance of data integrity, record management and information security
7.12 Basic Office Skills	» Have a good understanding of common presentation authoring packages<br>» Have a good understanding of common spreadsheet authoring packages<br>» Have a good understanding of common document authoring packages<br>» Have a good understanding of common email authoring packages<br>» Have a good understanding of common instant messaging packages<br>» Have a good understanding of common video conferencing authoring packages

7.13 Have a command of technology	» Enable the use of technology suitable to the job held<br>» Understand the need of monitoring new developments and technologies in the sector<br>» Compare new technologies and recognising potential benefits for the sector<br>» Understand that resources and support are provided across the organisation to enable colleagues to make the best use of available technology<br>» Understand that the organisation has a strategy for technology use<br>» Compare how to minimise environmental damage through technology use<br>» Enable the use of technology to maximise effectiveness and efficiency<br>» Enable the use of field-based technology (e.g., radio, general packet radio service (GPRS), satellite phone)
Advanced Technology Competency(ies):	7.2 Blockchain<br>7.3 Unmanned Aerial Vehicles (UAVs)<br>7.7 Automation<br>7.9 Additive Manufacturing<br>7.11 Cloud Computing<br>7.14 eProcurement
7.2 Blockchain	» Examine blockchain as a value-add capability for relevant global health use cases
7.3 Unmanned Aerial Vehicles (UAVs)	» Understand the current UAV landscape, their application and the enabling environment required to support country implementation<br>» Understand UAVs as a value-add capability for relevant global health use cases<br>» Understand the laws/ regulations around the use of UAV’s
7.7 Automation	» Understand automation as a value-add capability for relevant global health use cases
7.9 Additive Manufacturing	» Understand additive manufacturing as a value-add capability for relevant global health use cases
7.11 Cloud Computing	» Understand cloud computing as a value-add capability for relevant global health use cases

7.14 eProcurement	» Understand how to transact with an eProcurement system where necessary<br>» Understand eProcurement as a value-add capability for relevant global health use cases<br>» Understand the benefits and limitations of eProcurement to best utilise the system

Key Performance Indicators:

- % of emergency orders issued in the last 12 months
- % of product selection based on National Essential Medicines List
- % of procured products that meet Stringent Regulatory Authority (SRA) or WHO standards
- Supply adequacy - Net requirements are reflected in the Procurement pipeline to secure sufficient future supply.
- Supply Plan report; supplier performance report
- Fill Rate vs Planned fill rate
- Identify, Prioritize, and Aggregate Supply Chain Requirements Cycle Time - Actual Time taken to determine net supply chain requirements by product and SKU vs Planned Time
- Order accuracy - Net requirement versus order quantities in the planning horizon
- Ratio between median price of pro- ducts procured and the international median reference value
- Availability - Stock out rate - Number of missed line item quantity deliveries due to stock out versus total line item quantity deliveries
- Availability - Stock levels - Current on hand stock versus Planned on hand stock
- Data accuracy - Vendor Master data accuracy, item / SKU Master Data accuracy
- Demand analysis - Actual Demand versus Planned Demand
- Forecast Accuracy - Using various measures of Forecast Error e.g. MAD
- Inventory Days of Supply versus Planned Inventory days of Supply
- Reports submitted on time (for all and separate reports)

Training:	Qualifications Available:	Certifications Available:
» Contract Management<br>» Supplier Relationship Management (SRM)<br>» Analytical Skills<br>» Competitive Billing<br>» Negotiation<br>» Procurement/ RFQ Management		» CIPS Level 3 Advanced Certificate in Procurement and Supply Operations

Organisation:

Public Health Organisation – Intermediate Level

Competency Level:	Serial Number:	Primary Process:	Job Role:	Supervises:
Operational	3-33-25-3	Deliver	Officer – Receiving and Inventory Management	Assistant – Storage

Contributions	Timeous recording of receipts, ensuring that the required staff levels, handling equipment and space are available to minimize the risk of damage and shrinkage.
Technical Competency(ies):	2.5 Ensure quality of products<br>3.1 Undertake storage, warehousing and inventory management<br>3.1.1 Manage storage of commodities during emergency<br>3.3 Supply commodities to sections within a facility<br>3.5 Manage disposal of products (e.g. expired, damaged, redundant products)<br>3.5.1 Define and direct process for managing redundant and returned stock<br>3.7 Manage re-packing of products

2.5 Ensure quality of products

- Use and monitor the processes for prequalification of suppliers
- Ensure health commodities are not counterfeit and meet quality standards through certification (ISO, WHO GMP
- Describe the process of implementing, conducting and maintaining a reporting system of pharmacovigilance (e.g. report adverse drug reactions, quality defects, batch recall) and equipment faults
- Describe the process of inspecting products when delivered and during storage to catch defects or problems before they are given to, or needed by, clients
- Ensure compliance with good distribution and good storage practices (temperature monitoring in the warehouse, transportation and distribution)
- Define product-specific shelf-life by which the product are acceptable or rejected (e.g. no less than 1 year for most of drugs, and less for short shelf-life like laboratory)

3.1 Undertake storage, warehousing and inventory management

- Efficiently distribute products among suppliers, distribution centres, warehouses and customers through a logistics network
- Identify inputs to a total systems approach to designing and managing the entire flow of information, materials, and services
- Describe the choice of equipment or materials based on suitability for intended use, accuracy, safety of use and cost
- Maintain warehouses formal storage locations that identify the row, rack section, level and shelf location, typically with an alphanumeric location bar code or label

	» Describe how medicines are appropriately stored, considering temperature, access and cleanliness<br>» Identify appropriate use of, and ability to maintain, the cold chain<br>» Describe the movement and storage of materials within a warehouse<br>» Describe how to secure the medical store and limit access to staff<br>» Describe organisation processes, instructions, rules and parameters for warehouse and inventory management and use of equipment (e.g., annual stock turns, expiry date tracking, stock procurement, rotation and retrieval, equipment maintenance)<br>» Describe policies and procedures for stock handling, distribution and withdrawal consistent with maintaining safety (e.g., policies for distribution of concentrated electrolyte solutions and cytotoxics, identification of cytotoxic drug products within the work environment, separation of like-named or like-packaged products)<br>» Identify requirements of information on the supplies being stored<br>» Describe the process of consolidating several items into larger units for fewer handlings<br>» Describe methods of stock rotation (e.g., first in first out, FIFO, or first to expire first out, FEFO)<br>» Describe the process of entering the quantity received, and updating the warehouse management system software with the stock on hand when each order is delivered<br>» Asses required levels of stock and/or equipment in specific situations<br>» Ensure accurate verification of rolling stocks<br>» Monitor the location and condition of the supplies being stored<br>» Collate and prepare information on the storage locations and facilities<br>» Identify any problems in using the storage locations and facilities
3.1.1 Manage storage of commodities during emergency	» Describe the types of warehouses available for emergency or disaster supply<br>» Describe the importance of the choice of storage site for emergency or disaster supply<br>» Describe the staff required for the storage of emergency or disaster supply<br>» Describe the equipment and material required for the warehousing of emergency or disaster supply<br>» Provide input to a rapid assessment of logistical needs in emergencies
3.3 Supply commodities to sections within a facility	» Review imprest quantities regularly and assist in the development of the list in conjunction with doctors and nurses<br>» Validate the requisition from different health care services within the facility
3.5 Manage disposal of products (e.g. expired, damaged, redundant products)	

3.5.1 Define and direct process for managing redundant and returned stock	» Confirm information on the supplies being returned<br>» Describe the flow of returned supplies<br>» Identify any problems with the flow of returned supplies<br>» Integrate options for improving the flow of returned supplies<br>» Apply procedures for monitoring the flow of returned supplies<br>» Describe and/or demonstrate recall procedures to be used in response to a product recall notice or to access the information promptly.<br>» Identify issues relevant to the urgency and scope of action required in response to a product recall notice<br>» Identify the information required for reporting and procedures with regards to following up on medication incidents<br>» Identify follow-up strategies likely to be effective in preventing recurrence (e.g., root cause analysis)<br>» Describe an integrated healthcare waste management plan<br>» Describe guides and procedures for collection, sorting, transport and disposal<br>» Conduct pre-disposal inspections<br>» Describe the prerequisites for products disposal<br>» Generate product disposal report
3.7 Manage re-packing of products	» Predict when re-packing or pre-packs are required<br>» Demonstrate a safe system for repacking
Personal/Management Competency(ies):	6.1 Demonstrate generic skills (e.g., literacy, numeracy, technology)<br>6.1.1 Exhibit high understanding of literacy and numeracy<br>6.2 Demonstrate strong communication skills<br>6.2.1 Practice cultural awareness
6.1 Demonstrate generic skills (e.g., literacy, numeracy, technology)	
6.1.1 Exhibit high understanding of literacy and numeracy	» Speak clearly and confidently, organize information in a logical manner, and consider voice tone and pace<br>» Respond to verbal messages and other cues (active listening)<br>» Express ideas and opinions clearly in written and verbal form<br>» Communicate information accurately, concisely and confidently in writing and verbally<br>» Practice a level of mathematics suitable to the job held<br>» Describe visual presentation techniques, including charting, histograms and flow sheets<br>» Read and comprehend at a level necessary to properly complete duties of the position<br>» Interpret written information in documents, such as reports, SOPs, LMIS forms, graphs, calendars, schedules, notices and directions

- Pay attention to detail and identify the main ideas, detect inconsistencies and identify missing information in documents
- Apply computer skills — such as using Word, Excel, PowerPoint and the internet — suitable to the job level
- Describe the process of analysing and validating KPIs
- Participate in the evaluation of activities that act as standards against which the KPIs are measured
- Apply the process of performing critical self-review on work before submission

6.2 Demonstrate strong communication skills

- Be truthful and trustworthy, and supply accurate information at all times
- Work as part of a workplace team
- Describe vocabulary and communication style and form for both written and verbal communication that is appropriate for the situation, audience and material being communicated (e.g., avoid unnecessary jargon, clearly explain medical and SCM terminology)
- Encourage open communication within constraints of confidentiality
- Organize communications about logistics
- Encourage participation in meetings, expressing one's opinions, being aware of others' needs, and being appropriately assertive when required
- Apply perspective into the point of view of others, understanding their needs and goals
- Encourage building trust, rapport and credibility with others
- Anticipate the communication needs and concerns of others and respond to them
- Avoid conflict between work and personal interests
- Encourage meaningful two-way communication
- Influence others by persuasively presenting thoughts and ideas
- Apply assertiveness skills to deal with unreasonable requests and/or refusals that would compromise practice or consumer care
- Maintain a positive, supportive and appreciative attitude
- Apply the ability to balance the organisation's needs and the customer's needs
- Use self-control by maintaining composure and keeping emotions in check, even in difficult situations; deal calmly and effectively with stressful situations
- Encourage the expressing of opinions and providing information in written and/or verbal form in a manner that does not elicit concern, anger or other adverse response
- Apply the use of a systematic process for following up that demonstrates written reports have been received and understood
- Describe the means by which responses to input to the work environment are monitored
- Seek practical ways to overcome barriers to communication

	» Identify language representation (e.g., interpreters) at meetings when appropriate<br>» Tackle difficult situations and resolve disputes between staff<br>» Give both positive and negative feedback sensitively<br>» Maintain and improve communication<br>» Apply the process of giving and requesting feedback frequently<br>» Apply the ability to communicate effectively with shop stewards and union representatives
6.2.1 Practice cultural awareness	» Describe cultural awareness and sensitivity, treating all people with fairness, respect and dignity<br>» Describe anti-discriminatory practices in the organisation, including HR and disciplinary procedures<br>» Establish cultural sensitivity, equality and fairness at all levels of the organisation<br>» Challenge discriminatory behaviour directly and sensitively<br>» Act in a non-discriminatory way toward individuals and groups<br>» Integrate cultural awareness in learning and development approaches<br>» Avoid stereotypical responses by examining one's own behaviour and bias<br>» Manage cultural diversity in teams and make the most of differences<br>» Describe strategies and/or resources for communicating effectively with people from different cultural backgrounds<br>» Elicit information relating to values, beliefs and cultural backgrounds of consumers that may influence the way professional services are provided
Basal Technology Competency(ies):	7.4 Temperature and monitoring<br>7.6 ERP includes function of LMIS<br>7.12 Basic Office Skills<br>7.13 Have a command of technology
7.4 Temperature and monitoring	» Describe the current technology approaches for Temperature monitoring and sensors<br>» Describe what is required to implement this technology in a country context<br>» Describe infrastructure requirement for heat-sensitive items at peripheral 'lower level' of SC depending on volume and nature of items to handle;<br>» Define regular preventive maintenance and calibration of cold-chain equipment with biomedical engineers or logisticians<br>» Describe the policy for temperature monitoring tools during transportation and storage

	» Describe the steps involved in health logistic management cycle: selection-order-delivery-reception-storage-distribution-inventory<br>» Describe hardware qualification and system validation of temperature monitoring<br>» Consider mass data management of temperature monitoring
7.6 ERP includes function of LMIS	» Support a Master Data Management System<br>» Describe the critical components of an ERP system<br>» Describe the meaning of common document types found within an ERP/LMIS<br>» Describe the logic of data visibility within an ERP<br>» Describe the importance of data integrity, record management and information security
7.12 Basic Office Skills	» Have a good understanding of common presentation authoring packages<br>» Have a good understanding of common spreadsheet authoring packages<br>» Have a good understanding of common document authoring packages<br>» Have a good understanding of common email authoring packages<br>» Have a good understanding of common instant messaging packages<br>» Have a good understanding of common video conferencing authoring packages
7.13 Have a command of technology	» Apply the use of technology suitable to the job held<br>» Provide input on monitoring new developments and technologies in the sector<br>» Identify new technologies and recognising potential benefits for the sector<br>» Identify resources and support that are provided across the organisation to enable colleagues to make the best use of available technology<br>» Ensure that the organisation has a strategy for technology use<br>» Describe how to minimise environmental damage through technology use<br>» Use technology to maximise effectiveness and efficiency<br>» Use field-based technology (e.g., radio, general packet radio service (GPRS), satellite phone)
Advanced Technology Competency(ies):	7.7 Automation

7.7 Automation	» Describe automation as a value-add capability for relevant global health use cases

Key Performance Indicators:

- Availability - Stock out rate - Number of missed line item quantity deliveries due to stock out versus total line item quantity deliveries
- Availability - Stock levels - Current on hand stock versus Planned on hand stock
- Safe product handling - Number of products handled during period out of conformance versus Number of products handled during period expressed as a % with prior period comparisons
- Customer Incidents Report number of incidents by month/week/day comparison with previous periods
- No of Order Lines shipped with incorrectly captured Items - Number of order lines with incorrect items/SKU's versus total number of order lines shipped as a %
- Regulatory - % of procured products that meet Stringent Regulatory Authority (SRA) or WHO standards
- Waste Management - Existence of supply chain management protocols for disposal of medical waste and management of unusable products
- Number of order lines delivered to incorrect end-user - Number of orders incorrectly addressed versus number of orders processed expressed as a %
- Number of order lines delivered out of quality specifications - Number of Item/SKU delivered out of specification e.g. Cold Chain infractions, Expired products versus total Number of order lines delivered as a %. A comparative graph would be an appropriate tool for the measurement of this metric.
- % Product Transferred On-Time to Demand Requirement. Actual Number of products transferred outside of the Time Limits vs Number of products Transferred.
- Transfer Product Cycle Time - Actual Transfer Cycle Time versus Planned Transferred Cycle Time
- Accurate pick - Picking Accuracy number of order lines picked inaccurately divided by the total order lines picked (SKU and Quantity)
- On-time delivery - Actual Delivery Date & Time Vs Promised Delivery Data and Time
- Customer order fulfilment cycle time & rate - Actual Order Fulfilment Cycle Time versus Promised Customer Order Fulfilment Cycle time
- Warehousing and Inventory Management OTIF - On time and in full
- Number of warehouse locations used versus warehouse locations available (Pallet Positions may be used in larger warehouses)
- Line Items SKU's handled vs Items / SKU's Planned over a planning horizon
- Warehouse Utilization - Volume used vs Volume Available

Training:	Qualifications Available:	Certifications Available:
» Data management<br>» ERP Systems	» (CILT) International Diploma in Logistics & Transport	» SCPro™ Certification

» Legislation and Standards<br>» 3-way Receiving Match<br>» Code Handling/RFID (if available)<br>» Discrepancy Reporting and Resolution<br>» ID & Damage Inspection<br>» Inventory Management<br>» Property Control and Disposition<br>» Return Management<br>» MSDS/CoC/BoL/Environmental Interpretation		» APICS Certified in logistics transportation and distribution certificate<br>» Online Post Graduate Diploma in Global Health Procurement and Supply Chain Management

Manager - Occupational Safety

Organisation:

Public Health Organisation – Intermediate Level

Competency Level:	Serial Number:	Primary Process:	Job Role:	Supervises:
Operational	3-39-554-641-3	Enable – Regulatory (Individual) Compliance	Manager - Occupational Safety	-

Contributions	Responsible for compliance to the health and safety guidelines for the organisation. Promoting a safe and healthy working environment in the organisation. Responsible to reduce the number of work-related accidents and diseases in the organisation. Responsible to provide equitable compensation benefits to those who may get injured in work-related accidents or contract occupational diseases. Ensures the surveillance of the working environment
Technical Competency(ies):	3.9 Facility design

3.9 Facility design

» Provide inputs to the design and layout of the size and configuration of the facility/building.

» Describe the flow of product through the facility to making sure it is achievable with the selection of material handling equipment

» Achieve the balance of the health and safety requirements of the facility with its performance requirements

Personal/Management Competency(ies):	5.5 Manage and plan projects<br>5.5.4 Direct/participate in teamwork<br>6.1 Demonstrate generic skills (e.g., literacy, numeracy, technology)<br>6.1.1 Exhibit high understanding of literacy and numeracy<br>6.2 Demonstrate strong communication skills<br>6.2.1 Practice cultural awareness<br>6.4 Exhibit professional and ethical values<br>6.4.1 Demonstrate integrity

5.5 Manage and plan projects

5.5.4 Direct/participate in teamwork

» Describe the process of working with team members within their area of authority to establish achievable goals and strategies that are consistent with the objectives established for the organisation as a whole

» Describe an organisational chart that shows the lines of reporting and responsibility among staff

» Actively contribute a perspective and make a positive contribution to team-based problem solving and decision making

» Provide feedback, encouragement and support to team members for progressing strategic goals

	» Define team performance in relation to the organisation's mission and goals<br>» Encourage teams to think for themselves and resolve problems<br>» Encourage and harness diversity within the team to boost team effectiveness<br>» Encourage and support the team to work through its stages of development and perform well<br>» Describe roles and responsibilities in relation to employees' expertise and the expectations of collaborating team members<br>» Describe where their position fits in the structure (organisation, SC, healthcare sector) , the responsibilities their position entails and the accountabilities linked to the position
6.1 Demonstrate generic skills (e.g., literacy, numeracy, technology)	
6.1.1 Exhibit high understanding of literacy and numeracy	» Speak clearly and confidently, organize information in a logical manner, and consider voice tone and pace<br>» Respond to verbal messages and other cues (active listening)<br>» Express ideas and opinions clearly in written and verbal form<br>» Communicate information accurately, concisely and confidently in writing and verbally<br>» Practice a level of mathematics suitable to the job held<br>» Describe visual presentation techniques, including charting, histograms and flow sheets<br>» Read and comprehend at a level necessary to properly complete duties of the position<br>» Interpret written information in documents, such as reports, SOPs, LMIS forms, graphs, calendars, schedules, notices and directions<br>» Pay attention to detail and identify the main ideas, detect inconsistencies and identify missing information in documents<br>» Apply computer skills — such as using Word, Excel, PowerPoint and the internet — suitable to the job level<br>» Describe the process of analysing and validating KPIs<br>» Participate in the evaluation of activities that act as standards against which the KPIs are measured<br>» Apply the process of performing critical self-review on work before submission
6.2 Demonstrate strong communication skills	» Be truthful and trustworthy, and supply accurate information at all times<br>» Work as part of a workplace team<br>» Describe vocabulary and communication style and form for both written and verbal communication that is appropriate for the situation, audience and material being communicated (e.g., avoid unnecessary jargon, clearly explain medical and SCM terminology)<br>» Encourage open communication within constraints of confidentiality

- Organize communications about logistics
- Encourage participation in meetings, expressing one’s opinions, being aware of others’ needs, and being appropriately assertive when required
- Apply perspective into the point of view of others, understanding their needs and goals
- Encourage building trust, rapport and credibility with others
- Anticipate the communication needs and concerns of others and respond to them
- Avoid conflict between work and personal interests
- Encourage meaningful two-way communication
- Influence others by persuasively presenting thoughts and ideas
- Apply assertiveness skills to deal with unreasonable requests and/or refusals that would compromise practice or consumer care
- Maintain a positive, supportive and appreciative attitude
- Apply the ability to balance the organisation’s needs and the customer’s needs
- Use self-control by maintaining composure and keeping emotions in check, even in difficult situations; deal calmly and effectively with stressful situations
- Encourage the expressing of opinions and providing information in written and/or verbal form in a manner that does not elicit concern, anger or other adverse response
- Apply the use of a systematic process for following up that demonstrates written reports have been received and understood
- Describe the means by which responses to input to the work environment are monitored
- Seek practical ways to overcome barriers to communication
- Identify language representation (e.g., interpreters) at meetings when appropriate
- Tackle difficult situations and resolve disputes between staff
- Give both positive and negative feedback sensitively
- Maintain and improve communication
- Apply the process of giving and requesting feedback frequently
- Apply the ability to communicate effectively with shop stewards and union representatives

6.2.1 Practice cultural awareness

- Describe cultural awareness and sensitivity, treating all people with fairness, respect and dignity
- Describe anti-discriminatory practices in the organisation, including HR and disciplinary procedures
- Establish cultural sensitivity, equality and fairness at all levels of the organisation
- Challenge discriminatory behaviour directly and sensitively
- Act in a non-discriminatory way toward individuals and groups
- Integrate cultural awareness in learning and development approaches

» Avoid stereotypical responses by examining one's own behaviour and bias
» Manage cultural diversity in teams and make the most of differences
» Describe strategies and/or resources for communicating effectively with people from different cultural backgrounds
» Elicit information relating to values, beliefs and cultural backgrounds of consumers that may influence the way professional services are provided

6.4 Exhibit professional and ethical values

6.4.1 Demonstrate integrity

» Accept responsibility for one's own work tasks and performance
» Work within a framework of clearly understood humanitarian values and ethics
» Stand by decisions and hold others accountable when necessary
» Does not abuse one's own power or position
» Manage team members to ensure they do not abuse their power or position
» Identify when individuals or the organisation is straying from organisation goals, and challenge them to uphold ethics
» Resist undue political pressure in decision making
» Support staff in maintaining ethical stances
» Make time in team for ethical inquiry and reflection
» Show consistency between expressed principles and behaviour
» Provide input to ensure that principles, values and ethics are embedded in policy
» Act without consideration of personal gain
» Promote transparency in decision making structures and processes
» Provide input on ensuring programmes are acting with integrity, and recognising the impact of not doing so
» Recognise one's own limitations and act upon them
» Apply all standard operating procedures
» Work in a safe and legal way
» Demonstrate respect, dignity and consideration for consumers
» Discuss the impact of a no-blame culture on reporting and preventing recurrence of incidents
» Describe requests of colleagues that might be regarded as unreasonable
» Communicate (verbally and by example) expectations of the desired standards and approaches to be adopted
» Describe roles and responsibilities in terms of the position statement/duty statement of the position held
» Demonstrate punctuality
» Explain the obligation to apply professional care and expertise to deliver high quality of services

» Demonstrate care and attention to detail in undertaking work activities
» Describe appropriate attire and presentation for the role and situation
» Recognise and take responsibility for emotions
» Describe an environment in which others can talk and act without fear of repercussion
» Seek to keep commitments and not let people down
» Be trusting and cooperative when working alongside others
» Take on various responsibilities within the department as the need arises

Key Performance Indicators:
» Safety statics - Number of cases due to human error ranked by seriousness this period versus previous periods. A graphical representation would be indicated.<br>» Safety statics - Number of cases due to faulty equipment, ranked by seriousness of injury this period versus previous periods. A graphical representation would be indicated.<br>» Audit of safety equipment results this period versus previous periods<br>» Audit of Occupational safety signage and posters. This period versus previous periods<br>» Occupational safety educational awareness programmes delivered. Actual this period versus Planned this period<br>» Root cause analysis reports per reported incident. This period versus Last Period

Training:	Qualifications Available:	Certifications Available:
» SHE compliance training<br>» Reporting standards training		» Occupational Certificate: Compliance Officer<br>» Occupational Certificate: Maintenance Planner<br>» APICS Principles of operations management series certificate<br>» SCPro™ Certification<br>» Logistics and Supply Chain Management MIT

Officer – Performance Monitoring

Organisation:

Public Health Organisation – Intermediate Level

Competency Level:	Serial Number:	Primary Process:	Job Role:	Supervises:
Operational	5-531-532-52-2	Enable – SC Performance	Officer – Performance Monitoring	-

Contributions	Ensures the timely and accurate recording of transactional data prescribed by the Supply Chain Process Metrics at the appropriate transaction points, Creates and maintains the appropriate reporting infrastructure to disseminate the performance metric information in accordance with the Supply Chain RACI's
Personal/Management Competency(ies):	5.3 Implement risk management and monitoring and evaluation activities for the supply chain<br>5.3.1 Ensure monitoring and evaluation activities are completed<br>5.3.2 Implement risk management activities<br>6.1 Demonstrate generic skills (e.g., literacy, numeracy, technology)<br>6.1.1 Exhibit high understanding of literacy and numeracy<br>6.2 Demonstrate strong communication skills<br>6.2.1 Practice cultural awareness

5.3 Implement risk management and monitoring and evaluation activities for the supply chain

5.3.1 Ensure monitoring and evaluation activities are completed

- Understand the periodic evaluations to maintain processes by gathering pertinent information — such as problem symptoms from knowledgeable sources, carrying these through to the problems, potential causes and root causes of the problem
- Understand the process of quantifying the operational performance of similar companies and establish internal targets based on best-in-class results
- Enable key performance measurements and continuous process improvement initiatives to improve process quality on a continual basis
- Understand the need to provide results of the analyses to colleagues
- Understand of the need to control and check errors, taking corrective action so deviation from standards are minimised and the organisation’s goals are achieved
- Examine the use of appropriate technological developments to improve the system
- Understand that continuous process improvement is an accepted way of organisational life
- Examine ways of eliminating unnecessary steps in system design
- Enable processes that strive to eliminate waste

	» Enable processes to encourage sustainability (e.g., reducing carbon footprint, establishing a paperless office, using renewable energy)<br>» Understand the systematic approach used to close process or system performance gaps through streamlining and cycle time reduction, and identify and eliminate causes of quality below specifications, process variation and non-value-adding activities<br>» Examine the relationship between technology and process functionality<br>» Understand how policy, guidelines and regulatory reforms impact SC performance
5.3.2 Implement risk management activities	» Examine risks affecting supply, transformation, delivery and customer demand<br>» Understand what constitutes unsafe working conditions and how to take prompt, corrective action; stay alert to and take preventive action against hazards and threats; follow the process of recommending measures to protect employees from hazardous working conditions ; follow protocol for reporting safety violations<br>» Follow training programmes/drills in which personnel participate (e.g., fire and armed hold-up drills, safe manual handling practices, use of mandatory safety clothing)<br>» Enable systems of review (e.g., audit) to check compliance with policies and procedures intended to maintain workplace safety (e.g., manual handling techniques, correct use of protective clothing)<br>» Ensure equipment and tools are operating to prescribed standards<br>» Enable key security systems for the workplace (e.g., for cash, narcotics and other controlled substances, investigational drugs, consumer records, entry and exit points) and levels of access and/or authority applicable to each<br>» Follow the prevention strategies adopted (e.g., protocols, security barriers, fixed or personal duress alarms)<br>» Understand the need to document critical incidents<br>» Understand contingency planning and the need to manage operational continuity<br>» Understand what constitutes a safe working environment and engender a shared commitment from all personnel to creating one<br>» Understand relevant safety and health laws and regulations; comply with safety codes, standards and guidelines; adhere to worksite safety programmes; understand emergency response plans when they exist<br>» Understand how to handle hazardous commodities and first aid procedures
6.1 Demonstrate generic skills (e.g., literacy, numeracy, technology)	
6.1.1 Exhibit high understanding of literacy and numeracy	» Speak clearly and confidently, organize information in a logical manner, and consider voice tone and pace<br>» Respond to verbal messages and other cues (active listening)<br>» Express ideas and opinions clearly in written and verbal form

» Communicate information accurately, concisely and confidently in writing and verbally

» Practice a level of mathematics suitable to the job held

» Compare visual presentation techniques, including charting, histograms and flow sheets

» Read and comprehend at a level necessary to properly complete duties of the position

» Understand the need to interpret written information in documents, such as reports, SOPs, LMIS forms, graphs, calendars, schedules, notices and directions

» Understand the need to pay attention to detail and identify the main ideas, detect inconsistencies and identify missing information in documents

» Enable computer skills — such as using Word, Excel, PowerPoint and the internet — suitable to the job level

» Understand the process of analysing and validating KPIs

» Understand how the evaluation process of activities that act as standards against which the KPIs are measured is carried out

» Examine the process of performing critical self-review on work before submission

6.2 Demonstrate strong communication skills

» Be truthful and trustworthy, and supply accurate information at all times

» Enable working as part of a workplace team

» Understand vocabulary and communication style and form for both written and verbal communication that is appropriate for the situation, audience and material being communicated (e.g., avoid unnecessary jargon, clearly explain medical and SCM terminology)

» Enable open communication within constraints of confidentiality

» Participate in the organisation of communications about logistics

» Enable participation in meetings, expressing one's opinions, being aware of others' needs, and being appropriately assertive when required

» Show perspective into the point of view of others, understanding their needs and goals

» Enable the practise of building trust, rapport and credibility with others

» Understand the communication needs and concerns of others and respond to them

» Understand the need to avoid conflict between work and personal interests

» Enable meaningful two-way communication

» Show influence on others by persuasively presenting thoughts and ideas

» Show assertiveness skills to deal with unreasonable requests and/or refusals that would compromise practice or consumer care

» Maintain a positive, supportive and appreciative attitude

» Show the ability to balance the organisation's needs and the customer's needs

» Show self-control by maintaining composure and keeping emotions in check, even in difficult situations; deal calmly and effectively with stressful situations

	» Enable expressing of opinions and providing information in written and/or verbal form in a manner that does not elicit concern, anger or other adverse response<br>» Enable the use of a systematic process for following up that demonstrates written reports have been received and understood<br>» Understand the means by which responses to input to the work environment are monitored<br>» Seek practical ways to overcome barriers to communication<br>» Understand the need to ensuring language representation (e.g., interpreters) at meetings when appropriate<br>» Interpret difficult situations and disputes between staff that need to be tackled and resolved<br>» Enable giving both positive and negative feedback sensitively<br>» Understand the need to maintain and improve communication<br>» Examine the process of giving and requesting feedback frequently<br>» Examine effective communication with shop stewards and union representatives
6.2.1 Practice cultural awareness	» Understand the need for cultural awareness and sensitivity, treating all people with fairness, respect and dignity<br>» Enable anti-discriminatory practices in the organisation, including HR and disciplinary procedures<br>» Enable cultural sensitivity, equality and fairness at all levels of the organisation<br>» Challenge discriminatory behaviour directly and sensitively<br>» Act in a non-discriminatory way toward individuals and groups<br>» Enable integrating cultural awareness in learning and development approaches<br>» Avoid stereotypical responses by examining one's own behaviour and bias<br>» Enable managing cultural diversity in teams and make the most of differences<br>» Compare strategies and/or resources for communicating effectively with people from different cultural backgrounds<br>» Enable the process to elicit information relating to values, beliefs and cultural backgrounds of consumers that may influence the way professional services are provided
Basal Technology Competency(ies):	7.6 ERP includes function of LMIS<br>7.12 Basic Office Skills<br>7.13 Have a command of technology
7.6 ERP includes function of LMIS	» Enable a Master Data Management System<br>» Understand the critical components of an ERP system<br>» Understand the meaning of common document types found within an ERP/LMIS<br>» Understand the logic of data visibility within an ERP

	» Understand the importance of data integrity, record management and information security
7.12 Basic Office Skills	» Have a good understanding of common presentation authoring packages<br>» Have a good understanding of common spreadsheet authoring packages<br>» Have a good understanding of common document authoring packages<br>» Have a good understanding of common email authoring packages<br>» Have a good understanding of common instant messaging packages<br>» Have a good understanding of common video conferencing authoring packages
7.13 Have a command of technology	» Enable the use of technology suitable to the job held<br>» Understand the need of monitoring new developments and technologies in the sector<br>» Compare new technologies and recognising potential benefits for the sector<br>» Understand that resources and support are provided across the organisation to enable colleagues to make the best use of available technology<br>» Understand that the organisation has a strategy for technology use<br>» Compare how to minimise environmental damage through technology use<br>» Enable the use of technology to maximise effectiveness and efficiency<br>» Enable the use of field-based technology (e.g., radio, general packet radio service (GPRS), satellite phone)
Advanced Technology Competency(ies):	7.2 Blockchain<br>7.7 Automation<br>7.9 Additive Manufacturing<br>7.10 Internet of things<br>7.14 eProcurement
7.2 Blockchain	» Examine blockchain as a value-add capability for relevant global health use cases
7.7 Automation	» Understand automation as a value-add capability for relevant global health use cases
7.9 Additive Manufacturing	» Understand additive manufacturing as a value-add capability for relevant global health use cases
7.10 Internet of things (IoT)	» Understand the opportunity of IoT used in conjunction with AI

	» Understand the internet of things as a value-add capability for relevant global health use cases
7.14 eProcurement	» Understand how to transact with an eProcurement system where necessary<br>» Understand eProcurement as a value-add capability for relevant global health use cases<br>» Understand the benefits and limitations of eProcurement to best utilise the system

Key Performance Indicators:

- » Assess Delivery Performance Cycle Time - Actual Time from assessed requirement to time delivered to end-user compared to committed Delivery Cycle Time as per Supply Chain strategy
- » Assess Supplier Performance Cycle Time - Actual Supplier performance Cycle time versus Supplier commitment cycle time
- » Inventory accuracy - Cycle count results
- » Transportation lead time accuracy - Actual Transportation lead time versus Transportation promised lead time.
- » Transport efficiency - Total cost of transport versus planned cost of transport by mode
- » In-bound cost of acquisition - Actual total cost of acquisition versus Total Planned cost of acquisition including landed costs
- » Manage performance cycle time
- » Performance Report - Actual versus Plan reports for all metrics
- » Work/activities executed in alignment with SOP's
- » Reports available on time

Training:	Qualifications Available:	Certifications Available:
» RP Systems<br>» Basic Finance<br>» Benchmarking<br>» Lean Manufacturing<br>» Performance Management<br>» Optimization<br>» Quality Management<br>» Six Sigma<br>» Supply Chain Performance Measurements<br>» Total Quality Management (TQM)<br>» Risk Management<br>» Monitoring and Evaluation		» Manage Logistics Operations skills programme<br>» Apply fundamental concepts and principles of supply chain management skills programme

Tactical
Assistant – Storage

Organisation: **Public Health Organisation – Intermediate Level**				
Competency Level:	Serial Number:	Primary Process:	Job Role:	Supervises:
**Tactical**	**3-33-25-1**	**Deliver**	**Assistant – Storage**	**-**
Contributions	Performs the actual receiving, storage and disposition in accordance with the protocols governing the items being returned.			
Technical Competency(ies):	3.1 Undertake storage, warehousing and inventory management<br>3.1.1 Manage storage of commodities during emergency<br>3.3 Supply commodities to sections within a facility			

3.1 Undertake storage, warehousing and inventory management

» Execute distribution of products among suppliers, distribution centres, warehouses and customers through a logistics network

» Be aware of a total systems approach to designing and managing the entire flow of information, materials, and services

» Awareness of the choice of equipment or materials based on suitability for intended use, accuracy, safety of use and cost

» Awareness of the formal storage locations in the warehouse that identify the row, rack section, level and shelf location, typically with an alphanumeric location bar code or label

» Store medicines appropriately, considering temperature, access and cleanliness

» Ability to maintain the cold chain

» Execute the movement and storage of materials within a warehouse

» Be aware of the need to secure the medical store and limit access to staff

» Follow organisation processes, instructions, rules and parameters for warehouse and inventory management and use of equipment (e.g., annual stock turns, expiry date tracking, stock procurement, rotation and retrieval, equipment maintenance)

» Awareness of policies and procedures for stock handling, distribution and withdrawal consistent with maintaining safety (e.g., policies for distribution of concentrated electrolyte solutions and cytotoxics, identification of cytotoxic drug products within the work environment, separation of like-named or like-packaged products)

» Confirm information on the supplies being stored

» Awareness that one must consolidate several items into larger units for fewer handlings

	» Awareness of methods of stock rotation (e.g., first in first out, FIFO, or first to expire first out, FEFO)<br>» Enter the quantity received and update the warehouse/inventory/LMIS system software with the stock on hand when each order is delivered according to requisite SOP's<br>» Awareness that there are required levels of stock and/or equipment in specific situations<br>» Awareness of the importance of accurate verification of rolling stocks<br>» Awareness of the importance of monitoring the location and condition of the supplies being stored<br>» Awareness of the importance of the information on the storage locations and facilities<br>» Awareness of the need to communicate any problems in using the storage locations and facilities
3.1.1 Manage storage of commodities during emergency	» Awareness of the types of warehouses available for emergency or disaster supply<br>» Awareness of the choice of storage site for emergency or disaster supply<br>» Awareness of the staff required for the storage of emergency or disaster supply<br>» Awareness of the equipment and material required for the warehousing of emergency or disaster supply<br>» Awareness of the need for a rapid assessment of logistical needs in emergencies
3.3 Supply commodities to sections within a facility	» Awareness of the use of an imprest list<br>» Awareness of the process of validating the requisition from different health care services within the facility
Personal/Management Competency(ies):	6.1 Demonstrate generic skills (e.g., literacy, numeracy, technology)<br>6.1.1 Exhibit high understanding of literacy and numeracy<br>6.2 Demonstrate strong communication skills<br>6.2.1 Practice cultural awareness
6.1 Demonstrate generic skills (e.g., literacy, numeracy, technology)	
6.1.1 Exhibit high understanding of literacy and numeracy	» Speak clearly and confidently, organize information in a logical manner, and consider voice tone and pace<br>» Respond to verbal messages and other cues (active listening)<br>» Express ideas and opinions clearly in written and verbal form<br>» Communicate information accurately, concisely and confidently in writing and verbally<br>» Practice a level of mathematics suitable to the job held<br>» Awareness of visual presentation techniques, including charting, histograms and flow sheets

	» Read and comprehend at a level necessary to properly complete duties of the position<br>» Awareness of the need to interpret written information in documents, such as reports, SOPs, LMIS forms, graphs, calendars, schedules, notices and directions<br>» Awareness of the need to paying attention to detail and identifying the main ideas, detecting inconsistencies and identifying missing information in documents<br>» Execute computer skills — such as using Word, Excel, PowerPoint and the internet — suitable to the job level<br>» Operational knowledge of analysing and validating KPIs<br>» Awareness of how the evaluation process of activities that act as standards against which the KPIs are measured is carried out<br>» Execute the process of performing critical self-review on work before submission
6.2 Demonstrate strong communication skills	» Be truthful and trustworthy, and supply accurate information at all times<br>» Realize the need to work as part of a workplace team<br>» Awareness of vocabulary and communication style and form for both written and verbal communication that is appropriate for the situation, audience and material being communicated (e.g., avoid unnecessary jargon, clearly explain medical and SCM terminology)<br>» Awareness of open communication within constraints of confidentiality<br>» Awareness of communications about logistics<br>» Participate in meetings, expressing one's opinions, being aware of others' needs, and being appropriately assertive when required<br>» Awareness of perspective into the point of view of others, understanding their needs and goals<br>» Build trust, rapport and credibility with others<br>» Awareness of the communication needs and concerns of others and respond to them<br>» Awareness of the need to avoid conflict between work and personal interests<br>» Practice meaningful two-way communication<br>» Realize that others are influenced by persuasively presenting thoughts and ideas<br>» Awareness of the need for assertiveness skills to deal with unreasonable requests and/or refusals that would compromise practice or consumer care<br>» Maintain a positive, supportive and appreciative attitude<br>» Awareness of the need to balance the organisation's needs and the customer's needs<br>» Execute self-control by maintaining composure and keeping emotions in check, even in difficult situations; deal calmly and effectively with stressful situations<br>» Express opinions and provide information in written and/or verbal form in a manner that does not elicit concern, anger or other adverse response

	» Basic operational knowledge of a systematic process for following up that demonstrates written reports have been received and understood<br>» Basic operational knowledge of the means by which responses to input to the work environment are monitored<br>» Seek practical ways to overcome barriers to communication<br>» Awareness of the need for language representation (e.g., interpreters) at meetings when appropriate<br>» Realize that difficult situations and disputes between staff need to be tackled and resolved<br>» Realize the need to give both positive and negative feedback sensitively<br>» Awareness of the need to maintain and improve communication<br>» Execute the process of giving and requesting feedback frequently<br>» Execute effective communication with shop stewards and union representatives
6.2.1 Practice cultural awareness	» Awareness of the need for cultural awareness and sensitivity, treating all people with fairness, respect and dignity<br>» Awareness of the anti-discriminatory practices in the organisation, including HR and disciplinary procedures<br>» Awareness of the need for cultural sensitivity, equality and fairness at all levels of the organisation<br>» Challenge discriminatory behaviour directly and sensitively<br>» Act in a non-discriminatory way toward individuals and groups<br>» Awareness of the need for integrating cultural awareness in learning and development approaches<br>» Avoid stereotypical responses by examining one's own behaviour and bias<br>» Awareness of the need to manage cultural diversity in teams and make the most of differences<br>» Awareness of strategies and/or resources for communicating effectively with people from different cultural backgrounds<br>» Awareness of the need to elicit information relating to values, beliefs and cultural backgrounds of consumers that may influence the way professional services are provided
Basal Technology Competency(ies):	7.6 ERP includes function of LMIS<br>7.13 Have a command of technology
7.6 ERP includes function of LMIS	» Awareness of a Master Data Management System<br>» Awareness of the critical components of an ERP system<br>» Awareness of the meaning of common document types found within an ERP/LMIS<br>» Awareness of the logic of data visibility within an ERP<br>» Awareness of the importance of data integrity, record management and information security

7.13 Have a command of technology	» Execute the use of technology suitable to the job held<br>» Awareness of the need to monitor new developments and technologies in the sector<br>» Awareness of new technologies and recognising potential benefits for the sector<br>» Awareness that resources and support are provided across the organisation to enable colleagues to make the best use of available technology<br>» Awareness of the fact that the organisation has a strategy for technology use<br>» Awareness of how to minimise environmental damage through technology use<br>» Basic operational knowledge of technology to maximise effectiveness and efficiency<br>» Basic operational knowledge of field-based technology (e.g., radio, general packet radio service (GPRS), satellite phone)
Advanced Technology Competency(ies):	7.7 Automation
7.7 Automation	» Awareness of automation as a value-add capability for relevant global health use cases

Key Performance Indicators:

- Availability - Stock out rate - Number of missed line item quantity deliveries due to stock out versus total line item quantity deliveries
- Availability - Stock levels - Current on hand stock versus Planned on hand stock
- Safe product handling - Number of products handled during period out of conformance versus Number of products handled during period expressed as a % with prior period comparisons
- Customer Incidents Report number of incidents by month/week/day comparison with previous periods
- No of Order Lines shipped with incorrectly captured Items - Number of order lines with incorrect items/SKU's versus total number of order lines shipped as a %
- Regulatory - % of procured products that meet Stringent Regulatory Authority (SRA) or WHO standards
- Waste Management - Existence of supply chain management protocols for disposal of medical waste and management of unusable products
- Number of Order Lines delivered to incorrect end-user - Number of orders incorrectly addressed versus number of orders processed expressed as a %
- Number of Order Lines delivered out of Quality Specifications - Number of Item/SKU delivered out of specification e.g. Cold Chain infractions, Expired products versus total Number of order lines delivered as a %. A comparative graph would be an appropriate tool for the measurement of this metric.
- % Product Transferred On-Time to Demand Requirement. Actual Number of products transferred outside of the Time Limits vs Number of products Transferred.
- Transfer Product Cycle Time - Actual Transfer Cycle Time versus Planned Transferred Cycle Time
- Accurate pick - Picking Accuracy number of order lines picked inaccurately divided by the total order lines picked (SKU and Quantity)

- On-time delivery - Actual Delivery Date & Time Vs Promised Delivery Data and Time
- Customer order fulfilment cycle time & rate - Actual Order Fulfilment Cycle Time versus Promised Customer Order Fulfilment Cycle time
- Warehousing and Inventory Management OTIF - On time and in full

Training:	Qualifications Available:	Certifications Available:
» Material handling equipment usage		» Occupational Certificate: Store Person<br>» Occupational Certificate: Dispatching and Receiving Clerk<br>» Describe the use of stores and warehousing in operations skills programme

Public Health Organisation – Hospital Service Delivery

Managerial

Head – Demand and Supply Planning

Organisation: Public Health Organisation – Hospital Service Delivery				
Competency Level:	Serial Number:	Primary Process:	Job Role:	Supervises:
Managerial	1-14-15-13-3	Plan – Procurement	Head – Demand and Supply Planning	Officer – Demand and Supply Planning

Contributions	Articulates the Supply Chain Strategy to the Procurement functionality within the context. Ensures that the Sourcing/Procurement department is enabled to supply the public health system in line with the Supply Chain Materials and Resource Plan., Facilitates the execution of the Supply Strategy in the context, Implements best practices to extract the most efficiency from each process and guarantee supply accuracy, lead time and cost diminution as well as data accuracy<br>.
Technical Competency(ies):	» Select the appropriate product<br>» Define the specifications of the product, including product quality<br>» List any special considerations for the product (e.g., temperature requirements, size, implications for infrastructure)<br>» Forecast and quantify product needs<br>» Supply planning
1.1 Select the appropriate product	» Describe the broad concepts of the national medicines policy, essential medicines lists, essential equipment lists, standard treatment guidelines and “dangerous drug” (DDA) policy<br>» Use the government system required to add and subtract items from the essential medicines list and the essential equipment list<br>» Follow the government system required to alter standard treatment guidelines, dangerous drug policy and national medicines policy<br>» Confirm the type of supplies and services that are required<br>» Participate in regularly scheduled coordination meetings with stakeholders involved in financing, procuring or distributing commodities<br>» Describe the steps needed to bring a medicinal product to the market, including the safety, quality, efficacy and pharmacoeconomic assessments of the product

1.2 Define the specifications of the product, including product quality	» Define specifications for procurement<br>» Describe the characteristics of a good specification<br>» Discuss the types of specifications<br>» Identify the advantages of functional and performance specifications<br>» List the contents of a specification<br>» Identify the procurement staff responsibilities in the specification process<br>» Describe the quality standard of product<br>» Ensure that solution meets specification requirements
1.3 List any special considerations for the product (e.g., temperature requirements, size, implications for infrastructure)	» Describe the principles and processes of category management, including market segmentation principles<br>» List any specific considerations in the quantification of programme-specific products (e.g., ARVs, family planning commodities, vaccines)<br>» Describe current international trends in commodity availability<br>» Define regulatory requirements for the product
1.4 Forecast and quantify product needs	» Identify the factors that affect usage patterns of medications and equipment and how this affects ordering (e.g., disease outbreaks), using national policies as a guide and to ensure consistent application<br>» Apply principles and applications of demand forecasting<br>» Apply the critical requirements for effective forecasting: establishing time horizons, level of detail and use of data<br>» Describe policies and procedures for forecasting<br>» Quantify product requirements using a variety of methods<br>» Organize and summarize consumption data at the national level for various country programmes<br>» Describe the use different types of forecasts<br>» Apply VEN or ABC analysis to programme requirements for national level procurement<br>» Calculate average monthly dispensed-to-user quantities for all service delivery points nationally<br>» Calculate storage space requirements for all levels in the supply chain<br>» Calculate the months of supply on hand for each commodity at the national level<br>» Describe the maximum and minimum stock levels for each level in a programme<br>» Use various tools used in quantification (e.g., quantification software)<br>» Describe the use of various data sources for commodity forecasting (e.g., consumption data, services data, demographic data)<br>» Establish key performance indicators of forecast accuracy<br>» Establish the degree of error when using forecasting methods
1.5 Supply planning	» Describe a purchasing and supply plan in line with national priorities

- Balance supply chain resources and trigger activities to correct any imbalances
- Explain the formalisation of sales and operations plans through sales and operations meeting.
- Translate sales and operations plan into executable plans to ensure dispatch of goods in line with overall priorities.
- Describe the national commodity pipeline

Personal/Management Competency(ies):	6.1 Demonstrate generic skills (e.g., literacy, numeracy, technology)<br>6.1.1 Exhibit high understanding of literacy and numeracy<br>6.2 Demonstrate strong communication skills<br>6.2.1 Practice cultural awareness

6.1 Demonstrate generic skills (e.g., literacy, numeracy, technology)

6.1.1 Exhibit high understanding of literacy and numeracy

- Speak clearly and confidently, organize information in a logical manner, and consider voice tone and pace
- Respond to verbal messages and other cues (active listening)
- Express ideas and opinions clearly in written and verbal form
- Communicate information accurately, concisely and confidently in writing and verbally
- Practice a level of mathematics suitable to the job held
- Describe visual presentation techniques, including charting, histograms and flow sheets
- Read and comprehend at a level necessary to properly complete duties of the position
- Interpret written information in documents, such as reports, SOPs, LMIS forms, graphs, calendars, schedules, notices and directions
- Pay attention to detail and identify the main ideas, detect inconsistencies and identify missing information in documents
- Apply computer skills — such as using Word, Excel, PowerPoint and the internet — suitable to the job level
- Describe the process of analysing and validating KPIs
- Participate in the evaluation of activities that act as standards against which the KPIs are measured
- Apply the process of performing critical self-review on work before submission

6.2 Demonstrate strong communication skills

- Be truthful and trustworthy, and supply accurate information at all times
- Work as part of a workplace team
- Describe vocabulary and communication style and form for both written and verbal communication that is appropriate for the situation, audience and material being communicated (e.g., avoid unnecessary jargon, clearly explain medical and SCM terminology)

	» Encourage open communication within constraints of confidentiality<br>» Organize communications about logistics<br>» Encourage participation in meetings, expressing one's opinions, being aware of others' needs, and being appropriately assertive when required<br>» Apply perspective into the point of view of others, understanding their needs and goals<br>» Encourage building trust, rapport and credibility with others<br>» Anticipate the communication needs and concerns of others and respond to them<br>» Avoid conflict between work and personal interests<br>» Encourage meaningful two-way communication<br>» Influence others by persuasively presenting thoughts and ideas<br>» Apply assertiveness skills to deal with unreasonable requests and/or refusals that would compromise practice or consumer care<br>» Maintain a positive, supportive and appreciative attitude<br>» Apply the ability to balance the organisation's needs and the customer's needs<br>» Use self-control by maintaining composure and keeping emotions in check, even in difficult situations; deal calmly and effectively with stressful situations<br>» Encourage the expressing of opinions and providing information in written and/or verbal form in a manner that does not elicit concern, anger or other adverse response<br>» Apply the use of a systematic process for following up that demonstrates written reports have been received and understood<br>» Describe the means by which responses to input to the work environment are monitored<br>» Seek practical ways to overcome barriers to communication<br>» Identify language representation (e.g., interpreters) at meetings when appropriate<br>» Tackle difficult situations and resolve disputes between staff<br>» Give both positive and negative feedback sensitively<br>» Maintain and improve communication<br>» Apply the process of giving and requesting feedback frequently<br>» Apply the ability to communicate effectively with shop stewards and union representatives
6.2.1 Practice cultural awareness	» Describe cultural awareness and sensitivity, treating all people with fairness, respect and dignity<br>» Describe anti-discriminatory practices in the organisation, including HR and disciplinary procedures<br>» Establish cultural sensitivity, equality and fairness at all levels of the organisation<br>» Challenge discriminatory behaviour directly and sensitively

- Act in a non-discriminatory way toward individuals and groups
- Integrate cultural awareness in learning and development approaches
- Avoid stereotypical responses by examining one's own behaviour and bias
- Manage cultural diversity in teams and make the most of differences
- Describe strategies and/or resources for communicating effectively with people from different cultural backgrounds
- Elicit information relating to values, beliefs and cultural backgrounds of consumers that may influence the way professional services are provided

Basal Technology Competency(ies):	7.4 Temperature and monitoring<br>7.6 ERP includes function of LMIS<br>7.12 Basic Office Skills<br>7.13 Have a command of technology
7.4 Temperature and monitoring	» Describe the current technology approaches for Temperature monitoring and sensors<br>» Describe what is required to implement this technology in a country context<br>» Describe infrastructure requirement for heat-sensitive items at peripheral 'lower level' of SC depending on volume and nature of items to handle;<br>» Define regular preventive maintenance and calibration of cold-chain equipment with biomedical engineers or logisticians<br>» Describe the policy for temperature monitoring tools during transportation and storage<br>» Describe the steps involved in health logistic management cycle: selection-order-delivery-reception-storage-distribution-inventory<br>» Describe hardware qualification and system validation of temperature monitoring<br>» Consider mass data management of temperature monitoring
7.6 ERP includes function of LMIS	» Support a Master Data Management System<br>» Describe the critical components of an ERP system<br>» Describe the meaning of common document types found within an ERP/LMIS<br>» Describe the logic of data visibility within an ERP<br>» Describe the importance of data integrity, record management and information security
7.12 Basic Office Skills	» Have a good understanding of common presentation authoring packages<br>» Have a good understanding of common spreadsheet authoring packages<br>» Have a good understanding of common document authoring packages<br>» Have a good understanding of common email authoring packages

	» Have a good understanding of common instant messaging packages<br>» Have a good understanding of common video conferencing authoring packages
7.13 Have a command of technology	» Apply the use of technology suitable to the job held<br>» Provide input on monitoring new developments and technologies in the sector<br>» Identify new technologies and recognising potential benefits for the sector<br>» Identify resources and support that are provided across the organisation to enable colleagues to make the best use of available technology<br>» Ensure that the organisation has a strategy for technology use<br>» Describe how to minimise environmental damage through technology use<br>» Use technology to maximise effectiveness and efficiency<br>» Use field-based technology (e.g., radio, general packet radio service (GPRS), satellite phone)
Advanced Technology Competency(ies):	7.2 Blockchain<br>7.3 Unmanned Aerial Vehicles (UAVs)<br>7.7 Automation<br>7.9 Additive Manufacturing<br>7.11 Cloud Computing<br>7.14 eProcurement
7.2 Blockchain	» Describe blockchain as a value-add capability for relevant global health use cases
7.3 Unmanned Aerial Vehicles (UAVs)	» Describe the current UAV landscape, their application and the enabling environment required to support country implementation<br>» Describe UAVs as a value-add capability for relevant global health use cases<br>» Describe the laws/ regulations around the use of UAV's
7.7 Automation	» Describe automation as a value-add capability for relevant global health use cases
7.9 Additive Manufacturing	» Describe additive manufacturing as a value-add capability for relevant global health use cases

7.11 Cloud Computing	» Describe cloud computing as a value-add capability for relevant global health use cases
7.14 eProcurement	» Describe how to transact with an eProcurement system where necessary<br>» Describe eProcurement as a value-add capability for relevant global health use cases<br>» Apply the benefits and limitations of eProcurement to best utilise the system

Key Performance Indicators:

- » Vendor on time delivery (VOTD)
- » % of emergency orders issued in the last 12 months
- » % of product selection based on National Essential Medicines List
- » % of procured products that meet Stringent Regulatory Authority (SRA) or WHO standards
- » Supply adequacy - Net requirements are reflected in the Procurement pipeline to secure sufficient future supply.
- » Supply Plan report; supplier performance report
- » Data accuracy - Vendor Master data accuracy, item / SKU Master Data accuracy
- » Fill Rate vs Planned fill rate
- » Forecast accuracy, measured and reported on.
- » Identify, Prioritize, and Aggregate Supply Chain Requirements Cycle Time - Actual Time taken to determine net supply chain requirements by product and SKU vs Planned Time
- » Inventory Days of Supply - Actual inventory - Planned Demand over the planning horizon divided by the average daily usage
- » Order accuracy - Net requirement versus order quantities in the planning horizon
- » Ratio between median price of pro- ducts procured and the international median reference value
- » Supply Chain Planning Cost recorded and measured against required Supply China Planning Cycle times to accomplish Supply Chain Strategic objectives.
- » Availability - Stock out rate - Number of missed line item quantity deliveries due to stock out versus total line item quantity deliveries
- » Availability - Stock levels - Current on hand stock versus Planned on hand stock
- » Forecasting and Supply Planning done within the specified cycle time to support the Supply Chain performance requirements.
- » Establish Product Demand and Resources Cycle Time and recorded.in the "ERP" system against the products and resources.
- » Establish Supply Plan Cycle Times and recorded in the "ERP" system.
- » Material and Resource Plan valid. Number of valid actions performed versus number of actions performed. (Exception messages trigger due to invalid schedules)

- Procurement planning Cycle Time, by product and resource. Actual Overall planning cycle versus required Overall planning cycle time to achieve Supply Chain performance requirements.
- Reports submitted on time (for all and separate reports) in accordance with business rules (reporting calendar)

Training:	Qualifications Available:	Certifications Available:
» Risk and exception management<br>» Import & export regulations for products<br>» Inventory management, Min-Max Replenishment<br>» ABC/VEN Inventory Classification	» Diploma in International Trade Management in Exports/Imports	» Occupational Certificate: Procurement Officer<br>» CIPS Level 4 Diploma in Procurement and Supply<br>» SPSM2® (Global Procurement Management)

Lead - Procurement

Organisation: Public Health Organisation – Hospital Service Delivery				
Competency Level:	Serial Number:	Primary Process:	Job Role:	Supervises:
Managerial	2-21-22-4	Enable – Procurement	Lead - Procurement	Officer– Procurement, Lead – Returns and Disposal, Officer – Procurement and Supply

Contributions	Leads the procurement department within context to enable the procurement processes as defined in the Supply Chain design. Ensures that Procurement Master Data is adequate to eliminate bottlenecks in the procurement process.
Technical Competency(ies):	2.1 Manage procurement costs and budget<br>2.2 Build and maintain supplier relationships

2.1 Manage procurement costs and budget	» Ensure the organisation follows public procurement regulations<br>» Ensure adherence to the approved procurement budget<br>» Evaluate financial and management accounting practices within procurement<br>» Analyse commercial factors that contribute toward cost reduction, price savings and value improvement opportunities<br>» Analyse the principles of foreign exchange rates and how they impact prices
2.2 Build and maintain supplier relationships	» Analyse the market<br>» Analyse adherence to the processes for prequalification and tender contracting<br>» Determine the factors that define a good supplier<br>» Effectively locate and source key suppliers, while analysing the total cost associated with procuring an item or service<br>» Analyse supplier relationship management plans (e.g., sourcing, frequency of meetings, negotiate and monitor benchmarks for performance)<br>» Analyse of the process of educating suppliers to create value for customers by streamlining processes in the value chain<br>» Analyse the difference between strategic and non-strategic suppliers and the associated supplier management principles<br>» Analyse commercial factors that contribute toward cost reduction, price savings and value improvement opportunities

» Ensure the strategic sourcing practices drive down cost across consecutive tenders

» Analyse the process of working with suppliers to agree cost saving opportunities in the whole value chain, with an appropriate cost sharing mechanism applied to ensure a "win-win" outcome

» Evaluate the process of managing supplier risk

» Demonstrate ethical sourcing

Personal/Management Competency(ies):	5.5 Manage and plan projects<br>5.5.2 Execute strategic decision making<br>5.5.3 Manage partnerships<br>6.1 Demonstrate generic skills (e.g., literacy, numeracy, technology)<br>6.1.1 Exhibit high understanding of literacy and numeracy<br>6.2 Demonstrate strong communication skills<br>6.2.1 Practice cultural awareness<br>6.3 Utilize problem-solving skills<br>6.3.1 Negotiate

5.5 Manage and plan projects

5.5.2 Execute strategic decision making

» Determine how one can make decisions regarding one's own workload and area of responsibility

» Determine the process of prioritizing competing tasks and perform them quickly and efficiently according to their importance; finding new ways of organizing or planning work to accomplish it more efficiently

» Evaluate the proper time and space for consultation around decisions being made.

» Demonstrate the process of considering the input of resources including staff needed

» Analyse assumptions against facts

» Determine the key issues in a complex situation and come to the heart of the problem quickly

» Evaluate relevant information before making decisions

» Provide input to strategic decisions in the interest of the organisation's goals

» Evaluate when to analyse, evaluate and execute tough decisions when necessary

» Analyse the impact of decisions on others' work or team goals

» Communicate decisions and ensure they are incorporated into policies and processes

» Demonstrate inclusive and consultative strategic planning to establish strategic objectives

» Evaluate emerging trends and practices

» Analyse the macro and long-term consequences of decisions

5.5.3 Manage partnerships	» Demonstrate the concepts of partnership working<br>» Determine the process of supporting implementation of partnership programmes<br>» Analyse the process of experimenting with and trialling new ideas with partners<br>» Analyse partnerships to deliver programme and increase impact<br>» Analyse innovation and creativity in partnership work<br>» Communicate key information with partner members<br>» Involve and value partners in all aspects of programming<br>» Analyse problems with supply chain relationships<br>» Demonstrate the process of communicating effectively with nurses, doctors and other members of the healthcare team<br>» Analyse the process of responding to consumer complaints or comments about services and/or advice received<br>» Analyse collaboration and consultation across the sector and encourage the team to work with others to gain market insight<br>» Encourage input from other key actors in the humanitarian sector and develop relationships with a cross-section of actors<br>» Analyse the process of meeting the reporting requirements of vertical programmes<br>» Analyse the structure of the health system at a national level and explain this to others<br>» Analyse the roles of other members of the healthcare team (including with consumers) in a way that engenders understanding and confidence in the team and its members<br>» Analyse the structure of the organisation, environment and/or service in which they work<br>» Demonstrate how vertical programmes work within the health system<br>» Evaluate the communication network established to achieve work outcomes<br>» Demonstrate knowledge of the structure of the health system at the provincial/regional level and explain this to others<br>» Analyse the roles and functions of government agencies in regulating and supporting supply chain organisations<br>» Demonstrate knowledge of change management (e.g. PtD Theory of Change)
6.1 Demonstrate generic skills (e.g., literacy, numeracy, technology)	
6.1.1 Exhibit high understanding of literacy and numeracy	» Encourage speaking clearly and confidently, organizing information in a logical manner, and considering voice tone and pace

- Demonstrate responding to verbal messages and other cues (active listening)
- Demonstrate expressing ideas and opinions clearly in written and verbal form
- Demonstrate communicating information accurately, concisely and confidently in writing and verbally
- Demonstrate a level of mathematics suitable to the job held
- Demonstrate knowledge of visual presentation techniques, including charting, histograms and flow sheets
- Demonstrate reading and comprehending at a level necessary to properly complete duties of the position
- Demonstrate the process of interpreting written information in documents, such as reports, SOPs, LMIS forms, graphs, calendars, schedules, notices and directions
- Demonstrate paying attention to detail and identifying the main ideas, detecting inconsistencies and identifying missing information in documents
- Demonstrate computer skills — such as using Word, Excel, PowerPoint and the internet — suitable to the job level
- Demonstrate the process of analysing and validating KPIs
- Participate in the evaluation of activities that act as standards against which the KPIs are measured
- Demonstrate the process of performing critical self-review on work before submission

6.2 Demonstrate strong communication skills

- Demonstrate being truthful and trustworthy, and supplying accurate information at all times
- Demonstrate working as part of a workplace team
- Determine vocabulary and communication style and form for both written and verbal communication that is appropriate for the situation, audience and material being communicated (e.g., avoid unnecessary jargon, clearly explain medical and SCM terminology)
- Demonstrate open communication within constraints of confidentiality
- Analyse communications about logistics
- Demonstrate participation in meetings, expressing one’s opinions, being aware of others’ needs, and being appropriately assertive when required
- Demonstrate perspective into the point of view of others, understanding their needs and goals
- Demonstrate the practise of building trust, rapport and credibility with others
- Determine the communication needs and concerns of others and respond to them
- Demonstrate avoiding conflict between work and personal interests
- Demonstrate the practice of meaningful two-way communication
- Demonstrate influence on others by persuasively presenting thoughts and ideas

- Demonstrate assertiveness skills to deal with unreasonable requests and/or refusals that would compromise practice or consumer care
- Demonstrate a positive, supportive and appreciative attitude
- Demonstrate the ability to balance the organisation's needs and the customer's needs
- Demonstrate self-control by maintaining composure and keeping emotions in check, even in difficult situations; deal calmly and effectively with stressful situations
- Demonstrate the expressing of opinions and providing information in written and/or verbal form in a manner that does not elicit concern, anger or other adverse response
- Demonstrate the use of a systematic process for following up that demonstrates written reports have been received and understood
- Evaluate the means by which responses to input to the work environment are monitored
- Seek practical ways to overcome barriers to communication
- Ensure language representation (e.g., interpreters) at meetings when appropriate
- Demonstrate tackling difficult situations and resolving disputes between staff
- Demonstrate giving both positive and negative feedback sensitively
- Demonstrate maintaining and improving communication
- Demonstrate the process of giving and requesting feedback frequently
- Demonstrate the ability to communicate effectively with shop stewards and union representatives

6.2.1 Practice cultural awareness

- Demonstrate cultural awareness and sensitivity, treating all people with fairness, respect and dignity
- Demonstrate anti-discriminatory practices in the organisation, including HR and disciplinary procedures
- Demonstrate cultural sensitivity, equality and fairness at all levels of the organisation
- Demonstrate challenging discriminatory behaviour directly and sensitively
- Demonstrate acting in a non-discriminatory way toward individuals and groups
- Demonstrate the process of integrating cultural awareness in learning and development approaches
- Demonstrate avoiding stereotypical responses by examining one's own behaviour and bias
- Demonstrate managing cultural diversity in teams and make the most of differences
- Demonstrate the use of strategies and/or resources for communicating effectively with people from different cultural backgrounds
- Supply information relating to values, beliefs and cultural backgrounds of consumers that may influence the way professional services are provided

6.3 Utilize problem-solving skills	» Evaluate information to solve problems in a sensitive and ethical manner<br>» Demonstrate managing day to day and complex problems in a timely manner<br>» Evaluate problem situations and their step-by-step transformation based on planning and reasoning, without apportioning blame<br>» Demonstrate the process of critically reviewing, analysing, synthesizing, comparing and interpreting information; drawing conclusions from relevant and/or missing information; and understanding the relationship among facts and apply this understanding when solving problems<br>» Evaluate a range of possible approaches/strategies that are effective for resolving conflict in the workplace (e.g., negotiation, collaborative problem-solving, mediation, arbitration)<br>» Evaluate an open environment that encourages people to work together<br>» Demonstrate resolving conflicts as they arise<br>» Demonstrate creativity through questioning, attempting to improve on ideas, applying other experiences and working toward action in problem solving<br>» Analyse facts and thoughts into strengths and weaknesses<br>» Demonstrate thinking in a careful way to solve problems, analyse data, and recall and apply information, involving others<br>» Demonstrate goal-directed thinking and action in situations in which no routine solutions exist<br>» Demonstrate maintaining appropriate ethical and moral standards in resolving problems<br>» Analyse alternative courses of action, using cognitive processes such as memory, thinking and evaluation<br>» Demonstrate mapping likely consequences of decisions to choose the best course of action<br>» Demonstrate asking other people to help with solving problems<br>» Evaluate difficult or unusual situations to develop unique approaches and useful solutions<br>» Demonstrate committing to a solution in a timely manner, and developing a realistic approach for applying the chosen solution; evaluating the outcome of the solution to see if further action is needed, and identify lessons learned<br>» Evaluate the impact of conflict in the workplace (e.g., tension, low morale, absenteeism, system or service failure, aggressive or uncooperative behaviours)<br>» Demonstrate the practise of following up on problems to ensure they are fixed<br>» Determine situations where referral is warranted (e.g., severe emotional distress, intractable dispute)
6.3.1 Negotiate	» Demonstrate the principles of negotiation and aim for a win-win outcome<br>» Demonstrating managing and resolving relationship issues within and outside the team

	» Determine solution-focussed approaches to further the organisation's mission<br>» Demonstrate building consensus among parties<br>» Evaluate alternative ways of doing things<br>» Determine where fair approaches are being utilised to resolve issues<br>» Demonstrate seeking to reach constructive solutions while maintaining positive working relationships<br>» Demonstrate adapting style to take into account cultural differences regarding negotiation<br>» Demonstrate building consensus at a high level for the benefit of all parties
Basal Technology Competency(ies):	7.4 Temperature and monitoring<br>7.6 ERP includes function of LMIS<br>7.12 Basic Office Skills<br>7.13 Have a command of technology
7.4 Temperature and monitoring	» Analyse the current technology approaches for Temperature monitoring and sensors<br>» Evaluate what is required to implement this technology in a country context<br>» Evaluate infrastructure requirement for heat-sensitive items at peripheral 'lower level' of SC depending on volume and nature of items to handle;<br>» Analyse regular preventive maintenance and calibration of cold-chain equipment with biomedical engineers or logisticians<br>» Evaluate the policy for temperature monitoring tools during transportation and storage<br>» Demonstrate the steps involved in health logistic management cycle: selection-order-delivery-reception-storage-distribution-inventory<br>» Determine hardware qualification and system validation of temperature monitoring<br>» Evaluate mass data management of temperature monitoring
7.6 ERP includes function of LMIS	» Evaluate a Master Data Management System<br>» Demonstrate an understanding of the critical components of an ERP system<br>» Demonstrate an understanding of the meaning of common document types found within an ERP/LMIS<br>» Demonstrate an understanding of the logic of data visibility within an ERP<br>» Demonstrate the importance of data integrity, record management and information security
7.12 Basic Office Skills	» Demonstrate a good understanding of common presentation authoring packages<br>» Demonstrate a good understanding of common spreadsheet authoring packages

	» Demonstrate a good understanding of common document authoring packages<br>» Demonstrate a good understanding of common email authoring packages<br>» Demonstrate a good understanding of common instant messaging packages<br>» Demonstrate a good understanding of common video conferencing authoring packages
7.13 Have a command of technology	» Demonstrate the use of technology suitable to the job held<br>» Monitor new developments and technologies in the sector<br>» Experiment with new technologies and recognising potential benefits for the sector<br>» Ensure that resources and support are provided across the organisation to enable colleagues to make the best use of available technology<br>» Determine what the organisations' strategy is for technology use<br>» Determine how to minimise environmental damage through technology use<br>» Analyse the use of technology to maximise effectiveness and efficiency<br>» Analyse the use of field-based technology (e.g., radio, general packet radio service (GPRS), satellite phone)

Key Performance Indicators:

- Adherence to medicines budget
- Cost savings delivery
- Medicine availability
- Adherence to strategic sourcing principles for different categories

Training:	Qualifications Available:	Certifications Available:
» Contract Management<br>» Analytical Skills<br>» Competitive Billing<br>» Negotiation	» Bachelor of Business Science<br>» Bachelor of Accounting Honours	» Occupational Certificate: Supply and Distribution Manager<br>» SPSM3 TM (Enterprise-Wide Procurement Influence)<br>» CIPS Level 5 Advanced Diploma in Procurement and Supply

Lead – Receiving and Inventory Management

Organisation:<br>**Public Health Organisation – Hospital Service Delivery**				
Competency Level:	Serial Number:	Primary Process:	Job Role:	Supervises:
Managerial	3-33-37-4	Deliver	Lead – Receiving and Inventory Management	Officer – Receiving and Inventory Management, Clerk – Receiving and Storage
Contributions	Manages the receiving team in the context department, manages the warehouse personnel to perform according to best practices as defined by the Supply Chain model of the Public Health Supply Chain. Monitors the order cycle metrics for receiving and put away, inventory put away accuracy.			
Technical Competency(ies):	2.5 Ensure quality of products<br>3.1 Undertake storage, warehousing and inventory management<br>3.1.1 Manage storage of commodities during emergency<br>3.3 Supply commodities to sections within a facility<br>3.5 Manage disposal of products (e.g. expired, damaged, redundant products)<br>3.5.1 Define and direct process for managing redundant and returned stock<br>3.7 Manage re-packing of products			

2.5 Ensure quality of products	» Analyse the use and monitoring process for prequalification of suppliers<br>» Evaluate the process of ensuring that health commodities are not counterfeit and meet quality standards through certification (ISO, WHO GMP<br>» Implement, conduct and maintain a reporting system of pharmacovigilance (e.g., report adverse drug reactions, quality defects, batch recall) and equipment faults<br>» Analyse the process of inspecting products when delivered and during storage to catch defects or problems before they are given to, or needed by, clients<br>» Demonstrate compliance with good distribution and good storage practices (temperature monitoring in the warehouse, transportation and distribution)<br>» Evaluate product-specific shelf-life by which the product are acceptable or rejected (e.g. no less than 1 year for most of drugs, and less for short shelf-life like laboratory)
3.1 Undertake storage, warehousing and inventory management	» Analyse methods to distribute products among suppliers, distribution centres, warehouses and customers through a logistics network<br>» Analyse a total systems approach to designing and managing the entire flow of information, materials, and services<br>» Determine the choice of equipment or materials based on suitability for intended use, accuracy, safety of use and cost

	» Analyse the warehouse configuration to have formal storage locations that identify the row, rack section, level and shelf location, typically with an alphanumeric location bar code or label<br>» Evaluate how medicines are appropriately stored, considering temperature, access and cleanliness<br>» Determine the appropriate use of, and ability to maintain, the cold chain<br>» Analyse the movement and storage of materials within a warehouse<br>» Analyse the process of securing the medical store and limiting access to staff<br>» Analyse organisation processes, instructions, rules and parameters for warehouse and inventory management and use of equipment (e.g., annual stock turns, expiry date tracking, stock procurement, rotation and retrieval, equipment maintenance)<br>» Analyse policies and procedures for stock handling, distribution and withdrawal consistent with maintaining safety (e.g., policies for distribution of concentrated electrolyte solutions and cytotoxics, identification of cytotoxic drug products within the work environment, separation of like-named or like-packaged products)<br>» Analyse requirements for information on the supplies being stored<br>» Analyse processes to consolidate several items into larger units for fewer handlings<br>» Analyse methods of stock rotation (e.g., first in first out, FIFO, or first to expire first out, FEFO)<br>» Analyse the process of entering the quantity received and updating the warehouse management system software with the stock on hand when each order is delivered<br>» Determine required levels of stock and/or equipment in specific situations<br>» Measure the accuracy of verification of rolling stocks<br>» Implement the methodology for monitoring the location and condition of the supplies being stored<br>» Analyse information on the storage locations and facilities<br>» Analyse any problems in using the storage locations and facilities
3.1.1 Manage storage of commodities during emergency	» Analyse the different types of warehouses available for emergency or disaster supply<br>» Analyse the choice of storage site for emergency or disaster supply<br>» Evaluate the staff required for the storage of emergency or disaster supply<br>» Determine the equipment and material required for the warehousing of emergency or disaster supply<br>» Conduct a rapid assessment of logistical needs in emergencies
3.3 Supply commodities to sections within a facility	» Review imprest list and their implementations in the context<br>» Analyse and validate the requisition from different health care services within the facility
3.5 Manage disposal of products (e.g. expired,	»

damaged, redundant products)	
3.5.1 Define and direct process for managing redundant and returned stock	» Evaluate information on the supplies being returned<br>» Collate, prepare and analyse data on the flow of returned supplies<br>» Resolve any problems with the flow of returned supplies<br>» Recommend options for improving the flow of returned supplies<br>» Analyse procedures for monitoring the flow of returned supplies<br>» Determine recall procedures to be used in response to a product recall notice or to access the information promptly.<br>» Demonstrate knowledge of issues relevant to the urgency and scope of action required in response to a product recall notice<br>» Analyse the information provided in the reports when following up on medication incidents<br>» Evaluate follow-up strategies likely to be effective in preventing recurrence (e.g., root cause analysis)<br>» Evaluate an integrated healthcare waste management plan<br>» Evaluate guides and procedures for collection, sorting, transport and disposal<br>» Evaluate pre-disposal inspections<br>» Evaluate the prerequisites for products disposal<br>» Evaluate product disposal report
3.7 Manage re-packing of products	» Determine when re-packing or pre-packs are required<br>» Determine a safe system for repacking
Personal/Management Competency(ies):	5.5 Manage and plan projects<br>5.5.2 Execute strategic decision making<br>5.5.3 Manage partnerships<br>6.1 Demonstrate generic skills (e.g., literacy, numeracy, technology)<br>6.1.1 Exhibit high understanding of literacy and numeracy<br>6.2 Demonstrate strong communication skills<br>6.2.1 Practice cultural awareness
5.5 Manage and plan projects	
5.5.2 Execute strategic decision making	» Determine how one can make decisions regarding one's own workload and area of responsibility<br>» Determine the process of prioritizing competing tasks and perform them quickly and efficiently according to their importance; finding new ways of organizing or planning work to accomplish it more efficiently<br>» Evaluate the proper time and space for consultation around decisions being made.<br>» Demonstrate the process of considering the input of resources including staff needed<br>» Analyse assumptions against facts

	» Determine the key issues in a complex situation and come to the heart of the problem quickly<br>» Evaluate relevant information before making decisions<br>» Provide input to strategic decisions in the interest of the organisation's goals<br>» Evaluate when to analyse, evaluate and execute tough decisions when necessary<br>» Analyse the impact of decisions on others' work or team goals<br>» Communicate decisions and ensure they are incorporated into policies and processes<br>» Demonstrate inclusive and consultative strategic planning to establish strategic objectives<br>» Evaluate emerging trends and practices<br>» Analyse the macro and long-term consequences of decisions
5.5.3 Manage partnerships	» Demonstrate the concepts of partnership working<br>» Determine the process of supporting implementation of partnership programmes<br>» Analyse the process of experimenting with and trialling new ideas with partners<br>» Analyse partnerships to deliver programme and increase impact<br>» Analyse innovation and creativity in partnership work<br>» Communicate key information with partner members<br>» Involve and value partners in all aspects of programming<br>» Analyse problems with supply chain relationships<br>» Demonstrate the process of communicating effectively with nurses, doctors and other members of the healthcare team<br>» Analyse the process of responding to consumer complaints or comments about services and/or advice received<br>» Analyse collaboration and consultation across the sector and encourage the team to work with others to gain market insight<br>» Encourage input from other key actors in the humanitarian sector and develop relationships with a cross-section of actors<br>» Analyse the process of meeting the reporting requirements of vertical programmes<br>» Analyse the structure of the health system at a national level and explain this to others<br>» Analyse the roles of other members of the healthcare team (including with consumers) in a way that engenders understanding and confidence in the team and its members<br>» Analyse the structure of the organisation, environment and/or service in which they work<br>» Demonstrate how vertical programmes work within the health system<br>» Evaluate the communication network established to achieve work outcomes

	» Demonstrate knowledge of the structure of the health system at the provincial/regional level and explain this to others<br>» Analyse the roles and functions of government agencies in regulating and supporting supply chain organisations<br>» Demonstrate knowledge of change management (e.g. PtD Theory of Change)
6.1 Demonstrate generic skills (e.g., literacy, numeracy, technology)	
6.1.1 Exhibit high understanding of literacy and numeracy	» Encourage speaking clearly and confidently, organizing information in a logical manner, and considering voice tone and pace<br>» Demonstrate responding to verbal messages and other cues (active listening)<br>» Demonstrate expressing ideas and opinions clearly in written and verbal form<br>» Demonstrate communicating information accurately, concisely and confidently in writing and verbally<br>» Demonstrate a level of mathematics suitable to the job held<br>» Demonstrate knowledge of visual presentation techniques, including charting, histograms and flow sheets<br>» Demonstrate reading and comprehending at a level necessary to properly complete duties of the position<br>» Demonstrate the process of interpreting written information in documents, such as reports, SOPs, LMIS forms, graphs, calendars, schedules, notices and directions<br>» Demonstrate paying attention to detail and identifying the main ideas, detecting inconsistencies and identifying missing information in documents<br>» Demonstrate computer skills — such as using Word, Excel, PowerPoint and the internet — suitable to the job level<br>» Demonstrate the process of analysing and validating KPIs<br>» Participate in the evaluation of activities that act as standards against which the KPIs are measured<br>» Demonstrate the process of performing critical self-review on work before submission
6.2 Demonstrate strong communication skills	» Demonstrate being truthful and trustworthy, and supplying accurate information at all times<br>» Demonstrate working as part of a workplace team<br>» Determine vocabulary and communication style and form for both written and verbal communication that is appropriate for the situation, audience and material being communicated (e.g., avoid unnecessary jargon, clearly explain medical and SCM terminology)<br>» Demonstrate open communication within constraints of confidentiality<br>» Analyse communications about logistics

- Demonstrate participation in meetings, expressing one's opinions, being aware of others' needs, and being appropriately assertive when required
- Demonstrate perspective into the point of view of others, understanding their needs and goals
- Demonstrate the practise of building trust, rapport and credibility with others
- Determine the communication needs and concerns of others and respond to them
- Demonstrate avoiding conflict between work and personal interests
- Demonstrate the practice of meaningful two-way communication
- Demonstrate influence on others by persuasively presenting thoughts and ideas
- Demonstrate assertiveness skills to deal with unreasonable requests and/or refusals that would compromise practice or consumer care
- Demonstrate a positive, supportive and appreciative attitude
- Demonstrate the ability to balance the organisation's needs and the customer's needs
- Demonstrate self-control by maintaining composure and keeping emotions in check, even in difficult situations; deal calmly and effectively with stressful situations
- Demonstrate the expressing of opinions and providing information in written and/or verbal form in a manner that does not elicit concern, anger or other adverse response
- Demonstrate the use of a systematic process for following up that demonstrates written reports have been received and understood
- Evaluate the means by which responses to input to the work environment are monitored
- Seek practical ways to overcome barriers to communication
- Ensure language representation (e.g., interpreters) at meetings when appropriate
- Demonstrate tackling difficult situations and resolving disputes between staff
- Demonstrate giving both positive and negative feedback sensitively
- Demonstrate maintaining and improving communication
- Demonstrate the process of giving and requesting feedback frequently
- Demonstrate the ability to communicate effectively with shop stewards and union representatives

6.2.1 Practice cultural awareness

- Demonstrate cultural awareness and sensitivity, treating all people with fairness, respect and dignity
- Demonstrate anti-discriminatory practices in the organisation, including HR and disciplinary procedures
- Demonstrate cultural sensitivity, equality and fairness at all levels of the organisation

	» Demonstrate challenging discriminatory behaviour directly and sensitively<br>» Demonstrate acting in a non-discriminatory way toward individuals and groups<br>» Demonstrate the process of integrating cultural awareness in learning and development approaches<br>» Demonstrate avoiding stereotypical responses by examining one's own behaviour and bias<br>» Demonstrate managing cultural diversity in teams and make the most of differences<br>» Demonstrate the use of strategies and/or resources for communicating effectively with people from different cultural backgrounds<br>» Supply information relating to values, beliefs and cultural backgrounds of consumers that may influence the way professional services are provided
Basal Technology Competency(ies):	7.4 Temperature and monitoring<br>7.6 ERP includes function of LMIS<br>7.12 Basic Office Skills<br>7.13 Have a command of technology
7.4 Temperature and monitoring	» Analyse the current technology approaches for Temperature monitoring and sensors<br>» Evaluate what is required to implement this technology in a country context<br>» Evaluate infrastructure requirement for heat-sensitive items at peripheral 'lower level' of SC depending on volume and nature of items to handle;<br>» Analyse regular preventive maintenance and calibration of cold-chain equipment with biomedical engineers or logisticians<br>» Evaluate the policy for temperature monitoring tools during transportation and storage<br>» Demonstrate the steps involved in health logistic management cycle: selection-order-delivery-reception-storage-distribution-inventory<br>» Determine hardware qualification and system validation of temperature monitoring<br>» Evaluate mass data management of temperature monitoring
7.6 ERP includes function of LMIS	» Evaluate a Master Data Management System<br>» Demonstrate an understanding of the critical components of an ERP system<br>» Demonstrate an understanding of the meaning of common document types found within an ERP/LMIS<br>» Demonstrate an understanding of the logic of data visibility within an ERP

	» Demonstrate the importance of data integrity, record management and information security
7.12 Basic Office Skills	» Demonstrate a good understanding of common presentation authoring packages<br>» Demonstrate a good understanding of common spreadsheet authoring packages<br>» Demonstrate a good understanding of common document authoring packages<br>» Demonstrate a good understanding of common email authoring packages<br>» Demonstrate a good understanding of common instant messaging packages<br>» Demonstrate a good understanding of common video conferencing authoring packages
7.13 Have a command of technology	» Demonstrate the use of technology suitable to the job held<br>» Monitor new developments and technologies in the sector<br>» Experiment with new technologies and recognising potential benefits for the sector<br>» Ensure that resources and support are provided across the organisation to enable colleagues to make the best use of available technology<br>» Determine what the organisations' strategy is for technology use<br>» Determine how to minimise environmental damage through technology use<br>» Analyse the use of technology to maximise effectiveness and efficiency<br>» Analyse the use of field-based technology (e.g., radio, general packet radio service (GPRS), satellite phone)
Advanced Technology Competency(ies):	7.7 Automation
7.7 Automation	» Evaluate automation as a value-add capability for relevant global health use cases

Key Performance Indicators:

- Availability - Stock out rate - Number of missed line item quantity deliveries due to stock out versus total line item quantity deliveries
- Availability - Stock levels - Current on hand stock versus Planned on hand stock
- Safe product handling - Number of products handled during period out of conformance versus Number of products handled during period expressed as a % with prior period comparisons
- % Product Transferred On-Time to Demand Requirement. Actual Number of products transferred outside of the Time Limits vs Number of products Transferred.
- Transfer Product Cycle Time - Actual Transfer Cycle Time versus Planned Transferred Cycle Time
- Accurate pick - Picking Accuracy number of order lines picked inaccurately divided by the total order lines picked (SKU and Quantity)
- On-time delivery - Actual Delivery Date & Time Vs Promised Delivery Data and Time
- Customer order fulfilment cycle time & rate - Actual Order Fulfilment Cycle Time versus Promised Customer Order Fulfilment Cycle time
- Customer Incidents Report number of incidents by month/week/day comparison with previous periods
- No of Order Lines shipped with incorrectly captured Items
- Number of order lines with incorrect items/SKU's versus total number of order lines shipped as a %
- Regulatory - % of procured products that meet Stringent Regulatory Authority (SRA) or WHO standards
- Warehousing and Inventory Management OTIF - On time and in full
- Waste Management - Existence of supply chain management protocols for disposal of medical waste and management of unusable products
- Number of Order Lines delivered to incorrect end-user
- Number of orders incorrectly addressed versus number of orders processed expressed as a %
- Number of Order Lines delivered out of Quality Specifications - Number of Item/SKU delivered out of specification e.g. Cold Chain infractions, Expired products versus total Number of order lines delivered as a %. A comparative graph would be an appropriate tool for the measurement of this metric.
- Warehouse Utilization - Volume used vs Volume Available
- Line Items SKU's handled vs Items / SKU's Planned over a planning horizon
- Number of warehouse locations used versus warehouse locations available (Pallet Positions may be used in larger warehouses)
- Warehouse Utilization - Volume used vs Volume Available

Training:	Qualifications Available:	Certifications Available:
» Data management<br>» ERP Systems<br>» Legislation and Standards<br>» Supplier Relationship Management (SRM)<br>» 3-way Receiving Match<br>» Code Handling/RFID (if available)	» Bachelor of Commerce in Logistics Management<br>» Bachelor of Business Administration in Logistics and Supply Chain Management<br>» Bachelor of Business Management	» Occupational Certificate: Health Information Manager<br>» APICS Certified in logistics transportation and distribution certificate<br>» SPSM Level 4 (External Procurement Influence)

- Cross Docking
- Discrepancy Reporting and Resolution
- ID & Damage Inspection
- Inventory Management
- MSDS/Environmental Interpretation
- Property Control and Disposition
- Return Management
- Pharmacovigilance

Lead – Returns and Disposal

Organisation:

Public Health Organisation – Hospital Service Delivery

Competency Level:	Serial Number:	Primary Process:	Job Role:	Supervises:
**Managerial**	**3-351-352-3**	**Return**	**Lead – Returns and Disposal**	**-**

Contributions	Ensures regulatory compliance of returns. Manages the quarantine requirements for material returns and disposition of return materials. Ensures that the material being returned is accounted for at all times.
Technical Competency(ies):	3.5 Manage disposal of products (e.g. expired, damaged, redundant products)<br>3.5.1 Define and direct process for managing redundant and returned stock<br>3.5.2 Manage process for disposal of returned stock

3.5 Manage disposal of products (e.g. expired, damaged, redundant products)

3.5.1 Define and direct process for managing redundant and returned stock

- Confirm information on the supplies being returned
- Describe the flow of returned supplies
- Identify any problems with the flow of returned supplies
- Integrate options for improving the flow of returned supplies
- Apply procedures for monitoring the flow of returned supplies
- Describe and/or demonstrate recall procedures to be used in response to a product recall notice or to access the information promptly.
- Identify issues relevant to the urgency and scope of action required in response to a product recall notice
- Identify the information required for reporting and procedures with regards to following up on medication incidents
- Identify follow-up strategies likely to be effective in preventing recurrence (e.g., root cause analysis)
- Describe an integrated healthcare waste management plan
- Describe guides and procedures for collection, sorting, transport and disposal
- Conduct pre-disposal inspections
- Describe the prerequisites for products disposal
- Generate product disposal report

3.5.2 Manage process for disposal of returned stock	» Dispose of expired medications and/or medical equipment according to national policy<br>» Describe and/or use an appropriate recording system for disposal<br>» Describe the disposal of specific individual items of greatest risk with appropriate care (e.g., oncology medicine)
Personal/Management Competency(ies):	6.1 Demonstrate generic skills (e.g., literacy, numeracy, technology)<br>6.1.1 Exhibit high understanding of literacy and numeracy<br>6.2 Demonstrate strong communication skills<br>6.2.1 Practice cultural awareness
6.1 Demonstrate generic skills (e.g., literacy, numeracy, technology)	
6.1.1 Exhibit high understanding of literacy and numeracy	» Speak clearly and confidently, organize information in a logical manner, and consider voice tone and pace<br>» Respond to verbal messages and other cues (active listening)<br>» Express ideas and opinions clearly in written and verbal form<br>» Communicate information accurately, concisely and confidently in writing and verbally<br>» Practice a level of mathematics suitable to the job held<br>» Describe visual presentation techniques, including charting, histograms and flow sheets<br>» Read and comprehend at a level necessary to properly complete duties of the position<br>» Interpret written information in documents, such as reports, SOPs, LMIS forms, graphs, calendars, schedules, notices and directions<br>» Pay attention to detail and identify the main ideas, detect inconsistencies and identify missing information in documents<br>» Apply computer skills — such as using Word, Excel, PowerPoint and the internet — suitable to the job level<br>» Describe the process of analysing and validating KPIs<br>» Participate in the evaluation of activities that act as standards against which the KPIs are measured<br>» Apply the process of performing critical self-review on work before submission
6.2 Demonstrate strong communication skills	» Be truthful and trustworthy, and supply accurate information at all times<br>» Work as part of a workplace team<br>» Describe vocabulary and communication style and form for both written and verbal communication that is appropriate for the situation, audience and material being communicated (e.g., avoid unnecessary jargon, clearly explain medical and SCM terminology)<br>» Encourage open communication within constraints of confidentiality<br>» Organize communications about logistics

	» Encourage participation in meetings, expressing one's opinions, being aware of others' needs, and being appropriately assertive when required<br>» Apply perspective into the point of view of others, understanding their needs and goals<br>» Encourage building trust, rapport and credibility with others<br>» Anticipate the communication needs and concerns of others and respond to them<br>» Avoid conflict between work and personal interests<br>» Encourage meaningful two-way communication<br>» Influence others by persuasively presenting thoughts and ideas<br>» Apply assertiveness skills to deal with unreasonable requests and/or refusals that would compromise practice or consumer care<br>» Maintain a positive, supportive and appreciative attitude<br>» Apply the ability to balance the organisation's needs and the customer's needs<br>» Use self-control by maintaining composure and keeping emotions in check, even in difficult situations; deal calmly and effectively with stressful situations<br>» Encourage the expressing of opinions and providing information in written and/or verbal form in a manner that does not elicit concern, anger or other adverse response<br>» Apply the use of a systematic process for following up that demonstrates written reports have been received and understood<br>» Describe the means by which responses to input to the work environment are monitored<br>» Seek practical ways to overcome barriers to communication<br>» Identify language representation (e.g., interpreters) at meetings when appropriate<br>» Tackle difficult situations and resolve disputes between staff<br>» Give both positive and negative feedback sensitively<br>» Maintain and improve communication<br>» Apply the process of giving and requesting feedback frequently<br>» Apply the ability to communicate effectively with shop stewards and union representatives
6.2.1 Practice cultural awareness	» Describe cultural awareness and sensitivity, treating all people with fairness, respect and dignity<br>» Describe anti-discriminatory practices in the organisation, including HR and disciplinary procedures<br>» Establish cultural sensitivity, equality and fairness at all levels of the organisation<br>» Challenge discriminatory behaviour directly and sensitively<br>» Act in a non-discriminatory way toward individuals and groups<br>» Integrate cultural awareness in learning and development approaches

	» Avoid stereotypical responses by examining one's own behaviour and bias<br>» Manage cultural diversity in teams and make the most of differences<br>» Describe strategies and/or resources for communicating effectively with people from different cultural backgrounds<br>» Elicit information relating to values, beliefs and cultural backgrounds of consumers that may influence the way professional services are provided
Basal Technology Competency(ies):	7.6 ERP includes function of LMIS<br>7.12 Basic Office Skills<br>7.13 Have a command of technology
7.6 ERP includes function of LMIS	» Support a Master Data Management System<br>» Describe the critical components of an ERP system<br>» Describe the meaning of common document types found within an ERP/LMIS<br>» Describe the logic of data visibility within an ERP<br>» Describe the importance of data integrity, record management and information security
7.12 Basic Office Skills	» Have a good understanding of common presentation authoring packages<br>» Have a good understanding of common spreadsheet authoring packages<br>» Have a good understanding of common document authoring packages<br>» Have a good understanding of common email authoring packages<br>» Have a good understanding of common instant messaging packages<br>» Have a good understanding of common video conferencing authoring packages
7.13 Have a command of technology	» Apply the use of technology suitable to the job held<br>» Provide input on monitoring new developments and technologies in the sector<br>» Identify new technologies and recognising potential benefits for the sector<br>» Identify resources and support that are provided across the organisation to enable colleagues to make the best use of available technology<br>» Ensure that the organisation has a strategy for technology use<br>» Describe how to minimise environmental damage through technology use<br>» Use technology to maximise effectiveness and efficiency

» Use field-based technology (e.g., radio, general packet radio service (GPRS), satellite phone)

Advanced Technology Competency(ies):	7.7 Automation
7.7 Automation	» Describe automation as a value-add capability for relevant global health use cases

Key Performance Indicators:

- » Availability - Stock out rate - Number of missed line item quantity deliveries due to stock out versus total line item quantity deliveries
- » Availability - Stock levels - Current on hand stock versus Planned on hand stock
- » Safe product handling - Number of products handled during period out of conformance versus Number of products handled during period expressed as a % with prior period comparisons
- » Customer Incidents Report number of incidents by month/week/day comparison with previous periods
- » No of Order Lines shipped with incorrectly captured Items - Number of order lines with incorrect items/SKU's versus total number of order lines shipped as a %
- » Waste Management - Existence of supply chain management protocols for disposal of medical waste and management of unusable products
- » Number of Order Lines delivered to incorrect end-user - Number of orders incorrectly addressed versus number of orders processed expressed as a %
- » Number of Order Lines delivered out of Quality Specifications - Number of Item/SKU delivered out of specification e.g. Cold Chain infractions, Expired products versus total Number of order lines delivered as a %. A comparative graph would be an appropriate tool for the measurement of this metric.
- » % Product Transferred On-Time to Demand Requirement. Actual Number of products transferred outside of the Time Limits vs Number of products Transferred.
- » Transfer Product Cycle Time - Actual Transfer Cycle Time versus Planned Transferred Cycle Time
- » Accurate pick - Picking Accuracy number of order lines picked inaccurately divided by the total order lines picked (SKU and Quantity)
- » On-time delivery - Actual Delivery Date & Time Vs Promised Delivery Data and Time
- » Customer order fulfilment cycle time & rate - Actual Order Fulfilment Cycle Time versus Promised Customer Order Fulfilment Cycle time
- » Warehousing and Inventory Management OTIF - On time and in full
- » Regulatory - % of procured products that meet Stringent Regulatory Authority (SRA) or WHO standards

Training:	Qualifications Available:	Certifications Available:
» ERP Systems<br>» Inventory Management<br>» Return Management<br>» Regulatory Policy Management<br>» Logistics Management	» Bachelor of Business Administration in Logistics and Supply Chain Management<br>» Bachelor of Commerce in Logistics Management	» Occupational Certificate: Maintenance Planner

Operational
Officer – Demand and Supply Planning

Organisation:				
**Public Health Organisation – Hospital Service Delivery**				
Competency Level:	Serial Number:	Primary Process:	Job Role:	Supervises:
Operational	1-11-12-2	Plan – Procurement	Officer – Demand and Supply Planning	-

Contributions	Executes the Procurement process as prescribed in the Public Health System Organisation Operating Model as it relates to the context.
Technical Competency(ies):	1.1 Select the appropriate product<br>1.2 Define the specifications of the product, including product quality<br>1.3 List any special considerations for the product (e.g., temperature requirements, size, implications for infrastructure)<br>1.4 Forecast and quantify product needs<br>1.5 Supply planning

1.1 Select the appropriate product

» Compare the broad concepts of the national medicines policy, essential medicines lists, essential equipment lists, standard treatment guidelines and “dangerous drug” (DDA) policy

» Understand the government system required to add and subtract items from the essential medicines list and the essential equipment list

» Understand that the government system required to alter standard treatment guidelines, dangerous drug policy and national medicines policy

» Compare the type of supplies and services that are required

» Provide input for regularly scheduled coordination meetings with stakeholders involved in financing, procuring or distributing commodities

» Understand the steps needed to bring a medicinal product to the market, including the safety, quality, efficacy and pharmacoeconomic assessments of the product

1.2 Define the specifications of the product, including product quality

» Understand specifications for procurement

» Compare the characteristics of a good specification

» Compare the types of specifications

» Name the advantages of functional and performance specifications

» Examine the contents of a specification

» Name the procurement staff responsibilities in the specification process

» Compare the quality standard of product

» Not Applicable

1.3 List any special considerations for the product (e.g., temperature requirements, size, implications for infrastructure)	» Compare the principles and processes of category management, including market segmentation principles<br>» Examine any specific considerations in the quantification of programme-specific products (e.g., ARVs, family planning commodities, vaccines)<br>» Examine current international trends in commodity availability<br>» Compare regulatory requirements for the product
1.4 Forecast and quantify product needs	» Examine the factors that affect usage patterns of medications and equipment and how this affects ordering (e.g., disease outbreaks), using national policies as a guide and to ensure consistent application<br>» Understand principles and applications of demand forecasting<br>» Compare the critical requirements for effective forecasting: establishing time horizons, level of detail and use of data<br>» Understand policies and procedures for forecasting<br>» Understand the process of quantifying product requirements using a variety of methods<br>» Examine consumption data at the national level for various country programmes<br>» Compare and reconcile different types of forecasts<br>» Understand the process of applying VEN or ABC analysis to programme requirements for national level procurement<br>» Understand the importance of calculating average monthly dispensed-to-user quantities for all service delivery points nationally<br>» Understand the importance of calculating storage space requirements for all levels in the supply chain<br>» Understand the importance of calculating the months of supply on hand for each commodity at the national level<br>» Examine the maximum and minimum stock levels for each level in a programme<br>» Understand various tools used in quantification (e.g., quantification software)<br>» Understand the use of various data sources for commodity forecasting (e.g., consumption data, services data, demographic data)<br>» Compare key performance indicators of forecast accuracy<br>» Examine the degree of error when using forecasting methods
1.5 Supply planning	» Examine a purchasing and supply plan in line with national priorities<br>» Understand the importance of balancing of supply chain resources and trigger activities to correct any imbalances<br>» Understand the formalisation of sales and operations plans through sales and operations meeting.<br>» Understand that the sales and operations plan must be translated into executable plans to ensure dispatch of goods in line with overall priorities.<br>» Understand the national commodity pipeline
Personal/Management Competency(ies):	6.1 Demonstrate generic skills (e.g., literacy, numeracy, technology)<br>6.1.1 Exhibit high understanding of literacy and numeracy

6.1 Demonstrate generic skills (e.g., literacy, numeracy, technology)	
6.1.1 Exhibit high understanding of literacy and numeracy	» Speak clearly and confidently, organize information in a logical manner, and consider voice tone and pace<br>» Respond to verbal messages and other cues (active listening)<br>» Express ideas and opinions clearly in written and verbal form<br>» Communicate information accurately, concisely and confidently in writing and verbally<br>» Practice a level of mathematics suitable to the job held<br>» Compare visual presentation techniques, including charting, histograms and flow sheets<br>» Read and comprehend at a level necessary to properly complete duties of the position<br>» Understand the need to interpret written information in documents, such as reports, SOPs, LMIS forms, graphs, calendars, schedules, notices and directions<br>» Understand the need to pay attention to detail and identify the main ideas, detect inconsistencies and identify missing information in documents<br>» Enable computer skills — such as using Word, Excel, PowerPoint and the internet — suitable to the job level<br>» Understand the process of analysing and validating KPIs<br>» Understand how the evaluation process of activities that act as standards against which the KPIs are measured is carried out<br>» Examine the process of performing critical self-review on work before submission
Basal Technology Competency(ies):	7.6 ERP includes function of LMIS<br>7.12 Basic Office Skills<br>7.13 Have a command of technology
7.6 ERP includes function of LMIS	» Enable a Master Data Management System<br>» Understand the critical components of an ERP system<br>» Understand the meaning of common document types found within an ERP/LMIS<br>» Understand the logic of data visibility within an ERP<br>» Understand the importance of data integrity, record management and information security
7.12 Basic Office Skills	» Have a good understanding of common presentation authoring packages<br>» Have a good understanding of common spreadsheet authoring packages<br>» Have a good understanding of common document authoring packages<br>» Have a good understanding of common email authoring packages

	» Have a good understanding of common instant messaging packages<br>» Have a good understanding of common video conferencing authoring packages
7.13 Have a command of technology	» Enable the use of technology suitable to the job held<br>» Understand the need of monitoring new developments and technologies in the sector<br>» Compare new technologies and recognising potential benefits for the sector<br>» Understand that resources and support are provided across the organisation to enable colleagues to make the best use of available technology<br>» Understand that the organisation has a strategy for technology use<br>» Compare how to minimise environmental damage through technology use<br>» Enable the use of technology to maximise effectiveness and efficiency<br>» Enable the use of field-based technology (e.g., radio, general packet radio service (GPRS), satellite phone)
Advanced Technology Competency(ies):	7.7 Automation
7.7 Automation	» Understand automation as a value-add capability for relevant global health use cases

Key Performance Indicators:

- Vendor on time delivery (VOTD)
- % of emergency orders issued in the last 12 months
- % of product selection based on National Essential Medicines List
- % of procured products that meet Stringent Regulatory Authority (SRA) or WHO standards
- Reports submitted on time (for all and separate reports) in accordance with business rules (reporting calendar)
- Supply adequacy - Net requirements are reflected in the Procurement pipeline to secure sufficient future supply.
- Supply Plan report; supplier performance report
- Stock out rate - Number of missed line item quantity deliveries due to stock out versus total line item quantity deliveries
- Stock levels - Current on hand stock versus Planned on hand stock
- Forecasting and Supply Planning done within the specified cycle time to support the Supply Chain performance requirements.
- Data accuracy - Vendor Master data accuracy, item / SKU Master Data accuracy
- Establish Product Demand and Resources Cycle Time and recorded.in the "ERP" system against the products and resources.
- Establish Supply Plan Cycle Times and recorded in the "ERP" system.

- Fill Rate vs Planned fill rate
- Forecast accuracy, measured and reported on.
- Identify, Prioritize, and Aggregate Supply Chain Requirements Cycle Time - Actual Time taken to determine net supply chain requirements by product and SKU vs Planned Time
- Inventory Days of Supply - Actual inventory - Planned Demand over the planning horizon divided by the average daily usage
- Material and Resource Plan valid. Number of valid actions performed versus number of actions performed. (Exception messages trigger due to invalid schedules)
- Order accuracy - Net requirement versus order quantities in the planning horizon
- Procurement planning Cycle Time, by product and resource. Actual Overall planning cycle versus required Overall planning cycle time to achieve Supply Chain performance requirements.
- Ratio between median price of pro- ducts procured and the international median reference value
- Supply Chain Planning Cost recorded and measured against required Supply China Planning Cycle times to accomplish Supply Chain Strategic objectives.

Training:	Qualifications Available:	Certifications Available:
» Risk and exception management<br>» Import & export regulations for products<br>» Inventory management, Min-Max Replenishment<br>» ABC/VEN Inventory Classification		» CIPS Level 3 Advanced Certificate in Procurement and Supply Operations

Officer– Procurement

Organisation: **Public Health Organisation – Hospital Service Delivery**				
Competency Level:	Serial Number:	Primary Process:	Job Role:	Supervises:
Operational	2-21-22-2	Enable – Procurement	Officer– Procurement	Officer – Procurement and Supply

Contributions	Maintains the procurement process, to ensure that materials and resources are available when needed and within specifications stipulate in the Procurement business rules.
Technical Competency(ies):	2.1 Manage procurement costs and budget<br>2.2 Build and maintain supplier relationships
2.1 Manage procurement costs and budget	» Understand public procurement regulations<br>» Understand the importance of budget approval before initiating procurement<br>» Understanding the importance of financial and management accounting practices within procurement<br>» Understand the commercial factors that contribute toward cost reduction, price savings and value improvement opportunities<br>» Understand the principles of foreign exchange rates and how they impact prices
2.2 Build and maintain supplier relationships	» Understand the factors in the analysis of the market<br>» Understand the processes for prequalification and tender contracting<br>» Understand the factors that define a good supplier<br>» Understand that key suppliers must be located and that there is a total cost associated with procuring an item or service<br>» Understand supplier relationship management plans (e.g., sourcing, frequency of meetings, negotiate and monitor benchmarks for performance)<br>» Understand the process of educating suppliers to create value for customers by streamlining processes in the value chain<br>» Understand the difference between strategic and non-strategic suppliers and the associated supplier management principles<br>» Understand commercial factors that contribute toward cost reduction, price savings and value improvement opportunities<br>» Understand the process of strategic sourcing practices drive down cost across consecutive tenders<br>» Compare the process of working with suppliers to agree cost saving opportunities in the whole value chain, with an appropriate cost sharing mechanism applied to ensure a “win-win” outcome<br>» Understand the process of managing supplier risk<br>» Understand ethical sourcing
Personal/Management Competency(ies):	6.1 Demonstrate generic skills (e.g., literacy, numeracy, technology)<br>6.1.1 Exhibit high understanding of literacy and numeracy

	6.2 Demonstrate strong communication skills<br>6.2.1 Practice cultural awareness
**6.1 Demonstrate generic skills (e.g., literacy, numeracy, technology)**	
**6.1.1 Exhibit high understanding of literacy and numeracy**	» Speak clearly and confidently, organize information in a logical manner, and consider voice tone and pace<br>» Respond to verbal messages and other cues (active listening)<br>» Express ideas and opinions clearly in written and verbal form<br>» Communicate information accurately, concisely and confidently in writing and verbally<br>» Practice a level of mathematics suitable to the job held<br>» Compare visual presentation techniques, including charting, histograms and flow sheets<br>» Read and comprehend at a level necessary to properly complete duties of the position<br>» Understand the need to interpret written information in documents, such as reports, SOPs, LMIS forms, graphs, calendars, schedules, notices and directions<br>» Understand the need to pay attention to detail and identify the main ideas, detect inconsistencies and identify missing information in documents<br>» Enable computer skills — such as using Word, Excel, PowerPoint and the internet — suitable to the job level<br>» Understand the process of analysing and validating KPIs<br>» Understand how the evaluation process of activities that act as standards against which the KPIs are measured is carried out<br>» Examine the process of performing critical self-review on work before submission
**6.2 Demonstrate strong communication skills**	» Be truthful and trustworthy, and supply accurate information at all times<br>» Enable working as part of a workplace team<br>» Understand vocabulary and communication style and form for both written and verbal communication that is appropriate for the situation, audience and material being communicated (e.g., avoid unnecessary jargon, clearly explain medical and SCM terminology)<br>» Enable open communication within constraints of confidentiality<br>» Participate in the organisation of communications about logistics<br>» Enable participation in meetings, expressing one’s opinions, being aware of others’ needs, and being appropriately assertive when required<br>» Show perspective into the point of view of others, understanding their needs and goals<br>» Enable the practise of building trust, rapport and credibility with others<br>» Understand the communication needs and concerns of others and respond to them

» Understand the need to avoid conflict between work and personal interests
» Enable meaningful two-way communication
» Show influence on others by persuasively presenting thoughts and ideas
» Show assertiveness skills to deal with unreasonable requests and/or refusals that would compromise practice or consumer care
» Maintain a positive, supportive and appreciative attitude
» Show the ability to balance the organisation’s needs and the customer’s needs
» Show self-control by maintaining composure and keeping emotions in check, even in difficult situations; deal calmly and effectively with stressful situations
» Enable expressing of opinions and providing information in written and/or verbal form in a manner that does not elicit concern, anger or other adverse response
» Enable the use of a systematic process for following up that demonstrates written reports have been received and understood
» Understand the means by which responses to input to the work environment are monitored
» Seek practical ways to overcome barriers to communication
» Understand the need to ensuring language representation (e.g., interpreters) at meetings when appropriate
» Interpret difficult situations and disputes between staff that need to be tackled and resolved
» Enable giving both positive and negative feedback sensitively
» Understand the need to maintain and improve communication
» Examine the process of giving and requesting feedback frequently
» Examine effective communication with shop stewards and union representatives

6.2.1 Practice cultural awareness

» Understand the need for cultural awareness and sensitivity, treating all people with fairness, respect and dignity
» Enable anti-discriminatory practices in the organisation, including HR and disciplinary procedures
» Enable cultural sensitivity, equality and fairness at all levels of the organisation
» Challenge discriminatory behaviour directly and sensitively
» Act in a non-discriminatory way toward individuals and groups
» Enable integrating cultural awareness in learning and development approaches
» Avoid stereotypical responses by examining one’s own behaviour and bias
» Enable managing cultural diversity in teams and make the most of differences
» Compare strategies and/or resources for communicating effectively with people from different cultural backgrounds
» Enable the process to elicit information relating to values, beliefs and cultural backgrounds of consumers that may influence the way professional services are provided

Basal Technology Competency(ies):	7.4 Temperature and monitoring<br>7.6 ERP includes function of LMIS

	7.12 Basic Office Skills<br>7.13 Have a command of technology
7.4 Temperature and monitoring	» Compare the current technology approaches for Temperature monitoring and sensors<br>» Consider what is required to implement this technology in a country context<br>» Compare infrastructure requirement for heat-sensitive items at peripheral 'lower level' of SC depending on volume and nature of items to handle;<br>» Examine regular preventive maintenance and calibration of cold-chain equipment with biomedical engineers or logisticians<br>» Understand the policy for temperature monitoring tools during transportation and storage<br>» Understand the steps involved in health logistic management cycle: selection-order-delivery-reception-storage-distribution-inventory<br>» Understand hardware qualification and system validation of temperature monitoring<br>» Understand mass data management of temperature monitoring
7.6 ERP includes function of LMIS	» Enable a Master Data Management System<br>» Understand the critical components of an ERP system<br>» Understand the meaning of common document types found within an ERP/LMIS<br>» Understand the logic of data visibility within an ERP<br>» Understand the importance of data integrity, record management and information security
7.12 Basic Office Skills	» Have a good understanding of common presentation authoring packages<br>» Have a good understanding of common spreadsheet authoring packages<br>» Have a good understanding of common document authoring packages<br>» Have a good understanding of common email authoring packages<br>» Have a good understanding of common instant messaging packages<br>» Have a good understanding of common video conferencing authoring packages
7.13 Have a command of technology	» Enable the use of technology suitable to the job held<br>» Understand the need of monitoring new developments and technologies in the sector<br>» Compare new technologies and recognising potential benefits for the sector<br>» Understand that resources and support are provided across the organisation to enable colleagues to make the best use of available technology<br>» Understand that the organisation has a strategy for technology use<br>» Compare how to minimise environmental damage through technology use<br>» Enable the use of technology to maximise effectiveness and efficiency<br>» Enable the use of field-based technology (e.g., radio, general packet radio service (GPRS), satellite phone)

Advanced Technology Competency(ies):	7.2 Blockchain<br>7.3 Unmanned Aerial Vehicles (UAVs)<br>7.7 Automation<br>7.9 Additive Manufacturing<br>7.11 Cloud Computing<br>7.14 eProcurement
7.2 Blockchain	» Examine blockchain as a value-add capability for relevant global health use cases
7.3 Unmanned Aerial Vehicles (UAVs)	» Understand the current UAV landscape, their application and the enabling environment required to support country implementation<br>» Understand UAVs as a value-add capability for relevant global health use cases<br>» Understand the laws/ regulations around the use of UAV’s
7.7 Automation	» Understand automation as a value-add capability for relevant global health use cases
7.9 Additive Manufacturing	» Understand additive manufacturing as a value-add capability for relevant global health use cases
7.11 Cloud Computing	» Understand cloud computing as a value-add capability for relevant global health use cases
7.14 eProcurement	» Understand how to transact with an eProcurement system where necessary<br>» Understand eProcurement as a value-add capability for relevant global health use cases<br>» Understand the benefits and limitations of eProcurement to best utilise the system

Key Performance Indicators:

- % of emergency orders issued in the last 12 months
- % of product selection based on National Essential Medicines List
- % of procured products that meet Stringent Regulatory Authority (SRA) or WHO standards
- Supply adequacy - Net requirements are reflected in the Procurement pipeline to secure sufficient future supply.
- Supply Plan report; supplier performance report
- Fill Rate vs Planned fill rate
- Identify, Prioritize, and Aggregate Supply Chain Requirements Cycle Time - Actual Time taken to determine net supply chain requirements by product and SKU vs Planned Time
- Order accuracy - Net requirement versus order quantities in the planning horizon
- Ratio between median price of pro- ducts procured and the international median reference value
- Availability - Stock out rate - Number of missed line item quantity deliveries due to stock out versus total line item quantity deliveries
- Availability - Stock levels - Current on hand stock versus Planned on hand stock
- Data accuracy - Vendor Master data accuracy, item / SKU Master Data accuracy
- Demand analysis - Actual Demand versus Planned Demand
- Forecast Accuracy - Using various measures of Forecast Error e.g. MAD
- Inventory Days of Supply versus Planned Inventory days of Supply
- Reports submitted on time (for all and separate reports)

Training:	Qualifications Available:	Certifications Available:
» Contract Management<br>» Supplier Relationship Management (SRM)<br>» Analytical Skills<br>» Competitive Billing<br>» Negotiation<br>» Procurement/ RFQ Management		» CIPS Level 3 Advanced Certificate in Procurement and Supply Operations

Officer – Receiving and Inventory Management

Organisation:				
**Public Health Organisation – Hospital Service Delivery**				
Competency Level:	Serial Number:	Primary Process:	Job Role:	Supervises:
Operational	3-33-25-3	Deliver	Officer – Receiving and Inventory Management	Assistant – Storage

Contributions	Timeous recording of receipts, ensuring that the required staff levels, handling equipment and space are available to minimize the risk of damage and shrinkage.
Technical Competency(ies):	2.5 Ensure quality of products<br>3.1 Undertake storage, warehousing and inventory management<br>3.1.1 Manage storage of commodities during emergency<br>3.3 Supply commodities to sections within a facility<br>3.5 Manage disposal of products (e.g. expired, damaged, redundant products)<br>3.5.1 Define and direct process for managing redundant and returned stock<br>3.7 Manage re-packing of products

2.5 Ensure quality of products

» Use and monitor the processes for prequalification of suppliers

» Ensure health commodities are not counterfeit and meet quality standards through certification (ISO, WHO GMP

» Describe the process of implementing, conducting and maintaining a reporting system of pharmacovigilance (e.g. report adverse drug reactions, quality defects, batch recall) and equipment faults

» Describe the process of inspecting products when delivered and during storage to catch defects or problems before they are given to, or needed by, clients

» Ensure compliance with good distribution and good storage practices (temperature monitoring in the warehouse, transportation and distribution)

» Define product-specific shelf-life by which the product are acceptable or rejected (e.g. no less than 1 year for most of drugs, and less for short shelf-life like laboratory)

3.1 Undertake storage, warehousing and inventory management

» Efficiently distribute products among suppliers, distribution centres, warehouses and customers through a logistics network

» Identify inputs to a total systems approach to designing and managing the entire flow of information, materials, and services

» Describe the choice of equipment or materials based on suitability for intended use, accuracy, safety of use and cost

» Maintain warehouses formal storage locations that identify the row, rack section, level and shelf location, typically with an alphanumeric location bar code or label

	» Describe how medicines are appropriately stored, considering temperature, access and cleanliness<br>» Identify appropriate use of, and ability to maintain, the cold chain<br>» Describe the movement and storage of materials within a warehouse<br>» Describe how to secure the medical store and limit access to staff<br>» Describe organisation processes, instructions, rules and parameters for warehouse and inventory management and use of equipment (e.g., annual stock turns, expiry date tracking, stock procurement, rotation and retrieval, equipment maintenance)<br>» Describe policies and procedures for stock handling, distribution and withdrawal consistent with maintaining safety (e.g., policies for distribution of concentrated electrolyte solutions and cytotoxics, identification of cytotoxic drug products within the work environment, separation of like-named or like-packaged products)<br>» Identify requirements of information on the supplies being stored<br>» Describe the process of consolidating several items into larger units for fewer handlings<br>» Describe methods of stock rotation (e.g., first in first out, FIFO, or first to expire first out, FEFO)<br>» Describe the process of entering the quantity received, and updating the warehouse management system software with the stock on hand when each order is delivered<br>» Asses required levels of stock and/or equipment in specific situations<br>» Ensure accurate verification of rolling stocks<br>» Monitor the location and condition of the supplies being stored<br>» Collate and prepare information on the storage locations and facilities<br>» Identify any problems in using the storage locations and facilities
3.1.1 Manage storage of commodities during emergency	» Describe the types of warehouses available for emergency or disaster supply<br>» Describe the importance of the choice of storage site for emergency or disaster supply<br>» Describe the staff required for the storage of emergency or disaster supply<br>» Describe the equipment and material required for the warehousing of emergency or disaster supply<br>» Provide input to a rapid assessment of logistical needs in emergencies
3.3 Supply commodities to sections within a facility	» Review imprest quantities regularly and assist in the development of the list in conjunction with doctors and nurses<br>» Validate the requisition from different health care services within the facility
3.5 Manage disposal of products (e.g. expired, damaged, redundant products)	

3.5.1 Define and direct process for managing redundant and returned stock	» Confirm information on the supplies being returned<br>» Describe the flow of returned supplies<br>» Identify any problems with the flow of returned supplies<br>» Integrate options for improving the flow of returned supplies<br>» Apply procedures for monitoring the flow of returned supplies<br>» Describe and/or demonstrate recall procedures to be used in response to a product recall notice or to access the information promptly.<br>» Identify issues relevant to the urgency and scope of action required in response to a product recall notice<br>» Identify the information required for reporting and procedures with regards to following up on medication incidents<br>» Identify follow-up strategies likely to be effective in preventing recurrence (e.g., root cause analysis)<br>» Describe an integrated healthcare waste management plan<br>» Describe guides and procedures for collection, sorting, transport and disposal<br>» Conduct pre-disposal inspections<br>» Describe the prerequisites for products disposal<br>» Generate product disposal report
3.7 Manage re-packing of products	» Predict when re-packing or pre-packs are required<br>» Demonstrate a safe system for repacking
Personal/Management Competency(ies):	6.1 Demonstrate generic skills (e.g., literacy, numeracy, technology)<br>6.1.1 Exhibit high understanding of literacy and numeracy<br>6.2 Demonstrate strong communication skills<br>6.2.1 Practice cultural awareness
6.1 Demonstrate generic skills (e.g., literacy, numeracy, technology)	
6.1.1 Exhibit high understanding of literacy and numeracy	» Speak clearly and confidently, organize information in a logical manner, and consider voice tone and pace<br>» Respond to verbal messages and other cues (active listening)<br>» Express ideas and opinions clearly in written and verbal form<br>» Communicate information accurately, concisely and confidently in writing and verbally<br>» Practice a level of mathematics suitable to the job held<br>» Describe visual presentation techniques, including charting, histograms and flow sheets<br>» Read and comprehend at a level necessary to properly complete duties of the position<br>» Interpret written information in documents, such as reports, SOPs, LMIS forms, graphs, calendars, schedules, notices and directions

	» Pay attention to detail and identify the main ideas, detect inconsistencies and identify missing information in documents<br>» Apply computer skills — such as using Word, Excel, PowerPoint and the internet — suitable to the job level<br>» Describe the process of analysing and validating KPIs<br>» Participate in the evaluation of activities that act as standards against which the KPIs are measured<br>» Apply the process of performing critical self-review on work before submission
6.2 Demonstrate strong communication skills	» Be truthful and trustworthy, and supply accurate information at all times<br>» Work as part of a workplace team<br>» Describe vocabulary and communication style and form for both written and verbal communication that is appropriate for the situation, audience and material being communicated (e.g., avoid unnecessary jargon, clearly explain medical and SCM terminology)<br>» Encourage open communication within constraints of confidentiality<br>» Organize communications about logistics<br>» Encourage participation in meetings, expressing one's opinions, being aware of others' needs, and being appropriately assertive when required<br>» Apply perspective into the point of view of others, understanding their needs and goals<br>» Encourage building trust, rapport and credibility with others<br>» Anticipate the communication needs and concerns of others and respond to them<br>» Avoid conflict between work and personal interests<br>» Encourage meaningful two-way communication<br>» Influence others by persuasively presenting thoughts and ideas<br>» Apply assertiveness skills to deal with unreasonable requests and/or refusals that would compromise practice or consumer care<br>» Maintain a positive, supportive and appreciative attitude<br>» Apply the ability to balance the organisation's needs and the customer's needs<br>» Use self-control by maintaining composure and keeping emotions in check, even in difficult situations; deal calmly and effectively with stressful situations<br>» Encourage the expressing of opinions and providing information in written and/or verbal form in a manner that does not elicit concern, anger or other adverse response<br>» Apply the use of a systematic process for following up that demonstrates written reports have been received and understood<br>» Describe the means by which responses to input to the work environment are monitored<br>» Seek practical ways to overcome barriers to communication<br>» Identify language representation (e.g., interpreters) at meetings when appropriate

	» Tackle difficult situations and resolve disputes between staff<br>» Give both positive and negative feedback sensitively<br>» Maintain and improve communication<br>» Apply the process of giving and requesting feedback frequently<br>» Apply the ability to communicate effectively with shop stewards and union representatives
6.2.1 Practice cultural awareness	» Describe cultural awareness and sensitivity, treating all people with fairness, respect and dignity<br>» Describe anti-discriminatory practices in the organisation, including HR and disciplinary procedures<br>» Establish cultural sensitivity, equality and fairness at all levels of the organisation<br>» Challenge discriminatory behaviour directly and sensitively<br>» Act in a non-discriminatory way toward individuals and groups<br>» Integrate cultural awareness in learning and development approaches<br>» Avoid stereotypical responses by examining one's own behaviour and bias<br>» Manage cultural diversity in teams and make the most of differences<br>» Describe strategies and/or resources for communicating effectively with people from different cultural backgrounds<br>» Elicit information relating to values, beliefs and cultural backgrounds of consumers that may influence the way professional services are provided
Basal Technology Competency(ies):	7.4 Temperature and monitoring<br>7.6 ERP includes function of LMIS<br>7.12 Basic Office Skills<br>7.13 Have a command of technology
7.4 Temperature and monitoring	» Describe the current technology approaches for Temperature monitoring and sensors<br>» Describe what is required to implement this technology in a country context<br>» Describe infrastructure requirement for heat-sensitive items at peripheral 'lower level' of SC depending on volume and nature of items to handle;<br>» Define regular preventive maintenance and calibration of cold-chain equipment with biomedical engineers or logisticians<br>» Describe the policy for temperature monitoring tools during transportation and storage<br>» Describe the steps involved in health logistic management cycle: selection-order-delivery-reception-storage-distribution-inventory<br>» Describe hardware qualification and system validation of temperature monitoring<br>» Consider mass data management of temperature monitoring

7.6 ERP includes function of LMIS	» Support a Master Data Management System<br>» Describe the critical components of an ERP system<br>» Describe the meaning of common document types found within an ERP/LMIS<br>» Describe the logic of data visibility within an ERP<br>» Describe the importance of data integrity, record management and information security
7.12 Basic Office Skills	» Have a good understanding of common presentation authoring packages<br>» Have a good understanding of common spreadsheet authoring packages<br>» Have a good understanding of common document authoring packages<br>» Have a good understanding of common email authoring packages<br>» Have a good understanding of common instant messaging packages<br>» Have a good understanding of common video conferencing authoring packages
7.13 Have a command of technology	» Apply the use of technology suitable to the job held<br>» Provide input on monitoring new developments and technologies in the sector<br>» Identify new technologies and recognising potential benefits for the sector<br>» Identify resources and support that are provided across the organisation to enable colleagues to make the best use of available technology<br>» Ensure that the organisation has a strategy for technology use<br>» Describe how to minimise environmental damage through technology use<br>» Use technology to maximise effectiveness and efficiency<br>» Use field-based technology (e.g., radio, general packet radio service (GPRS), satellite phone)

Advanced Technology Competency(ies):	7.7 Automation
7.7 Automation	» Describe automation as a value-add capability for relevant global health use cases

Key Performance Indicators:

- Availability - Stock out rate - Number of missed line item quantity deliveries due to stock out versus total line item quantity deliveries
- Availability - Stock levels - Current on hand stock versus Planned on hand stock
- Safe product handling - Number of products handled during period out of conformance versus Number of products handled during period expressed as a % with prior period comparisons
- Customer Incidents Report number of incidents by month/week/day comparison with previous periods
- Number of order lines shipped with incorrectly captured Items
- Number of order lines with incorrect items/SKU's versus total number of order lines shipped as a %
- Regulatory - % of procured products that meet Stringent Regulatory Authority (SRA) or WHO standards
- Waste Management - Existence of supply chain management protocols for disposal of medical waste and management of unusable products
- Number of order lines delivered to incorrect end-user
- Number of orders incorrectly addressed versus number of orders processed expressed as a %
- Number of Order Lines delivered out of Quality Specifications - Number of Item/SKU delivered out of specification e.g. Cold Chain infractions, Expired products versus total Number of order lines delivered as a %. A comparative graph would be an appropriate tool for the measurement of this metric.
- % Product Transferred On-Time to Demand Requirement.
- Actual Number of products transferred outside of the Time Limits vs Number of products Transferred.
- Transfer Product Cycle Time - Actual Transfer Cycle Time versus Planned Transferred Cycle Time
- Accurate pick - Picking Accuracy number of order lines picked inaccurately divided by the total order lines picked (SKU and Quantity)
- On-time delivery - Actual Delivery Date & Time Vs Promised Delivery Data and Time
- Customer order fulfilment cycle time & rate - Actual Order Fulfilment Cycle Time versus Promised Customer Order Fulfilment Cycle time
- Warehousing and Inventory Management OTIF - On time and in full
- Number of warehouse locations used versus warehouse locations available (Pallet Positions may be used in larger warehouses)
- Line Items SKU's handled vs Items / SKU's Planned over a planning horizon
- Warehouse Utilization - Volume used vs Volume Available

Training:	Qualifications Available:	Certifications Available:
» Data management<br>» ERP Systems<br>» Legislation and Standards<br>» 3-way Receiving Match<br>» Code Handling/RFID (if available)<br>» Discrepancy Reporting and Resolution<br>» ID & Damage Inspection<br>» Inventory Management	» (CILT) International Diploma in Logistics & Transport	» SCPro™ Certification<br>» APICS Certified in logistics transportation and distribution certificate<br>» Online Post Graduate Diploma in Global Health Procurement and Supply Chain Management

» Property Control and Disposition<br>» Return Management<br>» MSDS/CoC/BoL/Environmental Interpretation		

Manager - Occupational Safety

Organisation:				
**Public Health Organisation – Hospital Service Delivery**				
Competency Level:	Serial Number:	Primary Process:	Job Role:	Supervises:
Operational	3-39-554-641-3	Enable – Regulatory (Individual) Compliance	Manager - Occupational Safety	-

Contributions	Responsible for compliance to the health and safety guidelines for the organisation. Promoting a safe and healthy working environment in the organisation. Responsible to reduce the number of work-related accidents and diseases in the organisation. Responsible to provide equitable compensation benefits to those who may get injured in work-related accidents or contract occupational diseases. Ensures the surveillance of the working environment
Technical Competency(ies):	3.9 Facility design

3.9 Facility design

- Provide inputs to the design and layout of the size and configuration of the facility/building.
- Describe the flow of product through the facility to making sure it is achievable with the selection of material handling equipment
- Achieve the balance of the health and safety requirements of the facility with its performance requirements

Personal/Management Competency(ies):	5.5 Manage and plan projects<br>5.5.4 Direct/participate in teamwork<br>6.1 Demonstrate generic skills (e.g., literacy, numeracy, technology)<br>6.1.1 Exhibit high understanding of literacy and numeracy<br>6.2 Demonstrate strong communication skills<br>6.2.1 Practice cultural awareness<br>6.4 Exhibit professional and ethical values<br>6.4.1 Demonstrate integrity

5.5 Manage and plan projects

5.5.4 Direct/participate in teamwork

- Describe the process of working with team members within their area of authority to establish achievable goals and strategies that are consistent with the objectives established for the organisation as a whole
- Describe an organisational chart that shows the lines of reporting and responsibility among staff
- Actively contribute a perspective and make a positive contribution to team-based problem solving and decision making

	» Provide feedback, encouragement and support to team members for progressing strategic goals<br>» Define team performance in relation to the organisation's mission and goals<br>» Encourage teams to think for themselves and resolve problems<br>» Encourage and harness diversity within the team to boost team effectiveness<br>» Encourage and support the team to work through its stages of development and perform well<br>» Describe roles and responsibilities in relation to employees' expertise and the expectations of collaborating team members<br>» Describe where their position fits in the structure (organisation, SC, healthcare sector) , the responsibilities their position entails and the accountabilities linked to the position
6.1 Demonstrate generic skills (e.g., literacy, numeracy, technology)	
6.1.1 Exhibit high understanding of literacy and numeracy	» Speak clearly and confidently, organize information in a logical manner, and consider voice tone and pace<br>» Respond to verbal messages and other cues (active listening)<br>» Express ideas and opinions clearly in written and verbal form<br>» Communicate information accurately, concisely and confidently in writing and verbally<br>» Practice a level of mathematics suitable to the job held<br>» Describe visual presentation techniques, including charting, histograms and flow sheets<br>» Read and comprehend at a level necessary to properly complete duties of the position<br>» Interpret written information in documents, such as reports, SOPs, LMIS forms, graphs, calendars, schedules, notices and directions<br>» Pay attention to detail and identify the main ideas, detect inconsistencies and identify missing information in documents<br>» Apply computer skills — such as using Word, Excel, PowerPoint and the internet — suitable to the job level<br>» Describe the process of analysing and validating KPIs<br>» Participate in the evaluation of activities that act as standards against which the KPIs are measured<br>» Apply the process of performing critical self-review on work before submission
6.2 Demonstrate strong communication skills	» Be truthful and trustworthy, and supply accurate information at all times<br>» Work as part of a workplace team<br>» Describe vocabulary and communication style and form for both written and verbal communication that is appropriate for the situation, audience

	and material being communicated (e.g., avoid unnecessary jargon, clearly explain medical and SCM terminology)<br>» Encourage open communication within constraints of confidentiality<br>» Organize communications about logistics<br>» Encourage participation in meetings, expressing one's opinions, being aware of others' needs, and being appropriately assertive when required<br>» Apply perspective into the point of view of others, understanding their needs and goals<br>» Encourage building trust, rapport and credibility with others<br>» Anticipate the communication needs and concerns of others and respond to them<br>» Avoid conflict between work and personal interests<br>» Encourage meaningful two-way communication<br>» Influence others by persuasively presenting thoughts and ideas<br>» Apply assertiveness skills to deal with unreasonable requests and/or refusals that would compromise practice or consumer care<br>» Maintain a positive, supportive and appreciative attitude<br>» Apply the ability to balance the organisation's needs and the customer's needs<br>» Use self-control by maintaining composure and keeping emotions in check, even in difficult situations; deal calmly and effectively with stressful situations<br>» Encourage the expressing of opinions and providing information in written and/or verbal form in a manner that does not elicit concern, anger or other adverse response<br>» Apply the use of a systematic process for following up that demonstrates written reports have been received and understood<br>» Describe the means by which responses to input to the work environment are monitored<br>» Seek practical ways to overcome barriers to communication<br>» Identify language representation (e.g., interpreters) at meetings when appropriate<br>» Tackle difficult situations and resolve disputes between staff<br>» Give both positive and negative feedback sensitively<br>» Maintain and improve communication<br>» Apply the process of giving and requesting feedback frequently<br>» Apply the ability to communicate effectively with shop stewards and union representatives
6.2.1 Practice cultural awareness	» Describe cultural awareness and sensitivity, treating all people with fairness, respect and dignity<br>» Describe anti-discriminatory practices in the organisation, including HR and disciplinary procedures<br>» Establish cultural sensitivity, equality and fairness at all levels of the organisation

- Challenge discriminatory behaviour directly and sensitively
- Act in a non-discriminatory way toward individuals and groups
- Integrate cultural awareness in learning and development approaches
- Avoid stereotypical responses by examining one's own behaviour and bias
- Manage cultural diversity in teams and make the most of differences
- Describe strategies and/or resources for communicating effectively with people from different cultural backgrounds
- Elicit information relating to values, beliefs and cultural backgrounds of consumers that may influence the way professional services are provided

6.4 Exhibit professional and ethical values

6.4.1 Demonstrate integrity

- Accept responsibility for one's own work tasks and performance
- Work within a framework of clearly understood humanitarian values and ethics
- Stand by decisions and hold others accountable when necessary
- Does not abuse one's own power or position
- Manage team members to ensure they do not abuse their power or position
- Identify when individuals or the organisation is straying from organisation goals, and challenge them to uphold ethics
- Resist undue political pressure in decision making
- Support staff in maintaining ethical stances
- Make time in team for ethical inquiry and reflection
- Show consistency between expressed principles and behaviour
- Provide input to ensure that principles, values and ethics are embedded in policy
- Act without consideration of personal gain
- Promote transparency in decision making structures and processes
- Provide input on ensuring programmes are acting with integrity, and recognising the impact of not doing so
- Recognise one's own limitations and act upon them
- Apply all standard operating procedures
- Work in a safe and legal way
- Demonstrate respect, dignity and consideration for consumers
- Discuss the impact of a no-blame culture on reporting and preventing recurrence of incidents
- Describe requests of colleagues that might be regarded as unreasonable
- Communicate (verbally and by example) expectations of the desired standards and approaches to be adopted
- Describe roles and responsibilities in terms of the position statement/duty statement of the position held

- Demonstrate punctuality
- Explain the obligation to apply professional care and expertise to deliver high quality of services
- Demonstrate care and attention to detail in undertaking work activities
- Describe appropriate attire and presentation for the role and situation
- Recognise and take responsibility for emotions
- Describe an environment in which others can talk and act without fear of repercussion
- Seek to keep commitments and not let people down
- Be trusting and cooperative when working alongside others
- Take on various responsibilities within the department as the need arises

Key Performance Indicators:
» Safety statics - Number of cases due to human error ranked by seriousness this period versus previous periods. A graphical representation would be indicated.<br>» Safety statics - Number of cases due to faulty equipment, ranked by seriousness of injury this period versus previous periods. A graphical representation would be indicated.<br>» Audit of safety equipment results this period versus previous periods<br>» Audit of Occupational safety signage and posters. This period versus previous periods<br>» Occupational safety educational awareness programmes delivered. Actual this period versus Planned this period<br>» Root cause analysis reports per reported incident. This period versus Last Periods

Training:	Qualifications Available:	Certifications Available:
» SHE compliance training<br>» Reporting standards training		» Occupational Certificate: Compliance Officer<br>» Occupational Certificate: Maintenance Planner<br>» APICS Principles of operations management series certificate<br>» SCPro™ Certification<br>» Logistics and Supply Chain Management MIT

Tactical
Assistant – Storage

Organisation:

Public Health Organisation – Hospital Service Delivery

Competency Level:	Serial Number:	Primary Process:	Job Role:	Supervises:
Tactical	3-33-25-1	Deliver	Assistant – Storage	-

Contributions	Performs the actual receiving, storage and disposition in accordance with the protocols governing the items being returned.
Technical Competency(ies):	3.1 Undertake storage, warehousing and inventory management<br>3.1.1 Manage storage of commodities during emergency<br>3.3 Supply commodities to sections within a facility

3.1 Undertake storage, warehousing and inventory management

- Execute distribution of products among suppliers, distribution centres, warehouses and customers through a logistics network
- Be aware of a total systems approach to designing and managing the entire flow of information, materials, and services
- Awareness of the choice of equipment or materials based on suitability for intended use, accuracy, safety of use and cost
- Awareness of the formal storage locations in the warehouse that identify the row, rack section, level and shelf location, typically with an alphanumeric location bar code or label
- Store medicines appropriately, considering temperature, access and cleanliness
- Ability to maintain the cold chain
- Execute the movement and storage of materials within a warehouse
- Be aware of the need to secure the medical store and limit access to staff
- Follow organisation processes, instructions, rules and parameters for warehouse and inventory management and use of equipment (e.g., annual stock turns, expiry date tracking, stock procurement, rotation and retrieval, equipment maintenance)
- Awareness of policies and procedures for stock handling, distribution and withdrawal consistent with maintaining safety (e.g., policies for distribution of concentrated electrolyte solutions and cytotoxics, identification of cytotoxic drug products within the work environment, separation of like-named or like-packaged products)
- Confirm information on the supplies being stored
- Awareness that one must consolidate several items into larger units for fewer handlings

	» Awareness of methods of stock rotation (e.g., first in first out, FIFO, or first to expire first out, FEFO)<br>» Enter the quantity received and update the warehouse/inventory/LMIS system software with the stock on hand when each order is delivered according to requisite SOP's<br>» Awareness that there are required levels of stock and/or equipment in specific situations<br>» Awareness of the importance of accurate verification of rolling stocks<br>» Awareness of the importance of monitoring the location and condition of the supplies being stored<br>» Awareness of the importance of the information on the storage locations and facilities<br>» Awareness of the need to communicate any problems in using the storage locations and facilities
3.1.1 Manage storage of commodities during emergency	» Awareness of the types of warehouses available for emergency or disaster supply<br>» Awareness of the choice of storage site for emergency or disaster supply<br>» Awareness of the staff required for the storage of emergency or disaster supply<br>» Awareness of the equipment and material required for the warehousing of emergency or disaster supply<br>» Awareness of the need for a rapid assessment of logistical needs in emergencies
3.3 Supply commodities to sections within a facility	» Awareness of the use of an imprest list<br>» Awareness of the process of validating the requisition from different health care services within the facility
Personal/Management Competency(ies):	6.1 Demonstrate generic skills (e.g., literacy, numeracy, technology)<br>6.1.1 Exhibit high understanding of literacy and numeracy<br>6.2 Demonstrate strong communication skills<br>6.2.1 Practice cultural awareness
6.1 Demonstrate generic skills (e.g., literacy, numeracy, technology)	
6.1.1 Exhibit high understanding of literacy and numeracy	» Speak clearly and confidently, organize information in a logical manner, and consider voice tone and pace<br>» Respond to verbal messages and other cues (active listening)<br>» Express ideas and opinions clearly in written and verbal form<br>» Communicate information accurately, concisely and confidently in writing and verbally<br>» Practice a level of mathematics suitable to the job held<br>» Awareness of visual presentation techniques, including charting, histograms and flow sheets

- Read and comprehend at a level necessary to properly complete duties of the position
- Awareness of the need to interpret written information in documents, such as reports, SOPs, LMIS forms, graphs, calendars, schedules, notices and directions
- Awareness of the need to paying attention to detail and identifying the main ideas, detecting inconsistencies and identifying missing information in documents
- Execute computer skills — such as using Word, Excel, PowerPoint and the internet — suitable to the job level
- Operational knowledge of analysing and validating KPIs
- Awareness of how the evaluation process of activities that act as standards against which the KPIs are measured is carried out
- Execute the process of performing critical self-review on work before submission

6.2 Demonstrate strong communication skills

- Be truthful and trustworthy, and supply accurate information at all times
- Realize the need to work as part of a workplace team
- Awareness of vocabulary and communication style and form for both written and verbal communication that is appropriate for the situation, audience and material being communicated (e.g., avoid unnecessary jargon, clearly explain medical and SCM terminology)
- Awareness of open communication within constraints of confidentiality
- Awareness of communications about logistics
- Participate in meetings, expressing one's opinions, being aware of others' needs, and being appropriately assertive when required
- Awareness of perspective into the point of view of others, understanding their needs and goals
- Build trust, rapport and credibility with others
- Awareness of the communication needs and concerns of others and respond to them
- Awareness of the need to avoid conflict between work and personal interests
- Practice meaningful two-way communication
- Realize that others are influenced by persuasively presenting thoughts and ideas
- Awareness of the need for assertiveness skills to deal with unreasonable requests and/or refusals that would compromise practice or consumer care
- Maintain a positive, supportive and appreciative attitude
- Awareness of the need to balance the organisation's needs and the customer's needs
- Execute self-control by maintaining composure and keeping emotions in check, even in difficult situations; deal calmly and effectively with stressful situations

	» Express opinions and provide information in written and/or verbal form in a manner that does not elicit concern, anger or other adverse response<br>» Basic operational knowledge of a systematic process for following up that demonstrates written reports have been received and understood<br>» Basic operational knowledge of the means by which responses to input to the work environment are monitored<br>» Seek practical ways to overcome barriers to communication<br>» Awareness of the need for language representation (e.g., interpreters) at meetings when appropriate<br>» Realize that difficult situations and disputes between staff need to be tackled and resolved<br>» Realize the need to give both positive and negative feedback sensitively<br>» Awareness of the need to maintain and improve communication<br>» Execute the process of giving and requesting feedback frequently<br>» Execute effective communication with shop stewards and union representatives
6.2.1 Practice cultural awareness	» Awareness of the need for cultural awareness and sensitivity, treating all people with fairness, respect and dignity<br>» Awareness of the anti-discriminatory practices in the organisation, including HR and disciplinary procedures<br>» Awareness of the need for cultural sensitivity, equality and fairness at all levels of the organisation<br>» Challenge discriminatory behaviour directly and sensitively<br>» Act in a non-discriminatory way toward individuals and groups<br>» Awareness of the need for integrating cultural awareness in learning and development approaches<br>» Avoid stereotypical responses by examining one's own behaviour and bias<br>» Awareness of the need to manage cultural diversity in teams and make the most of differences<br>» Awareness of strategies and/or resources for communicating effectively with people from different cultural backgrounds<br>» Awareness of the need to elicit information relating to values, beliefs and cultural backgrounds of consumers that may influence the way professional services are provided
Basal Technology Competency(ies):	7.6 ERP includes function of LMIS<br>7.13 Have a command of technology
7.6 ERP includes function of LMIS	» Awareness of a Master Data Management System<br>» Awareness of the critical components of an ERP system

	» Awareness of the meaning of common document types found within an ERP/LMIS<br>» Awareness of the logic of data visibility within an ERP<br>» Awareness of the importance of data integrity, record management and information security
7.13 Have a command of technology	» Execute the use of technology suitable to the job held<br>» Awareness of the need to monitor new developments and technologies in the sector<br>» Awareness of new technologies and recognising potential benefits for the sector<br>» Awareness that resources and support are provided across the organisation to enable colleagues to make the best use of available technology<br>» Awareness of the fact that the organisation has a strategy for technology use<br>» Awareness of how to minimise environmental damage through technology use<br>» Basic operational knowledge of technology to maximise effectiveness and efficiency<br>» Basic operational knowledge of field-based technology (e.g., radio, general packet radio service (GPRS), satellite phone)
Advanced Technology Competency(ies):	7.7 Automation
7.7 Automation	» Awareness of automation as a value-add capability for relevant global health use cases

Key Performance Indicators:

- » Availability - Stock out rate - Number of missed line item quantity deliveries due to stock out versus total line item quantity deliveries
- » Availability - Stock levels - Current on hand stock versus Planned on hand stock
- » Safe product handling - Number of products handled during period out of conformance versus Number of products handled during period expressed as a % with prior period comparisons
- » Customer Incidents Report number of incidents by month/week/day comparison with previous periods
- » No of order lines shipped with incorrectly captured Items
- » Number of order lines with incorrect items/SKU's versus total number of order lines shipped as a %
- » Regulatory - % of procured products that meet Stringent Regulatory Authority (SRA) or WHO standards
- » Waste Management - Existence of supply chain management protocols for disposal of medical waste and management of unusable products
- » Number of order lines delivered to incorrect end-user - Number of orders incorrectly addressed versus number of orders processed expressed as a %

- Number of Order Lines delivered out of Quality Specifications - Number of Item/SKU delivered out of specification e.g. Cold Chain infractions, Expired products versus total Number of order lines delivered as a %. A comparative graph would be an appropriate tool for the measurement of this metric.
- % Product Transferred On-Time to Demand Requirement. Actual Number of products transferred outside of the Time Limits vs Number of products Transferred.
- Transfer Product Cycle Time - Actual Transfer Cycle Time versus Planned Transferred Cycle Time
- Accurate pick - Picking Accuracy number of order lines picked inaccurately divided by the total order lines picked (SKU and Quantity)
- On-time delivery - Actual Delivery Date & Time Vs Promised Delivery Data and Time
- Customer order fulfilment cycle time & rate - Actual Order Fulfilment Cycle Time versus Promised Customer Order Fulfilment Cycle time
- Warehousing and Inventory Management OTIF - On time and in full

Training:	Qualifications Available:	Certifications Available:
» Material handling equipment usage		» Occupational Certificate: Store Person<br>» Occupational Certificate: Dispatching and Receiving Clerk<br>» Describe the use of stores and warehousing in operations skills programme

Public Health Organisation – Clinic Service Delivery Level

Managerial

Lead – Procurement and Supply

Organisation:<br>Public Health Organisation – Clinic Service Delivery Level				
Competency Level:<br>Managerial	Serial Number:<br>2-21-241-554-3	Primary Process:<br>Procure	Job Role:<br>Lead – Procurement and Supply	Supervises:<br>Officer – Procurement and Supply, Clerk – Supply

Contributions	Evaluates requirements for procurement and supply and takes corrective steps at the clinic and community level to support the Supply Chain Strategy of the Public Health System Organisation. Will be called upon to support the supply chain function across the clinic and community
Technical Competency(ies):	2.1 Manage procurement costs and budget<br>2.4 Undertake contract management and risk<br>2.4.1 Place commodity orders

2.1 Manage procurement costs and budget	» Incorporate and follow public procurement regulations<br>» Ensure budget approval is in place before initiating procurement<br>» Demonstrate sound understanding and application of financial and management accounting practices within procurement<br>» Apply commercial factors that contribute toward cost reduction, price savings and value improvement opportunities<br>» Describe the principles of foreign exchange rates and how they impact prices
2.4 Undertake contract management and risk	
2.4.1 Place commodity orders	» Obtain authorisations to administer the contracts<br>» Describe the type of supplies required<br>» Obtain reference sources and supplier catalogues to clarify required product and its availability<br>» Comply with policies and procedures to order required stock and equipment<br>» Confirm the orders with suppliers<br>» Identify any problems in placing orders

	» Comply with procedures for placing orders<br>» Identify any problems with order delivery and recommend options for progressing order delivery<br>» Ensure effective mechanisms for checking invoices exist and take action to adjust payments accordingly<br>» Receive or raise requisitions and arrange purchase orders and payments
Personal/Management Competency(ies):	5.5 Manage and plan projects<br>5.5.2 Execute strategic decision making<br>5.5.4 Direct/participate in teamwork<br>6.1 Demonstrate generic skills (e.g., literacy, numeracy, technology)<br>6.1.1 Exhibit high understanding of literacy and numeracy<br>6.2 Demonstrate strong communication skills<br>6.2.1 Practice cultural awareness
**5.5 Manage and plan projects**	
**5.5.2 Execute strategic decision making**	» Make decisions regarding one's own workload and area of responsibility<br>» Prioritize competing tasks and perform them quickly and efficiently according to their importance; find new ways of organizing or planning work to accomplish it more efficiently<br>» Ensure proper time and space for consultation around decisions being made.<br>» Consider the input of resources including staff needed<br>» Check assumptions against facts<br>» Identify the key issues in a complex situation and come to the heart of the problem quickly<br>» Gather relevant information before making decisions<br>» Describe the process of providing input for making strategic decisions in the interest of the organisation's goals<br>» Describe when to analyse, evaluate and execute tough decisions when necessary<br>» Consider the impact of decisions on others' work or team goals<br>» Describe the process to communicate decisions and ensure they are incorporated into policies and processes<br>» Describe inclusive and consultative strategic planning to establish strategic objectives<br>» Identify and support emerging trends and practices<br>» Describe the macro and long-term consequences of decisions
**5.5.4 Direct/participate in teamwork**	» Describe the process of working with team members within their area of authority to establish achievable goals and strategies that

	are consistent with the objectives established for the organisation as a whole<br>» Describe an organisational chart that shows the lines of reporting and responsibility among staff<br>» Actively contribute a perspective and make a positive contribution to team-based problem solving and decision making<br>» Provide feedback, encouragement and support to team members for progressing strategic goals<br>» Define team performance in relation to the organisation’s mission and goals<br>» Encourage teams to think for themselves and resolve problems<br>» Encourage and harness diversity within the team to boost team effectiveness<br>» Encourage and support the team to work through its stages of development and perform well<br>» Describe roles and responsibilities in relation to employees’ expertise and the expectations of collaborating team members<br>» Describe where their position fits in the structure (organisation, SC, healthcare sector) , the responsibilities their position entails and the accountabilities linked to the position
6.1 Demonstrate generic skills (e.g., literacy, numeracy, technology)	
6.1.1 Exhibit high understanding of literacy and numeracy	» Speak clearly and confidently, organize information in a logical manner, and consider voice tone and pace<br>» Respond to verbal messages and other cues (active listening)<br>» Express ideas and opinions clearly in written and verbal form<br>» Communicate information accurately, concisely and confidently in writing and verbally<br>» Practice a level of mathematics suitable to the job held<br>» Describe visual presentation techniques, including charting, histograms and flow sheets<br>» Read and comprehend at a level necessary to properly complete duties of the position<br>» Interpret written information in documents, such as reports, SOPs, LMIS forms, graphs, calendars, schedules, notices and directions<br>» Pay attention to detail and identify the main ideas, detect inconsistencies and identify missing information in documents<br>» Apply computer skills — such as using Word, Excel, PowerPoint and the internet — suitable to the job level<br>» Describe the process of analysing and validating KPIs<br>» Participate in the evaluation of activities that act as standards against which the KPIs are measured<br>» Apply the process of performing critical self-review on work before submission

6.2 Demonstrate strong communication skills	» Be truthful and trustworthy, and supply accurate information at all times<br>» Work as part of a workplace team<br>» Describe vocabulary and communication style and form for both written and verbal communication that is appropriate for the situation, audience and material being communicated (e.g., avoid unnecessary jargon, clearly explain medical and SCM terminology)<br>» Encourage open communication within constraints of confidentiality<br>» Organize communications about logistics<br>» Encourage participation in meetings, expressing one's opinions, being aware of others' needs, and being appropriately assertive when required<br>» Apply perspective into the point of view of others, understanding their needs and goals<br>» Encourage building trust, rapport and credibility with others<br>» Anticipate the communication needs and concerns of others and respond to them<br>» Avoid conflict between work and personal interests<br>» Encourage meaningful two-way communication<br>» Influence others by persuasively presenting thoughts and ideas<br>» Apply assertiveness skills to deal with unreasonable requests and/or refusals that would compromise practice or consumer care<br>» Maintain a positive, supportive and appreciative attitude<br>» Apply the ability to balance the organisation's needs and the customer's needs<br>» Use self-control by maintaining composure and keeping emotions in check, even in difficult situations; deal calmly and effectively with stressful situations<br>» Encourage the expressing of opinions and providing information in written and/or verbal form in a manner that does not elicit concern, anger or other adverse response<br>» Apply the use of a systematic process for following up that demonstrates written reports have been received and understood<br>» Describe the means by which responses to input to the work environment are monitored<br>» Seek practical ways to overcome barriers to communication<br>» Identify language representation (e.g., interpreters) at meetings when appropriate<br>» Tackle difficult situations and resolve disputes between staff<br>» Give both positive and negative feedback sensitively<br>» Maintain and improve communication<br>» Apply the process of giving and requesting feedback frequently

- Apply the ability to communicate effectively with shop stewards and union representatives

6.2.1 Practice cultural awareness	» Describe cultural awareness and sensitivity, treating all people with fairness, respect and dignity<br>» Describe anti-discriminatory practices in the organisation, including HR and disciplinary procedures<br>» Establish cultural sensitivity, equality and fairness at all levels of the organisation<br>» Challenge discriminatory behaviour directly and sensitively<br>» Act in a non-discriminatory way toward individuals and groups<br>» Integrate cultural awareness in learning and development approaches<br>» Avoid stereotypical responses by examining one's own behaviour and bias<br>» Manage cultural diversity in teams and make the most of differences<br>» Describe strategies and/or resources for communicating effectively with people from different cultural backgrounds<br>» Elicit information relating to values, beliefs and cultural backgrounds of consumers that may influence the way professional services are provided
Basal Technology Competency(ies):	7.4 Temperature and monitoring<br>7.6 ERP includes function of LMIS<br>7.12 Basic Office Skills<br>7.13 Have a command of technology
7.4 Temperature and monitoring	» Describe the current technology approaches for Temperature monitoring and sensors<br>» Describe what is required to implement this technology in a country context<br>» Describe infrastructure requirement for heat-sensitive items at peripheral 'lower level' of SC depending on volume and nature of items to handle;<br>» Define regular preventive maintenance and calibration of cold-chain equipment with biomedical engineers or logisticians<br>» Describe the policy for temperature monitoring tools during transportation and storage<br>» Describe the steps involved in health logistic management cycle: selection-order-delivery-reception-storage-distribution-inventory<br>» Describe hardware qualification and system validation of temperature monitoring<br>» Consider mass data management of temperature monitoring

7.6 ERP includes function of LMIS	» Support a Master Data Management System<br>» Describe the critical components of an ERP system<br>» Describe the meaning of common document types found within an ERP/LMIS<br>» Describe the logic of data visibility within an ERP<br>» Describe the importance of data integrity, record management and information security
7.12 Basic Office Skills	» Have a good understanding of common presentation authoring packages<br>» Have a good understanding of common spreadsheet authoring packages<br>» Have a good understanding of common document authoring packages<br>» Have a good understanding of common email authoring packages<br>» Have a good understanding of common instant messaging packages<br>» Have a good understanding of common video conferencing authoring packages
7.13 Have a command of technology	» Apply the use of technology suitable to the job held<br>» Provide input on monitoring new developments and technologies in the sector<br>» Identify new technologies and recognising potential benefits for the sector<br>» Identify resources and support that are provided across the organisation to enable colleagues to make the best use of available technology<br>» Ensure that the organisation has a strategy for technology use<br>» Describe how to minimise environmental damage through technology use<br>» Use technology to maximise effectiveness and efficiency<br>» Use field-based technology (e.g., radio, general packet radio service (GPRS), satellite phone)
Advanced Technology Competency(ies):	7.2 Blockchain<br>7.3 Unmanned Aerial Vehicles (UAVs)<br>7.7 Automation<br>7.9 Additive Manufacturing<br>7.11 Cloud Computing<br>7.14 eProcurement
7.2 Blockchain	» Describe blockchain as a value-add capability for relevant global health use cases

7.3 Unmanned Aerial Vehicles (UAVs)	» Describe the current UAV landscape, their application and the enabling environment required to support country implementation<br>» Describe UAVs as a value-add capability for relevant global health use cases<br>» Describe the laws/ regulations around the use of UAV’s
7.7 Automation	» Describe automation as a value-add capability for relevant global health use cases
7.9 Additive Manufacturing	» Describe additive manufacturing as a value-add capability for relevant global health use cases
7.11 Cloud Computing	» Describe cloud computing as a value-add capability for relevant global health use cases
7.14 eProcurement	» Describe how to transact with an eProcurement system where necessary<br>» Describe eProcurement as a value-add capability for relevant global health use cases<br>» Apply the benefits and limitations of eProcurement to best utilise the system

Key Performance Indicators:

- Vendor on time delivery (VOTD)
- % of emergency orders issued in the last 12 months
- Supply adequacy - Net requirements are reflected in the Procurement pipeline to secure sufficient future supply.
- Supply Plan report; supplier performance report
- Order accuracy - Net requirement versus order quantities in the planning horizon
- Availability - Stock out rate - Number of missed line item quantity deliveries due to stock out versus total line item quantity deliveries
- Availability - Stock levels - Current on hand stock versus Planned on hand stock
- % of product selection based on National Essential Medicines List
- % of procured products that meet Stringent Regulatory Authority (SRA) or WHO standards
- Data accuracy - Vendor Master data accuracy, item / SKU Master Data accuracy
- Demand analysis - Actual Demand versus Planned Demand

- Fill Rate vs Planned fill rate
- Forecast Accuracy - Using various measures of Forecast Error e.g. MAD
- Identify, Prioritize, and Aggregate Supply Chain Requirements Cycle Time - Actual Time taken to determine net supply chain requirements by product and SKU vs Planned Time
- Inventory Days of Supply versus Planned Inventory days of Supply
- Ratio between median price of pro- ducts procured and the international median reference value
- Reports submitted on time (for all and separate reports)

Training:	Qualifications Available:	Certifications Available:
» Procurement<br>» MSDS/CoC/BoL/Environmental Interpretation<br>» Logistics Management<br>» Delivery Balancing<br>» Forecasting<br>» Master Scheduling<br>» Order Management<br>» Prioritization<br>» Basics of Supply Chain Management	» (CILT) International Diploma in Logistics & Transport<br>» Diploma in Export Management<br>» Diploma in International Trade Management in Exports/Imports	» CIPS Level 4 Diploma in Procurement and Supply<br>» SPSM ® (Essential Procurement Skills)<br>» SPSM2® (Global Procurement Management)<br>» APICS Certified in production and inventory management certificate<br>» Manage Logistics Operations skills programme<br>» Apply fundamental concepts and principles of supply chain management skills programme

Operational
Officer – Receiving and Inventory Management

Organisation:

Public Health Organisation – Clinic Service Delivery Level

Competency Level:	Serial Number:	Primary Process:	Job Role:	Supervises:
Operational	3-33-25-3	Deliver	Officer – Receiving and Inventory Management	Assistant – Storage

Contributions	Timeous recording of receipts, ensuring that the required staff levels, handling equipment and space are available to minimize the risk of damage and shrinkage.
Technical Competency(ies):	2.5 Ensure quality of products<br>3.1 Undertake storage, warehousing and inventory management<br>3.1.1 Manage storage of commodities during emergency<br>3.3 Supply commodities to sections within a facility<br>3.5 Manage disposal of products (e.g. expired, damaged, redundant products)<br>3.5.1 Define and direct process for managing redundant and returned stock<br>3.7 Manage re-packing of products

2.5 Ensure quality of products

» Use and monitor the processes for prequalification of suppliers

» Ensure health commodities are not counterfeit and meet quality standards through certification (ISO, WHO GMP

» Describe the process of implementing, conducting and maintaining a reporting system of pharmacovigilance (e.g. report adverse drug reactions, quality defects, batch recall) and equipment faults

» Describe the process of inspecting products when delivered and during storage to catch defects or problems before they are given to, or needed by, clients

» Ensure compliance with good distribution and good storage practices (temperature monitoring in the warehouse, transportation and distribution)

» Define product-specific shelf-life by which the product are acceptable or rejected (e.g. no less than 1 year for most of drugs, and less for short shelf-life like laboratory)

3.1 Undertake storage, warehousing and inventory management

» Efficiently distribute products among suppliers, distribution centres, warehouses and customers through a logistics network

» Identify inputs to a total systems approach to designing and managing the entire flow of information, materials, and services
» Describe the choice of equipment or materials based on suitability for intended use, accuracy, safety of use and cost
» Maintain warehouses formal storage locations that identify the row, rack section, level and shelf location, typically with an alphanumeric location bar code or label
» Describe how medicines are appropriately stored, considering temperature, access and cleanliness
» Identify appropriate use of, and ability to maintain, the cold chain
» Describe the movement and storage of materials within a warehouse
» Describe how to secure the medical store and limit access to staff
» Describe organisation processes, instructions, rules and parameters for warehouse and inventory management and use of equipment (e.g., annual stock turns, expiry date tracking, stock procurement, rotation and retrieval, equipment maintenance)
» Describe policies and procedures for stock handling, distribution and withdrawal consistent with maintaining safety (e.g., policies for distribution of concentrated electrolyte solutions and cytotoxics, identification of cytotoxic drug products within the work environment, separation of like-named or like-packaged products)
» Identify requirements of information on the supplies being stored
» Describe the process of consolidating several items into larger units for fewer handlings
» Describe methods of stock rotation (e.g., first in first out, FIFO, or first to expire first out, FEFO)
» Describe the process of entering the quantity received, and updating the warehouse management system software with the stock on hand when each order is delivered
» Asses required levels of stock and/or equipment in specific situations
» Ensure accurate verification of rolling stocks
» Monitor the location and condition of the supplies being stored
» Collate and prepare information on the storage locations and facilities
» Identify any problems in using the storage locations and facilities

3.1.1 Manage storage of commodities during emergency

» Describe the types of warehouses available for emergency or disaster supply
» Describe the importance of the choice of storage site for emergency or disaster supply
» Describe the staff required for the storage of emergency or disaster supply
» Describe the equipment and material required for the warehousing of emergency or disaster supply
» Provide input to a rapid assessment of logistical needs in emergencies

3.3 Supply commodities to sections within a facility	» Review imprest quantities regularly and assist in the development of the list in conjunction with doctors and nurses<br>» Validate the requisition from different health care services within the facility
3.5 Manage disposal of products (e.g. expired, damaged, redundant products)	
3.5.1 Define and direct process for managing redundant and returned stock	» Confirm information on the supplies being returned<br>» Describe the flow of returned supplies<br>» Identify any problems with the flow of returned supplies<br>» Integrate options for improving the flow of returned supplies<br>» Apply procedures for monitoring the flow of returned supplies<br>» Describe and/or demonstrate recall procedures to be used in response to a product recall notice or to access the information promptly.<br>» Identify issues relevant to the urgency and scope of action required in response to a product recall notice<br>» Identify the information required for reporting and procedures with regards to following up on medication incidents<br>» Identify follow-up strategies likely to be effective in preventing recurrence (e.g., root cause analysis)<br>» Describe an integrated healthcare waste management plan<br>» Describe guides and procedures for collection, sorting, transport and disposal<br>» Conduct pre-disposal inspections<br>» Describe the prerequisites for products disposal<br>» Generate product disposal report
3.7 Manage re-packing of products	» Predict when re-packing or pre-packs are required<br>» Demonstrate a safe system for repacking
Personal/Management Competency(ies):	6.1 Demonstrate generic skills (e.g., literacy, numeracy, technology)<br>6.1.1 Exhibit high understanding of literacy and numeracy<br>6.2 Demonstrate strong communication skills<br>6.2.1 Practice cultural awareness
6.1 Demonstrate generic skills (e.g., literacy, numeracy, technology)	
6.1.1 Exhibit high understanding of literacy and numeracy	» Speak clearly and confidently, organize information in a logical manner, and consider voice tone and pace<br>» Respond to verbal messages and other cues (active listening)<br>» Express ideas and opinions clearly in written and verbal form

	» Communicate information accurately, concisely and confidently in writing and verbally<br>» Practice a level of mathematics suitable to the job held<br>» Describe visual presentation techniques, including charting, histograms and flow sheets<br>» Read and comprehend at a level necessary to properly complete duties of the position<br>» Interpret written information in documents, such as reports, SOPs, LMIS forms, graphs, calendars, schedules, notices and directions<br>» Pay attention to detail and identify the main ideas, detect inconsistencies and identify missing information in documents<br>» Apply computer skills — such as using Word, Excel, PowerPoint and the internet — suitable to the job level<br>» Describe the process of analysing and validating KPIs<br>» Participate in the evaluation of activities that act as standards against which the KPIs are measured<br>» Apply the process of performing critical self-review on work before submission
6.2 Demonstrate strong communication skills	» Be truthful and trustworthy, and supply accurate information at all times<br>» Work as part of a workplace team<br>» Describe vocabulary and communication style and form for both written and verbal communication that is appropriate for the situation, audience and material being communicated (e.g., avoid unnecessary jargon, clearly explain medical and SCM terminology)<br>» Encourage open communication within constraints of confidentiality<br>» Organize communications about logistics<br>» Encourage participation in meetings, expressing one's opinions, being aware of others' needs, and being appropriately assertive when required<br>» Apply perspective into the point of view of others, understanding their needs and goals<br>» Encourage building trust, rapport and credibility with others<br>» Anticipate the communication needs and concerns of others and respond to them<br>» Avoid conflict between work and personal interests<br>» Encourage meaningful two-way communication<br>» Influence others by persuasively presenting thoughts and ideas<br>» Apply assertiveness skills to deal with unreasonable requests and/or refusals that would compromise practice or consumer care<br>» Maintain a positive, supportive and appreciative attitude

	» Apply the ability to balance the organisation's needs and the customer's needs<br>» Use self-control by maintaining composure and keeping emotions in check, even in difficult situations; deal calmly and effectively with stressful situations<br>» Encourage the expressing of opinions and providing information in written and/or verbal form in a manner that does not elicit concern, anger or other adverse response<br>» Apply the use of a systematic process for following up that demonstrates written reports have been received and understood<br>» Describe the means by which responses to input to the work environment are monitored<br>» Seek practical ways to overcome barriers to communication<br>» Identify language representation (e.g., interpreters) at meetings when appropriate<br>» Tackle difficult situations and resolve disputes between staff<br>» Give both positive and negative feedback sensitively<br>» Maintain and improve communication<br>» Apply the process of giving and requesting feedback frequently<br>» Apply the ability to communicate effectively with shop stewards and union representatives
6.2.1 Practice cultural awareness	» Describe cultural awareness and sensitivity, treating all people with fairness, respect and dignity<br>» Describe anti-discriminatory practices in the organisation, including HR and disciplinary procedures<br>» Establish cultural sensitivity, equality and fairness at all levels of the organisation<br>» Challenge discriminatory behaviour directly and sensitively<br>» Act in a non-discriminatory way toward individuals and groups<br>» Integrate cultural awareness in learning and development approaches<br>» Avoid stereotypical responses by examining one's own behaviour and bias<br>» Manage cultural diversity in teams and make the most of differences<br>» Describe strategies and/or resources for communicating effectively with people from different cultural backgrounds<br>» Elicit information relating to values, beliefs and cultural backgrounds of consumers that may influence the way professional services are provided
Basal Technology Competency(ies):	7.4 Temperature and monitoring<br>7.6 ERP includes function of LMIS<br>7.12 Basic Office Skills

7.13 Have a command of technology	
7.4 Temperature and monitoring	» Describe the current technology approaches for Temperature monitoring and sensors<br>» Describe what is required to implement this technology in a country context<br>» Describe infrastructure requirement for heat-sensitive items at peripheral 'lower level' of SC depending on volume and nature of items to handle;<br>» Define regular preventive maintenance and calibration of cold-chain equipment with biomedical engineers or logisticians<br>» Describe the policy for temperature monitoring tools during transportation and storage<br>» Describe the steps involved in health logistic management cycle: selection-order-delivery-reception-storage-distribution-inventory<br>» Describe hardware qualification and system validation of temperature monitoring<br>» Consider mass data management of temperature monitoring
7.6 ERP includes function of LMIS	» Support a Master Data Management System<br>» Describe the critical components of an ERP system<br>» Describe the meaning of common document types found within an ERP/LMIS<br>» Describe the logic of data visibility within an ERP<br>» Describe the importance of data integrity, record management and information security
7.12 Basic Office Skills	» Have a good understanding of common presentation authoring packages<br>» Have a good understanding of common spreadsheet authoring packages<br>» Have a good understanding of common document authoring packages<br>» Have a good understanding of common email authoring packages<br>» Have a good understanding of common instant messaging packages<br>» Have a good understanding of common video conferencing authoring packages
7.13 Have a command of technology	» Apply the use of technology suitable to the job held<br>» Provide input on monitoring new developments and technologies in the sector<br>» Identify new technologies and recognising potential benefits for the sector

- Identify resources and support that are provided across the organisation to enable colleagues to make the best use of available technology
- Ensure that the organisation has a strategy for technology use
- Describe how to minimise environmental damage through technology use
- Use technology to maximise effectiveness and efficiency
- Use field-based technology (e.g., radio, general packet radio service (GPRS), satellite phone)

Advanced Technology Competency(ies):	7.7 Automation

7.7 Automation

- Describe automation as a value-add capability for relevant global health use cases

Key Performance Indicators:

- Availability - Stock out rate - Number of missed line item quantity deliveries due to stock out versus total line item quantity deliveries
- Availability - Stock levels - Current on hand stock versus planned on hand stock
- Safe product handling - Number of products handled during period out of conformance versus Number of products handled during period expressed as a % with prior period comparisons
- Customer Incidents Report number of incidents by month/week/day comparison with previous periods
- No of Order Lines shipped with incorrectly captured Items - Number of order lines with incorrect items/SKU's versus total number of order lines shipped as a %
- Regulatory - percentage of procured products that meet Stringent Regulatory Authority (SRA) or WHO standards
- Waste Management - Existence of supply chain management protocols for disposal of medical waste and management of unusable products
- Number of order lines delivered to incorrect end-user - Number of orders incorrectly addressed versus number of orders processed expressed as a %
- Number of Order Lines delivered out of Quality Specifications - Number of Item/SKU delivered out of specification e.g. Cold Chain infractions, Expired products versus total Number of order lines delivered as a %. A comparative graph would be an appropriate tool for the measurement of this metric.
- % Product Transferred On-Time to Demand Requirement. Actual Number of products transferred outside of the Time Limits vs Number of products Transferred.
- Transfer Product Cycle Time - Actual Transfer Cycle Time versus Planned Transferred Cycle Time
- Accurate pick - Picking Accuracy number of order lines picked inaccurately divided by the total order lines picked (SKU and Quantity)
- On-time delivery - Actual Delivery Date & Time Vs Promised Delivery Data and Time
- Customer order fulfilment cycle time & rate - Actual Order Fulfilment Cycle Time versus Promised Customer Order Fulfilment Cycle time
- Warehousing and Inventory Management OTIF - On time and in full
- Number of warehouse locations used versus warehouse locations available (Pallet Positions may be used in larger warehouses)
- Line Items SKU's handled vs Items / SKU's Planned over a planning horizon
- Warehouse Utilization - Volume used vs Volume Available

Training:

- Data management
- ERP Systems
- Legislation and Standards
- 3-way Receiving Match
- Code Handling/RFID (if available)
- Discrepancy Reporting and Resolution
- ID & Damage Inspection
- Inventory Management

Qualifications Available:

- (CILT) International Diploma in Logistics & Transport

Certifications Available:

- SCPro™ Certification
- APICS Certified in logistics transportation and distribution certificate
- Online Post Graduate Diploma in Global Health Procurement and Supply Chain Management

» Property Control and Disposition<br>» Return Management<br>» MSDS/CoC/BoL/Environmental Interpretation		

Officer – Procurement and Supply

Organisation: **Public Health Organisation – Clinic Service Delivery Level**				
Competency Level:	Serial Number:	Primary Process:	Job Role:	Supervises:
Operational	2-21-241-554-2	Procure	Officer – Procurement and Supply	-

Contributions	Executes the Procurements processes within the context as defined in the Operating Model of the Public Health System Organisation's Operating Model.
Technical Competency(ies):	2.1 Manage procurement costs and budget<br>2.4 Undertake contract management and risk<br>2.4.1 Place commodity orders

2.1 Manage procurement costs and budget

» Understand public procurement regulations

» Understand the importance of budget approval before initiating procurement

» Understanding the importance of financial and management accounting practices within procurement

» Understand the commercial factors that contribute toward cost reduction, price savings and value improvement opportunities

» Understand the principles of foreign exchange rates and how they impact prices

2.4 Undertake contract management

and risk

2.4.1 Place commodity orders

» Understand that authorisations are required to administer the contracts

» Understand the type of supplies required

» Understand the process of obtaining reference sources and supplier catalogues to clarify required product and its availability

» Understand the process to comply with policies and procedures to order required stock and equipment

» Understand the process of confirming the orders with suppliers

» Understand the process of identifying any problems in placing orders

» Understand the procedures for placing orders

» Examine any problems with order delivery and compare recommended options for progressing order delivery

» Enable the execution of effective mechanisms for checking invoices exist and take action to adjust payments accordingly

» Understand the process of receiving or raising requisitions and arranging purchase orders and payments

Personal/Management Competency(ies):	5.5 Manage and plan projects<br>5.5.4 Direct/participate in teamwork<br>6.1 Demonstrate generic skills (e.g., literacy, numeracy, technology)<br>6.1.1 Exhibit high understanding of literacy and numeracy<br>6.2 Demonstrate strong communication skills<br>6.2.1 Practice cultural awareness

5.5 Manage and plan projects

5.5.4 Direct/participate in teamwork

» Understand the need to work with team members within their area of authority to establish achievable goals and strategies that are consistent with the objectives established for the organisation as a whole

» Interpret an organisational chart that shows the lines of reporting and responsibility among staff

» Understand the need to actively contribute a perspective and making a positive contribution to team-based problem solving and decision making

» Understand the need to provide feedback, encouragement and support to team members for progressing strategic goals

» Understand the need to monitor team performance in relation to the organisation's mission and goals

» Understand the need for teams to think for themselves and resolve problems

» Understand the need to encourage and harness diversity within the team to boost team effectiveness

» Understand the need to encourage and support the team to work through its stages of development and perform well

» Understand roles and responsibilities in relation to employees' expertise and the expectations of collaborating team members

» Understand where their position fits in the structure (organisation, SC, healthcare sector) , the responsibilities their position entails and the accountabilities linked to the position

6.1 Demonstrate generic skills (e.g., literacy, numeracy, technology)

6.1.1 Exhibit high understanding of literacy and numeracy

» Speak clearly and confidently, organize information in a logical manner, and consider voice tone and pace

» Respond to verbal messages and other cues (active listening)

» Express ideas and opinions clearly in written and verbal form

» Communicate information accurately, concisely and confidently in writing and verbally

» Practice a level of mathematics suitable to the job held

» Compare visual presentation techniques, including charting, histograms and flow sheets
» Read and comprehend at a level necessary to properly complete duties of the position
» Understand the need to interpret written information in documents, such as reports, SOPs, LMIS forms, graphs, calendars, schedules, notices and directions
» Understand the need to pay attention to detail and identify the main ideas, detect inconsistencies and identify missing information in documents
» Enable computer skills — such as using Word, Excel, PowerPoint and the internet — suitable to the job level
» Understand the process of analysing and validating KPIs
» Understand how the evaluation process of activities that act as standards against which the KPIs are measured is carried out
» Examine the process of performing critical self-review on work before submission

6.2 Demonstrate strong communication skills

» Be truthful and trustworthy, and supply accurate information at all times
» Enable working as part of a workplace team
» Understand vocabulary and communication style and form for both written and verbal communication that is appropriate for the situation, audience and material being communicated (e.g., avoid unnecessary jargon, clearly explain medical and SCM terminology)
» Enable open communication within constraints of confidentiality
» Participate in the organisation of communications about logistics
» Enable participation in meetings, expressing one's opinions, being aware of others' needs, and being appropriately assertive when required
» Show perspective into the point of view of others, understanding their needs and goals
» Enable the practise of building trust, rapport and credibility with others
» Understand the communication needs and concerns of others and respond to them
» Understand the need to avoid conflict between work and personal interests
» Enable meaningful two-way communication
» Show influence on others by persuasively presenting thoughts and ideas
» Show assertiveness skills to deal with unreasonable requests and/or refusals that would compromise practice or consumer care
» Maintain a positive, supportive and appreciative attitude
» Show the ability to balance the organisation's needs and the customer's needs

	» Show self-control by maintaining composure and keeping emotions in check, even in difficult situations; deal calmly and effectively with stressful situations<br>» Enable expressing of opinions and providing information in written and/or verbal form in a manner that does not elicit concern, anger or other adverse response<br>» Enable the use of a systematic process for following up that demonstrates written reports have been received and understood<br>» Understand the means by which responses to input to the work environment are monitored<br>» Seek practical ways to overcome barriers to communication<br>» Understand the need to ensuring language representation (e.g., interpreters) at meetings when appropriate<br>» Interpret difficult situations and disputes between staff that need to be tackled and resolved<br>» Enable giving both positive and negative feedback sensitively<br>» Understand the need to maintain and improve communication<br>» Examine the process of giving and requesting feedback frequently<br>» Examine effective communication with shop stewards and union representatives
6.2.1 Practice cultural awareness	» Understand the need for cultural awareness and sensitivity, treating all people with fairness, respect and dignity<br>» Enable anti-discriminatory practices in the organisation, including HR and disciplinary procedures<br>» Enable cultural sensitivity, equality and fairness at all levels of the organisation<br>» Challenge discriminatory behaviour directly and sensitively<br>» Act in a non-discriminatory way toward individuals and groups<br>» Enable integrating cultural awareness in learning and development approaches<br>» Avoid stereotypical responses by examining one's own behaviour and bias<br>» Enable managing cultural diversity in teams and make the most of differences<br>» Compare strategies and/or resources for communicating effectively with people from different cultural backgrounds<br>» Enable the process to elicit information relating to values, beliefs and cultural backgrounds of consumers that may influence the way professional services are provided

Key Performance Indicators:
» Vendor on time delivery (VOTD)<br>» % of emergency orders issued in the last 12 months<br>» % of product selection based on National Essential Medicines List<br>» % of procured products that meet Stringent Regulatory Authority (SRA) or WHO standards<br>» Supply adequacy - Net requirements are reflected in the Procurement pipeline to secure sufficient future supply.<br>» Supply Plan report; supplier performance report<br>» Fill Rate vs Planned fill rate<br>» Identify, Prioritize, and Aggregate Supply Chain Requirements Cycle Time - Actual Time taken to determine net supply chain requirements by product and SKU vs Planned Time<br>» Order accuracy - Net requirement versus order quantities in the planning horizon<br>» Ratio between median price of pro- ducts procured and the international median reference value<br>» Availability - Stock out rate - Number of missed line item quantity deliveries due to stock out versus total line item quantity deliveries<br>» Availability - Stock levels - Current on hand stock versus Planned on hand stock<br>» Data accuracy - Vendor Master data accuracy, item / SKU Master Data accuracy<br>» Demand analysis - Actual Demand versus Planned Demand<br>» Forecast Accuracy - Using various measures of Forecast Error e.g. MAD<br>» Inventory Days of Supply versus Planned Inventory days of Supply<br>» Reports submitted on time (for all and separate reports)

Training:	Qualifications Available:	Certifications Available:
» Procurement<br>» MSDS/CoC/BoL/Environmental Interpretation<br>» Logistics Management<br>» Delivery Balancing<br>» Forecasting<br>» Master Scheduling<br>» Order Management<br>» Prioritization		» CIPS Level 3 Advanced Certificate in Procurement and Supply Operations

Public Health Organisation – Community Service Delivery Level

Tactical

Clerk – Supply

Organisation:

Public Health Organisation – Community Service Delivery Level

Competency Level:	Serial Number:	Primary Process:	Job Role:	Supervises:
Tactical	3-32-241-61-1	Procure	Clerk – Supply	-

Contributions	Timeous recording of receipts,
Technical Competency(ies):	2.4 Undertake contract management and risk<br>2.4.1 Place commodity orders<br>3.2 Supply commodities to facilities

2.4 Undertake contract management and risk	
2.4.1 Place commodity orders	» Awareness that authorisations are required to administer the contracts<br>» Awareness of the type of supplies required<br>» Awareness of the process of obtaining reference sources and supplier catalogues to clarify required product and its availability<br>» Awareness of the need to comply with policies and procedures to order required stock and equipment<br>» Awareness of the process of confirming the orders with suppliers<br>» Awareness that any problems in placing orders need to be identified<br>» Awareness of the procedures for placing orders<br>» Awareness of how to identify any problems with order delivery and recommended options for progressing order delivery<br>» Awareness of effective mechanisms for checking invoices exist and take action to adjust payments accordingly<br>» Awareness the process of receiving or raising requisitions and arranging purchase orders and payments
3.2 Supply commodities to facilities	» Working knowledge of the various sources of distribution demand (customers)<br>» Basic operational knowledge of order policies for planned order generation

	» Execute re-supply quantities using a variety of inventory methods, including visual review, two bin, periodic review, order point and just in time<br>» Basic knowledge of the use of order schedules<br>» Basic operational knowledge of safety stocks<br>» Awareness of the principles of EOQ in the ordering process<br>» Awareness of pull and push inventory control systems<br>» Execute a regular, rather than urgent, order culture<br>» Execute order-filling priorities in relation to delivery opportunities and urgency<br>» Awareness that orders must be screened (modify order quantities on the basis of available stock, impact on service delivery, distance of facility from hospital)<br>» Assemble, check, pick and pack orders<br>» Execute dispatch lists and invoices<br>» Awareness of the importance of the balancing of supply with demand, considering both lead time and demand variability created by supply patterns not matching demand patterns<br>» Basic operational knowledge on the storage locations and facilities<br>» Awareness of the measures of customer satisfaction by using performance metrics taken from the customer perspective, with criteria such as on-time delivery, perception of quality, complaints and length of wait times
Personal/Management Competency(ies):	6.1 Demonstrate generic skills (e.g., literacy, numeracy, technology)<br>6.1.1 Exhibit high understanding of literacy and numeracy<br>6.4 Exhibit professional and ethical values<br>6.4.1 Demonstrate integrity<br>6.4.2 Engage in continuous professional development
6.1 Demonstrate generic skills (e.g., literacy, numeracy, technology)	»
6.1.1 Exhibit high understanding of literacy and numeracy	» Speak clearly and confidently, organize information in a logical manner, and consider voice tone and pace<br>» Respond to verbal messages and other cues (active listening)<br>» Express ideas and opinions clearly in written and verbal form<br>» Communicate information accurately, concisely and confidently in writing and verbally<br>» Practice a level of mathematics suitable to the job held<br>» Awareness of visual presentation techniques, including charting, histograms and flow sheets<br>» Read and comprehend at a level necessary to properly complete duties of the position

- Awareness of the need to interpret written information in documents, such as reports, SOPs, LMIS forms, graphs, calendars, schedules, notices and directions
- Awareness of the need to paying attention to detail and identifying the main ideas, detecting inconsistencies and identifying missing information in documents
- Execute computer skills — such as using Word, Excel, PowerPoint and the internet — suitable to the job level
- Operational knowledge of analysing and validating KPIs
- Awareness of how the evaluation process of activities that act as standards against which the KPIs are measured is carried out
- Execute the process of performing critical self-review on work before submission

6.4 Exhibit professional and ethical values

6.4.1 Demonstrate integrity

- Accept responsibility for one's own work tasks and performance
- Awareness of the need to work within a framework of clearly understood humanitarian values and ethics
- Stand by decisions and hold others accountable when necessary
- Does not abuse one's own power or position
- Awareness of the process to manage team members to ensure they do not abuse their power or position
- Realize when individuals or the organisation is straying from organisation goals, and challenge them to uphold ethics
- Awareness of the need to resist undue political pressure in decision making
- Awareness of the need to support staff in maintaining ethical stances
- Realize the need to make time in team for ethical inquiry and reflection
- Awareness of the need to show consistency between expressed principles and behaviour
- Awareness of principles, values and ethics that are embedded in policy
- Realize the need to act without consideration of personal gain
- Awareness of the need for transparency in decision making structures and processes
- Awareness of the need to ensure programmes are acting with integrity, and recognising the impact of not doing so
- Recognise one's own limitations and act upon them
- Execute all standard operating procedures
- Execute the practise of working in a safe and legal way
- Demonstrate respect, dignity and consideration for consumers
- Awareness of the impact of a no-blame culture on reporting and preventing recurrence of incidents
- Awareness of requests of colleagues that might be regarded as unreasonable

- Awareness of the need to communicate (verbally and by example) expectations of the desired standards and approaches to be adopted
- Awareness of roles and responsibilities in terms of the position statement/duty statement of the position held
- Demonstrate punctuality
- Awareness of the obligation to apply professional care and expertise to deliver high quality of services
- Demonstrate care and attention to detail in undertaking work activities
- Demonstrate appropriate attire and presentation for the role and situation
- Recognise and take responsibility for emotions
- Awareness of the need to an environment in which others can talk and act without fear of repercussion
- Seek to keep commitments and not let people down
- Be trusting and cooperative when working alongside others
- Take on various responsibilities within the department as the need arises

6.4.2 Engage in continuous professional development

- Seek out opportunities to learn, and integrate new knowledge and skills into work
- Awareness of gaps in knowledge and skills for completing specific tasks, and develop a plan to acquire them
- Listen to and invite feedback from others on one's own performance
- Use lifelong learning (continuous striving to gain knowledge and maintain competence) in the context of career development and the professional's role in delivering healthcare services
- Awareness of organisational systems for capturing learning and ensuring lessons learned
- Awareness of the need for reflective learning within the team, in which lessons are captured and integrated into future projects
- Awareness that continuous learning is an integral part of organisational performance
- Be open to new ideas and different perspectives
- Keep up to date in the place of work with input from supervisors
- Awareness of the need to document continual professional development activities
- Awareness of multiple techniques to acquire new knowledge and skills; process and retain information; and identify when it is necessary to acquire new knowledge and skills.
- Awareness of professional organisations and/or committees
- Observe and/or execute quality improvement and/or quality assurance activities participated in
- Reflect on performance and seek what needs improvement
- Awareness of personal career development, identifying occupational interests, strengths, options and opportunities

	» Awareness of the need to contribute to others' professional development<br>» Share experiences and learning internally and externally<br>» Awareness of the need to seek out challenging projects outside of core experience and achieve solid results
Basal Technology Competency(ies):	7.4 Temperature and monitoring<br>7.6 ERP includes function of LMIS<br>7.12 Basic Office Skills<br>7.13 Have a command of technology
7.4 Temperature and monitoring	» Awareness of the current technology approaches for Temperature monitoring and sensors<br>» Awareness of what is required to implement this technology in a country context<br>» Awareness of infrastructure requirement for heat-sensitive items at peripheral 'lower level' of SC depending on volume and nature of items to handle;<br>» Awareness of regular preventive maintenance and calibration of cold-chain equipment with biomedical engineers or logisticians<br>» Awareness of the policy for temperature monitoring tools during transportation and storage<br>» Awareness of the steps involved in health logistic management cycle: selection-order-delivery-reception-storage-distribution-inventory<br>» Awareness of hardware qualification and system validation of temperature monitoring<br>» Basic operational knowledge mass data management of temperature monitoring
7.6 ERP includes function of LMIS	» Awareness of a Master Data Management System<br>» Awareness of the critical components of an ERP system<br>» Awareness of the meaning of common document types found within an ERP/LMIS<br>» Awareness of the logic of data visibility within an ERP<br>» Awareness of the importance of data integrity, record management and information security
7.12 Basic Office Skills	» Have a good understanding of common presentation authoring packages<br>» Have a good understanding of common spreadsheet authoring packages<br>» Have a good understanding of common document authoring packages<br>» Have a good understanding of common email authoring packages<br>» Have a good understanding of common instant messaging packages

	» Have a good understanding of common video conferencing authoring packages
7.13 Have a command of technology	» Execute the use of technology suitable to the job held<br>» Awareness of the need to monitor new developments and technologies in the sector<br>» Awareness of new technologies and recognising potential benefits for the sector<br>» Awareness that resources and support are provided across the organisation to enable colleagues to make the best use of available technology<br>» Awareness of the fact that the organisation has a strategy for technology use<br>» Awareness of how to minimise environmental damage through technology use<br>» Basic operational knowledge of technology to maximise effectiveness and efficiency<br>» Basic operational knowledge of field-based technology (e.g., radio, general packet radio service (GPRS), satellite phone)
Advanced Technology Competency(ies):	7.7 Automation
7.7 Automation	» Awareness of automation as a value-add capability for relevant global health use cases

Key Performance Indicators:

- » Receipt accuracy - Number of receipts incorrectly recorded versus total number of receipts recorded
- » Put away accuracy. Number of items incorrectly put away in store or warehouse versus total number of put away transactions
- » Inspection accuracy- Number of items put away with damaged or non-conformant to specifications versus total number of items put away
- » Adherence to warehouse binning rules. Number of items misplaced versus total number of items placed in warehouse/store

Training:	Qualifications Available:	Certifications Available:
» ERP Systems<br>» Legislation and Standards<br>» 3-way Receiving Match<br>» Code Handling/RFID (if available)<br>» Discrepancy Reporting and Resolution<br>» ID & Damage Inspection		» Dispatch stock in a DC warehouse skills programme<br>» Occupational Certificate: Store Person

» Property Control and Disposition		

Organisation:

Public Health Organisation – Community Service Delivery Level

Competency Level:	Serial Number:	Primary Process:	Job Role:	Supervises:
Tactical	3-31-25-1	Procure	Clerk – Receiving and Storage	-

Contributions	Accepts goods received and performs put away operations in compliance with the rules governing the organisation of inventory on clinic shelves.
Technical Competency(ies):	2.5 Ensure quality of products<br>3.1 Undertake storage, warehousing and inventory management<br>3.1.1 Manage storage of commodities during emergency<br>3.5 Manage disposal of products (e.g. expired, damaged, redundant products)<br>3.5.1 Define and direct process for managing redundant and returned stock<br>3.5.2 Manage process for disposal of returned stock<br>3.7 Manage re-packing of products

2.5 Ensure quality of products

» Basic operational knowledge on how to use and monitor the process for prequalification of suppliers

» Execute the process of ensuring that health commodities are not counterfeit and meet quality standards through certification (ISO, WHO GMP

» Awareness of the process of implementing, conducting and maintaining a reporting system of pharmacovigilance (e.g. report adverse drug reactions, quality defects, batch recall) and equipment faults

» Inspect products when delivered and during storage to catch defects or problems before they are given to, or needed by, clients

» Execute compliance with good distribution and good storage practices (temperature monitoring in the warehouse, transportation and distribution)

» Awareness of product-specific shelf-life by which the product are acceptable or rejected (e.g. no less than 1 year for most of drugs, and less for short shelf-life like laboratory)

3.1 Undertake storage, warehousing and inventory management

» Execute distribution of products among suppliers, distribution centres, warehouses and customers through a logistics network

» Be aware of a total systems approach to designing and managing the entire flow of information, materials, and services

» Awareness of the choice of equipment or materials based on suitability for intended use, accuracy, safety of use and cost

» Awareness of the formal storage locations in the warehouse that identify the row, rack section, level and shelf location, typically with an alphanumeric location bar code or label

	» Store medicines appropriately, considering temperature, access and cleanliness<br>» Ability to maintain the cold chain<br>» Execute the movement and storage of materials within a warehouse<br>» Be aware of the need to secure the medical store and limit access to staff<br>» Follow organisation processes, instructions, rules and parameters for warehouse and inventory management and use of equipment (e.g., annual stock turns, expiry date tracking, stock procurement, rotation and retrieval, equipment maintenance)<br>» Awareness of policies and procedures for stock handling, distribution and withdrawal consistent with maintaining safety (e.g., policies for distribution of concentrated electrolyte solutions and cytotoxics, identification of cytotoxic drug products within the work environment, separation of like-named or like-packaged products)<br>» Confirm information on the supplies being stored<br>» Awareness that one must consolidate several items into larger units for fewer handlings<br>» Awareness of methods of stock rotation (e.g., first in first out, FIFO, or first to expire first out, FEFO)<br>» Enter the quantity received and update the warehouse/inventory/LMIS system software with the stock on hand when each order is delivered according to requisite SOP's<br>» Awareness that there are required levels of stock and/or equipment in specific situations<br>» Awareness of the importance of accurate verification of rolling stocks<br>» Awareness of the importance of monitoring the location and condition of the supplies being stored<br>» Awareness of the importance of the information on the storage locations and facilities<br>» Awareness of the need to communicate any problems in using the storage locations and facilities
3.1.1 Manage storage of commodities during emergency	» Awareness of the types of warehouses available for emergency or disaster supply<br>» Awareness of the choice of storage site for emergency or disaster supply<br>» Awareness of the staff required for the storage of emergency or disaster supply<br>» Awareness of the equipment and material required for the warehousing of emergency or disaster supply<br>» Awareness of the need for a rapid assessment of logistical needs in emergencies
3.5 Manage disposal of products (e.g. expired, damaged, redundant products)	

3.5.1 Define and direct process for managing redundant and returned stock	» Take down information on the supplies being returned.<br>» Basic operational knowledge of the flow of returned supplies<br>» Awareness of any problems with the flow of returned supplies<br>» Awareness of the various options for improving the flow of returned supplies<br>» Awareness of procedures for monitoring the flow of returned supplies<br>» Awareness of the recall procedures to be used in response to a product recall notice or to access the information promptly.<br>» Execute action required in response to a product recall notice<br>» Awareness of reporting and the procedures for following up on medication incidents<br>» Realize follow-up strategies likely to be effective in preventing recurrence (e.g., root cause analysis)<br>» Execute an integrated healthcare waste management plan<br>» Follow guides and procedures for collection, sorting, transport and disposal<br>» Operational knowledge of pre-disposal inspections<br>» Operational knowledge of the prerequisites for products disposal<br>» Operational knowledge of the product disposal report
3.5.2 Manage process for disposal of returned stock	» Execute the disposal of expired medications and/or medical equipment according to national policy<br>» Awareness of a recording system for disposal<br>» Execute the disposal of specific individual items of greatest risk with appropriate care (e.g., oncology medicine)
3.7 Manage re-packing of products	» Execute re-packing or pre-packing<br>» Execute a safe system for repacking
Personal/Management Competency(ies):	6.1 Demonstrate generic skills (e.g., literacy, numeracy, technology)<br>6.1.1 Exhibit high understanding of literacy and numeracy<br>6.2 Demonstrate strong communication skills<br>6.2.1 Practice cultural awareness<br>6.4 Exhibit professional and ethical values<br>6.4.1 Demonstrate integrity<br>6.4.2 Engage in continuous professional development
6.1 Demonstrate generic skills (e.g., literacy, numeracy, technology)	
6.1.1 Exhibit high understanding of literacy and numeracy	» Speak clearly and confidently, organize information in a logical manner, and consider voice tone and pace<br>» Respond to verbal messages and other cues (active listening)<br>» Express ideas and opinions clearly in written and verbal form

- Communicate information accurately, concisely and confidently in writing and verbally
- Practice a level of mathematics suitable to the job held
- Awareness of visual presentation techniques, including charting, histograms and flow sheets
- Read and comprehend at a level necessary to properly complete duties of the position
- Awareness of the need to interpret written information in documents, such as reports, SOPs, LMIS forms, graphs, calendars, schedules, notices and directions
- Awareness of the need to paying attention to detail and identifying the main ideas, detecting inconsistencies and identifying missing information in documents
- Execute computer skills — such as using Word, Excel, PowerPoint and the internet — suitable to the job level
- Operational knowledge of analysing and validating KPIs
- Awareness of how the evaluation process of activities that act as standards against which the KPIs are measured is carried out
- Execute the process of performing critical self-review on work before submission

6.2 Demonstrate strong communication skills

- Be truthful and trustworthy, and supply accurate information at all times
- Realize the need to work as part of a workplace team
- Awareness of vocabulary and communication style and form for both written and verbal communication that is appropriate for the situation, audience and material being communicated (e.g., avoid unnecessary jargon, clearly explain medical and SCM terminology)
- Awareness of open communication within constraints of confidentiality
- Awareness of communications about logistics
- Participate in meetings, expressing one's opinions, being aware of others' needs, and being appropriately assertive when required
- Awareness of perspective into the point of view of others, understanding their needs and goals
- Build trust, rapport and credibility with others
- Awareness of the communication needs and concerns of others and respond to them
- Awareness of the need to avoid conflict between work and personal interests
- Practice meaningful two-way communication
- Realize that others are influenced by persuasively presenting thoughts and ideas
- Awareness of the need for assertiveness skills to deal with unreasonable requests and/or refusals that would compromise practice or consumer care
- Maintain a positive, supportive and appreciative attitude

	» Awareness of the need to balance the organisation’s needs and the customer’s needs<br>» Execute self-control by maintaining composure and keeping emotions in check, even in difficult situations; deal calmly and effectively with stressful situations<br>» Express opinions and provide information in written and/or verbal form in a manner that does not elicit concern, anger or other adverse response<br>» Basic operational knowledge of a systematic process for following up that demonstrates written reports have been received and understood<br>» Basic operational knowledge of the means by which responses to input to the work environment are monitored<br>» Seek practical ways to overcome barriers to communication<br>» Awareness of the need for language representation (e.g., interpreters) at meetings when appropriate<br>» Realize that difficult situations and disputes between staff need to be tackled and resolved<br>» Realize the need to give both positive and negative feedback sensitively<br>» Awareness of the need to maintain and improve communication<br>» Execute the process of giving and requesting feedback frequently<br>» Execute effective communication with shop stewards and union representatives
6.2.1 Practice cultural awareness	» Awareness of the need for cultural awareness and sensitivity, treating all people with fairness, respect and dignity<br>» Awareness of the anti-discriminatory practices in the organisation, including HR and disciplinary procedures<br>» Awareness of the need for cultural sensitivity, equality and fairness at all levels of the organisation<br>» Challenge discriminatory behaviour directly and sensitively<br>» Act in a non-discriminatory way toward individuals and groups<br>» Awareness of the need for integrating cultural awareness in learning and development approaches<br>» Avoid stereotypical responses by examining one’s own behaviour and bias<br>» Awareness of the need to manage cultural diversity in teams and make the most of differences<br>» Awareness of strategies and/or resources for communicating effectively with people from different cultural backgrounds<br>» Awareness of the need to elicit information relating to values, beliefs and cultural backgrounds of consumers that may influence the way professional services are provided
6.4 Exhibit professional and ethical values	

6.4.1 Demonstrate integrity

- » Accept responsibility for one's own work tasks and performance
- » Awareness of the need to work within a framework of clearly understood humanitarian values and ethics
- » Stand by decisions and hold others accountable when necessary
- » Does not abuse one's own power or position
- » Awareness of the process to manage team members to ensure they do not abuse their power or position
- » Realize when individuals or the organisation is straying from organisation goals, and challenge them to uphold ethics
- » Awareness of the need to resist undue political pressure in decision making
- » Awareness of the need to support staff in maintaining ethical stances
- » Realize the need to make time in team for ethical inquiry and reflection
- » Awareness of the need to show consistency between expressed principles and behaviour
- » Awareness of principles, values and ethics that are embedded in policy
- » Realize the need to act without consideration of personal gain
- » Awareness of the need for transparency in decision making structures and processes
- » Awareness of the need to ensure programmes are acting with integrity, and recognising the impact of not doing so
- » Recognise one's own limitations and act upon them
- » Execute all standard operating procedures
- » Execute the practise of working in a safe and legal way
- » Demonstrate respect, dignity and consideration for consumers
- » Awareness of the impact of a no-blame culture on reporting and preventing recurrence of incidents
- » Awareness of requests of colleagues that might be regarded as unreasonable
- » Awareness of the need to communicate (verbally and by example) expectations of the desired standards and approaches to be adopted
- » Awareness of roles and responsibilities in terms of the position statement/duty statement of the position held
- » Demonstrate punctuality
- » Awareness of the obligation to apply professional care and expertise to deliver high quality of services
- » Demonstrate care and attention to detail in undertaking work activities
- » Demonstrate appropriate attire and presentation for the role and situation
- » Recognise and take responsibility for emotions
- » Awareness of the need to an environment in which others can talk and act without fear of repercussion
- » Seek to keep commitments and not let people down

	» Be trusting and cooperative when working alongside others<br>» Take on various responsibilities within the department as the need arises
6.4.2 Engage in continuous professional development	» Seek out opportunities to learn, and integrate new knowledge and skills into work<br>» Awareness of gaps in knowledge and skills for completing specific tasks, and develop a plan to acquire them<br>» Listen to and invite feedback from others on one's own performance<br>» Use lifelong learning (continuous striving to gain knowledge and maintain competence) in the context of career development and the professional's role in delivering healthcare services<br>» Awareness of organisational systems for capturing learning and ensuring lessons learned<br>» Awareness of the need for reflective learning within the team, in which lessons are captured and integrated into future projects<br>» Awareness that continuous learning is an integral part of organisational performance<br>» Be open to new ideas and different perspectives<br>» Keep up to date in the place of work with input from supervisors<br>» Awareness of the need to document continual professional development activities<br>» Awareness of multiple techniques to acquire new knowledge and skills; process and retain information; and identify when it is necessary to acquire new knowledge and skills.<br>» Awareness of professional organisations and/or committees<br>» Observe and/or execute quality improvement and/or quality assurance activities participated in<br>» Reflect on performance and seek what needs improvement<br>» Awareness of personal career development, identifying occupational interests, strengths, options and opportunities<br>» Awareness of the need to contribute to others' professional development<br>» Share experiences and learning internally and externally<br>» Awareness of the need to seek out challenging projects outside of core experience and achieve solid results
Basal Technology Competency(ies):	7.4 Temperature and monitoring<br>7.6 ERP includes function of LMIS<br>7.12 Basic Office Skills<br>7.13 Have a command of technology
7.4 Temperature and monitoring	» Awareness of the current technology approaches for Temperature monitoring and sensors

	» Awareness of what is required to implement this technology in a country context<br>» Awareness of infrastructure requirement for heat-sensitive items at peripheral 'lower level' of SC depending on volume and nature of items to handle;<br>» Awareness of regular preventive maintenance and calibration of cold-chain equipment with biomedical engineers or logisticians<br>» Awareness of the policy for temperature monitoring tools during transportation and storage<br>» Awareness of the steps involved in health logistic management cycle: selection-order-delivery-reception-storage-distribution-inventory<br>» Awareness of hardware qualification and system validation of temperature monitoring<br>» Basic operational knowledge mass data management of temperature monitoring
7.6 ERP includes function of LMIS	» Awareness of a Master Data Management System<br>» Awareness of the critical components of an ERP system<br>» Awareness of the meaning of common document types found within an ERP/LMIS<br>» Awareness of the logic of data visibility within an ERP<br>» Awareness of the importance of data integrity, record management and information security
7.12 Basic Office Skills	» Have a good understanding of common presentation authoring packages<br>» Have a good understanding of common spreadsheet authoring packages<br>» Have a good understanding of common document authoring packages<br>» Have a good understanding of common email authoring packages<br>» Have a good understanding of common instant messaging packages<br>» Have a good understanding of common video conferencing authoring packages
7.13 Have a command of technology	» Execute the use of technology suitable to the job held<br>» Awareness of the need to monitor new developments and technologies in the sector<br>» Awareness of new technologies and recognising potential benefits for the sector<br>» Awareness that resources and support are provided across the organisation to enable colleagues to make the best use of available technology<br>» Awareness of the fact that the organisation has a strategy for technology use<br>» Awareness of how to minimise environmental damage through technology use

	» Basic operational knowledge of technology to maximise effectiveness and efficiency<br>» Basic operational knowledge of field-based technology (e.g., radio, general packet radio service (GPRS), satellite phone)
Advanced Technology Competency(ies):	7.7 Automation
7.7 Automation	» Awareness of automation as a value-add capability for relevant global health use cases

Key Performance Indicators:

- Safe product handling - Number of products handled during period out of conformance versus Number of products handled during period expressed as a % with prior period comparisons
- Customer Incidents Report number of incidents by month/week/day comparison with previous periods
- No of order lines shipped with incorrectly captured Items - Number of order lines with incorrect items/SKU's versus total number of order lines shipped as a %
- Number of order lines delivered to incorrect end-user - Number of orders incorrectly addressed versus number of orders processed expressed as a %
- Number of order lines delivered out of Quality Specifications - Number of Item/SKU delivered out of specification e.g. Cold Chain infractions, Expired products versus total Number of order lines delivered as a %. A comparative graph would be an appropriate tool for the measurement of this metric.

Training:	Qualifications Available:	Certifications Available:
» Inventory Management<br>» Order Management<br>» Documentation Management		» Occupational Certificate: Store Person<br>» Describe the use of stores and warehousing in operations skills programme<br>» Occupational Certificate: Dispatching and Receiving Clerk

PEOPLE THAT DELIVER

People that Deliver

@Pplthatdeliver
