# Supplementary material for: Presenting a Framework to Professionalize Health Supply Chain Management
Source: Glob Health Sci Pract. 2025 May 9;13(Suppl 1):e2300119. doi: 10.9745/GHSP-D-23-00119 (PMC12063754; doi:10.9745/GHSP-D-23-00119)
Supplement: GHSP-D-23-00119-Meier-Supplement6.pdf [file GHSP-D-23-00119-Meier-Supplement6.pdf]

Supplement to: Brown AN, Chovitz B, dos Santos R, et al. Presenting a framework to professionalize health supply chain management. *Glob Health Sci Pract*. 2024;12(Suppl 3):e2300119. https://doi.org/10.9745/GHSP-D-23-00119

Supplement 6

Annex 1. List of organizations interviewed

1. DSV - Global Transport and Logistics
2. Global Fund
3. International Association of Public Health Logisticians
4. Pamela Steele Associates
5. People that Deliver (PtD)
6. Public Health Supply Chain Initiative, Association for Supply Chain Management (ASCM)
7. United Pharmaceutical Distributors (UPD)
8. USAID
9. USAID Global Health Supply Chain Program-Procurement and Supply Management (GHSC-PSM) project, Rwanda
10. Village Reach

Annex 2. Interview Data Collection Tool

1. Do you think that it is important for countries to define the competencies required by different levels of SCM staff?

2. Do you think it is important to define the SCM education requirements required to meet agreed-to SCM competencies?

3. Would a "professional framework standard" that could be contextualized to specific country contexts assist countries to do this?

4. Should such a framework cover both the private and public sector?

5. Do you think that an international professional association is the right entity to hold that standard at the global level?

6. What is required in countries to implement such a professional standard for those working in HSCM?

7. What tools and/or guidance is required to assist countries in implementing such a standard for those working in HSCM?

8. What barriers would you see in implementing such a professional standard for those working in HSCM?

9. How could these barriers be overcome?

10. Can you recommend any other input works that we should consider?

11. Does your country or organization use an internal supply chain competency framework that governs recruitment and upskilling activities?

Annex 3. List of organizations participating in data validation workshop

1. CLX
2. Guidehouse
3. National Department of Health South Africa
4. People that Deliver (PtD)
5. SAPICS
6. USAID
7. USAID Global Health Supply Chain Program-Procurement and Supply Management (GHSC-PSM)
8. Village Reach
